# Growth-phase-dependent control of rRNA synthesis in *Saccharomyces cerevisiae*

**DOI:** 10.1128/msphere.00493-24

**Published:** 2024-10-03

**Authors:** Catarina A. Mendes Felgueira, David A. Schneider

**Affiliations:** 1 Department of Biochemistry and Molecular Genetics, University of Alabama at Birmingham, Birmingham, Alabama, USA; NC State University, Raleigh, North Carolina, USA

**Keywords:** transcription, growth phase, ribosome, mRNA, rRNA

## Abstract

*Saccharomyces cerevisiae* is one of the most well-studied model organisms used in the scientific community. Its ease of manipulation, accessible growth conditions, short life cycle, and conserved eukaryotic metabolic pathways make it a useful model organism. Consequently, yeast has been used to investigate a myriad of phenomena, from microbial to human studies. Most of the research performed using this model organism utilizes yeast cell populations when they are growing exponentially, a growth phase aptly termed exponential or log phase. However, log phase encompasses several yeast generations and ranges several hours of yeast growth, meaning that there is a potential for variability during this “homogenous” growth phase. Cells in log phase require robust ribosome biogenesis to support their rapid growth and cell division. Interestingly, during log phase, ribosomal RNA (rRNA) synthesis (which is the first and rate limiting step in ribosome biosynthesis) has been shown to decrease prior to growth rate decline in stationary phase. In this study, we utilized several genomic and biochemical methods to elucidate the relationship between subphases of log phase and rRNA synthesis. Our results indicate that as yeast cells progress through subphases of log growth, both polymerase I transcription and rRNA processing are repressed. Overall, this study establishes a growth-phase-dependent control of rRNA synthesis that unexpectedly begins prior to the switch to stationary phase (i.e., pre-diauxic shift) as a putative mechanism of anticipating nutrient starvation.

**IMPORTANCE:**

*Saccharomyces cerevisiae* is a ubiquitously used model organism in a wide range of scientific research fields. The conventional practice when performing yeast studies is to investigate its properties during logarithmic growth phase. This growth phase is defined as the period during which the cell population doubles at regular intervals, and nutrients are not limiting. However, this growth phase lasts hours and encompasses several yeast cell generations which consequently introduce heterogeneity to log growth phase depending on their time of harvest. This study reveals significant changes in the transcriptomic landscape even in early stages of exponential growth. The overall significance of this work is the revelation that even the seemingly homogenous log growth phase is far more diverse than was previously believed.

## INTRODUCTION

The budding yeast *Saccharomyces cerevisiae* is one of the most commonly used eukaryotic model organisms. This ubiquitous organism replicates quickly, is inexpensive to grow, and is genetically simpler than multicellular organisms. Moreover, despite its divergence from other organisms of interest, such as humans, yeast shares many central metabolic pathways and regulatory mechanisms with higher organisms, making it an excellent model system (1, 2).

*S. cerevisiae* has been used for a wide range of studies, from establishing genetic manipulation techniques to defining drug mechanisms to studying cell aging (2, 3). Irrespective of the research topic, authors most frequently work with yeast cells while they are growing exponentially, and their doubling time is constant—a growth phase designated as log phase. This is an important distinction since microbial growth has four total phases: lag (characterized by slow growth and metabolic changes to adapt to a new environment), log (exponential growth), stationary (characterized by a decline and growth rate toward a plateau), and death (characterized by a population death rate higher than the growth rate). However, the exact timepoint within log phase used varies widely within the literature since log phase lasts for several population doubling events.

Despite being a defined fungal growth phase, log phase encompasses several changes in yeast biological processes. For example, Warner and Ju demonstrated that rRNA synthesis is repressed in exponentially growing yeast cells (4). This counterintuitive observation contradicts the suggestion that proliferating organisms require higher amounts of ribosomes and, in turn, rRNA to be produced (5). It has been proposed that this decrease in rRNA synthesis is indicative of cells anticipating nutrient limitations through a quorum-sensing mechanism (6).

The synthesis of rRNA is carried out by RNA polymerase I (Pol I) and is the first and rate-limiting step in ribosome biogenesis. The observed decrease in rRNA produced by exponentially growing yeast cells could be a result of changes in several biological process, including lower Pol I transcription, altered pre-rRNA processing, and/or rRNA degradation. Whether one or more of these processes is altered during the progression through log phase remains to be elucidated.

In this study, we utilized genomic and biomolecular techniques to interrogate how Pol I transcription and rRNA processing are altered within different subphases of log phase (early, mid, and late log phase). The changes in the transcriptomic landscape between each of the defined subphases have implications on both the mechanism by which yeast cells repress rRNA synthesis in log phase as well as on many other cellular processes that remain to be interrogated. Knowing the transcriptomic landscape of each of the subphases will better inform future yeast studies on what subphase of log growth to utilize for their specific experiment.

Given the observed decrease in rRNA synthesis within log phase, we hypothesized that as yeast cells progress through log phase, both Pol I transcription and rRNA processing are repressed. We observed that as yeast cells progress from early to mid to late log phase, Pol I occupancy on the rDNA was reduced, and Pol I pausing preference was significantly changed. We also found that hundreds of genes, transcribed by RNA polymerase II, were differentially regulated between the three subphases studied here. Notably, genes associated with rRNA processing, such as *UTP*s, were downregulated in the later subphases of log growth. Finally, we found that pre-rRNA processing is reorganized during log phase, resulting in the accumulation of precursor molecules at later stages of growth. These results suggest that Pol I transcription and rRNA processing contribute to the dynamic control of ribosome biosynthesis during progression through the log phase.

## RESULTS

### Pol I occupancy on the rDNA decreases with the progression of log phase

Most commonly, yeast growth phases are determined by measuring optical density at 600 nm (OD_600_) via spectrophotometer, with higher OD_600_ values indicating higher cell density. In general, yeast samples obtained during log phase are those with an OD_600_ measurement ranging from 0.25 to 1.0. This is a rather large range that encompasses several yeast cell generations and various metabolic changes. Our lab has previously investigated an unexpected metabolic change in which rRNA synthesis is repressed while cells are rapidly growing (6). The study found that yeast exhibited quorum-sensing-like behaviors for which the mechanism remains unknown. To investigate the changes in the transcriptomic landscape of yeast cells as they progress through log phase, we divided the conventional log phase into three subphases. The first—early log phase—includes yeast cultures of OD_600_ = 0.25–0.4. The second—mid log phase—includes yeast cultures of OD_600_ = 0.5–0.75. The third—late log phase—includes yeast cultures of OD_600_ = 0.8–1.00.

We first interrogated Pol I occupancy on the rDNA in these subphases using native elongating transcript sequencing (NET-seq). This technique allows us to map Pol I to the rDNA at single-nucleotide resolution by analyzing the last incorporated nucleotide into the nascent RNA. Originally designed to probe for Pol II occupancy, NET-seq has been optimized by our lab to evaluate Pol I occupancy (7–11).

**Fig 1:**
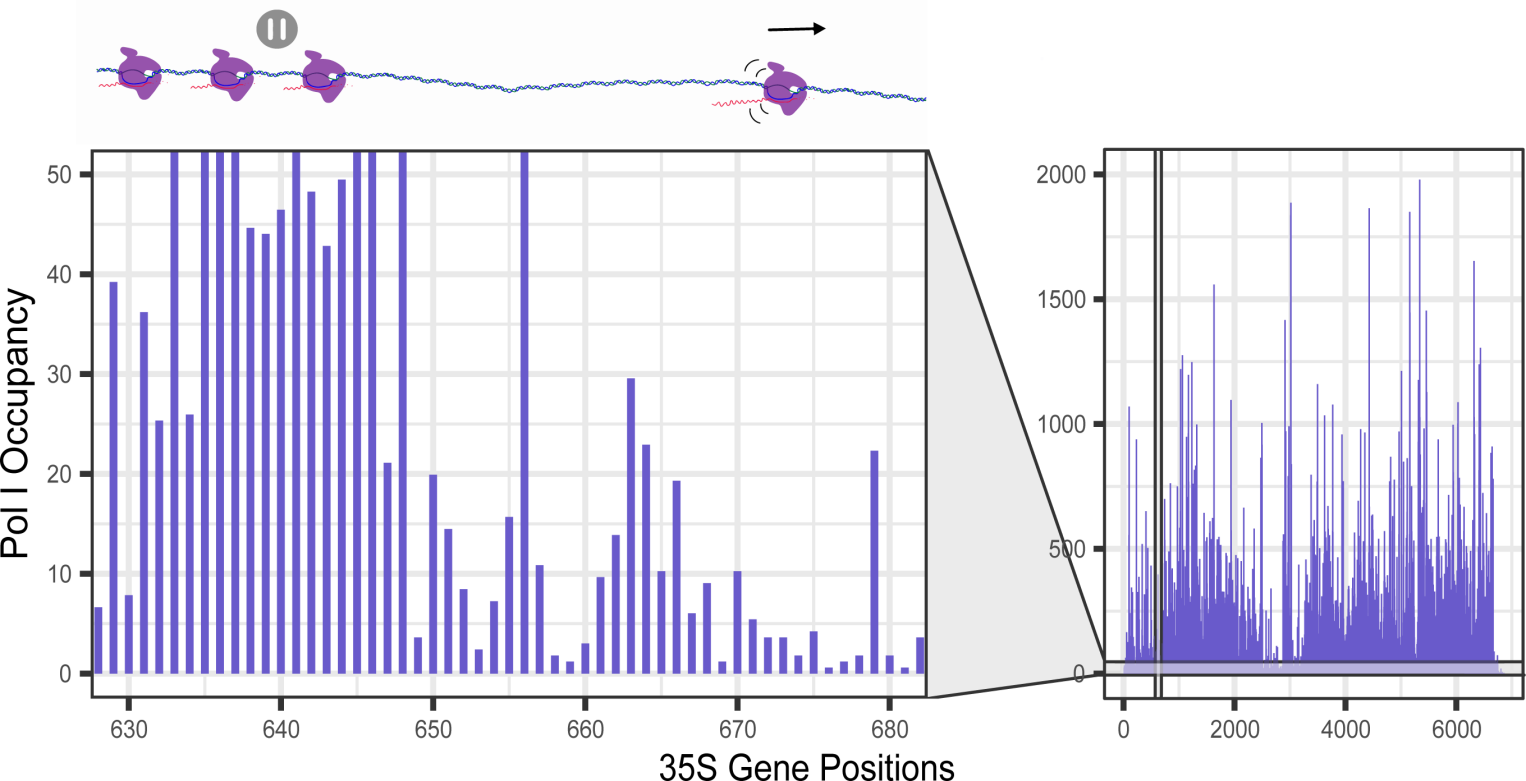
Native elongating transcript sequencing provides insight into transcription elongation. Schematic of NET-seq Pol I occupancy plot. Generated NET-seq reads are mapped to the rDNA (35S gene), and 5′ end of each read is then used for plotting the last incorporated nucleotide. This allows us to plot Pol I occupancy at each rDNA nucleotide position. We can then observe overall occupancy difference between samples and, also, specific areas (and nucleotides) of the gene with higher or lower Pol I traffic. The simplest interpretation of higher traffic areas is possible Pol I preferential pause sites.

In order to determine Pol I occupancy on the rDNA at a single-nucleotide resolution, we analyzed the 5′ end of the cDNA read which corresponds to the 3′ end of the nascent rRNA. This precise mapping of the polymerase provides insights into several properties about Pol I transcription regulation, such as potential pausing sites (Fig. 1). Additionally, NET-seq allows for investigation of these mechanisms *in vivo* and under different treatment conditions (7–12).

For this study, we obtained biological triplicates for each of the three log subphases. We prepared NET-seq libraries for those samples (described in Materials and Methods), generated reads, and mapped them to the *S. cerevisiae* genome. To analyze the resulting data, Pol I abundance on the rDNA was plotted for each subphase, and the Spearman correlation test was performed to test the similarity between triplicates (Fig. S1). As demonstrated by the histogram plots overlaying the biological replicates (Fig. S2) and the Spearman correlation factors of >0.88, the triplicates showed high similarity within each subphase.

**Fig 2:**
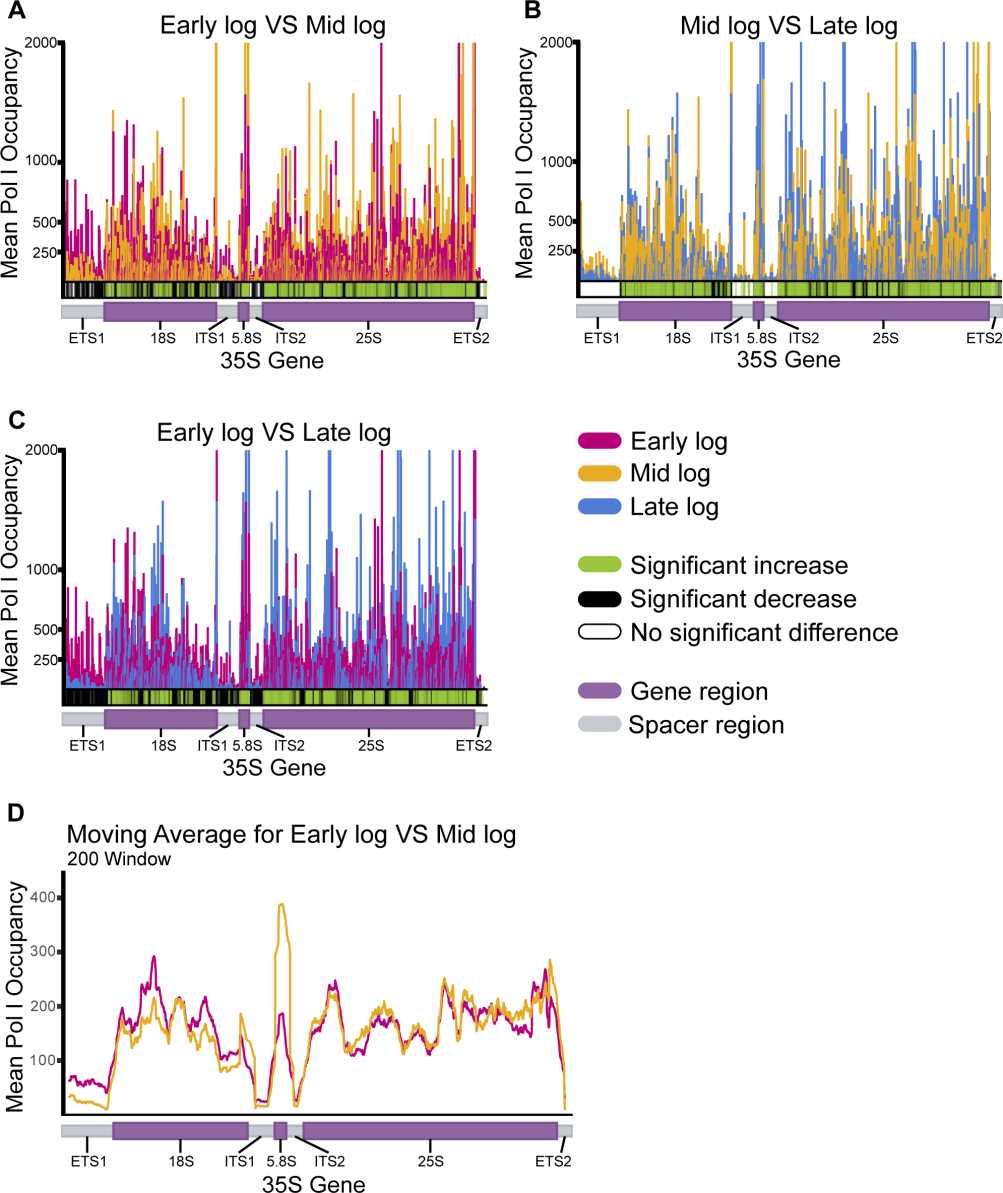
Pol I occupancy on the rDNA decreases with the progression of log phase and most notably between early log and mid log phase. (**A–C**) Mean Pol I occupancy on the rDNA gene obtained via NET-seq for early (pink), mid (orange), and late log (blue). Each plot is a two-way comparison between two of the studied yeast growth subphases as indicated in the title of each plot. Mean Pol I occupancy was calculated using normalized reads. Student’s *t*-test was performed between sample groupsto determine significant statistical differences. Indicated increased and decreased occupancy (green and black, respectively) represent a statistical significance of *P* < 0.05 between the latter subphases compared to the first subphase indicated in each plot title (i.e., early log vs mid log). (**D**) Moving average was calculated and plotted for every 200 positions. 35S gene spacer regions are indicated in gray, and gene regions are indicated in purple.

We then performed two-way comparisons of mean Pol I occupancy for each group combination (e.g., early vs late log phase) and conducted Student *t*-tests for each rDNA position to determine whether there was a significant difference between sample groups (Fig. 2A through C). We found that there were significant differences between subphases which, strikingly, were most prominent between early log and mid log phase as seen in both the occupancy plot and the rolling average plot (Fig. 2A and D). Due to a technological limitation of NET-seq wherein samples contain unavoidable mature rRNA contamination, changes in Pol I occupancy on the rDNA must be examined in the spacer regions(11). Overall, there was a significant decrease in Pol I occupancy on the spacer regions in later subphases when compared to early log phase.

This observed decrease in Pol I occupancy is evenly distributed over the rDNA spacer regions. This suggests that Pol I either prematurely disengages from the rDNA template or Pol I transcription initiation is repressed. This is different than Pol I pausing, which would be characterized by elevated Pol I occupancy at a specific region immediately followed by a region with lower Pol I occupancy. Overall, these results demonstrate Pol I occupancy (via regulation of transcription processivity and/or initiation) on the rDNA decreases as cells anticipate the imminent lack of nutrients.

### Pol I occupancy on the rDNA is decreased during the initial stages of the log phase

We observed a decrease in Pol I occupancy as cells progress through log phase with the sharpest decline between early and mid log phases. As shown in Fig. 2A through D, Pol I occupancy is significantly different between early log and mid log, and between early log and late log (Fig. 2, panels A and C). However, there is no significant difference in Pol I occupancy between mid and late log subphases (Fig. 2, panel B). These data indicate that the decrease in occupancy occurs before the diauxic shift whereupon yeast cells go from utilizing glucose as their main energy source to, instead, utilizing ethanol (13). This pre-diauxic shift decrease in Pol I occupancy on the rDNA coincides with the decrease in rRNA synthesis previously observed by our lab and others (6, 14).

Although there were significant differences in occupancy observed throughout the rDNA in all two-way comparisons, several reads mapping to the gene regions are from unavoidable mature rRNA contamination (11). In fact, it has previously been shown that a control yeast strain with an untagged Pol I resulted in high levels of NET-seq reads mapping to the gene regions revealing mature rRNA contamination. Because the objective of this method is to interrogate Pol I transcription, we cannot perform rRNA depletion. Additionally, we cannot distinguish between mature and nascent rRNA reads mapping to the gene regions as their sequences are identical. To circumvent this technological limitation, we evaluate the spacer regions of the rDNA. Processing of pre-rRNA occurs rapidly, co-transcriptionally, and post-transcriptionally, during which, the spacer regions are removed and degraded. Therefore, we routinely analyze Pol I occupancy on the spacer regions, which are indicative of only nascent rRNA.

**Fig 3:**
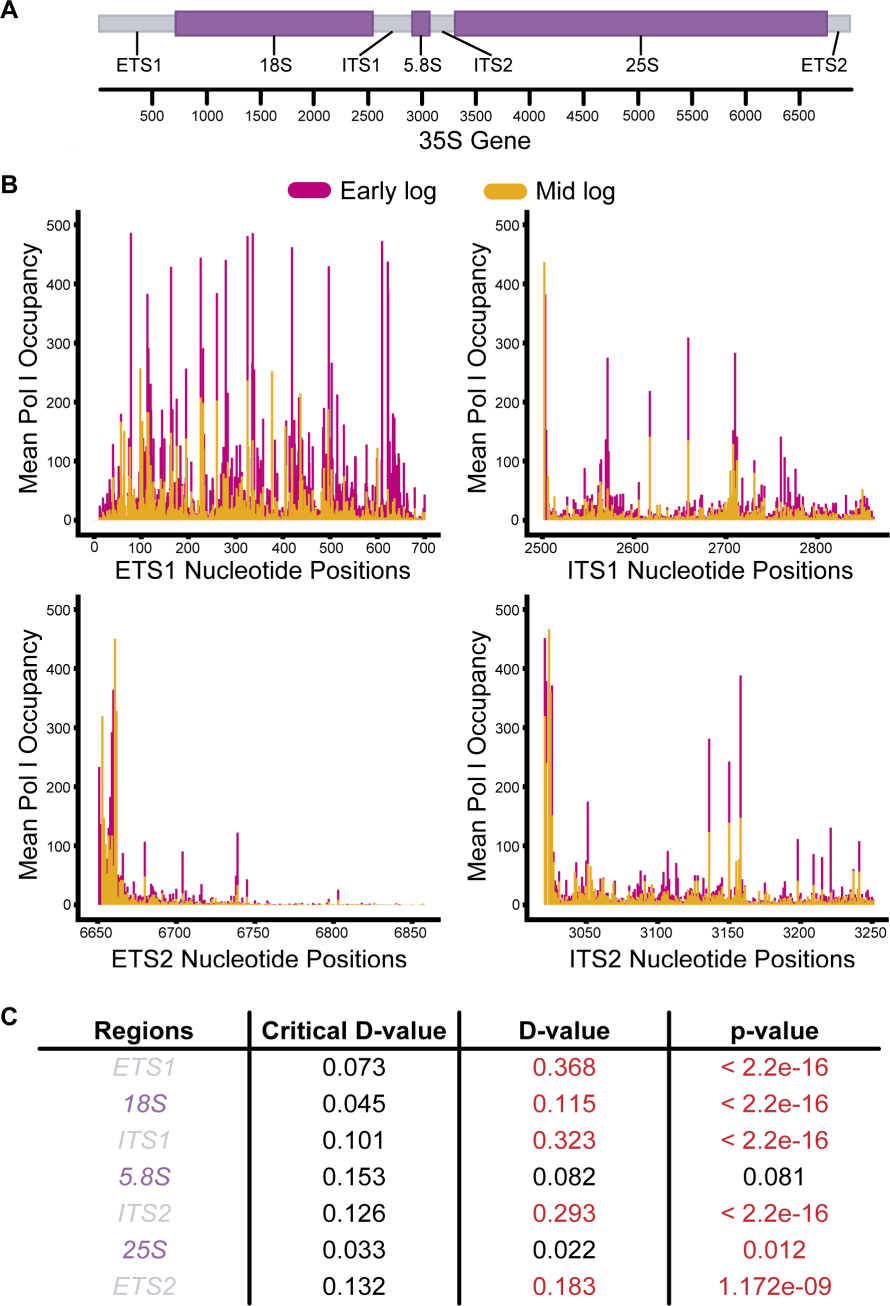
Pol I occupancy on the rDNA is decreased during the initial stages of the log phase. (**A**) Representative schematic of the rDNA gene in *S. cerevisiae*. Gray-colored regions represent the indicated spacers, and purple-colored regions represent the indicated gene-coding regions. Below the colored schematic are the corresponding nucleotide positions. (**B**) Mean Pol I occupancy on the spacer regions of early and mid log phase samples was plotted individually. (**C**) Two sample K-S test was performed for each rDNA spacer and gene region to determine whether the distribution of Pol I occupancy in those regions was significantly different between the two subphases. Critical *D*-values for each region were calculated at a 95% confidence interval (α=0.05) using the formula $D=1.63\surd \frac{n_{1}+n_{2}}{n_{1}n_{2}}$, where 1.63 is the coefficient at α=0.05, *n*1 is the size of sample 1, and *n*2 is the size of sample 2. *D*-value statistics < critical *D*-value and/or *P*-values <0.05 are denoted in red.

To take a closer look at the changes between early and mid log, we analyzed solely the spacer regions, which are illustrated in Fig. 3A as the gray boxes. We found that Pol I occupancy was uniformly decreased throughout all spacer regions in mid log phase samples when compared to early log phase samples (Fig. 3B). Additionally, when we performed a Kolmogorov-Smirnov test (15) to compare the sample distributions, we found that all the spacer regions differed significantly (Fig. 3C). These results suggest that there is a growth-phase-dependent control of Pol I initiation and elongation specifically between early and mid log phases prior to the diauxic shift. This was consistent with the previous findings of decreased rRNA synthesis during that timeline (6).

### Altered Pol I pause preference between logarithmic growth subphases indicates changes in transcription elongation efficiency

Changes in transcription elongation efficiency have been shown to result in altered Pol I pausing, represented by an accumulation of peaks in the NET-seq data. To test whether elongation efficiency might be affected during log phase progression, we looked at peak preference of each subphase. We did this by evaluating the top 2.5% occupied positions in each subphase’s spacer regions. The results from each subphase were then compared against the other two subphases in a pairwise manner. Jensen-Shannon (JS) divergence was calculated to determine the distance or differences between distributions, with JS distances ranging from 0 (distributions are identical) to 1 (distributions are dissimilar).

**Fig 4:**
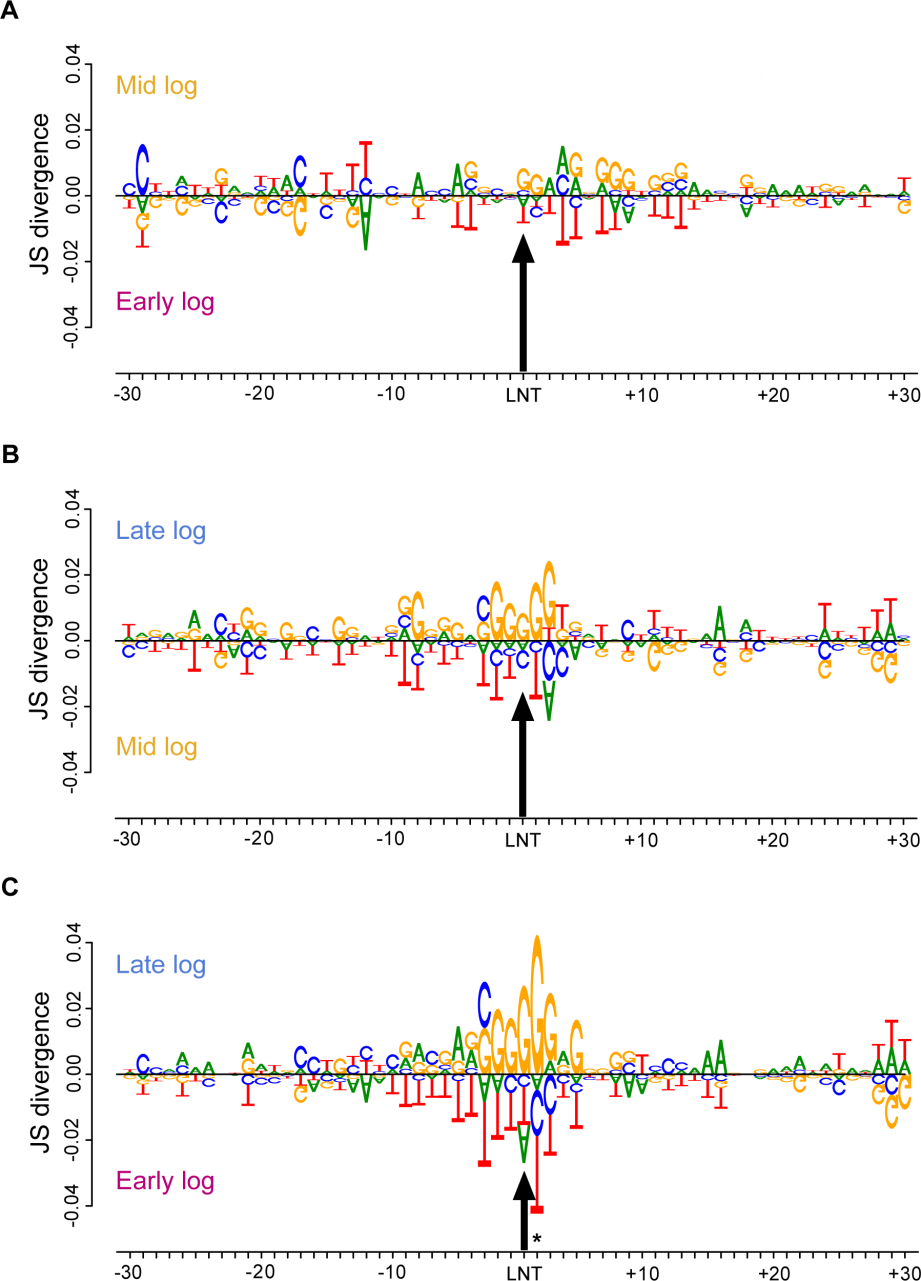
Pol I pausing preference sequence is altered with the progression of log phase. (A–C) The top 2.5% occupied nucleotides in the spacer regions of each sample were used to create these DiffLogos. Three two-way comparisons were produced, and the indicated sample was plotted either on top or on the bottom. The size of each letter correlates with its enrichment in the sample when compared to the indicated sample. We plotted 30 nucleotides down- and upstream of the last incorporated nucleotide (LNT; black arrow). Asterisks represent nucleotide position with statistically significant Jensen-Shannon divergence.

We found that in early log phase, Pol I shows an anti-preference for G as the last incorporated nucleotide which is consistent with previous NET-seq results in our lab (Fig. 4A and C) (7, 8, 11). It is important to note that our lab has historically used yeast cultures harvested at an OD_600_ equivalent to that of the early log cultures as defined in the present study. Interestingly, we observed enhanced preference for Pol I pausing at G nucleotides in late log (Fig. 4). Since samples for all three different subphases were processed in the exact same manner, we can infer that this change in pausing preference is not an artifact of library preparation.

We also observe changes in the downstream sequences that correlate with pausing. In both mid and late log, we observe an increase in the pausing directly upstream of G-rich regions. It has been shown that GC-rich transcription bubbles are correlated with increased Pol I occupancy, indicating a paused state or slower elongation (16). Interestingly, these studies were performed using yeast cell cultures harvested at an OD_600_ = 0.5, which corresponds to the mid log subphase in this project when we do see an increase in GC-rich transcription bubbles. This enrichment of G residues in the downstream DNA may reflect reduced efficiency of Pol I to unwind the DNA helix at the leading edge of the transcription elongation complex. How this change is related to DNA topology or *trans*-acting factors is not yet clear.

Additionally, further analysis of the sequence logos showed there was an increase in pausing directly downstream of T-rich regions in the earlier growth subphases. Our lab has previously shown that U-rich nascent RNA slows transcription elongation *in vitro* (8). This trend is recapitulated in early log NET-seq data (Fig. 4A and C). However, these pause site enhancements are lost in later subphases. It is plausible that if Pol I processivity is reduced in later subphases, then paused polymerases on U-rich RNA release from the template (consistent with mechanisms of Rho-independent termination of transcription in bacteria).

Lastly, the observed differences in pausing preference between subphases increase with the progression of log growth phase, once again, demonstrating that Pol I transcription elongation is continuously regulated as cell culture density increases. Since Pol I transcription elongation and pausing are sensitive to rDNA sequence (8), we hypothesize that growth-phase-dependent changes in Pol I pause profile reflect altered transcription elongation efficiency as discussed above. To achieve this change in elongation efficiency, it is plausible that different cohorts of transcription factors associate with the elongation complexes as cells divide.

### Transcriptomic landscape is significantly altered between the different log subphases

**Fig 5:**
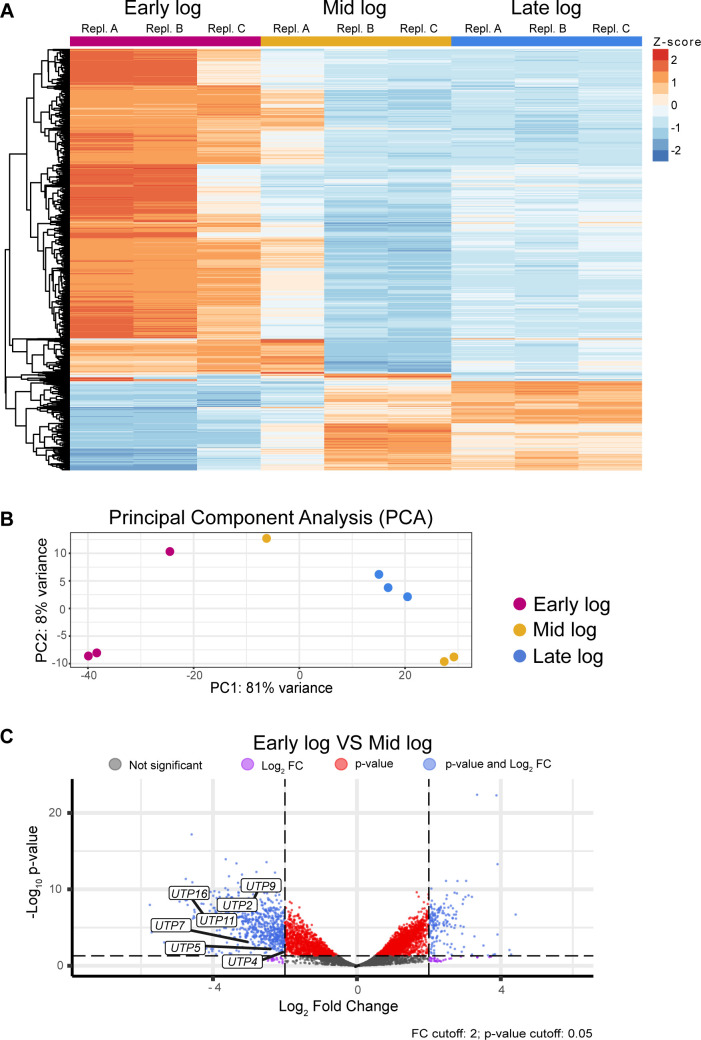
Several distinct genes involved rRNA synthesis are differentially regulated between growth phases. (**A**) Heatmap for normalized read counts of all three subphases. Colors indicate *Z* score of each replicate’s differentially expressed genes (DEGs) when compared to the mean for that specific DEG. Color indicates upregulation (orange) or downregulation of each gene (blue). (**B**) Principal component analysis of mRNA-seq data for all three subphases. (**C**) Volcano plot showing DEGs that are up- or downregulated in mid log phase when compared to early log phase. The fold change cutoff was set to 2, and the *P*-value cutoff was set to 0.05. Each gene is colored differently depending on whether they are below or above those thresholds (gray, non-significant; purple, log2 fold change > 2; red, *P*-value < 0.05; blue, log2 fold change > 2 and *P*-value < 0.05).

In order to further investigate transcriptomic differences between the three subphases, we performed mRNA-seq. This new ubiquitous technique allowed us to analyze which Pol II-derived transcripts are differentially expressed between subphases. Not surprisingly, there were hundreds of genes up- and downregulated between the sample groups (Fig. 5A).

To assess the variability among all subphases, our first step was to perform a principal component analysis. We found that 81% of the variance was explained by PC1. Interestingly, we saw that two replicates deviated from the other replicates in their sample group (early log C and mid log A) (Fig. 5B). This disparity was also noticeable in the heatmap of differentially expressed genes (DEGs; Fig. 5A). Even though each subphase was harvested in a smaller OD_600_ range when compared to a conventional log phase, there was still some variability in OD_600_ between each triplicate in the subphases. The two aforementioned samples were on the upper and lower limit of the OD_600_ range for early and mid log, respectively.

Interestingly, we observed that even small differences in harvest time influenced transcriptomic landscape between each individual sample. This was once again more pronounced in the earlier subphases. Even though the changes between individual samples are less pronounced than those between the subphases, they illustrate the complexity of the conventional yeast log phase.

In order to discern what pathways are significantly up- or downregulated between the subphases, we next performed gene set enrichment analysis (GSEA) on differentially expressed genes. Among the pathways of downregulated genes in mid log (when compared to early log) is rRNA processing (Fig. 5C). Namely, in the downregulated rRNA processing pathway, there were *UTP* genes which are essential for rRNA processing (17).

### rRNA processing decreases prior to exit from exponential growth phase

**Fig 6:**
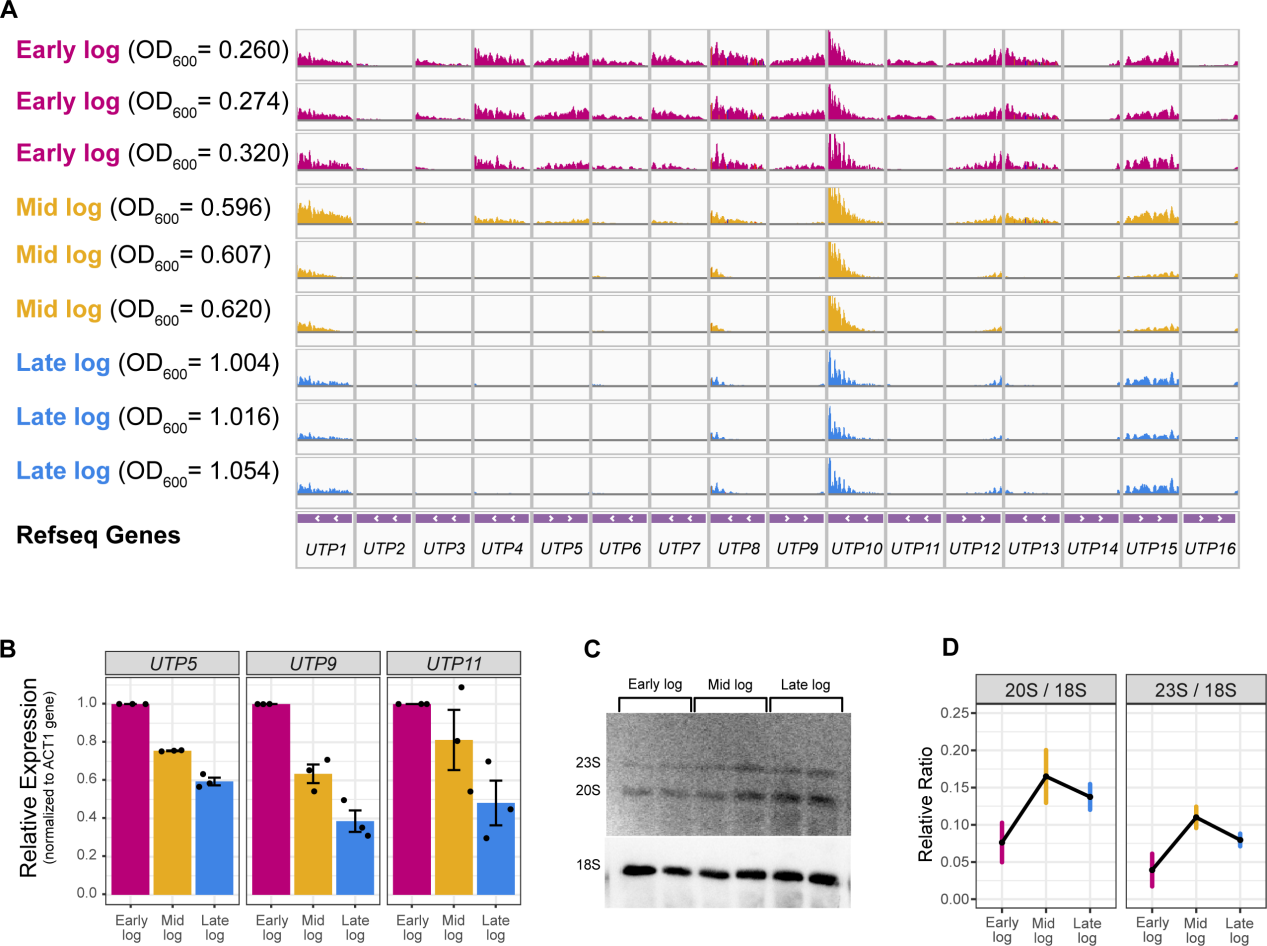
Several rRNA processing genes are downregulated prior to exit from exponential growth phase. (**A**) mRNA-seq normalized counts for *UTP1-16* were analyzed using Integrative Genomics Viewer with track range of 0–700 for all graphs. (**B**) qRT-PCR was used to validate the mRNA-seq findings between the three subphases for *UTP5*, *UTP9*, and *UTP11* genes. Delta Ct values were used to calculate relative expression (RE) where RE = 2^−ddCt^, and relative expression plotted was calculated for mid and late log with respect to early log. Error bars represent SEM for *n* = 3 biological replicates. Primers used are listed in Table 3. (**C**) Representative northern blot with all three subphases using probes for precursor rRNA (20S and 23S) and mature rRNA (18S). Biological duplicates were used for each subphase, and equal amounts of total RNA were loaded on each lane. (**D**) Relative ratio of precursor to mature rRNA. Quantification of the northern blot was performed using ImageQuant software, and precursor/mature rRNA ratios were calculated. Average ratios from two separate northern blots were plotted. Bars indicate average range of data for *n* = 2 from the two northern blots. Raw northern blots in Fig. S3. Probes used are listed in Table 4.

To confirm our mRNA-seq results, we performed qRT-PCR to detect the expression of UTP genes. We chose to target genes *UTP5*, *UTP9*, and *UTP11* because they are required for efficient rRNA processing (17). Additionally, *UTP5* and *UTP9* have been shown to also impact transcription efficiency and have therefore been dubbed t-*UTPs* (*UTP* required for transcription) (17). The mRNA-seq results show a continuous decrease in *UTP* gene expression as cells progress through log phase (Fig. 6A). Therefore, we examined *UTP5*, *UTP9*, and *UTP11* expression via qRT-PCR in all three subphases.

We saw a significant decrease in the expression of *UTP5* and *UTP9* mRNA in both mid and late log when compared to early log, which was consistent with our mRNA-seq findings (Fig. 6B). Despite the increased variability in *UTP11* gene expression, a clear downward trend was still observed (Fig. 6B). To determine whether the mRNA-seq (and qRT-PCR) results were consistent with decrease in rRNA processing, we performed northern blot analysis and probed for both precursor rRNA species (20S and 23S) and the mature 18S rRNA species.

We observed that as yeast cell cultures progress through log phase, the ratio of unprocessed pre-rRNA to mature rRNA increases significantly (Fig. 6C and D). This observation supports the predictions from mRNA-seq and shows that both Pol I transcription and pre-rRNA processing are repressed as cells progress through log phase. Interestingly and consistent with our other results in this study, the ratio of unprocessed to mature rRNA is the highest during mid log subphase.

## DISCUSSION

The observation that rRNA synthesis is decreased while cells are rapidly growing (log phase) contradicts the cellular need for ribosome biogenesis during proliferation (4, 14). This has led to investigative studies on the mechanisms that regulate said rRNA decrease which have shown that yeast cells display a quorum-sensing-like mechanism to regulate rRNA synthesis as they anticipate nutrient depletion (6). This cell density-dependent regulatory mechanism begs the question of whether the conventional yeast log phase has more complexity that has yet to be investigated. In this study, we sought to elucidate the dynamic relationship between rRNA synthesis and different subphases during log growth.

### Pol I initiation and elongation are altered in a growth-phase-dependent manner

The results obtained from our NET-seq experiments demonstrate a growth-phase-dependent control of Pol I transcription. It has previously been established that yeast metabolic and transcriptional landscape differ after the diauxic shift (18). However, within log phase, these changes remained largely unexplored. We showed that as *S. cerevisiae* cell cultures progress through log phase, Pol I occupancy on the rDNA is reduced. Further investigation of those data showed that the decrease in Pol I did not occur at any one specific locus of the 35S gene. Rather, the decrease in the polymerase’s occupancy can be observed at similar levels throughout the spacer regions (Fig. 2 and 3). The observed overall reduction in Pol I occupancy can be influenced by various factors, including the activity or abundance of key transcription factors, changes in chromatin modifications, or altered expression of the polymerase itself. It is unlikely that chromatin accessibility is a driver of the observed regulation since previous studies have shown that rDNA transcription can be regulated in a chromatin-independent manner and that the rate of rRNA synthesis remains stable even in strains with mutations in chromatin modifiers (19, 20). Additionally, our mRNA-seq data (Tables S1 to S3) indicate no significant changes in the expression of Pol I subunit genes across different subphases. Therefore, we propose that the simplest explanation for the observed Pol I occupancy on rDNA is that (i) Pol I transcription initiation is decreased leading to an overall decreased number of polymerases transcribing the template, and/or (ii) Pol I transcription elongation is affected by premature disengagement of the polymerase leading to an overall decrease in processivity.

Additionally, the analysis of the sequence preference of Pol I occupancy revealed that this too was altered between the subphases. More specifically, we see that Pol I pausing preference at G residues increases in the later subphases (Fig. 4A through C). Notably, this G preference at high-occupancy positions has not been observed in previous studies (8). Published WT Pol I NET-seq results were performed on *S. cerevisiae* samples harvested at an OD_600_ equivalent to what is described in this study as early log phase. Concurrently, Pol I *spt4*∆ NET-seq data, in which Pol I processivity is compromised, showed an increase in G residue preference pausing by the polymerase (7). These observations substantiate our findings that, in the later subphases, there is an increase in Pol I preferential pausing at G residues as well as a putative decrease in transcription processivity. The regulatory mechanism by which yeast cells regulate transcription elongation in anticipation of nutrient deprivation is yet to be elucidated.

### *Saccharomyces cerevisiae* cultures anticipate nutrient depletion

Ribosome biogenesis is an energetically demanding cellular process that is regulated on multiple levels (14). It is not surprising that *S. cerevisiae* decreases the synthesis of rRNA in culture conditions with low nutrient availability (stationary phase). In fact, rRNA synthesis decrease has been observed even prior to entry to stationary phase (4, 6). However, the mechanism for controlling the reduction in rRNA production is not fully understood. In this study, we compared three log subphases and found that most of the changes in Pol I transcription properties occurred between early and mid log phase (Fig. 2A through C). These results are consistent with the observation that rRNA synthesis decreases before the diauxic shift, at which point glucose is depleted and cells switch to ethanol as their carbon source (4, 6). In fact, the data presented here suggest that the repression of Pol I transcription occurs much earlier than the switch to a post-diauxic state since all subphases in this study are in a pre-diauxic state. These findings suggest that even when yeast cells are rapidly dividing, ribosome biogenesis is tightly controlled to be able to adapt to pending nutrient limitation.

### Pre-diauxic shift decrease in rRNA synthesis is associated with both altered Pol I transcription and altered rRNA processing

Investigation of the transcriptomic landscape between all the subphases revealed an array of differentially expressed genes between each sample (Fig. 5A). GSEA showed that rRNA processing is among the downregulated pathways in the latter subphases (Fig. 5C). Among the downregulated genes involved in rRNA processing are *UTP* genes, which are part of the ribosomal small subunit processome. Previous literature suggests that several of the *UTP* genes are essential for rRNA processing (17). Additionally, some Utps are also required for efficient Pol I transcription (named t-UTPs). Consistent with these findings, we found that a decrease in *UTP5*, *UTP9*, and *UTP11* mRNA in mid and late log is consistent with a decrease in rRNA processing (Fig. 6A through D). Moreover, the decrease in rRNA processing regulation occurs in the initial stages of log phase as observed by our results comparing early and mid log (Fig. 6B through D).

Previous literature suggests that yeast rRNA processing is altered prior to the transition from exponential to stationary growth phase (i.e., pre-diauxic shift) (21, 22). More specifically, rRNA processing pathway is rapidly altered from A2 to A3 cleavage prior to entry into stationary phase. Consequently, there is an observable accumulation of 23S pre-rRNA with the switch to A3 cleavage pathway. Interestingly, we see an increase in both 20S and 23S precursor rRNA in the later subphases. This increase is more prominent in mid log and abated in late log subphase. The increase of both 20S and 23S suggests another putative regulation of rRNA processing other than just the shift from A2 to A3 cleavage; perhaps an overall decrease in rRNA processing efficiency early in log phase. However, it is important to note that the change in 20S signal was more variable than that of 23S signal. Alongside altered Pol I transcription, these findings can offer a better insight into how rRNA synthesis decreases in rapidly growing yeast cells.

### Small variance in OD_600_ within log phase influences transcriptomic landscape

Finally, in this study, we observed a very high sensitivity to harvest time on the transcriptomic landscape of *S. cerevisiae*. As is to be expected of any RNA-seq experiment, changes in sample conditions result in many DEGs. Interestingly, these DEGs were observed not just between subphases but also within each subphase. In particular, it was notable that the transcriptomic landscape of the early log sample with the highest OD_600_ was more similar to the mid log sample with the lowest OD_600_. This is indicative of highly dynamic gene expression in concordance with log phase progression. Notably, the aforementioned *UTP* genes’ expression showed a decreasing pattern that is sensitive to small changes in harvest time (Fig. 6A).

One consideration when examining cell population gene expression is cell cycle. It has been well characterized that cell cycle state highly affects gene expression and vice versa (23). In this study, the yeast cell populations were not synchronized, and therefore, the cell populations within each subphase sample were heterogenous with respect to cell cycle stages. Consequently, we are most likely underestimating the gene expression changes between each individual sample because we analyzed an average with respect to gene expression that is affected by cell cycle. It is plausible that synchronized yeast cell populations would show an even higher degree of sensitivity to the time of harvest with respect to gene expression profiles. Nonetheless, these results show that small cell density changes within log growth phase of yeast result in a significantly altered transcriptomic landscape, including expression of genes involved in rRNA synthesis control as previously discussed here.

In conclusion, this study demonstrates growth-phase-dependent control of rRNA synthesis that is associated with the regulation of both Pol I transcription initiation and elongation, as well as pre-rRNA processing. The regulatory mechanisms explored here support the hypothesis that these cells anticipate nutrients deprivation and regulate ribosome synthesis accordingly. Additionally, transcriptomic landscape, including that of genes that impact rRNA synthesis, is highly sensitive to small changes in yeast cell population. Together, these data demonstrate that ribosome synthesis is highly dynamic and responsive to even small changes in cell growth and metabolic state. It will be interesting to evaluate changes in rRNA synthesis in the presence of other stimuli that perturb or promote cell growth.

## MATERIALS AND METHODS

### Strains and media

We used a wild-type *Saccharomyces cerevisiae* strain with HA tag on Rpa135: *MATa ade2-1 ura3-1 trp1-1 leu2-3,112 his3-11 can1-100 RPA135-(HA)3-(His)7::TRP1.*

All experiments were performed in YPD media containing 10% yeast extract, 20% peptone, and 2% glucose. Cells were grown at 30°C with nutation until the desired OD_600_ measure using GENESYS 30 Visible Spectrophotometer.

### NET-seq protocol

Wild-type yeast was grown in YPD until the desired OD_600_ of 0.25–0.4, 0.5–0.7, and 0.8–1.0 for early log, mid log, and late log growth phase, respectively. NET-seq protocol was performed in its entirety as described previously (7, 11), except that the detergent concentration was optimized in the lysis buffer (20 mM Tris Cl [pH 7.9], 1.6% Triton X-100, 0.35% NP-40, 100 mM NH_4_Cl, 5 mM EDTA Na [pH 8.5], 1× HALT Protease Inhibitor, 25 U/mL RiboLock RNase Inhibitor) and wash buffer (20 mM Tris Cl [pH 7.9], 1.6% Triton X-100, 0.35% NP-40, 300 mM KCl, 50 mM EDTA Na [pH 8.5], 25 U/mL RiboLock RNase Inhibitor).

In brief, cells were cryogenically harvested and lysed. Pol I was immunoprecipitated with HA-beads, and RNA was extracted. UMI linker was ligated on the RNA and then fragmented. RNA was reverse transcribed, and gel size selection was used to remove unused linker and primer. cDNA was circularized and then amplified using primers specific to each sample (listed on Table 1).

**TABLE 1:** Library amplification primers for NET-seq^*a*^

Sample	Forward primer	Reverse primer	Run #
Early log replicate 1	CAAGCAGAAGACGGCATACGAGATtcgccttaTCCGACGATCATTGATGGTGCC	AATGATACGGCGACCACCGAGATCTACACtagatcgcCGTCTCTTCTGCGGATGACTCG	3
Early log replicate 2	CAAGCAGAAGACGGCATACGAGATtcgccttaTCCGACGATCATTGATGGTGCC	AATGATACGGCGACCACCGAGATCTACACtagatcgcCGTCTCTTCTGCGGATGACTCG	1
Early log replicate 3	CAAGCAGAAGACGGCATACGAGATctagtacgTCCGACGATCATTGATGGTGCC	AATGATACGGCGACCACCGAGATCTACACtagatcgcCGTCTCTTCTGCGGATGACTCG	1
Mid log replicate 1	CAAGCAGAAGACGGCATACGAGATtcgccttaTCCGACGATCATTGATGGTGCC	AATGATACGGCGACCACCGAGATCTACACtagatcgcCGTCTCTTCTGCGGATGACTCG	2
Mid log replicate 2	CAAGCAGAAGACGGCATACGAGATctagtacgTCCGACGATCATTGATGGTGCC	AATGATACGGCGACCACCGAGATCTACACtagatcgcCGTCTCTTCTGCGGATGACTCG	2
Mid log replicate 3	CAAGCAGAAGACGGCATACGAGATttctgcctTCCGACGATCATTGATGGTGCC	AATGATACGGCGACCACCGAGATCTACACtagatcgcCGTCTCTTCTGCGGATGACTCG	2
Late log replicate 1	CAAGCAGAAGACGGCATACGAGATcagcctcgTCCGACGATCATTGATGGTGCC	AATGATACGGCGACCACCGAGATCTACACtagatcgcCGTCTCTTCTGCGGATGACTCG	3
Late log replicate 2	CAAGCAGAAGACGGCATACGAGATtgcctcttTCCGACGATCATTGATGGTGCC	AATGATACGGCGACCACCGAGATCTACACtagatcgcCGTCTCTTCTGCGGATGACTCG	3
Late log replicate 3	CAAGCAGAAGACGGCATACGAGATtcctctacTCCGACGATCATTGATGGTGCC	AATGATACGGCGACCACCGAGATCTACACtagatcgcCGTCTCTTCTGCGGATGACTCG	3

^*a*^ All samples were amplified using the same reverse primer and different primers when sequenced on the same run. All primers are listed in the 5′ to 3′ direction.

### Sequencing and data analysis

Libraries were sequenced utilizing the Illumina NovaSeq6000 according to manufacturer’s instructions. Data analysis was performed as described previously (7, 11).

In brief, reads were deduplicated using Fqtrim (version 0.9.7) with parameter “-C,” and adapters were trimmed using Cutadapt (version 3.4-GCCcore-10.3.0). More specifically, Cutadapt “-g -no-indels” option was used to trim 5*'* sequence (5′-AGNNNNNNNNTG-3′), and “-a -no-indels” option was used to trim 3*'* sequence (5′-CTGTAGGCACCAT-3′). Reads were then aligned to the *S. cerevisiae* R64-1-1 genome assembly (GenBank assembly: GCA_000146045.2) using STAR (version 2.7.3a-GCC-6.4.0–2.28) with “-alignIntronMax” option set to 1. Aligned BAM files were sorted and indexed using SAMtools (version 1.12-GCC-10.2.0) and converted to BED files using BEDTools (version 2.28.0-foss-2018b). BEDTools was also used to generate coverage files using the “genomecov” function and parameters “-d −5 -stand +” and “-d −5 -stand -” for plus and minus strands, respectively. The resulting count tiles were further processed for statistical analysis and visualization using R (version 4.4.0) and Rstudio (version 2024–04-24) with both base R base functions, ggplot2 (version 3.5.1) and/or DiffLogo (version 2.28.0).

The raw sequence data have been added to NCBI’s Gene Expression Omnibus (24) and can be found under the GEO series accession number GSE268167.

### mRNA-seq

#### Cell harvest and RNA extraction

Wild-type yeast was grown in YPD until the desired OD_600_ of 0.2–0.3, 0.5–0.7, and 0.8–1.0 for early log, mid log, and late log growth phase, respectively. Cells were harvested at the indicated ODs by pelleting. Pellets were washed with DI H_2_O once and resuspended in TES (10 mM Tris Cl [pH 7.5], 1% SDS, 10 mM EDTA [pH 8.5]). Total RNA was extracted via hot acidic phenol (pH 4.3) extraction at 65°C, followed by 5 minutes on ice and removal of the aqueous layer. Two extra acidic phenol extraction and two chloroform extractions were performed at room temperature. The final aqueous layer containing the RNA was combined with 1 mL of salty ethanol (1 M ammonium acetate, 95% ethanol) and precipitated at −80°C for at least 8 hours. Samples were then centrifuged at 16,000 × *g* for 1 hour at 4°C. RNA pellets were washed twice with 750 µL 75% ethanol, air dried, and then resuspended in 100 µL of DI H_2_O (depending on the sample, this volume had to be adjusted since some samples contained a large amount of RNA). RNA purity and concentration were determined via spectrophotometry (NanoDrop ND-1000 Spectrophotometer).

#### Library prep

Library prep was performed using NEBNext Poly(A) mRNA Magnetic Isolation Module (cat. E7490S), NEBNext Ultra II Directional RNA Library Prep Kit for Illumina (cat. E7760S), and NEBNext Multiplex Oligos for Illumina (Dual Index Primers Set 1) (cat. E7600S). The index primers used for each specific sample are listed in Table 2. mRNA-seq protocol was performed according to manufacturer’s instructions.

**TABLE 2:** Library amplification primer sequences for mRNA-seq^*a*^

Sample	Forward primer	Reverse primer	Run #
Early log replicate 1	CAAGCAGAAGACGGCATACGAGATacgaattcGTGACTGGAGTTCAGACGTGTGCTCTTCCGATC*T	AATGATACGGCGACCACCGAGATCTACACatagaggcACACTCTTTCCCTACACGACGCTCTTCCGATC*T	1
Early log replicate 2	CAAGCAGAAGACGGCATACGAGATttctgaatGTGACTGGAGTTCAGACGTGTGCTCTTCCGATC*T	AATGATACGGCGACCACCGAGATCTACACatagaggcACACTCTTTCCCTACACGACGCTCTTCCGATC*T	1
Early log replicate 3	CAAGCAGAAGACGGCATACGAGATcatagccgGTGACTGGAGTTCAGACGTGTGCTCTTCCGATC*T	AATGATACGGCGACCACCGAGATCTACACggctctgaACACTCTTTCCCTACACGACGCTCTTCCGATC*T	3
Mid log replicate 1	CAAGCAGAAGACGGCATACGAGATttctgaatGTGACTGGAGTTCAGACGTGTGCTCTTCCGATC*T	AATGATACGGCGACCACCGAGATCTACACcctatcctACACTCTTTCCCTACACGACGCTCTTCCGATC*T	2
Mid log replicate 2	CAAGCAGAAGACGGCATACGAGATacgaattcGTGACTGGAGTTCAGACGTGTGCTCTTCCGATC*T	AATGATACGGCGACCACCGAGATCTACACcctatcctACACTCTTTCCCTACACGACGCTCTTCCGATC*T	2
Mid log replicate 3	CAAGCAGAAGACGGCATACGAGATagcttcagGTGACTGGAGTTCAGACGTGTGCTCTTCCGATC*T	AATGATACGGCGACCACCGAGATCTACACcctatcctACACTCTTTCCCTACACGACGCTCTTCCGATC*T	2
Late log replicate 1	CAAGCAGAAGACGGCATACGAGATctatcgctGTGACTGGAGTTCAGACGTGTGCTCTTCCGATC*T	AATGATACGGCGACCACCGAGATCTACACggctctgaACACTCTTTCCCTACACGACGCTCTTCCGATC*T	3
Late log replicate 2	CAAGCAGAAGACGGCATACGAGATttcgcggaGTGACTGGAGTTCAGACGTGTGCTCTTCCGATC*T	AATGATACGGCGACCACCGAGATCTACACggctctgaACACTCTTTCCCTACACGACGCTCTTCCGATC*T	3
Late log replicate 3	CAAGCAGAAGACGGCATACGAGATgcgcgagaGTGACTGGAGTTCAGACGTGTGCTCTTCCGATC*T	AATGATACGGCGACCACCGAGATCTACACggctctgaACACTCTTTCCCTACACGACGCTCTTCCGATC*T	3

^*a*^ All samples were amplified using different primer combinations when sequenced on the same run. All primers are listed in the 5′ to 3′ direction.

#### Data analysis

Libraries were single-end sequenced using the Illumina NextSeq500 according to manufacturer’s instructions. Reads were first aligned to the *Saccharomyces cerevisiae* genome assembly R64-1-1 (GenBank assembly: GCA_000146045.2 [25]) using STAR (version 2.7.3a-GCC-6.4.0-2.28). Feature counts were determined using HTseq (version 2.0.3). Differential expression was calculated using DESeq2 (version 1.44.0). Gene set enrichment analysis was performed using WebgestaltR (version 0.4.6). All graphs were created using RStudio (version 2024-04-24, R version 4.4.0) with R base functions, DESeq2, enhancedvolcano (version 1.22.0), and/or pheatmap (version 1.0.12).

The raw sequence data have been added to NCBI’s Gene Expression Omnibus (24) and can be found under the GEO series accession number GSE268168. In addition, fold change and normalized counts are provided for each two-way comparison in Tables S1 to S3.

### qRT-PCR

#### Cell harvest and RNA extraction

Yeast cells were harvested, and total RNA was extracted as described above in “mRNA-seq.”

#### cDNA synthesis

cDNA synthesis was performed on 100 ng of RNA sample using the SuperScript First-Strand Synthesis system (Invitrogen cat # 18080–044). cDNA was precipitated overnight in 1 mL salty ethanol (1 M NH_4_OAc in 95% EtOH) at −80°C. Samples were centrifuged at max speed for 10 minutes at 4°C. cDNA pelleted were washed twice with 750 µL 75% ethanol, air dried, and then resuspended in 100 µL DI H_2_O.

#### qRT-PCR

qRT-PCR was prepared using PowerUp SYBR Green Master Mix (cat # A25742) and following manufacturer’s instructions. For each subphase, three biological replicates and three dilutions were made. A 96-well plate was then sealed and centrifuged at 1,000 RPM for 1 minute. Target genes were amplified using Bio-Rad CFX Opus 96 Real-Time PCR System with the following PCR conditions: 95°C 3 minutes, 95°C 10 s, 60°C 30 s, plate read, go to step 2 × 39. qRT-PCR primers are listed in Table 3.

**TABLE 3:** qRT-PCR primer sequences used^*a*^

Target gene	Forward primer	Reverse primer
*ACT1*	CGCTGGTTTCTCTCTACCTCACG	GCAGCGGTTTGCATTTCTTGTTCG
*UTP5*	CTTGCCTCAGCCACCAACTACG	CCTTGCGACGGTGCTTGATTC
*UTP9*	GCTGATCCAATCATGGCCTGTC	CTGTTGCTTGCTGTTTATTGGCGTG
*UTP11*	TTATGTGAGGACGCTGAGGC	CCCTCTGTAAGTGCTGCTTGA

^*a*^ *UTP5*, *UTP9*, and *UTP11* were the three genes of interest. *ACT1* was used as a normalization control. All sequences are listed 5′ to 3′.

### Northern blot

#### Cell harvest and RNA extraction

Yeast cells were harvested, and total RNA was extracted as described above in “mRNA-seq.”

#### Formaldehyde gel

Five micrograms of RNA sample was combined with RNA loading dye (48% formamide, 1× MOPS, 6.4% formaldehyde, 0.5 mg/mL bromophenol blue, 5% glycerol) and heated for 5 minutes at 65°C. RNA samples were run on a 0.8% agarose formaldehyde gel (5% formaldehyde, 1× MOPS) at 80 V for 2 hours 10 minutes.

#### Northern blot transfer

RNA was transferred from the agarose formaldehyde gel to a Genescreen Plus nylon membrane (cat # NEF1017001PK) using the Fisher Scientific Semidry Electroblotter (model FB-SDB-2020) for 4 hours 30 minutes at 250 mA. “Sandwich” assembly and transfer buffer selection were performed according to the manufacturer’s instructions.

#### Northern blot hybridization

Following transfer, nylon membrane was cross-linked using the UV Stratalinker 1800 at 1,200 × 100 µJ/cm^2^. The membrane was pre-hybridized in 25 mL of hybridization buffer (0.5M sodium phosphate pH 7.2, 7% SDS) for 4 hours at 65°C with rotation. The DNA probes used are listed in Table 4. Probe was boiled for 5 minutes at 95°C and then radiolabeled with γ-^32^P ATP using T4 polynucleotide kinase (NEB cat # M0201S). Labeling reaction was carried out for 2 hours at 37°C. Labeled probe was boiled for 5 minutes at 95°C and then immediately added to the pre-hybridized membrane, taking care to add it to the buffer and not directly the membrane. Hybridization was performed overnight at 65°C with rotation. Following overnight hybridization, the membrane was cooled down to 25°C with rotation over the span of 2 hours. Membrane was then washed three times using the wash buffer (40 mM sodium phosphate [pH 7.2], 1% SDS). The first wash was performed for 15 minutes at 25°C, the second wash for 10 minutes at 42°C, and the final wash for 15 minutes at 25°C. All membrane incubations were performed using the UVP HB-500 Minidizer Hybridization oven. Blot was exposed to phosphor screen overnight and then imaged using GE Typhoon Imaging systems. Quantification of band intensity was determined using ImageQuant software.

**TABLE 4:** Northern probe sequences^*a*^

Probe	Sequence
20S and 23S	GCACAGAAATCTCTCACCGT
18S	AGCCATTCGCAGTTTCACTG

^*a*^ All sequences are listed 5′ to 3′.

### Statistical statement

All statistical software used for analysis of NET-seq and mRNA-seq data are listed above, and the statistical tests performed are indicated in each figure legend. Statistical analysis of qRT-PCR data used average Ct values from biological triplicates to calculate relative expression (RE) where RE = 2^−ddCt^. In brief, the delta Ct (dCt) values were determined by normalizing the Ct values of the target genes to the Ct value of the ACT1 gene. Subsequently, these dCt values were used to calculate the ddCt for the mid and late log phases relative to the early log phase. Finally, the RE was calculated. Analysis of northern blot data was performed using the band intensity calculated using ImageQuant software. The background was subtracted using rolling ball method, and the resulting values were used to calculate precursor/product ratios. Both qRT-PCR and northern blot statistical and graphical analysis were performed using R (version 4.4.0) and R studio (version 2024-04-24).

## Data Availability

All raw blots and images are provided in supplemental materials. Sequence files have been deposited into GEO and are available under the following ascension numbers: GSE268167 for the NET-seq data set and GSE268168 for the mRNA-seq data set.

## SUPPLEMENTAL MATERIAL

Supplemental figures msphere.00493-24-s0001.docx Figures S1 to S3.

Table S1 msphere.00493-24-s0002.docx Differential gene expression data set.

Table S2 msphere.00493-24-s0003.docx Differential gene expression data set.

Table S3 msphere.00493-24-s0004.docx Differential gene expression data set.

ASM does not own the copyrights to Supplemental Material that may be linked to, or accessed through, an article. The authors have granted ASM a non-exclusive, world-wide license to publish the Supplemental Material files. Please contact the corresponding author directly for reuse.
