# Supplementary material for: Growth-phase-dependent control of rRNA synthesis in Saccharomyces cerevisiae
Source: mSphere. 2024 Oct 3;9(10):e00493-24. doi: 10.1128/msphere.00493-24 (PMC11520348; doi:10.1128/msphere.00493-24)
Supplement: Supplemental figures — Figures S1 to S3. [file msphere.00493-24-s0001.docx]

**Growth-phase dependent control of rRNA synthesis in *Saccharomyces cerevisiae***

**SUPPLEMENTAL FIGURES**

| **Early log** | **1** | **2** | **3** |
| --- | --- | --- | --- |
| **1** | 1.000 | 0.923 | 0.887 |
| **2** | 0.923 | 1.000 | 0.944 |
| **3** | 0.887 | 0.944 | 1.000 |

| **Mid**  **log** | **1** | **2** | **3** |
| --- | --- | --- | --- |
| **1** | 1.000 | 0.962 | 0.953 |
| **2** | 0.962 | 1.000 | 0.992 |
| **3** | 0.953 | 0.992 | 1.000 |

| **Late**  **log** | **1** | **2** | **3** |
| --- | --- | --- | --- |
| **1** | 1.000 | 0.989 | 0.988 |
| **2** | 0.989 | 1.000 | 0.987 |
| **3** | 0.988 | 0.987 | 1.000 |

0 1

**Supplementary figure 1. Spearman correlation shows similarity between biological triplicates in each subphase.** Total normalized counts for each triplicate were used to perform a Spearman correlation test. This test ranks each position from highest to lowest count and then compares said ranking between each replicate. A spearman correlation of 1 or 0 indicates 100% similarity or dissimilarity, respectively.


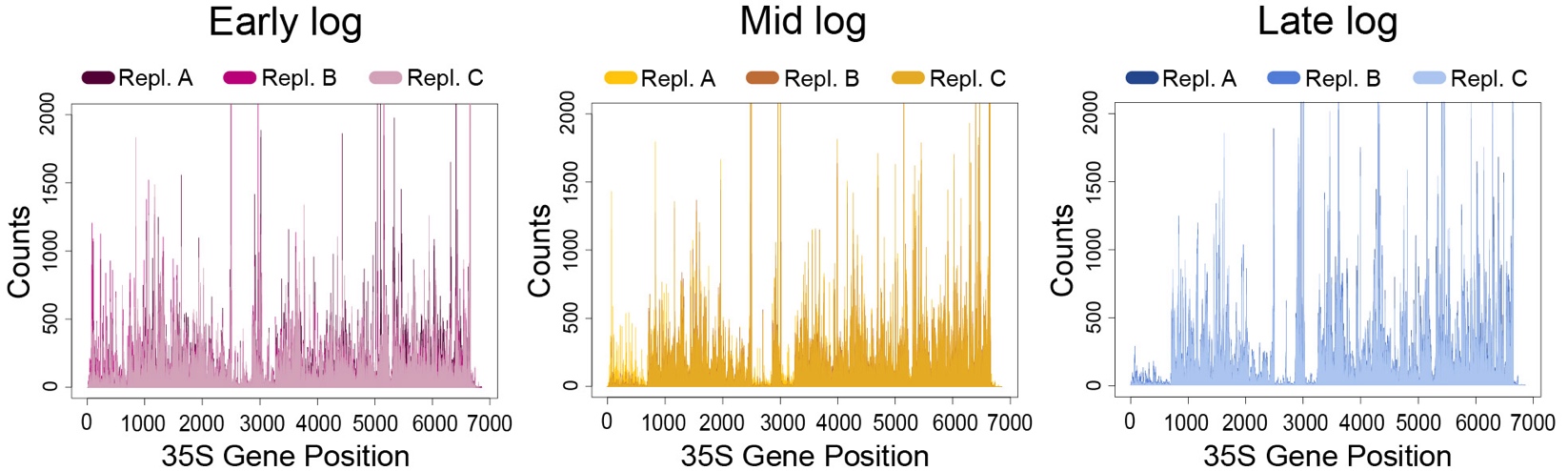


**Supplementary figure 2. Pol I occupancy on the rDNA decreases with the progression of Log phase.** Total normalized counts for Pol I occupancy were plotted for Early (pink), Mid (yellow), and Late log (blue). Each replicate was plotted in a different shade of the corresponding subphase.


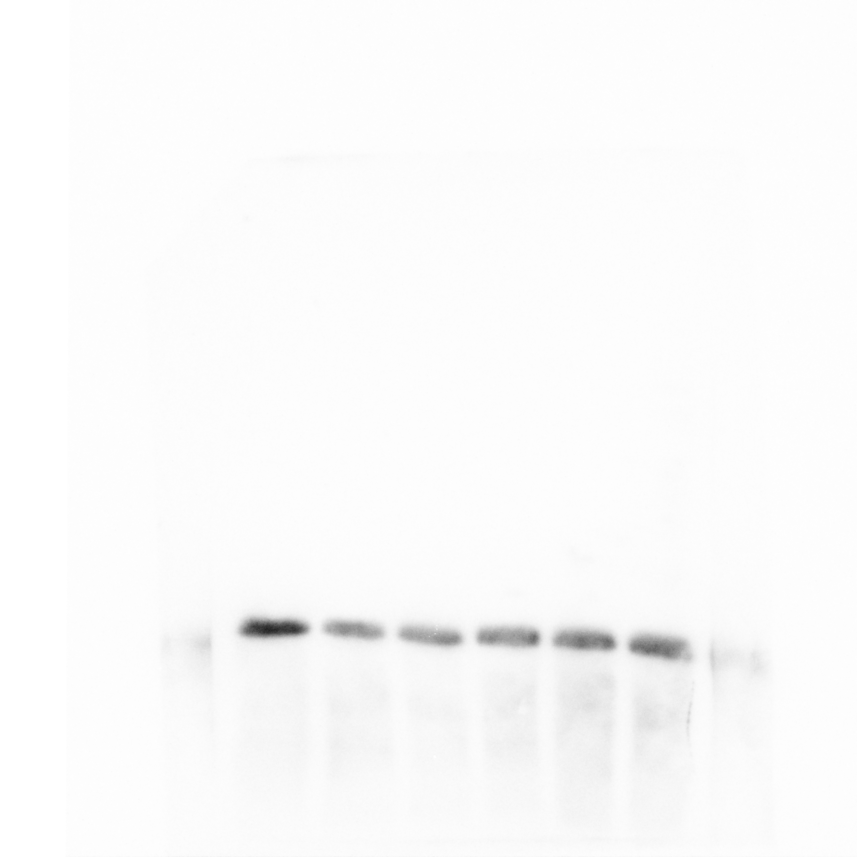

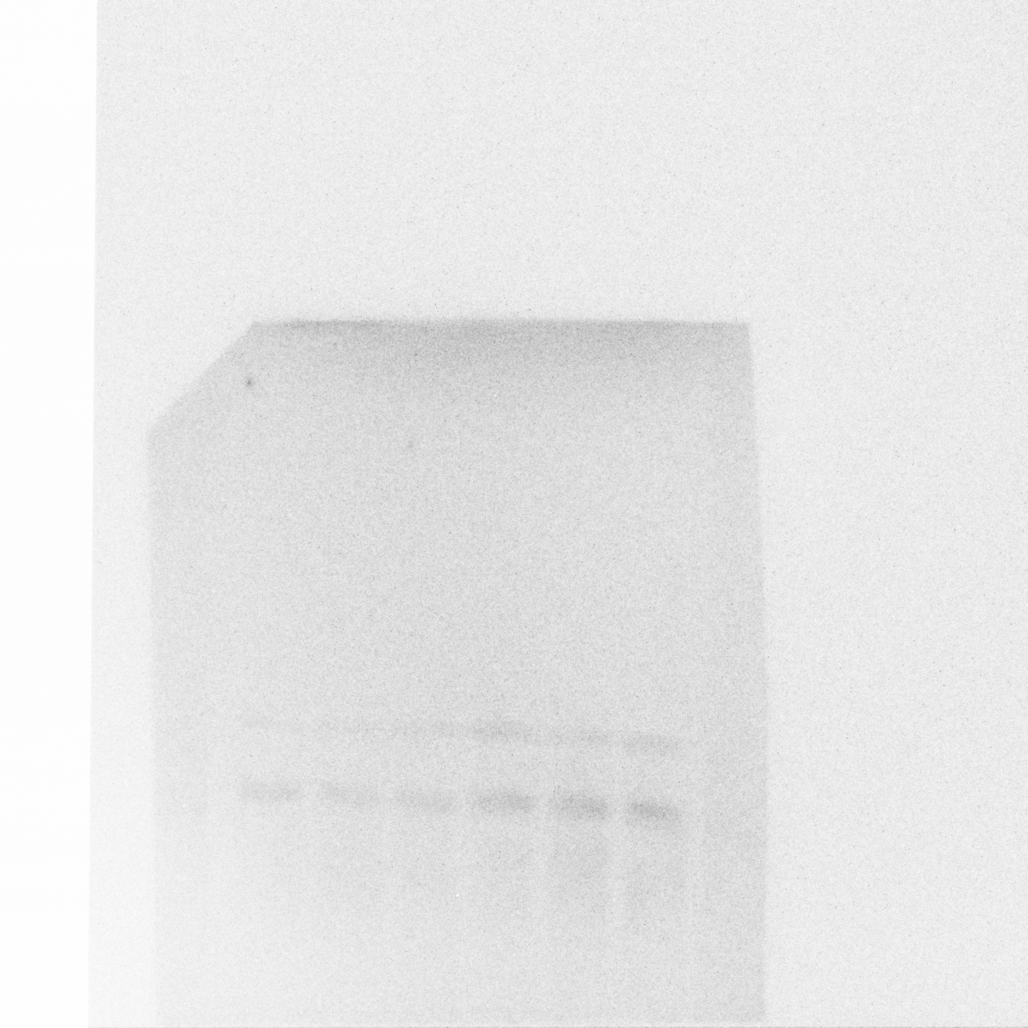


**B**

**A**

**D**

**C**


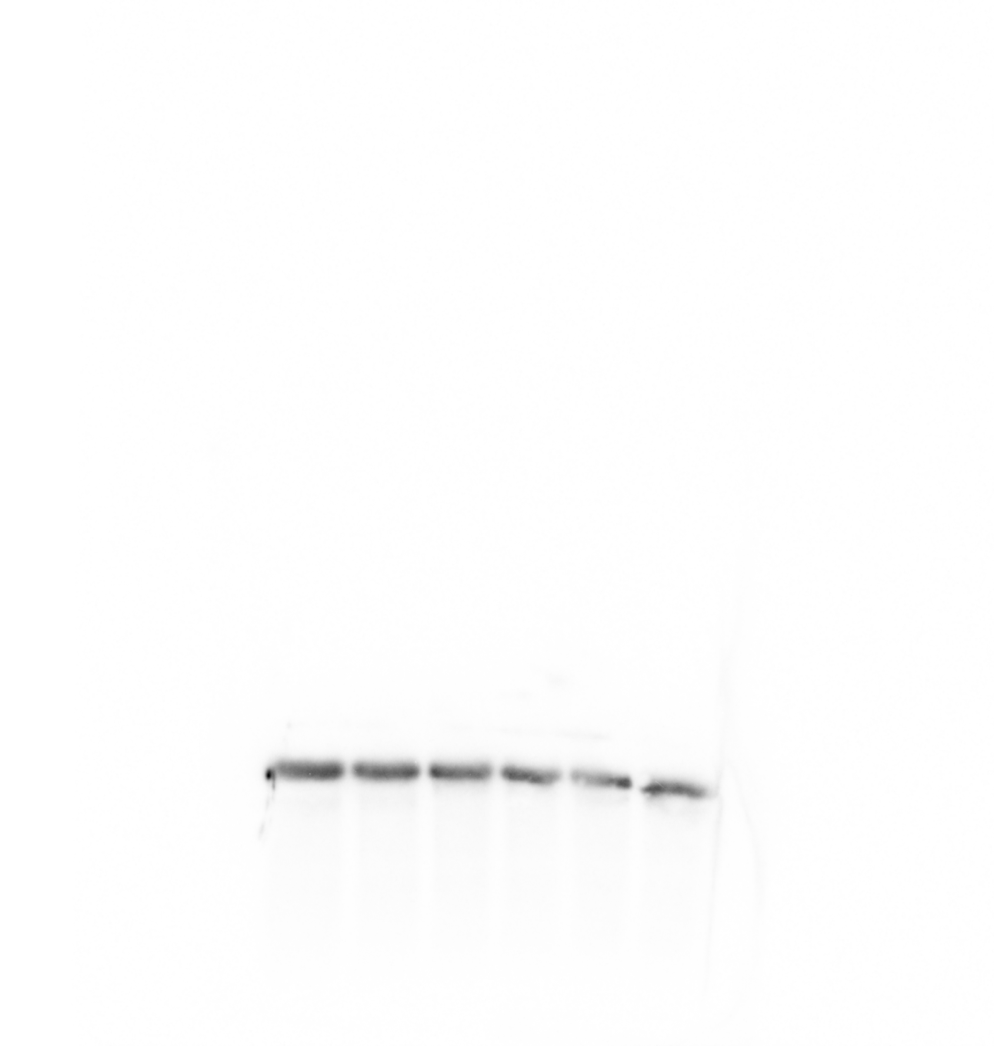

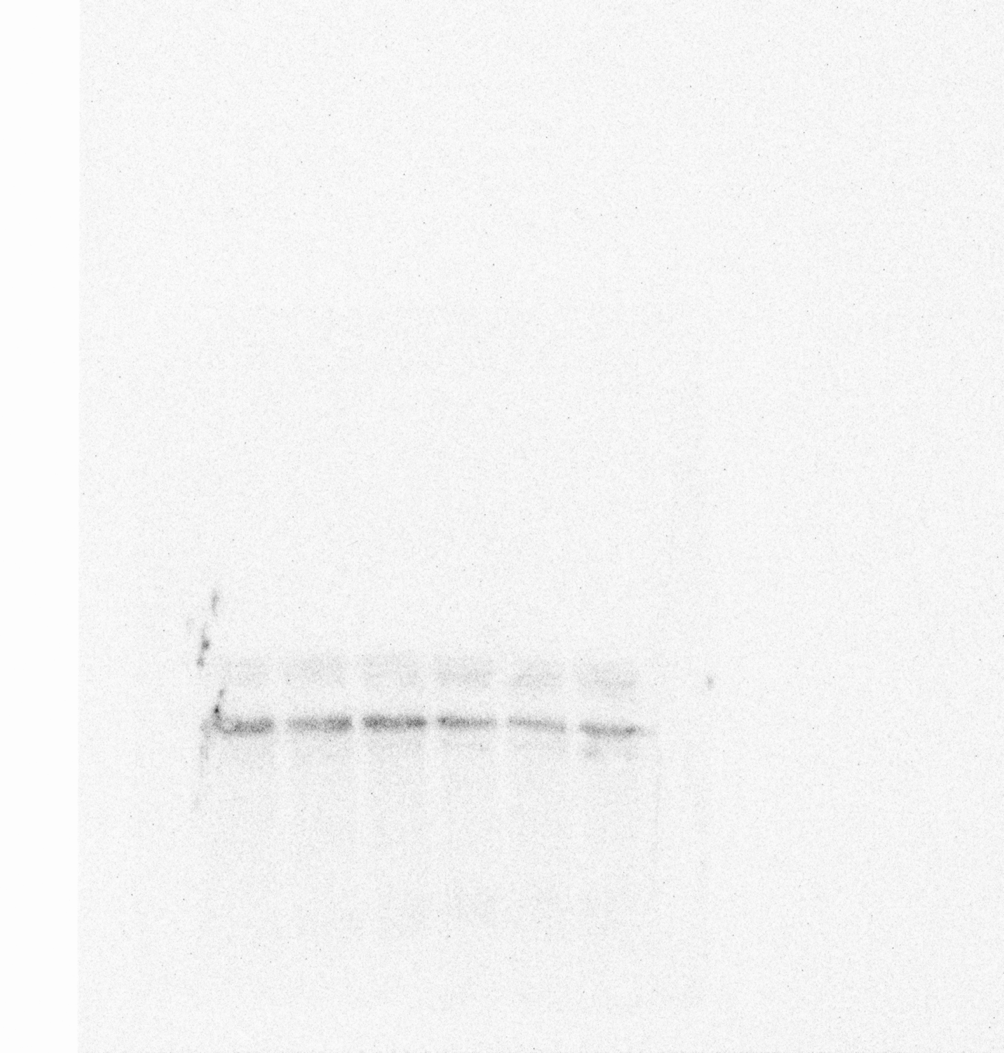


**Supplementary figure 3. rRNA processing decreases with the progression of exponential growth phase.** Raw images of Northern blots obtained as described in the “Methods and Materials” section for both 18S (A and C) and 20S and 23S (B and D). Images were obtained from GE Typhoon Imaging systems.
