# Supplementary material for: Growth-phase-dependent control of rRNA synthesis in Saccharomyces cerevisiae
Source: mSphere. 2024 Oct 3;9(10):e00493-24. doi: 10.1128/msphere.00493-24 (PMC11520348; doi:10.1128/msphere.00493-24)
Supplement: Table S1 — Differential gene expression data set. [file msphere.00493-24-s0002.docx]

**Supplemental Table 1. mRNA-seq statistics for two-way comparison between Early and Mid log subphases.** Table includes statistics output from DESeq as described in column headers. Statistics represent gene expression changes in Mid log when compared to Early log. Additionally, the individual sample’s normalized counts were appended on the table and column headers describe which individual sample the respective column values refer to.

|  | baseMean | log2FoldChange | lfcSE | stat | pvalue | padj | Early_RNAseq_A_i502_i706_counts.txt | Early_RNAseq_B_i502_i705_counts.txt | Early_RNAseq_C_i504_i709_counts.txt | Mid_RNAseq_A_i503_i705_counts.txt | Mid_RNAseq_B_i503_i706_counts.txt | Mid_RNAseq_C_i503_i707_counts.txt |
| --- | --- | --- | --- | --- | --- | --- | --- | --- | --- | --- | --- | --- |
| HRA1 | 7.29473751707722 | 0.665975258614589 | 0.817485763869723 | 0.814662821113935 | 0.41526534037684 | 0.512614297877487 | 7.5592583541153 | 2.47318449231126 | 6.58375719308209 | 5.22748998934596 | 10.9989753403642 | 10.9257597332445 |
| ICR1 | 415.11019714077 | 0.876277341049183 | 0.383427721913597 | 2.28537815856373 | 0.0222906635751571 | 0.0465020646012348 | 263.818116558624 | 291.011375261958 | 322.604102461022 | 331.945614323468 | 680.365188911101 | 600.916785328448 |
| IRT1 | 1070.10154178529 | 0.271778707409385 | 0.340510205705125 | 0.798151429401618 | 0.424782619470986 | 0.522061081945257 | 960.025810972642 | 911.780682832084 | 1035.84446504492 | 823.329673321988 | 1456.57859150252 | 1233.05002703759 |
| LSR1 | 229.246614308157 | 0.609656081461769 | 0.608879920568032 | 1.00127473557186 | 0.31669400367354 | 0.41235531412406 | 297.078853316731 | 178.893678277181 | 68.0321576618483 | 121.974766418072 | 367.680032806461 | 341.82019736865 |
| NME1 | 134.175260381077 | 0.567996413500233 | 0.539354028082955 | 1.05310498100678 | 0.292292880237852 | 0.386126389483439 | 133.798872867841 | 140.971516061742 | 49.0124146596111 | 87.9960814873236 | 226.264635573207 | 167.008041636738 |
| PWR1 | 3.10428590915199 | 0.0273691667608694 | 1.18121223986147 | 0.0231704056538385 | 0.981514345134557 | 0.986723756270145 | 2.26777750623459 | 1.64878966154084 | 5.12070003906385 | 1.74249666311532 | 6.28512876592241 | 1.56082281903493 |
| Q0020 | 39.9022057734116 | 0.288571760926987 | 0.900540187538806 | 0.320442957371685 | 0.748632565630324 | 0.808111586097769 | 64.25369600998 | 23.0830552615717 | 21.2143287332645 | 108.906041444707 | 15.712821914806 | 6.24329127613972 |
| Q0045 | 254.915233827703 | 1.31802427175104 | 0.54151713403963 | 2.43394749473353 | 0.0149351613611131 | 0.0333388004040098 | 206.367753067348 | 147.566674707905 | 83.3942577790398 | 152.46845802259 | 480.812350593065 | 458.881908796269 |
| Q0050 | 7.12853681389092 | -0.0758809237326461 | 0.804391030296623 | -0.0943333787606565 | 0.924844335711701 | 0.947050011445914 | 11.3388875311729 | 5.77076381539294 | 5.12070003906385 | 9.58373164713426 | 4.71384657444181 | 6.24329127613972 |
| Q0055 | 35.1989229919986 | 0.60456014729122 | 0.61421727823692 | 0.98427733753531 | 0.324979169121565 | 0.420592011483697 | 38.552217605988 | 34.6245828923576 | 10.2414000781277 | 24.3949532836145 | 54.9948767018211 | 48.3855073900828 |
| Q0060 | 5.35507897322487 | 0.782229589302496 | 1.0656217915787 | 0.734059302732201 | 0.462912595771282 | 0.559065371091492 | 6.80333251870377 | 3.29757932308168 | 1.46305715401824 | 1.74249666311532 | 14.1415397233254 | 4.68246845710479 |
| Q0065 | 12.7520921264001 | 0.0697841624861416 | 0.753704560554385 | 0.092588218432448 | 0.926230703868914 | 0.947872917715848 | 18.8981458852882 | 12.3659224615563 | 5.85222861607297 | 9.58373164713426 | 23.5692328722091 | 6.24329127613972 |
| Q0070 | 9.22757851292964 | 0.0592968321120605 | 0.908781449251569 | 0.0652487263696841 | 0.94797596572243 | 0.962472839232611 | 10.5829616957614 | 14.0147121230971 | 2.19458573102736 | 3.48499332623064 | 17.2841041062866 | 7.80411409517465 |
| Q0075 | 156.952561480127 | 1.46739904144411 | 0.665815619669704 | 2.20391201121424 | 0.0275305326750817 | 0.0551982037445598 | 122.459985336668 | 104.698143507843 | 22.6773858872828 | 90.6098264819966 | 287.54464104095 | 313.725386626021 |
| Q0085 | 26.3748937407885 | 2.4242850704069 | 0.754274095329198 | 3.21406380706849 | 0.00130870587724834 | 0.00441722528798706 | 12.0948133665845 | 7.41955347693378 | 5.12070003906385 | 11.3262283102496 | 81.7066739569914 | 40.5813932949082 |
| Q0105 | 2.3917409318765 | 0.694724468673056 | 1.24039273034766 | 0.560084279499396 | 0.575421952718004 | 0.66320927521403 | 2.26777750623459 | 2.47318449231126 | 0.731528577009121 | 2.61374499467298 | 3.14256438296121 | 3.12164563806986 |
| Q0120 | 1.18038075713363 | -0.448754234600489 | 2.00485466411155 | -0.223833798346352 | 0.822886629847088 | 0.867046776213228 | 0.75592583541153 | 2.47318449231126 | 0.731528577009121 | 0 | 0 | 3.12164563806986 |
| Q0130 | 188.548629911744 | 1.23327631562843 | 0.602453845340558 | 2.04708846190744 | 0.0406493996448639 | 0.0766565545279424 | 163.27998044889 | 135.200752246349 | 38.7710145814834 | 123.717263081188 | 344.110799934252 | 326.2119691783 |
| Q0140 | 5.28671857709755 | 0.413879955626349 | 1.17540930742698 | 0.352115601783305 | 0.72475156821465 | 0.788709386448777 | 6.04740668329224 | 7.41955347693378 | 0 | 2.61374499467298 | 4.71384657444181 | 10.9257597332445 |
| Q0158 | 137.964838512316 | -0.34484878849336 | 0.629734796562686 | -0.547609549886184 | 0.58396003065783 | 0.67047648376474 | 279.692559102266 | 128.605593600185 | 55.5961718526932 | 189.93213627957 | 106.847189020681 | 67.115381218502 |
| Q0160 | 232.89915921095 | 1.8404323853175 | 0.464984066638379 | 3.95805473211798 | 7.55626453521091e-05 | 0.000453516715813218 | 123.215911172079 | 96.4541952001391 | 84.857314933058 | 179.477156300878 | 515.380558805638 | 398.009818853907 |
| Q0250 | 1.69388345961347 | -0.671083080723399 | 1.52158137082613 | -0.441043176257502 | 0.659181739804694 | 0.736745991728953 | 3.77962917705765 | 1.64878966154084 | 0.731528577009121 | 0.87124833155766 | 1.5712821914806 | 1.56082281903493 |
| Q0275 | 2.10271510886079 | 0.0477761929067744 | 1.46862100612175 | 0.0325313288504154 | 0.974048332435512 | 0.981288075527846 | 3.77962917705765 | 1.64878966154084 | 0.731528577009121 | 1.74249666311532 | 4.71384657444181 | 0 |
| RDN5-1 | 172.415403308205 | 3.90833745160159 | 0.51908704865664 | 7.52925248610245 | 5.10316212908111e-14 | 4.69490915875462e-11 | 22.6777750623459 | 12.3659224615563 | 29.2611430803648 | 162.052189669725 | 413.247216359399 | 394.888173215837 |
| RME2 | 2034.96206053354 | 0.964609192066355 | 0.360222006806485 | 2.67781860585922 | 0.00741033380101752 | 0.0185546460647562 | 1409.8016830425 | 1458.35445563287 | 1267.73902395681 | 1728.5566898104 | 3134.7079720038 | 3210.61253875485 |
| RME3 | 231.338382649412 | 0.775781138055976 | 0.420661811442843 | 1.84419197786245 | 0.0651551670690048 | 0.112523270561649 | 162.524054613479 | 169.825335138706 | 178.492972790226 | 156.824699680379 | 358.252339657578 | 362.110894016104 |
| RNA170 | 21.1866975351731 | 0.637635140149092 | 0.516726357689413 | 1.23398996521163 | 0.217206643529226 | 0.303759127975725 | 14.3625908728191 | 18.1366862769492 | 16.8251572712098 | 18.2962149627109 | 28.2830794466509 | 31.2164563806986 |
| RPR1 | 471.586437982918 | 1.99936972052106 | 0.632636826273948 | 3.16037517495904 | 0.00157566103836809 | 0.0051300591946868 | 264.574042394035 | 246.494054400355 | 54.8646432756841 | 327.58937266568 | 1024.47598884535 | 911.520526316399 |
| RUF20 | 60.7770438462347 | -0.249159728080698 | 0.440652595369622 | -0.565433474575819 | 0.571778961335853 | 0.66002088385067 | 77.104435211976 | 72.5467451077969 | 47.5493575055929 | 37.4636782569794 | 67.565134233666 | 62.4329127613972 |
| RUF21 | 2.14222875903431 | 2.23814105867759 | 1.49116355934609 | 1.500935993674 | 0.133372117395495 | 0.203824498345274 | 1.51185167082306 | 0 | 0.731528577009121 | 4.3562416577883 | 1.5712821914806 | 4.68246845710479 |
| RUF23 | 17.1007705667293 | 0.723451416702297 | 0.645284509514046 | 1.12113557048986 | 0.262230159716029 | 0.352928365427634 | 12.0948133665845 | 5.77076381539294 | 20.4828001562554 | 15.6824699680379 | 28.2830794466509 | 20.2906966474541 |
| SCR1 | 1315.75531959903 | 1.80240268243797 | 0.61753980780294 | 2.91868258477214 | 0.00351513951546227 | 0.00993281982532723 | 807.328792219514 | 737.833373539525 | 213.606344486663 | 761.471041781395 | 2870.73256383506 | 2503.55980173203 |
| snR10 | 68.9574666050152 | 1.75447839827374 | 0.520706944173234 | 3.36941617143105 | 0.000753276001494377 | 0.00283193079370916 | 23.4337008977574 | 44.5173208616027 | 26.3350287723284 | 53.1461482250172 | 146.129243807696 | 120.18335706569 |
| snR11 | 309.284080289934 | 1.36329498885775 | 0.475702208030907 | 2.86585802176722 | 0.00415880717134732 | 0.0114066091071025 | 184.445903840413 | 201.976733538753 | 132.406672438651 | 203.000861252935 | 576.660564273382 | 557.21374639547 |
| snR128 | 93.6550262365079 | 1.83256761425968 | 0.53384112534065 | 3.43279587740697 | 0.000597391667525652 | 0.00234871937659658 | 51.402956807984 | 50.2880846769956 | 21.2143287332645 | 74.0561081824011 | 194.838991743595 | 170.129687274807 |
| snR13 | 82.5843215287847 | 1.86438234031321 | 0.48816295299195 | 3.81918031445528 | 0.000133895863263388 | 0.000703909681156095 | 39.3081434413995 | 36.2733725538985 | 30.7242002343831 | 61.8586315405938 | 169.698476679905 | 157.643104722528 |
| snR14 | 1.5745512659633 | 1.56300661297996 | 1.56739630151642 | 0.997199375466044 | 0.318667744052447 | 0.414171598728105 | 0.75592583541153 | 0.82439483077042 | 0.731528577009121 | 0.87124833155766 | 3.14256438296121 | 3.12164563806986 |
| snR161 | 14.5339719071685 | 1.0864006313988 | 0.683202396317119 | 1.59015928113714 | 0.111798906276722 | 0.176250907324869 | 5.29148084788071 | 9.06834313847462 | 13.1675143861642 | 9.58373164713426 | 21.9979506807285 | 28.0948107426287 |
| snR17a | 204.768156152714 | 1.27234141218411 | 0.543948256021419 | 2.33908537824967 | 0.0193310152586975 | 0.0412360842219318 | 154.208870423952 | 131.078778092497 | 73.8843862779212 | 111.51978643938 | 372.393879380903 | 385.523236301628 |
| snR17b | 171.450678457624 | 0.756709570170316 | 0.536415856120965 | 1.41067711093102 | 0.158339843043555 | 0.23463152075483 | 164.035906284302 | 147.566674707905 | 70.2267433928756 | 94.0948198082272 | 271.831819126144 | 280.948107426287 |
| snR18 | 43.7963628957905 | 1.01050666775814 | 0.464327003154195 | 2.17628236327786 | 0.0295341534915047 | 0.0584331638971705 | 24.1896267331689 | 38.7465570462097 | 24.140443041301 | 50.5324032303443 | 45.5671835529375 | 79.6019637707814 |
| snR189 | 46.5804679650828 | 1.25287173948859 | 0.487522240523784 | 2.56987606994612 | 0.010173490229817 | 0.0241582880088576 | 28.7251817456381 | 32.9757932308168 | 20.4828001562554 | 39.2061749200947 | 67.565134233666 | 90.5277235040259 |
| snR19 | 173.642411145209 | 1.39158819029888 | 0.528019851208943 | 2.63548460746831 | 0.00840172363117362 | 0.0206122286418126 | 115.656652817964 | 112.117696984777 | 59.2538147377388 | 106.292296450034 | 353.538493083136 | 294.995512797602 |
| snR190 | 39.0112143097715 | 0.760263086553451 | 0.567412096509275 | 1.33987817889431 | 0.180284953434696 | 0.2606724517556 | 36.2844400997534 | 36.2733725538985 | 13.8990429631733 | 27.0086982782874 | 62.8512876592241 | 57.7504443042924 |
| snR191 | 72.2499421733642 | 2.04361908236859 | 0.539587688807608 | 3.78737158900831 | 0.000152249323806505 | 0.00077786741886463 | 41.5759209476341 | 21.4342656000309 | 21.2143287332645 | 54.8886448881326 | 142.986679424735 | 151.399813446388 |
| snR24 | 64.4765566709837 | 0.638938669896717 | 0.495895773737934 | 1.28845354958475 | 0.197588128346448 | 0.281457099436214 | 58.2062893266878 | 61.005217477011 | 31.4557288113922 | 43.562416577883 | 97.4194958717974 | 95.2101919611307 |
| snR3 | 11.622215568184 | 1.77663334062903 | 0.74602693534101 | 2.38146004717232 | 0.0172441612262728 | 0.0374797159288548 | 6.04740668329224 | 6.59515864616336 | 2.92611430803648 | 8.7124833155766 | 28.2830794466509 | 17.1690510093842 |
| snR30 | 1249.36999036687 | 2.15444991102237 | 0.47194128226022 | 4.56508042844713 | 4.99302875137558e-06 | 5.38516921705934e-05 | 500.422903042432 | 525.963902031528 | 348.207602656342 | 920.038238124888 | 2718.31819126144 | 2483.26910508457 |
| snR31 | 703.833742011402 | 1.35237395582743 | 0.468059109167141 | 2.88932301356943 | 0.00386072268688271 | 0.0107446214794834 | 436.169207032453 | 431.9828913237 | 319.677988152986 | 453.920380741541 | 1354.44524905628 | 1226.80673576146 |
| snR32 | 75.657516289675 | 2.01708608529472 | 0.511128614002871 | 3.94633763408008 | 7.93556912646947e-05 | 0.000468424062094073 | 33.2607367581073 | 32.1513984000464 | 24.140443041301 | 55.7598932196902 | 172.841041062866 | 135.791585256039 |
| snR33 | 436.947363115585 | 1.55070889249522 | 0.499147178479831 | 3.10671673476741 | 0.001891775763506 | 0.00594584476182461 | 262.306264887801 | 278.645452800402 | 125.822915245569 | 321.490634344776 | 823.351868335836 | 810.067043079129 |
| snR34 | 70.8923605345983 | 1.66958570560078 | 0.502981728514512 | 3.31937645236473 | 0.000902187164897332 | 0.00327144444928988 | 32.5048109226958 | 46.9905053539139 | 21.9458573102736 | 59.2448865459209 | 133.558986275851 | 131.109116798934 |
| snR35 | 146.31279200331 | 1.64073730980509 | 0.498250374526668 | 3.2929976447359 | 0.000991253049777353 | 0.00354451395922607 | 76.3485093765645 | 87.3858520616645 | 49.0124146596111 | 102.807303123804 | 298.543616381315 | 263.779056416903 |
| snR36 | 54.328472983125 | 1.39880264004104 | 0.538964883241216 | 2.59535024179859 | 0.00944945725426875 | 0.0227069047453324 | 32.5048109226958 | 35.448977723128 | 21.2143287332645 | 34.8499332623064 | 91.134367105875 | 110.81842015148 |
| snR37 | 354.834763156107 | 1.74503176822275 | 0.461647860743332 | 3.78000618352903 | 0.000156824463970836 | 0.000796187366276909 | 154.208870423952 | 163.230176492543 | 171.177687020134 | 246.563277830818 | 705.505703974791 | 688.322863194404 |
| snR38 | 41.1251694474011 | 0.736435763322981 | 0.52911696829899 | 1.39182034870377 | 0.163976806401798 | 0.241373859023447 | 33.2607367581073 | 42.8685312000618 | 16.0936286942007 | 33.9786849307487 | 54.9948767018211 | 65.554558399467 |
| snR39 | 71.1363741847732 | 0.139018982616554 | 0.37678046092004 | 0.36896547734214 | 0.712153457159791 | 0.778673982522065 | 57.4503634912762 | 84.0882727385828 | 61.4484004687662 | 68.8286181930551 | 72.2789808081078 | 82.7236094088513 |
| snR39B | 39.5569831166509 | 0.882828827369119 | 0.514754194377401 | 1.71504931288012 | 0.0863361862057546 | 0.141946652837646 | 32.5048109226958 | 32.9757932308168 | 17.5566858482189 | 32.2361882676334 | 48.7097479358987 | 73.3586724946417 |
| snR4 | 499.738683250338 | 1.07940755906187 | 0.44032968642175 | 2.45136222323201 | 0.0142316657644507 | 0.0320237342847878 | 333.363293416485 | 396.533913600572 | 232.6260874889 | 369.409292580448 | 868.919051888774 | 797.580460526849 |
| snR40 | 21.3732372303137 | -0.192987863490648 | 0.542020566216372 | -0.356052658366488 | 0.721801130075973 | 0.786617856920084 | 24.9455525685805 | 24.7318449231126 | 18.288214425228 | 14.8112216364802 | 29.8543616381315 | 15.6082281903493 |
| snR41 | 215.238664453615 | 1.1015274471567 | 0.431979730600005 | 2.54995169710097 | 0.0107737844965632 | 0.0253500811683839 | 145.137760399014 | 134.376357415578 | 130.212086707624 | 153.339706354148 | 388.106701295709 | 340.259374549615 |
| snR42 | 151.543091396496 | 1.30729202867236 | 0.550964910816617 | 2.37273191632974 | 0.0176570743738146 | 0.0382095292229052 | 113.388875311729 | 104.698143507843 | 43.1601860435381 | 96.7085648029002 | 284.402076657989 | 266.900702054973 |
| snR43 | 221.168487996349 | 1.47177439980748 | 0.499813175353568 | 2.94464906565607 | 0.00323321156910953 | 0.00925828479549371 | 126.995540349137 | 145.917885046364 | 78.273557739976 | 147.240968033244 | 436.816449231608 | 391.766527577767 |
| snR44 | 1250.74809331186 | 1.43277295248386 | 0.407706131717106 | 3.51422959093002 | 0.000441031446034106 | 0.00183113570004361 | 746.09879955118 | 741.130952862607 | 540.59961840974 | 1043.75550120608 | 2135.37249822214 | 2297.53118961942 |
| snR45 | 61.6473033496556 | 1.49523132065865 | 0.485563811767472 | 3.0793714120003 | 0.00207437896501788 | 0.00642877792815934 | 30.2370334164612 | 42.8685312000618 | 23.4089144642919 | 54.0173965565749 | 125.702575318448 | 93.6493691420958 |
| snR46 | 121.153739236587 | 1.39877306221441 | 0.606461689654955 | 2.30644917242874 | 0.021085547096148 | 0.0444342026502594 | 93.7348035910297 | 82.439483077042 | 23.4089144642919 | 82.7685914979777 | 194.838991743595 | 249.731651045589 |
| snR47 | 34.5515462021466 | 0.94543558093297 | 0.533996483533141 | 1.77049027491263 | 0.0766455030371498 | 0.129044977662548 | 25.701478403992 | 25.556239753883 | 19.0197430022371 | 22.6524566204991 | 65.9938520421854 | 48.3855073900828 |
| snR48 | 39.0594467899922 | 1.13026588263382 | 0.560298697753736 | 2.01725595145073 | 0.0436688098362944 | 0.0810920228793933 | 27.9692559102266 | 22.2586604308013 | 22.6773858872828 | 23.5237049520568 | 86.4205205314332 | 51.5071530281527 |
| snR49 | 258.661420942148 | 1.90999944261864 | 0.489696120939574 | 3.90037690916144 | 9.60430479128491e-05 | 0.000541134933122264 | 118.68035615961 | 117.0640659694 | 89.9780149721219 | 172.507169648417 | 496.525172507871 | 557.21374639547 |
| snR5 | 220.112621342357 | 1.21920046369302 | 0.431101725376456 | 2.82810388343577 | 0.00468246091371932 | 0.0126701883547699 | 118.68035615961 | 137.67393673866 | 139.721958208742 | 162.052189669725 | 364.5374684235 | 398.009818853907 |
| snR50 | 34.339529622509 | 0.350928593202853 | 0.582377238915722 | 0.602579513334375 | 0.546788452449055 | 0.63699667759984 | 24.9455525685805 | 44.5173208616027 | 20.4828001562554 | 17.4249666311532 | 50.2810301273793 | 48.3855073900828 |
| snR51 | 237.994283025158 | 1.4460482812337 | 0.479381628154221 | 3.01648664927245 | 0.00255722478745455 | 0.00760733064357408 | 148.917389576071 | 127.781198769415 | 106.071643666323 | 154.210954685706 | 432.102602657166 | 458.881908796269 |
| snR52 | 5.26231248509181 | 1.77279483911008 | 1.02941692792036 | 1.72213492029075 | 0.0850450806080494 | 0.140221046398441 | 1.51185167082306 | 3.29757932308168 | 2.19458573102736 | 2.61374499467298 | 15.712821914806 | 6.24329127613972 |
| snR53 | 5.65245342703427 | -0.0808335616115241 | 1.05163254064444 | -0.07686483489945 | 0.938731072438738 | 0.956252468602574 | 9.07111002493835 | 7.41955347693378 | 0.731528577009121 | 2.61374499467298 | 4.71384657444181 | 9.36493691420958 |
| snR54 | 42.913854071669 | 0.388705655682719 | 0.504344185803384 | 0.770715052585644 | 0.440875847776806 | 0.537327868978544 | 43.0877726184572 | 44.5173208616027 | 23.4089144642919 | 32.2361882676334 | 43.9959013614569 | 70.2370268565718 |
| snR55 | 101.035049226067 | 0.376414050504492 | 0.422428497530826 | 0.891071631541677 | 0.372890742796112 | 0.47105068332816 | 96.7585069326758 | 84.0882727385828 | 81.9312006250216 | 66.2148731983821 | 142.986679424735 | 134.230762437004 |
| snR56 | 25.4968541267927 | 1.50428095439901 | 0.619032499997691 | 2.43005166029994 | 0.0150966708588044 | 0.0336177594504497 | 9.82703586034988 | 16.4878966154084 | 13.1675143861642 | 14.8112216364802 | 53.4235945103405 | 45.263861752013 |
| snR57 | 263.556798124382 | 0.687179730504438 | 0.485262747276714 | 1.41609825679156 | 0.156746738424395 | 0.232538354170261 | 227.53367645887 | 248.142844061896 | 129.480558130614 | 163.79468633284 | 348.824646508694 | 463.564377253374 |
| snR58 | 3.03716684228931 | 0.631112947448439 | 1.36974616351836 | 0.46075175405299 | 0.644976719376761 | 0.724515972926276 | 3.02370334164612 | 3.29757932308168 | 0.731528577009121 | 1.74249666311532 | 9.42769314888362 | 0 |
| snR59 | 13.6920566178802 | -0.0403904246981505 | 0.615530624245729 | -0.0656188711124567 | 0.947681264518065 | 0.962325345868234 | 10.5829616957614 | 12.3659224615563 | 18.288214425228 | 9.58373164713426 | 17.2841041062866 | 14.0474053713144 |
| snR6 | 1.9921379254984 | 1.13189624814043 | 1.37017122327172 | 0.826098394795997 | 0.408748278187548 | 0.506901388701678 | 0.75592583541153 | 0.82439483077042 | 2.19458573102736 | 3.48499332623064 | 1.5712821914806 | 3.12164563806986 |
| snR60 | 3.3917524758727 | 0.680566438449414 | 1.25997644656471 | 0.540142190994884 | 0.589098976166951 | 0.675052919308748 | 5.29148084788071 | 1.64878966154084 | 0.731528577009121 | 1.74249666311532 | 1.5712821914806 | 9.36493691420958 |
| snR61 | 43.9195264217467 | 0.367402589030763 | 0.532371815163853 | 0.690124042193489 | 0.490116185102058 | 0.584400709508842 | 37.7962917705765 | 42.0441363692914 | 34.3818431194287 | 20.9099599573838 | 59.708723276263 | 68.6762040375369 |
| snR62 | 27.8628628397531 | -0.25764065225048 | 0.623211981898344 | -0.413407732415045 | 0.679307910358507 | 0.75257920913621 | 34.0166625935188 | 37.9221622154393 | 18.288214425228 | 9.58373164713426 | 37.7107725955345 | 29.6556335616637 |
| snR63 | 380.084698279687 | 0.697494273518216 | 0.463663789886887 | 1.50431042650187 | 0.132501455669969 | 0.202830847281816 | 331.09551591025 | 327.284747815857 | 210.680230178627 | 232.623304525895 | 529.522098528963 | 649.302292718531 |
| snR64 | 29.0765857486654 | 2.20925678710535 | 0.604934920542352 | 3.65205696031673 | 0.000260148161577691 | 0.00118735234625112 | 12.850739201996 | 9.06834313847462 | 8.77834292410945 | 20.0387116258262 | 62.8512876592241 | 60.8720899423623 |
| snR65 | 4.09202358749281 | 0.65857962052493 | 1.10158905064435 | 0.5978451039793 | 0.549943291941838 | 0.640324498301471 | 4.53555501246918 | 4.1219741538521 | 0.731528577009121 | 2.61374499467298 | 9.42769314888362 | 3.12164563806986 |
| snR66 | 42.6855634140265 | -0.31723544276234 | 0.555724719596479 | -0.570849975852595 | 0.568101342836921 | 0.657189266727101 | 43.8436984538687 | 57.7076381539294 | 39.5025431584925 | 14.8112216364802 | 53.4235945103405 | 46.8246845710479 |
| snR67 | 148.772900714294 | 0.586078677963232 | 0.583689428969445 | 1.00409335662975 | 0.315333617269819 | 0.41103193847171 | 148.16146374066 | 167.352150646395 | 40.9656003125108 | 97.5798131344579 | 232.549764339129 | 206.028612112611 |
| snR68 | 146.50880675605 | 1.00901624106566 | 0.5030250901277 | 2.00589644705298 | 0.044867297413243 | 0.0829587698367169 | 116.412578653376 | 109.644512492466 | 65.1060433538118 | 89.7385781504389 | 271.831819126144 | 226.319308760065 |
| snR69 | 16.4055336283639 | 0.501273281678086 | 0.654081620911155 | 0.766377261877191 | 0.443451858300913 | 0.539956507365831 | 18.1422200498767 | 14.8391069538676 | 7.31528577009121 | 9.58373164713426 | 25.1405150636897 | 23.412342285524 |
| snR7-L | 1.30230218663515 | -0.707342236278144 | 1.91406561735673 | -0.369549627695085 | 0.711718089239057 | 0.778573890725247 | 0.75592583541153 | 2.47318449231126 | 1.46305715401824 | 0 | 0 | 3.12164563806986 |
| snR70 | 327.036521080141 | 1.25814538219615 | 0.472232743161373 | 2.66424850969348 | 0.00771605373082138 | 0.019185863330691 | 230.557379800516 | 186.313231754115 | 160.936286942007 | 208.228351242281 | 601.801079337071 | 574.382797404854 |
| snR71 | 13.7505354437377 | 0.257054237951523 | 0.64547430756942 | 0.398240851629678 | 0.690452655711927 | 0.761650411576706 | 15.1185167082306 | 13.1903172923267 | 8.77834292410945 | 7.84123498401894 | 17.2841041062866 | 20.2906966474541 |
| snR72 | 13.5639362592136 | -0.492162402807652 | 0.706816582592031 | -0.696308511895404 | 0.486235643706122 | 0.580741384545146 | 13.6066650374075 | 24.7318449231126 | 8.77834292410945 | 6.09873832090362 | 10.9989753403642 | 17.1690510093842 |
| snR73 | 12.3561543891407 | -0.368979677740104 | 0.747441753208728 | -0.493656764766076 | 0.621548604254773 | 0.703351434089042 | 18.1422200498767 | 18.1366862769492 | 5.12070003906385 | 6.09873832090362 | 15.712821914806 | 10.9257597332445 |
| snR74 | 10.5608994191635 | -0.159052712353244 | 0.809186742745071 | -0.196558722420088 | 0.844172875106788 | 0.883835687804864 | 12.0948133665845 | 17.3122914461788 | 4.38917146205473 | 13.9399733049226 | 3.14256438296121 | 12.4865825522794 |
| snR75 | 117.923884702081 | 1.39021436473273 | 0.589999301439798 | 2.35629832330333 | 0.0184580874759991 | 0.0397027666484417 | 83.1518418952682 | 84.0882727385828 | 27.7980859263466 | 72.3136115192857 | 240.406175296532 | 199.785320836471 |
| snR76 | 201.557068478868 | 0.907996531582081 | 0.415488762163604 | 2.18536965200649 | 0.028861754160867 | 0.0573671903691308 | 118.68035615961 | 172.298519631018 | 128.749029553605 | 156.824699680379 | 333.111824593888 | 299.677981254707 |
| snR77 | 70.717212563972 | -0.27542170343397 | 0.568261344313777 | -0.484674360116058 | 0.627907356950494 | 0.709425154168629 | 91.4670260847951 | 108.820117661695 | 31.4557288113922 | 37.4636782569794 | 86.4205205314332 | 68.6762040375369 |
| snR78 | 25.0885492517924 | 0.153340264918817 | 0.568538513734336 | 0.269709546872438 | 0.787383715308921 | 0.838279240633072 | 34.7725884289304 | 22.2586604308013 | 13.8990429631733 | 20.0387116258262 | 37.7107725955345 | 21.851519466489 |
| snR79 | 16.4478778970768 | 1.28896411939099 | 0.631512926607243 | 2.04107321494725 | 0.0412435502831042 | 0.0775499164447274 | 12.850739201996 | 7.41955347693378 | 8.04681434710033 | 13.9399733049226 | 36.1394904040539 | 20.2906966474541 |
| snR8 | 311.923781407115 | 2.46394874192257 | 0.504399751267593 | 4.88491268231297 | 1.03474699817314e-06 | 1.59420350914713e-05 | 106.585542793026 | 113.766486646318 | 66.56910050783 | 226.524566204991 | 730.646219038481 | 627.450773252042 |
| snR80 | 22.8801848942294 | 1.00426601282445 | 0.580873641492429 | 1.72888893743605 | 0.0838289743561419 | 0.138852519252457 | 13.6066650374075 | 22.2586604308013 | 9.50987150111857 | 18.2962149627109 | 37.7107725955345 | 35.8989248378034 |
| snR81 | 46.3769173356488 | 1.36078340440118 | 0.597248687756364 | 2.27842008245029 | 0.0227015610090213 | 0.047236850694054 | 32.5048109226958 | 29.6782139077351 | 15.3621001171915 | 25.2662016151721 | 95.8482136803168 | 79.6019637707814 |
| snR82 | 281.849393993626 | 1.06715414863512 | 0.518310976584172 | 2.0589070979511 | 0.0395031390054518 | 0.0748015922361392 | 218.462566433932 | 210.220681846457 | 117.044572321459 | 158.567196343494 | 523.236969763041 | 463.564377253374 |
| snR83 | 539.26212316557 | 2.00441733407534 | 0.467586093840888 | 4.2867342730629 | 1.81318969332708e-05 | 0.000145873123332317 | 209.391456408994 | 204.449918031064 | 231.163030334882 | 376.379279232909 | 1079.47086554717 | 1134.71818943839 |
| snR84 | 511.254870978172 | 1.71814213675461 | 0.506990588246538 | 3.38890341672204 | 0.000701727224768339 | 0.00267403747189829 | 247.18774817957 | 298.430928738892 | 168.983101289107 | 321.490634344776 | 1059.04419705793 | 972.392616258761 |
| snR85 | 61.6952855204898 | 1.22629541235662 | 0.505819161568915 | 2.42437516315709 | 0.0153347545755286 | 0.0340654775668866 | 37.0403659351649 | 32.1513984000464 | 40.9656003125108 | 39.2061749200947 | 109.989753403642 | 110.81842015148 |
| snR86 | 2373.8200701656 | 1.37071041930671 | 0.438496329645403 | 3.12593362050523 | 0.00177241553920866 | 0.00567313920104561 | 1464.98426902754 | 1415.48592443281 | 1090.7091083206 | 1702.41923986367 | 4515.86501831525 | 4053.45686103371 |
| snR87 | 59.1924030156217 | -0.337690161977453 | 0.507471931129345 | -0.66543613796717 | 0.505771577333401 | 0.599515729436242 | 83.1518418952682 | 82.439483077042 | 32.1872573884013 | 38.334926588537 | 62.8512876592241 | 56.1896214852575 |
| snR9 | 259.44634398284 | 1.8720112754284 | 0.483643050193492 | 3.87064649162117 | 0.000108547104640927 | 0.000597472952040657 | 119.436281995022 | 135.200752246349 | 79.0050863169851 | 190.803384611127 | 548.377484826731 | 483.855073900828 |
| SRG1 | 848.663085659421 | 0.830627717601488 | 0.394577074064951 | 2.10510891837765 | 0.0352818281003015 | 0.0678703571380298 | 660.679180149677 | 615.822938585503 | 555.230189949923 | 627.298798721515 | 1382.72832850293 | 1250.21907804698 |
| tD(GUC)D | 1.08092938484763 | -0.710027045533568 | 2.09294799658874 | -0.339247342356727 | 0.73442340755026 | 0.797048659356871 | 0.75592583541153 | 3.29757932308168 | 0 | 0.87124833155766 | 0 | 1.56082281903493 |
| tD(GUC)G2 | 1.4526432364624 | -0.0245725344610229 | 1.6092180555783 | -0.0152698600266403 | 0.987816887894213 | 0.991357450216414 | 1.51185167082306 | 0.82439483077042 | 2.19458573102736 | 2.61374499467298 | 1.5712821914806 | 0 |
| tD(GUC)N | 10.6831972130154 | 2.08052211585692 | 0.710753878127418 | 2.92720473272457 | 0.00342023651197601 | 0.00971249694349791 | 2.26777750623459 | 4.1219741538521 | 5.85222861607297 | 17.4249666311532 | 14.1415397233254 | 20.2906966474541 |
| tD(GUC)O | 23.8338170054262 | 0.766798374630357 | 0.602419856219048 | 1.27286371243304 | 0.203066407902091 | 0.287859931078465 | 15.1185167082306 | 28.0294242461943 | 9.50987150111857 | 18.2962149627109 | 37.7107725955345 | 34.3381020187685 |
| tE(UUC)C | 8.89734513610124 | 1.83378027709133 | 0.751877306204633 | 2.43893553104826 | 0.0147305959610606 | 0.0329506905137999 | 5.29148084788071 | 4.1219741538521 | 2.19458573102736 | 10.4549799786919 | 15.712821914806 | 15.6082281903493 |
| tE(UUC)E2 | 1.72592170093503 | 2.40484224752578 | 1.81934987127009 | 1.32181406418929 | 0.186230073878331 | 0.26812467600636 | 0 | 1.64878966154084 | 0 | 0.87124833155766 | 4.71384657444181 | 3.12164563806986 |
| tE(UUC)G1 | 22.6968090376613 | -1.494339550044 | 0.74931794630888 | -1.99426632900636 | 0.0461229568644965 | 0.0848499142941847 | 50.6470309725725 | 36.2733725538985 | 13.1675143861642 | 7.84123498401894 | 23.5692328722091 | 4.68246845710479 |
| tE(UUC)J | 5.73213406961191 | 0.0805633485575307 | 0.979519586849167 | 0.0822478178478082 | 0.934449649078975 | 0.953399198363213 | 6.04740668329224 | 6.59515864616336 | 3.6576428850456 | 0.87124833155766 | 7.85641095740302 | 9.36493691420958 |
| tE(UUC)K | 9.06560314199768 | -0.127126127118349 | 0.91502918878818 | -0.138931226102971 | 0.889504495383943 | 0.919487792981155 | 15.1185167082306 | 11.5415276307859 | 1.46305715401824 | 4.3562416577883 | 9.42769314888362 | 12.4865825522794 |
| tE(UUC)P | 1.05578340540188 | -0.658053672591179 | 1.86111441836635 | -0.353580449486177 | 0.723653328120173 | 0.788149405224744 | 1.51185167082306 | 1.64878966154084 | 0.731528577009121 | 0.87124833155766 | 1.5712821914806 | 0 |
| tF(GAA)D | 1.69903249274833 | 2.46474917778844 | 1.71444556684544 | 1.43763629796865 | 0.150537276253835 | 0.224776271522072 | 0.75592583541153 | 0 | 0.731528577009121 | 0.87124833155766 | 4.71384657444181 | 3.12164563806986 |
| tF(GAA)F | 1.29890698192942 | 0.0554476303983302 | 2.20734486550544 | 0.0251196046729353 | 0.979959562847888 | 0.985468392370455 | 3.77962917705765 | 0 | 0 | 0.87124833155766 | 3.14256438296121 | 0 |
| tF(GAA)H2 | 1.30428223166682 | 0.532226371676441 | 2.15806959072972 | 0.246621505609778 | 0.805201159477527 | 0.852737291076348 | 0 | 1.64878966154084 | 1.46305715401824 | 0 | 4.71384657444181 | 0 |
| tF(GAA)M | 2.13465665852716 | 2.89013405991621 | 1.55713320051219 | 1.85606090664919 | 0.0634448771454091 | 0.110101053305426 | 0.75592583541153 | 0 | 0.731528577009121 | 3.48499332623064 | 4.71384657444181 | 3.12164563806986 |
| tF(GAA)P1 | 15.1021106420623 | 1.70744610504276 | 0.809472040714429 | 2.10933302098463 | 0.0349158464434985 | 0.0673227697892606 | 10.5829616957614 | 8.2439483077042 | 2.19458573102736 | 6.96998665246128 | 28.2830794466509 | 34.3381020187685 |
| tG(CCC)O | 1.20180802920406 | 1.07597124010985 | 1.77202401407613 | 0.607199017373829 | 0.543718844282453 | 0.634108902060666 | 1.51185167082306 | 0.82439483077042 | 0 | 1.74249666311532 | 1.5712821914806 | 1.56082281903493 |
| tG(GCC)D2 | 18.4007775345242 | 1.64890426575597 | 0.608436603311561 | 2.71006750215457 | 0.00672695161324002 | 0.0170759039768489 | 6.80333251870377 | 12.3659224615563 | 7.31528577009121 | 16.5537182995955 | 37.7107725955345 | 29.6556335616637 |
| tG(GCC)E | 1.84318716317465 | 0.456012027995876 | 1.36284280215777 | 0.334603541416425 | 0.737924173100422 | 0.799635146351458 | 1.51185167082306 | 1.64878966154084 | 1.46305715401824 | 1.74249666311532 | 1.5712821914806 | 3.12164563806986 |
| tG(GCC)G1 | 1.43328269580252 | -2.32688107702441 | 1.84369092980359 | -1.26207762885306 | 0.206920854269653 | 0.292102214269304 | 2.26777750623459 | 3.29757932308168 | 1.46305715401824 | 0 | 1.5712821914806 | 0 |
| tG(GCC)G2 | 1.06916494438371 | -1.14779830767466 | 2.25409247309247 | -0.509206397419867 | 0.610607559343093 | 0.695000474049049 | 3.02370334164612 | 1.64878966154084 | 0 | 1.74249666311532 | 0 | 0 |
| tG(GCC)O1 | 1.72742811338525 | 1.11496770469122 | 1.61239008515944 | 0.691499975690415 | 0.48925139598935 | 0.583801925175359 | 0.75592583541153 | 2.47318449231126 | 0 | 0.87124833155766 | 3.14256438296121 | 3.12164563806986 |
| tG(GCC)O2 | 1.58852256247329 | 3.34753268886306 | 1.90093593549494 | 1.76099184951831 | 0.0782397825149732 | 0.131248814638298 | 0 | 0.82439483077042 | 0 | 0.87124833155766 | 4.71384657444181 | 3.12164563806986 |
| tG(GCC)P1 | 1.89524635049592 | 2.01172153478639 | 1.46280705907786 | 1.3752473522069 | 0.169054772490741 | 0.247041691590736 | 0.75592583541153 | 0.82439483077042 | 0.731528577009121 | 4.3562416577883 | 3.14256438296121 | 1.56082281903493 |
| tH(GUG)E2 | 1.74839175815055 | 1.7122955512618 | 1.76071677907883 | 0.972499138764175 | 0.330802293129028 | 0.426585255857217 | 0 | 2.47318449231126 | 0 | 1.74249666311532 | 4.71384657444181 | 1.56082281903493 |
| tH(GUG)M | 3.28125255087397 | 1.61815844446109 | 1.24483720941457 | 1.2998956266917 | 0.193636744211957 | 0.276931075444149 | 1.51185167082306 | 2.47318449231126 | 0.731528577009121 | 0.87124833155766 | 7.85641095740302 | 6.24329127613972 |
| tK(CUU)E1 | 2.0991285649994 | 3.79179284614314 | 1.80661658997327 | 2.09883650310066 | 0.0358313157970469 | 0.0688200637438062 | 0.75592583541153 | 0 | 0 | 0.87124833155766 | 6.28512876592241 | 4.68246845710479 |
| tK(CUU)M | 3.4163126697757 | 0.0386967074237257 | 1.19943407089442 | 0.0322624714127634 | 0.974262737088717 | 0.981288075527846 | 4.53555501246918 | 3.29757932308168 | 2.19458573102736 | 2.61374499467298 | 7.85641095740302 | 0 |
| tK(CUU)P | 1.57780090715447 | 0.46037833227749 | 1.50489843840653 | 0.305919868429769 | 0.75966565791482 | 0.816074445795334 | 1.51185167082306 | 1.64878966154084 | 0.731528577009121 | 0.87124833155766 | 3.14256438296121 | 1.56082281903493 |
| tK(UUU)D | 21.7822857972138 | 1.05234886601381 | 0.71429882268703 | 1.47326137547744 | 0.14068057123756 | 0.213172442063503 | 19.6540717206998 | 18.9610811077197 | 3.6576428850456 | 14.8112216364802 | 37.7107725955345 | 35.8989248378034 |
| tK(UUU)L | 3.92193580764601 | 1.46863513490353 | 1.21073154669197 | 1.21301467605781 | 0.225124209492014 | 0.313081640135765 | 1.51185167082306 | 1.64878966154084 | 2.92611430803648 | 1.74249666311532 | 14.1415397233254 | 1.56082281903493 |
| tK(UUU)O | 2.2566942129754 | 1.73409341887056 | 1.44274250358104 | 1.20194242185723 | 0.229385836956525 | 0.317482224371378 | 0 | 1.64878966154084 | 1.46305715401824 | 2.61374499467298 | 1.5712821914806 | 6.24329127613972 |
| tK(UUU)P | 10.29872895906 | 2.59857927031929 | 0.898375315328676 | 2.89253191397916 | 0.00382150355799603 | 0.0106446725404388 | 3.02370334164612 | 4.94636898462252 | 0.731528577009121 | 6.09873832090362 | 25.1405150636897 | 21.851519466489 |
| tL(CAA)A | 2.49886109632484 | 0.403942366636689 | 1.29588407785405 | 0.311711806279469 | 0.755259554599691 | 0.812676947639434 | 2.26777750623459 | 3.29757932308168 | 0.731528577009121 | 0.87124833155766 | 3.14256438296121 | 4.68246845710479 |
| tL(CAA)G2 | 1.0182019804642 | -1.93872861785877 | 2.09461674248195 | -0.92557677905388 | 0.354665965598898 | 0.451125581366167 | 0.75592583541153 | 0.82439483077042 | 3.6576428850456 | 0.87124833155766 | 0 | 0 |
| tL(CAA)K | 1.71415726047111 | 1.78718731896614 | 1.76073242069871 | 1.01502493959697 | 0.310093939376715 | 0.405236398049116 | 2.26777750623459 | 0 | 0 | 1.74249666311532 | 4.71384657444181 | 1.56082281903493 |
| tL(CAA)L | 7.43043124131752 | 0.160188825709257 | 0.898874623345255 | 0.178210421730561 | 0.858557725378098 | 0.895601357385108 | 12.850739201996 | 4.94636898462252 | 2.92611430803648 | 3.48499332623064 | 12.5702575318448 | 7.80411409517465 |
| tL(GAG)G | 0.888935261469935 | -1.70974631025335 | 2.35022952604338 | -0.727480567879562 | 0.466931615052986 | 0.562838914410535 | 2.26777750623459 | 0 | 2.19458573102736 | 0.87124833155766 | 0 | 0 |
| tL(UAA)B2 | 1.68892002824919 | 1.18815191069745 | 1.82858684203448 | 0.649765099138262 | 0.515843966165848 | 0.608654294999644 | 0 | 0.82439483077042 | 2.19458573102736 | 0.87124833155766 | 0 | 6.24329127613972 |
| tL(UAA)L | 1.31559703923811 | 0.689165568257081 | 1.71917951106828 | 0.400868881824238 | 0.688516661595379 | 0.760165832448867 | 0 | 0.82439483077042 | 2.19458573102736 | 1.74249666311532 | 1.5712821914806 | 1.56082281903493 |
| tM(CAU)E | 1.31092767197559 | 1.21865991334855 | 1.78964352114417 | 0.680951205617433 | 0.495902365837947 | 0.589989143912133 | 0.75592583541153 | 0.82439483077042 | 0.731528577009121 | 0.87124833155766 | 0 | 4.68246845710479 |
| tM(CAU)J1 | 1.19948504821127 | 1.08671965207548 | 1.77218755577705 | 0.613208036888053 | 0.539738791330854 | 0.631296370535906 | 0.75592583541153 | 0.82439483077042 | 0.731528577009121 | 1.74249666311532 | 3.14256438296121 | 0 |
| tM(CAU)J2 | 3.40517345869869 | 1.72808681828596 | 1.17586444220402 | 1.46963098488366 | 0.141661723716547 | 0.214393072089783 | 0.75592583541153 | 1.64878966154084 | 2.19458573102736 | 1.74249666311532 | 6.28512876592241 | 7.80411409517465 |
| tM(CAU)O2 | 1.43889535624251 | 2.18407532473315 | 1.80077264176361 | 1.21285456813368 | 0.225185428122494 | 0.313081640135765 | 0.75592583541153 | 0 | 0.731528577009121 | 0.87124833155766 | 4.71384657444181 | 1.56082281903493 |
| tN(GUU)K | 5.38956972048656 | 0.926101321087523 | 0.942307739561507 | 0.982801352685986 | 0.325705215985142 | 0.421337794830603 | 3.77962917705765 | 4.94636898462252 | 2.19458573102736 | 2.61374499467298 | 10.9989753403642 | 7.80411409517465 |
| tN(GUU)P | 46.6516342417389 | 2.57643489785913 | 0.557358860431612 | 4.6225781642082 | 3.7899993107339e-06 | 4.40508100064548e-05 | 11.3388875311729 | 13.1903172923267 | 15.3621001171915 | 34.8499332623064 | 105.2759068292 | 99.8926604182355 |
| tP(AGG)C | 1.45437306520273 | 2.14446215484649 | 1.79640460815537 | 1.19375231231928 | 0.232574870726957 | 0.321136828362264 | 0.75592583541153 | 0.82439483077042 | 0 | 0.87124833155766 | 4.71384657444181 | 1.56082281903493 |
| tP(UGG)M | 7.10631621680902 | 1.65616696769334 | 0.866129936371194 | 1.9121460858773 | 0.0558574590866846 | 0.0992508072085782 | 3.02370334164612 | 4.1219741538521 | 2.92611430803648 | 4.3562416577883 | 17.2841041062866 | 10.9257597332445 |
| tP(UGG)N2 | 4.77721939766139 | 1.83644442297495 | 0.958892882995867 | 1.91517160627718 | 0.0554706222622614 | 0.0986825434720893 | 2.26777750623459 | 2.47318449231126 | 1.46305715401824 | 5.22748998934596 | 9.42769314888362 | 7.80411409517465 |
| tP(UGG)O3 | 7.8884308168114 | 0.767358810782407 | 0.848078559650224 | 0.90482043444052 | 0.365560524174129 | 0.462789419241476 | 6.04740668329224 | 8.2439483077042 | 2.92611430803648 | 3.48499332623064 | 14.1415397233254 | 12.4865825522794 |
| tQ(UUG)E1 | 1.56313976673682 | 1.59399483504102 | 1.65973225351368 | 0.960392757124836 | 0.336857581983756 | 0.433006552490098 | 1.51185167082306 | 0 | 0.731528577009121 | 0.87124833155766 | 3.14256438296121 | 3.12164563806986 |
| tR(ACG)D | 1.17271196497128 | 0.348686907547144 | 1.78898577925696 | 0.194907590429236 | 0.845465291852046 | 0.884757308990441 | 0.75592583541153 | 0.82439483077042 | 1.46305715401824 | 0.87124833155766 | 0 | 3.12164563806986 |
| tR(ACG)L | 1.57722718819353 | 2.23499470856681 | 2.13795315252354 | 1.04538993566287 | 0.295842786316428 | 0.390015873874677 | 0 | 1.64878966154084 | 0 | 0 | 1.5712821914806 | 6.24329127613972 |
| tR(UCU)G2 | 9.43180088365819 | 1.02249470636523 | 0.697703507494574 | 1.46551464251193 | 0.142780560405995 | 0.215593624622417 | 7.5592583541153 | 7.41955347693378 | 3.6576428850456 | 11.3262283102496 | 14.1415397233254 | 12.4865825522794 |
| tS(AGA)D3 | 0.912221887228025 | -0.838843100768587 | 2.32994720099228 | -0.360026656574595 | 0.71882719930778 | 0.784485199719048 | 2.26777750623459 | 0 | 1.46305715401824 | 1.74249666311532 | 0 | 0 |
| tS(AGA)G | 1.82127742491945 | 1.27698713647994 | 1.7707283313856 | 0.721164909289445 | 0.470808058836386 | 0.566094828025826 | 2.26777750623459 | 0.82439483077042 | 0 | 0 | 4.71384657444181 | 3.12164563806986 |
| tS(AGA)J | 1.58862264885279 | -0.830424284560799 | 1.60409454835937 | -0.517690360216072 | 0.604674325077994 | 0.68909974402801 | 3.02370334164612 | 2.47318449231126 | 0.731528577009121 | 1.74249666311532 | 0 | 1.56082281903493 |
| tS(GCU)F | 4.46686638895475 | -0.132174767179099 | 0.937327665288944 | -0.141012339733251 | 0.887860194054859 | 0.918313490914086 | 6.04740668329224 | 4.94636898462252 | 2.92611430803648 | 3.48499332623064 | 4.71384657444181 | 4.68246845710479 |
| tS(GCU)O | 2.9163182309126 | 1.77342685083643 | 1.28952924680722 | 1.37525136031408 | 0.169053530305207 | 0.247041691590736 | 2.26777750623459 | 1.64878966154084 | 0 | 2.61374499467298 | 6.28512876592241 | 4.68246845710479 |
| tS(UGA)E | 5.42186157282378 | 0.588487881527673 | 0.846258510886159 | 0.695399660928005 | 0.48680487298486 | 0.581313440019006 | 3.77962917705765 | 4.1219741538521 | 5.12070003906385 | 6.96998665246128 | 7.85641095740302 | 4.68246845710479 |
| tT(AGU)I1 | 2.26389393895359 | 1.35433877441502 | 1.32581899795654 | 1.02151106335212 | 0.307012370204887 | 0.402188703034881 | 2.26777750623459 | 0.82439483077042 | 0.731528577009121 | 3.48499332623064 | 4.71384657444181 | 1.56082281903493 |
| tT(AGU)N1 | 1.49582672215188 | 0.89026447557931 | 1.65756045284222 | 0.537093216752833 | 0.591203226099157 | 0.677102752281446 | 0.75592583541153 | 2.47318449231126 | 0 | 2.61374499467298 | 1.5712821914806 | 1.56082281903493 |
| tT(CGU)K | 2.08168421100814 | 0.483827172082971 | 1.5806369886368 | 0.306096324178926 | 0.75953130657594 | 0.816074445795334 | 2.26777750623459 | 0 | 2.92611430803648 | 2.61374499467298 | 0 | 4.68246845710479 |
| tV(AAC)E2 | 1.74815494185983 | 0.674874818689414 | 1.59354390342473 | 0.423505632470509 | 0.67192638997025 | 0.746842587402212 | 0.75592583541153 | 3.29757932308168 | 0 | 1.74249666311532 | 1.5712821914806 | 3.12164563806986 |
| tV(AAC)G3 | 4.14922416757861 | 0.514892740049273 | 1.07620114177464 | 0.478435415149459 | 0.632340332051114 | 0.713626719311802 | 4.53555501246918 | 2.47318449231126 | 2.92611430803648 | 0.87124833155766 | 6.28512876592241 | 7.80411409517465 |
| tV(AAC)L | 1.84424054800794 | 2.61936040770036 | 1.63756155382894 | 1.59954928202594 | 0.109698607485293 | 0.173321646762829 | 0.75592583541153 | 0 | 0.731528577009121 | 1.74249666311532 | 4.71384657444181 | 3.12164563806986 |
| tV(AAC)O | 2.14557509188067 | 1.16638421167986 | 1.4410745987832 | 0.809385032991852 | 0.41829370854671 | 0.515660697366158 | 1.51185167082306 | 2.47318449231126 | 0 | 2.61374499467298 | 4.71384657444181 | 1.56082281903493 |
| tV(UAC)D | 1.92074219720607 | -0.164285004873269 | 1.53886234253814 | -0.106757440436357 | 0.91498141205851 | 0.939040684248096 | 3.02370334164612 | 0 | 2.92611430803648 | 0.87124833155766 | 3.14256438296121 | 1.56082281903493 |
| tW(CCA)G1 | 2.75829175594391 | -1.04017046954183 | 1.34624304925965 | -0.772646863516852 | 0.439731401746898 | 0.536745683709254 | 2.26777750623459 | 5.77076381539294 | 2.92611430803648 | 0.87124833155766 | 4.71384657444181 | 0 |
| tW(CCA)K | 1.56232319819425 | 0.481484790872664 | 1.50453329434618 | 0.32002268921666 | 0.748951130804344 | 0.808319973584712 | 1.51185167082306 | 0.82439483077042 | 1.46305715401824 | 0.87124833155766 | 3.14256438296121 | 1.56082281903493 |
| tW(CCA)P | 6.67372474462862 | 0.42122945426723 | 0.852224304385 | 0.494270642247414 | 0.621115055641302 | 0.703331205853667 | 4.53555501246918 | 9.06834313847462 | 3.6576428850456 | 8.7124833155766 | 3.14256438296121 | 10.9257597332445 |
| tX(XXX)D | 2.66866541175807 | 3.19787935083333 | 1.50469167955453 | 2.12527216989734 | 0.0335639090707667 | 0.0651058959083548 | 0.75592583541153 | 0.82439483077042 | 0 | 3.48499332623064 | 3.14256438296121 | 7.80411409517465 |
| tY(GUA)D | 2.71755797588213 | 0.108887633642918 | 1.21182674551569 | 0.0898541264630867 | 0.928403135405889 | 0.948432137058046 | 3.02370334164612 | 1.64878966154084 | 2.92611430803648 | 0.87124833155766 | 4.71384657444181 | 3.12164563806986 |
| tY(GUA)J1 | 1.5816403058738 | 0.92948608013921 | 2.11829797461738 | 0.438789108650827 | 0.660814352773546 | 0.737930367931618 | 0 | 2.47318449231126 | 0.731528577009121 | 0 | 6.28512876592241 | 0 |
| YAL001C | 390.836195723052 | -0.789638025481356 | 0.294814734491797 | -2.67842116793293 | 0.00739701420463029 | 0.0185284992134652 | 511.761790573605 | 506.178426093038 | 466.715232131819 | 246.563277830818 | 293.829769806873 | 319.968677902161 |
| YAL002W | 898.838809153344 | 0.579088526382543 | 0.342361690898033 | 1.69145246614352 | 0.0907504189933582 | 0.147845357530288 | 570.724005735705 | 632.310835200912 | 959.033964458958 | 980.154373002367 | 1187.88933675934 | 1062.92033976279 |
| YAL003W | 49681.7070437512 | -0.0172576767616827 | 0.524248475638079 | -0.0329188878244764 | 0.973739270652144 | 0.981204960569521 | 48922.0082161634 | 46257.618349359 | 54758.5716320178 | 92679.041269446 | 25996.8638580466 | 29476.1389374747 |
| YAL005C | 11630.0288709197 | 1.69007649250065 | 0.594574635710886 | 2.84249678844769 | 0.00447616820806302 | 0.0121682242549286 | 2790.12225850396 | 2408.88169551117 | 11310.163329138 | 21061.5571670749 | 15990.9388626981 | 16218.509912592 |
| YAL007C | 4376.59443807636 | 0.759576345115122 | 0.291717687387609 | 2.60380627557171 | 0.00921948520841067 | 0.0222372602030579 | 3160.5259178556 | 3241.52047458929 | 3348.20629697075 | 4442.49524261251 | 5876.59539613746 | 6190.22330029253 |
| YAL008W | 794.778870214443 | 1.7712852146434 | 0.448708281626451 | 3.94752066581644 | 7.89647037570357e-05 | 0.000467401371503042 | 325.048109226958 | 342.948249600495 | 411.850588856135 | 583.736382143632 | 1499.0032106725 | 1606.08668078694 |
| YAL009W | 791.591395363387 | 1.13584677684711 | 0.369023840396527 | 3.07797668472208 | 0.00208411220239629 | 0.00645583577846662 | 431.633652019983 | 483.095370831466 | 569.860761490105 | 707.45364522482 | 1304.1642189289 | 1253.34072368505 |
| YAL010C | 299.346137622397 | -1.77490719131731 | 0.559538477861973 | -3.17209139592924 | 0.00151345368430963 | 0.00496264853714562 | 451.287723740683 | 416.319389539062 | 524.50598971554 | 261.374499467298 | 83.277956148472 | 59.3112671233273 |
| YAL011W | 93.4379674432223 | -0.925490115796913 | 0.555743544735719 | -1.66531869702063 | 0.0958491902707484 | 0.154433021101731 | 154.964796259364 | 164.878966154084 | 46.8178289285837 | 43.562416577883 | 84.8492383399526 | 65.554558399467 |
| YAL012W | 24041.8872322521 | 0.924889598004461 | 0.381561525377354 | 2.4239592739068 | 0.0153523270605972 | 0.0340927538862918 | 12679.1440373576 | 13790.4767291276 | 23297.7221205865 | 39017.1140321467 | 25778.4556334308 | 29688.4108408634 |
| YAL013W | 490.159261325762 | -0.0881068246508292 | 0.4607903371701 | -0.191208056123592 | 0.848362592189102 | 0.887212584231538 | 611.544000847927 | 659.515864616336 | 245.062073298056 | 607.260087095689 | 424.246191699763 | 393.327350396802 |
| YAL014C | 1739.1569031928 | 1.7840985835863 | 0.402011397309059 | 4.437930356026 | 9.0827995254871e-06 | 8.50170999015124e-05 | 655.387699301796 | 617.471728247044 | 1074.6154796264 | 1795.64281134034 | 3070.2854021531 | 3221.5382984881 |
| YAL015C | 727.986416824866 | 0.576540716616401 | 0.316401285298928 | 1.82218196766078 | 0.0684273797411931 | 0.117231265105955 | 504.958458054902 | 568.008038400819 | 681.053105195492 | 1046.36924620075 | 773.070838208457 | 794.45881488878 |
| YAL016C-A | 23.024977664558 | 0.86193070712075 | 0.570130388535534 | 1.51181330525943 | 0.130581363631225 | 0.200320148114599 | 13.6066650374075 | 11.5415276307859 | 23.4089144642919 | 19.1674632942685 | 28.2830794466509 | 42.1422161139431 |
| YAL016W | 6231.11617704307 | 0.947582597031087 | 0.278466550835146 | 3.402859676285 | 0.000666845195298439 | 0.00256081279530229 | 4168.17505645917 | 4384.95610486786 | 4212.14154641852 | 6941.23545751987 | 8788.18129695102 | 8892.007600042 |
| YAL017W | 777.589388446987 | -0.418276423671577 | 0.403525181374251 | -1.03655593994677 | 0.299942856202243 | 0.394532678501317 | 748.366577057414 | 845.00470153968 | 1076.81006535743 | 985.381862991713 | 487.097479358987 | 522.875644376702 |
| YAL018C | 19.3374504435718 | 0.0721274702163933 | 0.546494130608952 | 0.131982149810141 | 0.894998410615552 | 0.923668271006776 | 17.3862942144652 | 18.1366862769492 | 20.4828001562554 | 13.0687249733649 | 18.8553862977672 | 28.0948107426287 |
| YAL019W | 350.591298432516 | -3.2443402164717 | 0.558495044565338 | -5.80907610200317 | 6.28185319714479e-09 | 3.67773950814659e-07 | 824.715086433979 | 793.067827201144 | 286.759202187575 | 109.777289776265 | 39.2820547870151 | 49.9463302091178 |
| YAL020C | 2180.15156876198 | 2.24641336247452 | 0.438843053897165 | 5.11894478567029 | 3.07249889137513e-07 | 6.6001848490919e-06 | 769.532500448937 | 707.33076480102 | 799.560734670969 | 1729.42793814195 | 4754.69991142031 | 4320.35756308869 |
| YAL021C | 1422.40190580276 | 0.258573035956239 | 0.469591028656913 | 0.550634531276693 | 0.581884232336443 | 0.668927964342501 | 1102.89579386542 | 1201.96766326327 | 1582.29631207073 | 2616.35873966765 | 977.337523100936 | 1053.55540284858 |
| YAL022C | 3563.84242142391 | 1.20442611617708 | 0.371776511744075 | 3.23965091427343 | 0.00119676117054998 | 0.00411047570044898 | 2156.65640842909 | 2263.78820529557 | 2049.74307277956 | 3037.17168381 | 6005.44053583887 | 5870.25462239037 |
| YAL023C | 14048.6036204517 | 0.982059561899992 | 0.280632619667432 | 3.49944907710228 | 0.000466220647432954 | 0.00190753555874728 | 8946.38226209545 | 9130.1727507824 | 10254.5675925139 | 21117.3170602946 | 16319.3368407175 | 18523.8452163065 |
| YAL024C | 216.36930988518 | -0.94379988688499 | 0.3083536525311 | -3.06077090100238 | 0.00220767946519232 | 0.00675734589155823 | 303.126260000023 | 302.552902892744 | 248.719716183101 | 152.46845802259 | 155.55693695658 | 135.791585256039 |
| YAL025C | 402.313196112604 | -3.92395333251132 | 0.833002555559992 | -4.71061379862564 | 2.46971849886062e-06 | 3.15575141521079e-05 | 1056.02839206991 | 1092.32315077081 | 117.776100898468 | 105.421048118477 | 28.2830794466509 | 14.0474053713144 |
| YAL026C | 1009.10127209258 | -0.878171039080541 | 0.391651510504474 | -2.24222558965596 | 0.0249467960857696 | 0.0509699767742246 | 1359.91057790534 | 1394.05165883278 | 1168.98266606058 | 1008.03431961221 | 678.793906719621 | 444.834503424955 |
| YAL026C-A | 45.5116254614066 | -0.767953991574325 | 0.445487528767461 | -1.72385070733414 | 0.0847347978692729 | 0.139956937234706 | 55.1825859850417 | 48.6392950154547 | 68.0321576618483 | 32.2361882676334 | 45.5671835529375 | 23.412342285524 |
| YAL027W | 180.206443140983 | -1.10909090636758 | 0.361934918556695 | -3.06433795001145 | 0.0021815232402437 | 0.00670597120151286 | 273.645152418974 | 220.113419815702 | 246.525130452074 | 149.854713027917 | 102.133342446239 | 88.966900684991 |
| YAL028W | 215.275310272093 | 0.155152291621186 | 0.491396792183953 | 0.315737290289646 | 0.752201919887676 | 0.810334620956279 | 157.98849960101 | 154.986228184839 | 299.19518799673 | 361.568057596429 | 160.270783531022 | 157.643104722528 |
| YAL029C | 1584.52472831787 | -1.23079365352076 | 0.437516531164056 | -2.81313634080547 | 0.00490608444586643 | 0.0131646599297416 | 2434.83711586054 | 2401.46214203423 | 1832.47908540785 | 1521.19958689967 | 678.793906719621 | 638.376532985286 |
| YAL030W | 1142.74692485553 | 1.32992317332181 | 0.416392305444671 | 3.19391870582614 | 0.00140355635673353 | 0.00466644446947029 | 541.242898154655 | 522.666322708446 | 886.612635335055 | 1015.00430626467 | 1849.39913937267 | 2041.55624729769 |
| YAL031C | 371.041733931489 | -1.53995683568083 | 0.560463888979059 | -2.74764684391355 | 0.00600246234749113 | 0.0155619394194214 | 516.297345586075 | 575.427591877753 | 566.934647182069 | 364.181802591102 | 75.421545191069 | 127.987471160864 |
| YAL031W-A | 2.7874178531564 | 1.06148100070556 | 1.18429145764891 | 0.896300478948688 | 0.370092303386601 | 0.46797455994693 | 3.02370334164612 | 1.64878966154084 | 0.731528577009121 | 3.48499332623064 | 4.71384657444181 | 3.12164563806986 |
| YAL032C | 171.147361726277 | -2.19179515333798 | 0.598859397260417 | -3.65994950294629 | 0.000252264956547546 | 0.00115711276365114 | 387.034027730703 | 374.27525316977 | 81.9312006250216 | 81.89734316642 | 43.9959013614569 | 57.7504443042924 |
| YAL033W | 841.256705318191 | 0.506633098709122 | 0.30451658157502 | 1.66372910167556 | 0.0961665778551914 | 0.154905643168442 | 707.546581945192 | 648.79873181632 | 727.870934124075 | 788.479740059682 | 1030.76111761128 | 1144.0831263526 |
| YAL034C | 585.011904405667 | -0.440733153990537 | 0.562063175581906 | -0.78413454774767 | 0.432961165651721 | 0.530190132496118 | 474.72142463844 | 464.958684554517 | 1082.6622939735 | 881.703311536352 | 298.543616381315 | 307.482095349881 |
| YAL034C-B | 2.32234468190736 | -0.0128617409292923 | 1.31902974795817 | -0.00975091043185489 | 0.992220022399888 | 0.994376559147228 | 3.77962917705765 | 0.82439483077042 | 2.19458573102736 | 0.87124833155766 | 3.14256438296121 | 3.12164563806986 |
| YAL034W-A | 47.1483509522476 | -2.33394267821352 | 0.694516845830249 | -3.36052709480854 | 0.000777938999007289 | 0.00290262291634237 | 90.7111002493835 | 60.1808226462406 | 87.0519006640854 | 33.9786849307487 | 6.28512876592241 | 4.68246845710479 |
| YAL035W | 7698.21781939101 | 1.26765932346725 | 0.350642255896246 | 3.61524973716329 | 0.000300058221893334 | 0.00133913717878938 | 4490.9553881799 | 4972.74961920717 | 4089.976274058 | 7261.85484353309 | 12582.8277893767 | 12790.9430019913 |
| YAL036C | 8448.24989181168 | 1.94122546462801 | 0.358688239330132 | 5.41201314058512 | 6.23201145610168e-08 | 1.94825989210169e-06 | 3354.79885755637 | 3402.27746658952 | 3714.70211405232 | 8656.72342235691 | 14711.9151588329 | 16849.0823314821 |
| YAL037C-A | 3.24436096984395 | 0.0771313026968328 | 1.15932739961926 | 0.0665310789015804 | 0.946955015130597 | 0.962042955898571 | 1.51185167082306 | 3.29757932308168 | 4.38917146205473 | 0.87124833155766 | 4.71384657444181 | 4.68246845710479 |
| YAL037C-B | 69.6657663661575 | 0.682440578855752 | 0.609698320201203 | 1.11930860926522 | 0.263008504233065 | 0.353754128500614 | 106.585542793026 | 22.2586604308013 | 31.4557288113922 | 77.5411015086317 | 98.990778063278 | 81.1627865898164 |
| YAL037W | 133.320185060062 | -0.371286515476283 | 0.391792162623013 | -0.947661926135922 | 0.34330158958461 | 0.439622636095624 | 150.429241246894 | 148.391069538676 | 153.621001171915 | 159.438444675052 | 111.561035595123 | 76.4803181327116 |
| YAL038W | 357930.15277659 | 1.18186087971802 | 0.37013191887603 | 3.19308014101282 | 0.00140763874227135 | 0.004677602425298 | 166607.565976372 | 179313.295246043 | 311094.963358938 | 567777.72645449 | 441410.878359497 | 481376.487264201 |
| YAL039C | 2351.76393979447 | 1.3690658045064 | 0.335627197675656 | 4.07912652486955 | 4.52052300943932e-05 | 0.000301368200629288 | 1106.67542304248 | 1079.13283347848 | 1752.01094193684 | 3153.04771190717 | 3621.80545136279 | 3397.91127703904 |
| YAL040C | 4814.48996607245 | -0.400136923877859 | 0.316343626576632 | -1.26488062430089 | 0.205914123983296 | 0.291127762558162 | 5163.72938169616 | 6131.84875127038 | 5137.52519633506 | 3662.7279858684 | 5241.79739077929 | 3549.31109048543 |
| YAL041W | 552.783617116839 | -2.4781025931702 | 0.57558340403663 | -4.30537533881448 | 1.66702845679078e-05 | 0.000137109364773086 | 1001.60173192028 | 975.259084801406 | 837.600220675444 | 336.301855981257 | 73.8502629995884 | 92.0885463230609 |
| YAL042C-A | 5.26213486668804 | 0.11561113784833 | 1.04435582195979 | 0.1107009080788 | 0.911853525864832 | 0.937174705804264 | 4.53555501246918 | 0.82439483077042 | 9.50987150111857 | 2.61374499467298 | 6.28512876592241 | 7.80411409517465 |
| YAL042W | 11348.7036304498 | 1.70748978514977 | 0.362526227975476 | 4.70997586763646 | 2.47746123930988e-06 | 3.15937631310012e-05 | 4904.44682015 | 4926.58350868403 | 6130.20947533643 | 11314.0308336078 | 19677.1668839116 | 21139.7842610091 |
| YAL043C | 1209.05288387901 | -0.237440323398311 | 0.614178699164209 | -0.386598108533275 | 0.69905375845204 | 0.768860436621827 | 1061.31987291779 | 1159.92352689398 | 1705.19311300826 | 2307.06558196468 | 474.527221827142 | 546.287986662226 |
| YAL044C | 1370.14317106783 | 1.20025149422149 | 0.41194111608896 | 2.91364820685269 | 0.00357232222147745 | 0.0100769842778426 | 610.032149177104 | 615.822938585503 | 1267.0074953798 | 2129.33092232692 | 1552.42680518284 | 2046.23871575479 |
| YAL044W-A | 1116.14113761007 | 0.750283801086616 | 0.357573266644856 | 2.09826592498539 | 0.0358816608408138 | 0.0688962122286347 | 765.752871271879 | 812.028908308863 | 918.068364146447 | 920.038238124888 | 1657.70271201204 | 1623.25573179633 |
| YAL045C | 1.46917433025591 | 2.27993325612135 | 1.87532738773767 | 1.21575212468463 | 0.224079361301593 | 0.31194792191575 | 0.75592583541153 | 0 | 0.731528577009121 | 2.61374499467298 | 4.71384657444181 | 0 |
| YAL046C | 860.662215666871 | 1.29084540539164 | 0.344017036755737 | 3.75227174085621 | 0.000175239364192759 | 0.000872225297071747 | 476.989202144675 | 474.027027692991 | 546.451847025813 | 852.952116594949 | 1315.16319426927 | 1498.38990627353 |
| YAL047C | 24.3677120049407 | -2.66173090683036 | 0.809782094692095 | -3.28697179682941 | 0.00101270975638469 | 0.00360522434003174 | 59.7181409975108 | 50.2880846769956 | 17.5566858482189 | 13.9399733049226 | 3.14256438296121 | 1.56082281903493 |
| YAL047W-A | 16.2988224819762 | 1.35487343670825 | 0.664694821472861 | 2.03833908876566 | 0.0415160332779012 | 0.0779939481650187 | 9.82703586034988 | 10.7171328000155 | 6.58375719308209 | 9.58373164713426 | 31.4256438296121 | 29.6556335616637 |
| YAL048C | 407.786469339683 | -0.571081102419764 | 0.546835760142768 | -1.04433752150859 | 0.296329258899476 | 0.390450033910759 | 409.711802793049 | 443.524418954486 | 610.826361802616 | 619.457563737496 | 163.413347913983 | 199.785320836471 |
| YAL049C | 2758.27885627417 | 1.37091029768899 | 0.339287786650409 | 4.04055303971648 | 5.33253028464005e-05 | 0.000342046763277709 | 1395.43909216968 | 1394.05165883278 | 1824.43227106075 | 2907.35568240791 | 4310.0270512313 | 4718.36738194259 |
| YAL051W | 695.13601948014 | -0.496439174744981 | 0.299576829171008 | -1.65713475277354 | 0.097492234335694 | 0.156667137637333 | 873.850265735728 | 807.906934155011 | 757.863605781449 | 476.57283736204 | 661.509802613334 | 593.112671233273 |
| YAL053W | 7532.10361953046 | -0.433833943983107 | 0.266197056768104 | -1.62974733549003 | 0.103154908536152 | 0.164598020558181 | 8396.82417975127 | 9361.00330339811 | 8210.67674835037 | 6437.65392187955 | 6830.36368636618 | 5956.09987743729 |
| YAL054C | 442.635682511005 | 1.45334611368543 | 0.446578604192261 | 3.25440157688285 | 0.00113631527199227 | 0.0039556055954758 | 201.832198054878 | 215.99144566185 | 291.879902226639 | 326.718124334122 | 826.494432718797 | 792.897992069744 |
| YAL055W | 63.9046175743221 | -0.436505462299695 | 0.429554745679016 | -1.01618121250108 | 0.309543101273365 | 0.404680790134078 | 61.2299926683339 | 56.0588484923885 | 103.145529358286 | 54.8886448881326 | 61.2800054677435 | 46.8246845710479 |
| YAL056C-A | 3.89594985438075 | 1.2176979180588 | 1.22587895242007 | 0.993326392997352 | 0.320550919483508 | 0.416057365104276 | 4.53555501246918 | 0.82439483077042 | 1.46305715401824 | 0.87124833155766 | 10.9989753403642 | 4.68246845710479 |
| YAL056W | 1519.00365896427 | 0.0336029815524986 | 0.304358813171997 | 0.110405810833245 | 0.912087544925783 | 0.937265643740553 | 1557.20722094775 | 1713.9168531717 | 1233.35718083738 | 1663.21306494357 | 1330.87601618407 | 1615.45161770115 |
| YAL058W | 736.325051904889 | -0.0274698866275476 | 0.602600149269805 | -0.0455855954580064 | 0.963640550396199 | 0.974842152772781 | 647.828440947681 | 693.316052677923 | 890.2702782201 | 1505.51711693164 | 309.542591721679 | 371.475830930313 |
| YAL059C-A | 6.97881555457154 | 0.789272842083509 | 0.843643858661258 | 0.935552169295668 | 0.349503810974279 | 0.446057182456273 | 8.31518418952682 | 3.29757932308168 | 3.6576428850456 | 7.84123498401894 | 4.71384657444181 | 14.0474053713144 |
| YAL059W | 1323.02954553426 | -0.237416054618257 | 0.384075374937117 | -0.618149639656352 | 0.536476703187098 | 0.628737027939019 | 1594.24758688292 | 1558.10623015609 | 1144.11069444227 | 1720.71545482638 | 881.489309420619 | 1039.50799747726 |
| YAL060W | 3582.93746583927 | 2.16966172423812 | 0.423115037124188 | 5.12782939359659 | 2.93101865443365e-07 | 6.37694599140295e-06 | 1194.36281995022 | 1230.82148234024 | 1483.5399541745 | 3033.68669048377 | 7584.57913827687 | 6970.63470981 |
| YAL061W | 781.475746482706 | 1.49729289225009 | 0.343770634522152 | 4.35549969045889 | 1.32763704544199e-05 | 0.000114304579848214 | 375.69514019953 | 319.865194338923 | 530.358218331613 | 973.184386349906 | 1208.31600524858 | 1281.43553442768 |
| YAL062W | 428.19051970119 | 1.52376653016347 | 0.434778048241802 | 3.50469978032566 | 0.00045712245073184 | 0.00188123238770974 | 248.699599850393 | 223.410999138784 | 190.197430022371 | 324.975627671007 | 815.495457378433 | 766.364004146151 |
| YAL063C | 254.79648814225 | 0.901875748742757 | 0.443030410661716 | 2.03569715992115 | 0.0417807744649555 | 0.0783541606156998 | 119.436281995022 | 151.688648861757 | 261.155701992256 | 221.297076215646 | 389.67798348719 | 385.523236301628 |
| YAL063C-A | 74.0110548234913 | 0.78332912037297 | 0.430361056130082 | 1.82016729723844 | 0.0687335316513244 | 0.117661867047987 | 66.5214735162146 | 46.1661105231435 | 49.7439432366202 | 59.2448865459209 | 113.132317786603 | 109.257597332445 |
| YAL064C-A | 12.8059508032766 | 0.0671973104894725 | 0.634718487099977 | 0.1058694710414 | 0.915685916486559 | 0.939613974215015 | 8.31518418952682 | 10.7171328000155 | 18.288214425228 | 11.3262283102496 | 14.1415397233254 | 14.0474053713144 |
| YAL064W | 15.4803501406542 | 1.2356491213495 | 0.779325541921722 | 1.58553653753288 | 0.112844482238178 | 0.177638344075743 | 14.3625908728191 | 4.1219741538521 | 8.77834292410945 | 6.09873832090362 | 31.4256438296121 | 28.0948107426287 |
| YAL064W-B | 14.4142448045253 | 0.957013626710509 | 0.609621191792477 | 1.56984966991812 | 0.116450088954783 | 0.182245096687436 | 6.04740668329224 | 14.0147121230971 | 9.50987150111857 | 20.9099599573838 | 15.712821914806 | 20.2906966474541 |
| YAL065C | 11.5121305826221 | 0.632846744978736 | 0.751448827483698 | 0.842168783598861 | 0.399693484332941 | 0.498359349294122 | 3.77962917705765 | 9.06834313847462 | 13.8990429631733 | 7.84123498401894 | 21.9979506807285 | 12.4865825522794 |
| YAL067C | 133.68151528019 | 0.982761350434804 | 0.390115309577222 | 2.51915607080339 | 0.0117636501374112 | 0.0273001466251994 | 80.1281385536221 | 84.0882727385828 | 104.608586512304 | 126.331008075861 | 168.127194488425 | 238.805891312344 |
| YAL068C | 1.89186998466984 | 0.243878799625533 | 1.49452749307503 | 0.163181206605805 | 0.870375762823191 | 0.904616725573948 | 0.75592583541153 | 2.47318449231126 | 2.19458573102736 | 4.3562416577883 | 1.5712821914806 | 0 |
| YAL069W | 1.78179660803807 | -3.91235838737852 | 1.8185774926447 | -2.1513289388009 | 0.0314502456746549 | 0.0616371217726043 | 5.29148084788071 | 2.47318449231126 | 2.92611430803648 | 0 | 0 | 0 |
| YAR002C-A | 10770.4942646658 | 1.62614111929273 | 0.418165621098859 | 3.88874894837012 | 0.00010076228026322 | 0.000562800594011395 | 5692.12154064882 | 5939.76475570087 | 4179.95428903012 | 8852.75429695738 | 19892.4325441444 | 20065.9381615131 |
| YAR002W | 711.362129811954 | -1.67246017317436 | 0.392180622973621 | -4.26451506067104 | 2.00336831581161e-05 | 0.00015638414489487 | 1317.5787311223 | 1291.00230498648 | 640.81903345999 | 370.280540912005 | 347.253364317213 | 301.238804073742 |
| YAR003W | 1055.26481707427 | 0.960705842325874 | 0.380120645810813 | 2.52737085689374 | 0.0114920080146296 | 0.0267795507351822 | 693.183991072373 | 785.64827372421 | 669.348647963346 | 843.368384947815 | 1623.13450379946 | 1716.90510093842 |
| YAR007C | 1554.69719546308 | -0.42367183728893 | 0.426795026537966 | -0.992682226701727 | 0.320864839046167 | 0.416354939241852 | 1748.45645730687 | 1770.80009649486 | 1826.62685679178 | 2128.45967399536 | 875.204180654696 | 978.635907534901 |
| YAR008W | 601.779283616412 | 0.380318230217865 | 0.46569149957762 | 0.816674194317078 | 0.414114642384463 | 0.511978939711258 | 586.598448279347 | 599.335041970095 | 381.857917198761 | 323.233131007892 | 881.489309420619 | 838.161853821757 |
| YAR009C | 54.584222474606 | -2.0708423936409 | 0.600735517909365 | -3.4471782205382 | 0.000566474762270356 | 0.00224636543658934 | 96.0025810972642 | 109.644512492466 | 60.716871891757 | 39.2061749200947 | 12.5702575318448 | 9.36493691420958 |
| YAR014C | 810.382929332378 | -1.96355992924329 | 0.491261443453654 | -3.99697545046303 | 6.41569515858986e-05 | 0.000398045056082068 | 1430.21168059861 | 1393.22726400201 | 1049.01197943108 | 581.122637148959 | 202.695402700998 | 206.028612112611 |
| YAR015W | 1073.23612374629 | -0.0144053796084877 | 0.543363144799818 | -0.026511513977995 | 0.978849350001426 | 0.984659452360073 | 945.663220099823 | 984.327427939881 | 1307.2415671153 | 2026.52361920312 | 523.236969763041 | 652.423938356601 |
| YAR018C | 369.748967925349 | -2.15083015576647 | 0.721929265596793 | -2.97928101583257 | 0.00288925666708979 | 0.00843081691710841 | 683.356955212023 | 700.735606154857 | 428.675746127345 | 318.005641018546 | 47.1384657444181 | 40.5813932949082 |
| YAR019C | 465.974403355957 | -0.72866216125013 | 0.394977198484735 | -1.84482082521605 | 0.0650636069952664 | 0.112433291381 | 473.209572967617 | 523.490717539216 | 748.353734280331 | 485.285320677616 | 307.971309530198 | 257.535765140763 |
| YAR019W-A | 6.44809868668839 | -0.278122092463309 | 0.851499366568944 | -0.326626305764597 | 0.74395054475348 | 0.804946489955042 | 3.77962917705765 | 7.41955347693378 | 10.2414000781277 | 7.84123498401894 | 6.28512876592241 | 3.12164563806986 |
| YAR020C | 90.8845062812317 | 1.19561302216127 | 0.478544806484066 | 2.49843485074178 | 0.012474306929617 | 0.0286193575442584 | 52.9148084788071 | 40.3953467077506 | 71.6898005468939 | 67.0861215299398 | 158.699501339541 | 154.521459084458 |
| YAR023C | 241.107530261453 | 1.2630503294191 | 0.356390398905451 | 3.54400773224586 | 0.000394093687420038 | 0.00167131248889363 | 118.68035615961 | 133.551962584808 | 172.640744174153 | 260.50325113574 | 406.962087593476 | 354.306779920929 |
| YAR027W | 2509.79104881686 | 0.513131188094727 | 0.311796829273987 | 1.64572291927902 | 0.0998208181137378 | 0.159991555164876 | 2121.12789416475 | 2034.6064423414 | 2047.54848704853 | 2218.1982521458 | 3379.82799387478 | 3257.4372233259 |
| YAR028W | 1742.30112216787 | 0.934097940659226 | 0.32413704861971 | 2.88179936430268 | 0.00395411423161317 | 0.0109524712479952 | 1266.17577431431 | 1297.59746363264 | 1027.06612212081 | 1738.14042145753 | 2441.77252556086 | 2683.05442592104 |
| YAR029W | 112.362867088282 | 0.205084838703331 | 0.346426506158425 | 0.592001001821562 | 0.553849917540162 | 0.643915151837855 | 102.805913615968 | 84.9126675693532 | 125.09138666856 | 112.391034770938 | 120.988728744006 | 127.987471160864 |
| YAR030C | 38.7288229125463 | 0.675691253804777 | 0.472485911548347 | 1.43007703994924 | 0.152694909124839 | 0.227470556272025 | 34.7725884289304 | 32.9757932308168 | 21.2143287332645 | 32.2361882676334 | 53.4235945103405 | 57.7504443042924 |
| YAR031W | 161.195769007673 | 1.17372125005645 | 0.322363407906353 | 3.64098784561 | 0.000271593959377631 | 0.00123086917550453 | 90.7111002493835 | 89.8590365539757 | 116.31304374445 | 217.812082889415 | 210.551813658401 | 241.927536950414 |
| YAR033W | 164.972368541332 | 1.1400791925056 | 0.313242929359224 | 3.63960072406989 | 0.000273061113325117 | 0.00123491121475685 | 92.2229519202066 | 99.7517745232208 | 117.044572321459 | 236.108297852126 | 215.265660232843 | 229.440954398135 |
| YAR035W | 432.078564014973 | 0.18408934018049 | 0.33550713170826 | 0.548689797570578 | 0.583218348930555 | 0.670102795202993 | 414.247357805518 | 403.953467077506 | 394.293903007916 | 327.58937266568 | 527.950816337483 | 524.436467195736 |
| YAR042W | 604.629206405506 | -1.68655399029381 | 0.58478651473575 | -2.8840507566354 | 0.00392595470113774 | 0.0108979087393651 | 1111.96690389036 | 1145.08441994011 | 512.801532483394 | 546.272703886653 | 157.12821914806 | 154.521459084458 |
| YAR050W | 137.105035116692 | -0.0233928563000658 | 0.345408433791335 | -0.0677252030105253 | 0.946004386267313 | 0.961532236041903 | 125.483688678314 | 126.956803938645 | 161.667815519016 | 120.232269754957 | 164.984630105463 | 123.305002703759 |
| YAR053W | 3.42029822642146 | 1.44777862522865 | 1.09608572767422 | 1.32086258280243 | 0.186547187874175 | 0.268461204449092 | 2.26777750623459 | 1.64878966154084 | 1.46305715401824 | 2.61374499467298 | 6.28512876592241 | 6.24329127613972 |
| YAR068W | 4.01507467030804 | 0.245694909872853 | 1.08248018126864 | 0.226974049155251 | 0.820443921345978 | 0.864755949831113 | 5.29148084788071 | 2.47318449231126 | 2.92611430803648 | 0.87124833155766 | 6.28512876592241 | 6.24329127613972 |
| YAR071W | 295.602134208589 | 2.47611896054376 | 0.361913645231963 | 6.84173971654682 | 7.82371473295863e-12 | 3.38828639432281e-09 | 103.56183945138 | 105.522538338614 | 61.4484004687662 | 569.796408838709 | 455.671835529375 | 477.611782624689 |
| YAR073W | 77.7325215399244 | -1.29254028928733 | 0.669497015462786 | -1.93061396755274 | 0.053530809255978 | 0.0958138998356026 | 164.791832119713 | 144.269095384823 | 21.9458573102736 | 38.334926588537 | 42.4246191699763 | 54.6287986662226 |
| YAR075W | 113.0962780879 | -1.13506778341945 | 0.606304723006665 | -1.87210777080978 | 0.0611916951444718 | 0.106998239677002 | 224.509973117224 | 191.259600738737 | 49.7439432366202 | 39.2061749200947 | 91.134367105875 | 82.7236094088513 |
| YBL001C | 2969.15806108988 | 1.24035267439753 | 0.387844843306216 | 3.19806411198879 | 0.00138353517592858 | 0.00461362245821822 | 1697.05350049888 | 1704.02411520246 | 1896.12207160764 | 2417.71412007251 | 4916.54197714281 | 5183.492582015 |
| YBL002W | 14972.3695748102 | 0.917698476509218 | 0.368443230374485 | 2.49074593004863 | 0.0127475239991799 | 0.0291320278760535 | 8822.41042508796 | 9308.24203422881 | 12962.6863846016 | 13344.9106944687 | 21487.2839684973 | 23908.6839419771 |
| YBL003C | 17850.8087909993 | 0.873262778982797 | 0.320455246718769 | 2.72506937528525 | 0.00642879772401392 | 0.0164683601203857 | 13959.6824025447 | 13267.8104064191 | 10593.9968522461 | 17830.0971053275 | 24966.1027404353 | 26487.1632390228 |
| YBL004W | 3328.05161207865 | -1.07809675289659 | 0.384829743874211 | -2.80149019159232 | 0.005086718739699 | 0.013564583305864 | 5068.4827264343 | 5065.08184025346 | 3418.43304036362 | 2985.7680322481 | 1854.11298594711 | 1576.43104722528 |
| YBL005W | 738.516273737808 | -0.08336771542882 | 0.333913471046738 | -0.249668619739966 | 0.802843627607226 | 0.850800224089277 | 676.553622693319 | 789.770247878062 | 811.996720480124 | 519.264005608365 | 837.493408059162 | 796.019637707814 |
| YBL005W-B | 87.3194701016447 | -4.1856195536942 | 1.5123519621247 | -2.76762265565076 | 0.00564667929101354 | 0.0148064391832765 | 234.337008977574 | 223.410999138784 | 39.5025431584925 | 23.5237049520568 | 3.14256438296121 | 0 |
| YBL006C | 1014.83131348243 | 0.314314738746311 | 0.371415773073212 | 0.846261148646358 | 0.397407076288638 | 0.49604281431738 | 1000.08988024945 | 863.141387816629 | 852.230792215626 | 1599.61193673986 | 826.494432718797 | 947.419451154203 |
| YBL006W-A | 12.118359158403 | 1.20127853689006 | 0.785749359229867 | 1.52883171049286 | 0.126306169006426 | 0.19492253256683 | 6.04740668329224 | 2.47318449231126 | 13.1675143861642 | 8.7124833155766 | 25.1405150636897 | 17.1690510093842 |
| YBL007C | 1888.0384572836 | -1.25153713084891 | 0.755084063529975 | -1.65748052607287 | 0.0974223628450919 | 0.156667137637333 | 2560.32080453885 | 2615.80479803454 | 2803.21750709895 | 2724.3935327808 | 259.2615615943 | 365.232539654174 |
| YBL008W | 550.880723054124 | -0.19156231437048 | 0.429415515273179 | -0.446100123440148 | 0.655524923653291 | 0.733677530122904 | 551.069934015005 | 570.48122289313 | 642.282090614008 | 818.102183332642 | 337.82567116833 | 385.523236301628 |
| YBL009W | 1122.02302579996 | -0.107867497464998 | 0.45651092120856 | -0.23628678406955 | 0.813210141413799 | 0.858818188046059 | 1055.2724662345 | 1090.67436110926 | 1347.4756388508 | 1800.87030132968 | 752.644169719209 | 685.201217556334 |
| YBL010C | 98.2108660088278 | -2.3305818005619 | 1.4851811610593 | -1.56922391804352 | 0.11659577293016 | 0.182428760366917 | 179.154422992532 | 136.025147077119 | 177.029915636207 | 92.3523231451119 | 3.14256438296121 | 1.56082281903493 |
| YBL011W | 1305.24821425216 | 0.0444275694103385 | 0.4906827557729 | 0.0905423491811085 | 0.927856242351487 | 0.948432137058046 | 966.829143491346 | 969.488320986013 | 1920.26251464894 | 2136.30090897938 | 931.770339547998 | 906.838057859294 |
| YBL012C | 1.32814860967675 | 1.18526833115016 | 1.75586941425604 | 0.675032164423431 | 0.499655330001711 | 0.593467415199376 | 0.75592583541153 | 1.64878966154084 | 0 | 0.87124833155766 | 1.5712821914806 | 3.12164563806986 |
| YBL013W | 41.4441402310011 | 0.704787986650696 | 0.462934659797252 | 1.52243512499014 | 0.127900089309042 | 0.196675399988117 | 21.1659233915228 | 29.6782139077351 | 43.8917146205473 | 58.3736382143632 | 51.8523123188599 | 43.703038932978 |
| YBL014C | 447.078554012274 | -1.18519275932361 | 0.544438316348235 | -2.17690916259011 | 0.0294873456583426 | 0.0583584837245624 | 648.584366783092 | 624.066886893208 | 592.538147377388 | 522.748998934596 | 164.984630105463 | 129.548293979899 |
| YBL015W | 1880.42448198745 | 1.71844550562055 | 0.379894969258694 | 4.52347528837727 | 6.08324249310942e-06 | 6.27821821404241e-05 | 693.939916907784 | 741.130952862607 | 1193.85463767889 | 2148.49838562119 | 2990.15001038759 | 3514.97298846666 |
| YBL016W | 1395.32501139444 | -0.381656248049249 | 0.729547724188856 | -0.523140893179527 | 0.60087620993811 | 0.685499165987852 | 1391.65946299263 | 1371.79299840198 | 1974.39562934762 | 2873.37699747716 | 325.255413636485 | 435.469566510745 |
| YBL017C | 2613.83306095772 | 0.00770528618548483 | 0.459014393090216 | 0.0167865894871196 | 0.986606868430147 | 0.990297417813302 | 2309.35342718222 | 2427.84277661889 | 3084.85600924746 | 4369.31038276166 | 1887.1099119682 | 1604.52585796791 |
| YBL018C | 946.250132312464 | 1.17082400880495 | 0.407689482414119 | 2.87185237615637 | 0.00408073546112096 | 0.0112259446260654 | 683.356955212023 | 636.432809354764 | 425.749631819308 | 793.707230049028 | 1557.14065175728 | 1581.11351568238 |
| YBL019W | 270.870630140469 | -1.15920614057989 | 0.492551552760559 | -2.35347170074481 | 0.0185990235323358 | 0.039952538875331 | 365.86810433918 | 378.397227323623 | 380.394860044743 | 295.353184398047 | 111.561035595123 | 93.6493691420958 |
| YBL020W | 2427.37339818617 | 0.992336938830158 | 0.346980447963423 | 2.8599217755773 | 0.00423745517019556 | 0.0115926980866863 | 1633.55573032432 | 1771.62449132563 | 1465.98326832628 | 2197.28829218842 | 3624.94801574575 | 3870.84059120663 |
| YBL021C | 262.439432895704 | -0.31193001980521 | 0.367629910157097 | -0.848489231118096 | 0.39616556729484 | 0.494726828268134 | 322.780331720723 | 285.240611446565 | 265.544873454311 | 320.619386013219 | 175.983605445828 | 204.467789293576 |
| YBL022C | 1050.87933816083 | -0.327816202797977 | 0.358592729051231 | -0.914174148665305 | 0.360625345570727 | 0.45744085591402 | 1066.61135376567 | 1098.91830941697 | 1345.28105311977 | 1281.60629572132 | 766.785709442535 | 746.073307498697 |
| YBL023C | 782.819313222289 | -0.0037665611065708 | 0.290479777658521 | -0.0129666895813954 | 0.989654368489619 | 0.992737403905475 | 850.416564837971 | 843.355911878139 | 657.6441907312 | 756.243551792048 | 754.21545191069 | 835.040208183688 |
| YBL024W | 4068.09458315818 | -0.165971650814125 | 0.435172073011011 | -0.381393157115406 | 0.702911537841596 | 0.771824433708419 | 4176.4902406487 | 4616.61105231435 | 4114.1167170993 | 6222.4555839848 | 2842.44948438841 | 2436.44442051353 |
| YBL025W | 214.948646967648 | 0.487030512533741 | 0.371490997005621 | 1.31101565437499 | 0.189852474826723 | 0.27269161235011 | 166.303683790536 | 165.703360984854 | 204.096472985545 | 175.992162974647 | 248.262586253935 | 329.33361481637 |
| YBL026W | 1342.73145910395 | -0.439208435319829 | 0.428366253043679 | -1.02531054255351 | 0.305216682905726 | 0.400406485620874 | 1664.54868957619 | 1740.29748775636 | 1230.43106652934 | 600.290100443228 | 1469.14884903436 | 1351.67256128425 |
| YBL027W | 34273.2986600129 | -1.31703484705904 | 0.613983687346086 | -2.14506488397413 | 0.0319476596175198 | 0.0624599052631535 | 60297.9361132715 | 56804.9258142358 | 29642.2694689866 | 39946.7360019187 | 8605.91256273927 | 10342.0119989254 |
| YBL028C | 374.15445092223 | -2.27118440900756 | 0.417141101380384 | -5.44464307518934 | 5.1909366164146e-08 | 1.7003096659235e-06 | 732.492134513772 | 652.096311139402 | 476.956632209947 | 181.219652963993 | 122.560010935487 | 79.6019637707814 |
| YBL029C-A | 812.88350845099 | 0.711197044430378 | 0.315656572390852 | 2.2530721886879 | 0.0242545976981369 | 0.0498880898038971 | 600.205113316754 | 517.719953723823 | 730.797048432112 | 846.853378274045 | 1126.60933129159 | 1055.11622566761 |
| YBL029W | 153.368559740623 | -0.831512145350689 | 0.415008279346693 | -2.00360375137493 | 0.0451125236953348 | 0.0832926182906985 | 214.682937256874 | 154.161833354068 | 221.653158833764 | 154.210954685706 | 103.70462463772 | 71.7978496756068 |
| YBL030C | 36385.2017989091 | 2.16848655981083 | 0.418919722087893 | 5.1763773474381 | 2.262356610728e-07 | 5.25977493613298e-06 | 12878.7084579062 | 12771.5247182953 | 14074.6098216555 | 30500.6615911705 | 75195.2805554958 | 72890.4256489312 |
| YBL031W | 88.808135155719 | -2.95477765709767 | 0.522209080521246 | -5.65822726435233 | 1.52944523562514e-08 | 6.70042674654822e-07 | 216.194788927697 | 169.000940307936 | 87.7834292410945 | 27.0086982782874 | 12.5702575318448 | 20.2906966474541 |
| YBL032W | 2042.38212848703 | -0.153523638539694 | 0.579204221810582 | -0.265059598598542 | 0.790963550100071 | 0.841135061179616 | 1748.45645730687 | 1803.77588972568 | 2901.97386499518 | 3709.77539577251 | 758.929298485132 | 1331.3818646368 |
| YBL033C | 788.932989823159 | -0.5052323129467 | 0.746345016613005 | -0.676942033108896 | NA | NA | 980.435808528754 | 863.141387816629 | 934.893521417657 | 1571.73199013002 | 149.271808190657 | 234.12342285524 |
| YBL034C | 358.847838817033 | -0.481121622151858 | 0.384008542669782 | -1.25289301849096 | 0.21024464300448 | 0.295950929169148 | 480.768831321733 | 440.226839631404 | 332.113973962141 | 195.159626268916 | 367.680032806461 | 337.137728911545 |
| YBL035C | 541.541122345809 | -0.385381200926976 | 0.440968039653015 | -0.873943611038615 | 0.382148963125885 | 0.480390264011458 | 615.323630024985 | 577.900776370064 | 648.86584780709 | 766.69853177074 | 312.68515610464 | 327.772791997335 |
| YBL036C | 1470.81543205456 | -0.403183832827631 | 0.338372026437564 | -1.19154008406787 | 0.233441630449353 | 0.322109821619483 | 1895.1060693767 | 1879.62021415656 | 1249.45080953158 | 1021.10304458558 | 1374.87191754553 | 1404.74053713144 |
| YBL037W | 680.309270424434 | -0.29983801240914 | 0.361431124679458 | -0.829585478215404 | 0.40677319001362 | 0.505132923966007 | 711.326211122249 | 737.833373539525 | 804.681434710033 | 849.467123268718 | 455.671835529375 | 522.875644376702 |
| YBL038W | 450.847922123527 | -0.755691809320663 | 0.329534931249342 | -2.29320699464443 | 0.0218360906481974 | 0.0457463968036407 | 596.425484139697 | 534.207850339232 | 569.860761490105 | 433.010420784157 | 282.830794466509 | 288.752221521462 |
| YBL039C | 8235.81189693695 | 0.678689961478112 | 0.306404952340721 | 2.21500976499692 | 0.0267593942757602 | 0.0539713432934218 | 6748.14993271872 | 6891.94078524071 | 5361.37294089985 | 11821.0973625743 | 8409.50228880419 | 10182.8080713839 |
| YBL039W-B | 221.133871476207 | 0.72785282636006 | 0.407888259213673 | 1.78444171882568 | 0.0743519171460206 | 0.125841352541491 | 143.625908728191 | 150.864254030987 | 204.096472985545 | 167.279679659071 | 340.968235551291 | 319.968677902161 |
| YBL040C | 4264.48680683768 | 1.33266055379001 | 0.394437006221967 | 3.37863976444457 | 0.000728453935129723 | 0.00275568463802623 | 2277.60454209494 | 2539.13607877289 | 2454.2783758656 | 3444.91590297899 | 7188.61602602376 | 7682.36991528993 |
| YBL041W | 2820.91270228583 | 0.00907830847719986 | 0.321776113934967 | 0.0282131211238216 | 0.977492172247065 | 0.983908969876696 | 2642.71672059871 | 2447.62825255738 | 3346.74323981673 | 3478.89458790973 | 2466.91304062455 | 2542.5803722079 |
| YBL042C | 12232.2180367038 | 1.60765578963249 | 0.381084323843493 | 4.21863532306498 | 2.45785437473934e-05 | 0.000185129616062238 | 5692.12154064882 | 6087.33143040878 | 6352.59416274721 | 10873.1791778396 | 21782.6850204956 | 22605.3968880829 |
| YBL043W | 207.901560808977 | -1.97109983413201 | 0.354372963520642 | -5.56221844507953 | 2.66366570321026e-08 | 1.05478049259596e-06 | 352.261439301773 | 293.484559754269 | 349.67065981036 | 106.292296450034 | 81.7066739569914 | 63.9937355804321 |
| YBL044W | 7.66583788513563 | 1.95822984668943 | 0.853088942706414 | 2.29545800989633 | 0.0217068901627592 | 0.0455498118762363 | 4.53555501246918 | 3.29757932308168 | 1.46305715401824 | 6.96998665246128 | 10.9989753403642 | 18.7298738284192 |
| YBL045C | 5880.31357674248 | 0.388968840767592 | 0.36490791730451 | 1.06593697292406 | 0.28645213405757 | 0.379422407102171 | 4240.74393665868 | 4500.37138117572 | 6536.9393641535 | 8809.1918803795 | 5689.61281535127 | 5505.0220827362 |
| YBL046W | 306.356892865814 | -1.80268216753921 | 0.391036700802007 | -4.610007612692 | 4.02654226265955e-06 | 4.58954551708451e-05 | 544.266601496301 | 544.100588308477 | 341.62384546326 | 173.378417979974 | 97.4194958717974 | 137.352408075074 |
| YBL047C | 1583.88288276177 | -0.0262949656759315 | 0.272159176908001 | -0.0966161272776779 | 0.923031248698167 | 0.94564448641683 | 1586.6883285288 | 1772.4488861564 | 1435.9905966689 | 1590.02820509273 | 1583.85244901245 | 1534.28883111134 |
| YBL048W | 135.578543957898 | 0.235075787992963 | 0.493898433812803 | 0.475959776139037 | 0.634103034698299 | 0.714388112094458 | 164.035906284302 | 118.71285563094 | 89.9780149721219 | 69.6998665246128 | 169.698476679905 | 201.346143655506 |
| YBL049W | 248.171063006995 | 0.0288233427210076 | 0.450028093585065 | 0.0640478741924571 | 0.948932106938394 | 0.963226796508801 | 293.299224139673 | 247.318449231126 | 195.318130061435 | 128.073504738976 | 337.82567116833 | 287.191398702427 |
| YBL050W | 1714.33366527278 | 0.591785562846077 | 0.294853493475093 | 2.00704952100581 | 0.044744390341582 | 0.0827790502153944 | 1484.63834074824 | 1477.31553674059 | 1140.45305155722 | 1865.34267786495 | 2028.52530920146 | 2289.72707552424 |
| YBL051C | 492.382606528261 | -2.33055071520286 | 0.504728939003674 | -4.61743033756559 | 3.88521042145988e-06 | 4.48400629286767e-05 | 991.018770224515 | 897.765970708987 | 577.907575837206 | 284.026956087797 | 100.562060254759 | 103.014306056305 |
| YBL052C | 45.6255171840873 | -2.60922681955236 | 0.664517662748773 | -3.92649731650368 | 8.61918534917242e-05 | 0.000499168647919698 | 84.6636935660913 | 110.468907323236 | 41.6971288895199 | 24.3949532836145 | 6.28512876592241 | 6.24329127613972 |
| YBL053W | 16.5899172893937 | -0.724996034662988 | 0.590816470325901 | -1.22710870647034 | 0.219781735936488 | 0.306787852986022 | 21.1659233915228 | 18.1366862769492 | 21.9458573102736 | 6.96998665246128 | 15.712821914806 | 15.6082281903493 |
| YBL054W | 1113.9371552656 | -2.57800006650112 | 0.697887655292092 | -3.69400439591115 | 0.000220749982539171 | 0.00103844403765687 | 2164.97159261862 | 2216.79769994166 | 1345.28105311977 | 730.977350176876 | 125.702575318448 | 99.8926604182355 |
| YBL055C | 1998.598086736 | 0.758837234892422 | 0.27772562000928 | 2.7323270891143 | 0.00628886721548399 | 0.0161742431580339 | 1499.00093162106 | 1630.65297526389 | 1324.79825296352 | 2524.00641652254 | 2309.78482147649 | 2703.3451225685 |
| YBL056W | 5620.54837802035 | 1.4799965543541 | 0.332231172669516 | 4.45471911158172 | 8.40031001283292e-06 | 8.00266220157456e-05 | 2666.90634733188 | 2841.68898166564 | 3389.90342586027 | 6013.35598441097 | 9204.57107769338 | 9606.86445115999 |
| YBL057C | 2234.77470036282 | 0.904770276860883 | 0.295765215793627 | 3.05908277426442 | 0.0022201579789839 | 0.00678586491915345 | 1667.57239291783 | 1619.1114476331 | 1381.12595339322 | 2457.79154332416 | 2924.1561583454 | 3358.89070656317 |
| YBL058W | 1156.82020307251 | -1.15951612108127 | 0.761423638002204 | -1.52282653599192 | NA | NA | 1548.89203675822 | 1481.43751089444 | 1765.90998490002 | 1756.43663642024 | 174.412323254347 | 213.832726207785 |
| YBL059C-A | 244.064421651579 | -0.538163706411002 | 0.305101427590346 | -1.76388458966368 | 0.0777514118768841 | 0.130565604299122 | 316.732925037431 | 293.484559754269 | 256.766530530201 | 191.674632942685 | 221.550788998765 | 184.177092646122 |
| YBL059W | 115.946496804806 | -0.992887330412322 | 0.415161761475927 | -2.39156739022048 | 0.0167766037700548 | 0.03668635934776 | 150.429241246894 | 160.756992000232 | 153.621001171915 | 115.004779765611 | 54.9948767018211 | 60.8720899423623 |
| YBL060W | 188.846317113931 | -1.93874404541993 | 0.460233944471063 | -4.21251858692885 | 2.52538926577851e-05 | 0.000189551362140019 | 318.244776708254 | 314.9188253543 | 267.739459185338 | 127.202256407418 | 59.708723276263 | 45.263861752013 |
| YBL061C | 1019.85606519677 | -1.8595977383173 | 0.681889801466005 | -2.72712355327697 | 0.00638891114687482 | 0.0163857378677315 | 1764.33089985051 | 1726.28277563326 | 1308.70462426932 | 1001.93558129131 | 155.55693695658 | 162.325573179633 |
| YBL062W | 3.65595258913379 | -2.11411489447413 | 1.29056482836868 | -1.63813149715729 | 0.101394265788278 | 0.162109997933592 | 7.5592583541153 | 5.77076381539294 | 5.12070003906385 | 3.48499332623064 | 0 | 0 |
| YBL063W | 80.7062769014903 | -2.56438665952125 | 0.56915988849408 | -4.5055646249181 | 6.61966884576273e-06 | 6.73470258557851e-05 | 188.225533017471 | 145.093490215594 | 82.6627292020307 | 40.0774232516523 | 12.5702575318448 | 15.6082281903493 |
| YBL064C | 1045.2884932604 | 1.01757648718751 | 0.414106806450238 | 2.45728027488915 | 0.0139993419516437 | 0.0316114173101631 | 509.494013067371 | 509.476005416119 | 1054.13267947014 | 1127.39534103561 | 1571.2821914806 | 1499.95072909257 |
| YBL065W | 16.1582100721944 | 1.49079534645447 | 0.761458560775199 | 1.9578154652786 | 0.0502516700449503 | 0.0909813761848412 | 7.5592583541153 | 5.77076381539294 | 11.7044572321459 | 6.09873832090362 | 39.2820547870151 | 26.5339879235938 |
| YBL066C | 500.803502624055 | -0.921600713870847 | 0.502636238907528 | -1.83353415956225 | 0.0667231734022758 | 0.114631269907081 | 579.795115760643 | 586.969119508539 | 801.755320401997 | 615.101322079708 | 201.124120509517 | 220.076017483925 |
| YBL067C | 896.630029294922 | -0.492133920642905 | 0.278800792896591 | -1.76518120888358 | 0.0775333156932013 | 0.130335304897994 | 1087.02135132178 | 1065.94251618615 | 991.952750424368 | 809.389700017066 | 769.928273825496 | 655.545583994671 |
| YBL068W | 4446.30124958628 | 1.36609242633011 | 0.342608849575334 | 3.98732381846934 | 6.68227925609128e-05 | 0.00041023716310036 | 2449.19970673336 | 2657.84893440383 | 2348.93826077629 | 4370.18163109322 | 7095.91037672641 | 7755.72858778457 |
| YBL069W | 1680.53253688897 | 0.273621218756258 | 0.291790598311621 | 0.937731442820655 | 0.348382440850463 | 0.444872347462239 | 1604.07462274327 | 1497.10101267908 | 1464.52021117226 | 2111.03470736421 | 1519.42987916174 | 1887.03478821323 |
| YBL070C | 8.57549658035583 | 2.14615003443095 | 0.829457282655281 | 2.58741478230275 | 0.00966991113601271 | 0.0231761174975519 | 3.02370334164612 | 4.1219741538521 | 2.19458573102736 | 6.09873832090362 | 17.2841041062866 | 18.7298738284192 |
| YBL071C | 5.61664526381725 | 0.513587632381711 | 0.970871277414819 | 0.528996628419437 | 0.596807788759673 | 0.681582223729792 | 8.31518418952682 | 2.47318449231126 | 2.92611430803648 | 4.3562416577883 | 3.14256438296121 | 12.4865825522794 |
| YBL071C-B | 4.97762532170514 | -0.636786931982577 | 1.01011830201487 | -0.630408270706895 | 0.528427500965014 | 0.620658965204211 | 4.53555501246918 | 5.77076381539294 | 8.04681434710033 | 5.22748998934596 | 6.28512876592241 | 0 |
| YBL071W-A | 443.087808842279 | 0.848440615975772 | 0.391710965509712 | 2.16598637945134 | 0.0303122277754093 | 0.0597340106712472 | 286.49589162097 | 252.264818215748 | 409.656003125108 | 380.735520890697 | 636.369287549644 | 693.005331651509 |
| YBL072C | 21523.1064469733 | 0.234631067827481 | 0.326673870180527 | 0.718242532522785 | 0.472607757063536 | 0.567940652265193 | 22699.6969115728 | 22439.20289874 | 14191.6543939769 | 24041.2264610021 | 21399.2921657743 | 24367.5658507733 |
| YBL073W | 1.86927040250698 | 3.64345943099073 | 1.80910197357806 | 2.01396023231607 | 0.0440137088694946 | 0.0816383309676109 | 0.75592583541153 | 0 | 0 | 2.61374499467298 | 6.28512876592241 | 1.56082281903493 |
| YBL074C | 79.9649247928504 | -0.805102017997461 | 0.432498135684045 | -1.86151558023273 | 0.0626714025061616 | 0.109141111990179 | 101.294061945145 | 115.415276307859 | 89.9780149721219 | 85.3823364926506 | 47.1384657444181 | 40.5813932949082 |
| YBL075C | 487.96318712586 | -0.33661484369842 | 0.317229081232713 | -1.0611096636865 | 0.288640066634379 | 0.382084692523207 | 533.68363980054 | 497.110082954563 | 604.242604609534 | 527.105240592384 | 359.823621849058 | 405.813932949082 |
| YBL076C | 19837.9162828455 | 0.413515621842726 | 0.343347025798778 | 1.20436640125455 | 0.228447992289033 | 0.316456242276054 | 17260.8105257869 | 18069.0859008261 | 15713.9653627329 | 30850.0321721252 | 18065.0313554525 | 19068.5723801497 |
| YBL077W | 2.30975027290025 | 0.308784966514047 | 1.72090651883022 | 0.179431574658653 | 0.857598840688876 | 0.895358210659676 | 5.29148084788071 | 0 | 0.731528577009121 | 0 | 4.71384657444181 | 3.12164563806986 |
| YBL078C | 155.16328952212 | 0.251394902134002 | 0.489475081445083 | 0.51360102212314 | 0.607530964817791 | 0.692232734151906 | 118.68035615961 | 77.4931140924194 | 228.236916026846 | 124.588511412745 | 171.269758871386 | 210.711080569716 |
| YBL079W | 2182.09554304228 | -0.551040213126236 | 0.299802956497272 | -1.83800793549296 | 0.0660612359869868 | 0.113752502608608 | 2557.2971011972 | 2780.68376418862 | 2444.76850436448 | 2111.03470736421 | 1740.98066816051 | 1457.80851297862 |
| YBL080C | 156.482769120383 | -0.859063869393261 | 0.777478529220411 | -1.10493581122382 | 0.269187403698696 | 0.36040891472341 | 162.524054613479 | 189.610811077197 | 254.571944799174 | 272.700727777547 | 26.7117972551703 | 32.7772791997335 |
| YBL081W | 851.184464307465 | -0.881672421061423 | 0.317359737479113 | -2.77814831857633 | 0.00546696573919476 | 0.014418826159375 | 1144.47171481306 | 1208.56282190944 | 956.107850150921 | 471.345347372694 | 691.364164251466 | 635.254887347216 |
| YBL082C | 2511.34054906845 | 1.77528251036213 | 0.413641336296146 | 4.29184018758493 | 1.77198467236546e-05 | 0.000143181697491011 | 1169.41726738164 | 1133.54289230933 | 1103.14509412975 | 2037.84984751337 | 4932.25479905761 | 4691.833394019 |
| YBL083C | 57.6206136991245 | 1.45088503222959 | 0.465416252008863 | 3.11739228264414 | 0.00182458610021076 | 0.00577695894068696 | 30.2370334164612 | 28.8538190769647 | 32.9187859654104 | 47.0474099041136 | 95.8482136803168 | 110.81842015148 |
| YBL084C | 368.124853380259 | -0.443495991449196 | 0.301479964227588 | -1.47106290325283 | 0.141274106219977 | 0.213920819199777 | 409.711802793049 | 377.572832492852 | 485.734975134056 | 326.718124334122 | 278.116947892067 | 330.894437635405 |
| YBL085W | 429.505212492351 | -3.19626715781341 | 0.631920212248821 | -5.05802330081961 | 4.23624682919678e-07 | 8.09537969733747e-06 | 1053.76061456367 | 925.795394955181 | 346.013016925314 | 163.79468633284 | 39.2820547870151 | 48.3855073900828 |
| YBL086C | 93.6839931791218 | -3.91931519056552 | 0.822885552346249 | -4.76289221434328 | 1.90837788047608e-06 | 2.55508389818419e-05 | 248.699599850393 | 249.791633723437 | 29.992671657374 | 22.6524566204991 | 6.28512876592241 | 4.68246845710479 |
| YBL087C | 64926.675169643 | 1.24924161012812 | 0.442175318079601 | 2.82521786958545 | 0.00472484770368438 | 0.0127634308774024 | 43710.6555068363 | 43398.6170762472 | 28241.3922440141 | 46902.782681075 | 112912.338279796 | 114394.265229889 |
| YBL088C | 852.189456803002 | -0.360994562638215 | 0.304374162504634 | -1.18602236033329 | 0.235613469250883 | 0.324289536647935 | 936.592110074885 | 935.688132924426 | 1001.46262192549 | 619.457563737496 | 908.201106675789 | 711.735205479928 |
| YBL089W | 1335.98334874572 | 1.2621822217923 | 0.395570365441494 | 3.19079064576433 | 0.001418840509265 | 0.00470037035048803 | 795.98990468834 | 805.4337496627 | 756.400548627431 | 1062.05171616879 | 2254.78994477467 | 2341.2342285524 |
| YBL090W | 448.186269484762 | -0.750517278944206 | 0.385412419672941 | -1.94730953294419 | 0.0514976365115302 | 0.0927937266743857 | 620.615110872866 | 524.315112369987 | 543.525732717777 | 487.027817340732 | 252.976432828377 | 260.657410778833 |
| YBL091C | 3233.6063750468 | 0.106594785407895 | 0.347747901114304 | 0.30652891093326 | 0.759201970800366 | 0.815964734972356 | 2710.75004578574 | 2780.68376418862 | 3852.22948653003 | 4342.30168448338 | 2804.73871179288 | 2910.93455750014 |
| YBL091C-A | 159.277232468925 | -0.116596317028113 | 0.394380720793157 | -0.295644058851611 | 0.767501922769466 | 0.822141114876141 | 132.287021197018 | 131.903172923267 | 233.35761606591 | 177.734659637763 | 149.271808190657 | 131.109116798934 |
| YBL092W | 65158.3171605662 | -0.788639119135989 | 0.429246045946225 | -1.8372658911686 | 0.0661706526254751 | 0.113849586670601 | 93531.459541304 | 94122.8066187203 | 59958.2767573986 | 73364.3370071443 | 33496.5937579835 | 36476.4292808463 |
| YBL093C | 763.640976960762 | -1.88508864523466 | 0.648513825464537 | -2.90678250981674 | 0.00365166992056 | 0.0102603640001773 | 1344.0361353617 | 1315.73414990959 | 948.061035803821 | 710.93863855105 | 128.84513970141 | 134.230762437004 |
| YBL094C | 26.3359005752016 | 0.170317764792966 | 0.47849267079228 | 0.355946444301762 | 0.721880673043121 | 0.786617856920084 | 26.4574042394035 | 23.9074500923422 | 23.4089144642919 | 20.0387116258262 | 31.4256438296121 | 32.7772791997335 |
| YBL095W | 1060.93808921074 | 1.76932583205023 | 0.407721917100197 | 4.33954064729715 | 1.42780856358172e-05 | 0.000121628136897702 | 503.446606384079 | 497.934477785333 | 441.843260513509 | 893.029539846601 | 2009.66992290369 | 2019.7047278312 |
| YBL096C | 24.5762851433316 | 0.815442232449974 | 0.588956005704514 | 1.38455542443197 | 0.1661884495719 | 0.243849080711559 | 12.0948133665845 | 12.3659224615563 | 28.5296145033557 | 20.9099599573838 | 29.8543616381315 | 43.703038932978 |
| YBL097W | 68.4771909358233 | -1.82640170599744 | 0.492430625708824 | -3.70895230849716 | 0.000208118619695725 | 0.000995014039228264 | 125.483688678314 | 138.49833156943 | 56.3277004297023 | 27.8799466098451 | 36.1394904040539 | 26.5339879235938 |
| YBL098W | 1298.08411918459 | 1.27996464962082 | 0.33205851790902 | 3.85463579636743 | 0.000115902037659742 | 0.000629350018995562 | 631.198072568627 | 720.521082093347 | 919.531421300465 | 1490.70589529516 | 1819.54477773454 | 2207.00346611539 |
| YBL099W | 27596.514680716 | 1.62120816186687 | 0.35808380021119 | 4.52745463746397 | 5.96984109132387e-06 | 6.18099302702987e-05 | 13506.1269012978 | 13698.1445080813 | 13414.7710451933 | 26472.8805543795 | 49595.9510918938 | 48891.2139834502 |
| YBL100W-B | 9.42457243685753 | -4.45581994288856 | 1.3690341213992 | -3.25471796008602 | 0.00113505025798986 | 0.00395373897440984 | 26.4574042394035 | 24.7318449231126 | 2.92611430803648 | 0.87124833155766 | 0 | 1.56082281903493 |
| YBL100W-C | 3.22304438384122 | -0.384556576401412 | 1.13712062824545 | -0.338184504659611 | 0.735224152154551 | 0.797514492146759 | 3.77962917705765 | 2.47318449231126 | 4.38917146205473 | 0.87124833155766 | 3.14256438296121 | 4.68246845710479 |
| YBL101C | 407.220352007565 | -0.878898188255663 | 0.464123879678706 | -1.89367155351732 | 0.0582686193949349 | 0.102792624927619 | 420.29476448881 | 469.905053539139 | 694.220619581656 | 462.632864057117 | 207.40924927544 | 188.859561103227 |
| YBL102W | 2732.18067740557 | 1.22530019630204 | 0.400729420532717 | 3.05767466404929 | 0.00223061603878587 | 0.00681303864937556 | 1635.82350783055 | 1734.52672394096 | 1539.8676546042 | 2115.390949022 | 4836.4065853773 | 4531.0686436584 |
| YBL103C | 376.26256330571 | -2.59535644022971 | 0.563437525172496 | -4.60628964930078 | 4.09917312991233e-06 | 4.63134648362025e-05 | 629.686220897804 | 724.643056247199 | 585.222861607297 | 209.970847905396 | 53.4235945103405 | 54.6287986662226 |
| YBL104C | 507.5531603114 | -1.15490613143296 | 0.623110952230406 | -1.85345182474968 | 0.0638176250854345 | 0.110628669057927 | 690.160287730726 | 740.306558031837 | 673.006290848391 | 670.861215299398 | 144.557961616216 | 126.426648341829 |
| YBL105C | 1111.16059964506 | -0.542117276029619 | 0.305297744896745 | -1.7757002306485 | 0.0757823393692606 | 0.127792161701503 | 1452.88945566096 | 1394.87605366355 | 1103.87662270676 | 768.441028433856 | 1018.19086007943 | 928.689577325783 |
| YBL106C | 537.139150030745 | 0.439646899526359 | 0.351232887238621 | 1.25172475442959 | 0.210670176695152 | 0.296420349118806 | 439.192910374099 | 413.846205046751 | 515.72764679143 | 814.617190006412 | 460.385682103817 | 579.065265861959 |
| YBL107C | 288.092761129856 | -1.78507167342454 | 0.48458380219582 | -3.68372129925877 | 0.000229853549999141 | 0.00107342774618888 | 448.264020399037 | 478.973396677614 | 414.776703164172 | 220.425827884088 | 100.562060254759 | 65.554558399467 |
| YBL109W | 7.84699616048774 | -1.574997493577 | 0.810498143741783 | -1.94324626865373 | 0.0519864101371085 | 0.0935435823646211 | 8.31518418952682 | 14.8391069538676 | 12.4359858091551 | 5.22748998934596 | 3.14256438296121 | 3.12164563806986 |
| YBL113C | 237.219115954356 | -0.418356585857171 | 0.354705508229268 | -1.1794476718043 | 0.238219961917052 | 0.32732129500027 | 288.763669127204 | 309.972456369678 | 216.5324587947 | 252.662016151721 | 161.842065722502 | 193.542029560331 |
| YBL113W-A | 3.27836925092087 | 1.35929701717242 | 1.265127911792 | 1.07443445402057 | 0.282628007340972 | 0.375206012631594 | 0 | 2.47318449231126 | 2.92611430803648 | 1.74249666311532 | 6.28512876592241 | 6.24329127613972 |
| YBR001C | 774.809870454509 | 0.36560822569919 | 0.288388359137005 | 1.26776346588074 | 0.204882432612724 | 0.289987443082625 | 715.105840299307 | 638.905993847075 | 676.663933733437 | 742.303578487126 | 903.487260101347 | 972.392616258761 |
| YBR002C | 932.593416549562 | 0.725215323049017 | 0.360148175427012 | 2.01365818996351 | 0.0440454325092748 | 0.0816736496860725 | 767.264722942702 | 713.925923447183 | 626.919990496817 | 771.054773428529 | 1257.02575318448 | 1459.36933579766 |
| YBR003W | 1375.51195004083 | 0.917173100464932 | 0.368152319170464 | 2.49128703720119 | 0.0127281245538749 | 0.0291083530280378 | 766.508797107291 | 749.374901170311 | 1341.62341023473 | 2064.85854579165 | 1627.84835037391 | 1702.85769556711 |
| YBR004C | 3974.48915549873 | 0.860045206197496 | 0.400436503082334 | 2.14776924575395 | 0.0317320913036741 | 0.0621138808497451 | 2656.32338563611 | 2802.11802978866 | 3011.70315154655 | 2880.34698412962 | 6874.35958772764 | 5622.08379416382 |
| YBR005W | 5615.69884519744 | 1.58197974031794 | 0.437022542874819 | 3.61990420428056 | 0.0002947121038614 | 0.00131985114663937 | 2270.80120957623 | 2336.33495040337 | 3828.82057206574 | 4757.01589030482 | 11226.8112581289 | 9274.40919070555 |
| YBR006W | 2044.62262838522 | 0.912885120997759 | 0.341500796459971 | 2.67315663817131 | 0.00751411554775623 | 0.0187488973760365 | 1443.81834563602 | 1391.57847434047 | 1419.1654393977 | 1826.13650294485 | 3272.9808048541 | 2914.05620313821 |
| YBR007C | 320.720089129097 | -1.37967936109638 | 0.417585773354887 | -3.30394244519401 | 0.000953354023541328 | 0.00343955177120793 | 577.527338254409 | 545.749377970018 | 267.739459185338 | 216.940834557857 | 161.842065722502 | 154.521459084458 |
| YBR008C | 1191.81807115516 | 1.45214869212653 | 0.405774069082307 | 3.57871239877574 | 0.000345291187681616 | 0.00149641672185034 | 573.747709077351 | 558.939695262344 | 780.540991668732 | 1010.64806460689 | 2158.94173109435 | 2068.09023522128 |
| YBR009C | 10686.627174631 | 0.650045705792647 | 0.441189033872713 | 1.47339497558815 | 0.140644564294537 | 0.21316803813999 | 7934.95349431482 | 7192.84489847191 | 9828.08643211754 | 6385.37902198609 | 17065.6958816708 | 15712.8033192246 |
| YBR010W | 18928.2913864669 | 0.889632625322458 | 0.341601943800883 | 2.60429614487504 | 0.00920631699329484 | 0.0222138184476653 | 14250.7138491782 | 13313.1521221115 | 12246.5199077097 | 16909.1876188711 | 26779.3623894039 | 30070.812431527 |
| YBR011C | 27598.1663788306 | 0.763227917335991 | 0.442291029499622 | 1.72562377807991 | 0.0844151187198164 | 0.139715590993477 | 16222.9243537668 | 17111.9635023016 | 28057.0470426078 | 53277.7067230824 | 24246.4554967372 | 26672.9011544879 |
| YBR012W-B | 1.33208809477558 | -2.36616148468851 | 1.92842381637625 | -1.22699246119809 | 0.219825424416622 | 0.306787852986022 | 2.26777750623459 | 4.1219741538521 | 0.731528577009121 | 0.87124833155766 | 0 | 0 |
| YBR014C | 749.777922737865 | -1.0936051105234 | 0.330814289020675 | -3.30579768413526 | 0.000947064434242948 | 0.00341877519984562 | 1077.19431546143 | 910.956288001314 | 1076.81006535743 | 617.715067074381 | 421.103627316802 | 394.888173215837 |
| YBR015C | 6738.11840132593 | 0.674545543879769 | 0.308000804372893 | 2.19007721506826 | 0.0285186373694278 | 0.0568782981291777 | 4599.05278264375 | 4954.61293293022 | 6019.74866020806 | 9486.1518339998 | 6758.08470555808 | 8611.05949261571 |
| YBR016W | 645.88348717411 | -0.375666530973937 | 0.536294827784718 | -0.70048509049716 | 0.48362441305479 | 0.578266101016125 | 517.809197256898 | 456.714736246812 | 1215.06896641215 | 910.454506477754 | 397.534394444593 | 377.719122206453 |
| YBR017C | 5461.49981853269 | 1.33173872094084 | 0.319558982834003 | 4.16742696177818 | 3.08057170416211e-05 | 0.000221169250555229 | 2964.74112648402 | 2972.76775975813 | 3378.93049720513 | 5847.81880141501 | 8239.80381212429 | 9364.93691420958 |
| YBR018C | 91.4394705786937 | 1.44087640435088 | 0.495005322510211 | 2.91083012409662 | 0.00360469942898887 | 0.0101594154585069 | 30.2370334164612 | 38.7465570462097 | 78.273557739976 | 95.8373164713426 | 177.554887637308 | 127.987471160864 |
| YBR019C | 50.7486443025428 | 1.17893021301649 | 0.536540181039082 | 2.19728224404989 | 0.0280002949598854 | 0.0560179868100844 | 24.9455525685805 | 23.0830552615717 | 44.6232431975564 | 34.8499332623064 | 92.7056492973556 | 84.2844322278862 |
| YBR020W | 174.396451136049 | 1.31856962738732 | 0.38536025765887 | 3.42165441604658 | 0.000622413701832303 | 0.00242342457061671 | 89.1992485785605 | 100.576169353991 | 108.997757974359 | 160.309693006609 | 298.543616381315 | 288.752221521462 |
| YBR021W | 1879.1747269553 | 0.925632361511293 | 0.416641077283602 | 2.22165410944641 | 0.0263066900988239 | 0.0532249714848965 | 1556.45129511234 | 1409.71516061742 | 921.726007031492 | 1467.1821903431 | 2779.59819672919 | 3140.37551189828 |
| YBR022W | 409.146071619361 | -0.0427569540296567 | 0.276880318999817 | -0.154423955390216 | 0.877275466533964 | 0.909767150479667 | 405.17624778058 | 431.158496492929 | 408.924474548099 | 378.121775896024 | 405.390805401996 | 426.104629596536 |
| YBR023C | 5463.90700590756 | 0.482682269870875 | 0.336908782970412 | 1.43267939059121 | 0.151949481867059 | 0.226517283153671 | 4395.70873291804 | 5074.15018339193 | 4204.09473207142 | 4484.31516252727 | 7073.91242604568 | 7551.26079849099 |
| YBR024W | 323.755379501482 | 0.374005687992969 | 0.484715727599164 | 0.77159800414451 | 0.4403525567437 | 0.537091201872278 | 213.171085586051 | 230.830552615717 | 403.072245932026 | 586.350127138305 | 276.545665700586 | 232.562600036205 |
| YBR025C | 27492.4783443502 | 0.956794552658358 | 0.332722573575466 | 2.87565265673609 | 0.00403193125832445 | 0.0111297202330088 | 18631.304065388 | 19581.8504152898 | 17874.1692506409 | 25328.9314950443 | 40837.6241565809 | 42700.9906831576 |
| YBR026C | 1140.05931970012 | -0.19819888062846 | 0.613121107791259 | -0.32326220400805 | 0.746496675446575 | 0.806918200994191 | 1056.02839206991 | 1093.97194043235 | 1506.21734006178 | 2220.81199714047 | 513.809276614157 | 449.51697188206 |
| YBR027C | 41.3277976894822 | 1.05790241975628 | 0.482810473284453 | 2.19113395067759 | 0.0284421005261978 | 0.0567432240981146 | 24.1896267331689 | 34.6245828923576 | 21.2143287332645 | 34.8499332623064 | 62.8512876592241 | 70.2370268565718 |
| YBR028C | 431.111007411494 | -0.00218814609942446 | 0.325588693429683 | -0.00672058380275734 | 0.994637790309376 | 0.995871902859125 | 374.183288528707 | 408.899836062128 | 512.070003906385 | 514.907763950577 | 366.108750614981 | 410.496401406187 |
| YBR029C | 4426.64368092236 | 1.47569278092384 | 0.350647815510602 | 4.20847561469901 | 2.57099268557924e-05 | 0.000192301891929504 | 2177.0664059852 | 2260.49062597249 | 2585.95351972724 | 4341.43043615182 | 7550.0109300643 | 7644.91016763309 |
| YBR030W | 504.203010163988 | -0.500906229988924 | 0.768890158803213 | -0.651466564182055 | NA | NA | 548.046230673359 | 577.076381539294 | 648.86584780709 | 1028.9442795696 | 97.4194958717974 | 124.865825522794 |
| YBR031W | 78760.2186036854 | 1.17445711058531 | 0.409209837509383 | 2.87006079260835 | 0.00410392888842562 | 0.0112801118401455 | 53485.5324845428 | 53484.2634358925 | 38117.0280336373 | 61665.214410988 | 130542.124468209 | 135267.148788843 |
| YBR032W | 15.6305706137926 | 1.56907503070193 | 0.641389193479982 | 2.4463696093609 | 0.0144302983019623 | 0.0324223416442399 | 6.80333251870377 | 11.5415276307859 | 5.12070003906385 | 13.9399733049226 | 28.2830794466509 | 28.0948107426287 |
| YBR033W | 84.8058242152055 | -1.19205692609991 | 0.362810823201364 | -3.28561567039666 | 0.00101759751506165 | 0.00361862396300223 | 120.192207830433 | 127.781198769415 | 106.803172243332 | 60.1161348774785 | 43.9959013614569 | 49.9463302091178 |
| YBR034C | 7863.26043146105 | 1.15503842136486 | 0.295341799216707 | 3.91085320272378 | 9.19706598527915e-05 | 0.000526480932846202 | 4581.66648842928 | 4996.65706929951 | 5041.69495274686 | 8666.30715400404 | 11982.5979922311 | 11910.6389320556 |
| YBR035C | 2540.28859951055 | 0.235201108583729 | 0.352379377030709 | 0.667465589404321 | 0.504474782504765 | 0.598088659670598 | 2112.05678413981 | 2121.99229440306 | 2768.10413540251 | 3660.11424087373 | 2111.80326534993 | 2467.66087689422 |
| YBR036C | 9350.71954087593 | 1.4896249488829 | 0.383779314002075 | 3.88146232622336 | 0.000103830238539989 | 0.000578431432696824 | 4923.34496603529 | 5145.87253366896 | 4662.76314985614 | 8066.88830189237 | 17208.6825610956 | 16096.7657327072 |
| YBR037C | 974.328355203726 | 2.09518279349936 | 0.433675870422372 | 4.83121828165997 | 1.3570012865647e-06 | 1.96384006415206e-05 | 286.49589162097 | 313.270035692759 | 508.412361021339 | 920.038238124888 | 1872.96837224488 | 1944.78523251752 |
| YBR038W | 2732.27505849598 | 0.138444971049213 | 0.322751159770121 | 0.428952667893804 | 0.667957670492019 | 0.743843575647346 | 2929.21261221968 | 2889.50388185032 | 1984.63702942575 | 2487.41398659712 | 2823.59409809064 | 3279.28874279239 |
| YBR039W | 3530.5733434786 | -0.869847399297032 | 0.499814598250757 | -1.74034012280015 | 0.0817993127369343 | 0.136085655909547 | 4608.12389266868 | 4588.58162806815 | 4496.70616287507 | 4481.7014175326 | 1734.69553939459 | 1273.6314203325 |
| YBR040W | 19.1580314284008 | 1.6158822346771 | 0.673312763844267 | 2.39989841489303 | 0.0163996223053834 | 0.036045586227532 | 3.02370334164612 | 8.2439483077042 | 16.8251572712098 | 22.6524566204991 | 31.4256438296121 | 32.7772791997335 |
| YBR041W | 3344.80158830327 | 0.954395386900102 | 0.310160213420111 | 3.0771044950481 | 0.00209022013156586 | 0.00647164309965584 | 2171.01899930191 | 2159.09006178773 | 2500.36467621718 | 3397.86849307487 | 4845.83427852618 | 4994.63302091178 |
| YBR042C | 1946.3687917255 | 1.21049218057897 | 0.34311194268354 | 3.52798031776888 | 0.000418743291341561 | 0.00175678301865124 | 1184.53578408987 | 1258.02651175566 | 1080.46770824247 | 1887.99513448545 | 2886.44538574987 | 3380.74222602966 |
| YBR043C | 1227.62780243611 | -0.247905888835748 | 0.272810061184872 | -0.908712412434644 | 0.363501950458406 | 0.460636080470708 | 1285.82984603501 | 1256.37772209412 | 1455.74186824815 | 1037.65676288517 | 1150.1785641638 | 1179.98205119041 |
| YBR044C | 425.085712124421 | -0.91625907545797 | 0.575638151789979 | -1.59172749861838 | 0.111445946797567 | 0.175808942128464 | 485.304386334202 | 520.193138216135 | 663.496419347273 | 585.478878806747 | 157.12821914806 | 138.913230894109 |
| YBR045C | 103.693592816609 | -1.86233179464446 | 0.414999032057633 | -4.48755695985774 | 7.20445538470775e-06 | 7.1625903264829e-05 | 191.249236359117 | 165.703360984854 | 132.406672438651 | 59.2448865459209 | 29.8543616381315 | 43.703038932978 |
| YBR046C | 323.940466459462 | 0.549351832795425 | 0.383535149122825 | 1.43233764637175 | 0.152047214047566 | 0.226574904507889 | 207.879604738171 | 241.547685415733 | 338.697731155223 | 269.215734451317 | 432.102602657166 | 454.199440339165 |
| YBR047W | 68.0387386204352 | -1.25635374661296 | 0.399853749137792 | -3.14203317918625 | 0.00167779022021242 | 0.00540609923837978 | 111.877023640906 | 102.224959015532 | 73.1528577009121 | 34.8499332623064 | 42.4246191699763 | 43.703038932978 |
| YBR048W | 50933.083278322 | 0.299193913544167 | 0.273335431132334 | 1.09460347787592 | 0.273690391273602 | 0.365390435899146 | 46648.9392290809 | 50424.9342189035 | 39937.802661813 | 55357.3764905106 | 54097.6745704857 | 59131.7724991383 |
| YBR049C | 564.439178667348 | -2.29725439430034 | 0.518834443374145 | -4.42772145072052 | 9.52337768182784e-06 | 8.80863421471746e-05 | 1291.12132688289 | 1190.42613563249 | 332.84550253915 | 207.357102910723 | 182.26873421175 | 182.616269827087 |
| YBR050C | 32.4329368903808 | -0.739518756921205 | 0.470634039207623 | -1.5713244162413 | 0.116107312595751 | 0.18184121914315 | 41.5759209476341 | 42.8685312000618 | 38.0394860044743 | 31.3649399360757 | 25.1405150636897 | 15.6082281903493 |
| YBR051W | 2.21118774629313 | 0.989394219017566 | 1.62469322976073 | 0.60897294387278 | 0.542542372746037 | 0.633195520203784 | 0 | 0 | 4.38917146205473 | 2.61374499467298 | 3.14256438296121 | 3.12164563806986 |
| YBR052C | 2914.44432185014 | 1.56804507113246 | 0.338140927552005 | 4.63725311953341 | 3.53069936899775e-06 | 4.19514832773902e-05 | 1285.0739201996 | 1242.36300997102 | 1882.22302864447 | 3518.97201116139 | 4622.71220733594 | 4935.32175378845 |
| YBR053C | 1417.69100975773 | 1.791385265831 | 0.439617070837976 | 4.07487648834098 | 4.60387752678645e-05 | 0.000305974935732763 | 481.524757157144 | 527.612691693069 | 896.854035413182 | 1283.34879238443 | 2675.89357209147 | 2640.9122098071 |
| YBR054W | 9716.7593268977 | 0.541498722000928 | 0.393503399205432 | 1.37609668199647 | 0.168791702668574 | 0.246769254298665 | 9990.31584079877 | 8194.48461785797 | 5557.42259953829 | 7704.44899596438 | 13354.3273453937 | 13499.5565618331 |
| YBR055C | 917.259349964978 | 0.391620498845355 | 0.30924657165049 | 1.26636973453004 | 0.205380741263712 | 0.290565020592773 | 890.480634114782 | 845.82909637045 | 643.745147768027 | 899.999526499062 | 1071.61445458977 | 1151.88724044778 |
| YBR056C-B | 5.60849621137069 | -0.808094850122645 | 0.853251749930948 | -0.947076698275793 | 0.343599697518422 | 0.439916908950027 | 7.5592583541153 | 6.59515864616336 | 7.31528577009121 | 4.3562416577883 | 3.14256438296121 | 4.68246845710479 |
| YBR056W | 1585.12406176402 | 0.886742635625799 | 0.311668229573457 | 2.84514926927066 | 0.00443906154110091 | 0.0120724477722508 | 1015.9643227931 | 975.259084801406 | 1347.4756388508 | 2380.25044181553 | 1959.38889277631 | 1832.40598954701 |
| YBR056W-A | 1552.80777663871 | 0.854126884198321 | 0.400130590998436 | 2.13462030500352 | 0.0327920374930324 | 0.0637622951253407 | 1260.88429346643 | 1093.14754560158 | 963.423135921012 | 1143.94905933521 | 2537.62073924118 | 2317.82188626687 |
| YBR057C | 153.480348633082 | -3.90143784253418 | 0.733322374459679 | -5.32022201751147 | 1.03640696030525e-07 | 2.9019394888547e-06 | 442.972539551156 | 356.962961723592 | 64.3745147768027 | 33.1074365991911 | 7.85641095740302 | 15.6082281903493 |
| YBR058C | 1620.05369564108 | 0.0804408785992899 | 0.447092721600429 | 0.179919901874808 | 0.857215450723194 | 0.895307736402427 | 1444.57427147143 | 1591.08202338691 | 1690.56254146808 | 2746.17474106974 | 1224.02882716339 | 1023.89976928691 |
| YBR058C-A | 1307.01510157473 | 0.0124737052819483 | 0.386874708571529 | 0.0322422350326424 | 0.974278874988361 | 0.981288075527846 | 1437.01501311732 | 1244.83619446333 | 1220.92119502822 | 775.411015086317 | 1659.27399420352 | 1504.63319754967 |
| YBR059C | 428.281822522437 | -1.8422546877312 | 0.485028371786319 | -3.79824108215842 | 0.000145726548538478 | 0.000753193396940449 | 896.528040798074 | 833.463173908894 | 279.443916417484 | 190.803384611127 | 164.984630105463 | 204.467789293576 |
| YBR060C | 511.683510497762 | -0.871166453231665 | 0.411463029135121 | -2.1172411408695 | 0.0342393874164094 | 0.0660778108965167 | 746.09879955118 | 777.404325416506 | 463.057589246774 | 516.650260613692 | 281.259512275028 | 285.630575883392 |
| YBR061C | 1674.1709084325 | 0.462441208448975 | 0.27619682628831 | 1.67431760409242 | 0.0940681965352943 | 0.152426985649622 | 1368.22576209487 | 1417.95910892512 | 1438.91671097694 | 2205.12952717244 | 1865.11196128748 | 1749.68238013816 |
| YBR062C | 374.915115343636 | -0.390792171537543 | 0.349243542257253 | -1.11896749475094 | 0.263154006533753 | 0.353875924426262 | 405.17624778058 | 343.772644431265 | 528.163632600585 | 400.774232516523 | 285.97335884947 | 285.630575883392 |
| YBR063C | 343.975602382755 | -1.12769432488648 | 0.376215065585043 | -2.9974725311248 | 0.00272228390544575 | 0.0080346051104815 | 486.816238005025 | 503.705241600726 | 427.212688973327 | 301.45192271895 | 193.267709552114 | 151.399813446388 |
| YBR065C | 123.787952080492 | -2.56223758825527 | 0.667326506501877 | -3.83955614424266 | 0.000123256941403017 | 0.000657098263771053 | 215.438863092286 | 244.020869908044 | 177.761444213216 | 75.7986048455164 | 7.85641095740302 | 21.851519466489 |
| YBR066C | 100.901306555181 | -2.94479366673521 | 0.533185421636195 | -5.52301984870193 | 3.33222074631255e-08 | 1.21928986399164e-06 | 247.943674014982 | 180.542467938722 | 108.997757974359 | 36.5924299254217 | 17.2841041062866 | 14.0474053713144 |
| YBR067C | 19783.1243575227 | 2.79468013322723 | 0.408767881833889 | 6.83683884528604 | 8.09597259451854e-12 | 3.38828639432281e-09 | 5013.30014044926 | 5061.78426093038 | 4876.3694943428 | 18336.2923859625 | 42388.4796795722 | 43022.5201838788 |
| YBR068C | 20675.8012470185 | 0.696529992278821 | 0.389136117509768 | 1.78993920362927 | 0.0734636855670953 | 0.12446885952436 | 17529.9201231934 | 16792.0983079627 | 13015.3564421463 | 15311.3181787943 | 31688.0479555893 | 29718.0664744251 |
| YBR069C | 8130.55347212064 | 0.552353962068714 | 0.345308567075085 | 1.59959530325991 | 0.109688391069831 | 0.173321646762829 | 7491.98095476367 | 7618.23263114945 | 4667.8838498952 | 7968.43724042635 | 10788.4235267058 | 10248.3626297834 |
| YBR070C | 1663.69154988084 | 1.14935777878388 | 0.422102157049917 | 2.7229374680688 | 0.00647043037785187 | 0.0165548488349139 | 1057.54024374073 | 1205.26524258635 | 838.331749252453 | 1234.5588858172 | 2723.03203783589 | 2923.42114005242 |
| YBR071W | 1995.84433350505 | -0.772512931787454 | 0.290703598865241 | -2.65739032747772 | 0.00787482081930738 | 0.0195053254139767 | 2500.60266354134 | 2414.65245932656 | 2639.35510584891 | 1737.26917312597 | 1426.72422986439 | 1256.46236932312 |
| YBR072W | 950.425940481597 | 1.71420075881592 | 0.38164136930603 | 4.49165341255586 | 7.06723659745336e-06 | 7.04535660798756e-05 | 345.458106783069 | 344.597039262035 | 641.550562036999 | 1250.24135578524 | 1500.57449286398 | 1620.13408615826 |
| YBR073W | 1323.35647958708 | -1.35192503861358 | 0.492924063219915 | -2.74266391010095 | 0.00609430258353611 | 0.0157619713405512 | 2100.71789660864 | 2059.33828726451 | 1547.18294037429 | 1308.6149939996 | 510.666712231196 | 413.618047044256 |
| YBR074W | 2255.46279294521 | -0.0181706540412325 | 0.321018350756786 | -0.0566031630229113 | 0.954861314858875 | 0.967937489011673 | 2268.53343207 | 2277.80291741867 | 2264.08094584323 | 2915.19691739193 | 1923.24940237226 | 1883.91314257516 |
| YBR076C-A | 445.263717083045 | 1.28560725033315 | 0.386986319845533 | 3.32209999269821 | 0.000893426704316037 | 0.00324515960281742 | 269.865523241916 | 248.967238892667 | 257.498059107211 | 376.379279232909 | 732.217501229961 | 786.654700793605 |
| YBR076W | 47.2702234644679 | -1.67423200918699 | 0.433743041807753 | -3.85996280703231 | 0.000113404284442443 | 0.000618918298143501 | 75.592583541153 | 64.3027968000927 | 75.3474434319395 | 18.2962149627109 | 20.4266684892478 | 29.6556335616637 |
| YBR077C | 641.410331997888 | 0.645811088771736 | 0.285261208645118 | 2.2639288806182 | 0.0235784893165154 | 0.0487668209878035 | 444.484391221979 | 514.422374400742 | 541.33114698675 | 721.393618529742 | 771.499556016976 | 855.330904831142 |
| YBR078W | 93667.7826711197 | 1.74979232355075 | 0.409091796644578 | 4.27726084439425 | 1.89207052760339e-05 | 0.000149876189394414 | 37081.1859302772 | 39744.0747914419 | 51983.1522108451 | 81087.9534664029 | 181195.548474969 | 170914.781152782 |
| YBR079C | 3155.20911190145 | -2.93037485422244 | 0.707427341177535 | -4.14229799111911 | 3.43843184651384e-05 | 0.000242270252642769 | 7985.6005252874 | 7896.05368911908 | 855.156906523662 | 1140.46406600898 | 532.664662911925 | 521.314821557667 |
| YBR080C | 3193.59612430139 | 0.80425732734158 | 0.279633312645121 | 2.87611415011291 | 0.004026040859313 | 0.0111229957674713 | 2107.52122912734 | 2197.01222400317 | 2673.00542039133 | 4287.41303959524 | 3815.07316091491 | 4081.55167177634 |
| YBR081C | 483.580452816318 | -0.841864481260796 | 0.393258487340699 | -2.14074078083774 | 0.0322949478935856 | 0.0629515313286616 | 611.544000847927 | 679.301340554826 | 573.518404375151 | 514.036515619019 | 265.546690360222 | 257.535765140763 |
| YBR082C | 4550.07935841482 | -1.67281386251824 | 0.599246370647507 | -2.7915294016895 | 0.00524595913440712 | 0.0139093529462475 | 6158.52778109773 | 5653.69974942354 | 8972.19799701687 | 4445.98023593874 | 1000.90675597314 | 1069.16363103893 |
| YBR083W | 325.24949212986 | -3.12803117000196 | 0.650132898483726 | -4.81137191687626 | 1.49897783388334e-06 | 2.13099718547653e-05 | 831.518418952682 | 788.945853047292 | 131.675143861642 | 97.5798131344579 | 48.7097479358987 | 53.0679758471876 |
| YBR084C-A | 23003.6287330229 | -1.62603854542964 | 0.73396695506356 | -2.21541110837725 | 0.0267318595653611 | 0.0539496006270527 | 42872.3337553649 | 39860.3144625806 | 21517.1815641463 | 26537.3529309148 | 3309.12029525815 | 3925.46938987285 |
| YBR084W | 9859.13338747698 | -0.17864288438692 | 0.560901555026968 | -0.318492403499096 | 0.750111456027658 | 0.80888586535676 | 10622.2698392028 | 10572.0393097999 | 10213.6019922013 | 18342.3911242834 | 4536.2916868045 | 4868.20637256995 |
| YBR085C-A | 2860.1091560545 | 1.15702835081544 | 0.386523022407105 | 2.9934267397837 | 0.00275863728856595 | 0.00812700097820892 | 1706.88053635923 | 1547.38909735608 | 2057.78988712666 | 2375.89420015774 | 4699.70503471849 | 4772.99618060882 |
| YBR085W | 590.877910788626 | 1.93691804253944 | 0.368236229902304 | 5.2599877069492 | 1.44065032605843e-07 | 3.74059318155718e-06 | 236.604786483809 | 249.791633723437 | 247.256659029083 | 597.676355448554 | 1041.76009295164 | 1172.17793709523 |
| YBR086C | 4657.46633607449 | -1.51670693772456 | 0.735144314842151 | -2.06314176292069 | 0.0390991612899856 | 0.0741238147505172 | 6975.68360917759 | 7080.72720148713 | 6653.25240789796 | 5808.61262649492 | 692.935446442946 | 733.586724946417 |
| YBR087W | 1745.32230283423 | -0.13485174343968 | 0.44085342989872 | -0.305887930758893 | 0.759689975658287 | 0.816074445795334 | 1848.9945934166 | 1932.38148332586 | 1700.80394154621 | 2729.62102277015 | 1181.60420799341 | 1078.52856795314 |
| YBR088C | 3744.85836333633 | 0.442976678236927 | 0.287173003343377 | 1.54254290298748 | 0.122941720680282 | 0.190919865247411 | 3229.31516887805 | 3408.04823040491 | 2885.14870772397 | 3634.84803925856 | 4677.70708403776 | 4634.08294971471 |
| YBR089C-A | 1635.67254190417 | -0.156166557128983 | 0.28514576974201 | -0.547672712347361 | 0.583916652180721 | 0.67047648376474 | 1688.73831630936 | 1705.672904864 | 1776.88291355516 | 1291.19002736845 | 1711.12630652238 | 1640.42478280571 |
| YBR089W | 12.6677054959586 | 1.11159456837166 | 0.713758878372062 | 1.55738107371355 | 0.119380036941632 | 0.186089494779003 | 6.04740668329224 | 6.59515864616336 | 10.9729286551368 | 6.96998665246128 | 23.5692328722091 | 21.851519466489 |
| YBR090C | 22.7475363911064 | 0.938493672553757 | 0.489667025728433 | 1.91659561139051 | 0.0552893259903453 | 0.0984143889933177 | 15.1185167082306 | 16.4878966154084 | 15.3621001171915 | 33.1074365991911 | 32.9969260210927 | 23.412342285524 |
| YBR091C | 600.826186452049 | 1.51629061394659 | 0.37108943227885 | 4.08605172245162 | 4.38776008335764e-05 | 0.000295577143690619 | 291.78737246885 | 276.996663138861 | 364.301231350542 | 586.350127138305 | 977.337523100936 | 1108.1842015148 |
| YBR092C | 14319.5742514758 | -0.440407344703214 | 0.354475885384238 | -1.24241835019561 | 0.214082248632476 | 0.300433576202473 | 19363.7961999017 | 19352.6686523356 | 10749.080910572 | 12745.491842357 | 11115.2502225338 | 12591.1576811548 |
| YBR093C | 9891.17435515165 | 2.12649779742175 | 0.475865256231536 | 4.46869732466261 | 7.8697394708623e-06 | 7.63309222131213e-05 | 4639.11685192056 | 4848.26599976084 | 1571.32338341559 | 13075.6949600174 | 16485.8927530145 | 18726.7521827811 |
| YBR094W | 877.767953227789 | -0.3355519291703 | 0.443979050896672 | -0.75578324808932 | 0.449779162111175 | 0.546701734093548 | 927.521000049947 | 986.800612432192 | 1025.60306496679 | 1277.25005406353 | 553.091331401173 | 496.341656453108 |
| YBR095C | 260.811700460587 | -2.28465984362101 | 0.487155819189014 | -4.68979278010958 | 2.73481864243481e-06 | 3.42650429129965e-05 | 513.273642244429 | 485.568555323777 | 301.389773727758 | 137.65723638611 | 83.277956148472 | 43.703038932978 |
| YBR096W | 3251.6756177868 | 1.08112507678254 | 0.363193223269282 | 2.9767215011635 | 0.00291348536959911 | 0.00848997546616211 | 2153.63270508745 | 2318.19826412642 | 1789.31889936431 | 2884.70322578741 | 4974.67941822759 | 5389.52119412761 |
| YBR097W | 655.504111709544 | 0.314676773184079 | 0.318068116466401 | 0.989337682380747 | 0.322497953225294 | 0.417968770128978 | 568.45622822947 | 576.251986708523 | 607.16871891757 | 556.727683865344 | 878.346745037657 | 746.073307498697 |
| YBR098W | 55.7610320679504 | -2.66625340899709 | 0.576490389057697 | -4.62497460426916 | 3.74644300941793e-06 | 4.39473460485455e-05 | 117.924430324199 | 130.254383261726 | 40.9656003125108 | 15.6824699680379 | 12.5702575318448 | 17.1690510093842 |
| YBR099C | 118.687902516015 | 1.27449444073114 | 0.390998992853487 | 3.25958497087157 | 0.00111575353047511 | 0.00389878064908285 | 65.0096218453915 | 64.3027968000927 | 78.273557739976 | 111.51978643938 | 188.553862977672 | 204.467789293576 |
| YBR101C | 1887.45440181099 | -0.283981960227575 | 0.406344841941784 | -0.69886936147761 | 0.484633676438456 | 0.579257772135051 | 1892.83829187047 | 1690.00940307936 | 2636.42899154087 | 2489.15648326023 | 1225.60010935487 | 1390.69313176012 |
| YBR102C | 545.839491740856 | -1.04094376596767 | 0.477597817889348 | -2.17954045637797 | 0.0292915426672826 | 0.0580423183930154 | 750.634354563649 | 814.502092801175 | 640.81903345999 | 611.616328753477 | 256.118997211338 | 201.346143655506 |
| YBR103C-A | 4.21493114874027 | 1.88469686668284 | 1.04931142587586 | 1.79612726994722 | 0.0724742783273761 | 0.123083953699447 | 1.51185167082306 | 0.82439483077042 | 2.92611430803648 | 4.3562416577883 | 9.42769314888362 | 6.24329127613972 |
| YBR103W | 689.711324970651 | -0.275108953238897 | 0.374040966383383 | -0.73550487236448 | 0.462032073711554 | 0.558567027351682 | 747.610651222003 | 764.214008124179 | 755.669020050422 | 899.999526499062 | 460.385682103817 | 510.389061824422 |
| YBR104W | 2905.77248884699 | 1.23092818293684 | 0.365744084611346 | 3.36554502103533 | 0.000763925995426517 | 0.00286361083268147 | 1845.21496423954 | 1975.25001452593 | 1387.7097105863 | 2774.92593601115 | 4333.5962841035 | 5117.93802361554 |
| YBR105C | 1040.65563364226 | -0.962767254212934 | 0.484127160151826 | -1.98866606432699 | 0.0467380740878673 | 0.0858500193643508 | 1471.03167571084 | 1493.803433356 | 1163.86196602151 | 1230.20264415942 | 476.098504018623 | 408.935578587152 |
| YBR106W | 17998.6342170596 | 0.938555679534245 | 0.266909472480438 | 3.51638205572877 | 0.000437470977287494 | 0.00181996969879294 | 12499.2336885296 | 12951.2427914033 | 11575.7082025923 | 21675.787240823 | 23539.3785105709 | 25750.4548684383 |
| YBR107C | 170.750753312579 | -0.773373948300298 | 0.601996270718472 | -1.28468229110006 | 0.198903329063017 | 0.282642859480545 | 213.171085586051 | 204.449918031064 | 230.431501757873 | 254.404512814837 | 45.5671835529375 | 76.4803181327116 |
| YBR108W | 272.161431103845 | -1.5550476127205 | 0.56418011644544 | -2.75629638016654 | 0.0058459998658263 | 0.0152271544342423 | 439.192910374099 | 422.090153354455 | 359.180531311478 | 268.344486119759 | 84.8492383399526 | 59.3112671233273 |
| YBR109C | 6758.09429419917 | 0.956029428248172 | 0.394113785940109 | 2.42577007543058 | 0.0152759448498135 | 0.0339582619374523 | 4567.30389755646 | 4800.45109957615 | 4423.55330517415 | 5023.61787976147 | 10428.5999048568 | 11305.03967827 |
| YBR109W-A | 14.5569362012448 | 0.11589375351844 | 0.594493973075995 | 0.19494521183922 | 0.845435839204693 | 0.884757308990441 | 15.8744425436421 | 14.0147121230971 | 11.7044572321459 | 11.3262283102496 | 12.5702575318448 | 21.851519466489 |
| YBR110W | 2193.99410425475 | 0.714141667672857 | 0.380309936126757 | 1.8777886135345 | 0.0604100885515674 | 0.10588172792779 | 1576.10536683304 | 1819.43939151032 | 1588.88006926381 | 1631.8481250075 | 3288.6936267689 | 3258.99804614493 |
| YBR111C | 2136.17593937774 | -0.0607099751552046 | 0.300143227037613 | -0.202270015400336 | 0.839705639716231 | 0.880590183972077 | 2195.96455187049 | 2146.72413932617 | 2201.90101679745 | 2569.31132976354 | 1773.9775941816 | 1929.17700432717 |
| YBR111W-A | 191.145548540116 | -2.19813301191793 | 0.432765137035133 | -5.07927470077026 | 3.7887854189954e-07 | 7.55411086635615e-06 | 405.932173615991 | 304.201692554285 | 233.35761606591 | 98.4510614660155 | 53.4235945103405 | 51.5071530281527 |
| YBR112C | 352.095188218643 | -1.55488783812589 | 0.526531480516454 | -2.95307668327972 | 0.0031462379625505 | 0.00903736506638055 | 666.726586832969 | 704.857580308709 | 205.559530139563 | 245.69202949926 | 147.700525999177 | 142.034876532179 |
| YBR114W | 784.199299862105 | -0.0313478101117181 | 0.274300952427102 | -0.114282541982967 | 0.909013821301911 | 0.934852923855686 | 765.752871271879 | 777.404325416506 | 834.674106367407 | 740.561081824011 | 854.777512165448 | 732.025902127382 |
| YBR115C | 6949.27094047431 | 0.342660138542116 | 0.279673444905049 | 1.22521513852862 | 0.220494172105466 | 0.307488624590559 | 6062.52520000047 | 6493.7580819786 | 5826.62511587765 | 6746.07583125096 | 7747.99248619086 | 8818.64892754735 |
| YBR116C | 24.2440099840511 | 1.72288891929136 | 0.681742850920594 | 2.52718296490363 | 0.0114981583809241 | 0.0267805207859499 | 15.8744425436421 | 6.59515864616336 | 10.9729286551368 | 14.8112216364802 | 65.9938520421854 | 31.2164563806986 |
| YBR117C | 232.504287576204 | 1.88319850273531 | 0.446012319670347 | 4.22230153670912 | 2.41820298216773e-05 | 0.000183214437707767 | 105.073691122203 | 99.7517745232208 | 92.1726007031492 | 182.090901295551 | 428.960038274205 | 486.976719538898 |
| YBR118W | 127264.315106769 | 1.63783015501714 | 0.372505750807712 | 4.39679159708486 | 1.09862735857449e-05 | 9.89532893597165e-05 | 60282.0616707278 | 61634.2307328889 | 63781.2451008483 | 117437.30510732 | 215796.753613563 | 244654.294415268 |
| YBR119W | 147.93453309108 | -2.12934102154509 | 0.507045588803218 | -4.19950605737638 | 2.6749787458029e-05 | 0.000197782584649491 | 337.898848428954 | 277.821057969631 | 107.534700820341 | 68.8286181930551 | 47.1384657444181 | 48.3855073900828 |
| YBR120C | 153.049878729607 | -1.56714184021581 | 0.446690702054764 | -3.50833772229196 | 0.000450916234508224 | 0.00186386428127918 | 201.832198054878 | 181.366862769492 | 305.047416612803 | 114.133531434053 | 62.8512876592241 | 53.0679758471876 |
| YBR121C | 27489.2639695956 | 1.21904889677766 | 0.286358298720656 | 4.25707549676025 | 2.07118428503889e-05 | 0.000160703937296993 | 15677.1459005997 | 16717.9027731933 | 17165.318059519 | 31628.9281805377 | 41788.2498824267 | 41958.039021297 |
| YBR122C | 1380.59008631363 | 1.07190426927333 | 0.270773988348653 | 3.95866780192019 | 7.53689801326991e-05 | 0.000452776335871812 | 811.108421396571 | 897.765970708987 | 961.228550189985 | 1830.49274460264 | 1802.26067362825 | 1980.68415735533 |
| YBR123C | 229.191586796907 | -1.75105150836046 | 0.615430391921548 | -2.84524705205601 | 0.00443769895981368 | 0.0120724477722508 | 352.261439301773 | 328.933537477397 | 381.126388621752 | 220.425827884088 | 47.1384657444181 | 45.263861752013 |
| YBR124W | 3.31701746069246 | 2.02767913070274 | 1.18464655779517 | 1.71163214661814 | 0.0869644802430805 | 0.142797361745395 | 0.75592583541153 | 1.64878966154084 | 1.46305715401824 | 3.48499332623064 | 9.42769314888362 | 3.12164563806986 |
| YBR125C | 914.541348128529 | 0.289534594184576 | 0.302671982165523 | 0.956595295385609 | 0.338771564349163 | 0.434911411075182 | 738.539541197064 | 804.609354831929 | 926.846707070556 | 1174.44275093973 | 859.49135873989 | 983.318375992006 |
| YBR126C | 9101.43929288051 | 1.5326863044797 | 0.329212709176004 | 4.65561098268625 | 3.2302134508991e-06 | 3.88833170538135e-05 | 3985.996930125 | 3850.74825452863 | 6189.46329007417 | 12762.0455606566 | 14110.1140794958 | 13710.2676424028 |
| YBR126W-A | 9.33313429868615 | 1.92609647461769 | 0.841115890314262 | 2.28992995709313 | 0.0220253773142411 | 0.0460829856737207 | 1.51185167082306 | 5.77076381539294 | 4.38917146205473 | 13.0687249733649 | 6.28512876592241 | 24.9731651045589 |
| YBR126W-B | 385.27152086297 | 1.97029180304532 | 0.342048656800187 | 5.76026762238186 | 8.39806749590849e-09 | 4.32668437389206e-07 | 165.547757955125 | 152.513043692528 | 151.426415440888 | 437.366662441945 | 708.648268357752 | 696.126977289579 |
| YBR127C | 23600.9795892213 | 1.94402714810729 | 0.384855453756444 | 5.05131765480336 | 4.38772678848287e-07 | 8.26226915726014e-06 | 7173.73617805541 | 7998.27864813461 | 14038.033392805 | 30810.8259972051 | 38339.2854721267 | 43245.7178470008 |
| YBR128C | 68.3109073487473 | -1.20216337406788 | 0.39278461045271 | -3.06061730036293 | 0.00220881220629557 | 0.00675760123921306 | 84.6636935660913 | 110.468907323236 | 91.4410721261401 | 49.6611548987866 | 40.8533369784957 | 32.7772791997335 |
| YBR129C | 608.742047371195 | 0.118927430939748 | 0.279857776366345 | 0.424956677937964 | 0.670868256109843 | 0.746008061052608 | 629.686220897804 | 586.969119508539 | 534.015861216658 | 595.933858785439 | 619.085183443358 | 686.762040375369 |
| YBR130C | 322.118208062399 | -2.29498012773889 | 0.484883878654403 | -4.7330510020413 | 2.21169886153899e-06 | 2.86010856793395e-05 | 745.342873715768 | 622.418097231667 | 238.478316104973 | 130.687249733649 | 102.133342446239 | 93.6493691420958 |
| YBR131W | 156.08860032587 | -0.745709545518102 | 0.42699288360194 | -1.74642148418868 | 0.0807377394290403 | 0.134528083291855 | 170.839238803006 | 186.313231754115 | 231.163030334882 | 175.992162974647 | 80.1353917655108 | 92.0885463230609 |
| YBR132C | 490.240609053058 | 1.17553157084714 | 0.467060622991558 | 2.5168715001444 | 0.01184020031845 | 0.0273693072687789 | 222.998121446401 | 279.469847631172 | 399.41460304698 | 337.173104312814 | 867.347769697293 | 835.040208183688 |
| YBR133C | 1732.83271839126 | -0.353416406759359 | 0.474269213942608 | -0.7451809992502 | 0.456162307770632 | 0.552819958984357 | 1730.31423725699 | 1755.96098954099 | 2347.47520362227 | 2604.16126302584 | 975.766240909455 | 983.318375992006 |
| YBR134W | 2.51452176848905 | 0.611823081146258 | 1.39429992943364 | 0.438803063982638 | 0.660804240000443 | 0.737930367931618 | 0 | 1.64878966154084 | 4.38917146205473 | 4.3562416577883 | 1.5712821914806 | 3.12164563806986 |
| YBR135W | 665.152409710839 | -0.398042680231702 | 0.63058497917608 | -0.631227659040949 | 0.52789167945284 | 0.620224390794825 | 723.421024488834 | 642.203573170157 | 904.900849760283 | 1218.00516751761 | 204.266684892478 | 298.117158435672 |
| YBR136W | 682.704873109149 | -0.456267899369318 | 0.341818293491327 | -1.33482586525433 | 0.181933345909672 | 0.262760876353059 | 773.312129625995 | 752.672480493393 | 844.915506445535 | 770.183525096971 | 457.243117720856 | 497.902479272143 |
| YBR137W | 1330.30970298733 | 1.28648840224071 | 0.379309512853646 | 3.39165868148711 | 0.00069470922677513 | 0.00265043093627479 | 716.61769197013 | 745.252927016459 | 858.083020831699 | 1145.69155599832 | 2223.36430094505 | 2292.84872116231 |
| YBR138C | 64.1979064063863 | -3.68933226861017 | 0.740681702032884 | -4.98099555920496 | 6.32579916986412e-07 | 1.08346134717886e-05 | 162.524054613479 | 155.810623015609 | 40.2340717355017 | 15.6824699680379 | 1.5712821914806 | 9.36493691420958 |
| YBR139W | 2890.07475626246 | 1.22223987675739 | 0.354010396572718 | 3.452553621561 | 0.000555307094526916 | 0.00221298124304043 | 1553.42759177069 | 1527.60362141759 | 2120.70134474944 | 2851.59578918822 | 4723.2742675907 | 4563.84592285814 |
| YBR140C | 3655.23631144794 | 0.866124165536574 | 0.354258766043042 | 2.44489127315297 | 0.0144895815946375 | 0.03252454007301 | 2425.01008000019 | 2705.66383458852 | 2637.89204869489 | 3074.63536206698 | 5647.18819618129 | 5441.02834715577 |
| YBR141C | 273.686603890329 | -0.846544440990251 | 0.433480307484158 | -1.95290172673228 | 0.0508312410678027 | 0.0918241774128048 | 384.010324389057 | 365.206910031296 | 307.97353092084 | 309.293157702969 | 139.844115041774 | 135.791585256039 |
| YBR141W-A | 5.2378463442774 | 0.597870089858583 | 0.910369605332978 | 0.656733360116856 | 0.51135238001554 | 0.60491467511076 | 6.04740668329224 | 3.29757932308168 | 2.92611430803648 | 3.48499332623064 | 9.42769314888362 | 6.24329127613972 |
| YBR142W | 1407.48104579073 | -3.21682501121525 | 0.731442664942825 | -4.39791820383721 | 1.09294151067544e-05 | 9.87172977384267e-05 | 2929.21261221968 | 3018.93387028128 | 1678.85808423593 | 645.595013684226 | 89.5630849143944 | 82.7236094088513 |
| YBR143C | 17817.0542587959 | 1.07725662791922 | 0.309868776154608 | 3.47649298934761 | 0.000508017510200916 | 0.00204989521660019 | 11423.551224739 | 12238.9656576176 | 10710.3098959905 | 18460.8808973752 | 26050.2874525569 | 28018.330424496 |
| YBR144C | 4.97588532594266 | 1.50207526306592 | 0.974551677310018 | 1.5412987305219 | 0.123244097726253 | 0.191155682466458 | 3.02370334164612 | 1.64878966154084 | 2.92611430803648 | 3.48499332623064 | 6.28512876592241 | 12.4865825522794 |
| YBR145W | 2048.65266549703 | 1.45735553222518 | 0.443644399143796 | 3.28496321612035 | 0.00101995686591767 | 0.00362376596992308 | 1170.17319321705 | 1286.88033083262 | 823.70117771227 | 1525.55582855746 | 3736.50905134088 | 3749.0964113219 |
| YBR146W | 1227.2527173521 | -0.118494312940308 | 0.422218594351391 | -0.280646836793954 | 0.778981288531991 | 0.831395342748761 | 1127.841346434 | 1115.40620603238 | 1591.07465499484 | 1805.22654298747 | 826.494432718797 | 897.473120945085 |
| YBR147W | 314.284033561022 | 0.0710484000496554 | 0.448883688293455 | 0.158277972451536 | 0.874237764776941 | 0.90793278586736 | 309.929592518727 | 370.153279015918 | 238.478316104973 | 163.79468633284 | 397.534394444593 | 405.813932949082 |
| YBR148W | 12.5000705249673 | -1.61022057660851 | 0.68351059281017 | -2.35580924940502 | 0.0184824057374944 | 0.0397418006509062 | 18.1422200498767 | 16.4878966154084 | 21.2143287332645 | 3.48499332623064 | 9.42769314888362 | 6.24329127613972 |
| YBR149W | 2983.19631587216 | 0.686684669323691 | 0.465801369218649 | 1.47420062434672 | 0.140427581824818 | 0.212941556684187 | 1642.62684034925 | 1693.30698240244 | 3523.77315545294 | 5408.70964230995 | 2710.46178030404 | 2920.29949441435 |
| YBR150C | 357.884420063851 | -0.517589837693535 | 0.359293028029951 | -1.44057857323741 | 0.149703777347509 | 0.223843121921978 | 365.112178503769 | 464.134289723746 | 433.796446166409 | 215.198337894742 | 389.67798348719 | 279.387284607252 |
| YBR151W | 3169.55300287587 | 0.656520215896591 | 0.313631765476233 | 2.09328355149135 | 0.0363238500833942 | 0.0696207126598389 | 2247.36750867848 | 2340.45692455722 | 2792.97610702082 | 3011.90548219483 | 4380.73474984792 | 4243.87724495597 |
| YBR152W | 12.1478025268912 | -5.70400298497108 | 1.39368070154635 | -4.09276169114075 | 4.26265747260712e-05 | 0.000288356240794011 | 30.9929592518727 | 32.9757932308168 | 8.04681434710033 | 0.87124833155766 | 0 | 0 |
| YBR153W | 738.590624696963 | 1.04435959585939 | 0.382097907926432 | 2.73322510852498 | 0.00627174515112651 | 0.0161485795695313 | 449.019946234449 | 468.256263877598 | 528.895161177595 | 600.290100443228 | 1194.17446552526 | 1190.90781092365 |
| YBR154C | 2100.25057315395 | -1.79632944995571 | 0.548395366277336 | -3.27561019005267 | 0.00105433975427324 | 0.00371612167793764 | 3728.98214608507 | 3403.10186142029 | 2654.7172059661 | 1807.84028798214 | 488.668761550468 | 518.193175919597 |
| YBR155W | 100.762784823526 | -2.07566229283625 | 0.558713818291245 | -3.71507241253564 | 0.000203145511263521 | 0.000979570492827334 | 235.092934812986 | 195.381574892589 | 58.5222861607297 | 43.562416577883 | 32.9969260210927 | 39.0205704758732 |
| YBR156C | 60.2981019558351 | -1.58790730580662 | 0.429780112937264 | -3.69469702763657 | 0.000220149130312296 | 0.00103637456082689 | 99.7822102743219 | 106.346933169384 | 65.1060433538118 | 27.8799466098451 | 36.1394904040539 | 26.5339879235938 |
| YBR157C | 310.37208878994 | -0.386714667044157 | 0.558606507393369 | -0.69228457228092 | 0.48875863666801 | 0.583430142751063 | 272.889226583562 | 281.118637292713 | 502.560132405266 | 492.255307330078 | 186.982580786192 | 126.426648341829 |
| YBR158W | 4740.9746119046 | -0.657557908137978 | 0.335710527562842 | -1.95870505733512 | 0.0501473378376153 | 0.0908435599646252 | 6568.23958389078 | 6392.35751779383 | 4449.88833394648 | 4506.96761914777 | 3437.96543495956 | 3090.42918168916 |
| YBR159W | 7664.30390154934 | 1.16814181800513 | 0.335137029386473 | 3.4855647558362 | 0.000491099342618652 | 0.00199538155612878 | 4970.21236783081 | 5050.24273329959 | 4140.45174587163 | 7560.69302125737 | 11674.6266827009 | 12589.5968583357 |
| YBR160W | 1675.8279495248 | -0.0960596829276055 | 0.287917430085041 | -0.333636219589876 | 0.738654083520788 | 0.800156820500231 | 1775.66978738168 | 1903.5276642489 | 1516.45874013991 | 1830.49274460264 | 1530.42885450211 | 1498.38990627353 |
| YBR161W | 511.784599116813 | 0.387924167987011 | 0.330037815808834 | 1.17539308953525 | 0.23983748691437 | 0.329118562908277 | 393.081434413995 | 413.846205046751 | 522.311403984512 | 449.564139083752 | 634.798005358164 | 657.106406813706 |
| YBR162C | 52985.2067038656 | 1.47435890522611 | 0.394032296107187 | 3.74172097006244 | 0.00018276437677527 | 0.000901227095277746 | 28767.5135924212 | 30164.6068578897 | 25201.8910065412 | 44283.8101964127 | 96395.0198829521 | 93098.3986869765 |
| YBR162W-A | 1051.58825530056 | -1.06985065607929 | 0.347061779150213 | -3.08259428249009 | 0.00205204714946376 | 0.00637183396458371 | 1895.1060693767 | 1211.03600640175 | 1168.25113748357 | 771.054773428529 | 675.65134233666 | 588.430202776169 |
| YBR163W | 705.306192016681 | 0.0706725699766715 | 0.305141839157521 | 0.231605636813995 | 0.816844329004192 | 0.861667072692383 | 594.913632468874 | 677.652550893285 | 792.245448900878 | 829.428411642892 | 699.220575208869 | 638.376532985286 |
| YBR164C | 2244.10267700572 | 0.764589096556243 | 0.390834742988866 | 1.95629766870041 | 0.0504300986694178 | 0.0912787620660625 | 1870.91644264354 | 1878.79581932579 | 1238.47788087644 | 1796.51405967189 | 3221.12849253524 | 3458.7833669814 |
| YBR165W | 433.68462439354 | -0.0835589730440533 | 0.304350313175333 | -0.274548667856687 | 0.783663008294853 | 0.8350082351785 | 463.382537107268 | 463.309894892976 | 411.119060279126 | 346.756835959949 | 435.245167040127 | 482.294251081793 |
| YBR166C | 1784.51513587806 | 0.169275535652767 | 0.27077438318898 | 0.625153434601772 | 0.531870360431103 | 0.623906215150511 | 1746.94460563604 | 1765.85372751024 | 1526.70014021804 | 1768.63411306205 | 1879.2535010108 | 2019.7047278312 |
| YBR167C | 317.942153959176 | -1.85423720239567 | 0.376335220328106 | -4.92708920727394 | 8.34636549155284e-07 | 1.35391923842822e-05 | 591.134003291816 | 565.534853908508 | 337.234674001205 | 125.459759744303 | 164.984630105463 | 123.305002703759 |
| YBR168W | 409.896743736965 | 0.275513238198371 | 0.352715418096004 | 0.781120484286232 | 0.434731635109501 | 0.53205468074975 | 309.929592518727 | 338.001880615872 | 463.789117823783 | 342.40059430216 | 482.383632784545 | 522.875644376702 |
| YBR169C | 1157.43436743451 | -0.844347490183334 | 0.63342284367005 | -1.33299185310588 | 0.182534477348652 | 0.263333789006567 | 1357.64280039911 | 1232.47027200178 | 1871.98162856634 | 1772.99035471984 | 370.822597189422 | 338.69855173058 |
| YBR170C | 577.852307178566 | 0.25034174511922 | 0.355772832833689 | 0.703656159255548 | 0.481646928653454 | 0.576727846266125 | 412.735506134695 | 461.661105231435 | 709.582719698847 | 727.492356850646 | 564.090306741537 | 591.551848414239 |
| YBR171W | 1484.3929450344 | 0.506809290871756 | 0.362914657252249 | 1.39649716743044 | 0.162564832994454 | 0.23978871380767 | 1143.71578897764 | 1117.87939052469 | 1416.23932508966 | 1141.33531434053 | 2019.09761605258 | 2068.09023522128 |
| YBR172C | 409.839547389435 | -1.42541705676447 | 0.49518839341536 | -2.87853486818066 | 0.00399527111879075 | 0.0110474650085927 | 795.233978852929 | 732.887004554903 | 264.813344877302 | 299.709426055835 | 174.412323254347 | 191.981206741296 |
| YBR173C | 1440.71411584428 | -0.89585406691813 | 0.461123869252198 | -1.94276229589011 | 0.0520448854396085 | 0.0935964988078969 | 1734.84979226946 | 1670.22392714087 | 2219.45770264567 | 1690.22176322186 | 659.938520421854 | 669.592989365985 |
| YBR174C | 12.5613862968921 | 0.677016432957733 | 0.600645846064543 | 1.12714744868973 | 0.259680144847743 | 0.350448477120592 | 9.82703586034988 | 8.2439483077042 | 10.9729286551368 | 16.5537182995955 | 17.2841041062866 | 12.4865825522794 |
| YBR175W | 1442.39421106859 | 1.58761090188068 | 0.403450960163601 | 3.93507776319802 | 8.31697627524194e-05 | 0.000485523370660568 | 717.373617805541 | 753.496875324163 | 689.099919542592 | 1186.64022758153 | 2723.03203783589 | 2584.72258832184 |
| YBR176W | 505.823579438324 | 0.795982396704307 | 0.315934133342733 | 2.51945678766152 | 0.0117536066402477 | 0.0272866715080011 | 363.600326832946 | 338.826275446642 | 407.46141739408 | 786.737243396567 | 540.521073869328 | 597.795139690378 |
| YBR177C | 9949.4357169477 | 2.07989271058551 | 0.426928634556027 | 4.87175734358609 | 1.10609968560651e-06 | 1.67384885798402e-05 | 2814.31188523712 | 3027.17781858898 | 5577.17387111754 | 10566.4997651313 | 18351.004714302 | 19360.4462473093 |
| YBR178W | 15.2165101763348 | 1.17856052846262 | 0.645624486367013 | 1.82545822432245 | 0.067931909841555 | 0.116444370343256 | 9.07111002493835 | 8.2439483077042 | 10.2414000781277 | 10.4549799786919 | 32.9969260210927 | 20.2906966474541 |
| YBR179C | 411.042944736795 | -0.895062760451097 | 0.327976100674185 | -2.72904872827994 | 0.00635173174853772 | 0.0163228860576947 | 514.02956807984 | 447.646393108338 | 643.013619191017 | 333.688110986584 | 282.830794466509 | 245.049182588484 |
| YBR180W | 34.4497289206298 | 0.186024078067028 | 0.497317490886972 | 0.37405496785414 | 0.708363421136173 | 0.776090580489444 | 29.4811075810496 | 30.5026087385055 | 35.8449002734469 | 20.0387116258262 | 45.5671835529375 | 45.263861752013 |
| YBR181C | 23350.2785865514 | 0.482612034281669 | 0.331865478786662 | 1.45423994097293 | 0.145879795073544 | 0.219501373895706 | 22209.8569702261 | 22487.8421937555 | 13744.6904334244 | 27906.9553081234 | 24961.3888938609 | 28790.9377199183 |
| YBR182C | 1076.18357914396 | -1.21930965344745 | 0.453045908734941 | -2.69136003645233 | 0.00711613511060764 | 0.0179365597308467 | 1522.43463251882 | 1614.98947347925 | 1381.85748197023 | 1079.47668279994 | 452.529271146414 | 405.813932949082 |
| YBR182C-A | 13.8134824548569 | 1.33751398173889 | 0.665902091415131 | 2.00857453217558 | 0.0445822744616911 | 0.0825265442751627 | 6.04740668329224 | 9.06834313847462 | 8.04681434710033 | 9.58373164713426 | 28.2830794466509 | 21.851519466489 |
| YBR183W | 2729.10391340478 | 1.13731475147864 | 0.412899301054483 | 2.75446034559544 | 0.00587890135803216 | 0.0152970200992837 | 1892.83829187047 | 1793.05875692566 | 1430.86989662984 | 2043.94858583427 | 4745.27221827142 | 4468.635730897 |
| YBR184W | 74.1902501244584 | -1.34554422365648 | 0.414742440217922 | -3.24428872760038 | 0.00117744328693221 | 0.0040592798543059 | 125.483688678314 | 111.293302154007 | 81.9312006250216 | 33.9786849307487 | 54.9948767018211 | 37.4597476568383 |
| YBR185C | 920.561808985094 | 0.953004034173468 | 0.328324984654348 | 2.90262416421572 | 0.00370050436291756 | 0.0103659191375333 | 591.134003291816 | 562.237274585426 | 727.139405547066 | 927.879473108908 | 1279.02370386521 | 1435.95699351214 |
| YBR186W | 26.3669788903666 | -0.168649376511754 | 0.483521319911985 | -0.348794085320692 | 0.727243904131494 | 0.791123436251153 | 24.1896267331689 | 31.3270035692759 | 27.7980859263466 | 20.0387116258262 | 32.9969260210927 | 21.851519466489 |
| YBR187W | 11087.7378922834 | 1.82726487096131 | 0.435159429421579 | 4.19906992108649 | 2.68013558556632e-05 | 0.000197936618934026 | 5499.36045261888 | 5532.51370930029 | 3593.2683702688 | 9135.91000471362 | 21396.1496013914 | 21369.2252154072 |
| YBR188C | 117.451003000137 | -2.04149316895872 | 0.640888678288237 | -3.18540994422212 | 0.00144549085168955 | 0.00476893498200855 | 193.517013865352 | 209.396287015687 | 166.05698698107 | 94.9660681397849 | 28.2830794466509 | 12.4865825522794 |
| YBR189W | 91862.080234441 | 0.372374513783841 | 0.330260403894923 | 1.12751789010201 | 0.25952358090977 | 0.350384037957844 | 90835.8280122264 | 92892.8095312109 | 56487.9051880673 | 97580.6843827894 | 103616.632834997 | 109758.621457355 |
| YBR190W | 6.91772094868601 | -0.78138807753723 | 0.900017205641103 | -0.868192377478638 | 0.385289026408881 | 0.483393985987375 | 6.04740668329224 | 16.4878966154084 | 3.6576428850456 | 4.3562416577883 | 4.71384657444181 | 6.24329127613972 |
| YBR191W | 63135.6227794664 | 0.58531184797499 | 0.334793313139727 | 1.74827819135894 | 0.0804158677114023 | 0.134095853977584 | 55264.9819011015 | 55322.6639085105 | 40915.1248406971 | 57081.5769386632 | 79693.8614697047 | 90535.5276181211 |
| YBR191W-A | 4665.80946515556 | 0.376448798570309 | 0.394148169258589 | 0.955094626669019 | 0.339529846042194 | 0.435572153089986 | 4583.93426593551 | 4334.66802019087 | 3261.88592488367 | 3161.76019522275 | 5948.87437694557 | 6703.73400775502 |
| YBR192W | 601.735749826275 | -0.391969387761902 | 0.284492211559202 | -1.37778600550664 | 0.168269368026233 | 0.246229204746407 | 676.553622693319 | 671.881787077892 | 700.804376774738 | 543.65895889198 | 447.815424571972 | 569.700328947749 |
| YBR193C | 389.901594860314 | -0.693650656063229 | 0.456448538533792 | -1.5196689166568 | 0.128594207513817 | 0.197442917166815 | 456.579204588564 | 451.76836726219 | 539.136561255722 | 495.740300656308 | 194.838991743595 | 201.346143655506 |
| YBR194W | 117.716902327102 | -1.44286066148298 | 0.369289264365258 | -3.90712864064165 | 9.33994334481446e-05 | 0.000531823476044254 | 181.422200498767 | 184.664442092574 | 151.426415440888 | 77.5411015086317 | 64.4225698507047 | 46.8246845710479 |
| YBR195C | 530.606666980458 | 0.0460085899958839 | 0.420087824194997 | 0.109521360406122 | 0.912788980521462 | 0.9376871964521 | 461.114759601033 | 473.202632862221 | 633.503747689899 | 825.072169985104 | 380.250290338306 | 410.496401406187 |
| YBR196C | 113099.733627031 | 1.78534154928088 | 0.366259958019222 | 4.87452016031516 | 1.09073246812385e-06 | 1.66059505785286e-05 | 47063.1865868864 | 49865.9945236411 | 55667.1301246631 | 109936.72822094 | 205628.986552492 | 210436.375753565 |
| YBR196C-A | 94.9188988557421 | 1.32416347267354 | 0.405780408034719 | 3.26325137057934 | 0.00110141779712124 | 0.00385077666311662 | 40.8199951122226 | 63.4784019693223 | 57.7907575837206 | 95.8373164713426 | 147.700525999177 | 163.886395998668 |
| YBR196C-B | 50.81813290694 | 1.34273265364923 | 0.498448528806435 | 2.69382408824535 | 0.0070637434952788 | 0.0178184520601627 | 33.2607367581073 | 31.3270035692759 | 21.2143287332645 | 43.562416577883 | 109.989753403642 | 65.554558399467 |
| YBR197C | 314.99878880784 | -0.0730281921485537 | 0.502657260308777 | -0.145284268059101 | 0.884486435206842 | 0.916212424438164 | 272.889226583562 | 314.9188253543 | 382.58944577577 | 543.65895889198 | 201.124120509517 | 174.812155731912 |
| YBR198C | 1512.88660128927 | 0.398044953060783 | 0.321884409476669 | 1.23660836418868 | 0.216232513186332 | 0.302725518460865 | 1522.43463251882 | 1403.94439680202 | 989.758164693341 | 1533.39706354148 | 1706.41245994794 | 1921.372890232 |
| YBR199W | 2911.19061803256 | 0.159061705410963 | 0.259888185833468 | 0.612039000160196 | 0.540511954570619 | 0.631512515862625 | 2617.77116803013 | 2858.17687828104 | 2776.88247832662 | 3159.14645022807 | 2924.1561583454 | 3131.01057498407 |
| YBR200W | 516.571570251785 | -1.33555607889555 | 0.699788281028078 | -1.90851449660382 | 0.0563247513251472 | 0.0998709797725628 | 751.39028039906 | 759.267639139556 | 711.045776852866 | 680.444946946532 | 92.7056492973556 | 104.57512887534 |
| YBR201C-A | 375.971192727876 | 0.13956084759124 | 0.438493197001961 | 0.318273689410546 | 0.750277340253203 | 0.80888586535676 | 379.474769376588 | 403.129072246735 | 289.685316495612 | 203.000861252935 | 520.09440538008 | 460.442731615304 |
| YBR201W | 645.61181433754 | 0.205062605273417 | 0.271361317534558 | 0.755681049666563 | 0.449840448596539 | 0.546701734093548 | 627.41844339157 | 608.40338510857 | 563.277004297023 | 652.565000336687 | 718.075961506636 | 703.931091384753 |
| YBR202W | 1092.91921568766 | -0.55705950515122 | 0.589180470234795 | -0.945481958913582 | 0.344412877077476 | 0.440607653631098 | 1306.23984359112 | 1393.22726400201 | 1206.29062348804 | 1812.19652963993 | 444.672860189011 | 394.888173215837 |
| YBR203W | 544.921022749532 | -0.48890466678608 | 0.300144224686345 | -1.62889913106605 | 0.103334374086683 | 0.164843539538826 | 592.645854962639 | 727.11624073951 | 590.343561646361 | 518.392757276808 | 430.531320465685 | 410.496401406187 |
| YBR204C | 508.770060352258 | 0.873821358325044 | 0.341921505198453 | 2.55561976956633 | 0.0105998875404188 | 0.0250140255625859 | 304.638111670846 | 304.201692554285 | 468.909817862847 | 699.612410240801 | 713.362114932194 | 561.896214852575 |
| YBR205W | 2138.93559158537 | 0.68576176965693 | 0.370320975278358 | 1.8518037471182 | 0.0640540093881261 | 0.110919015988043 | 1511.09574498765 | 1684.23863926397 | 1725.67591316452 | 3792.54398727049 | 2033.2391557759 | 2086.8201090497 |
| YBR206W | 261.346738642708 | 1.00871454286321 | 0.365079332124046 | 2.76300095377754 | 0.00572725991001719 | 0.0149872221944375 | 133.798872867841 | 146.742279877135 | 239.941373258992 | 353.72682261241 | 364.5374684235 | 329.33361481637 |
| YBR207W | 2289.47484625472 | 0.76179121830035 | 0.304273664629124 | 2.50363835867587 | 0.0122923607718323 | 0.0282623360837557 | 1612.38980693279 | 1851.59078991036 | 1631.30872673034 | 2274.82939369705 | 3302.83516649223 | 3063.89519376557 |
| YBR208C | 4188.68266969141 | 1.26863524454732 | 0.47234917401486 | 2.68579964640185 | 0.00723564623127412 | 0.0181737760255091 | 1588.95610603503 | 1618.28705280233 | 4163.86066033592 | 4628.07113723429 | 6120.14413581695 | 7012.77692592394 |
| YBR210W | 1209.88033167968 | 1.00630936492265 | 0.385738974344066 | 2.60878322350973 | 0.00908647836468061 | 0.0220236810946719 | 845.12508399009 | 862.316992985859 | 704.462019659784 | 972.313138018348 | 1806.97452020269 | 2068.09023522128 |
| YBR211C | 331.741558086899 | -0.985255902501203 | 0.409854212799258 | -2.4039179584663 | 0.0162204130718735 | 0.0356882337488435 | 464.894388778091 | 492.16371396994 | 367.227345658579 | 329.331869328795 | 188.553862977672 | 148.278167808318 |
| YBR212W | 1054.19597866009 | -2.0528210478206 | 0.678879214085075 | -3.02383841665735 | 0.0024958970579606 | 0.0074760823503564 | 1598.02721605997 | 1569.64775778688 | 1931.23544330408 | 927.00822477735 | 171.269758871386 | 127.987471160864 |
| YBR213W | 328.975879255471 | 0.77881910908718 | 0.505337714536589 | 1.54118540271902 | 0.123271669143354 | 0.191155682466458 | 151.185167082306 | 179.718073107951 | 396.488488738944 | 618.586315405938 | 298.543616381315 | 329.33361481637 |
| YBR214W | 1781.93635217995 | 0.634119880903556 | 0.565259871141954 | 1.12182009245144 | 0.261938941427756 | 0.352610113460441 | 801.281385536221 | 821.097251447338 | 2567.66530530201 | 3356.91982149166 | 1580.70988462949 | 1563.944464673 |
| YBR215W | 73.4298356054828 | -0.49596944741489 | 0.396798808922668 | -1.24992675447156 | 0.211326305016151 | 0.297083912749185 | 90.7111002493835 | 103.049353846302 | 63.6429861997935 | 53.1461482250172 | 72.2789808081078 | 57.7504443042924 |
| YBR216C | 1451.17114301341 | 1.0424648487397 | 0.337670511487468 | 3.08722501158763 | 0.00202034602669556 | 0.00629463903558149 | 895.016189127251 | 891.995206893594 | 1057.79032235519 | 1380.05735718733 | 2267.36020230651 | 2214.80758021057 |
| YBR217W | 81.8031259680138 | -2.11644833754183 | 0.412111437397567 | -5.13562144964221 | 2.81213203663019e-07 | 6.25412977010055e-06 | 129.263317855372 | 134.376357415578 | 136.795843900706 | 41.8199199147677 | 26.7117972551703 | 21.851519466489 |
| YBR218C | 10630.3118873955 | 0.764819861773439 | 0.30056091426664 | 2.5446417863067 | 0.0109389891558905 | 0.025673137814845 | 6869.09806638457 | 7408.01194930299 | 9353.32438563862 | 14129.0341928706 | 11544.210260808 | 14478.192469368 |
| YBR220C | 1713.32609737425 | 0.809988980790376 | 0.285518647929077 | 2.83690395238765 | 0.00455533132853512 | 0.0123625510980894 | 1210.23726249386 | 1318.2073344019 | 1204.82756633402 | 1873.18391284897 | 2169.94070643471 | 2503.55980173203 |
| YBR221C | 21342.4741835297 | 1.53520522259035 | 0.361009542913134 | 4.25253363166565 | 2.11365461079872e-05 | 0.000163212658195968 | 10438.5798611978 | 11059.2566547852 | 11350.3974008735 | 20041.3253708208 | 37393.3735928554 | 37771.9122206453 |
| YBR221W-A | 1.3458760132515 | 0.817407056376001 | 1.86660943647045 | 0.437910063243666 | 0.661451480402988 | 0.738242800657686 | 0 | 0.82439483077042 | 2.19458573102736 | 3.48499332623064 | 1.5712821914806 | 0 |
| YBR222C | 3515.42226009342 | 0.755340215880515 | 0.264729934901467 | 2.85324822129259 | 0.00432748048268874 | 0.0118088874188625 | 2489.26377601017 | 2626.52193083456 | 2730.79617797505 | 4197.6744614448 | 4248.74704576355 | 4799.53016853241 |
| YBR223C | 377.202566247664 | 0.518392409681649 | 0.396486974076467 | 1.30746391073537 | 0.191055203978423 | 0.274152298043905 | 227.53367645887 | 247.318449231126 | 455.742303476682 | 491.38405899852 | 461.956964295297 | 379.279945025488 |
| YBR224W | 1.54100661219151 | -0.440376482720528 | 1.68833249914065 | -0.260835163064549 | 0.794219626600588 | 0.8438829228358 | 3.77962917705765 | 0 | 1.46305715401824 | 0.87124833155766 | 1.5712821914806 | 1.56082281903493 |
| YBR225W | 384.099601392908 | -0.916680723109129 | 0.400512412515701 | -2.28876982201692 | 0.0220927297554711 | 0.0461639129218799 | 443.728465386568 | 448.470787939108 | 615.94706184168 | 378.121775896024 | 238.834893105052 | 179.494624189017 |
| YBR226C | 3.71815971281293 | 0.128357765476397 | 1.01850289204266 | 0.1260259214571 | 0.899711405805008 | 0.927210986299288 | 3.77962917705765 | 4.1219741538521 | 2.92611430803648 | 5.22748998934596 | 1.5712821914806 | 4.68246845710479 |
| YBR227C | 358.13092596504 | -1.20445531675507 | 0.484206433151386 | -2.48748309458849 | 0.0128650567815721 | 0.0293797750614624 | 482.280682992556 | 455.065946585272 | 563.277004297023 | 377.250527564467 | 133.558986275851 | 137.352408075074 |
| YBR228W | 253.083767103947 | 0.27359099939473 | 0.400265901295796 | 0.683523124275697 | 0.494276343245678 | 0.588271973849966 | 250.211451521216 | 248.967238892667 | 187.271315714335 | 168.150927990628 | 317.399002679082 | 346.502665825754 |
| YBR229C | 1320.57060303403 | -0.374420331196429 | 0.416120302135068 | -0.899788665141591 | 0.368232727566194 | 0.465897596370588 | 1433.23538394026 | 1419.60789858666 | 1621.79885522922 | 1783.44533469853 | 928.627775165037 | 736.708370584487 |
| YBR230C | 1507.67413232903 | 0.791367906926559 | 0.351327700412364 | 2.25250643771529 | 0.0242902863534712 | 0.0499455440984529 | 1090.80098049884 | 993.395771078356 | 1227.5049522213 | 1288.57628237378 | 2191.93865711544 | 2253.82815068644 |
| YBR230W-A | 463.468189036914 | 0.649832178470453 | 0.289933417120232 | 2.24131521273029 | 0.02500566423818 | 0.0510560908241095 | 410.46772862846 | 314.9188253543 | 356.985945580451 | 566.311415512479 | 548.377484826731 | 583.747734319064 |
| YBR231C | 176.035450234054 | 0.223984186053696 | 0.354064598904155 | 0.632608249305174 | 0.526989498724822 | 0.619421860154746 | 162.524054613479 | 150.039859200216 | 175.566858482189 | 242.207036173029 | 183.840016403231 | 142.034876532179 |
| YBR233W | 125.791130835231 | -3.07520258183487 | 0.800340739004603 | -3.84236667204964 | 0.000121853610705515 | 0.000652575338723298 | 210.147382244405 | 219.289024984932 | 247.256659029083 | 67.0861215299398 | 6.28512876592241 | 4.68246845710479 |
| YBR233W-A | 202.255087821836 | -0.801729857206686 | 0.326640117684461 | -2.45447455410596 | 0.0141090649626988 | 0.031803422597053 | 275.157004089797 | 292.660164923499 | 203.364944408536 | 146.369719701687 | 147.700525999177 | 148.278167808318 |
| YBR234C | 8351.38261601819 | 1.41254817828215 | 0.375454700371892 | 3.76223330506451 | 0.000168402842979298 | 0.00084463731213916 | 4490.19946234449 | 4718.83601132988 | 4473.29724841077 | 7285.37854848515 | 14306.5243534309 | 14834.060072108 |
| YBR235W | 1027.59319662037 | -0.159735730987703 | 0.42468588759434 | -0.376126769581482 | 0.706822653831462 | 0.775061789660244 | 938.85988758112 | 1018.95201083224 | 1297.00016703717 | 1512.4871035841 | 667.794931379257 | 730.465079308347 |
| YBR236C | 1072.10463830668 | -0.508022010382559 | 0.330328493976045 | -1.53792972646011 | 0.124065798049408 | 0.191985898786668 | 1326.64984114723 | 1385.80771052508 | 1065.83713670229 | 1127.39534103561 | 771.499556016976 | 755.438244412906 |
| YBR237W | 320.603288131262 | -1.9730094820885 | 0.517039120560413 | -3.81597717393217 | 0.000135645044366226 | 0.000711363261985743 | 598.693261645931 | 541.627403816166 | 395.025431584925 | 231.752056194337 | 65.9938520421854 | 90.5277235040259 |
| YBR238C | 1485.68730088682 | 0.119131980619227 | 0.331765605300075 | 0.359084783702863 | 0.719531664390568 | 0.78485499977562 | 1251.81318344149 | 1307.49020160189 | 1714.70298450938 | 1892.35137614324 | 1514.7160325873 | 1233.05002703759 |
| YBR239C | 387.819481565263 | -0.79237893164677 | 0.291171943102058 | -2.72134369542959 | 0.00650171240956946 | 0.0166154872688997 | 458.091056259387 | 506.178426093038 | 511.338475329376 | 301.45192271895 | 301.686180764276 | 248.170828226554 |
| YBR240C | 273.611006084154 | 1.02175549385919 | 0.342949799035534 | 2.97931503891427 | 0.00288893584243517 | 0.00843081691710841 | 185.201829675825 | 177.24488861564 | 178.492972790226 | 261.374499467298 | 413.247216359399 | 426.104629596536 |
| YBR241C | 1588.48199488273 | 2.13068981844064 | 0.485740803602726 | 4.38647484962633 | 1.15202478575849e-05 | 0.000102190628378577 | 471.697721296794 | 483.919765662236 | 815.65436336517 | 1209.29268420203 | 3684.65673902202 | 2865.67069574813 |
| YBR242W | 1120.18874969048 | -1.86100285437698 | 0.615065115561893 | -3.02570054339183 | 0.002480578437067 | 0.00744057994164486 | 1954.0682845388 | 1976.0744093567 | 1342.35493881174 | 1013.26180960156 | 219.979506807285 | 215.39354902682 |
| YBR243C | 3044.32040068604 | 1.31823092088622 | 0.385480086632203 | 3.41971211120946 | 0.000626874393969306 | 0.00243784486543619 | 1746.18867980063 | 1806.24907421799 | 1675.20044135089 | 2524.00641652254 | 5244.93995516226 | 5269.33783706192 |
| YBR244W | 285.577295952174 | 0.43000703154155 | 0.521970678616155 | 0.823814534336645 | 0.410044947862257 | 0.508118041992099 | 182.93405216959 | 159.932597169461 | 386.247088660816 | 162.052189669725 | 430.531320465685 | 391.766527577767 |
| YBR245C | 668.856476324426 | -2.4426991684771 | 0.363046267888178 | -6.72834121856192 | 1.71608121077766e-11 | 5.81661210389902e-09 | 1279.02651351631 | 1321.50491372498 | 790.050863169851 | 238.722042846799 | 215.265660232843 | 168.568864455772 |
| YBR246W | 3422.26470365224 | 1.79396002098391 | 0.387118051553584 | 4.63414199824674 | 3.58420916518738e-06 | 4.25088527141928e-05 | 1285.0739201996 | 1384.9833156943 | 1925.38321468801 | 3376.95853311749 | 6049.43643720032 | 6511.75280101373 |
| YBR247C | 3153.31103335914 | 0.368058611016328 | 0.271197179583815 | 1.35716238487863 | 0.17472961972032 | 0.254008747403806 | 2845.304844489 | 2970.29457526582 | 2444.03697578747 | 3441.43090965276 | 3750.6505910642 | 3468.14830389561 |
| YBR248C | 2343.52985721637 | 0.239354451130548 | 0.351580302716395 | 0.680795964054976 | 0.496000604181083 | 0.589997024552304 | 2094.67048992535 | 2272.03215360328 | 2083.39338732198 | 3498.062051204 | 1913.82170922338 | 2199.19935202022 |
| YBR249C | 29298.9046776494 | 0.828617505137308 | 0.318120390411199 | 2.60472931039173 | 0.00919468702396976 | 0.022202393863654 | 20564.2064265352 | 21845.6386205853 | 20915.8650738448 | 27737.0618834697 | 39601.0250718857 | 45129.630989576 |
| YBR250W | 60.3798990326098 | -0.182550845371134 | 0.382696630640683 | -0.47701189599062 | 0.633353650687681 | 0.714200229456954 | 66.5214735162146 | 51.9368743385364 | 73.8843862779212 | 54.0173965565749 | 62.8512876592241 | 53.0679758471876 |
| YBR251W | 361.641192202473 | -0.518901343947126 | 0.582612709015646 | -0.890645425198218 | 0.373119421456949 | 0.471247121824426 | 417.271061147164 | 434.456075816011 | 427.944217550336 | 602.032597106343 | 142.986679424735 | 145.156522170248 |
| YBR252W | 4041.84760317302 | 1.62406757164477 | 0.357714778875371 | 4.54011874139144 | 5.62225564111041e-06 | 5.92591265609673e-05 | 2061.40975316724 | 2030.48446818754 | 1847.84118552504 | 3948.49743861931 | 6824.07855760026 | 7538.77421593871 |
| YBR253W | 490.68110201262 | -0.280421518953083 | 0.303249801192263 | -0.924721196355518 | 0.35511095082133 | 0.451602394014487 | 564.676599052412 | 568.008038400819 | 482.80886082602 | 503.581535640327 | 369.251314997942 | 455.7602631582 |
| YBR254C | 643.174258258633 | 0.624269975897154 | 0.322092097022016 | 1.9381722857189 | 0.0526022067545289 | 0.0944932249649 | 509.494013067371 | 499.583267446874 | 508.412361021339 | 576.766395491171 | 862.633923122851 | 902.15558940219 |
| YBR255C-A | 457.952101092565 | 1.08173508583804 | 0.447842953724351 | 2.41543397488365 | 0.0157164715909589 | 0.0347218103072985 | 336.386996758131 | 314.09443052353 | 230.431501757873 | 310.164406034527 | 777.784684782899 | 778.85058669843 |
| YBR255W | 164.345442500196 | -1.75960149931877 | 0.543055419287056 | -3.24018771717414 | 0.00119451033576142 | 0.00410493413143198 | 371.159585187061 | 295.957744246581 | 93.6356578571675 | 59.2448865459209 | 94.2769314888362 | 71.7978496756068 |
| YBR256C | 2657.67161272292 | -0.0604564490825321 | 0.294663007108229 | -0.20517149293982 | 0.837438140988503 | 0.878784687626847 | 2830.18632778077 | 2788.10331766556 | 2520.84747637343 | 2167.66584891546 | 2575.33151183671 | 3063.89519376557 |
| YBR257W | 226.68666696669 | -1.8628177926454 | 0.317454093391743 | -5.86799109358672 | 4.41106941310359e-09 | 2.92858629076156e-07 | 387.789953566115 | 370.977673846689 | 308.705059497849 | 106.292296450034 | 92.7056492973556 | 93.6493691420958 |
| YBR258C | 65.8670979286471 | -0.915068474138518 | 0.405871658999495 | -2.25457593268333 | 0.0241599595305365 | 0.0497410931511046 | 92.9788777556181 | 64.3027968000927 | 100.21941505025 | 37.4636782569794 | 51.8523123188599 | 48.3855073900828 |
| YBR259W | 407.588358281602 | -0.487558418102904 | 0.284973372712037 | -1.71089113857517 | 0.087101210760999 | 0.142985418633911 | 437.681058703276 | 469.905053539139 | 520.116818253485 | 353.72682261241 | 347.253364317213 | 316.847032264091 |
| YBR260C | 613.982400779919 | -0.223652986507431 | 0.402452872589387 | -0.555724661793181 | 0.57839909470095 | 0.665753381568207 | 492.107718852906 | 573.778802216212 | 919.531421300465 | 752.758558465818 | 447.815424571972 | 497.902479272143 |
| YBR261C | 1209.9792026676 | 0.25532031763915 | 0.535863341006735 | 0.476465356184798 | 0.633742882825767 | 0.714379271476048 | 1056.02839206991 | 1015.65443150916 | 1239.20940945345 | 2469.98901996596 | 603.372361528552 | 875.621601478596 |
| YBR262C | 1154.41690540819 | 1.34023179718104 | 0.313099360890846 | 4.28053188408864 | 1.86447171735723e-05 | 0.000148603933908175 | 692.428065236961 | 652.096311139402 | 615.94706184168 | 1265.92382575328 | 1784.97656952197 | 1915.12959895586 |
| YBR263W | 7952.14490022237 | 0.646271249974576 | 0.435782446212172 | 1.48301349811581 | 0.138070824530757 | 0.209958939782308 | 5341.37195301787 | 5441.00588308477 | 7819.3089596505 | 15031.6474643643 | 5999.15540707295 | 8080.37973414383 |
| YBR264C | 392.768608599849 | -0.307889290124104 | 0.317275220139566 | -0.97041707193101 | 0.331838642463162 | 0.427708428768125 | 496.643273865375 | 469.080658708369 | 337.966202578214 | 354.598070943967 | 329.969260210927 | 368.354185292243 |
| YBR265W | 3936.55916326879 | 1.94509436426913 | 0.395545511635068 | 4.91749825760552 | 8.7657236902494e-07 | 1.39730842983184e-05 | 1406.02205386544 | 1445.16413834055 | 2017.55581539116 | 3779.47526229713 | 7462.01912734139 | 7509.11858237705 |
| YBR267W | 1266.13299331184 | -2.04472219055757 | 0.554996914592484 | -3.68420460870298 | 0.000229417904147975 | 0.00107312002683852 | 2766.6885576062 | 2563.867923696 | 785.661691707796 | 772.797270091644 | 344.110799934252 | 363.671716835139 |
| YBR268W | 825.115597569764 | 1.24523455998188 | 0.324746861208117 | 3.83447758463125 | 0.000125831446048197 | 0.000667507835708723 | 486.060312169613 | 469.905053539139 | 512.070003906385 | 850.338371600276 | 1288.4513970141 | 1343.86844718907 |
| YBR269C | 852.225490457506 | 1.35580978202858 | 0.431444226615193 | 3.14249142389806 | 0.00167516620442963 | 0.00540213838584216 | 390.813656907761 | 361.084935877444 | 683.979219503528 | 763.21353844451 | 1437.72320520475 | 1476.53838680704 |
| YBR270C | 190.242215870698 | -0.155422511225574 | 0.390923466010901 | -0.397577850241509 | 0.690941390403936 | 0.762050909479956 | 187.469607182059 | 171.474124800247 | 243.599016144037 | 243.078284504587 | 125.702575318448 | 170.129687274807 |
| YBR271W | 749.140700267683 | -0.787598019779959 | 0.529821467728255 | -1.48653474378263 | 0.137137733967226 | 0.208879644285276 | 901.063595810543 | 996.693350401437 | 950.255621534848 | 1035.91426622206 | 329.969260210927 | 280.948107426287 |
| YBR272C | 1013.34966872603 | 0.848132434003657 | 0.320459257791327 | 2.64661548506716 | 0.00813017395001522 | 0.0200683481173239 | 761.21731625941 | 737.008978708755 | 672.274762271382 | 971.44188968679 | 1510.00218601286 | 1428.15287941696 |
| YBR273C | 574.03262663887 | -1.47265545255516 | 0.540386873483934 | -2.72518731452671 | 0.00642650161492044 | 0.0164683601203857 | 884.43322743149 | 838.409542893517 | 811.265191903115 | 581.122637148959 | 179.126169828789 | 149.838990627353 |
| YBR274W | 1328.54289813468 | 1.20137182898094 | 0.37721078100675 | 3.18488200622093 | 0.00144813040622582 | 0.00477519703845073 | 730.224357007537 | 786.47266855498 | 898.317092567201 | 1136.97907268275 | 2196.65250368988 | 2222.61169430574 |
| YBR275C | 196.136799966978 | -1.69509609027274 | 0.465305635848011 | -3.64297347738645 | 0.000269506617327017 | 0.00122485717401975 | 406.688099451403 | 348.719013415887 | 144.111129670797 | 101.936054792246 | 83.277956148472 | 92.0885463230609 |
| YBR276C | 2143.75166718372 | 0.252459563341717 | 0.272711356507269 | 0.925739091232119 | 0.354581587418017 | 0.451107353412096 | 1792.30015576074 | 1887.86416246426 | 2189.4650309883 | 2214.71325881957 | 2418.20329268865 | 2359.96410238081 |
| YBR277C | 7.09829448340924 | 1.69479356338394 | 0.861284295937426 | 1.96775161393058 | 0.0490966239458527 | 0.0893675122134797 | 4.53555501246918 | 1.64878966154084 | 3.6576428850456 | 6.09873832090362 | 17.2841041062866 | 9.36493691420958 |
| YBR278W | 185.053870803651 | -2.25311297354037 | 0.517102733663931 | -4.35718635168671 | 1.31745093618402e-05 | 0.000113731689397119 | 337.898848428954 | 330.582327138938 | 251.645830491138 | 115.004779765611 | 40.8533369784957 | 34.3381020187685 |
| YBR279W | 27.4784072087654 | -3.24622846358792 | 0.853368796971941 | -3.80401588985525 | 0.000142369041699638 | 0.000740595014980345 | 68.0333251870376 | 70.8979554462561 | 10.9729286551368 | 8.7124833155766 | 1.5712821914806 | 4.68246845710479 |
| YBR280C | 738.638634621727 | 1.41471601048711 | 0.35220267037638 | 4.01676684897157 | 5.90020458120967e-05 | 0.0003717937133365 | 312.197370024962 | 352.016592738969 | 544.257261294786 | 940.076949750715 | 1142.3221532064 | 1140.96148071453 |
| YBR281C | 1121.31821835698 | -0.227999318619007 | 0.394319154851213 | -0.578210101675219 | 0.563122279538792 | 0.652954173609978 | 1067.36727960108 | 1229.1726926787 | 1334.30812446464 | 1538.62455353083 | 813.924175186953 | 744.512484679662 |
| YBR282W | 300.705613037543 | -0.572214653974459 | 0.29559425071848 | -1.93581117556792 | 0.052890831675733 | 0.0949058110871331 | 385.52217605988 | 315.743220185071 | 377.468745736706 | 244.820781167702 | 226.264635573207 | 254.414119502694 |
| YBR283C | 19295.9259249576 | 1.64434707119278 | 0.388735067431699 | 4.22999417587066 | 2.33697362272793e-05 | 0.000178579384435237 | 8848.11190349195 | 9768.2543497987 | 9442.57087203373 | 16749.749174196 | 35547.1170178657 | 35419.7522323597 |
| YBR284W | 174.315018254631 | 0.569728323051681 | 0.381493447610069 | 1.49341574965662 | 0.13532839280504 | 0.206541710373614 | 130.775169526195 | 103.049353846302 | 186.539787137326 | 169.893424653744 | 215.265660232843 | 240.366714131379 |
| YBR285W | 20.5392291069695 | -0.806045832217779 | 0.646673126824932 | -1.24645017518409 | 0.212599170383033 | 0.298742888340985 | 14.3625908728191 | 17.3122914461788 | 46.8178289285837 | 16.5537182995955 | 14.1415397233254 | 14.0474053713144 |
| YBR286W | 40568.2044392651 | 0.988217956292264 | 0.296314078373403 | 3.33503545196712 | 0.000852885251495699 | 0.0031225588514112 | 23755.7253036427 | 24677.4348642817 | 33145.5598242833 | 54914.7823380793 | 50551.290664314 | 56364.4336409894 |
| YBR287W | 14419.7636004776 | 1.45606740592933 | 0.397590544086424 | 3.6622284598721 | 0.000250030723094519 | 0.00115014132623479 | 7717.2468537163 | 8361.0123736736 | 7032.18421078868 | 11895.1534707567 | 27137.6147290615 | 24375.3699648685 |
| YBR288C | 1258.72928727427 | -0.0958516006735549 | 0.263464741019701 | -0.363811872141128 | 0.715998496627413 | 0.781795577870556 | 1238.9624442395 | 1319.85612406344 | 1343.08646738875 | 1270.28006741107 | 1162.74882169565 | 1217.44179884725 |
| YBR289W | 304.060092743101 | -2.0648848525039 | 0.834840183706328 | -2.47338938973529 | 0.0133838227365306 | 0.0303920375258312 | 541.998823990067 | 508.651610585349 | 423.555046088281 | 303.194419382066 | 20.4266684892478 | 26.5339879235938 |
| YBR290W | 340.325323003806 | -2.34292879368431 | 0.401045230174984 | -5.84205625051828 | 5.1560389042239e-09 | 3.16942140632647e-07 | 722.665098653422 | 535.032245170002 | 449.890074860609 | 154.210954685706 | 98.990778063278 | 81.1627865898164 |
| YBR291C | 1441.52018629205 | 0.826591817062242 | 0.408301091295967 | 2.02446634281236 | 0.0429221916321188 | 0.0800379680652428 | 1180.0002290774 | 1207.73842707866 | 730.065519855103 | 1143.94905933521 | 2094.51916124364 | 2292.84872116231 |
| YBR292C | 83.3036492181121 | 0.710799032442597 | 0.48085408826743 | 1.47820107967405 | 0.139353965017322 | 0.211660267620649 | 46.1114759601033 | 58.5320329846998 | 84.1257863560489 | 55.7598932196902 | 131.987704084371 | 123.305002703759 |
| YBR293W | 1223.39838705732 | 1.20098570438911 | 0.379360735412019 | 3.1658144670263 | 0.00154649399203376 | 0.00505297884763948 | 715.861766134718 | 812.028908308863 | 696.415205312683 | 1031.55802456427 | 2088.23403247772 | 1996.29238554568 |
| YBR294W | 43.4274884116031 | 0.415834813196989 | 0.420004563419126 | 0.990072131149736 | 0.322138863966495 | 0.417587416252864 | 33.2607367581073 | 34.6245828923576 | 43.1601860435381 | 38.334926588537 | 54.9948767018211 | 56.1896214852575 |
| YBR295W | 2040.97671486222 | 0.363511415644382 | 0.3668610123555 | 0.990869575674965 | 0.321749270164689 | 0.417166357934487 | 1561.74277596022 | 1764.2049378487 | 2028.52874404629 | 1495.06213695294 | 2897.44436109023 | 2498.87733327492 |
| YBR296C | 272.185808464742 | 1.38554894545429 | 0.433241838291659 | 3.19809589701153 | 0.00138338268558628 | 0.00461362245821822 | 139.846279551133 | 188.786416246426 | 122.896800937532 | 221.297076215646 | 526.379534146002 | 433.90874369171 |
| YBR296C-A | 1.94752131373695 | 1.46840929597997 | 1.6041507526325 | 0.915381109643361 | 0.359991591820169 | 0.456816916516629 | 2.26777750623459 | 0 | 0.731528577009121 | 0.87124833155766 | 1.5712821914806 | 6.24329127613972 |
| YBR297W | 467.818362948559 | 1.27505079528037 | 0.396099318980169 | 3.21901789319716 | 0.00128630458425306 | 0.00436679047052698 | 278.180707431443 | 303.377297723514 | 238.478316104973 | 389.448004206274 | 809.210328612511 | 788.21552361264 |
| YBR298C | 583.784821392471 | 0.92260842145835 | 0.438218947562306 | 2.10535949344631 | 0.0352600269730695 | 0.0678644870611379 | 445.240317057391 | 501.232057108415 | 262.618759146274 | 448.692890752195 | 942.769314888362 | 902.15558940219 |
| YBR299W | 27.7580941034594 | 0.585745009191039 | 0.516566157834463 | 1.13392060301934 | 0.256827853955809 | 0.347342726421005 | 18.8981458852882 | 21.4342656000309 | 25.6035001953192 | 19.1674632942685 | 42.4246191699763 | 39.0205704758732 |
| YBR300C | 16.3884977158574 | 2.63017271543305 | 0.825291868702175 | 3.1869606561969 | 0.00143776331128479 | 0.00475318055681421 | 2.26777750623459 | 1.64878966154084 | 9.50987150111857 | 11.3262283102496 | 32.9969260210927 | 40.5813932949082 |
| YBR301W | 51.5004640224557 | 1.60536640092316 | 0.531646078350944 | 3.01961486465334 | 0.00253096300554923 | 0.00754601933135975 | 17.3862942144652 | 19.7854759384901 | 38.7710145814834 | 43.562416577883 | 95.8482136803168 | 93.6493691420958 |
| YBR302C | 89.2027667809175 | 1.0793702648877 | 0.500175483174972 | 2.1579831503059 | 0.030929141863975 | 0.0607452496505029 | 59.7181409975108 | 54.4100588308477 | 57.0592290067114 | 52.2748998934596 | 172.841041062866 | 138.913230894109 |
| YCL001W | 3325.79604087309 | 0.835038867020439 | 0.390915684853066 | 2.13610990649891 | 0.0326704564793998 | 0.0636027024568726 | 2227.71343695778 | 2285.2224708956 | 2653.98567738909 | 2463.89028164506 | 5026.53173054645 | 5297.43264780455 |
| YCL001W-A | 3.2365932622899 | -3.75065149770609 | 1.60322715565498 | -2.3394386032426 | 0.0193127456204913 | 0.0412107626891862 | 10.5829616957614 | 5.77076381539294 | 2.19458573102736 | 0.87124833155766 | 0 | 0 |
| YCL001W-B | 6.69526224952432 | -2.51942118621625 | 1.06361419804574 | -2.36873594847209 | 0.0178489914009684 | 0.0385471175795562 | 14.3625908728191 | 15.663501784638 | 4.38917146205473 | 2.61374499467298 | 3.14256438296121 | 0 |
| YCL002C | 2270.66803023864 | 1.41997883606953 | 0.42588060700029 | 3.33421811824496 | 0.00085539551697245 | 0.00312870181262118 | 1313.04317610983 | 1460.82764012518 | 931.96740710962 | 1807.84028798214 | 4030.33882114775 | 4079.99084895731 |
| YCL004W | 347.542782568322 | -0.404818305525514 | 0.613373818445421 | -0.659986281370005 | 0.509262633006133 | 0.603098815108403 | 325.048109226958 | 315.743220185071 | 548.646432756841 | 610.745080421919 | 150.843090382138 | 134.230762437004 |
| YCL005W | 1353.95702088232 | 0.853634877815199 | 0.364665334948246 | 2.34087201608113 | 0.0192387608643331 | 0.0410937379656071 | 1018.23210029933 | 1065.94251618615 | 809.070606172088 | 1148.305300993 | 1971.95915030816 | 2110.23245133523 |
| YCL005W-A | 3665.99126795124 | 1.05735659453369 | 0.392720643674947 | 2.69238862678392 | 0.00709422255587969 | 0.0178883293891406 | 2725.86856249398 | 2426.19398695734 | 1986.10008657976 | 2929.13689069685 | 5988.15643173258 | 5940.49164924694 |
| YCL007C | 9.30802246537499 | 0.559522127842789 | 0.700070744459212 | 0.799236551835923 | 0.424153261197613 | 0.521486636523984 | 9.07111002493835 | 6.59515864616336 | 6.58375719308209 | 6.96998665246128 | 14.1415397233254 | 12.4865825522794 |
| YCL008C | 934.247740973191 | -0.188407024906322 | 0.662371568177886 | -0.284443104079195 | 0.776070830246355 | 0.829526331416851 | 737.783615361653 | 755.970059816475 | 1493.04982567562 | 1881.02514783299 | 377.107725955345 | 360.550071197069 |
| YCL009C | 29522.0978774352 | 1.02925472217471 | 0.346979274313446 | 2.96632911061115 | 0.00301377794820017 | 0.00871910601366087 | 17096.0186936671 | 17531.5804711637 | 23620.3262230475 | 28726.7999881192 | 42214.0673563179 | 47943.794532296 |
| YCL010C | 315.453377576098 | -0.258311262723707 | 0.382968521989253 | -0.674497374828513 | 0.499995154153336 | 0.593740967607456 | 353.017365137184 | 355.314172062051 | 324.067159615041 | 409.4867158321 | 197.981556126556 | 252.853296683659 |
| YCL011C | 4341.84269452596 | 0.741846277041141 | 0.293828799074595 | 2.52475686310384 | 0.0115778360225135 | 0.0269174238212949 | 3170.35295371595 | 3248.94002806622 | 3328.4550253915 | 4456.43521591743 | 5427.208689374 | 6419.66425469067 |
| YCL012C | 639.720926518516 | 1.30823402436007 | 0.445326835356674 | 2.93769411697876 | 0.00330663092660189 | 0.00944332734692514 | 284.984039950147 | 323.162773662004 | 495.244846635175 | 495.740300656308 | 1084.18471212162 | 1155.00888608585 |
| YCL014W | 386.631875139451 | -2.11070216959666 | 0.434551336405806 | -4.85719866162277 | 1.19058128392284e-06 | 1.76505400761077e-05 | 804.305088877867 | 738.657768370296 | 341.62384546326 | 176.863411306205 | 120.988728744006 | 137.352408075074 |
| YCL016C | 387.616394785934 | -0.67424495664918 | 0.784659183012589 | -0.859283840992609 | NA | NA | 487.572163840437 | 492.988108800711 | 450.621603437619 | 745.788571813357 | 67.565134233666 | 81.1627865898164 |
| YCL017C | 3785.92299569984 | 1.25492930541754 | 0.309281855143325 | 4.05755877542828 | 4.95883399111533e-05 | 0.000322900818026114 | 1954.82421037422 | 2144.25095483386 | 2607.89937703752 | 4395.44783270839 | 5482.20356607583 | 6130.9120331692 |
| YCL018W | 15908.2713745376 | 2.09842381743197 | 0.392694961474087 | 5.34364843784846 | 9.1094165802697e-08 | 2.66657467167895e-06 | 6186.49703700796 | 5643.80701145429 | 6238.47570473378 | 14596.894546917 | 30694.9976105736 | 32088.9563365391 |
| YCL019W | 8.7217460943512 | -4.47323610822985 | 1.40383440746792 | -3.18644142388429 | 0.00144034650986215 | 0.0047592773337672 | 27.9692559102266 | 18.9610811077197 | 3.6576428850456 | 1.74249666311532 | 0 | 0 |
| YCL021W-A | 426.074930160295 | 1.41265855154079 | 0.420928973191442 | 3.35604969368145 | 0.000790643607672458 | 0.00294150481421758 | 170.839238803006 | 200.327943877212 | 326.261745346068 | 408.615467500542 | 763.643145059573 | 686.762040375369 |
| YCL023C | 8.83197065364355 | 1.30260997060555 | 1.00126511716784 | 1.30096409858968 | 0.1932707437702 | 0.276469033736137 | 0 | 4.94636898462252 | 10.2414000781277 | 9.58373164713426 | 18.8553862977672 | 9.36493691420958 |
| YCL024W | 96.8247444097955 | -3.02436713016716 | 0.644739119083945 | -4.69083857431239 | 2.72087580144629e-06 | 3.41568034333608e-05 | 260.794413216978 | 211.045076677227 | 46.0863003515746 | 27.0086982782874 | 17.2841041062866 | 18.7298738284192 |
| YCL025C | 16741.5902283857 | 1.70197373457144 | 0.403800934355818 | 4.21488310146332 | 2.49907599660136e-05 | 0.000187795209079496 | 7490.46910309285 | 7862.25350105749 | 8262.61527731802 | 13886.8271566975 | 32032.1587555236 | 30915.2175766249 |
| YCL026C-A | 43.2565646120863 | -0.0358312211697555 | 0.446056535344179 | -0.0803288783609191 | 0.935975690811322 | 0.954200324335114 | 36.2844400997534 | 46.9905053539139 | 47.5493575055929 | 33.1074365991911 | 59.708723276263 | 35.8989248378034 |
| YCL026C-B | 166.090300172408 | 0.184291777469861 | 0.386751175120825 | 0.476512521034453 | 0.633709289187981 | 0.714379271476048 | 134.554798703252 | 119.537250461711 | 212.874815909654 | 211.713344568511 | 152.414372573619 | 165.447218817703 |
| YCL027W | 81.2475825972535 | -0.73936384345509 | 0.656567963467086 | -1.12610405105785 | 0.260121479523863 | 0.350823524216477 | 77.8603610473875 | 79.9662985847307 | 148.500301132852 | 126.331008075861 | 29.8543616381315 | 24.9731651045589 |
| YCL028W | 1753.99043896715 | -0.403140591790485 | 0.770784943942697 | -0.523026033342519 | NA | NA | 1546.62425925199 | 1728.75596012557 | 2718.36019216589 | 3696.70667079915 | 471.384657444181 | 362.110894016104 |
| YCL029C | 170.4747128425 | -3.15990098144295 | 0.683160601572055 | -4.6254145426003 | 3.7384992532666e-06 | 4.39473460485455e-05 | 452.043649576095 | 411.373020554439 | 57.0592290067114 | 44.4336649094406 | 28.2830794466509 | 29.6556335616637 |
| YCL030C | 4502.33941747816 | -0.246535988398362 | 0.272516604047295 | -0.904664100230664 | 0.365643365368028 | 0.462803316228401 | 4476.59279730708 | 4732.02632862221 | 5449.88789871795 | 4262.14683798007 | 3829.21470063823 | 4264.16794160343 |
| YCL031C | 279.805282432515 | -4.59328707231642 | 0.532604771102201 | -8.62419437740076 | 6.4545408652772e-18 | 1.38557477241284e-14 | 755.169909576118 | 520.193138216135 | 338.697731155223 | 36.5924299254217 | 12.5702575318448 | 15.6082281903493 |
| YCL032W | 371.80986149353 | -2.1504480855311 | 0.680315828013131 | -3.16095554003425 | 0.001572524990495 | 0.00512243851228519 | 686.380658553669 | 627.364466216289 | 509.143889598348 | 309.293157702969 | 51.8523123188599 | 46.8246845710479 |
| YCL033C | 1534.95696112205 | 1.77417517759408 | 0.420814934447845 | 4.21604613420384 | 2.48622919201231e-05 | 0.000187048084071954 | 608.520297506281 | 573.778802216212 | 900.511678298228 | 1361.76114222462 | 2737.17357755921 | 3027.99626892776 |
| YCL034W | 1616.14237024375 | 1.08535531515736 | 0.373696272032489 | 2.90437822473914 | 0.00367983321960104 | 0.0103169899583068 | 886.701004937724 | 813.677697970404 | 1405.99792501153 | 2645.98118294061 | 1685.98579145869 | 2258.51061914354 |
| YCL035C | 2249.14839349488 | 0.689932937091928 | 0.310581252565016 | 2.2214249295279 | 0.0263221940840624 | 0.053239613662488 | 1565.52240513728 | 1434.44700554053 | 2163.86153079298 | 2687.80110285538 | 2712.03306249552 | 2931.2252541476 |
| YCL036W | 1109.9978819594 | 0.857836887224998 | 0.394874654270819 | 2.17242833376858 | 0.029823369796806 | 0.0588968112515886 | 692.428065236961 | 680.950130216367 | 995.610393309414 | 2033.49360585558 | 1021.33342446239 | 1236.17167267566 |
| YCL037C | 173.086514164968 | -0.598091728233976 | 0.392137373402007 | -1.52520970660154 | 0.127206800310118 | 0.195889955522994 | 232.06923147134 | 262.981951015764 | 130.212086707624 | 129.816001402091 | 139.844115041774 | 143.595699351214 |
| YCL038C | 1242.34031911653 | 1.20943911822874 | 0.414195313295662 | 2.91997296783851 | 0.00350061749368655 | 0.00989928251874483 | 769.532500448937 | 826.04362043196 | 653.986547846154 | 932.235714766696 | 2151.08532013695 | 2121.15821106847 |
| YCL039W | 912.638399029677 | 0.0693650886366095 | 0.544745737037683 | 0.127334798458113 | 0.898675419278193 | 0.92658816845206 | 693.183991072373 | 687.54528886253 | 1292.61099557512 | 1697.19174987432 | 504.381583465274 | 600.916785328448 |
| YCL040W | 10724.7986363764 | 2.54272425352034 | 0.455581852943785 | 5.58126764068913 | 2.3877188713165e-08 | 9.732221222328e-07 | 2514.96525441416 | 2535.83849944981 | 4374.54089051454 | 9603.77035876008 | 22453.6225162578 | 22866.0542988617 |
| YCL041C | 8.38713447797459 | 2.14596808420376 | 0.931536376837826 | 2.30368683130595 | 0.0212402295923068 | 0.0447091679068241 | 0.75592583541153 | 2.47318449231126 | 5.85222861607297 | 5.22748998934596 | 17.2841041062866 | 18.7298738284192 |
| YCL042W | 63.0244974579866 | 1.58116684504104 | 0.562461825813724 | 2.8111540596618 | 0.00493641431533884 | 0.0132185065242337 | 22.6777750623459 | 14.0147121230971 | 57.7907575837206 | 87.9960814873236 | 84.8492383399526 | 110.81842015148 |
| YCL043C | 31614.424130098 | 1.4783272634342 | 0.362112107672396 | 4.08251265868096 | 4.45513845093072e-05 | 0.000298865537749936 | 12890.8032712728 | 13740.1886444506 | 23467.4367504526 | 43030.0838473012 | 44209.5957394983 | 52348.4365276125 |
| YCL044C | 851.862468130535 | -0.50781443917527 | 0.54817040257018 | -0.926380623241066 | 0.354248210313526 | 0.45095047922892 | 799.769533865398 | 915.902656985936 | 1286.75876695904 | 1332.13869895166 | 366.108750614981 | 410.496401406187 |
| YCL045C | 4778.34929481686 | -0.464990012786727 | 0.404066913821491 | -1.15077477734827 | 0.249824903913933 | 0.339926554237425 | 4958.87348029963 | 5620.72395619272 | 6047.5467461344 | 6070.85837429377 | 3340.54593908776 | 2631.54727289289 |
| YCL046W | 1.97035771219054 | -0.0473763086959926 | 1.41043053186813 | -0.0335899625153763 | 0.973204126505381 | 0.98083546550264 | 1.51185167082306 | 1.64878966154084 | 2.92611430803648 | 2.61374499467298 | 0 | 3.12164563806986 |
| YCL047C | 530.333800688798 | 1.10945438549837 | 0.314335865160027 | 3.52951892693998 | 0.00041631592470756 | 0.00174877192090882 | 351.505513466361 | 299.255323569662 | 356.254417003442 | 581.122637148959 | 743.216476570326 | 850.648436374037 |
| YCL048W | 52.1502521675135 | -0.32482669893825 | 0.405477658111938 | -0.801096416633088 | 0.423075830536115 | 0.520559485795296 | 61.2299926683339 | 51.9368743385364 | 59.9853433147479 | 34.8499332623064 | 48.7097479358987 | 56.1896214852575 |
| YCL048W-A | 55.1017625679008 | 2.21644869261204 | 0.647404148261344 | 3.42359359074929 | 0.000617989674057689 | 0.00240911228869947 | 15.8744425436421 | 11.5415276307859 | 30.7242002343831 | 29.6224432729604 | 138.272832850293 | 104.57512887534 |
| YCL049C | 1773.07452916169 | 1.50391265721966 | 0.405046782356152 | 3.71293569713458 | 0.000204868952638106 | 0.000983126717577795 | 725.688801995068 | 747.72611150877 | 1299.1947527682 | 1788.67282468788 | 2946.15410902613 | 3131.01057498407 |
| YCL050C | 5324.37892264277 | 0.142365503796755 | 0.327901844832765 | 0.434171097358001 | 0.664164199676637 | 0.740515485789048 | 4407.80354628463 | 4489.6542483757 | 6288.95117654741 | 6691.18718636283 | 5364.35740171478 | 4704.31997657128 |
| YCL051W | 305.561750841363 | -2.92057885490192 | 0.707204928152964 | -4.12974901423512 | 3.6315947177709e-05 | 0.000253111146996154 | 615.323630024985 | 563.061669416197 | 443.306317667527 | 164.665934664398 | 28.2830794466509 | 18.7298738284192 |
| YCL052C | 2202.08269887545 | 0.617887570253278 | 0.280523906444633 | 2.20262001226348 | 0.0276215422052747 | 0.0553634397142761 | 1629.77610114726 | 1613.34068381771 | 1970.00645788556 | 2891.67321243987 | 2448.05765432678 | 2659.64208363552 |
| YCL054W | 350.789310323465 | -2.34296330567453 | 0.593161442451756 | -3.94995887795774 | 7.8164621159988e-05 | 0.000464036006482583 | 840.589528977621 | 750.199296001082 | 168.251572712098 | 157.695948011936 | 105.2759068292 | 82.7236094088513 |
| YCL054W-A | 209.83194615954 | 0.572658482185819 | 0.43182075369832 | 1.32614858660983 | 0.184790485250494 | 0.266290159993999 | 178.398497157121 | 187.137626584885 | 139.721958208742 | 135.043491391437 | 325.255413636485 | 293.434689978567 |
| YCL055W | 312.846903310491 | -1.62542598983671 | 0.490911077657583 | -3.31103954221719 | 0.000929500730520602 | 0.00335913844250992 | 504.20253221949 | 472.37823803145 | 443.306317667527 | 269.215734451317 | 102.133342446239 | 85.8452550469212 |
| YCL056C | 242.078690719428 | 0.19845887693567 | 0.571514787631213 | 0.34725064203191 | 0.728403028192017 | 0.791921439646503 | 281.204410773089 | 259.684371692682 | 134.601258169678 | 86.2535848242083 | 361.394904040539 | 329.33361481637 |
| YCL057C-A | 3084.11629467467 | 1.74655862217038 | 0.408987887358263 | 4.27044094985736 | 1.95086870365557e-05 | 0.000153214566482218 | 1474.81130488789 | 1329.74886203269 | 1443.305882439 | 2540.56013482214 | 5972.44360981777 | 5743.82797404854 |
| YCL057W | 3234.87569704034 | 1.16049397797825 | 0.357687523626614 | 3.24443516008593 | 0.00117683807046184 | 0.0040592798543059 | 1602.56277107244 | 1582.83807507921 | 2813.45890717708 | 4275.21556295344 | 4407.44654710309 | 4727.7323188568 |
| YCL058C | 784.463175742362 | 1.76770119148304 | 0.45758183712618 | 3.86313670705332 | 0.000111940320735811 | 0.000613528225990318 | 330.339590074838 | 280.294242461943 | 457.205360630701 | 597.676355448554 | 1524.14372573619 | 1517.11978010195 |
| YCL058W-A | 88.6995964096729 | -2.79664149795702 | 0.654530680949459 | -4.27274317210039 | 1.93082803979446e-05 | 0.000152383977650445 | 212.41515975064 | 174.771704123329 | 79.7366148939942 | 41.8199199147677 | 6.28512876592241 | 17.1690510093842 |
| YCL059C | 706.71593439828 | -3.26471035232755 | 0.813973599332957 | -4.01083076281951 | 6.05054706095853e-05 | 0.000380151444610467 | 1626.75239780561 | 1254.72893243258 | 961.228550189985 | 338.044352644372 | 34.5682082125733 | 24.9731651045589 |
| YCL061C | 16.7182836249274 | -2.89122974467118 | 0.905618337438937 | -3.19254770486152 | 0.00141023648105869 | 0.00468152779927382 | 37.0403659351649 | 42.0441363692914 | 10.2414000781277 | 7.84123498401894 | 3.14256438296121 | 0 |
| YCL063W | 448.240069955732 | -0.979794766392336 | 0.435109241643739 | -2.25183625769681 | 0.0243326214816342 | 0.0500166237924431 | 598.693261645931 | 596.861857477784 | 591.07509022337 | 486.156569009174 | 221.550788998765 | 195.102852379366 |
| YCL064C | 4280.59249823456 | -0.76952420938981 | 0.656821986000572 | -1.17158716637287 | 0.241362843050765 | 0.330507486550484 | 7481.39799306791 | 7816.08739053435 | 890.2702782201 | 3161.76019522275 | 3081.28437749346 | 3252.75475486879 |
| YCL068C | 9.2947148099237 | -1.2522838533086 | 1.00512908256269 | -1.24589356236292 | 0.212803473830222 | 0.298834359238253 | 14.3625908728191 | 15.663501784638 | 10.2414000781277 | 13.9399733049226 | 0 | 1.56082281903493 |
| YCR002C | 3409.79648637618 | 0.541322433309935 | 0.36037872804764 | 1.50209319024616 | 0.133073043318056 | 0.203464007352394 | 2622.3067230426 | 2950.50909932733 | 2758.5942639014 | 2576.281316416 | 4751.55734703735 | 4799.53016853241 |
| YCR003W | 363.033040099923 | -1.60397673109713 | 0.461824995773794 | -3.47312671634337 | 0.000514432411406028 | 0.00207059045590926 | 518.565123092309 | 504.529636431497 | 618.141647572707 | 298.838177724277 | 131.987704084371 | 106.135951694375 |
| YCR004C | 4852.76362260154 | 1.36315210791287 | 0.391219744352145 | 3.4843642929378 | 0.000493307554221651 | 0.00200182775626177 | 2061.40975316724 | 2128.58745304922 | 3959.76418735037 | 5610.83925523133 | 7359.88578489515 | 7996.09530191595 |
| YCR005C | 9146.29910889251 | 0.141335494850301 | 0.351627928838161 | 0.401946157454835 | 0.687723652114552 | 0.75968101537182 | 9299.39962723263 | 9737.7517410602 | 7057.787710984 | 6837.55690606451 | 11977.8841456566 | 9967.41452235706 |
| YCR006C | 117.198155601721 | 0.522734164378502 | 0.349302452997121 | 1.49650871298866 | 0.134521139082898 | 0.205434227103121 | 100.538136109733 | 98.9273796924503 | 88.5149578181036 | 111.51978643938 | 133.558986275851 | 170.129687274807 |
| YCR007C | 284.46981833075 | -0.58402597771237 | 0.338953203686164 | -1.72302834539106 | 0.0848833992495161 | 0.14013892060035 | 286.49589162097 | 300.079718400433 | 436.722560474445 | 207.357102910723 | 251.405150636897 | 224.75848594103 |
| YCR008W | 1993.86657787516 | 0.306487009042236 | 0.28282921382411 | 1.08364692917768 | 0.278521374706881 | 0.370860312584167 | 1589.71203187045 | 1700.72653587938 | 2057.78988712666 | 2076.1847741019 | 2331.78277215722 | 2207.00346611539 |
| YCR009C | 2097.25534215177 | -0.881779028332875 | 0.32956733886003 | -2.67556558056675 | 0.00746032759297064 | 0.0186580620189246 | 2948.11075810497 | 2808.71318843482 | 2401.60831832094 | 1881.02514783299 | 1396.86986822626 | 1147.20477199067 |
| YCR010C | 37.1546331049416 | 0.114887669610603 | 0.596178112087087 | 0.192706956665026 | 0.847188474946867 | 0.886272543641622 | 49.891105137161 | 41.219741538521 | 15.3621001171915 | 20.9099599573838 | 50.2810301273793 | 45.263861752013 |
| YCR011C | 3474.69442404113 | 1.12942089542434 | 0.357973998500274 | 3.15503612037755 | 0.0016047823625484 | 0.00520885917507117 | 1930.63458364105 | 2000.80625427981 | 2607.89937703752 | 3241.91504172605 | 5494.77382360767 | 5572.1374639547 |
| YCR012W | 409207.454900797 | 2.93955572852931 | 0.509963121268082 | 5.7642515819963 | 8.20209895854962e-09 | 4.29443230024875e-07 | 65215.2336726235 | 67584.7126213898 | 150331.317161105 | 360743.856674775 | 920384.82878853 | 890984.780486357 |
| YCR013C | 46.8614095242737 | 2.3052238668552 | 0.664348721841995 | 3.46990035664351 | 0.000520651479530601 | 0.00209300594767607 | 27.9692559102266 | 6.59515864616336 | 12.4359858091551 | 32.2361882676334 | 87.9918027229138 | 113.94006578955 |
| YCR014C | 113.41454040724 | -2.24467971291286 | 0.55436246124811 | -4.04911924927079 | 5.14107453654002e-05 | 0.000332414859591543 | 195.784791371586 | 165.703360984854 | 202.633415831527 | 74.0561081824011 | 25.1405150636897 | 17.1690510093842 |
| YCR015C | 230.039548503792 | -0.268620608293438 | 0.329027584951055 | -0.816407561491833 | 0.41426707332878 | 0.512069088721179 | 237.36071231922 | 287.713795938876 | 228.236916026846 | 168.150927990628 | 218.408224615804 | 240.366714131379 |
| YCR016W | 73.0595508758665 | -1.9963759508275 | 0.498821802897627 | -4.00218262159086 | 6.27608254227839e-05 | 0.00039202688236928 | 144.381834563602 | 143.444700554053 | 63.6429861997935 | 38.334926588537 | 25.1405150636897 | 23.412342285524 |
| YCR017C | 6995.0587961846 | 1.32756495203109 | 0.377809599818813 | 3.5138465318715 | 0.000441667905939901 | 0.00183151404652477 | 4102.40950877837 | 4290.97509416003 | 3564.00722718844 | 6063.01713930975 | 11591.3487265524 | 12358.5950811186 |
| YCR018C | 346.287004688142 | -0.0691215925764672 | 0.311832004935064 | -0.22166291940066 | 0.824576298040986 | 0.868542911250237 | 319.756628379077 | 350.367803077428 | 394.293903007916 | 385.091762548486 | 348.824646508694 | 279.387284607252 |
| YCR018C-A | 43.8457329292498 | 0.791629935936607 | 0.489236910434411 | 1.61809119273869 | 0.105642946274068 | 0.167695482870346 | 34.7725884289304 | 33.8001880615872 | 27.0665573493375 | 29.6224432729604 | 69.1364164251466 | 68.6762040375369 |
| YCR019W | 581.06380224524 | 0.712212373306765 | 0.295828089039296 | 2.40752112356768 | 0.0160612330276602 | 0.0353742615246689 | 444.484391221979 | 453.417156923731 | 422.823517511272 | 593.320113790766 | 791.926224506224 | 780.411409517465 |
| YCR020C | 196.49340360111 | 0.193747375611884 | 0.384899017451658 | 0.503371967262083 | 0.614702776829471 | 0.698550535165307 | 179.154422992532 | 177.24488861564 | 192.392015753399 | 130.687249733649 | 237.263610913571 | 262.218233597868 |
| YCR020C-A | 803.678345456953 | 0.941348308604358 | 0.423911932036144 | 2.22062234503014 | 0.0263765510567068 | 0.0532941435362854 | 653.119921795561 | 593.564278154702 | 403.803774509035 | 605.517590432573 | 1183.17549018489 | 1382.88901766495 |
| YCR020W-B | 73.0064360080017 | -2.54338657296026 | 0.595166806182157 | -4.27340124909761 | 1.92513566213154e-05 | 0.000152121149253093 | 178.398497157121 | 129.429988430956 | 67.3006290848391 | 33.1074365991911 | 21.9979506807285 | 7.80411409517465 |
| YCR021C | 22968.9865159214 | 1.72540449873268 | 0.388798224401407 | 4.43778903926094 | 9.0887630908964e-06 | 8.50170999015124e-05 | 13459.2594995023 | 8915.83009478209 | 9623.989959132 | 22194.1799980998 | 41673.5462824486 | 41947.1132615637 |
| YCR022C | 37.7343876863185 | 0.899068513197065 | 0.553451014291815 | 1.62447712621414 | 0.10427401830573 | 0.166012261316152 | 34.0166625935188 | 23.9074500923422 | 20.4828001562554 | 22.6524566204991 | 72.2789808081078 | 53.0679758471876 |
| YCR023C | 2040.16763798301 | 1.08029993625027 | 0.343519699175967 | 3.14479763123245 | 0.00166201754556994 | 0.00536242133941404 | 1273.73503266843 | 1366.84662941736 | 1288.95335269007 | 1872.31266451741 | 3162.99105145046 | 3276.16709715432 |
| YCR024C | 363.555592707946 | -0.292762328501734 | 0.338650517199684 | -0.864496918305628 | 0.387314972498847 | 0.485462908309182 | 334.119219251896 | 361.909330708214 | 505.486246713303 | 372.023037575121 | 329.969260210927 | 277.826461788218 |
| YCR024C-A | 11477.8962509974 | 1.12756252502856 | 0.392278714359967 | 2.87439130330655 | 0.00404807082815505 | 0.011164700699494 | 7640.89834433974 | 7745.18943508809 | 6236.28111900276 | 9111.51505143 | 19147.6447853826 | 18985.8487707409 |
| YCR024C-B | 28082.8915975397 | 1.28443019528467 | 0.400699412450078 | 3.20547062305636 | 0.00134841810297169 | 0.00452046464504826 | 15082.9881939662 | 15470.5933942377 | 18486.4586695975 | 22227.287434699 | 48555.7622811336 | 48674.2596116043 |
| YCR025C | 14.7185676235481 | 0.190568067192224 | 0.675473870258943 | 0.282125002287904 | 0.777847662544959 | 0.830736143746192 | 15.8744425436421 | 12.3659224615563 | 12.4359858091551 | 6.96998665246128 | 12.5702575318448 | 28.0948107426287 |
| YCR026C | 736.459445949036 | -2.16104130797964 | 0.867731108262344 | -2.49045042571677 | 0.0127581292848039 | 0.0291459214594314 | 1324.382063641 | 1295.9486739711 | 992.684279001377 | 711.809886882608 | 42.4246191699763 | 51.5071530281527 |
| YCR027C | 751.999635777969 | 0.563061080437008 | 0.33886165784683 | 1.66162523082361 | 0.0965879411872531 | 0.155506585311477 | 620.615110872866 | 656.218285293254 | 543.525732717777 | 642.110020357995 | 963.19598337761 | 1086.33268204831 |
| YCR028C | 1595.25288224775 | 1.09453921726852 | 0.382009884193597 | 2.86521177214836 | 0.00416730448001524 | 0.0114250493194117 | 1017.47617446392 | 1169.81626486323 | 864.666778024781 | 1339.10868560412 | 2591.04433375152 | 2589.40505677895 |
| YCR028C-A | 4976.24317383274 | 0.886897918533249 | 0.399831321575915 | 2.21818019418185 | 0.0265425486102161 | 0.0535886495741585 | 3497.66884044915 | 3627.33725538985 | 3353.32699700981 | 3559.04943441304 | 7674.14222319127 | 8145.9342925433 |
| YCR030C | 2313.75515319361 | 0.0914056043418446 | 0.281485070398383 | 0.324726296185013 | 0.745388230807832 | 0.80609575254449 | 2016.81012887796 | 2092.31408049532 | 2612.28854849957 | 2404.64539509914 | 2441.77252556086 | 2314.7002406288 |
| YCR031C | 122753.221666223 | 1.23402302468375 | 0.46368559125539 | 2.66133571531247 | 0.00778313118948058 | 0.0193302602623428 | 87313.2136192087 | 84487.2798366757 | 47910.0010940584 | 86886.1111129192 | 213892.359597489 | 216030.364736987 |
| YCR032W | 636.635635074615 | -0.979682961981095 | 0.292800611280946 | -3.34590477012726 | 0.000820145577195796 | 0.00302505012436479 | 878.385820748197 | 891.170812062824 | 765.91042012855 | 479.186582356713 | 435.245167040127 | 369.915008111278 |
| YCR033W | 639.832067915346 | -0.203903218485235 | 0.275480560292562 | -0.74017280300537 | 0.459195147873765 | 0.555972316658592 | 706.79065610978 | 723.818661416428 | 623.99387618878 | 562.826422186248 | 619.085183443358 | 602.477608147483 |
| YCR034W | 17073.2884921117 | 1.37129952071296 | 0.410976733580036 | 3.33668407154709 | 0.000847842644457655 | 0.00311294562731317 | 10241.2832181554 | 10549.7806493691 | 7766.63890210584 | 13604.5426972729 | 29320.1256930281 | 30957.3597927388 |
| YCR035C | 876.104465490066 | -2.27791313540185 | 0.766337507691515 | -2.97246723870236 | 0.00295416735699359 | 0.00857747420155037 | 1641.87091451384 | 1614.98947347925 | 1103.14509412975 | 735.333591834665 | 78.5641095740302 | 82.7236094088513 |
| YCR036W | 1828.65495500502 | 1.07343776045788 | 0.38175623817109 | 2.8118407851054 | 0.00492588793550131 | 0.0132063759623743 | 1094.58060967589 | 1154.97715790936 | 1283.83265265101 | 1483.73590864269 | 2853.44845972878 | 3101.35494142241 |
| YCR037C | 2280.86036863502 | 0.167090171104834 | 0.345531118777952 | 0.483574885225347 | 0.628687602171657 | 0.710057551382931 | 2400.82045326702 | 2328.09100209566 | 1716.89757024041 | 1759.05038141491 | 2851.8771775373 | 2628.42562725482 |
| YCR038C | 159.964845384473 | -1.63952224986612 | 0.679721963430717 | -2.41204836399734 | 0.0158631771199412 | 0.034985911182336 | 232.06923147134 | 211.869471507998 | 284.564616456548 | 173.378417979974 | 23.5692328722091 | 34.3381020187685 |
| YCR041W | 12.1222889118861 | 1.79555764844458 | 0.677075901571066 | 2.65192963488765 | 0.0080033218932252 | 0.0197931616714172 | 5.29148084788071 | 4.1219741538521 | 6.58375719308209 | 11.3262283102496 | 21.9979506807285 | 23.412342285524 |
| YCR042C | 654.386063320835 | -0.960461872475297 | 0.304280626149641 | -3.15650024988103 | 0.00159674750574286 | 0.00518822095710596 | 957.002107630996 | 915.078262155166 | 721.287176930993 | 432.139172452599 | 501.239019082313 | 399.570641672942 |
| YCR043C | 2488.05092068376 | 0.557680209814303 | 0.406966833063152 | 1.37033331590381 | 0.170582877708972 | 0.248879413784726 | 2143.8056692271 | 2342.93010904953 | 1551.57211183635 | 1729.42793814195 | 3444.25056372548 | 3716.31913212217 |
| YCR044C | 1902.95019648119 | 1.58672630753613 | 0.37986204631758 | 4.17711198820205 | 2.95233690172552e-05 | 0.000214836719176411 | 925.253222543712 | 985.151822770651 | 940.74575003373 | 1698.93424653744 | 3516.52954453359 | 3351.086592468 |
| YCR045C | 28.8010187479809 | 1.18581774375412 | 0.581809155875892 | 2.03815586567887 | 0.0415343476612502 | 0.0780055989905079 | 9.07111002493835 | 20.6098707692605 | 22.6773858872828 | 21.7812082889415 | 50.2810301273793 | 48.3855073900828 |
| YCR045W-A | 395.816063234617 | 1.57659180642421 | 0.442338131825241 | 3.5642231428673 | 0.000364935407466573 | 0.00157180726266515 | 166.303683790536 | 174.771704123329 | 254.571944799174 | 316.26314435543 | 777.784684782899 | 685.201217556334 |
| YCR046C | 2104.27462978319 | 2.1258849846206 | 0.454771618584057 | 4.67462105757522 | 2.94496881565893e-06 | 3.63325654652174e-05 | 595.669558304285 | 495.461293293022 | 1261.88679534073 | 2520.52142319631 | 3676.80032806461 | 4075.3083805002 |
| YCR047C | 2984.67704573316 | 1.60156658173069 | 0.321985819305077 | 4.97402831338116 | 6.55757249515947e-07 | 1.11317889741651e-05 | 1545.86833341658 | 1404.76879163279 | 1487.19759705954 | 3295.93243828263 | 4817.55119907953 | 5356.74391492788 |
| YCR047W-A | 5.41302590944803 | 1.8627134395312 | 0.961683458013823 | 1.93692989518431 | 0.0527539135018739 | 0.0947129085453215 | 0.75592583541153 | 2.47318449231126 | 3.6576428850456 | 5.22748998934596 | 10.9989753403642 | 9.36493691420958 |
| YCR048W | 4487.95525139632 | 1.90116105052076 | 0.418252478007024 | 4.54548663902675 | 5.48084740900477e-06 | 5.82453090989946e-05 | 1686.47053880312 | 1861.48352787961 | 2138.25803059766 | 3721.10162408276 | 8937.45310514167 | 8582.96468187308 |
| YCR049C | 2.47025809796504 | 1.10959355530408 | 1.49648162163401 | 0.741468214018235 | 0.458409597636743 | 0.555277271633426 | 0 | 1.64878966154084 | 2.92611430803648 | 0.87124833155766 | 1.5712821914806 | 7.80411409517465 |
| YCR050C | 2.07734835169153 | 1.53778442466812 | 1.55846216088351 | 0.986731961330605 | 0.32377405720144 | 0.419369454621334 | 1.51185167082306 | 0.82439483077042 | 0.731528577009121 | 0 | 4.71384657444181 | 4.68246845710479 |
| YCR051W | 5271.10288708493 | 1.99035292839446 | 0.328147616479248 | 6.06541942845509 | 1.31609736045215e-09 | 1.24642161783998e-07 | 2071.23678902759 | 1973.60122486438 | 2313.82488907985 | 6101.35206589829 | 9099.29517086418 | 10067.3071827753 |
| YCR052W | 918.61598917219 | -1.2612285013926 | 0.766932565403389 | -1.64451029762861 | NA | NA | 1392.41538882804 | 1489.68145920215 | 1008.77790769558 | 1329.52495395699 | 147.700525999177 | 143.595699351214 |
| YCR053W | 11159.7534742909 | -0.468628056285457 | 0.574371799469307 | -0.815896700914717 | 0.414559219190168 | 0.512233570910338 | 11912.6352402503 | 12678.3681024183 | 14280.1693517951 | 18752.7490884471 | 5290.50713871519 | 4044.0919241195 |
| YCR054C | 867.143056554104 | 0.515144261562952 | 0.317599354929301 | 1.62199404239226 | 0.104804623764483 | 0.166652290627967 | 732.492134513772 | 716.399107939495 | 692.026033850629 | 774.539766754759 | 1057.47291486645 | 1229.92838139952 |
| YCR057C | 4131.65096302693 | 0.53124688465752 | 0.339362799365026 | 1.56542463007591 | 0.117483377007345 | 0.183594503258263 | 3275.42664483816 | 3293.45734892783 | 3570.59098438152 | 6609.28984319641 | 3953.3459937652 | 4087.79496305248 |
| YCR059C | 4717.04132703421 | 2.19143486887103 | 0.468325867378865 | 4.67929495574544 | 2.87863077113201e-06 | 3.57883825600195e-05 | 1464.22834319213 | 1342.11478449424 | 2276.51693165238 | 3701.9341607885 | 9987.06960905072 | 9530.38413302728 |
| YCR060W | 1199.77742276076 | 0.698351951992411 | 0.326765410576724 | 2.13716608119524 | 0.0325844857624741 | 0.0634545171788125 | 941.127665087354 | 885.400048247431 | 917.336835569438 | 1085.57542112084 | 1552.42680518284 | 1816.79776135666 |
| YCR061W | 864.99289797172 | 2.20091361941738 | 0.523368212699217 | 4.20528714968453 | 2.60750886075219e-05 | 0.000194241052365645 | 202.58812389029 | 206.923102523375 | 517.190703945449 | 760.599793449837 | 1901.25145169153 | 1601.40421232984 |
| YCR063W | 106.119929005424 | -0.719444723577487 | 0.551307344063722 | -1.30497939366164 | 0.191899864595237 | 0.274996690697225 | 130.019243690783 | 103.873748677073 | 163.862401250043 | 148.112216364802 | 48.7097479358987 | 42.1422161139431 |
| YCR064C | 31.9407507213322 | 1.42884132732072 | 0.661776117460585 | 2.15910077384415 | 0.0308423496422726 | 0.0606302599805359 | 18.1422200498767 | 23.9074500923422 | 9.50987150111857 | 14.8112216364802 | 61.2800054677435 | 63.9937355804321 |
| YCR065W | 497.536304044749 | -0.365737732177451 | 0.356928438397808 | -1.02468084028044 | 0.30551380635 | 0.400633050884545 | 469.42994379056 | 502.880846769956 | 709.582719698847 | 553.242690539114 | 367.680032806461 | 382.401590663558 |
| YCR066W | 155.185329544194 | -2.82596175329417 | 0.435793906634139 | -6.4846288813915 | 8.89504603190787e-11 | 1.90946988151622e-08 | 306.149963341669 | 284.416216615795 | 227.505387449837 | 56.6311415512479 | 32.9969260210927 | 23.412342285524 |
| YCR067C | 2609.61783250086 | 0.0960646435697943 | 0.277816042465553 | 0.345785083961469 | 0.729504235751802 | 0.792645061285913 | 2403.08823077325 | 2460.8185698497 | 2705.19267777973 | 3067.66537541452 | 2545.47715019858 | 2475.4649909894 |
| YCR068W | 338.337005988377 | 0.252950044162841 | 0.428025825082353 | 0.590969117609139 | 0.554541113293146 | 0.644280131626892 | 230.557379800516 | 246.494054400355 | 449.890074860609 | 487.899065672289 | 267.117972551703 | 348.063488644789 |
| YCR069W | 3316.65957737216 | 0.998695607031831 | 0.348166352206843 | 2.86844377896263 | 0.00412496501755152 | 0.0113282621377534 | 1718.21942389041 | 1946.39619544896 | 2972.20060838806 | 3793.41523560205 | 4721.70298539921 | 4748.02301550426 |
| YCR071C | 310.629416554743 | -0.361358307356848 | 0.45182173191887 | -0.799780714004553 | 0.423837858911633 | 0.521221392552894 | 309.929592518727 | 257.211187200371 | 482.077332249011 | 406.001722505869 | 177.554887637308 | 231.00177721717 |
| YCR072C | 1303.22858938332 | 0.79408816839694 | 0.318794167525086 | 2.49091184622897 | 0.0127415728930797 | 0.0291287644414033 | 839.077677306798 | 958.771188185998 | 1062.91102239425 | 1989.05994094614 | 1302.59293673742 | 1666.95877072931 |
| YCR073C | 752.935111658509 | -0.858784544991179 | 0.387687123989165 | -2.21514848405226 | 0.0267498744971845 | 0.0539690450381792 | 916.938038354185 | 972.785900309095 | 1024.14000781277 | 787.608491728124 | 443.10157799753 | 373.036653749348 |
| YCR073W-A | 2637.53930754412 | 2.11661757099652 | 0.412194459032493 | 5.13499763185722 | 2.8214757682694e-07 | 6.25412977010055e-06 | 751.39028039906 | 726.29184590874 | 1487.19759705954 | 3295.93243828263 | 4644.71015801666 | 4919.7135255981 |
| YCR075C | 862.272357485188 | 1.81149127616782 | 0.414781946078957 | 4.3673339529175 | 1.25772346942658e-05 | 0.000110200532559281 | 355.285142643419 | 374.27525316977 | 416.971288895199 | 720.522370198184 | 1755.12220788383 | 1551.45788212072 |
| YCR075W-A | 267.972613134074 | 0.332346470774866 | 0.320381948948357 | 1.03734455660121 | 0.299575307408904 | 0.394210253312902 | 233.581083142163 | 200.327943877212 | 277.249330686457 | 265.730741125086 | 290.687205423912 | 340.259374549615 |
| YCR076C | 667.896231873392 | 1.5930705814049 | 0.354120283823888 | 4.49867080248124 | 6.8379646665678e-06 | 6.86621749313954e-05 | 290.275520798027 | 299.255323569662 | 407.46141739408 | 725.74986018753 | 1110.89650937679 | 1173.73875991427 |
| YCR077C | 2999.45386518214 | -0.0095181289714835 | 0.481948347311216 | -0.0197492719387566 | 0.984243385108205 | 0.988695585727163 | 2415.93896997525 | 2523.47257698825 | 4089.976274058 | 4998.35167814629 | 2081.9489037118 | 1887.03478821323 |
| YCR079W | 1361.0776769361 | 1.55786413963487 | 0.449151449305673 | 3.46846067633336 | 0.000523449128004181 | 0.00210293972822641 | 610.032149177104 | 594.388672985472 | 865.39830660179 | 1008.90556794377 | 2732.45973098477 | 2355.28163392371 |
| YCR081C-A | 3.52767464045205 | 1.50770151703735 | 1.1688852055644 | 1.28986277682362 | 0.197098306820083 | 0.281194748764142 | 1.51185167082306 | 1.64878966154084 | 2.19458573102736 | 1.74249666311532 | 3.14256438296121 | 10.9257597332445 |
| YCR081W | 346.787133054447 | -1.17600152160777 | 0.504403037361454 | -2.33147192721017 | 0.0197284886715644 | 0.0419804300122636 | 462.626611271856 | 488.041739816088 | 493.781789481157 | 382.478017553813 | 141.415397233254 | 112.379242970515 |
| YCR082W | 1042.77377958594 | -0.683943147472529 | 0.270097547554063 | -2.53220791401532 | 0.011334675966816 | 0.0264859627091056 | 1216.28466917715 | 1270.39243421722 | 1370.15302473808 | 857.308358252737 | 765.214427251054 | 777.289763879395 |
| YCR083W | 520.251979415234 | 1.14329116774642 | 0.41989318489816 | 2.72281429865005 | 0.00647284306930329 | 0.0165548488349139 | 260.038487381566 | 224.235393969554 | 487.929560865084 | 548.886448881326 | 790.354942314744 | 810.067043079129 |
| YCR084C | 5346.25082779612 | 0.332999636509554 | 0.315261048084344 | 1.05626634984879 | 0.290846545080369 | 0.384531256480718 | 4922.58904019988 | 5263.76099446913 | 4008.77660200998 | 4748.30340698924 | 6996.91959866313 | 6137.15532444535 |
| YCR085W | 3.43132734584266 | 0.730957305582315 | 1.2834508337682 | 0.569524976220735 | 0.568999925928137 | 0.657874241109013 | 6.04740668329224 | 1.64878966154084 | 0 | 3.48499332623064 | 6.28512876592241 | 3.12164563806986 |
| YCR086W | 101.166909060096 | -2.14687844588653 | 0.572056144709981 | -3.75291562854367 | 0.000174789696529587 | 0.000871242759791441 | 201.832198054878 | 168.176545477166 | 127.285972399587 | 70.5711148561704 | 17.2841041062866 | 21.851519466489 |
| YCR087C-A | 791.683424176351 | -1.12415440838192 | 0.407213360870659 | -2.76060295757088 | 0.00576947695157205 | 0.0150670849830186 | 1358.39872623452 | 1089.02557144772 | 810.533663326106 | 710.93863855105 | 353.538493083136 | 427.665452415571 |
| YCR087W | 6.137532699634 | 0.982183558808256 | 0.865001344914432 | 1.13547055687574 | 0.256178211537415 | 0.346885551366895 | 3.77962917705765 | 2.47318449231126 | 5.85222861607297 | 4.3562416577883 | 10.9989753403642 | 9.36493691420958 |
| YCR088W | 540.818865587061 | -1.3202176729997 | 0.543627276384952 | -2.42853464193146 | 0.0151599767629997 | 0.0337354009515267 | 892.748411621016 | 920.024631139788 | 506.217775290312 | 568.925160507152 | 169.698476679905 | 187.298738284192 |
| YCR089W | 631.498808819208 | -0.449528858174889 | 0.411699351774522 | -1.09188624232055 | 0.274883105495776 | 0.366814587524409 | 711.326211122249 | 596.037462647013 | 881.491935295991 | 799.805968369932 | 413.247216359399 | 387.084059120663 |
| YCR090C | 1304.13130964996 | 0.134889654999074 | 0.366641694739731 | 0.367905933597727 | 0.712943375708338 | 0.779252433734165 | 1433.23538394026 | 1309.9633860942 | 985.368993231286 | 923.523231451119 | 1572.85347367208 | 1599.8433895108 |
| YCR091W | 75.8039477008487 | -2.98868502266935 | 0.605001977753371 | -4.93995909528693 | 7.81389610119299e-07 | 1.28371150233885e-05 | 158.744425436421 | 143.444700554053 | 103.877057935295 | 33.1074365991911 | 6.28512876592241 | 9.36493691420958 |
| YCR092C | 490.949730162872 | -0.775612730727123 | 0.285382541525113 | -2.71780020803715 | 0.00657175075287703 | 0.0167289439614781 | 653.875847630973 | 654.569495631713 | 550.841018487868 | 350.241829286179 | 388.106701295709 | 348.063488644789 |
| YCR093W | 2052.14684496676 | -0.6948887264799 | 0.284898877233123 | -2.43907148118136 | 0.0147250552228112 | 0.0329497413602864 | 2308.59750134681 | 2542.43365809597 | 2760.78884963242 | 1729.42793814195 | 1615.27809284206 | 1356.35502974135 |
| YCR094W | 688.09019234148 | -0.576756146853304 | 0.300785943753498 | -1.91749700686137 | 0.0551748208692245 | 0.0982377236377677 | 836.053973965152 | 847.477886031991 | 789.319334592842 | 669.118718636283 | 488.668761550468 | 497.902479272143 |
| YCR095C | 362.722789510677 | -1.44768971533557 | 0.362455626257012 | -3.99411572193124 | 6.4936144563631e-05 | 0.000400976130265874 | 616.079555860397 | 564.710459077737 | 413.313646010153 | 251.790767820164 | 166.555912296944 | 163.886395998668 |
| YCR095W-A | 17.2276211018787 | 0.773681104971714 | 0.571396916767588 | 1.35401694035812 | 0.175730973183523 | 0.255155789982584 | 9.82703586034988 | 14.0147121230971 | 13.8990429631733 | 13.9399733049226 | 26.7117972551703 | 24.9731651045589 |
| YCR097W | 290.920436441884 | -1.39450478744291 | 0.355185300976953 | -3.92613315812131 | 8.63223801160847e-05 | 0.000499475406960993 | 517.053271421486 | 352.016592738969 | 396.488488738944 | 191.674632942685 | 164.984630105463 | 123.305002703759 |
| YCR098C | 2721.89743741775 | 3.87754752801188 | 0.392418290783546 | 9.88115900578829 | 5.02483300628398e-23 | 1.61799622802344e-19 | 281.9603366085 | 327.284747815857 | 430.870331858372 | 3180.92765851702 | 6084.0046454129 | 6026.33690429386 |
| YCR099C | 324.177260969577 | 0.4475478510662 | 0.344814520015998 | 1.29793794949655 | 0.19430865816806 | 0.277768647858448 | 240.384415660866 | 254.73800270806 | 326.993273923077 | 298.838177724277 | 468.24209306122 | 355.867602739964 |
| YCR100C | 404.650478545948 | 0.482467002729395 | 0.302479393029805 | 1.59504089814758 | 0.110703090409919 | 0.174780069193399 | 311.44144418955 | 306.674877046596 | 394.293903007916 | 436.495414110387 | 523.236969763041 | 455.7602631582 |
| YCR101C | 164.831881840201 | 0.392087237716314 | 0.399412854969119 | 0.981659034851617 | 0.326267853308089 | 0.421666661710634 | 111.877023640906 | 121.186040123252 | 193.855072907417 | 137.65723638611 | 215.265660232843 | 209.150257750681 |
| YCR102C | 383.838740162136 | 2.00717561842392 | 0.518223394757214 | 3.87318604048023 | 0.00010742176335911 | 0.000592489737727779 | 90.7111002493835 | 94.8054055385983 | 272.860159224402 | 432.139172452599 | 699.220575208869 | 713.296028298963 |
| YCR102W-A | 21.3658604510889 | 1.85303812221579 | 0.572440186928512 | 3.23708601270371 | 0.00120757008794001 | 0.00414097516844177 | 9.07111002493835 | 7.41955347693378 | 10.9729286551368 | 20.9099599573838 | 32.9969260210927 | 46.8246845710479 |
| YCR104W | 26.549188059572 | 1.52414463624708 | 0.602621407086185 | 2.52919099508375 | 0.0114325794571142 | 0.0266856874606072 | 9.07111002493835 | 8.2439483077042 | 23.4089144642919 | 26.1374499467298 | 51.8523123188599 | 40.5813932949082 |
| YCR105W | 109.567236412126 | 0.785513683372519 | 0.417802339808877 | 1.88010838745387 | 0.0600933076120868 | 0.105421111692138 | 74.8366577057414 | 95.6298003693687 | 70.2267433928756 | 87.9960814873236 | 141.415397233254 | 187.298738284192 |
| YCR106W | 380.866102077534 | 0.479327315357146 | 0.305702455982078 | 1.56795376019238 | 0.116891924012195 | 0.182758919795712 | 282.716262443912 | 314.9188253543 | 356.254417003442 | 382.478017553813 | 446.244142380491 | 502.584947729247 |
| YCR107W | 567.586668563747 | 1.0998236418011 | 0.351823206638619 | 3.12606906266647 | 0.00177159941510265 | 0.00567313920104561 | 320.512554214488 | 342.948249600495 | 419.165874626226 | 543.65895889198 | 922.342646399114 | 856.891727650177 |
| YDL001W | 297.444160627709 | -0.734080190046177 | 0.29686136436415 | -2.47280474378507 | 0.0134057364060296 | 0.0304203461785873 | 415.759209476341 | 356.962961723592 | 341.62384546326 | 216.0695862263 | 241.977457488013 | 212.27190338875 |
| YDL002C | 267.187988149693 | -2.51498502800787 | 0.467502873549602 | -5.37961405223541 | 7.46456925093526e-08 | 2.27828559128072e-06 | 560.896969875355 | 544.924983139247 | 259.692644838238 | 102.807303123804 | 86.4205205314332 | 48.3855073900828 |
| YDL003W | 253.677519347314 | -3.17448186549632 | 0.602823485665599 | -5.26602221210951 | 1.39411423967438e-07 | 3.67954741946844e-06 | 688.648436059903 | 551.520141785411 | 130.943615284633 | 67.9573698614975 | 40.8533369784957 | 42.1422161139431 |
| YDL004W | 6522.36075254732 | 0.886656194526271 | 0.372221343119388 | 2.38206704402192 | 0.0172157628058004 | 0.0374516735538414 | 4861.35904753155 | 4788.0851771146 | 4086.31863117295 | 5225.74749268284 | 9935.21729673186 | 10237.4368700501 |
| YDL005C | 121.201109768607 | -1.68141720342417 | 0.574146787112318 | -2.92854935561146 | 0.00340547761484646 | 0.00968268248989457 | 278.180707431443 | 212.693866338768 | 62.9114576227844 | 41.8199199147677 | 73.8502629995884 | 57.7504443042924 |
| YDL006W | 990.381137669594 | 0.315833175559744 | 0.394915674107339 | 0.799748392548987 | 0.423856588944023 | 0.521221392552894 | 967.585069326758 | 1031.31793329379 | 647.402790653072 | 699.612410240801 | 1280.59498605669 | 1315.77363644645 |
| YDL007W | 1662.84685755054 | -0.500392935136204 | 0.381302192076746 | -1.312326405497 | 0.189410026411888 | 0.272276912967089 | 1706.12461052382 | 1622.40902695619 | 2517.92136206539 | 1883.63889282766 | 1082.61342993014 | 1164.37382300006 |
| YDL008W | 845.202706247228 | 0.720374261843395 | 0.307574764441441 | 2.34211107387696 | 0.0191750073611641 | 0.0410119719049807 | 661.435105985088 | 683.423314708678 | 569.860761490105 | 848.59587493716 | 1088.89855869606 | 1219.00262166628 |
| YDL009C | 13.9569368406928 | 1.44046262883388 | 0.76235823079696 | 1.88948262200572 | 0.0588271891813597 | 0.103566729996708 | 9.07111002493835 | 5.77076381539294 | 7.31528577009121 | 5.22748998934596 | 25.1405150636897 | 31.2164563806986 |
| YDL010W | 362.539336584719 | -1.68698287633509 | 0.450767786570321 | -3.74246547023812 | 0.000182223578421539 | 0.000900629198031242 | 589.622151620993 | 537.505429662314 | 534.747389793667 | 273.571976109105 | 147.700525999177 | 92.0885463230609 |
| YDL011C | 1.5532641185284 | 1.62902138326236 | 1.85836402514896 | 0.876588957393198 | 0.38070993920842 | 0.479048848867179 | 0 | 0 | 2.19458573102736 | 0.87124833155766 | 1.5712821914806 | 4.68246845710479 |
| YDL012C | 927.771672924408 | -0.363665161310626 | 0.451919213288471 | -0.804712768603821 | 0.420985449556774 | 0.518482749119454 | 935.080258404062 | 854.897439508925 | 1343.81799596576 | 1294.67502069468 | 516.951840997119 | 621.207481975902 |
| YDL013W | 531.13352217156 | -1.10954106034519 | 0.313848897888558 | -3.53527148831718 | 0.00040735640148929 | 0.00172024604956789 | 685.624732718257 | 777.404325416506 | 716.16647689193 | 415.585454153004 | 304.828745147237 | 287.191398702427 |
| YDL014W | 62517.4721789256 | 1.68361494356006 | 0.407402336079197 | 4.13256084823424 | 3.58743752584165e-05 | 0.000250847965976332 | 32243.2605836434 | 33115.9403520477 | 23689.8214378634 | 53918.9454951089 | 114246.356860363 | 117890.508344527 |
| YDL015C | 8794.03144476493 | 1.34154459938817 | 0.368947856566457 | 3.63613604337751 | 0.000276758208165236 | 0.00124899990230141 | 4804.66460987568 | 5186.26788037671 | 4937.81789481157 | 7759.33764085252 | 14628.6372026844 | 15447.4634399887 |
| YDL016C | 1487.60822114431 | 1.33212291853743 | 0.40727818246168 | 3.27079371275374 | 0.00107246110096192 | 0.00376179166132612 | 802.037311371633 | 831.814384247353 | 902.706264029255 | 1152.66154265078 | 2559.6186899219 | 2676.8111346449 |
| YDL017W | 502.98353547876 | -0.474145537409749 | 0.352546404699528 | -1.34491667221471 | 0.178652145544729 | 0.258660030869617 | 693.183991072373 | 658.691469785565 | 403.072245932026 | 437.366662441945 | 455.671835529375 | 369.915008111278 |
| YDL018C | 459.295819283566 | -0.176150345762904 | 0.300174299830311 | -0.58682687312832 | 0.557319986549145 | 0.647041772737786 | 479.25697965091 | 497.110082954563 | 486.466503711065 | 501.839038977212 | 433.673884848647 | 357.428425558999 |
| YDL019C | 624.137096168711 | -1.13276147846217 | 0.504046887897398 | -2.24733354309044 | 0.0246187187079898 | 0.0504758193185146 | 1015.9643227931 | 958.771188185998 | 599.12190457047 | 677.831201951859 | 221.550788998765 | 271.583170512078 |
| YDL020C | 484.501099996333 | -2.12593812847543 | 0.532269749628752 | -3.99409910098823 | 6.49406993582775e-05 | 0.000400976130265874 | 839.077677306798 | 821.097251447338 | 707.38813396782 | 338.91560097593 | 111.561035595123 | 88.966900684991 |
| YDL021W | 46.1598249180187 | -0.912460848626603 | 0.456895584497478 | -1.99708834925639 | 0.0458155863448357 | 0.0844212807040749 | 74.8366577057414 | 40.3953467077506 | 65.8375719308209 | 34.8499332623064 | 25.1405150636897 | 35.8989248378034 |
| YDL022W | 6291.99477827968 | 2.30420914831842 | 0.424320339688661 | 5.430352808468 | 5.62427511139492e-08 | 1.79308572858333e-06 | 1740.89719895275 | 1714.74124800247 | 2900.51080784116 | 6201.54562402742 | 12816.9488359073 | 12377.324954947 |
| YDL023C | 7.06753880284136 | 0.515525614697943 | 1.15914556300672 | 0.44474622614326 | 0.656503159000861 | 0.734517085470039 | 10.5829616957614 | 0 | 6.58375719308209 | 1.74249666311532 | 12.5702575318448 | 10.9257597332445 |
| YDL024C | 99.4880847008707 | -0.0156255983829838 | 0.338745902168408 | -0.0461277857029706 | 0.963208399785908 | 0.974711202800322 | 84.6636935660913 | 105.522538338614 | 109.729286551368 | 94.9660681397849 | 105.2759068292 | 96.7710147801657 |
| YDL025C | 279.247585946039 | -1.58742685256256 | 0.397340003103685 | -3.99513474647133 | 6.46574684266876e-05 | 0.000399994329171823 | 415.759209476341 | 396.533913600572 | 446.963960552573 | 204.74335791605 | 116.274882169565 | 95.2101919611307 |
| YDL025W-A | 5.08822177661321 | 2.16267659163013 | 1.29022906822242 | 1.67619583599191 | 0.0936998395605082 | 0.151958440385211 | 2.26777750623459 | 2.47318449231126 | 0.731528577009121 | 0 | 12.5702575318448 | 12.4865825522794 |
| YDL026W | 26.9141855016299 | 1.14730209907701 | 0.529171365699285 | 2.16811069805503 | 0.0301502650804761 | 0.0594694355645531 | 9.82703586034988 | 18.1366862769492 | 21.9458573102736 | 27.0086982782874 | 42.4246191699763 | 42.1422161139431 |
| YDL027C | 477.738434832976 | -0.763139440063732 | 0.318270513492295 | -2.39776984581454 | 0.0164952260693432 | 0.0362186348061952 | 577.527338254409 | 522.666322708446 | 704.462019659784 | 416.456702484561 | 339.39695335981 | 305.921272530846 |
| YDL028C | 384.777396223263 | -0.849155607783308 | 0.358741311556755 | -2.36704159913561 | 0.0179309172575081 | 0.0387110650815798 | 562.408821546178 | 583.671540185457 | 337.966202578214 | 240.464539509914 | 295.401051998354 | 288.752221521462 |
| YDL029W | 5073.5061196958 | 1.42264081631565 | 0.356973649108518 | 3.98528244274741 | 6.73999063298932e-05 | 0.000413386092156679 | 2491.5315535164 | 2283.57368123406 | 3494.51201237257 | 5380.82969570011 | 8095.24585050807 | 8695.3439248436 |
| YDL030W | 420.623815564408 | -0.508475181718116 | 0.299145701355702 | -1.69975760779363 | 0.0891765284384627 | 0.145834647827247 | 548.80215650877 | 523.490717539216 | 409.656003125108 | 343.271842633718 | 359.823621849058 | 338.69855173058 |
| YDL031W | 816.782488577607 | -2.97191567068165 | 0.680729622206881 | -4.3657798540438 | 1.26670007856961e-05 | 0.000110836256874841 | 2102.98567411488 | 1940.62543163357 | 304.315888035794 | 308.421909371412 | 128.84513970141 | 115.500888608585 |
| YDL032W | 4.0910430742077 | 0.781355636660842 | 0.989658457416487 | 0.789520496495911 | 0.429807855033686 | 0.52692917806441 | 3.02370334164612 | 1.64878966154084 | 4.38917146205473 | 6.09873832090362 | 3.14256438296121 | 6.24329127613972 |
| YDL033C | 332.986283782058 | 0.413955308867826 | 0.404751100780237 | 1.02274041520788 | 0.306430597110415 | 0.401753470152906 | 247.943674014982 | 227.532973292636 | 381.857917198761 | 503.581535640327 | 262.404125977261 | 374.597476568383 |
| YDL034W | 1.15697800642882 | -0.402609150512264 | 1.96992969441551 | -0.204377421008277 | 0.838058573220854 | 0.879149244427806 | 1.51185167082306 | 0.82439483077042 | 1.46305715401824 | 0 | 3.14256438296121 | 0 |
| YDL035C | 555.423563716876 | -0.981071694263266 | 0.299826809407017 | -3.27212798683208 | 0.00106741243530206 | 0.00374816580335075 | 809.596569725748 | 748.550506339541 | 653.986547846154 | 382.478017553813 | 417.961062933841 | 319.968677902161 |
| YDL036C | 425.917746013636 | -1.67099890566057 | 0.544784970333283 | -3.06726322614646 | 0.00216028530078244 | 0.0066470316947152 | 752.902132069883 | 727.94063557028 | 465.98370355481 | 372.023037575121 | 146.129243807696 | 90.5277235040259 |
| YDL037C | 1692.31998675338 | -0.891071863778328 | 0.498184096462887 | -1.78863972195208 | 0.0736728563717848 | 0.12475761110552 | 2635.15746224459 | 2751.82994511166 | 1211.4113235271 | 1897.57886613258 | 755.78673410217 | 902.15558940219 |
| YDL039C | 6024.02208608994 | -0.656341517654024 | 0.445442553728187 | -1.47345939933375 | 0.140627203801758 | 0.21316803813999 | 8702.21821725753 | 8665.21406622788 | 4747.6204647892 | 7077.15019724287 | 3755.36443763864 | 3196.56513338354 |
| YDL040C | 3147.04000869613 | -0.318394230887497 | 0.392731059066805 | -0.810718234621055 | 0.417527499250068 | 0.514913269852631 | 3338.92441501273 | 3490.48771348196 | 3651.05912785252 | 4224.68315972309 | 2158.94173109435 | 2018.14390501216 |
| YDL041W | 6.88659571173756 | 0.0829830150700594 | 0.812247674075062 | 0.102164669372006 | 0.918625970734865 | 0.94218048280499 | 8.31518418952682 | 4.1219741538521 | 7.31528577009121 | 4.3562416577883 | 6.28512876592241 | 10.9257597332445 |
| YDL042C | 1111.04806604411 | 0.367698836914658 | 0.30287426797345 | 1.21403128557257 | 0.224735777531917 | 0.312658977598952 | 904.087299152189 | 845.00470153968 | 1160.93585171348 | 1108.22787774134 | 1304.1642189289 | 1343.86844718907 |
| YDL043C | 388.575533763474 | -1.7479422801175 | 0.446143467819528 | -3.91789279950764 | 8.93264126938048e-05 | 0.000512711317066045 | 698.475471920253 | 643.027968000927 | 457.205360630701 | 280.541962761566 | 136.701550658813 | 115.500888608585 |
| YDL044C | 95.9115520809959 | -1.99934550203673 | 0.509081134915793 | -3.92736121005042 | 8.58829505241462e-05 | 0.000498725159040128 | 203.344049725701 | 184.664442092574 | 71.6898005468939 | 29.6224432729604 | 45.5671835529375 | 40.5813932949082 |
| YDL045C | 737.874902509143 | -0.0248754375016085 | 0.297010227182191 | -0.0837527977996173 | 0.933252977995038 | 0.952329136157193 | 677.30954852873 | 680.950130216367 | 874.908178102909 | 803.290961696162 | 718.075961506636 | 672.714635004055 |
| YDL045W-A | 338.291589478708 | -0.966848617810067 | 0.345390827720083 | -2.79928863251005 | 0.00512153347278406 | 0.013640477901046 | 516.297345586075 | 360.260541046673 | 467.446760708828 | 284.898204419355 | 191.696427360634 | 209.150257750681 |
| YDL046W | 8283.09511171053 | 1.63175498069932 | 0.408802067825992 | 3.99155265866679 | 6.5642108384296e-05 | 0.000404918752868646 | 3748.63621780577 | 3918.3486306518 | 4457.20361971657 | 6736.49209960382 | 15348.2844463825 | 15489.6056561026 |
| YDL047W | 3791.26423248849 | 1.0775791466121 | 0.264614078786989 | 4.07226687087778 | 4.65577909820314e-05 | 0.000308152285636467 | 2356.22082897774 | 2314.07628997257 | 2643.01274873395 | 5378.21595070543 | 4875.68864016431 | 5180.37093637693 |
| YDL048C | 4941.76852474914 | -2.10585181755545 | 0.650734474338855 | -3.23611534442676 | 0.00121168411275356 | 0.00415066259900687 | 10005.434357507 | 8856.47366696662 | 5201.16818253485 | 4016.45480848081 | 837.493408059162 | 733.586724946417 |
| YDL049C | 245.757213520718 | 1.16453662575759 | 0.351288279490931 | 3.31504548755563 | 0.000916282131739567 | 0.00331695161798921 | 121.704059501256 | 150.039859200216 | 183.613672829289 | 412.971709158331 | 326.826695827966 | 279.387284607252 |
| YDL050C | 4.57050466231848 | 0.462481137396278 | 1.07576331411939 | 0.429909749966573 | 0.667261292793889 | 0.743325155784924 | 6.80333251870377 | 1.64878966154084 | 2.92611430803648 | 3.48499332623064 | 10.9989753403642 | 1.56082281903493 |
| YDL051W | 3217.23115765936 | -1.80049039260628 | 0.784918003188769 | -2.29385793839827 | 0.0217986601297135 | 0.045682841274115 | 5837.25930104783 | 5513.55262819257 | 3648.8645421215 | 3572.1181593864 | 403.819523210515 | 327.772791997335 |
| YDL052C | 2653.90285010317 | 1.26950749907435 | 0.293006230452908 | 4.33269796724813 | 1.47293115293801e-05 | 0.000124811534538432 | 1446.84204897767 | 1475.66674707905 | 1745.42718474376 | 3148.69147024938 | 3967.48753348852 | 4139.30211608063 |
| YDL053C | 225.820566138465 | -3.81574802130902 | 0.898484884612173 | -4.24686946509519 | 2.16778171471926e-05 | 0.000166394687041621 | 492.863644688317 | 488.041739816088 | 286.027673610566 | 80.1548465033047 | 4.71384657444181 | 3.12164563806986 |
| YDL054C | 1472.37534777729 | 0.796270351617214 | 0.394239369313227 | 2.0197636603476 | 0.0434079082271339 | 0.0807705660163947 | 1052.24876289285 | 1137.66486646318 | 1037.30752219893 | 1057.695474511 | 2275.21661326391 | 2274.11884733389 |
| YDL055C | 92724.4013765668 | 1.62094913572251 | 0.355053043652338 | 4.56537175135363 | 4.98609950078934e-06 | 5.38516921705934e-05 | 38463.7742832449 | 42748.9939496001 | 55287.4667931954 | 97646.8992559878 | 161816.925207439 | 160382.348769934 |
| YDL056W | 32.9638709431602 | -2.35386659941199 | 0.696305075719927 | -3.38051047089994 | 0.000723513182859173 | 0.00274083817506651 | 67.2773993516261 | 71.7223502770265 | 27.7980859263466 | 20.0387116258262 | 3.14256438296121 | 7.80411409517465 |
| YDL057W | 299.33859866419 | 0.314175666295326 | 0.360631586799232 | 0.871181776071743 | 0.383654908785762 | 0.481907082617576 | 222.998121446401 | 222.586604308013 | 355.522888426433 | 392.061749200947 | 292.258487615392 | 310.603740987951 |
| YDL058W | 323.178925294754 | 0.0278754205428868 | 0.297484498925254 | 0.093703774965064 | 0.925344471486046 | 0.947411509756778 | 303.126260000023 | 370.153279015918 | 286.759202187575 | 311.906902697642 | 333.111824593888 | 334.016083273475 |
| YDL059C | 138.17592253602 | -0.683049073924252 | 0.575030079163173 | -1.18784929462869 | 0.234892789426151 | 0.323457925129292 | 146.649612069837 | 150.039859200216 | 215.800930217691 | 203.872109584492 | 47.1384657444181 | 65.554558399467 |
| YDL060W | 2833.8083942944 | -0.333951534015768 | 0.308885620241547 | -1.08114950043521 | 0.279630617311041 | 0.371840011456351 | 3471.96736204515 | 3600.95662080519 | 2408.19207551403 | 2512.68018821229 | 2400.91918858236 | 2608.13493060737 |
| YDL061C | 26252.12080685 | 0.437846893253614 | 0.498408390798681 | 0.878490212718892 | 0.379677746774352 | 0.477936803992734 | 28402.4014139174 | 27146.4973824391 | 11346.7397579885 | 16026.6130590031 | 36390.8955546908 | 38199.5776730609 |
| YDL062W | 3.3837007094932 | -0.665589970866592 | 1.1165838941735 | -0.596094905487834 | 0.551111827350417 | 0.641105521700991 | 6.80333251870377 | 3.29757932308168 | 2.19458573102736 | 1.74249666311532 | 3.14256438296121 | 3.12164563806986 |
| YDL063C | 2106.38011897765 | -0.429178745633535 | 0.423972111559161 | -1.01228060509741 | 0.311403905516183 | 0.406535810161001 | 2534.61932613486 | 2696.59549145004 | 2022.67651543022 | 2766.21345269557 | 1508.43090382138 | 1109.74502433384 |
| YDL064W | 1943.38204754281 | 1.13488236043606 | 0.366048956922246 | 3.10035676642324 | 0.0019328767323163 | 0.00604552023123701 | 993.28654773075 | 985.151822770651 | 1669.34821273481 | 2135.42966064782 | 2900.58692547319 | 2976.48911589961 |
| YDL065C | 61.2966863839796 | -2.09704734457678 | 0.63645127048364 | -3.29490636884617 | 0.000984544783599538 | 0.00352873071845057 | 154.208870423952 | 104.698143507843 | 39.5025431584925 | 27.0086982782874 | 32.9969260210927 | 9.36493691420958 |
| YDL066W | 3849.53347127149 | 0.876531510584301 | 0.336104728534494 | 2.60791186844116 | 0.00910964052486734 | 0.0220715142889938 | 2206.54751356625 | 2375.90590228035 | 3561.81264145741 | 4939.10679160037 | 4393.30500737977 | 5620.52297134478 |
| YDL067C | 1696.22254526014 | 0.603369355377993 | 0.41161142638991 | 1.46587124820591 | 0.142683366168846 | 0.215515564499183 | 1551.15981426446 | 1394.05165883278 | 1093.63522262864 | 1141.33531434053 | 2488.91099130528 | 2508.24227018913 |
| YDL068W | 8.77186632764556 | 1.0826068775007 | 0.760072078895573 | 1.42434764749389 | 0.154345861015421 | 0.229717435853781 | 4.53555501246918 | 3.29757932308168 | 8.77834292410945 | 7.84123498401894 | 12.5702575318448 | 15.6082281903493 |
| YDL069C | 138.895717248696 | -1.70192423989955 | 0.383804184937211 | -4.43435560812859 | 9.23480879883798e-06 | 8.5891130797207e-05 | 269.865523241916 | 206.098707692605 | 162.399344096025 | 77.5411015086317 | 61.2800054677435 | 56.1896214852575 |
| YDL070W | 923.614430422299 | -1.90568270238741 | 0.67779700939719 | -2.81158322619668 | 0.00492983351142046 | 0.0132063759623743 | 1573.8375893268 | 1279.46077735569 | 1523.04249733299 | 883.445808199467 | 142.986679424735 | 138.913230894109 |
| YDL071C | 31.3628227509483 | 0.63025954189111 | 0.584371980843124 | 1.07852457433325 | 0.280799721056089 | 0.373240496099321 | 18.8981458852882 | 27.2050294154238 | 27.0665573493375 | 14.8112216364802 | 45.5671835529375 | 54.6287986662226 |
| YDL072C | 7598.78862090947 | 1.06470329917367 | 0.398196906387218 | 2.67381107712154 | 0.00749946864213803 | 0.0187268623712171 | 4701.1027704243 | 4825.18294449927 | 5219.45639696008 | 5722.35904167071 | 13140.6329673523 | 11983.9976045502 |
| YDL073W | 565.470494185876 | -0.989242807613624 | 0.331650697586073 | -2.98278524608526 | 0.00285638340648093 | 0.00836521561516015 | 791.454349675871 | 849.951070524303 | 613.752476110653 | 303.194419382066 | 383.392854721267 | 451.077794701095 |
| YDL074C | 493.091952897847 | -1.54299663782736 | 0.49493308136578 | -3.117586388789 | 0.00182338499285254 | 0.00577695894068696 | 1001.60173192028 | 896.117181047446 | 304.315888035794 | 194.288377937358 | 284.402076657989 | 277.826461788218 |
| YDL075W | 30823.5911063721 | -1.41899534305608 | 0.427802491847594 | -3.31694034068789 | 0.000910090477770066 | 0.00329824573823254 | 58007.4808319745 | 53095.1490757689 | 23501.818593572 | 20460.3958183001 | 14686.7746437692 | 15189.9276748479 |
| YDL076C | 614.817020548142 | -0.737082893106375 | 0.308631266029471 | -2.38823144067325 | 0.0169296766065655 | 0.0369458208560764 | 662.946957655911 | 710.628344124102 | 931.96740710962 | 459.147870730887 | 499.667736890832 | 424.543806777501 |
| YDL077C | 600.811295177842 | -1.36838887744061 | 0.323933856842172 | -4.22428483018162 | 2.39700739414693e-05 | 0.000182036882291347 | 830.762493117271 | 887.048837908971 | 882.223463873 | 417.327950816119 | 329.969260210927 | 257.535765140763 |
| YDL078C | 5789.86593895896 | 1.40598678927428 | 0.332753595291283 | 4.22530908507096 | 2.38613044717046e-05 | 0.000181424794330316 | 2755.34967007502 | 2755.12752443474 | 4006.58201627896 | 6926.42423588339 | 8403.21716003827 | 9892.49502704339 |
| YDL079C | 86.8038892116451 | -0.628030211496762 | 0.357157275508411 | -1.75841360253061 | 0.0786771603230231 | 0.131742306937147 | 117.924430324199 | 97.2785900309095 | 100.21941505025 | 56.6311415512479 | 73.8502629995884 | 74.9194953136766 |
| YDL080C | 883.370471784278 | -0.437427782834942 | 0.573200274409957 | -0.763132542609514 | 0.445384353986899 | 0.541899724102707 | 960.025810972642 | 978.556664124488 | 1111.92343705386 | 1502.90337193696 | 345.682082125733 | 401.131464491977 |
| YDL081C | 90468.7672274321 | 0.677186082240742 | 0.464734986255101 | 1.45714461417592 | 0.145076465381127 | 0.218496828123119 | 85875.442680256 | 83321.5855459663 | 39655.4326310874 | 63006.9368415868 | 132102.407684349 | 138850.797981347 |
| YDL082W | 21365.7389433578 | -0.640049285866146 | 0.386244093835847 | -1.65711086869891 | 0.0974970621437494 | 0.156667137637333 | 28511.2547342167 | 26952.7645972081 | 22624.7158297381 | 24631.9328297982 | 12832.6616578221 | 12641.1040113639 |
| YDL083C | 77376.7285014201 | 1.07161466722073 | 0.405781953351966 | 2.64086329707037 | 0.00826950789075459 | 0.0203421049719097 | 56166.8014227475 | 56564.2025236508 | 36943.6561961146 | 62451.9516543846 | 122632.289916295 | 129501.469295328 |
| YDL084W | 18412.1117471891 | 1.04596286039782 | 0.296128594161224 | 3.53212381722367 | 0.000412236287991634 | 0.00173629934248929 | 10886.8438815968 | 11670.1332243861 | 13488.6554314712 | 20507.4432282042 | 26172.8474634924 | 27746.747253984 |
| YDL085C-A | 191.02732368992 | -1.43531504164934 | 0.347627048689822 | -4.12889344214993 | 3.6451327478889e-05 | 0.000253506453758073 | 328.071812568604 | 253.089213046519 | 256.035001953192 | 111.51978643938 | 117.846164361045 | 79.6019637707814 |
| YDL085W | 89.8722398965061 | 0.463278794457507 | 0.397855737536579 | 1.16443914401238 | 0.24424609204986 | 0.333675187272188 | 60.4740668329224 | 73.3711399385673 | 92.1726007031492 | 78.4123498401894 | 102.133342446239 | 132.669939617969 |
| YDL086C-A | 87.6596729311781 | 2.47560546557169 | 0.479622756987668 | 5.16156798130273 | 2.44889926628626e-07 | 5.61242394123968e-06 | 25.701478403992 | 25.556239753883 | 28.5296145033557 | 74.9273565139587 | 199.552838318037 | 171.690510093842 |
| YDL086W | 7610.8922760204 | 1.83688989869492 | 0.397002138975359 | 4.62690176792455 | 3.71176451891031e-06 | 4.3779786633301e-05 | 2793.90188768101 | 2762.54707791168 | 4430.13706236724 | 7663.50032438117 | 13842.9961069441 | 14172.2711968372 |
| YDL087C | 163.398710815583 | -0.764994505190265 | 0.565326327430449 | -1.35319101211394 | 0.175994615864989 | 0.255329532985867 | 228.289602294282 | 195.381574892589 | 195.318130061435 | 236.108297852126 | 64.4225698507047 | 60.8720899423623 |
| YDL088C | 944.095138694116 | -0.972885952937783 | 0.292386758471898 | -3.32739402434769 | 0.000876623251098995 | 0.00319493703286787 | 1294.14503022454 | 1321.50491372498 | 1138.25846582619 | 742.303578487126 | 598.65851495411 | 569.700328947749 |
| YDL089W | 338.749356155685 | -1.07592490238045 | 0.355023001940449 | -3.03057800902975 | 0.00244086124931922 | 0.00734882956784283 | 459.60290793021 | 419.616968862144 | 501.097075251248 | 293.610687734931 | 171.269758871386 | 187.298738284192 |
| YDL090C | 1046.40614598938 | 0.906524103712954 | 0.265354278448017 | 3.41627845239564 | 0.000634832966895938 | 0.00246433050440617 | 740.807318703299 | 712.277133785642 | 730.797048432112 | 1288.57628237378 | 1349.73140248184 | 1456.24769015959 |
| YDL091C | 216.977567975997 | -2.41447138574602 | 0.628978751624884 | -3.83871693520417 | 0.000123678913618413 | 0.000658258019588909 | 424.074393665868 | 361.909330708214 | 312.362702382895 | 139.399733049226 | 18.8553862977672 | 45.263861752013 |
| YDL092W | 776.923418249134 | -1.82423300934504 | 0.412472975681256 | -4.42267279773194 | 9.74873238490722e-06 | 8.98166474374857e-05 | 1390.90353715721 | 1258.85090658643 | 987.563578962313 | 518.392757276808 | 252.976432828377 | 252.853296683659 |
| YDL093W | 1395.90050998068 | 0.76175313818641 | 0.275251115021065 | 2.76748429566983 | 0.00564907670674472 | 0.0148066967812112 | 1054.51654039908 | 1099.74270424774 | 952.450207265876 | 1576.08823178781 | 1830.5437530749 | 1862.06162310867 |
| YDL094C | 14.5633588025245 | 0.219102608016244 | 0.587208393101263 | 0.373125811194698 | 0.709054809075689 | 0.776511889015866 | 12.0948133665845 | 14.0147121230971 | 13.8990429631733 | 11.3262283102496 | 21.9979506807285 | 14.0474053713144 |
| YDL095W | 9798.41907175126 | 0.992848760356535 | 0.258410732366315 | 3.84213438530526 | 0.00012196902081688 | 0.000652575338723298 | 6250.75073301794 | 6460.78228874778 | 6950.25301016366 | 13487.7954208441 | 12622.1098441637 | 13018.8231335704 |
| YDL096C | 0.950986221946331 | -0.0778721415687003 | 2.31971504088934 | -0.0335697015349115 | 0.97322028331706 | 0.98083546550264 | 2.26777750623459 | 0.82439483077042 | 0 | 2.61374499467298 | 0 | 0 |
| YDL097C | 2076.3346889775 | -0.779120535810324 | 0.678708548600211 | -1.14794566447882 | 0.25099099532795 | 0.341296877092905 | 2302.55009466352 | 2244.82712418785 | 3325.52891108346 | 3452.757137963 | 581.374410847823 | 550.97045511933 |
| YDL098C | 115.493117082892 | -2.85387137221079 | 0.659707989095179 | -4.32596151537443 | 1.5186793789226e-05 | 0.00012784699608185 | 249.455525685805 | 215.16705083108 | 146.305715401824 | 60.1161348774785 | 9.42769314888362 | 12.4865825522794 |
| YDL099W | 111.137797601886 | -0.432861149628901 | 0.341422167501398 | -1.26781794163125 | 0.204862973457383 | 0.289987443082625 | 111.877023640906 | 152.513043692528 | 118.507629475478 | 89.7385781504389 | 97.4194958717974 | 96.7710147801657 |
| YDL100C | 4129.66485629434 | -0.342238659036198 | 0.693230784134337 | -0.493686470463893 | 0.621527621693285 | 0.703351434089042 | 4057.05395865368 | 4131.04249699057 | 5664.95730035863 | 8339.58902966992 | 1500.57449286398 | 1084.77185922928 |
| YDL101C | 344.645727499788 | -1.86394436170905 | 0.411553659442745 | -4.52904334329787 | 5.92513447347357e-06 | 6.15084564962848e-05 | 599.449187481343 | 597.686252308554 | 427.212688973327 | 219.55457955253 | 116.274882169565 | 107.69677451341 |
| YDL102W | 1108.10616945504 | 0.431711867399655 | 0.308440428051954 | 1.39966044699866 | 0.161615023458317 | 0.238606316155791 | 933.568406733239 | 895.292786216676 | 1000.73109334848 | 984.510614660155 | 1418.86781890699 | 1415.66629686468 |
| YDL103C | 1062.21041625684 | -0.700325759233123 | 0.701008790632458 | -0.999025645029761 | 0.317782268338221 | 0.413104119519205 | 1270.71132932678 | 1281.10956701723 | 1395.02499635639 | 1884.51014115922 | 279.688230083547 | 262.218233597868 |
| YDL104C | 399.154666764581 | 1.12081340680414 | 0.358221469537165 | 3.12882811924218 | 0.00175504938090955 | 0.00562594226633026 | 233.581083142163 | 252.264818215748 | 267.739459185338 | 366.795547585775 | 606.514925911513 | 668.03216654695 |
| YDL105W | 80.8521884937679 | -1.92972456104853 | 0.454025117397616 | -4.25025948368086 | 2.13523007853862e-05 | 0.000164287714525552 | 151.941092917717 | 110.468907323236 | 123.628329514541 | 50.5324032303443 | 23.5692328722091 | 24.9731651045589 |
| YDL106C | 301.525142320198 | -1.47075968869221 | 0.39923127290672 | -3.6839791582055 | 0.000229621024376316 | 0.00107312002683852 | 526.124381446425 | 489.690529477629 | 313.094230959904 | 138.528484717668 | 218.408224615804 | 123.305002703759 |
| YDL107W | 101.539092961024 | -1.53458718868008 | 0.369306020629309 | -4.15532675601957 | 3.248229912059e-05 | 0.000231400449487389 | 184.445903840413 | 126.132409107874 | 142.648072516779 | 55.7598932196902 | 53.4235945103405 | 46.8246845710479 |
| YDL108W | 922.069882999165 | 0.705584176621757 | 0.287492405617314 | 2.45427066188654 | 0.0141170680527594 | 0.0318103282924319 | 600.205113316754 | 694.964842339464 | 807.60754901807 | 1113.45536773069 | 1161.17753950417 | 1155.00888608585 |
| YDL109C | 150.201123009012 | -0.952290183437404 | 0.770647488867871 | -1.2357014032919 | 0.21656957574719 | 0.30299979748249 | 161.012202942656 | 176.42049378487 | 258.22958768422 | 249.177022825491 | 26.7117972551703 | 29.6556335616637 |
| YDL110C | 299.692966650637 | -1.1365884191213 | 0.457735245685661 | -2.48306948139586 | 0.0130255678153083 | 0.0297147207688933 | 399.128841097288 | 394.885123939031 | 444.037846244536 | 311.035654366084 | 135.130268467332 | 113.94006578955 |
| YDL111C | 3368.84103213875 | 0.973455346047388 | 0.3038826799333 | 3.20339200069269 | 0.00135818970989473 | 0.00454376193855691 | 2196.7204777059 | 2336.33495040337 | 2286.75833173051 | 3467.56835959949 | 4748.41478265438 | 5177.24929073886 |
| YDL112W | 3516.83239515907 | 0.0880692876273315 | 0.283756060613416 | 0.31036971487744 | 0.756279821793024 | 0.813633724238045 | 3438.70662528705 | 3532.53184985125 | 3258.22828199862 | 4167.18076984029 | 3609.23519383095 | 3095.11165014627 |
| YDL113C | 202.068659872932 | -2.81927916635487 | 0.518343185810918 | -5.4390204087361 | 5.35743185932971e-08 | 1.73376186804439e-06 | 413.491431970107 | 399.831492923653 | 250.914301914129 | 87.124833155766 | 26.7117972551703 | 34.3381020187685 |
| YDL114W | 7.55136096343387 | -0.990105112180272 | 0.782923363138528 | -1.26462583542176 | 0.206005487255334 | 0.29119300656812 | 8.31518418952682 | 10.7171328000155 | 10.9729286551368 | 4.3562416577883 | 3.14256438296121 | 7.80411409517465 |
| YDL114W-A | 9.04208692548913 | 0.800649276555379 | 0.699341771467042 | 1.14486122411338 | 0.25226664880615 | 0.342814352882803 | 8.31518418952682 | 5.77076381539294 | 5.85222861607297 | 13.9399733049226 | 12.5702575318448 | 7.80411409517465 |
| YDL115C | 351.569045706642 | -0.703469852317333 | 0.294579558301174 | -2.38804707419011 | 0.016938172047258 | 0.0369518387480833 | 466.406240448914 | 455.890341416042 | 384.784031506798 | 274.443224440663 | 284.402076657989 | 243.488359769449 |
| YDL116W | 1686.7698513399 | -0.149159854938746 | 0.300618392084901 | -0.496176743892038 | 0.619769717466429 | 0.702449310187223 | 1811.95422748144 | 2040.37720615679 | 1468.90938263432 | 1448.88597538039 | 1786.54785171345 | 1563.944464673 |
| YDL117W | 1501.87354320852 | -1.49607145626248 | 0.363623919898281 | -4.11433729849507 | 3.88293317275722e-05 | 0.000267731152382832 | 2421.23045082313 | 2442.68188357275 | 1790.78195651833 | 1069.02170282125 | 680.365188911101 | 607.160076604588 |
| YDL118W | 1.56674234916543 | 0.913696833003771 | 1.66594365494704 | 0.548456023882041 | 0.583378817168881 | 0.670167603026685 | 0.75592583541153 | 1.64878966154084 | 0.731528577009121 | 0 | 3.14256438296121 | 3.12164563806986 |
| YDL119C | 439.186106695671 | -0.466854532170548 | 0.363516417187163 | -1.28427358462377 | 0.199046246071563 | 0.282783548356688 | 476.233276309264 | 474.851422523762 | 579.370632991224 | 507.937777298116 | 307.971309530198 | 288.752221521462 |
| YDL120W | 1448.47110407319 | 0.60671189110209 | 0.278480989599624 | 2.17864742571609 | 0.0293578701602237 | 0.0581379716580076 | 1095.33653551131 | 1079.95722830925 | 1269.20208111083 | 1617.03690337102 | 1698.55604899053 | 1930.73782714621 |
| YDL121C | 1373.57313987284 | -0.444782209280475 | 0.41446730364772 | -1.07314185067424 | 0.283207476082907 | 0.375820347408597 | 1920.8075477807 | 1907.64963840275 | 921.726007031492 | 859.050854915852 | 1271.16729290781 | 1361.03749819846 |
| YDL122W | 688.279288312251 | -3.19360123502868 | 0.449199967537995 | -7.10953131304169 | 1.16437682425701e-12 | 7.49858674821515e-10 | 1480.10278573577 | 1408.06637095588 | 836.868692098435 | 207.357102910723 | 92.7056492973556 | 104.57512887534 |
| YDL123W | 870.172826424516 | 1.14871544126335 | 0.44929207365351 | 2.55672313985498 | 0.0105663277932281 | 0.0249439703036617 | 374.939214364119 | 428.685312000618 | 818.580477673206 | 765.827283439183 | 1423.58166548143 | 1409.42300558854 |
| YDL124W | 5032.87225416153 | 0.356254254060399 | 0.409610843614894 | 0.869738337287137 | 0.384443416241355 | 0.482615126821507 | 3507.4958763095 | 3347.86740775867 | 6389.17059159766 | 7497.09189305366 | 4476.58296352824 | 4979.02479272143 |
| YDL125C | 10952.0319964842 | 1.24933079557783 | 0.401236964206895 | 3.11369815601938 | 0.00184758398002774 | 0.00582971133335552 | 6894.79954478856 | 6977.67784764083 | 5583.75762831062 | 8648.01093904133 | 19215.2099196163 | 18392.7360995076 |
| YDL126C | 14890.9319982887 | 1.54566354778549 | 0.394512090224204 | 3.91791173473816 | 8.93193979858959e-05 | 0.000512711317066045 | 5462.32008668371 | 5906.78896247006 | 11426.4763728825 | 21156.5232352146 | 20645.0767138637 | 24748.4066186178 |
| YDL127W | 237.855190338382 | -1.52086152007237 | 0.547730664063174 | -2.7766594420519 | 0.00549206906185745 | 0.0144599038259861 | 384.010324389057 | 348.719013415887 | 327.724802500086 | 233.494552857453 | 76.9928273825496 | 56.1896214852575 |
| YDL128W | 6963.4322355678 | 1.33259963394488 | 0.418968735022136 | 3.18066605584417 | 0.00146936901008672 | 0.00483285823542314 | 4355.64466364123 | 4423.70266191407 | 3094.36588074858 | 5448.7870655616 | 12427.2708524201 | 12030.8222891212 |
| YDL129W | 280.83829877033 | -1.05017987815221 | 0.3993418512214 | -2.62977665611607 | 0.00854409802050346 | 0.0209057717522957 | 383.254398553645 | 328.933537477397 | 425.749631819308 | 258.760754472625 | 171.269758871386 | 117.06171142762 |
| YDL130W | 86791.1889703602 | 1.08051851371743 | 0.499137355895842 | 2.16477188283801 | 0.0304051590691538 | 0.059880496760046 | 72311.8654154669 | 64822.9899383089 | 30049.7308863807 | 59043.628181331 | 145294.89296402 | 149224.026436654 |
| YDL130W-A | 159.06338433907 | -0.989467364595383 | 0.45187410618667 | -2.1896969776503 | 0.0285462204280037 | 0.0568980685720655 | 170.839238803006 | 145.093490215594 | 319.677988152986 | 140.270981380783 | 84.8492383399526 | 93.6493691420958 |
| YDL131W | 3587.3336699981 | -0.650020428837151 | 0.615858876331615 | -1.05546977370696 | 0.291210526451732 | 0.384775500687147 | 4029.84062857886 | 4295.92146314466 | 4822.23637964413 | 5902.70744630314 | 1081.04214773866 | 1392.25395457916 |
| YDL132W | 857.814453613738 | -1.79388850561101 | 0.475397462423404 | -3.77344989699022 | 0.00016100556536451 | 0.000813235953684272 | 1323.62613780559 | 1362.7246552635 | 1310.89921000034 | 667.376221973167 | 229.407199956168 | 252.853296683659 |
| YDL133C-A | 2367.04344331607 | -0.413462237782117 | 0.535952613938367 | -0.771452973694543 | 0.440438486190811 | 0.537091201872278 | 3836.32361471351 | 3343.74543360482 | 931.235878532611 | 1452.37096870662 | 2317.64123243389 | 2320.94353190494 |
| YDL133W | 668.49213795018 | -0.543455751818806 | 0.301998179783194 | -1.79953320317677 | 0.0719343765637521 | 0.122360640536335 | 836.809899800563 | 813.677697970404 | 729.333991278094 | 631.655040379303 | 551.520049209692 | 447.956149063025 |
| YDL134C | 3068.21236458779 | 0.498969490257478 | 0.258484310439847 | 1.9303666416287 | 0.0535614251486834 | 0.0958420611162882 | 2415.18304413984 | 2609.20963938838 | 2604.24173415247 | 3626.13555594298 | 3470.96236098065 | 3683.54185292243 |
| YDL135C | 1103.10124250612 | -1.22689022090299 | 0.43877921141717 | -2.79614482404574 | 0.00517162201300025 | 0.0137568136157462 | 1649.43017286796 | 1549.03788701762 | 1441.11129670797 | 1078.60543446838 | 443.10157799753 | 457.321085977234 |
| YDL136W | 2727.50180291929 | -1.83992917722399 | 0.537336990082347 | -3.42416251101942 | 0.000616697302897467 | 0.00240911228869947 | 5460.80823501289 | 4833.42689280697 | 2499.63314764017 | 2122.36093567446 | 754.21545191069 | 694.566154470544 |
| YDL137W | 9062.12703571444 | 1.57460533042407 | 0.381117976919703 | 4.13154305433308 | 3.60336181534098e-05 | 0.000251688178858958 | 4099.38580543672 | 4255.52611643691 | 5310.89746908622 | 8311.70908306007 | 15530.5531805943 | 16864.6905596724 |
| YDL138W | 648.506826088833 | -0.856622011058312 | 0.437111821360816 | -1.95973197062362 | 0.0500271263060663 | 0.0906512924623148 | 885.189153266901 | 930.741763939804 | 692.757562427638 | 737.07608849778 | 333.111824593888 | 312.164563806986 |
| YDL139C | 19.1464666461107 | -3.5541374942836 | 0.961904550654628 | -3.69489622630937 | 0.00021997661162334 | 0.00103631995527016 | 50.6470309725725 | 47.8149001846843 | 8.04681434710033 | 5.22748998934596 | 3.14256438296121 | 0 |
| YDL140C | 7007.19566046867 | -0.16835172809791 | 0.352183869807394 | -0.478022256357112 | 0.632634364236769 | 0.713764068970706 | 6973.41583167136 | 7492.92461687234 | 7782.00100222303 | 9182.95741461773 | 5369.07124828922 | 5242.80384913833 |
| YDL141W | 2020.37887329728 | 1.10322585224526 | 0.308423541660762 | 3.5769832818362 | 0.00034758239444779 | 0.00150533330211417 | 1155.81060234423 | 1217.63116504791 | 1476.22466840441 | 2221.68324547203 | 2990.15001038759 | 3060.7735481275 |
| YDL142C | 839.219244811429 | 1.33174794228872 | 0.380429197958683 | 3.50064597942178 | 0.000464131961628191 | 0.00190504132115076 | 418.782912817987 | 464.958684554517 | 547.183375602822 | 748.40231680803 | 1359.15909563072 | 1496.8290834545 |
| YDL143W | 10976.6033649451 | 1.07846682237173 | 0.264919271760575 | 4.0709262682347 | 4.68265702038151e-05 | 0.000309400791673689 | 6773.0954852873 | 6855.66741268681 | 7535.47587177096 | 14017.5144064312 | 14521.7900136637 | 16156.0769998306 |
| YDL144C | 2551.65548951512 | 1.60115823510235 | 0.388038193989413 | 4.12629029797525 | 3.68661855327382e-05 | 0.000255288424549284 | 1028.05913615968 | 1026.37156430917 | 1740.3064847047 | 2728.74977443859 | 4306.88448684833 | 4479.56149063025 |
| YDL145C | 6804.58782465349 | -0.282415213086288 | 0.592908564405635 | -0.47632169619509 | 0.633845210747537 | 0.714379271476048 | 6759.4888202499 | 7131.8396809949 | 8515.72416496318 | 12627.8733175967 | 3106.42489255715 | 2686.17607155911 |
| YDL146W | 889.160573722449 | 0.818691353863265 | 0.386009080946478 | 2.12091216055298 | 0.0339291951166732 | 0.0656168217871998 | 605.496594164635 | 660.340259447106 | 663.496419347273 | 666.50497364161 | 1389.01345726885 | 1350.11173846521 |
| YDL147W | 1325.57791799897 | -2.62539895701595 | 0.676626037384282 | -3.88013291236218 | 0.000104399399512863 | 0.000580532343390234 | 2349.41749645903 | 2380.85227126497 | 2116.31217328739 | 839.012143290026 | 146.129243807696 | 121.744179884725 |
| YDL148C | 190.713748677622 | -3.12479281419568 | 0.537181021836511 | -5.81702012389168 | 5.99059323545357e-09 | 3.53939637030468e-07 | 393.837360249407 | 378.397227323623 | 256.766530530201 | 71.4423631877281 | 20.4266684892478 | 23.412342285524 |
| YDL149W | 193.815443805105 | -0.889244977794211 | 0.391904708993756 | -2.26903366401851 | 0.0232662787786613 | 0.0481939000754516 | 230.557379800516 | 235.77692160034 | 290.416845072621 | 189.93213627957 | 122.560010935487 | 93.6493691420958 |
| YDL150W | 590.573449205265 | -2.68526431023894 | 0.600993505378997 | -4.46804214389233 | 7.89387563752981e-06 | 7.63918593190104e-05 | 1420.38464473826 | 1374.26618289429 | 272.860159224402 | 216.0695862263 | 114.703599978084 | 145.156522170248 |
| YDL153C | 505.461565032884 | -1.95986416396738 | 0.505322935896724 | -3.87843896396567 | 0.000105128895103257 | 0.000583143914267854 | 1078.70616713225 | 999.166534893748 | 335.771616847187 | 276.185721103778 | 168.127194488425 | 174.812155731912 |
| YDL154W | 164.487459133855 | -1.43263640651573 | 0.479107274066919 | -2.99022052901587 | 0.00278776100371841 | 0.00820154447873302 | 311.44144418955 | 281.943032123483 | 128.017500976596 | 124.588511412745 | 69.1364164251466 | 71.7978496756068 |
| YDL155W | 645.741343466279 | -1.21088811011706 | 0.544495845255951 | -2.2238702474357 | 0.0261571753685449 | 0.052972392884726 | 916.182112518774 | 967.839531324473 | 823.70117771227 | 750.144813471145 | 210.551813658401 | 206.028612112611 |
| YDL156W | 74.542364144357 | -2.8262064398414 | 0.949135603499331 | -2.97766349657686 | 0.00290454683414821 | 0.00847159493293228 | 154.964796259364 | 137.67393673866 | 100.950943627259 | 50.5324032303443 | 1.5712821914806 | 1.56082281903493 |
| YDL157C | 1994.62847658684 | 1.09921149182003 | 0.409937971350534 | 2.68140930736105 | 0.00733127836278915 | 0.0183852930904837 | 1483.12648907742 | 1362.7246552635 | 961.960078766994 | 1584.80071510338 | 3194.41669528007 | 3380.74222602966 |
| YDL158C | 499.50086151125 | 1.11300598246295 | 0.445249526600609 | 2.49973535280436 | 0.0124286113523845 | 0.0285348510193784 | 315.976999202019 | 366.031304862066 | 264.813344877302 | 337.173104312814 | 821.780586144356 | 891.229829668945 |
| YDL159C-B | 0.950522594641205 | -0.886325239991215 | 2.09668264598615 | -0.422727417374289 | 0.672494149399566 | 0.747024384185541 | 0.75592583541153 | 2.47318449231126 | 0.731528577009121 | 1.74249666311532 | 0 | 0 |
| YDL159W | 277.38656498924 | -0.786694541325727 | 0.55909290995984 | -1.40709089189172 | 0.15940043856766 | 0.2359859366381 | 309.929592518727 | 318.216404677382 | 427.212688973327 | 391.190500869389 | 122.560010935487 | 95.2101919611307 |
| YDL160C | 992.06528959244 | 0.597836621665516 | 0.335980837805454 | 1.7793771382036 | 0.0751779520096709 | 0.126972465497582 | 661.435105985088 | 760.092033970327 | 945.866450072793 | 925.265728114235 | 1420.43910109847 | 1239.29331831373 |
| YDL160C-A | 476.264724432858 | 0.770522642422438 | 0.475257086504952 | 1.6212754408122 | 0.10495857965533 | 0.16681472186089 | 445.240317057391 | 396.533913600572 | 213.606344486663 | 314.520647692315 | 736.931347804403 | 750.755775955801 |
| YDL161W | 141.696905015015 | -1.54657799546181 | 0.488933824584211 | -3.16316425188423 | 0.00156064252488986 | 0.00508930667161705 | 252.479229027451 | 282.767426954254 | 98.7563578962313 | 86.2535848242083 | 56.5661588933017 | 73.3586724946417 |
| YDL162C | 2.49411844268407 | 0.525487990751721 | 1.19400048724813 | 0.440107015335344 | 0.659859601174344 | 0.73724771539951 | 1.51185167082306 | 1.64878966154084 | 2.92611430803648 | 2.61374499467298 | 3.14256438296121 | 3.12164563806986 |
| YDL164C | 943.017231479977 | 0.118719022691041 | 0.342131060190923 | 0.346998669529714 | 0.728592318308312 | 0.791921439646503 | 898.795818304309 | 925.795394955181 | 889.538749643091 | 1329.52495395699 | 787.212377931782 | 827.236094088513 |
| YDL165W | 1127.62508101038 | 0.323120136249025 | 0.275121299718325 | 1.17446426932354 | 0.24020911558236 | 0.329488115942577 | 943.395442593589 | 943.93208123213 | 1118.50719424695 | 1315.58498065207 | 1184.74677237638 | 1259.58401496119 |
| YDL166C | 1202.83275613011 | 0.0269265907418404 | 0.329074921403349 | 0.0818251072643616 | 0.934785790307401 | 0.953483280607473 | 1388.63575965098 | 1323.97809821729 | 862.472192293754 | 1258.95383910082 | 1109.32522718531 | 1273.6314203325 |
| YDL167C | 893.482130540234 | -0.969763440889617 | 0.369426675118108 | -2.62504985753825 | 0.00866362801630803 | 0.0211821429100318 | 1286.58577187042 | 1182.18218732478 | 1081.93076539649 | 838.140894958468 | 417.961062933841 | 554.0921007574 |
| YDL168W | 3310.38114109041 | 1.3834655509733 | 0.363859702907978 | 3.80219502164322 | 0.000143419759615006 | 0.000744257253763607 | 1609.36610359115 | 1619.93584246387 | 2273.59081734435 | 3283.73496164082 | 5402.06817431032 | 5673.59094719197 |
| YDL169C | 34.0043240776347 | -0.858672599139157 | 0.459272951117076 | -1.86963459757565 | 0.0615345773986256 | 0.107485087380096 | 45.3555501246918 | 36.2733725538985 | 50.4754718136293 | 29.6224432729604 | 23.5692328722091 | 18.7298738284192 |
| YDL170W | 744.639320586271 | 0.0959965008422289 | 0.302499180633318 | 0.317344664012805 | 0.750982090412383 | 0.809426721716443 | 642.5369600998 | 705.681975139479 | 810.533663326106 | 642.981268689553 | 809.210328612511 | 856.891727650177 |
| YDL171C | 11305.7010369076 | 0.666064318231305 | 0.324455250631375 | 2.05286959275639 | 0.0400852267860563 | 0.0757256850989153 | 9838.37474788106 | 10005.6800610606 | 6379.66072009654 | 12832.6166755128 | 13513.0268467332 | 15264.8471701616 |
| YDL172C | 123.701918272352 | 0.432905762974714 | 0.433242980456794 | 0.999221643518093 | 0.317687333421878 | 0.413064087873389 | 102.049987780556 | 109.644512492466 | 103.145529358286 | 76.669853177074 | 161.842065722502 | 188.859561103227 |
| YDL173W | 168.694682622947 | -3.76909935722453 | 0.639778886786265 | -5.89125311114509 | 3.83277963395527e-09 | 2.65409686480343e-07 | 440.704762044922 | 388.289965292868 | 114.849986590432 | 32.2361882676334 | 28.2830794466509 | 7.80411409517465 |
| YDL174C | 2035.16970231467 | -0.210947101505312 | 0.53666563893391 | -0.393069885980328 | 0.694267865191413 | 0.764810990734297 | 1804.39496912732 | 1920.83995569508 | 2827.35795014025 | 3505.90328618802 | 960.053418994649 | 1192.46863374269 |
| YDL175C | 299.40823358037 | -1.22589767890331 | 0.647369630296673 | -1.89365954399423 | 0.0582702144750575 | 0.102792624927619 | 397.616989426464 | 404.777861908276 | 457.93688920771 | 392.061749200947 | 69.1364164251466 | 74.9194953136766 |
| YDL176W | 421.15752403634 | -0.464888312891941 | 0.280031472558436 | -1.66012880139724 | 0.0968885439737809 | 0.155912599498038 | 498.155125536198 | 509.476005416119 | 457.93688920771 | 365.924299254217 | 367.680032806461 | 327.772791997335 |
| YDL177C | 121.784405090349 | -1.60295130207625 | 0.468855752160015 | -3.41885813428003 | 0.000628845032811644 | 0.00244255851104161 | 196.540717206998 | 185.488836923344 | 169.714629866116 | 97.5798131344579 | 32.9969260210927 | 48.3855073900828 |
| YDL178W | 2925.88344588107 | 1.32236051743176 | 0.337997496795958 | 3.91233819767027 | 9.14067798726873e-05 | 0.000523718560836393 | 1727.29053391534 | 1733.70232911019 | 1553.03516899036 | 2902.12819241856 | 4614.85579637853 | 5024.28865447344 |
| YDL179W | 1015.69718767829 | -1.69988890360421 | 0.405419137845297 | -4.19291726739566 | 2.75389898508137e-05 | 0.000202455587487717 | 1702.34498134676 | 1688.36061341782 | 1271.39666684185 | 717.908625203512 | 366.108750614981 | 348.063488644789 |
| YDL180W | 1230.38200074158 | 0.103113021619126 | 0.278657684772091 | 0.370034731694052 | 0.711356612096695 | 0.778310666310349 | 1099.87209052378 | 1260.49969624797 | 1198.24380914094 | 1122.16785104627 | 1365.44422439664 | 1336.0643330939 |
| YDL181W | 1711.51024770223 | 1.07760235532628 | 0.37369989497449 | 2.883603580889 | 0.0039315332619528 | 0.0108994717177057 | 1053.76061456367 | 969.488320986013 | 1277.24889545793 | 1472.40968033244 | 2653.89562141074 | 2842.25835346261 |
| YDL182W | 3730.67518715806 | -1.03446729164334 | 0.606714282801408 | -1.70503204056257 | 0.0881884488995241 | 0.144512369189042 | 5363.2938022448 | 5715.52936173132 | 3964.15335881243 | 5069.79404133402 | 980.480087483897 | 1290.80047134189 |
| YDL183C | 98.0597778047093 | -1.79821524802208 | 0.481920898336914 | -3.73134938581753 | 0.000190456864520382 | 0.000930608655167876 | 145.137760399014 | 142.620305723283 | 171.177687020134 | 71.4423631877281 | 34.5682082125733 | 23.412342285524 |
| YDL184C | 2194.70536837554 | 0.0864869384303944 | 0.525878990940072 | 0.164461672590853 | 0.869367716714516 | 0.903895398069339 | 2943.5752030925 | 2558.92155471138 | 883.686521027018 | 1304.25875234182 | 2708.89049811256 | 2768.89968096797 |
| YDL185W | 40379.7177948246 | 1.81941708128871 | 0.368194048920574 | 4.9414624886596 | 7.75387358632929e-07 | 1.27999741889194e-05 | 16876.8002013978 | 18377.4095675342 | 18235.5443676834 | 38767.0657609896 | 74780.4620569449 | 75241.0248143978 |
| YDL186W | 7.73673660550947 | -0.900461881881116 | 0.797075041157975 | -1.12970778833188 | 0.258599374362204 | 0.34935598298565 | 10.5829616957614 | 7.41955347693378 | 11.7044572321459 | 2.61374499467298 | 7.85641095740302 | 6.24329127613972 |
| YDL187C | 145.414821709639 | 1.72723195180165 | 0.480294213879338 | 3.59619562736512 | 0.000322905003292312 | 0.00141655873378916 | 71.8129543640953 | 70.0735606154857 | 59.9853433147479 | 101.936054792246 | 315.827720487601 | 252.853296683659 |
| YDL188C | 2496.96939183869 | 1.40794173061824 | 0.354024784598247 | 3.97695808844568 | 6.98024618886443e-05 | 0.000424483337641991 | 1164.12578653376 | 1293.47548947879 | 1642.28165538548 | 2540.56013482214 | 4036.62394991367 | 4304.74933489834 |
| YDL189W | 320.166185686505 | -1.50591144769161 | 0.31096889196864 | -4.8426433851894 | 1.2812311631492e-06 | 1.87525652060928e-05 | 514.785493915252 | 503.705241600726 | 403.072245932026 | 184.704646290224 | 150.843090382138 | 163.886395998668 |
| YDL190C | 1605.42199193563 | -0.429545227540075 | 0.365984668695348 | -1.17367000391384 | 0.24052722948759 | 0.329713784142205 | 1778.69349072333 | 1854.88836923344 | 1896.12207160764 | 1931.55755106333 | 1197.31702990822 | 973.953439077796 |
| YDL191W | 6656.73836875046 | -1.42367081063768 | 0.546181849322961 | -2.60658755394826 | 0.00914494408397558 | 0.0221154487047701 | 12077.42707237 | 10927.3534818619 | 6092.16998933196 | 6665.92098474765 | 1995.52838318037 | 2182.03030101083 |
| YDL192W | 14548.5299955663 | 0.55990135350387 | 0.363038080571183 | 1.54226617941279 | 0.123008923946449 | 0.190945526879734 | 13463.0391286793 | 13333.7619928808 | 8483.53690757478 | 12689.7319491373 | 19356.6253168496 | 19964.4846782758 |
| YDL193W | 660.584113936661 | 0.164741058084949 | 0.379361239610594 | 0.434259067304959 | 0.664100324390859 | 0.740515485789048 | 589.622151620993 | 638.905993847075 | 641.550562036999 | 996.708091301963 | 622.227747826319 | 474.490136986619 |
| YDL194W | 299.946003372513 | -0.674020352969458 | 0.462551231313894 | -1.45717989130605 | 0.145066729845927 | 0.218496828123119 | 405.17624778058 | 389.114360123638 | 313.825759536913 | 381.606769222255 | 136.701550658813 | 173.251332912877 |
| YDL195W | 5411.46811365354 | -0.768346758538707 | 0.613541978409368 | -1.25231326555793 | 0.210455736971333 | 0.296183336122243 | 5788.12412174608 | 6079.91187693184 | 8591.80313697213 | 8384.89394291092 | 1852.54170375563 | 1771.53389960465 |
| YDL196W | 3.82306418761186 | 1.39255221597287 | 1.10027464776701 | 1.26564055510961 | 0.205641799619477 | 0.290814365428898 | 2.26777750623459 | 2.47318449231126 | 1.46305715401824 | 2.61374499467298 | 10.9989753403642 | 3.12164563806986 |
| YDL197C | 125.731643599159 | -3.71563696764408 | 0.806191126064995 | -4.60887852460004 | 4.0484677088304e-06 | 4.59825961990613e-05 | 323.536257556135 | 319.865194338923 | 59.2538147377388 | 39.2061749200947 | 6.28512876592241 | 6.24329127613972 |
| YDL198C | 8212.65834005167 | 1.00462033955067 | 0.409125748399587 | 2.45552948813545 | 0.0140677210609688 | 0.0317213318041453 | 4749.48202389064 | 5180.49711656132 | 6459.39733499054 | 6081.31335427246 | 13084.066808459 | 13721.1934021361 |
| YDL199C | 265.873670471603 | 1.05746263992976 | 0.423744188976467 | 2.49552127778792 | 0.012577221187978 | 0.0288041623223963 | 128.50739201996 | 150.039859200216 | 238.478316104973 | 236.979546183683 | 461.956964295297 | 379.279945025488 |
| YDL200C | 313.768469038944 | -0.177326859384121 | 0.438752876971473 | -0.404161131906721 | 0.686094233577909 | 0.758401452839295 | 377.962917705765 | 313.270035692759 | 306.510473766822 | 147.240968033244 | 372.393879380903 | 365.232539654174 |
| YDL201W | 1279.20912622501 | -0.942165737671525 | 0.538474578768769 | -1.74969399637361 | 0.080171130212201 | 0.133791676228706 | 1752.23608648393 | 1714.74124800247 | 1583.02784064774 | 1672.79679659071 | 521.66568757156 | 430.787098053641 |
| YDL202W | 250.457083970737 | -1.82200963028459 | 0.754301388520196 | -2.41549287594319 | 0.0157139298838443 | 0.0347218103072985 | 421.050690324222 | 352.84098756974 | 399.41460304698 | 268.344486119759 | 32.9969260210927 | 28.0948107426287 |
| YDL203C | 559.379692781129 | -0.750327121082936 | 0.601241740168357 | -1.24796245994637 | 0.212044805127869 | 0.298028927329437 | 634.977701745685 | 622.418097231667 | 849.30467790759 | 858.179606584295 | 179.126169828789 | 212.27190338875 |
| YDL204W | 269.124561730699 | -0.506603136366153 | 0.752229072964139 | -0.673469232410675 | 0.500648816449722 | 0.594209063386696 | 209.391456408994 | 201.152338707982 | 538.405032678713 | 512.294018955904 | 81.7066739569914 | 71.7978496756068 |
| YDL205C | 1424.8221088747 | 0.149746943085056 | 0.287220761369508 | 0.521365316250266 | 0.602112308130814 | 0.686566412940786 | 1394.68316633427 | 1419.60789858666 | 1237.74635229943 | 1271.15131574263 | 1569.71090928912 | 1656.03301099606 |
| YDL206W | 763.722828416059 | 0.492175203430038 | 0.341614792828296 | 1.44073153084275 | 0.149660543402421 | 0.223830445776032 | 618.347333366631 | 663.637838770188 | 621.067761880744 | 615.101322079708 | 1054.33035048349 | 1009.8523639156 |
| YDL207W | 412.72461341196 | -0.184456935726627 | 0.438906443406288 | -0.420264816107697 | 0.674292009578153 | 0.748309588434138 | 555.605489027474 | 499.583267446874 | 261.155701992256 | 250.048271157048 | 468.24209306122 | 441.712857786885 |
| YDL208W | 3825.91061034411 | -1.41409201187905 | 0.586143932627241 | -2.41253373645061 | 0.0158420712503753 | 0.0349633100933575 | 6237.14406798053 | 5945.53551951627 | 4511.33673441525 | 4237.75188469646 | 1068.47189020681 | 955.223565249377 |
| YDL209C | 122.193917671742 | -0.938556303369903 | 0.360060655332724 | -2.60666165399995 | 0.00914296550486635 | 0.0221154487047701 | 150.429241246894 | 150.864254030987 | 181.419087098262 | 100.193558129131 | 62.8512876592241 | 87.4060778659561 |
| YDL210W | 514.070657449969 | 1.6557248212112 | 0.458387572006668 | 3.61206307135028 | 0.000303770656520037 | 0.0013519578631576 | 217.70664059852 | 183.840047261804 | 340.89231688625 | 419.941695810792 | 1038.61752856868 | 883.42571557377 |
| YDL211C | 140.625451982997 | -1.36217850542455 | 0.426116413236563 | -3.19672855377275 | 0.00138995661534591 | 0.00462839741614669 | 178.398497157121 | 201.976733538753 | 228.968444603855 | 118.489773091842 | 62.8512876592241 | 53.0679758471876 |
| YDL212W | 10068.0997628605 | 1.37359081250735 | 0.388056329368655 | 3.53966862167177 | 0.000400629740883632 | 0.0016947589325668 | 6000.53928149672 | 6282.71300530137 | 4537.67176318758 | 8811.80562537417 | 17227.5379473933 | 17548.3309544097 |
| YDL213C | 301.46021285531 | -2.66295072943886 | 0.684249674656353 | -3.89178223690973 | 9.95105416044767e-05 | 0.000556774880914709 | 780.115462144698 | 630.662045539371 | 152.889472594906 | 154.210954685706 | 53.4235945103405 | 37.4597476568383 |
| YDL214C | 34.8073753839721 | -0.964137763122372 | 0.587761541737385 | -1.64035530509949 | 0.100931312654676 | 0.161490100247482 | 39.3081434413995 | 45.3417156923731 | 54.8646432756841 | 42.6911682463253 | 15.712821914806 | 10.9257597332445 |
| YDL215C | 1817.7471871236 | 0.454224677210636 | 0.353445494583371 | 1.28513358968138 | 0.198745605545899 | 0.282594548380676 | 1383.3442788031 | 1481.43751089444 | 1735.91731324264 | 1412.29354545497 | 2391.49149543348 | 2501.99897891299 |
| YDL216C | 168.850229174012 | -2.08213553960769 | 0.507854126469911 | -4.09986929530452 | 4.13383539137586e-05 | 0.000281415432563008 | 305.394037506258 | 288.538190769647 | 227.505387449837 | 105.421048118477 | 59.708723276263 | 26.5339879235938 |
| YDL217C | 992.004543201765 | 1.6150329044056 | 0.320330759446068 | 5.04176653905606 | 4.61253816163703e-07 | 8.58518663611054e-06 | 439.94883620951 | 443.524418954486 | 580.833690145242 | 1205.8076908758 | 1566.56834490616 | 1715.34427811939 |
| YDL218W | 72.6209495627307 | 2.42012337222299 | 0.459299821038554 | 5.2691580997151 | 1.3705088634005e-07 | 3.64713928937985e-06 | 17.3862942144652 | 16.4878966154084 | 34.3818431194287 | 94.9660681397849 | 138.272832850293 | 134.230762437004 |
| YDL219W | 669.116506741483 | -0.058770227603573 | 0.282143157711715 | -0.208299318970629 | 0.834995263367 | 0.87707869777907 | 687.892510224492 | 658.691469785565 | 700.804376774738 | 568.925160507152 | 686.650317677024 | 711.735205479928 |
| YDL220C | 367.348881095859 | -0.302055891724882 | 0.452364009442219 | -0.667727505769805 | 0.504307548959904 | 0.598000481550687 | 328.827738404015 | 323.987168492775 | 565.47159002805 | 501.839038977212 | 251.405150636897 | 232.562600036205 |
| YDL221W | 7.15480742548795 | 0.280082600998567 | 0.928851626164382 | 0.301536427464897 | 0.763005476704921 | 0.818549936694934 | 1.51185167082306 | 6.59515864616336 | 10.9729286551368 | 3.48499332623064 | 10.9989753403642 | 9.36493691420958 |
| YDL222C | 395.516985249814 | 2.50213964296691 | 0.524438813636776 | 4.77108020593587 | 1.83240550700343e-06 | 2.48435609791623e-05 | 78.6162868827991 | 75.8443244308786 | 201.170358677508 | 378.993024227582 | 879.918027229138 | 758.559890050976 |
| YDL223C | 125.101960848808 | -0.360399515163928 | 0.324281909416233 | -1.11137718355215 | 0.266406029165077 | 0.357279222786983 | 150.429241246894 | 147.566674707905 | 123.628329514541 | 101.936054792246 | 109.989753403642 | 117.06171142762 |
| YDL224C | 1869.58126233667 | -0.0823709184203209 | 0.268123280066288 | -0.307212855220763 | 0.758681361309228 | 0.815813652835436 | 1734.84979226946 | 2050.26994412603 | 1983.90550084874 | 1860.98643620716 | 1747.26579692643 | 1840.21010364218 |
| YDL225W | 204.976290320749 | -4.57308731132583 | 0.67446222374103 | -6.78034610442725 | 1.19888323969423e-11 | 4.54165180213577e-09 | 554.093637356651 | 511.94918990843 | 115.581515167441 | 27.8799466098451 | 9.42769314888362 | 10.9257597332445 |
| YDL226C | 1499.55250891428 | -0.325126042208564 | 0.657149811607399 | -0.494751784852986 | 0.620775342461303 | 0.703217802190113 | 1427.18797725697 | 1472.36916775597 | 2105.33924463225 | 2918.68191071816 | 452.529271146414 | 621.207481975902 |
| YDL227C | 530.73743114596 | -0.579337096577421 | 0.51118493650927 | -1.13332192559026 | 0.257079087510561 | 0.347520848775821 | 802.037311371633 | 749.374901170311 | 357.71747415746 | 696.12741691457 | 249.833868445416 | 329.33361481637 |
| YDL228C | 10.3365663599193 | 1.11595394588904 | 0.756991911071992 | 1.47419533758124 | 0.140429004850581 | 0.212941556684187 | 8.31518418952682 | 5.77076381539294 | 5.12070003906385 | 5.22748998934596 | 18.8553862977672 | 18.7298738284192 |
| YDL229W | 199166.801541205 | 1.29633967359041 | 0.384548545303239 | 3.37106898315834 | 0.00074877105729211 | 0.00281663879028107 | 123120.664516817 | 131471.190031943 | 91178.4533669939 | 176670.865424931 | 324680.324354403 | 347879.311552143 |
| YDL230W | 528.011406805071 | 0.297553586041611 | 0.387491105844708 | 0.767897847340164 | 0.4425478764294 | 0.538957701249119 | 418.782912817987 | 378.397227323623 | 624.725404765789 | 733.591095171549 | 408.533369784957 | 604.038430966518 |
| YDL231C | 1410.2262407956 | 0.122875034306937 | 0.273700200978162 | 0.44894024143132 | 0.653474765743755 | 0.732273793525279 | 1220.82022418962 | 1314.08536024805 | 1515.7272115629 | 1483.73590864269 | 1473.86269560881 | 1453.12604452152 |
| YDL232W | 396.695587808432 | -0.0204019864884394 | 0.41471688739135 | -0.0491949739900196 | 0.96076391659378 | 0.972871094725105 | 455.823278753152 | 446.821998277567 | 294.806016534676 | 238.722042846799 | 424.246191699763 | 519.753998738632 |
| YDL233W | 407.062933951283 | -0.73478785220021 | 0.481314586330834 | -1.52662701914284 | 0.126853784404501 | 0.195580170353121 | 479.25697965091 | 486.392950154548 | 561.813947143005 | 523.620247266153 | 163.413347913983 | 227.8801315791 |
| YDL234C | 2261.95440741209 | -1.86475921621126 | 0.363263401787912 | -5.13335284268461 | 2.84625588747809e-07 | 6.27735887512292e-06 | 3498.42476628456 | 3703.18157982072 | 3448.425712021 | 1306.00124900493 | 974.194958717974 | 641.498178623356 |
| YDL235C | 1230.71574195144 | -1.64217904366664 | 0.470220574513198 | -3.49235897507616 | 0.000478774337866068 | 0.00195022563937854 | 2067.45715985053 | 1952.99135409512 | 1574.24949772363 | 979.283124670809 | 505.952865656754 | 304.360449711811 |
| YDL236W | 8989.13039738872 | 1.67532716930076 | 0.36488687899182 | 4.59136040717518 | 4.40366133320265e-06 | 4.91500502353988e-05 | 4416.11873047416 | 4339.61438917549 | 4103.87531702117 | 8543.46113925441 | 15866.8075695711 | 16664.905238836 |
| YDL237W | 3559.5838845356 | 1.74327579314392 | 0.370358355226421 | 4.70699734066525 | 2.51392186220221e-06 | 3.19953691553008e-05 | 1461.9605656859 | 1567.99896812534 | 1881.49150006746 | 3478.89458790973 | 6307.12671660314 | 6660.03096882205 |
| YDL238C | 462.273089823636 | 1.11925284285787 | 0.367532156136448 | 3.04531950244468 | 0.00232433246550681 | 0.00706376397680976 | 235.848860648397 | 246.494054400355 | 391.36778869988 | 500.096542314097 | 669.366213570737 | 730.465079308347 |
| YDL239C | 96.0620647196973 | -0.538780660780923 | 0.374341475301872 | -1.43927589200861 | 0.15007237047817 | 0.224342169424191 | 117.924430324199 | 123.659224615563 | 98.7563578962313 | 57.5023898828055 | 89.5630849143944 | 88.966900684991 |
| YDL240W | 383.566481691399 | -0.590541401461146 | 0.329203240940105 | -1.793850509414 | 0.0728370325249013 | 0.123634815355921 | 447.508094563625 | 486.392950154548 | 450.621603437619 | 393.804245864062 | 263.975408168741 | 259.096587959798 |
| YDL241W | 1069.8044478019 | 0.262217450904655 | 0.302725013754839 | 0.866190235330242 | 0.386385847311112 | 0.484419405474682 | 930.544703391593 | 988.449402093733 | 998.53650761745 | 917.424493130216 | 1279.02370386521 | 1304.8478767132 |
| YDL242W | 11.9025444071259 | 0.578863444556335 | 0.710044544805178 | 0.815249478066426 | 0.414929521445248 | 0.512494460703375 | 9.82703586034988 | 8.2439483077042 | 10.2414000781277 | 8.7124833155766 | 7.85641095740302 | 26.5339879235938 |
| YDL243C | 529.479087740027 | -0.479305491420875 | 0.612945359984477 | -0.781970992378528 | 0.434231621312714 | 0.531543744773594 | 802.037311371633 | 873.858520616645 | 173.372272751162 | 234.36580118901 | 568.804153315979 | 524.436467195736 |
| YDL244W | 77.391937833319 | 1.64789534058355 | 0.502131908662975 | 3.28179769529365 | 0.00103147579859844 | 0.00365787673071254 | 33.2607367581073 | 34.6245828923576 | 43.8917146205473 | 54.8886448881326 | 169.698476679905 | 127.987471160864 |
| YDL248W | 473.648261095567 | 0.610253513003843 | 0.400595344604827 | 1.52336646249805 | 0.127667047700571 | 0.196410842616264 | 398.372915261876 | 434.456075816011 | 291.14837364963 | 349.370580954622 | 658.367238230373 | 710.174382660893 |
| YDR001C | 2655.61831918774 | 0.439694122131558 | 0.313228701410628 | 1.40374786905348 | 0.160393942482084 | 0.237020878748192 | 1914.00421526199 | 1976.89880418747 | 2871.2496647608 | 3166.98768521209 | 3060.85770900422 | 2943.71183669988 |
| YDR002W | 5990.4027630619 | -1.08083902869161 | 0.346590380425017 | -3.11849113459582 | 0.00181779610612566 | 0.00577695894068696 | 9536.00441371644 | 9028.77218659763 | 5841.25568741783 | 4603.67618395067 | 3681.51417463905 | 3251.19393204976 |
| YDR003W | 1034.60849160351 | 1.68127379147599 | 0.402832875359624 | 4.17362607253704 | 2.99789643605153e-05 | 0.0002164667663362 | 398.372915261876 | 413.02181021598 | 663.496419347273 | 1024.58803791181 | 1824.25862430898 | 1883.91314257516 |
| YDR003W-A | 256.635065881923 | 1.73558498345885 | 0.405539488665499 | 4.2796941653453 | 1.87150306230696e-05 | 0.000148979971832594 | 110.365171970083 | 106.346933169384 | 138.258901054724 | 234.36580118901 | 458.814399912336 | 491.659187996003 |
| YDR004W | 486.626024111335 | -0.552621403490261 | 0.375145442698732 | -1.47308574379792 | 0.140727917010958 | 0.213173587368018 | 600.205113316754 | 545.749377970018 | 591.806618800379 | 555.856435533787 | 271.831819126144 | 354.306779920929 |
| YDR005C | 1333.26767963768 | 0.230905761333497 | 0.284963036469215 | 0.810300747053003 | 0.417767346099965 | 0.515110417170931 | 1165.63763820458 | 1212.68479606329 | 1301.38933849923 | 1266.79507408484 | 1660.845276395 | 1392.25395457916 |
| YDR006C | 1640.18128619181 | -0.420248257104726 | 0.264728441251171 | -1.58746923873586 | 0.112406408579447 | 0.17703528277125 | 1917.78384443905 | 1879.62021415656 | 1835.40519971588 | 1513.35835191565 | 1313.59191207778 | 1381.32819484591 |
| YDR007W | 5429.19083468732 | 1.82422367297531 | 0.416539044314959 | 4.37947822148445 | 1.18963809433434e-05 | 0.00010494889489744 | 2412.9152666336 | 2452.574621542 | 2307.24113188677 | 4378.8941144088 | 10202.3352692836 | 10821.1846043692 |
| YDR008C | 25.0971495887015 | 0.827193385958547 | 0.596647359576426 | 1.38640249165904 | 0.165624036046446 | 0.243210063343724 | 13.6066650374075 | 18.1366862769492 | 21.9458573102736 | 13.9399733049226 | 34.5682082125733 | 48.3855073900828 |
| YDR009W | 129.573160607642 | -0.398819774680192 | 0.49537532149973 | -0.805086077911148 | 0.420770009131452 | 0.518316537644711 | 117.924430324199 | 137.67393673866 | 188.002844291344 | 188.189639616454 | 73.8502629995884 | 71.7978496756068 |
| YDR010C | 33.1693420186344 | 1.77386958966026 | 0.547632536771515 | 3.23916033206836 | 0.00119882162632066 | 0.00411349904341634 | 15.8744425436421 | 14.8391069538676 | 13.8990429631733 | 24.3949532836145 | 69.1364164251466 | 60.8720899423623 |
| YDR011W | 4511.02417012129 | -0.213055003705492 | 0.349497435732217 | -0.60960391099617 | 0.542124220244662 | 0.632822182051046 | 4942.24311192058 | 5686.67554265435 | 3900.51037261263 | 2998.83675722146 | 4643.13887582518 | 4894.74036049354 |
| YDR012W | 82135.6783604882 | 1.17885693057747 | 0.422867718023986 | 2.78776761698939 | 0.00530725988095723 | 0.0140470990255596 | 55633.1177829469 | 56189.1028756503 | 39163.1138987603 | 61323.6850650174 | 138609.08723927 | 141895.963301285 |
| YDR013W | 461.31920849229 | 0.413569409492894 | 0.372861065102251 | 1.10917831922054 | 0.267353263625462 | 0.35840029511823 | 407.444025286814 | 456.714736246812 | 321.872573884013 | 351.113077617737 | 595.515950571149 | 635.254887347216 |
| YDR014W | 141.305699406399 | -1.64523965523234 | 0.463347723441824 | -3.55076667477984 | 0.000384110779346384 | 0.00163711013831285 | 263.062190723212 | 238.250106092651 | 139.721958208742 | 39.2061749200947 | 87.9918027229138 | 79.6019637707814 |
| YDR014W-A | 9.72648487350365 | -2.51900437568846 | 0.891059627712447 | -2.82697621724297 | 0.00469898176190516 | 0.0127042160145547 | 17.3862942144652 | 16.4878966154084 | 16.8251572712098 | 6.09873832090362 | 0 | 1.56082281903493 |
| YDR016C | 385.518633267322 | 1.16403325640846 | 0.397294351689272 | 2.92990134760048 | 0.00339069631325904 | 0.0096449135412492 | 264.574042394035 | 213.518261169539 | 234.820673219928 | 312.7781510292 | 664.652366996295 | 622.768304794937 |
| YDR017C | 752.440775987055 | -1.39701214034237 | 0.351940682816746 | -3.96945340095789 | 7.20376927215776e-05 | 0.000435199569537486 | 1244.25392508738 | 1256.37772209412 | 772.494177321632 | 481.800327351386 | 408.533369784957 | 351.185134282859 |
| YDR018C | 87.6790767883979 | 0.262382532298914 | 0.455202208058656 | 0.576408742431022 | 0.564338936605372 | 0.653776353973484 | 67.2773993516261 | 63.4784019693223 | 107.534700820341 | 59.2448865459209 | 98.990778063278 | 129.548293979899 |
| YDR019C | 257.262004620961 | 0.0154279597784446 | 0.458689928983606 | 0.0336348343479676 | 0.97316834418197 | 0.98083546550264 | 227.53367645887 | 215.16705083108 | 326.261745346068 | 412.971709158331 | 163.413347913983 | 198.224498017436 |
| YDR020C | 370.320550140297 | -0.702512245743664 | 0.485310763561301 | -1.44755133924601 | 0.14774256850799 | 0.221682698320469 | 452.799575411506 | 421.265758523684 | 504.023189559284 | 491.38405899852 | 190.125145169153 | 162.325573179633 |
| YDR021W | 287.487173018855 | -1.85576561294865 | 0.405842535013276 | -4.57262473212166 | 4.81652322523598e-06 | 5.25735755432537e-05 | 517.809197256898 | 507.827215754578 | 327.724802500086 | 174.249666311532 | 97.4194958717974 | 99.8926604182355 |
| YDR022C | 120.293773002438 | -0.281341162584815 | 0.354364201285304 | -0.79393223571786 | 0.427234882088292 | 0.524674416599657 | 139.090353715721 | 117.88846080017 | 138.258901054724 | 91.4810748135543 | 138.272832850293 | 96.7710147801657 |
| YDR023W | 11389.1605647513 | -0.651524865520352 | 0.364960699512111 | -1.78519184775601 | 0.0742302038407432 | 0.125668378741952 | 15337.7352004999 | 15729.4533710996 | 10688.3640386803 | 11754.8824893759 | 8181.6663710395 | 6642.86191781266 |
| YDR024W | 255.460999739857 | -0.00400574346460523 | 0.31763346772513 | -0.0126112134634114 | 0.989937974200562 | 0.992867240905096 | 211.659233915228 | 266.279530338846 | 288.953787918603 | 225.653317873434 | 260.83284378578 | 279.387284607252 |
| YDR025W | 36371.6301165198 | -0.249962156744793 | 0.363696766129364 | -0.687281768834543 | 0.491905185156284 | 0.586099795079828 | 39410.1934291801 | 40218.9262139657 | 38916.5887683082 | 47422.0466866834 | 24769.6924665002 | 27492.3331344813 |
| YDR026C | 180.180236897222 | -3.21210425011679 | 0.632188728384512 | -5.08092616318068 | 3.75599100740183e-07 | 7.5281334365445e-06 | 449.77587206986 | 432.80728615447 | 94.3671864341766 | 54.0173965565749 | 25.1405150636897 | 24.9731651045589 |
| YDR027C | 545.779015712367 | -0.564103131954504 | 0.284243952212895 | -1.98457391111702 | 0.0471918973279721 | 0.0865117616829321 | 675.041771022496 | 645.501152493238 | 632.040690535881 | 388.576755874716 | 490.240043741948 | 443.27368060592 |
| YDR028C | 630.140065697915 | -3.19190894486737 | 0.66599267108596 | -4.79270881414158 | 1.64544363362622e-06 | 2.26909143480789e-05 | 1588.20018019962 | 1441.86655901746 | 379.663331467734 | 239.593291178356 | 61.2800054677435 | 70.2370268565718 |
| YDR030C | 143.779982581362 | -0.522297781424093 | 0.400543135453975 | -1.30397386746404 | 0.192242492591722 | 0.275303903111116 | 153.45294458854 | 126.956803938645 | 228.968444603855 | 148.112216364802 | 108.418471212162 | 96.7710147801657 |
| YDR031W | 241.432873987966 | 0.699490000352979 | 0.383168067279751 | 1.82554356713207 | 0.067919042942 | 0.116444370343256 | 203.344049725701 | 171.474124800247 | 176.298387059198 | 188.189639616454 | 336.254388976849 | 373.036653749348 |
| YDR032C | 7324.25008669932 | 1.84034998931331 | 0.426037084007678 | 4.31969436087902 | 1.56245446258422e-05 | 0.000130183949226617 | 2893.68409795533 | 2943.0895458504 | 3755.66771436483 | 5900.96494964003 | 14281.3838383672 | 14170.7103740181 |
| YDR033W | 57803.2998451371 | 1.0602201963092 | 0.369100180646451 | 2.87244561748062 | 0.00407308175420388 | 0.0112144704989624 | 40203.1596305268 | 40397.8198922429 | 31810.5201712416 | 49649.8286704763 | 94138.6586559859 | 90619.812050349 |
| YDR034C | 1017.10346610164 | -1.30291483491074 | 0.325405403035886 | -4.00397418959591 | 6.22871633831192e-05 | 0.000389445953579891 | 1570.81388598516 | 1578.71610092535 | 1194.58616625589 | 724.878611855973 | 523.236969763041 | 510.389061824422 |
| YDR034C-A | 2.23898020940127 | 0.526244300387692 | 1.40359265576016 | 0.374926655698899 | 0.707715014308584 | 0.775644093285787 | 1.51185167082306 | 2.47318449231126 | 1.46305715401824 | 1.74249666311532 | 0 | 6.24329127613972 |
| YDR034C-D | 6.59845736090071 | -2.94915288062382 | 1.25950978838568 | -2.3415085041965 | 0.019205988403568 | 0.0410373474847305 | 16.6303683790536 | 17.3122914461788 | 1.46305715401824 | 2.61374499467298 | 1.5712821914806 | 0 |
| YDR034W-B | 250.822115313061 | 0.256085818919349 | 0.421480579562518 | 0.607586283536852 | 0.54346190033858 | 0.634038883728343 | 184.445903840413 | 188.786416246426 | 311.631173805886 | 173.378417979974 | 311.11387391316 | 335.57690609251 |
| YDR035W | 7363.57815996346 | 0.394597881646868 | 0.299293761965852 | 1.31843002358295 | 0.187359735725393 | 0.269510095615709 | 5853.13374359147 | 6288.48376911676 | 6945.8638387016 | 6813.1619527809 | 8511.63563125043 | 9769.19002433963 |
| YDR036C | 585.045548100414 | -0.694715419410223 | 0.810587334725214 | -0.857051905018636 | NA | NA | 591.889929127228 | 602.632621293177 | 976.590650307177 | 1130.88033436184 | 103.70462463772 | 104.57512887534 |
| YDR037W | 20494.455323525 | -0.102812944994752 | 0.471145016086349 | -0.218219319921468 | 0.827258232678228 | 0.870798139661293 | 19663.1428307247 | 20664.2808280913 | 23347.4660638231 | 34166.8745703652 | 12956.7929509491 | 12168.1746971963 |
| YDR038C | 714.813085597825 | 1.29413324272241 | 0.418220419332137 | 3.09438081667325 | 0.00197224157252551 | 0.00615668236891143 | 396.105137755641 | 440.226839631404 | 405.266831663053 | 539.302717234191 | 1367.01550658813 | 1140.96148071453 |
| YDR040C | 281.57982904831 | 0.728023335763961 | 0.427783688687226 | 1.70184921729509 | 0.0887836375195648 | 0.145265910982215 | 245.675896508747 | 258.859976861912 | 130.943615284633 | 237.850794515241 | 444.672860189011 | 371.475830930313 |
| YDR041W | 704.257455613004 | 0.356579489556039 | 0.369197338193025 | 0.965823565525303 | 0.334132474440611 | 0.430104564340903 | 523.85660394019 | 458.363525908353 | 870.519006640854 | 764.956035107625 | 733.788783421442 | 874.060778659561 |
| YDR042C | 109.040404703956 | -0.945861580356915 | 0.35058012466599 | -2.69798974273875 | 0.00697595900697299 | 0.0176177160803553 | 128.50739201996 | 154.161833354068 | 147.037243978833 | 60.1161348774785 | 80.1353917655108 | 84.2844322278862 |
| YDR043C | 1051.45638219978 | -1.39533727330629 | 0.374545697008822 | -3.72541263843013 | 0.0001949959783049 | 0.000947039291314901 | 1442.3064939652 | 1464.94961427904 | 1665.69056984977 | 805.033458359277 | 545.23492044377 | 385.523236301628 |
| YDR044W | 8279.88569088763 | 1.98468513975872 | 0.318545742154007 | 6.23045571520834 | 4.65080287815535e-10 | 6.1863391586301e-08 | 3496.15698877832 | 3397.3310976049 | 3126.55313813698 | 9758.85256177734 | 14498.2207807915 | 15402.1995782367 |
| YDR045C | 943.2690617798 | 0.233026361562142 | 0.487648068407477 | 0.47785765321114 | 0.63275152332588 | 0.713771205153033 | 1068.8791312719 | 1060.99614720153 | 471.104403593874 | 543.65895889198 | 1246.02677784412 | 1268.9489518754 |
| YDR046C | 12708.3606433692 | 0.909730462329026 | 0.386709929881602 | 2.35248798138583 | 0.018648292401088 | 0.040031667687669 | 9601.01403556184 | 9897.68433822966 | 6988.29249616813 | 10227.5841641554 | 19688.165859252 | 19847.4229668482 |
| YDR047W | 7003.78976368839 | 1.8182833611409 | 0.384635503468184 | 4.72728945910033 | 2.27536793072e-06 | 2.91960827659725e-05 | 3419.05255356635 | 3018.93387028128 | 2844.91463598847 | 6455.95013684226 | 12606.3970222489 | 13677.4903632031 |
| YDR048C | 14.457355162842 | 1.53110163092789 | 0.587139760885148 | 2.607729424796 | 0.00911449686805704 | 0.0220749754908941 | 6.80333251870377 | 6.59515864616336 | 8.77834292410945 | 19.1674632942685 | 20.4266684892478 | 24.9731651045589 |
| YDR049W | 190.309155400896 | -3.81410519925602 | 0.623702389949478 | -6.11526468507677 | 9.63968300016336e-10 | 1.00128320195245e-07 | 501.934754713256 | 457.539131077583 | 107.534700820341 | 35.721181593864 | 15.712821914806 | 23.412342285524 |
| YDR050C | 228851.993866431 | 2.48730959651047 | 0.413434876763284 | 6.01620650870876 | 1.78551767174593e-09 | 1.51299129026892e-07 | 61157.4237881344 | 63635.8613819995 | 83023.3727904962 | 212397.274508784 | 472955.939635662 | 479942.091093508 |
| YDR051C | 912.599903940018 | -0.248517117488509 | 0.329085890151808 | -0.755174028804053 | 0.450144569524276 | 0.546968118440819 | 1121.79393975071 | 1103.04028357082 | 747.622205703322 | 721.393618529742 | 831.208279293239 | 950.541096792272 |
| YDR052C | 217.561971067687 | -2.42964404390672 | 0.520116166971355 | -4.67134882204216 | 2.99228288868901e-06 | 3.68457013444689e-05 | 486.816238005025 | 474.027027692991 | 140.453486785751 | 74.0561081824011 | 70.7076986166272 | 59.3112671233273 |
| YDR053W | 1.54314562073836 | -0.793490641838804 | 1.89074343883559 | -0.419671239122466 | 0.674725638427656 | 0.748661804182306 | 0 | 0.82439483077042 | 5.12070003906385 | 1.74249666311532 | 1.5712821914806 | 0 |
| YDR054C | 326.587545802407 | -3.84320640655304 | 0.656216430227933 | -5.85661411314881 | 4.72399035448306e-09 | 3.09971505153779e-07 | 781.627313815521 | 584.495935016227 | 468.178289285837 | 92.3523231451119 | 14.1415397233254 | 18.7298738284192 |
| YDR055W | 22168.0945009612 | 0.158441099651692 | 0.366382344220541 | 0.432447420436612 | 0.665416259237317 | 0.7417830551304 | 23138.1338961115 | 24153.1197519118 | 15564.0020044461 | 16832.517765694 | 29304.4128711133 | 24016.3807164905 |
| YDR056C | 1662.73549671801 | 0.0299623395652453 | 0.369672655721867 | 0.0810510031009388 | 0.935401391282988 | 0.953765826450672 | 1802.8831174565 | 1765.85372751024 | 1366.49538185304 | 1089.06041444707 | 1893.39504073413 | 2058.72529830707 |
| YDR057W | 335.83054964089 | -2.40814769940703 | 0.644675021596357 | -3.73544440025601 | 0.000187383978999735 | 0.000919080597683393 | 626.662517556158 | 630.662045539371 | 440.380203359491 | 224.782069541876 | 61.2800054677435 | 31.2164563806986 |
| YDR058C | 205.407299018063 | 0.22329987211669 | 0.356622937249924 | 0.626151177595734 | 0.531215786890777 | 0.623592721031099 | 159.500351271833 | 182.191257600263 | 226.042330295818 | 168.150927990628 | 267.117972551703 | 229.440954398135 |
| YDR059C | 713.394319654915 | 1.26544609066803 | 0.435215140932428 | 2.90763342460207 | 0.00364174953508731 | 0.0102459008326615 | 375.69514019953 | 378.397227323623 | 502.560132405266 | 514.907763950577 | 1257.02575318448 | 1251.77990086601 |
| YDR060W | 1148.64249272717 | -4.06623054366891 | 0.640644644372855 | -6.34709207262547 | 2.19422821872589e-10 | 3.84319824158969e-08 | 3091.73666683316 | 2787.27892283479 | 626.188461919808 | 222.168324547203 | 89.5630849143944 | 74.9194953136766 |
| YDR061W | 701.122980524342 | -0.151040955365093 | 0.47646826838488 | -0.317001079373213 | 0.751242783241016 | 0.809572209516757 | 650.096218453915 | 708.979554462561 | 855.888435100672 | 1143.07781100365 | 410.104651976438 | 438.591212148815 |
| YDR062W | 6569.72119048319 | 0.40746380964479 | 0.273824294507968 | 1.48804842308444 | 0.136738123905578 | 0.208375181720759 | 5859.18115027477 | 6196.97594290124 | 4888.07395157495 | 7300.18977012163 | 7675.71350538275 | 7498.1928226438 |
| YDR063W | 825.84286118874 | -0.445304197012452 | 0.310901876931211 | -1.43229819455538 | 0.152058499578121 | 0.226574904507889 | 929.03285172077 | 829.341199755042 | 1099.48745124471 | 828.557163311334 | 656.795956038892 | 611.842545061693 |
| YDR064W | 102923.565372695 | 0.513373232629118 | 0.436963108084178 | 1.17486630594503 | 0.240048207616062 | 0.329337549434904 | 102028.06593133 | 102677.551777625 | 49700.0515219997 | 77004.4125363922 | 136891.675803982 | 149239.634664844 |
| YDR065W | 168.893851651856 | -2.63865268078033 | 0.534029734873221 | -4.94102202269082 | 7.77141290041535e-07 | 1.27999741889194e-05 | 341.678477606011 | 287.713795938876 | 245.793601875065 | 80.1548465033047 | 40.8533369784957 | 17.1690510093842 |
| YDR066C | 265.609389840401 | 0.657313093705264 | 0.336324702471547 | 1.95440028304455 | 0.050653897262461 | 0.0915294888805412 | 223.754047281813 | 210.220681846457 | 183.613672829289 | 244.820781167702 | 351.967210891655 | 379.279945025488 |
| YDR067C | 415.670800297919 | -0.571918516229414 | 0.299079705827619 | -1.91226119688325 | 0.0558427002175743 | 0.0992508072085782 | 567.700302394059 | 499.583267446874 | 423.555046088281 | 324.975627671007 | 356.681057466097 | 321.529500721196 |
| YDR068W | 37.2080569400666 | -2.02883169428276 | 0.580042779530939 | -3.49772769505623 | 0.000469239969876634 | 0.00191867009905113 | 67.2773993516261 | 84.0882727385828 | 28.5296145033557 | 18.2962149627109 | 12.5702575318448 | 12.4865825522794 |
| YDR069C | 394.524372984512 | 0.319425262575608 | 0.344555361304938 | 0.927065135094242 | 0.353892716815884 | 0.450715409724612 | 305.394037506258 | 306.674877046596 | 440.380203359491 | 374.636782569794 | 535.807227294886 | 404.253110130047 |
| YDR070C | 114.82228956632 | 0.279943548649485 | 0.363762873671583 | 0.769577020942021 | 0.441550840281784 | 0.538008607610158 | 112.632949476318 | 85.7370624001236 | 111.923872282396 | 96.7085648029002 | 147.700525999177 | 134.230762437004 |
| YDR071C | 1671.81126307035 | -1.6436436139733 | 0.473127986855859 | -3.47399363308864 | 0.000512773201056508 | 0.00206520288605623 | 2852.86410284311 | 2525.94576148057 | 2222.38381695371 | 1387.89859217135 | 575.089282081901 | 466.686022891444 |
| YDR072C | 5396.11341703342 | 0.935278296025674 | 0.399423787129459 | 2.34156884532903 | 0.0192028840019913 | 0.0410373474847305 | 3706.30437102273 | 4278.60917169848 | 3131.67383817605 | 4114.03462161527 | 9207.71364207634 | 7938.34485761165 |
| YDR073W | 268.360953677406 | -2.33212924761838 | 0.54409405464195 | -4.28626122215776 | 1.81705310455019e-05 | 0.00014590800490403 | 526.880307281836 | 431.158496492929 | 387.710145814834 | 167.279679659071 | 54.9948767018211 | 42.1422161139431 |
| YDR074W | 3889.32743889059 | 0.868447780960578 | 0.377015616716369 | 2.30347959727598 | 0.0212518738083144 | 0.0447115541736506 | 2217.13047526202 | 2267.91017944942 | 3773.95592879006 | 6385.37902198609 | 4594.42912788929 | 4097.15989996669 |
| YDR075W | 630.613492709386 | 0.471000813774814 | 0.38858437966591 | 1.21209404809262 | 0.225476381822671 | 0.313350862955978 | 583.574744937701 | 555.642115939263 | 445.500903398555 | 443.465400762849 | 873.632898463216 | 881.864892754735 |
| YDR076W | 72.7428714252539 | -1.83808318137516 | 0.449466536193775 | -4.08947726551711 | 4.3234643976558e-05 | 0.000291856506508421 | 131.531095361606 | 100.576169353991 | 110.460815128377 | 45.3049132409983 | 29.8543616381315 | 18.7298738284192 |
| YDR077W | 59092.2946144218 | -1.91348003169596 | 0.642108946392905 | -2.97999279163617 | 0.00288255166549984 | 0.00842270087378357 | 84218.4532490339 | 89462.5026403752 | 106501.050941027 | 53977.3191333232 | 11599.2051375098 | 8795.23658526183 |
| YDR078C | 213.126459157632 | 0.684002110113692 | 0.438556641396878 | 1.55966651863948 | 0.118838708201495 | 0.185397597097294 | 184.445903840413 | 170.649729969477 | 134.601258169678 | 137.65723638611 | 315.827720487601 | 335.57690609251 |
| YDR079C-A | 168.116845876009 | -0.0732718876647433 | 0.429078005955477 | -0.170765890229168 | 0.864407850716418 | 0.899900833917513 | 215.438863092286 | 198.679154215671 | 102.414000781277 | 121.103518086515 | 172.841041062866 | 198.224498017436 |
| YDR079W | 391.737521287953 | 1.32964908644578 | 0.350337257749587 | 3.79534022440795 | 0.000147441123933734 | 0.00075900946293625 | 207.123678902759 | 230.006157784947 | 231.163030334882 | 386.834259211601 | 675.65134233666 | 619.646659156867 |
| YDR080W | 506.554370588779 | -0.949108789986908 | 0.283427097850069 | -3.34868753618251 | 0.000811953095499916 | 0.0029999873408029 | 697.719546084842 | 696.613632001004 | 608.631776071589 | 375.508030901351 | 328.397978019446 | 332.45526045444 |
| YDR081C | 643.780638717738 | -1.64667862449627 | 0.436027293670611 | -3.7765494233951 | 0.000159016017636698 | 0.000805081095582024 | 1057.54024374073 | 978.556664124488 | 893.927921105146 | 503.581535640327 | 212.123095849882 | 216.954371845855 |
| YDR082W | 270.739673744371 | -0.700745027378619 | 0.458649231097723 | -1.52784520253412 | 0.126550976185323 | 0.195253542557135 | 358.308845985065 | 347.070223754347 | 302.121302304767 | 338.044352644372 | 160.270783531022 | 118.622534246655 |
| YDR083W | 765.858713785499 | -0.485375885206088 | 0.367641614142473 | -1.32024195992674 | 0.186754246738287 | 0.268699139632388 | 1051.49283705744 | 962.89316233985 | 664.959476501291 | 459.147870730887 | 765.214427251054 | 691.444508832474 |
| YDR084C | 1929.19380133533 | 1.34535333486574 | 0.388449603799626 | 3.46339221795091 | 0.000533410246363527 | 0.00213762413601812 | 1079.46209296766 | 1123.65015434008 | 1065.10560812528 | 1593.51319841896 | 3332.68952813036 | 3380.74222602966 |
| YDR085C | 328.244447394696 | -2.59638802104961 | 0.396353656039966 | -6.55068518098342 | 5.72736859740911e-11 | 1.5087005818353e-08 | 646.316589276858 | 659.515864616336 | 385.515560083807 | 116.747276428726 | 91.134367105875 | 70.2370268565718 |
| YDR086C | 3601.69639308465 | -0.378960087348573 | 0.386493537912254 | -0.980508210811557 | 0.326835318623415 | 0.422315299344863 | 4744.19054304276 | 4568.79615212966 | 2901.97386499518 | 2099.70847905396 | 3785.21879927677 | 3510.29052000956 |
| YDR087C | 1241.60307478809 | -1.19552637923171 | 0.46509111127965 | -2.57052080815379 | 0.0101545726225235 | 0.024131161508875 | 1954.0682845388 | 1962.0596972336 | 1271.39666684185 | 1242.40012080122 | 540.521073869328 | 479.172605443724 |
| YDR088C | 13.1682733145102 | -2.71612380352214 | 0.871126319290462 | -3.11794483001552 | 0.00182116890768694 | 0.00577695894068696 | 22.6777750623459 | 35.448977723128 | 10.9729286551368 | 5.22748998934596 | 0 | 4.68246845710479 |
| YDR089W | 588.423758120736 | 0.235964299041665 | 0.329163029811008 | 0.716861487078746 | 0.473459567683346 | 0.568858137291184 | 461.870685436444 | 462.485500062205 | 697.146733889692 | 696.998665246128 | 595.515950571149 | 616.525013518797 |
| YDR090C | 1016.98252371049 | 0.604083874522345 | 0.320157764952295 | 1.88683187056968 | 0.0591829429906025 | 0.104079233440601 | 799.769533865398 | 893.643996555135 | 727.139405547066 | 931.364466435138 | 1378.01448192849 | 1371.9632579317 |
| YDR091C | 11526.2532794136 | 0.646545702141725 | 0.353106088694281 | 1.8310239410839 | 0.0670969697329263 | 0.115197143449759 | 9627.47143980124 | 10555.5514131845 | 6773.95462310446 | 10412.2888104456 | 15321.5726491274 | 16466.6807408185 |
| YDR092W | 1372.21626877176 | 0.684158209654221 | 0.361756172540562 | 1.89121364495172 | 0.0585958306139785 | 0.10327234514341 | 839.077677306798 | 832.638779078124 | 1486.46606848253 | 1633.59062167061 | 1627.84835037391 | 1813.67611571859 |
| YDR093W | 3352.3741178304 | 0.808292022851302 | 0.258981705861204 | 3.12103907171146 | 0.00180214121818533 | 0.00574590547034886 | 2431.81341251889 | 2502.03831138822 | 2377.46787527964 | 4296.12552291082 | 4033.48138553071 | 4473.31819935411 |
| YDR094W | 6.39484378399774 | 0.318943134209493 | 0.829032316926326 | 0.384717371925848 | 0.700446823079491 | 0.769774324339919 | 7.5592583541153 | 4.1219741538521 | 5.12070003906385 | 4.3562416577883 | 6.28512876592241 | 10.9257597332445 |
| YDR095C | 6.44763549204929 | 3.51718006553355 | 1.1510656467371 | 3.05558599155802 | 0.00224621180771488 | 0.00684924433791848 | 0 | 0.82439483077042 | 2.19458573102736 | 4.3562416577883 | 14.1415397233254 | 17.1690510093842 |
| YDR096W | 502.190951319612 | -1.24128459619309 | 0.417777353803316 | -2.97116295292892 | 0.00296674319796879 | 0.00860622801572928 | 672.01806768085 | 674.354971570203 | 773.225705898641 | 464.375360720233 | 226.264635573207 | 202.906966474541 |
| YDR097C | 843.219699241547 | 0.189708762070735 | 0.33288526780535 | 0.56989233354017 | 0.5687507249618 | 0.657704196220864 | 906.355076658424 | 845.00470153968 | 611.557890379625 | 736.204840166222 | 908.201106675789 | 1051.99458002954 |
| YDR098C | 2577.6534484604 | 0.758368386957416 | 0.285878591449619 | 2.65276382926024 | 0.00798357090227803 | 0.0197519003498542 | 1779.44941655874 | 1871.37626584885 | 2095.82937313113 | 3717.61663075653 | 2856.59102411174 | 3145.05798035538 |
| YDR099W | 4026.1451916164 | -1.42047114421447 | 0.651099309226931 | -2.1816505164182 | 0.0291353355678227 | 0.0578217445475433 | 5237.05418773108 | 5257.16583582297 | 7094.36413983446 | 4734.36343368432 | 1165.89138607861 | 668.03216654695 |
| YDR100W | 1366.37260354718 | 1.65357854659958 | 0.410608102250259 | 4.02714543998879 | 5.64581121935182e-05 | 0.000359278895776934 | 628.174369226981 | 632.310835200912 | 716.16647689193 | 1113.45536773069 | 2512.48022417749 | 2595.64834805509 |
| YDR101C | 6634.0384418318 | 0.329955906358571 | 0.321789278776952 | 1.0253788056975 | 0.305184484633342 | 0.400406485620874 | 6112.41630513763 | 6535.80221834789 | 4989.02489520221 | 9284.02222107842 | 6555.38930285708 | 6327.57570836761 |
| YDR102C | 15.9619736799828 | 1.09984287985174 | 0.584888306381179 | 1.88043232845035 | 0.0600491810578328 | 0.105372404908023 | 9.82703586034988 | 11.5415276307859 | 8.77834292410945 | 13.9399733049226 | 26.7117972551703 | 24.9731651045589 |
| YDR103W | 1124.05969363567 | -0.362418992044271 | 0.304925223481195 | -1.18855038591657 | 0.234616641322464 | 0.323193833180036 | 1256.34873845396 | 1207.73842707866 | 1330.65048157959 | 1193.61021423399 | 950.625725845765 | 805.384574622024 |
| YDR104C | 333.229089485508 | -1.13862957818266 | 0.292559970987053 | -3.89195273140442 | 9.94406216770527e-05 | 0.000556774880914709 | 439.192910374099 | 499.583267446874 | 436.722560474445 | 223.039572878761 | 190.125145169153 | 210.711080569716 |
| YDR105C | 2183.84949629807 | 1.26843675702762 | 0.357907547162368 | 3.54403467343533 | 0.000394053418614773 | 0.00167131248889363 | 1278.2705876809 | 1310.78778092497 | 1253.83998099363 | 1980.34745763056 | 3543.24134178876 | 3736.60982876962 |
| YDR106W | 33.9921520395982 | 0.0524069512456788 | 0.447941160617344 | 0.116995167788226 | 0.906863882730944 | 0.933088896754638 | 25.701478403992 | 34.6245828923576 | 39.5025431584925 | 30.4936916045181 | 40.8533369784957 | 32.7772791997335 |
| YDR107C | 556.983042769638 | 1.37754295008343 | 0.413298193291547 | 3.33304856503857 | 0.000858999467726712 | 0.0031360298028118 | 253.991080698274 | 281.943032123483 | 392.099317276889 | 474.830340698924 | 1011.90573131351 | 927.128754506748 |
| YDR108W | 557.127256351307 | -0.425888689321333 | 0.339157401156453 | -1.25572577177778 | 0.209215411984109 | 0.294695374710774 | 555.605489027474 | 642.203573170157 | 719.824119776975 | 609.002583758804 | 441.53029580605 | 374.597476568383 |
| YDR109C | 360.066614970561 | -0.0567532230991862 | 0.329879173710419 | -0.172042455608327 | 0.863404151832263 | 0.899146626422991 | 356.04106847883 | 368.504489354378 | 378.200274313716 | 454.791629073098 | 292.258487615392 | 310.603740987951 |
| YDR110W | 299.916386070909 | -2.22210619545718 | 0.515459891380421 | -4.31091969058213 | 1.62576947582341e-05 | 0.000134575262523171 | 572.235857406528 | 581.198355693146 | 330.650916808123 | 183.833397958666 | 70.7076986166272 | 60.8720899423623 |
| YDR111C | 1697.73068691578 | 0.936672395114858 | 0.379572573358288 | 2.46770304510571 | 0.0135983075889179 | 0.0307922295614034 | 1378.05279795522 | 1258.02651175566 | 858.814549408708 | 1526.42707688902 | 2389.920213242 | 2775.14297224411 |
| YDR113C | 92.5276847979992 | -3.85418983306312 | 0.770245964976003 | -5.00384293890225 | 5.61985507894747e-07 | 9.99775323437063e-06 | 251.723303192039 | 225.059788800325 | 43.8917146205473 | 23.5237049520568 | 6.28512876592241 | 4.68246845710479 |
| YDR114C | 8.79540358452507 | 0.64774910594578 | 0.795941085044646 | 0.813815391762879 | 0.415750716183624 | 0.51301678716661 | 8.31518418952682 | 8.2439483077042 | 3.6576428850456 | 4.3562416577883 | 15.712821914806 | 12.4865825522794 |
| YDR115W | 959.19495612328 | 1.09729152138667 | 0.372568117945775 | 2.94521046899235 | 0.0032273504375281 | 0.00924972711067245 | 632.70992423945 | 609.22777993934 | 590.343561646361 | 803.290961696162 | 1568.13962709764 | 1551.45788212072 |
| YDR116C | 817.626100685363 | 0.362680008611225 | 0.373552568409159 | 0.970894163988118 | 0.331600984634007 | 0.427529597806407 | 670.506216010027 | 601.808226462406 | 874.908178102909 | 1265.92382575328 | 773.070838208457 | 719.539319575103 |
| YDR117C | 519.129830757714 | -2.14075518704922 | 0.665756425358182 | -3.21552313355064 | 0.0013020699975602 | 0.00439943902638388 | 873.094339900317 | 878.804889601267 | 789.319334592842 | 429.525427457926 | 65.9938520421854 | 78.0411409517465 |
| YDR118W | 291.41648133305 | -0.630641276779689 | 0.334425426910631 | -1.88574559836987 | 0.0593292450364293 | 0.104222678132734 | 380.986621047411 | 366.031304862066 | 316.751873844949 | 290.125694408701 | 191.696427360634 | 202.906966474541 |
| YDR118W-A | 10.9037803874311 | -0.266246282911957 | 0.63859860804516 | -0.416922742326317 | 0.676734918216977 | 0.750440156125058 | 12.850739201996 | 10.7171328000155 | 12.4359858091551 | 12.1974766418072 | 7.85641095740302 | 9.36493691420958 |
| YDR119W | 5472.38281305038 | 1.24602133676177 | 0.313518543214652 | 3.97431464176163 | 7.05822052878189e-05 | 0.000428416024555658 | 3214.95257800523 | 3303.35008689707 | 3218.72573884013 | 5796.41514985311 | 8076.3904642103 | 9224.46286049644 |
| YDR119W-A | 199.926238802288 | 1.24598567230766 | 0.498802823855229 | 2.49795232247782 | 0.0124912992686494 | 0.0286481364993241 | 140.602205386544 | 145.917885046364 | 68.7636862388574 | 141.142229712341 | 348.824646508694 | 354.306779920929 |
| YDR120C | 1651.91609255381 | -1.47413448948275 | 0.811786532617258 | -1.81591395059244 | 0.0693835729700249 | 0.118680002636643 | 2480.94859182064 | 2316.54947446488 | 2492.31786187008 | 2236.49446710851 | 183.840016403231 | 201.346143655506 |
| YDR121W | 493.004064653519 | -2.28922886670604 | 0.683845560232226 | -3.34758167608582 | 0.000815199620444419 | 0.00301025547916402 | 920.717667531243 | 923.32221046287 | 613.752476110653 | 378.121775896024 | 54.9948767018211 | 67.115381218502 |
| YDR122W | 734.483803425488 | -0.644674487005595 | 0.355881170049993 | -1.81148805067443 | 0.0700653371113882 | 0.119623746287736 | 828.494715611036 | 789.770247878062 | 1070.95783674135 | 764.084786776068 | 458.814399912336 | 494.780833634073 |
| YDR123C | 426.888374805855 | -1.1108744639884 | 0.82724136433315 | -1.34286619587004 | NA | NA | 442.216613715745 | 459.187920739124 | 850.767735061608 | 682.187443609648 | 81.7066739569914 | 45.263861752013 |
| YDR124W | 213.054428957518 | 0.995389668860384 | 0.517858077195323 | 1.92212830637176 | 0.0545896151110326 | 0.0973842441315927 | 98.2703586034988 | 98.1029848616799 | 231.163030334882 | 435.62416577883 | 232.549764339129 | 182.616269827087 |
| YDR125C | 193.803279302296 | -0.00846999367195099 | 0.324232789725936 | -0.0261231866126508 | 0.979159083129217 | 0.984817194338928 | 170.839238803006 | 192.083995569508 | 219.458573102736 | 163.79468633284 | 219.979506807285 | 196.663675198401 |
| YDR126W | 413.374428679741 | 0.0231795629757776 | 0.307684998330789 | 0.0753353692949874 | 0.939947880800594 | 0.957340558651878 | 442.216613715745 | 422.090153354455 | 365.032759927551 | 345.885587628391 | 430.531320465685 | 474.490136986619 |
| YDR127W | 10937.4620451516 | 0.663065347339492 | 0.300624974713968 | 2.20562296253121 | 0.027410409929094 | 0.0550050628325874 | 7753.53129381606 | 8336.28052875048 | 9311.6272567491 | 11469.113036625 | 12538.8318880152 | 16215.3882669539 |
| YDR128W | 886.963819770793 | -0.432440060445913 | 0.355341733045545 | -1.21696952603787 | 0.223615810642507 | 0.311398567983298 | 1031.83876533674 | 995.868955570667 | 1030.72376500585 | 1049.85423952698 | 579.803128656343 | 633.694064528182 |
| YDR129C | 11388.9398548361 | 1.35038464153255 | 0.30763358313963 | 4.389587858877 | 1.13565689126224e-05 | 0.000101181934648335 | 5791.90375092314 | 6015.60908013175 | 7441.84021391379 | 13190.699739783 | 18190.7339307709 | 17702.8524134942 |
| YDR130C | 91.3297034576176 | -3.499381364568 | 0.580448314542739 | -6.02875618189143 | 1.6522633132924e-09 | 1.43791564021663e-07 | 219.974418104755 | 208.571892184916 | 75.3474434319395 | 17.4249666311532 | 18.8553862977672 | 7.80411409517465 |
| YDR131C | 365.090521667092 | -0.777111993484541 | 0.325233519534357 | -2.38939699265053 | 0.0168760555258907 | 0.0368537801243594 | 426.342171172103 | 437.753655139093 | 520.116818253485 | 312.7781510292 | 284.402076657989 | 209.150257750681 |
| YDR132C | 393.186102983884 | -0.125339753033515 | 0.286878396625859 | -0.436908998752459 | 0.662177345563829 | 0.7388118685778 | 405.932173615991 | 422.914548185225 | 401.609188778007 | 356.340567607083 | 425.817473891244 | 346.502665825754 |
| YDR133C | 15724.5634726138 | 1.07915688300988 | 0.48196699720983 | 2.23906800518968 | 0.0251514916631487 | 0.0512496354443822 | 7171.46840054918 | 7440.16334770304 | 15697.1402054617 | 11760.9812276968 | 26410.111074406 | 25867.5165798659 |
| YDR135C | 4488.55564653753 | 0.5238570106847 | 0.325133706811318 | 1.6112048665219 | 0.107135077469778 | 0.169771136541676 | 3404.68996269353 | 3572.10280172823 | 4071.68805963277 | 6840.17065105919 | 4346.16654163535 | 4696.5158624761 |
| YDR136C | 9.3980560571274 | 2.05927799250653 | 0.759406705217061 | 2.71169319201353 | 0.00669405200494905 | 0.0170192242052396 | 4.53555501246918 | 3.29757932308168 | 2.92611430803648 | 9.58373164713426 | 21.9979506807285 | 14.0474053713144 |
| YDR137W | 539.998062736132 | -1.63640941635196 | 0.401761798472446 | -4.07308365945648 | 4.63947498136573e-05 | 0.000307389083127524 | 913.158409177128 | 957.122398524457 | 583.02827587627 | 368.53804424889 | 210.551813658401 | 207.589434931646 |
| YDR138W | 298.742070209914 | -1.84688491118673 | 0.358501798667435 | -5.15167543943063 | 2.5816955217053e-07 | 5.84116648245245e-06 | 541.998823990067 | 496.285688123793 | 365.76428850456 | 162.923438001282 | 108.418471212162 | 117.06171142762 |
| YDR139C | 987.464339598085 | 0.540910003566817 | 0.309494022256778 | 1.74772358969195 | 0.0805119019957172 | 0.134221239671866 | 879.141746583609 | 910.956288001314 | 623.262347611771 | 1104.74288441511 | 1156.46369292972 | 1250.21907804698 |
| YDR140W | 1170.36558556917 | 0.807403775046211 | 0.29604528618795 | 2.72729819630912 | 0.00638553034422343 | 0.0163835917995215 | 771.800277955172 | 841.707122216598 | 939.282692879711 | 1248.49885912213 | 1546.14167641691 | 1674.76288482448 |
| YDR141C | 2112.94144314609 | 0.216231143455425 | 0.288498605760344 | 0.749504985944534 | 0.453552894614286 | 0.550426246267105 | 1790.78830408991 | 1940.62543163357 | 2132.40580198159 | 1916.74632942685 | 2462.19919405011 | 2434.88359769449 |
| YDR142C | 1057.20147712589 | 0.690249891701027 | 0.28977958995206 | 2.3819824295259 | 0.0172197190428171 | 0.0374516735538414 | 849.660639002559 | 807.082539324241 | 769.568063013595 | 1087.31791778396 | 1378.01448192849 | 1451.56522170248 |
| YDR143C | 1125.18954643673 | -0.982967371328947 | 0.644124579861514 | -1.52605163979348 | 0.126997004467957 | 0.19575411890226 | 1467.25204653378 | 1396.52484332509 | 1621.06732665221 | 1657.11432662267 | 303.257462955757 | 305.921272530846 |
| YDR144C | 6204.56120851792 | 0.637330604775433 | 0.299689008700572 | 2.12663990427559 | 0.0334500142614335 | 0.0649240783133307 | 4835.65756912755 | 5474.80607114636 | 4256.76478961608 | 6245.97928893686 | 8054.39251352957 | 8359.76701875108 |
| YDR145W | 342.406884951656 | -1.74743574492689 | 0.487533923614848 | -3.58423416358481 | 0.000338068688497136 | 0.00147105564454159 | 672.01806768085 | 686.72089403176 | 223.847744564791 | 135.043491391437 | 185.411298594711 | 151.399813446388 |
| YDR146C | 189.427970770704 | -3.41072424236701 | 0.591075817391424 | -5.77036674824467 | 7.90991920571843e-09 | 4.21739751401558e-07 | 484.54846049879 | 417.968179200603 | 137.527372477715 | 47.9186582356713 | 32.9969260210927 | 15.6082281903493 |
| YDR147W | 371.881555771563 | -0.327708554628497 | 0.291163798761088 | -1.12551270461132 | 0.260371836661515 | 0.351087652449781 | 418.026986982576 | 443.524418954486 | 379.663331467734 | 296.224432729604 | 361.394904040539 | 332.45526045444 |
| YDR148C | 2900.61448585827 | 0.746378410724811 | 0.369743844680667 | 2.0186364735008 | 0.0435250171711196 | 0.0809183344636289 | 1948.02087785551 | 1938.97664197203 | 2613.75160565359 | 5060.21030968689 | 2899.01564328171 | 2943.71183669988 |
| YDR150W | 674.276756152038 | -3.76889849814756 | 0.665360833361298 | -5.66444297466035 | 1.47502904583507e-08 | 6.51021226535105e-07 | 1778.69349072333 | 1581.18928541766 | 411.119060279126 | 175.992162974647 | 50.2810301273793 | 48.3855073900828 |
| YDR151C | 809.971870774327 | -1.12206061667237 | 0.429967685858795 | -2.60963940681082 | 0.00906377072378516 | 0.0219851915108009 | 1142.95986314223 | 1140.13805095549 | 1049.01197943108 | 819.844679995758 | 358.252339657578 | 349.624311463824 |
| YDR152W | 656.310533150462 | -2.87892767844677 | 0.547363633414985 | -5.25962541662702 | 1.44349148633501e-07 | 3.74059318155718e-06 | 1449.1098264839 | 1305.84141194034 | 713.971891160902 | 285.769452750912 | 83.277956148472 | 99.8926604182355 |
| YDR153C | 459.706889741418 | -3.05501288102602 | 0.615633364788103 | -4.96239004537632 | 6.96309864687092e-07 | 1.16473650093114e-05 | 1179.24430324199 | 1075.8352541554 | 207.75411587059 | 134.17224305988 | 70.7076986166272 | 90.5277235040259 |
| YDR154C | 3908.0567556945 | 2.75133614414333 | 0.529081694648174 | 5.20021042491916 | 1.99063030646796e-07 | 4.76567255526159e-06 | 721.909172818011 | 730.413820062592 | 1579.37019776269 | 2962.24432729604 | 8868.31668871653 | 8586.08632751115 |
| YDR155C | 20303.5154807929 | 2.76641611218546 | 0.515772832658279 | 5.36363285737139 | 8.15646080167078e-08 | 2.44314453780278e-06 | 3660.19289506263 | 3724.61584542076 | 8224.57579131355 | 16840.359000678 | 45752.5948515322 | 43618.7545007502 |
| YDR156W | 2410.0114107238 | 0.966301847517554 | 0.291347605356908 | 3.31666308474996 | 0.000910994015799933 | 0.00329966336431472 | 1511.09574498765 | 1640.54571323313 | 1743.23259901274 | 2783.63841932672 | 3015.29052545128 | 3766.26546233129 |
| YDR157W | 174.082475553325 | 1.23129627515924 | 0.411573495941716 | 2.99168019150974 | 0.00277446746849851 | 0.0081661656751053 | 122.459985336668 | 120.361645292481 | 68.7636862388574 | 170.764672985301 | 271.831819126144 | 290.313044340497 |
| YDR158W | 32650.2651169994 | 1.11919401858777 | 0.348796460743949 | 3.20873100661812 | 0.00133322160406482 | 0.0044858657942411 | 18919.3118086798 | 19915.7303217518 | 22918.7903176958 | 30126.0248086008 | 50048.4803630402 | 53973.2530822279 |
| YDR159W | 382.429830318812 | -2.40344523456698 | 0.442049696799874 | -5.43704758077252 | 5.4170676070756e-08 | 1.73561768107298e-06 | 755.92583541153 | 712.277133785642 | 463.789117823783 | 185.575894621781 | 95.8482136803168 | 81.1627865898164 |
| YDR160W | 974.041177275545 | 0.747838298981488 | 0.308416070695517 | 2.42477085352594 | 0.015318051989635 | 0.0340401155325222 | 698.475471920253 | 726.29184590874 | 755.669020050422 | 954.016923055637 | 1437.72320520475 | 1272.07059751347 |
| YDR161W | 1670.83202856595 | -0.703730924137528 | 0.504236487850411 | -1.39563665282846 | 0.162823939152033 | 0.240005989503111 | 2285.16380044905 | 2199.48540849548 | 1728.60202747255 | 2295.73935365443 | 769.928273825496 | 746.073307498697 |
| YDR162C | 338.929207308943 | -1.83160158301983 | 0.396801960016786 | -4.61590860826984 | 3.91379164655224e-06 | 4.50790718513266e-05 | 565.432524887824 | 461.661105231435 | 562.545475720014 | 215.198337894742 | 128.84513970141 | 99.8926604182355 |
| YDR163W | 63.1191524612994 | -2.11328784683054 | 0.472627169991959 | -4.47136343614459 | 7.77224847113687e-06 | 7.57235705811217e-05 | 123.215911172079 | 122.834829784793 | 61.4484004687662 | 22.6524566204991 | 26.7117972551703 | 21.851519466489 |
| YDR164C | 762.227690492353 | -1.37893542931008 | 0.763901860336482 | -1.80512118232399 | 0.0710557210565378 | 0.121025877705396 | 1070.39098294273 | 1095.62073009389 | 1138.9899944032 | 1042.88425287452 | 108.418471212162 | 117.06171142762 |
| YDR165W | 2041.58025748194 | -1.12654395032592 | 0.406832581729493 | -2.76906029880117 | 0.00562182296459074 | 0.0147592906204502 | 3056.96407840423 | 3099.72456369678 | 2246.52425999501 | 1826.13650294485 | 1236.59908469524 | 783.533055155535 |
| YDR166C | 739.878222894014 | -1.00949610478884 | 0.287515510764687 | -3.5111013736405 | 0.000446254187455888 | 0.00184696463188684 | 1074.17061211978 | 1043.68385575535 | 847.841620753571 | 469.602850709579 | 523.236969763041 | 480.733428262758 |
| YDR167W | 1137.19516686703 | -0.761482358329665 | 0.367263114657126 | -2.07339732180995 | 0.0381353179898049 | 0.0724672315887706 | 1552.67166593528 | 1497.10101267908 | 1243.59858091551 | 1170.95775761349 | 608.086208102994 | 750.755775955801 |
| YDR168W | 845.982932804588 | -2.54074405411575 | 0.396690266775456 | -6.40485604743592 | 1.50511321165607e-10 | 2.93725123729246e-08 | 1656.98943122207 | 1685.88742892551 | 990.48969327035 | 335.430607649699 | 193.267709552114 | 213.832726207785 |
| YDR169C | 203.512573587994 | -2.78090091094723 | 0.567962862999461 | -4.89627243630165 | 9.767163438499e-07 | 1.51933653487762e-05 | 473.965498803029 | 431.9828913237 | 161.667815519016 | 84.511088161093 | 40.8533369784957 | 28.0948107426287 |
| YDR169C-A | 2.50794961455853 | 0.400571683879161 | 1.3190907704529 | 0.303672569660711 | 0.761377357928648 | 0.817283400158721 | 0.75592583541153 | 4.1219741538521 | 1.46305715401824 | 0.87124833155766 | 4.71384657444181 | 3.12164563806986 |
| YDR170C | 3466.66197343454 | -0.14020561269464 | 0.306343779337946 | -0.457674097374019 | 0.647186600158687 | 0.726364884109784 | 3728.22622024966 | 3910.1046823441 | 3267.73815349974 | 3959.82366692956 | 3257.26798293929 | 2676.8111346449 |
| YDR170W-A | 42.1990352089409 | -2.19964514962343 | 0.785735613595502 | -2.79947238175691 | 0.00511861950665418 | 0.0136383573118961 | 71.0570285286838 | 74.1955347693378 | 64.3745147768027 | 35.721181593864 | 6.28512876592241 | 1.56082281903493 |
| YDR171W | 450.19211001751 | -1.51306571886646 | 0.603177336734307 | -2.50849232343248 | 0.0121247602653743 | 0.0279268441019351 | 887.456930773136 | 648.79873181632 | 465.98370355481 | 462.632864057117 | 89.5630849143944 | 146.717344989283 |
| YDR172W | 4074.62298797785 | -1.60174479202637 | 0.58447923371155 | -2.74046484398598 | 0.00613523451175791 | 0.015855100423644 | 7790.57165975122 | 7931.50266684221 | 2668.61624892927 | 3616.55182429585 | 1312.0206298863 | 1128.47489816225 |
| YDR173C | 174.943822903576 | 0.712189017945936 | 0.392249602609244 | 1.81565261815042 | 0.0694236766262185 | 0.118717067836656 | 161.768128778067 | 121.186040123252 | 114.118458013423 | 148.112216364802 | 284.402076657989 | 220.076017483925 |
| YDR174W | 4428.43637744393 | -1.09381082864241 | 0.732444722295915 | -1.49336980026801 | NA | NA | 6047.40668329224 | 5891.94985551619 | 6155.81297553175 | 6787.02450283417 | 879.918027229138 | 808.506220260094 |
| YDR175C | 572.376111550702 | -0.310523134508276 | 0.334974353472348 | -0.927005698464345 | 0.353923575617603 | 0.450715409724612 | 610.788075012516 | 636.432809354764 | 655.449605000172 | 667.376221973167 | 395.963112253112 | 468.246845710479 |
| YDR176W | 31.6357720776844 | -3.09158716716879 | 0.833168380097314 | -3.71063909891502 | 0.000206736662268097 | 0.000991350785559603 | 86.9314710723259 | 65.9515864616336 | 18.288214425228 | 13.9399733049226 | 3.14256438296121 | 1.56082281903493 |
| YDR177W | 1272.89912351043 | -0.98368181686475 | 0.67504117154567 | -1.45721751254427 | 0.145056347951628 | 0.218496828123119 | 1589.71203187045 | 1429.50063655591 | 2054.86377281862 | 1904.54885278504 | 249.833868445416 | 408.935578587152 |
| YDR178W | 2726.09775957987 | 1.39607035940092 | 0.404271890736996 | 3.45329564431464 | 0.000553781713022589 | 0.00221018199271662 | 1404.51020219462 | 1490.50585403292 | 1607.89981226605 | 2155.46837227365 | 4811.26607031361 | 4886.93624639837 |
| YDR179C | 2.05252867522781 | -4.11466934423529 | 1.72792475811939 | -2.38127807643289 | 0.0172526827507269 | 0.0374855860036036 | 4.53555501246918 | 4.1219741538521 | 3.6576428850456 | 0 | 0 | 0 |
| YDR179W-A | 692.676569665682 | 0.509045724670639 | 0.343632755869239 | 1.48136554497832 | 0.138509190964778 | 0.210476448752518 | 560.141044039943 | 522.666322708446 | 631.309161958871 | 573.28140216494 | 991.479062824261 | 877.182424297631 |
| YDR180W | 676.691277763537 | -0.821008902372838 | 0.322630938466106 | -2.54473085029039 | 0.0109361996919283 | 0.025673137814845 | 961.537662643466 | 902.712339693609 | 727.139405547066 | 385.091762548486 | 537.378509486367 | 546.287986662226 |
| YDR181C | 189.444139223872 | -2.1840886877067 | 0.50334081998761 | -4.33918450675322 | 1.43012416762519e-05 | 0.000121664460231258 | 402.152544438934 | 389.114360123638 | 140.453486785751 | 68.8286181930551 | 48.7097479358987 | 87.4060778659561 |
| YDR182W | 1560.11510035814 | -0.0353023097013983 | 0.36078967353792 | -0.097847339573836 | 0.922053516164761 | 0.944943450684447 | 1432.47945810485 | 1493.803433356 | 1812.7278138286 | 2134.55841231627 | 1282.16626824817 | 1204.95521629497 |
| YDR182W-A | 47.9440203618151 | 0.114392248568327 | 0.419925165519998 | 0.272411034062875 | 0.785305989451675 | 0.836343736078846 | 34.0166625935188 | 48.6392950154547 | 55.5961718526932 | 52.2748998934596 | 54.9948767018211 | 42.1422161139431 |
| YDR183C-A | 122.042089153316 | -1.11578790715648 | 0.375233547419046 | -2.97358249237351 | 0.00294345275167443 | 0.00856180475193467 | 173.10701630924 | 158.283807507921 | 168.251572712098 | 54.0173965565749 | 98.990778063278 | 79.6019637707814 |
| YDR183W | 238.399318040907 | -1.58875082283572 | 0.406500342252273 | -3.90836281719473 | 9.29236866580387e-05 | 0.000529582780599796 | 442.216613715745 | 394.885123939031 | 237.746787527964 | 155.082203017263 | 102.133342446239 | 98.3318375992006 |
| YDR184C | 489.823368253822 | -2.96759940642702 | 0.508003982128631 | -5.84168532300127 | 5.16753490161924e-09 | 3.16942140632647e-07 | 1037.13024618462 | 1013.18124701685 | 557.42477568095 | 175.992162974647 | 103.70462463772 | 51.5071530281527 |
| YDR185C | 445.629984973642 | 1.42533498050163 | 0.371360803908506 | 3.83814060477099 | 0.000123969493224547 | 0.000659259732754814 | 226.777750623459 | 238.250106092651 | 259.692644838238 | 415.585454153004 | 815.495457378433 | 717.978496756068 |
| YDR186C | 379.850506688807 | -3.99449589327022 | 0.647801654126757 | -6.16623293229289 | 6.99360417202493e-10 | 7.90154576628782e-08 | 1044.68950453873 | 887.048837908971 | 214.337873063672 | 76.669853177074 | 25.1405150636897 | 31.2164563806986 |
| YDR187C | 2.08434069994684 | 1.11836856279626 | 1.37806799787874 | 0.81154817071274 | 0.417050941552877 | 0.514506687908103 | 1.51185167082306 | 0.82439483077042 | 1.46305715401824 | 0.87124833155766 | 4.71384657444181 | 3.12164563806986 |
| YDR188W | 7316.00741613019 | 0.958416033115721 | 0.383846463281439 | 2.49687342413522 | 0.0125293670814933 | 0.0287149907490452 | 4868.91830588566 | 4828.48052382235 | 5217.99333980606 | 13488.6666691757 | 5985.01386734962 | 9506.97179074176 |
| YDR189W | 943.485926938844 | -2.29043239125533 | 0.770313982389434 | -2.97337506993011 | 0.00294544283823086 | 0.00856372545291502 | 1508.072041646 | 1599.32597169461 | 1594.73229787988 | 792.83598171747 | 75.421545191069 | 90.5277235040259 |
| YDR190C | 2708.91102865815 | -1.15728791632899 | 0.535316138374391 | -2.16187750259754 | 0.0306276195244102 | 0.0602633271424387 | 4165.15135311753 | 3958.74397735955 | 3100.21810936465 | 3176.57141685923 | 950.625725845765 | 902.15558940219 |
| YDR191W | 237.723783173397 | -0.0716253194914287 | 0.363705590169839 | -0.196932138046 | 0.843880643594775 | 0.883835687804864 | 272.889226583562 | 291.011375261958 | 166.78851555808 | 213.455841231627 | 219.979506807285 | 262.218233597868 |
| YDR192C | 399.762210386004 | -1.18857596409481 | 0.830039016199849 | -1.43195192141261 | NA | NA | 529.148084788071 | 517.719953723823 | 621.799290457753 | 628.170047053073 | 42.4246191699763 | 59.3112671233273 |
| YDR193W | 3.79360178214103 | 1.91167346189723 | 1.22824799934589 | 1.55642302117757 | 0.119607534458813 | 0.186371285244316 | 2.26777750623459 | 1.64878966154084 | 0.731528577009121 | 0.87124833155766 | 10.9989753403642 | 6.24329127613972 |
| YDR194C | 933.337315621281 | -1.34481887261681 | 0.5893414601801 | -2.28190100897677 | 0.0224951852684263 | 0.046913534044257 | 1283.56206852878 | 1089.02557144772 | 1647.40235542454 | 1037.65676288517 | 350.395928700175 | 191.981206741296 |
| YDR195W | 207.24565092503 | -3.09738268463497 | 0.529447994078596 | -5.85021138860933 | 4.90948657231141e-09 | 3.09971505153779e-07 | 484.54846049879 | 405.602256739046 | 225.310801718809 | 73.1848598508434 | 29.8543616381315 | 24.9731651045589 |
| YDR196C | 600.119558930608 | -0.569001318201587 | 0.336271632081515 | -1.69208837117624 | 0.0906291253589392 | 0.147698517961873 | 835.29804812974 | 797.189801354996 | 517.922232522458 | 433.881669115714 | 518.523123188599 | 497.902479272143 |
| YDR197W | 614.353403102706 | 0.397617208579173 | 0.280509384202387 | 1.4174827330992 | 0.156341839160757 | 0.232205130118837 | 518.565123092309 | 484.744160493007 | 587.417447338324 | 717.037376871954 | 725.932372464039 | 652.423938356601 |
| YDR198C | 557.691811775329 | -0.692635201802143 | 0.272239066877412 | -2.54421677882857 | 0.0109523090040544 | 0.0256950346033189 | 717.373617805541 | 701.560000985627 | 648.134319230081 | 420.81294414235 | 441.53029580605 | 416.739692682326 |
| YDR200C | 31.0138578386444 | -2.39444793318638 | 0.676881746400077 | -3.53746861386201 | 0.000403982196941372 | 0.00170711637027719 | 73.3248060349184 | 62.6540071385519 | 21.2143287332645 | 14.8112216364802 | 4.71384657444181 | 9.36493691420958 |
| YDR201W | 308.730108001986 | 0.0764290305234659 | 0.440447797157498 | 0.17352574134032 | 0.862238196122686 | 0.898337477851267 | 340.9225517706 | 331.406721969709 | 228.236916026846 | 167.279679659071 | 384.964136912748 | 399.570641672942 |
| YDR202C | 368.348407576049 | -1.86207187733806 | 0.764155579707813 | -2.43677063517622 | 0.0148190756771013 | 0.0331141038724956 | 586.598448279347 | 572.130012554671 | 576.444518683187 | 390.319252537832 | 47.1384657444181 | 37.4597476568383 |
| YDR203W | 3.21756608840646 | 1.96258577065175 | 1.24845483884596 | 1.57201182580695 | 0.115947808657206 | 0.181635584469085 | 0.75592583541153 | 2.47318449231126 | 0.731528577009121 | 4.3562416577883 | 9.42769314888362 | 1.56082281903493 |
| YDR204W | 1166.0792615509 | 1.73084829536887 | 0.370614879756994 | 4.67020724182407 | 3.0089602148611e-06 | 3.69099119689628e-05 | 439.192910374099 | 417.968179200603 | 762.252777243504 | 1536.01080853615 | 1855.68426813859 | 1985.36662581243 |
| YDR205W | 1097.57268832097 | 0.621168076988633 | 0.325058952251627 | 1.91093976242127 | 0.0560123224466991 | 0.0993717235693504 | 824.715086433979 | 903.53673452438 | 865.39830660179 | 966.214399697444 | 1511.57346820434 | 1513.99813446388 |
| YDR206W | 1815.90679204663 | -0.407215688879476 | 0.413509367228627 | -0.984779840922755 | 0.324732224833543 | 0.420362651174879 | 1957.09198788045 | 1916.71798154123 | 2339.42838927517 | 2421.19911339874 | 1077.89958335569 | 1183.10369682848 |
| YDR207C | 131.47519667672 | -4.92897979925362 | 0.837790903207484 | -5.88330546486362 | 4.02152714164512e-09 | 2.74869057973991e-07 | 377.962917705765 | 333.05551163125 | 54.133114698675 | 17.4249666311532 | 4.71384657444181 | 1.56082281903493 |
| YDR208W | 969.60307957088 | -0.897013919532686 | 0.271777378220509 | -3.3005466658262 | 0.000964966643464958 | 0.00347035448046194 | 1286.58577187042 | 1263.79727557105 | 1235.55176656841 | 747.531068476472 | 642.654416315567 | 641.498178623356 |
| YDR209C | 1.76516306295909 | 1.32919362967571 | 1.71344610136813 | 0.77574288949877 | 0.437900810534195 | 0.534916771593364 | 2.26777750623459 | 0.82439483077042 | 0 | 4.3562416577883 | 3.14256438296121 | 0 |
| YDR210C-D | 6.04958008952927 | -3.34754046637681 | 1.2942439453527 | -2.58648338931541 | 0.00969608437217617 | 0.0232302021416721 | 14.3625908728191 | 15.663501784638 | 3.6576428850456 | 2.61374499467298 | 0 | 0 |
| YDR210W | 1197.69544599539 | -0.432218806703886 | 0.266186903192679 | -1.62374182020152 | 0.104430921599141 | 0.166181155201006 | 1486.15019241907 | 1295.9486739711 | 1345.28105311977 | 1030.68677623271 | 1027.61855322831 | 1000.48742700139 |
| YDR210W-B | 3.52208375596435 | -2.92477740214868 | 1.69597461840254 | -1.72454078640845 | 0.0846102625466872 | 0.139858852874914 | 12.0948133665845 | 6.59515864616336 | 0 | 0.87124833155766 | 1.5712821914806 | 0 |
| YDR211W | 2035.45022945716 | -1.15559779963779 | 0.446163971402954 | -2.59007421868699 | 0.00959552416714692 | 0.0230149629930824 | 3106.85518354139 | 2970.29457526582 | 2354.05896081535 | 2064.85854579165 | 897.202131335425 | 819.431979993338 |
| YDR212W | 12522.6913683268 | 1.50555315726701 | 0.334498458543682 | 4.50092704110444 | 6.76577281210363e-06 | 6.84012196388499e-05 | 6341.46183326732 | 6725.41302942508 | 6502.55752103408 | 12845.6854004861 | 20596.3669659278 | 22124.6634598201 |
| YDR213W | 774.930691669764 | 0.285147068708804 | 0.36830579153641 | 0.774212828745635 | 0.438804943096048 | 0.535817943408902 | 835.29804812974 | 809.555723816552 | 450.621603437619 | 738.818585160895 | 945.911879271323 | 869.378310202456 |
| YDR214W | 3931.27947865616 | 1.09419354705178 | 0.548107194156451 | 1.99631305466765 | 0.0458998579474478 | 0.0845523699031933 | 1353.86317122205 | 1336.34402067885 | 4833.94083687627 | 6311.32291380369 | 4482.86809229416 | 5269.33783706192 |
| YDR215C | 12.1487125007981 | -0.272968008921618 | 0.879946422679885 | -0.310209805831463 | 0.75640141413248 | 0.813633724238045 | 6.80333251870377 | 4.1219741538521 | 28.5296145033557 | 5.22748998934596 | 17.2841041062866 | 10.9257597332445 |
| YDR216W | 661.99986888716 | -0.91428373909384 | 0.378775716892435 | -2.4137865716283 | 0.0157877072377477 | 0.0348672272328859 | 617.59140753122 | 792.243432370373 | 1184.34476617777 | 373.765534238236 | 535.807227294886 | 468.246845710479 |
| YDR217C | 293.455537337729 | -0.745857735997258 | 0.402590450082348 | -1.85264637013793 | 0.063933061526823 | 0.110789659781849 | 438.436984538687 | 430.334101662159 | 233.35761606591 | 163.79468633284 | 238.834893105052 | 255.974942321729 |
| YDR218C | 12.3355859768601 | -3.494001021975 | 0.876192773915198 | -3.98770810031032 | 6.67146772340107e-05 | 0.000409964237964722 | 25.701478403992 | 20.6098707692605 | 21.9458573102736 | 2.61374499467298 | 3.14256438296121 | 0 |
| YDR219C | 205.28876266842 | -3.58011528296446 | 0.574788912650045 | -6.22857401068935 | 4.70699718591421e-10 | 6.1863391586301e-08 | 463.382537107268 | 450.943972431419 | 224.5792731418 | 58.3736382143632 | 17.2841041062866 | 17.1690510093842 |
| YDR220C | 1.42465780487395 | -1.32128302070458 | 2.0086267580845 | -0.657804151710399 | 0.510663986274365 | 0.604424935049974 | 5.29148084788071 | 0.82439483077042 | 0 | 0.87124833155766 | 0 | 1.56082281903493 |
| YDR221W | 595.493871101475 | -0.543112580436382 | 0.546486919863658 | -0.993825397636017 | 0.320307879126208 | 0.415883617252576 | 648.584366783092 | 683.423314708678 | 788.587806015832 | 933.978211429811 | 263.975408168741 | 254.414119502694 |
| YDR222W | 1924.82274710214 | -0.174683778649563 | 0.328769531502816 | -0.5313259347698 | 0.59519293643587 | 0.680220498783851 | 2137.75826254381 | 1999.15746461827 | 1985.36855800275 | 1292.93252403157 | 1975.10171469112 | 2158.61795872531 |
| YDR223W | 37.9482559592236 | 0.0305508560768866 | 0.435066146881408 | 0.0702211750922883 | 0.94401762063943 | 0.960422350224001 | 35.5285142643419 | 42.0441363692914 | 35.1133716964378 | 38.334926588537 | 28.2830794466509 | 48.3855073900828 |
| YDR224C | 14634.8490549595 | -0.383471704646869 | 0.313738782258156 | -1.22226427312176 | 0.221607705145661 | 0.308840861531715 | 15714.1862665349 | 13948.7605366355 | 20043.1514814729 | 14514.9972037506 | 12788.6657564606 | 10799.3330849027 |
| YDR225W | 40906.6615597878 | 1.40526962128125 | 0.389469898385036 | 3.60815977591156 | 0.000308376567619465 | 0.00136755490981954 | 23144.1813027948 | 22083.888726678 | 22039.4929681308 | 33807.0490094319 | 71776.170506834 | 72589.1868448575 |
| YDR226W | 14031.244230523 | 0.871049299021404 | 0.267629023916174 | 3.25468921970971 | 0.00113516511858444 | 0.00395373897440984 | 9473.26256937729 | 9901.80631238351 | 10384.0481506445 | 19319.0605039595 | 16199.919394165 | 18909.3684526082 |
| YDR227W | 272.07550108127 | -2.10902285145371 | 0.412013916770128 | -5.11881459730007 | 3.0746202713161e-07 | 6.6001848490919e-06 | 486.060312169613 | 496.285688123793 | 343.818431194287 | 122.84601474963 | 124.131293126968 | 59.3112671233273 |
| YDR228C | 273.723885547073 | -2.14132483606636 | 0.520676070108862 | -4.11258546147253 | 3.91252561059795e-05 | 0.000269483047403752 | 607.008445835458 | 554.817721108492 | 176.298387059198 | 74.0561081824011 | 108.418471212162 | 121.744179884725 |
| YDR229W | 126.549046988663 | -3.44139289716769 | 0.587989102970416 | -5.852817475328 | 4.83314458557995e-09 | 3.09971505153779e-07 | 337.898848428954 | 245.669659569585 | 113.386929436414 | 35.721181593864 | 12.5702575318448 | 14.0474053713144 |
| YDR230W | 2.2279022181207 | 1.18843280456237 | 1.53981702388698 | 0.77180131543318 | 0.440232112527404 | 0.537091201872278 | 0.75592583541153 | 2.47318449231126 | 0.731528577009121 | 0 | 6.28512876592241 | 3.12164563806986 |
| YDR231C | 368.62887759679 | 0.640718489588395 | 0.307433578888839 | 2.08408753495357 | 0.0371522056834133 | 0.0708709136851841 | 305.394037506258 | 263.806345846534 | 294.806016534676 | 424.29793746858 | 388.106701295709 | 535.362226928981 |
| YDR232W | 8600.84010419247 | 1.59558894153923 | 0.36937732413366 | 4.31967215443324 | 1.56261168869837e-05 | 0.000130183949226617 | 3787.18843541176 | 4187.10134548296 | 4855.15516560954 | 8181.02183332642 | 14905.182868385 | 15689.3909769391 |
| YDR233C | 29724.7606395486 | 0.657279843836735 | 0.305488941254616 | 2.15156673474773 | 0.0314314944756298 | 0.0616191246341115 | 22756.3913492287 | 24573.5611156047 | 21874.1675097267 | 28010.6338595788 | 40432.2333511789 | 40701.5766519739 |
| YDR234W | 8446.38339948813 | 1.03142109455511 | 0.295162521420794 | 3.49441754864493 | 0.000475097333347611 | 0.00193755569763672 | 5177.33604673357 | 5578.67981982343 | 5891.73115923146 | 9359.82082592394 | 11310.0892142774 | 13360.643330939 |
| YDR235W | 724.355859906527 | -0.0511831304692558 | 0.328073086708681 | -0.156011366195072 | 0.876024061999526 | 0.908908483853222 | 799.013608029987 | 789.770247878062 | 621.799290457753 | 542.787710560422 | 812.352892995472 | 780.411409517465 |
| YDR236C | 1052.16118631228 | 1.45825935740094 | 0.39896754403934 | 3.65508267323407 | 0.000257099114303795 | 0.00117426829511804 | 511.761790573605 | 585.320329846998 | 586.685918761315 | 871.24833155766 | 1799.11810924529 | 1958.83263788884 |
| YDR237W | 914.347743282575 | 0.099734966653528 | 0.565101515153483 | 0.176490354350651 | 0.859908725855452 | 0.896693086271905 | 743.075096209534 | 708.979554462561 | 1197.51228056393 | 1821.78026128707 | 499.667736890832 | 515.071530281527 |
| YDR238C | 6333.61501209825 | 0.321764811703163 | 0.375704470230799 | 0.856430618207711 | 0.391759634813497 | 0.490365801399207 | 5089.64864982583 | 5531.68931446952 | 6270.66296212219 | 10092.5406727639 | 5991.29899611554 | 5025.84947729248 |
| YDR239C | 347.316449846827 | -1.57383465725075 | 0.419581876590163 | -3.7509595744242 | 0.00017615910293889 | 0.000874009724904816 | 670.506216010027 | 580.373960862375 | 310.168116651867 | 222.168324547203 | 150.843090382138 | 149.838990627353 |
| YDR240C | 336.489248202103 | -0.750944320666839 | 0.429973729403191 | -1.7464888417931 | 0.0807260443093407 | 0.134528083291855 | 396.861063591053 | 436.104865477552 | 435.259503320427 | 390.319252537832 | 210.551813658401 | 149.838990627353 |
| YDR241W | 35.9058447297223 | 0.243444371382226 | 0.448950699521009 | 0.542251903476172 | 0.587644983889778 | 0.673746429811317 | 36.2844400997534 | 38.7465570462097 | 23.4089144642919 | 33.9786849307487 | 43.9959013614569 | 39.0205704758732 |
| YDR242W | 665.568211537704 | -0.430735144198087 | 0.282022670312137 | -1.52730680736182 | 0.126684737536334 | 0.195413104127902 | 758.193612917764 | 679.301340554826 | 855.156906523662 | 579.380140485844 | 575.089282081901 | 546.287986662226 |
| YDR243C | 263.634764884884 | -1.33139973263199 | 0.468859159726309 | -2.83965814682853 | 0.00451619028985962 | 0.01227184196907 | 382.498472718234 | 443.524418954486 | 307.97353092084 | 245.69202949926 | 116.274882169565 | 85.8452550469212 |
| YDR244W | 247.972190015114 | -1.66774803463608 | 0.486523648232149 | -3.42788688832715 | 0.000608298852746462 | 0.00238432417022959 | 463.382537107268 | 475.675817354532 | 193.855072907417 | 165.537182995955 | 78.5641095740302 | 110.81842015148 |
| YDR245W | 3325.55625882026 | -0.34638377894793 | 0.345630790482827 | -1.00217859197108 | 0.316257345363351 | 0.412036678968234 | 4020.76951855393 | 4201.94045243683 | 2947.32863676975 | 3798.6427255914 | 2487.3397091138 | 2497.31651045589 |
| YDR246W | 200.145065536172 | -2.39614644730874 | 0.725994846926703 | -3.30050062676362 | 0.000965124980513562 | 0.00347035448046194 | 428.609948678337 | 403.953467077506 | 178.492972790226 | 142.884726375456 | 15.712821914806 | 31.2164563806986 |
| YDR246W-A | 21.0481159223372 | 0.555625702017694 | 0.498212656770542 | 1.11523803032084 | 0.264748433079149 | 0.355426289145241 | 15.1185167082306 | 20.6098707692605 | 15.3621001171915 | 23.5237049520568 | 25.1405150636897 | 26.5339879235938 |
| YDR247W | 1136.60399672255 | 0.734224318088784 | 0.319383519802302 | 2.29887978735806 | 0.0215117664698271 | 0.0451845323110523 | 926.009148379124 | 772.457956431883 | 861.009135139735 | 1075.99168947371 | 1652.9888654376 | 1531.16718547327 |
| YDR248C | 185.522312038803 | -1.64354199760552 | 0.510728008752177 | -3.21803772152825 | 0.00129070842090485 | 0.00437473123794242 | 284.984039950147 | 260.508766523453 | 299.92671657374 | 161.180941338167 | 59.708723276263 | 46.8246845710479 |
| YDR249C | 971.080884417348 | 0.390253268143505 | 0.389296010567314 | 1.00245894525042 | 0.316121985388477 | 0.411943663679035 | 757.437687082353 | 877.156099939726 | 885.881106758046 | 646.466262015783 | 1392.15602165181 | 1267.38812905636 |
| YDR250C | 15.5979947497206 | 0.462763876832729 | 0.659407749760092 | 0.701787137019687 | 0.482811921727411 | 0.577509059421346 | 12.850739201996 | 9.06834313847462 | 16.8251572712098 | 7.84123498401894 | 26.7117972551703 | 20.2906966474541 |
| YDR251W | 1377.95853731065 | -1.35764052035761 | 0.318086123933738 | -4.26815386841717 | 1.97097361654407e-05 | 0.000154416911077176 | 2047.04716229442 | 2241.52954486477 | 1658.37528407968 | 751.016061802703 | 642.654416315567 | 927.128754506748 |
| YDR252W | 119.37842216971 | 0.284174383660638 | 0.347576198074202 | 0.817588733737087 | 0.413592062796627 | 0.511652224089288 | 114.144801147141 | 117.0640659694 | 92.1726007031492 | 143.755974707014 | 144.557961616216 | 104.57512887534 |
| YDR253C | 12.0893500834644 | -1.8743376429528 | 0.704471580680385 | -2.66062917845819 | 0.00779948024857374 | 0.0193633973788801 | 21.9218492269344 | 14.8391069538676 | 19.7512715792463 | 3.48499332623064 | 7.85641095740302 | 4.68246845710479 |
| YDR254W | 163.357995589967 | -0.498805038668215 | 0.358958437414766 | -1.38958995437085 | 0.164653427955295 | 0.242122818395732 | 178.398497157121 | 185.488836923344 | 211.411758755636 | 177.734659637763 | 119.417446552526 | 107.69677451341 |
| YDR255C | 164.237378935083 | -0.669742583556088 | 0.346178838062333 | -1.93467222694732 | 0.0530305305807043 | 0.0951299768634361 | 201.832198054878 | 234.95252676957 | 167.520044135089 | 105.421048118477 | 149.271808190657 | 126.426648341829 |
| YDR256C | 136.126060273396 | 0.677622609828594 | 0.327308704707426 | 2.07028594132352 | 0.0384255753258816 | 0.0729542173050347 | 113.388875311729 | 95.6298003693687 | 104.608586512304 | 142.884726375456 | 188.553862977672 | 171.690510093842 |
| YDR257C | 465.773781232937 | -2.18769311345911 | 0.663968049626192 | -3.29487708737004 | 0.000984647375940323 | 0.00352873071845057 | 863.267304039967 | 840.882727385828 | 589.612033069352 | 367.666795917332 | 81.7066739569914 | 51.5071530281527 |
| YDR258C | 748.859292663036 | 0.092676643859568 | 0.480315378999639 | 0.19294956587188 | 0.846998466368133 | 0.886217729230021 | 557.873266533709 | 426.212127508307 | 1190.92852337085 | 1042.01300454296 | 609.657490294474 | 666.471343727915 |
| YDR259C | 23.1963689663507 | -2.64912748714897 | 0.746183659256457 | -3.55023519248428 | 0.000384887130946097 | 0.00163933407625189 | 55.1825859850417 | 52.7612691693068 | 12.4359858091551 | 7.84123498401894 | 4.71384657444181 | 6.24329127613972 |
| YDR260C | 481.348350627128 | 0.88360306484124 | 0.283736074421577 | 3.11417244579333 | 0.0018446164479056 | 0.00582320094338825 | 319.756628379077 | 324.811563323545 | 370.153459966615 | 568.925160507152 | 642.654416315567 | 661.78887527081 |
| YDR261C | 2267.12920652148 | -0.294371689603521 | 0.465689117985386 | -0.632120610584589 | 0.527308068351841 | 0.619683204413477 | 2059.89790149642 | 2162.38764111081 | 3272.1273249618 | 3350.82108317076 | 1341.87499152444 | 1415.66629686468 |
| YDR261C-D | 96.1278464673063 | -1.20857519814977 | 0.433535078312969 | -2.78772182138698 | 0.00530801011985534 | 0.0140470990255596 | 132.287021197018 | 153.337438523298 | 117.776100898468 | 71.4423631877281 | 70.7076986166272 | 31.2164563806986 |
| YDR261W-B | 18.2369497048507 | -2.90144418636293 | 0.862839668711105 | -3.36266897730497 | 0.000771928664948412 | 0.00288856513786622 | 40.8199951122226 | 42.8685312000618 | 13.8990429631733 | 8.7124833155766 | 0 | 3.12164563806986 |
| YDR262W | 1695.57194197321 | 0.868852758167569 | 0.395931762644209 | 2.19445076183073 | 0.0282030200206488 | 0.0563885280760565 | 1204.94578164598 | 1170.640659694 | 1223.11578075925 | 1226.71765083318 | 2674.32228989999 | 2673.68948900684 |
| YDR263C | 111.448522627975 | -1.41990806211478 | 0.418587210193418 | -3.39214392493902 | 0.000693480028371273 | 0.00264730965187374 | 185.201829675825 | 165.703360984854 | 137.527372477715 | 86.2535848242083 | 53.4235945103405 | 40.5813932949082 |
| YDR264C | 3568.36477222885 | 0.351192938995245 | 0.346388329059035 | 1.01387058839211 | 0.31064450725623 | 0.405791202176495 | 3273.15886733192 | 3616.62012258983 | 2517.92136206539 | 2942.20561567022 | 4176.46806495544 | 4883.8146007603 |
| YDR265W | 538.715480984082 | 0.839902708301115 | 0.364656446299337 | 2.3032712483894 | 0.021263586271446 | 0.0447215857570583 | 337.898848428954 | 361.084935877444 | 458.668417784719 | 467.860354046463 | 807.63904642103 | 799.141283345884 |
| YDR266C | 718.918182903539 | -3.09274193632325 | 0.721118895376556 | -4.28881000921252 | 1.7963294543289e-05 | 0.000144966938419525 | 1465.74019486296 | 1352.83191729426 | 1044.62280796902 | 356.340567607083 | 48.7097479358987 | 45.263861752013 |
| YDR267C | 2151.74218400902 | 1.22016158567589 | 0.391649485727463 | 3.11544283891889 | 0.00183668942465191 | 0.00580440439257618 | 1446.08612314226 | 1432.79821587899 | 997.804979040441 | 1858.37269121249 | 3560.52544589505 | 3614.8656488849 |
| YDR268W | 435.178615291838 | 0.653794827444116 | 0.368936687584386 | 1.77210575539353 | 0.0763770037515205 | 0.12869385247509 | 269.109597406505 | 266.279530338846 | 479.151217940974 | 534.946475576403 | 502.810301273793 | 558.774569214505 |
| YDR269C | 1.94031555446786 | 0.58361008354452 | 1.42781852211444 | 0.408742479877806 | 0.68272865578944 | 0.75571889709247 | 0.75592583541153 | 0.82439483077042 | 2.92611430803648 | 0.87124833155766 | 3.14256438296121 | 3.12164563806986 |
| YDR270W | 444.143509517708 | -0.0864221948615568 | 0.310346546090815 | -0.278469974775448 | 0.78065161051656 | 0.83276401718182 | 452.799575411506 | 491.33931913917 | 427.212688973327 | 345.885587628391 | 499.667736890832 | 447.956149063025 |
| YDR271C | 9.55574078936798 | 1.33875751209079 | 0.776992105253522 | 1.7230001476707 | 0.0848884983326031 | 0.14013892060035 | 6.80333251870377 | 3.29757932308168 | 5.85222861607297 | 6.96998665246128 | 10.9989753403642 | 23.412342285524 |
| YDR272W | 615.499730726372 | -1.18656979069703 | 0.365504676393448 | -3.24638743997832 | 0.00116879656709029 | 0.00404028442944793 | 888.968782443959 | 751.023690831852 | 927.578235647566 | 514.907763950577 | 270.260536934664 | 340.259374549615 |
| YDR273W | 39.2675942901322 | -0.955824244679586 | 0.45744291271202 | -2.08949405077201 | 0.0366632729283779 | 0.0701460123763379 | 47.6233276309264 | 48.6392950154547 | 59.9853433147479 | 33.9786849307487 | 17.2841041062866 | 28.0948107426287 |
| YDR274C | 3.09088096489322 | 0.44241763861022 | 1.31971178387745 | 0.335238075476109 | 0.737445502047792 | 0.799375367826942 | 3.02370334164612 | 1.64878966154084 | 2.92611430803648 | 0 | 3.14256438296121 | 7.80411409517465 |
| YDR275W | 506.21508022647 | -0.377398205767452 | 0.377240840797166 | -1.00041714722604 | 0.317108675135646 | 0.412714706256774 | 625.150665885335 | 575.427591877753 | 517.190703945449 | 625.5563020584 | 378.679008146825 | 315.286209445056 |
| YDR276C | 9187.13271158729 | 1.181646905029 | 0.434532929707806 | 2.71934949975732 | 0.00654104537485487 | 0.0166895135554934 | 6200.10370204537 | 6265.40071385519 | 4399.41286213285 | 6532.61999001933 | 15940.6578325707 | 15784.6011689002 |
| YDR277C | 124.963340207211 | -3.07404048093801 | 0.603790571574604 | -5.09123630884353 | 3.5573634110411e-07 | 7.27283186257292e-06 | 245.675896508747 | 262.157556184993 | 164.593929827052 | 52.2748998934596 | 15.712821914806 | 9.36493691420958 |
| YDR279W | 384.918522548987 | -1.99292214896543 | 0.780000084078834 | -2.55502812069442 | 0.0106179219546248 | 0.0250290693220293 | 632.70992423945 | 629.01325587783 | 585.954390184306 | 385.091762548486 | 39.2820547870151 | 37.4597476568383 |
| YDR280W | 1393.56060776475 | -0.276328649800834 | 0.31155057243195 | -0.886946371639845 | 0.375107774033654 | 0.472929231567651 | 1715.95164638417 | 1656.20921501777 | 1208.48520921907 | 1431.46100874923 | 1205.17344086562 | 1144.0831263526 |
| YDR281C | 243.566341148493 | 2.71431605690531 | 0.429898394837418 | 6.3138548305858 | 2.72169261767656e-10 | 4.17326201377072e-08 | 68.0333251870376 | 70.0735606154857 | 54.8646432756841 | 231.752056194337 | 512.237994422677 | 524.436467195736 |
| YDR282C | 248.698755041974 | -0.562018698373178 | 0.312782415448804 | -1.79683598122596 | 0.0723616623276804 | 0.122989998783389 | 314.465147531196 | 317.392009846612 | 257.498059107211 | 190.803384611127 | 230.978482147649 | 181.055447008052 |
| YDR283C | 1105.03762571344 | -0.577714664631598 | 0.327517551700827 | -1.76391970943687 | 0.0777454980239045 | 0.130565604299122 | 1372.00539127193 | 1343.76357415578 | 1256.03456672466 | 1159.63152930324 | 763.643145059573 | 735.147547765452 |
| YDR284C | 3502.19389473654 | 1.29477897988733 | 0.366718514395833 | 3.53071614619859 | 0.000414436245208471 | 0.00174328505495921 | 1923.07532528693 | 2058.51389243374 | 2102.41313032421 | 3095.54532202436 | 6014.86822898775 | 5818.74746936222 |
| YDR285W | 10.7460897853143 | -1.46651113346157 | 0.751624689146224 | -1.95112155659415 | 0.0510425874725096 | 0.0921801074938199 | 12.0948133665845 | 16.4878966154084 | 18.288214425228 | 3.48499332623064 | 10.9989753403642 | 3.12164563806986 |
| YDR286C | 209.532460002911 | 0.355458963073974 | 0.438064048396915 | 0.811431489013462 | 0.417117921982641 | 0.514506687908103 | 197.296643042409 | 193.732785231049 | 159.473229787988 | 121.103518086515 | 276.545665700586 | 309.042918168916 |
| YDR287W | 811.819753687659 | 1.30185274596624 | 0.395155902887519 | 3.29452941599308 | 0.000985866255024815 | 0.00353113386115673 | 393.837360249407 | 422.090153354455 | 588.880504492342 | 720.522370198184 | 1422.01038328995 | 1323.57775054162 |
| YDR288W | 409.05871265521 | -1.11887155714192 | 0.374216771459076 | -2.98990222372831 | 0.00279066760981195 | 0.00820634676127349 | 678.065474364142 | 628.18886104706 | 375.274160005679 | 309.293157702969 | 229.407199956168 | 234.12342285524 |
| YDR289C | 130.325227017028 | -2.63013996065957 | 0.450719186687615 | -5.83542932793422 | 5.36522345621458e-09 | 3.2291625287871e-07 | 303.882185835435 | 226.708578461865 | 141.916543939769 | 29.6224432729604 | 32.9969260210927 | 46.8246845710479 |
| YDR291W | 803.184957388783 | 0.00250345452367117 | 0.311923590727316 | 0.0080258582489187 | 0.993596360363837 | 0.995451238447902 | 768.020648778114 | 843.355911878139 | 797.366148939942 | 1007.16307128065 | 707.076986166272 | 696.126977289579 |
| YDR292C | 2395.53662450686 | -0.487962844491611 | 0.31728581611822 | -1.5379283274037 | 0.124066140165147 | 0.191985898786668 | 3033.53037750647 | 3264.60352985086 | 2092.9032588231 | 2142.39964730028 | 1899.68016950005 | 1940.10276406042 |
| YDR293C | 4438.76211428756 | -1.07720208339286 | 0.563034286010402 | -1.91320868046206 | 0.0557213427000059 | 0.0991012004937968 | 5342.88380468869 | 5884.53030203925 | 6843.44983792033 | 5600.38427525264 | 1707.98374213942 | 1253.34072368505 |
| YDR294C | 4631.64183887118 | 0.505023963423656 | 0.371967635793261 | 1.35770942100013 | 0.174555906222399 | 0.253928176207872 | 3342.70404418978 | 3528.4098756974 | 4617.40837808157 | 7543.26805462622 | 4028.76753895627 | 4729.29314167584 |
| YDR295C | 124.380201678458 | -3.6845450038913 | 0.736839892902464 | -5.00046894770806 | 5.71910389851521e-07 | 1.00670795589489e-05 | 351.505513466361 | 284.416216615795 | 57.7907575837206 | 32.2361882676334 | 6.28512876592241 | 14.0474053713144 |
| YDR296W | 513.503323285857 | -0.386106020118674 | 0.601071860978097 | -0.642362494711334 | 0.52063784561807 | 0.613187221247324 | 585.842522443935 | 586.969119508539 | 574.24993295216 | 925.265728114235 | 197.981556126556 | 210.711080569716 |
| YDR297W | 8914.79151820013 | 1.01355986901884 | 0.388141564636511 | 2.61131494630839 | 0.00901947871539094 | 0.0218942491244318 | 6692.96734673368 | 6411.31859890155 | 4613.01920661952 | 7376.8596232987 | 14317.5233287713 | 14077.061004876 |
| YDR298C | 7913.5781588215 | 0.920867465647605 | 0.39119301308691 | 2.35399773217069 | 0.0185727243960462 | 0.0399136130163837 | 5972.57002558649 | 6295.90332259369 | 4141.91480302564 | 6479.47384179431 | 11850.6102881467 | 12740.9966717821 |
| YDR299W | 310.087938298057 | -1.95539320321253 | 0.504254645680001 | -3.87778916855714 | 0.000105410002664162 | 0.000583697693170422 | 547.290304837947 | 563.886064246967 | 370.153459966615 | 219.55457955253 | 65.9938520421854 | 93.6493691420958 |
| YDR300C | 1586.7057367507 | -0.0777332520692683 | 0.303418107499214 | -0.256191869067899 | 0.797802686720869 | 0.846433163506161 | 1808.17459830438 | 1799.65391557183 | 1280.90653834297 | 1634.46187000217 | 1445.57961616216 | 1551.45788212072 |
| YDR301W | 876.782267935485 | -0.0523642042768958 | 0.292446703200266 | -0.179055546545304 | 0.85789408654646 | 0.895358210659676 | 838.321751471386 | 851.599860185843 | 989.026636116332 | 998.450587965078 | 796.640071080666 | 786.654700793605 |
| YDR302W | 1440.41231505078 | 1.10507840712887 | 0.369927762637747 | 2.98728162290167 | 0.00281470303925391 | 0.00825817201494087 | 836.809899800563 | 851.599860185843 | 1053.40115089313 | 1255.46884577459 | 2372.63610913571 | 2272.55802451486 |
| YDR303C | 927.41934446943 | -0.566761775993585 | 0.299579435788396 | -1.89185807931727 | 0.0585098924759216 | 0.103149112385693 | 1111.96690389036 | 1182.18218732478 | 1027.79765069782 | 757.114800123606 | 870.490334080254 | 614.964190699762 |
| YDR304C | 6251.72648583873 | 1.03627806258507 | 0.276214284457738 | 3.75171785419963 | 0.000175627048624317 | 0.000872714655201081 | 3809.1102846387 | 3872.18252012866 | 4613.01920661952 | 7717.51772093775 | 8462.92588331453 | 9035.60329939321 |
| YDR305C | 378.103682328323 | -0.251068908931076 | 0.351257786358796 | -0.71477108460343 | 0.474750505185652 | 0.569983828000671 | 444.484391221979 | 377.572832492852 | 411.850588856135 | 439.980407436618 | 243.548739679494 | 351.185134282859 |
| YDR306C | 666.527535561732 | 0.0841500672837371 | 0.303963006309401 | 0.276843120830571 | 0.78190056903769 | 0.83382011336359 | 659.923254314265 | 678.476945724055 | 602.048018878507 | 568.925160507152 | 818.638021761394 | 671.15381218502 |
| YDR307W | 1233.30548517142 | 0.486497881177752 | 0.32916876517222 | 1.47795882432289 | 0.139418799708511 | 0.211678829499956 | 935.836184239473 | 1106.3378628939 | 1038.77057935295 | 1042.88425287452 | 1616.84937503354 | 1659.15465663413 |
| YDR308C | 492.588061601395 | -0.76903905729602 | 0.294474383489344 | -2.61156521726396 | 0.00901287955013001 | 0.0218942491244318 | 659.167328478854 | 661.164654277876 | 542.062675563759 | 353.72682261241 | 405.390805401996 | 334.016083273475 |
| YDR309C | 1056.49213216118 | -1.44299348189715 | 0.400443468213596 | -3.60348862308689 | 0.00031397449633908 | 0.00138587783168175 | 1583.66462518715 | 1594.37960270999 | 1458.66798255619 | 866.020841568314 | 411.675934167918 | 424.543806777501 |
| YDR310C | 571.421818954704 | -1.9524392674992 | 0.803513979148449 | -2.42987591773869 | 0.0151039927367849 | 0.0336224380314188 | 1214.77281750633 | 1066.76691101692 | 444.769374821546 | 578.508892154286 | 61.2800054677435 | 62.4329127613972 |
| YDR311W | 448.823024828025 | -2.96803491739879 | 0.450531514527464 | -6.58785195195897 | 4.46235263741384e-11 | 1.30625231749751e-08 | 1028.05913615968 | 962.89316233985 | 397.220017315953 | 110.648538107823 | 86.4205205314332 | 107.69677451341 |
| YDR312W | 88.9552853508934 | -2.9167560417821 | 0.564910082856515 | -5.16322177687691 | 2.42735262878951e-07 | 5.58291104621586e-06 | 223.754047281813 | 182.191257600263 | 65.8375719308209 | 24.3949532836145 | 14.1415397233254 | 23.412342285524 |
| YDR313C | 378.439789995839 | 0.454766949274756 | 0.354432696487721 | 1.28308407712185 | 0.199462621635804 | 0.283250117604097 | 258.526635710743 | 274.52347864655 | 424.28657466529 | 383.34926588537 | 424.246191699763 | 505.706593367317 |
| YDR314C | 125.175644334477 | -2.75725628201808 | 0.524305646127329 | -5.25887200029976 | 1.44941730665218e-07 | 3.74059318155718e-06 | 253.235154862862 | 253.089213046519 | 149.96335828687 | 54.0173965565749 | 25.1405150636897 | 15.6082281903493 |
| YDR315C | 144.626445964522 | -1.40197173482437 | 0.372877175179341 | -3.75987544464224 | 0.000169997975109783 | 0.000850650318342661 | 214.682937256874 | 201.152338707982 | 213.606344486663 | 78.4123498401894 | 105.2759068292 | 54.6287986662226 |
| YDR316W | 89.8173035380371 | -1.27954821033015 | 0.410607391636476 | -3.11623277221218 | 0.00183177617895495 | 0.00579402681359033 | 152.697018753129 | 134.376357415578 | 93.6356578571675 | 39.2061749200947 | 54.9948767018211 | 63.9937355804321 |
| YDR317W | 84.3420373486054 | 0.191201490069864 | 0.420250720359002 | 0.454970047181665 | 0.649130787770621 | 0.72829307896216 | 91.4670260847951 | 83.2638779078124 | 60.716871891757 | 59.2448865459209 | 97.4194958717974 | 113.94006578955 |
| YDR318W | 247.668266288312 | -0.614609667412977 | 0.365990777409535 | -1.67930370203084 | 0.093092869878481 | 0.151202542753447 | 322.780331720723 | 316.567615015841 | 258.22958768422 | 134.17224305988 | 243.548739679494 | 210.711080569716 |
| YDR319C | 1373.67629304487 | 0.726241752328409 | 0.39525221285926 | 1.83741350130532 | 0.0661488751836233 | 0.113842532384429 | 1065.09950209485 | 1185.47976664786 | 853.693849369644 | 1014.13305793312 | 2103.94685439253 | 2019.7047278312 |
| YDR320C | 424.885775154175 | -1.38004825524244 | 0.371481452115787 | -3.71498562682557 | 0.000203215245095857 | 0.000979570492827334 | 596.425484139697 | 572.130012554671 | 675.200876579419 | 332.816862655026 | 190.125145169153 | 182.616269827087 |
| YDR320C-A | 579.302835841451 | -0.600039010927553 | 0.309096185227459 | -1.94126954522584 | 0.052225591505745 | 0.0938690508783136 | 793.722127182106 | 631.486440370141 | 667.885590809327 | 378.993024227582 | 487.097479358987 | 516.632353100562 |
| YDR320W-B | 33.6730701907725 | 1.58704124847018 | 0.614438768151011 | 2.58291196899237 | 0.00979703173949204 | 0.023376392887117 | 18.8981458852882 | 16.4878966154084 | 14.6305715401824 | 17.4249666311532 | 54.9948767018211 | 79.6019637707814 |
| YDR321W | 12714.4500539185 | 1.6747191301851 | 0.401274354245401 | 4.17350152698998 | 2.99953649623336e-05 | 0.0002164667663362 | 6547.82958633467 | 6792.18901071749 | 4855.15516560954 | 11224.2922554573 | 22805.5897271495 | 24061.6445782425 |
| YDR322C-A | 2191.58753601428 | 0.276256633335383 | 0.343604172537993 | 0.803996736404116 | 0.421398859828334 | 0.518793473006017 | 2164.21566678321 | 1952.99135409512 | 1828.8214425228 | 1642.30310498619 | 2811.0238405588 | 2750.16980713955 |
| YDR322W | 711.935361274824 | -0.25508029921525 | 0.44382315971204 | -0.574734088641859 | 0.565471149229787 | 0.654499317224988 | 681.8451035412 | 728.765030401051 | 915.14224983841 | 1051.5967361901 | 455.671835529375 | 438.591212148815 |
| YDR323C | 181.559592776043 | -1.46084716179793 | 0.390399426174612 | -3.74192958251055 | 0.00018261269056724 | 0.000901169139657493 | 287.251817456381 | 280.294242461943 | 233.35761606591 | 135.043491391437 | 67.565134233666 | 85.8452550469212 |
| YDR324C | 1818.6989438387 | -2.04038016035783 | 0.838165010352642 | -2.43434184815157 | 0.0149188978169942 | 0.0333255990084781 | 3294.32479072345 | 3337.15027495866 | 2148.49943067579 | 1839.20522791822 | 171.269758871386 | 121.744179884725 |
| YDR325W | 358.734145886691 | -1.74111820154881 | 0.423812577325102 | -4.10822683115706 | 3.98708419699772e-05 | 0.000273448586034775 | 631.953998404039 | 532.559060677691 | 494.513318058166 | 255.275761146394 | 128.84513970141 | 109.257597332445 |
| YDR326C | 953.597017985971 | -2.68149821120452 | 0.447902873271347 | -5.98678501796531 | 2.14029374549593e-09 | 1.74474578746757e-07 | 1964.65124623457 | 1969.47925071053 | 1017.55625061969 | 362.439305927986 | 246.691304062455 | 160.764750360598 |
| YDR327W | 1.97080793949504 | 2.03714925792499 | 1.71504834777496 | 1.18780864724187 | 0.234908806805854 | 0.323457925129292 | 2.26777750623459 | 0 | 0 | 1.74249666311532 | 1.5712821914806 | 6.24329127613972 |
| YDR328C | 2008.62596790991 | -0.377327489437711 | 0.584882532641584 | -0.645133797608104 | 0.518840483335504 | 0.611293946703374 | 2224.68973361613 | 2159.9144566185 | 2426.48028993925 | 3566.89066939706 | 790.354942314744 | 883.42571557377 |
| YDR329C | 698.523123894206 | -1.41430731223684 | 0.433074230722063 | -3.26573878542432 | 0.00109178911548167 | 0.00382333980625445 | 1031.08283950133 | 1083.25480763233 | 935.625049994666 | 613.358825416592 | 273.403101317625 | 254.414119502694 |
| YDR330W | 227.467941100602 | -2.07890482535467 | 0.620290634216417 | -3.35150123293551 | 0.000803746810737776 | 0.00297727936121843 | 364.356252668357 | 354.48977723128 | 386.978617237825 | 183.833397958666 | 34.5682082125733 | 40.5813932949082 |
| YDR331W | 1693.24056440574 | 0.924098801257109 | 0.315862256766335 | 2.92563857017184 | 0.00343750047474216 | 0.00975650200852336 | 1121.0380139153 | 1224.22632369407 | 1160.20432313647 | 1657.11432662267 | 2444.91508994382 | 2551.94530912211 |
| YDR332W | 259.011267338712 | -2.01233048978544 | 0.586654193755562 | -3.4301817172791 | 0.000603177152455089 | 0.00236568870999438 | 476.989202144675 | 437.753655139093 | 332.84550253915 | 204.74335791605 | 42.4246191699763 | 59.3112671233273 |
| YDR333C | 1211.75669460967 | -0.185204892202028 | 0.271873453554563 | -0.681217271420209 | 0.495734021008362 | 0.589897837267895 | 1341.01243202005 | 1364.37344492504 | 1163.1304374445 | 1161.37402596636 | 1068.47189020681 | 1172.17793709523 |
| YDR334W | 387.452100388669 | -0.844785175732445 | 0.38291507175459 | -2.20619463177952 | 0.0273703749929614 | 0.0549455158836257 | 476.233276309264 | 563.886064246967 | 455.010774899673 | 397.289239190293 | 226.264635573207 | 206.028612112611 |
| YDR335W | 1651.69421662151 | -0.242259804296903 | 0.290279733955048 | -0.834573605935642 | 0.40395782821321 | 0.502217840481289 | 1647.16239536172 | 1647.96526671007 | 2075.34657297488 | 1613.55191004479 | 1580.70988462949 | 1345.42927000811 |
| YDR336W | 457.42168927647 | -0.352316741273143 | 0.341189538393421 | -1.03261296618917 | 0.30178505783726 | 0.396793746931799 | 589.622151620993 | 572.954407385442 | 376.737217159697 | 423.426689137023 | 441.53029580605 | 340.259374549615 |
| YDR337W | 307.705489360399 | -1.56628670767759 | 0.624072002027272 | -2.5097852532874 | 0.0120804601884263 | 0.0278447257027435 | 495.131422194552 | 387.465570462097 | 499.63401809723 | 329.331869328795 | 65.9938520421854 | 68.6762040375369 |
| YDR338C | 983.991552480277 | 0.476540957598501 | 0.269383509701145 | 1.76900567568956 | 0.076892927423705 | 0.129393899296749 | 827.738789775625 | 849.126675693532 | 791.513920323869 | 1047.24049453231 | 1213.02985182303 | 1175.2995827333 |
| YDR339C | 1377.71673046551 | -1.52082478340011 | 0.397933991319914 | -3.82180164694063 | 0.000132480232893608 | 0.000697606459390709 | 2412.15934079819 | 2297.58839335716 | 1422.09155370573 | 987.995607986386 | 592.373386188188 | 554.0921007574 |
| YDR340W | 0.946596509543 | 0.755783703325759 | 2.67722475407735 | 0.282301178552424 | 0.77771258195962 | 0.830729644687337 | 0 | 0 | 2.19458573102736 | 3.48499332623064 | 0 | 0 |
| YDR341C | 13831.0848674636 | 0.592635401414577 | 0.264426997875741 | 2.2412061029149 | 0.0250127277251654 | 0.0510560908241095 | 10211.8021105744 | 11107.8959498006 | 11768.8317469227 | 16025.7418106716 | 16067.9316900807 | 17804.3058967314 |
| YDR342C | 4607.27147259039 | 1.67393407552676 | 0.449529394689632 | 3.72374775776897 | 0.000196287046627718 | 0.000952591243619068 | 2301.0382429927 | 2309.95431581872 | 1984.63702942575 | 3247.1425317154 | 9564.39469954243 | 8236.46201604733 |
| YDR343C | 986.494840512926 | 3.10167887843291 | 0.501511110829326 | 6.18466632434885 | 6.22339661479946e-10 | 7.45161815811373e-08 | 178.398497157121 | 174.771704123329 | 264.081816300293 | 712.681135214166 | 2377.34995571015 | 2211.6859345725 |
| YDR344C | 17.2360255564114 | 0.359534666604624 | 0.639100826826587 | 0.562563294417673 | 0.57373229234509 | 0.66175089185995 | 8.31518418952682 | 11.5415276307859 | 25.6035001953192 | 23.5237049520568 | 14.1415397233254 | 20.2906966474541 |
| YDR345C | 53351.9007330185 | 0.958609809096517 | 0.533856973967357 | 1.79563039510866 | 0.0725533185160246 | 0.123185702937832 | 47229.490270677 | 46945.1636382215 | 14579.3645397918 | 39517.2105744608 | 86261.8210300937 | 85578.3543448662 |
| YDR346C | 3693.15897413066 | -0.811483004580769 | 0.40876809664904 | -1.98519163122824 | 0.0471231550543303 | 0.086434952591822 | 5203.03752513756 | 5533.33810413106 | 3381.12508293616 | 3866.60009545289 | 2292.5007173702 | 1882.35231975613 |
| YDR347W | 1740.55098409849 | 0.660772092467336 | 0.371084596941986 | 1.7806508217064 | 0.0749695117893923 | 0.126653634817336 | 1058.29616957614 | 1144.26002510934 | 1844.18354263999 | 2537.94638982746 | 1681.27194488425 | 2177.34783255373 |
| YDR348C | 520.970974563772 | -2.85614742410732 | 0.904914536242727 | -3.15626206643365 | 0.00159805208270962 | 0.00518984135786684 | 1022.01172947639 | 972.785900309095 | 753.474434319395 | 341.529345970603 | 18.8553862977672 | 17.1690510093842 |
| YDR349C | 4575.78992591615 | 0.140522691240979 | 0.258358748541849 | 0.543905294610982 | 0.586506645328808 | 0.672677846789752 | 4314.82466852901 | 4622.38181612974 | 4122.1635314464 | 4765.7283736204 | 4828.5501744199 | 4801.09099135145 |
| YDR350C | 1454.95080825344 | 0.104560846008535 | 0.32187813057752 | 0.324846070843425 | 0.745297574298205 | 0.80609575254449 | 1183.77985825446 | 1345.41236381732 | 1676.66349850491 | 1226.71765083318 | 1742.55195035199 | 1554.57952775879 |
| YDR351W | 609.080961368409 | -2.10491474379947 | 0.355838177079982 | -5.91537074822171 | 3.31128852815825e-09 | 2.36941090237101e-07 | 1179.24430324199 | 1117.05499569392 | 669.348647963346 | 245.69202949926 | 215.265660232843 | 227.8801315791 |
| YDR352W | 3099.46062161853 | 0.917842565794635 | 0.363574729223125 | 2.52449494428794 | 0.0115864671511631 | 0.0269277692001049 | 2312.37713052387 | 2478.95525612665 | 1644.4762411165 | 2835.04207088862 | 4454.58501284751 | 4871.32801820802 |
| YDR353W | 12352.4879121927 | 0.928613250858032 | 0.306841171222828 | 3.02636457538376 | 0.00247513667672435 | 0.00743117957953603 | 7435.2865171078 | 7728.70153847268 | 10362.8338219112 | 17942.4881400984 | 14132.1120301765 | 16513.5054253896 |
| YDR354W | 5160.88911845277 | 0.649390553321846 | 0.389602707249749 | 1.66680195295862 | 0.0955537917934484 | 0.154111299561685 | 4124.3313580053 | 4565.49857280658 | 3365.03145424196 | 3762.05029566597 | 7364.59963146959 | 7783.8233985272 |
| YDR356W | 159.531074010345 | 0.841178918033317 | 0.347792275493194 | 2.41862449889224 | 0.0155793132566483 | 0.0344779303686649 | 111.877023640906 | 115.415276307859 | 114.849986590432 | 151.597209691033 | 216.836942424323 | 246.610005407519 |
| YDR357C | 235.851617745209 | -0.990178314556889 | 0.296829310385614 | -3.33585087426353 | 0.00085038766587173 | 0.00311695877530674 | 327.315886733192 | 314.9188253543 | 298.463659419721 | 145.498471370129 | 171.269758871386 | 157.643104722528 |
| YDR358W | 201.661508787579 | -2.03950784723746 | 0.516275559943098 | -3.95042493869407 | 7.80125616490882e-05 | 0.000463897411837607 | 371.915511022473 | 325.635958154316 | 277.980859263466 | 142.013478043899 | 50.2810301273793 | 42.1422161139431 |
| YDR359C | 238.690947914689 | -1.97438709102476 | 0.426542725990184 | -4.6288143501718 | 3.67765278252012e-06 | 4.34570347145497e-05 | 478.501053815498 | 430.334101662159 | 234.089144642919 | 126.331008075861 | 84.8492383399526 | 78.0411409517465 |
| YDR360W | 3.43960532882469 | 0.917598543445115 | 1.24740407671066 | 0.73560649718636 | 0.461970207425495 | 0.558567027351682 | 3.77962917705765 | 3.29757932308168 | 0 | 2.61374499467298 | 3.14256438296121 | 7.80411409517465 |
| YDR361C | 1803.30684340951 | -0.480567319797319 | 0.398196770524275 | -1.2068589083849 | 0.227486484957046 | 0.315395686355947 | 2345.63786728198 | 2251.42228283402 | 1707.38769873929 | 2228.65323212449 | 1192.60318333378 | 1094.13679614349 |
| YDR362C | 601.679824581051 | 0.240824672416002 | 0.277208094493651 | 0.868750506206872 | 0.384983609745721 | 0.483199073623551 | 553.33771152124 | 544.100588308477 | 556.693247103941 | 585.478878806747 | 713.362114932194 | 657.106406813706 |
| YDR363W | 207.391528572515 | -2.67957991935418 | 0.515136444598588 | -5.20168966387575 | 1.97484793277588e-07 | 4.74553010711815e-06 | 461.870685436444 | 472.37823803145 | 141.916543939769 | 52.2748998934596 | 61.2800054677435 | 54.6287986662226 |
| YDR363W-A | 334.302753297214 | -2.2885971652606 | 0.430434537252781 | -5.3169459399504 | 1.05523402738867e-07 | 2.92918411050994e-06 | 628.174369226981 | 511.12479507766 | 528.163632600585 | 178.60590796932 | 81.7066739569914 | 78.0411409517465 |
| YDR364C | 592.566368475305 | -0.288700222880932 | 0.287296365984637 | -1.00488644153741 | 0.314951533851906 | 0.410667721807304 | 666.726586832969 | 671.057392247121 | 618.141647572707 | 609.873832090362 | 474.527221827142 | 515.071530281527 |
| YDR365C | 88.8924405056644 | -2.38899056933792 | 0.62218178102181 | -3.83969868968917 | 0.000123185401494206 | 0.000657098263771053 | 145.893686234425 | 257.211187200371 | 44.6232431975564 | 26.1374499467298 | 26.7117972551703 | 32.7772791997335 |
| YDR366C | 5.17764177289449 | 0.126069643602922 | 0.888700116296216 | 0.141858475419509 | 0.887191794344587 | 0.917833759932392 | 4.53555501246918 | 7.41955347693378 | 2.92611430803648 | 5.22748998934596 | 4.71384657444181 | 6.24329127613972 |
| YDR367W | 3166.90307160113 | 0.982474963878015 | 0.369760696469747 | 2.65705623463526 | 0.007882629295372 | 0.0195171598086104 | 2268.53343207 | 2491.32117858821 | 1624.72496953726 | 2885.57447411897 | 4619.56964295298 | 5111.6947323394 |
| YDR368W | 2746.62067361225 | 1.79548594864258 | 0.411655645884311 | 4.36162109421712 | 1.29102309575258e-05 | 0.000112353901846576 | 900.307669975132 | 928.268579447492 | 1856.61952844915 | 3365.63230480724 | 4449.87116627307 | 4979.02479272143 |
| YDR369C | 278.030688921173 | -1.1219510923847 | 0.396970744239724 | -2.82628155516461 | 0.00470918514015951 | 0.012726459212181 | 452.043649576095 | 400.655887754424 | 288.953787918603 | 117.618524760284 | 230.978482147649 | 177.933801369982 |
| YDR370C | 453.648043360309 | -0.642266713152528 | 0.304620732614088 | -2.10841431455091 | 0.0349951673177656 | 0.0674555155721072 | 585.842522443935 | 593.564278154702 | 480.614275094993 | 410.357964163658 | 331.540542402407 | 319.968677902161 |
| YDR371C-A | 3.53490380499808 | 1.8338596878652 | 1.1664087836933 | 1.57222726157677 | 0.115897855209113 | 0.181601505485812 | 0.75592583541153 | 0.82439483077042 | 2.92611430803648 | 2.61374499467298 | 6.28512876592241 | 7.80411409517465 |
| YDR371W | 301.321692473086 | -1.29045476963015 | 0.387979243608735 | -3.3260922868635 | 0.000880727630391753 | 0.00320445533317677 | 431.633652019983 | 460.836710400665 | 392.830845853898 | 251.790767820164 | 122.560010935487 | 148.278167808318 |
| YDR372C | 2681.26702393528 | -0.355665099141966 | 0.284338386689866 | -1.25085150577962 | 0.210988659348762 | 0.29673696262672 | 3103.07555436433 | 3153.31022769685 | 2774.6878925956 | 2605.90375968896 | 2452.77150090122 | 1997.85320836471 |
| YDR373W | 1607.40030256096 | 0.959263832854318 | 0.408410142470863 | 2.34877573571218 | 0.0188352474960284 | 0.0403791590793684 | 1147.4954181547 | 1129.42091815547 | 997.804979040441 | 1147.43405266144 | 2539.19202143266 | 2683.05442592104 |
| YDR374C | 20.9560369619653 | -0.0960605035108926 | 0.620588383264464 | -0.154789400029675 | 0.876987348040253 | 0.909614836749755 | 18.8981458852882 | 20.6098707692605 | 24.8719716183101 | 11.3262283102496 | 12.5702575318448 | 37.4597476568383 |
| YDR374W-A | 319.977863050741 | 1.04096277244071 | 0.412017914192903 | 2.52649881614939 | 0.0115205776693839 | 0.0268133430396937 | 238.116638154632 | 233.303737108029 | 155.815586902943 | 260.50325113574 | 532.664662911925 | 499.463302091178 |
| YDR375C | 735.953800845804 | 0.3950810078257 | 0.483683606810154 | 0.816817031346628 | 0.414032997735916 | 0.511976287522907 | 485.304386334202 | 529.261481354609 | 893.927921105146 | 1370.4736255402 | 540.521073869328 | 596.234316871343 |
| YDR376W | 1226.96693763728 | 1.24434720023619 | 0.353249653242054 | 3.52257161136839 | 0.00042738156274716 | 0.00178290412271507 | 582.062893266878 | 646.325547324009 | 956.107850150921 | 1372.21612220331 | 1846.25657498971 | 1958.83263788884 |
| YDR377W | 3369.39265249388 | 0.79924171025107 | 0.419037200445811 | 1.90732877510818 | 0.0564780262254347 | 0.100087641412163 | 2719.06522997527 | 2690.00033280388 | 1967.81187215454 | 2327.10429359051 | 4958.96659631279 | 5553.40759012628 |
| YDR378C | 1392.36711993521 | 0.940759177444654 | 0.454775185598602 | 2.06862469025519 | 0.0385813191689329 | 0.0731636323462684 | 1186.8035615961 | 1093.97194043235 | 580.102161568233 | 1038.52801121673 | 2141.65762698806 | 2313.13941780977 |
| YDR379C-A | 159.899340441971 | 0.608906953127324 | 0.506768307429672 | 1.20154900020425 | 0.22953831097613 | 0.317624994131129 | 86.1755452369144 | 76.668719261649 | 216.5324587947 | 138.528484717668 | 201.124120509517 | 240.366714131379 |
| YDR379W | 191.561621656023 | -2.19982489551995 | 0.490869029140878 | -4.48149050953592 | 7.41235297207356e-06 | 7.2994486040447e-05 | 398.372915261876 | 352.84098756974 | 194.586601484426 | 108.03479311315 | 48.7097479358987 | 46.8246845710479 |
| YDR380W | 2788.90560590779 | 0.533538860239962 | 0.572508950027658 | 0.931931038308111 | 0.35137216583599 | 0.447820452797106 | 1145.22764064847 | 1293.47548947879 | 4398.68133355584 | 4652.4660905179 | 2696.32024058072 | 2547.26284066501 |
| YDR381C-A | 196.078386741198 | 0.305594297504421 | 0.420102672500181 | 0.727427644498714 | 0.466964024797437 | 0.562838914410535 | 179.910348827944 | 129.429988430956 | 215.800930217691 | 135.914739722995 | 285.97335884947 | 229.440954398135 |
| YDR381W | 4888.89153238436 | -0.958934696992122 | 0.558382248710225 | -1.71734452376864 | 0.0859162414570932 | 0.141436757408916 | 7055.0558218958 | 6962.01434585619 | 5354.05765512976 | 6502.12629841481 | 1838.40016403231 | 1621.69490897729 |
| YDR382W | 84122.851525516 | 0.735450472233879 | 0.465542910429726 | 1.57976946003755 | 0.114159671956442 | 0.179139446247438 | 77991.1362169137 | 74817.9528665694 | 36591.0594219962 | 58391.9344293259 | 123223.092020292 | 133721.934197999 |
| YDR383C | 61.6220941755017 | -1.53990251514447 | 0.458025940940939 | -3.36204214106518 | 0.000773683152268349 | 0.00289344918734505 | 115.656652817964 | 93.9810107078278 | 66.56910050783 | 41.8199199147677 | 29.8543616381315 | 21.851519466489 |
| YDR384C | 2952.62968772566 | 0.831265959312693 | 0.406913080232767 | 2.04285878162772 | 0.0410664196303862 | 0.077352366896662 | 2350.17342229445 | 2310.77871064949 | 1712.50839877835 | 2138.91465397405 | 4574.00245940004 | 4629.4004812576 |
| YDR385W | 43056.5465898902 | 1.61833147988151 | 0.417242055228129 | 3.87863941231111 | 0.000105042322152686 | 0.000583143914267854 | 21936.2118178072 | 23165.4947446488 | 18368.682568699 | 34146.8358587394 | 80342.8010147862 | 80379.2535346608 |
| YDR386W | 142.875699116934 | -1.77054978098599 | 0.42082338921431 | -4.20734642219307 | 2.58386887997812e-05 | 0.000193040784072611 | 247.18774817957 | 216.81584049262 | 200.438830100499 | 87.9960814873236 | 36.1394904040539 | 68.6762040375369 |
| YDR387C | 2070.55165867601 | 1.351267890044 | 0.385009998017953 | 3.50969558453127 | 0.000448619984321887 | 0.00185556371164608 | 1147.4954181547 | 1145.08441994011 | 1204.82756633402 | 1744.23915977843 | 3799.3603390001 | 3382.30304884869 |
| YDR388W | 3953.68941937528 | 0.632478542238757 | 0.335249273329289 | 1.88659183644978 | 0.0592152456294155 | 0.1041076117536 | 2549.73784284309 | 2610.03403421915 | 4141.91480302564 | 4601.062438956 | 5196.23020722636 | 4623.15718998146 |
| YDR389W | 438.732056330245 | -1.61199302481322 | 0.495230049036943 | -3.25503880054939 | 0.00113376875185521 | 0.00395315146829864 | 610.032149177104 | 653.745100800943 | 722.018705508002 | 385.091762548486 | 127.273857509929 | 134.230762437004 |
| YDR390C | 1004.94113104197 | -1.20239520647118 | 0.497623302146545 | -2.41627592856792 | 0.0156801738240308 | 0.0346654031674419 | 1394.68316633427 | 1325.62688787883 | 1485.00301132852 | 1096.03040109954 | 378.679008146825 | 349.624311463824 |
| YDR391C | 1530.72481627544 | 0.47240529605239 | 0.32492144617942 | 1.45390617211377 | 0.145972321393177 | 0.219587816362879 | 1409.8016830425 | 1294.29988430956 | 1141.91610871124 | 1321.68371897297 | 1973.53043249964 | 2043.11707011672 |
| YDR392W | 344.159382208075 | -1.97211700064723 | 0.478025444500966 | -4.12554817600978 | 3.69852747720841e-05 | 0.000255837990904642 | 608.520297506281 | 602.632621293177 | 436.722560474445 | 236.979546183683 | 91.134367105875 | 88.966900684991 |
| YDR393W | 205.482799265154 | -3.1706435079733 | 0.554133552116514 | -5.72180387898011 | 1.05398934608903e-08 | 5.10352736001004e-07 | 499.666977207021 | 478.973396677614 | 131.675143861642 | 50.5324032303443 | 37.7107725955345 | 34.3381020187685 |
| YDR394W | 2780.20746835374 | -0.131697814217009 | 0.487609169481115 | -0.270088879495753 | 0.787091877177703 | 0.83810709143922 | 2577.70709875332 | 2565.51671335755 | 3579.36932730563 | 4628.07113723429 | 1608.99296407614 | 1721.58756939553 |
| YDR395W | 6207.65679195916 | 0.48791102738878 | 0.25766077127921 | 1.89361781759189 | 0.0582757567718534 | 0.102792624927619 | 4986.08681037445 | 5244.79991336141 | 5272.85798308174 | 7458.75696646512 | 6852.36163704691 | 7431.0774414253 |
| YDR396W | 48.0281890272006 | 1.1253838124178 | 0.47169364199486 | 2.38583629759857 | 0.0170403344192369 | 0.0371369724737345 | 34.0166625935188 | 22.2586604308013 | 33.6503145424196 | 40.0774232516523 | 78.5641095740302 | 79.6019637707814 |
| YDR397C | 856.875307828568 | -1.25592357893967 | 0.524627161618593 | -2.39393548565969 | 0.0166686804118997 | 0.036499932625853 | 1382.58835296769 | 1270.39243421722 | 972.933007422131 | 925.265728114235 | 246.691304062455 | 343.381020187685 |
| YDR398W | 1332.01331237179 | -2.38429844863646 | 0.862673312497452 | -2.76384862507671 | 0.0057124033205144 | 0.0149605032062272 | 2553.51747202015 | 2641.36103778842 | 1514.26415440888 | 1124.78159604094 | 76.9928273825496 | 81.1627865898164 |
| YDR399W | 5721.7518473185 | 0.152785067581223 | 0.265724409841424 | 0.574975658699928 | 0.565307759372208 | 0.65442782138361 | 5579.4885911725 | 5822.70068973147 | 4855.15516560954 | 6100.48081756673 | 6038.43746185996 | 5934.2483579708 |
| YDR400W | 2332.92628613697 | 1.55775199518147 | 0.371617401116364 | 4.19181661165993 | 2.76729652091165e-05 | 0.000203208547259647 | 1083.24172214472 | 1181.35779249401 | 1283.83265265101 | 2155.46837227365 | 4137.18601016843 | 4156.47116709002 |
| YDR401W | 104.78235324925 | 1.26388650732389 | 0.482910299470134 | 2.61722831074564 | 0.0088647026548814 | 0.0215754667790764 | 58.2062893266878 | 59.3564278154702 | 66.56910050783 | 67.0861215299398 | 197.981556126556 | 179.494624189017 |
| YDR402C | 27.3863866086272 | -0.454720275248372 | 0.47092308029246 | -0.965593521060752 | 0.334247618584234 | 0.430166799297055 | 27.9692559102266 | 32.1513984000464 | 35.1133716964378 | 25.2662016151721 | 17.2841041062866 | 26.5339879235938 |
| YDR403W | 181.918116736478 | 0.758676195387529 | 0.436819457814294 | 1.73681868290324 | 0.0824191802514883 | 0.136909421216708 | 158.744425436421 | 159.108202338691 | 87.0519006640854 | 148.112216364802 | 235.692328722091 | 302.799626892776 |
| YDR404C | 4295.85823598989 | 0.749647897332345 | 0.391174379772037 | 1.916403364068 | 0.0553137729580841 | 0.0984306984940762 | 3318.51441745661 | 3727.08902991307 | 2566.202248148 | 3270.66623666745 | 6373.12056864533 | 6519.5569151089 |
| YDR405W | 219.571190214079 | -1.33054778956299 | 0.602582940547239 | -2.2080741090258 | 0.0272391075082646 | 0.0547161111519726 | 384.010324389057 | 402.304677415965 | 158.01017263397 | 235.237049520568 | 76.9928273825496 | 60.8720899423623 |
| YDR406W | 826.252699089462 | 1.32481737911063 | 0.398929503594695 | 3.3209310596807 | 0.000897176956864764 | 0.00325694453337603 | 378.718843541176 | 401.480282585194 | 633.503747689899 | 779.767256744105 | 1381.15704631145 | 1382.88901766495 |
| YDR406W-A | 26.1515655130557 | 1.50633889472742 | 0.574948230144927 | 2.61995570339911 | 0.00879411891725213 | 0.0214523203890544 | 10.5829616957614 | 11.5415276307859 | 18.288214425228 | 20.9099599573838 | 56.5661588933017 | 39.0205704758732 |
| YDR407C | 746.868158826377 | -1.23081481649642 | 0.320336871081071 | -3.84225147839733 | 0.000121910831024623 | 0.000652575338723298 | 1034.86246867838 | 1074.18646449386 | 1035.11293646791 | 569.796408838709 | 367.680032806461 | 399.570641672942 |
| YDR408C | 1390.63367532805 | 0.780727269731775 | 0.389653044785658 | 2.00364729643327 | 0.04510785561813 | 0.0832926182906985 | 890.480634114782 | 885.400048247431 | 1294.80558130614 | 2463.0190333135 | 1264.88216414189 | 1545.21459084458 |
| YDR409W | 225.217501083798 | -1.2177912618528 | 0.458914895611279 | -2.6536320208798 | 0.00796306133092605 | 0.0197087298121306 | 311.44144418955 | 258.035582031141 | 377.468745736706 | 219.55457955253 | 94.2769314888362 | 90.5277235040259 |
| YDR410C | 1988.58440155561 | 1.41183065003574 | 0.438948467100157 | 3.21639271088649 | 0.00129813061683073 | 0.00439234333259726 | 1133.88875311729 | 1192.07492529403 | 932.698935686629 | 1410.55104879185 | 3719.22494723459 | 3543.06779920929 |
| YDR411C | 2530.79711103078 | 1.34141351146559 | 0.354190195391311 | 3.78726889936519 | 0.000152312238383276 | 0.00077786741886463 | 1320.60243446394 | 1372.61739323275 | 1602.77911222698 | 2409.00163675693 | 4195.32345125321 | 4284.45863825088 |
| YDR412W | 408.720692683 | -3.932653135142 | 0.725249902562323 | -5.42248005997362 | 5.8777772936629e-08 | 1.86408548284653e-06 | 1025.03543281803 | 1042.03506609381 | 236.283730373946 | 101.936054792246 | 31.4256438296121 | 15.6082281903493 |
| YDR413C | 7.06528658431541 | 0.450748349003041 | 0.844084731080315 | 0.534008414565376 | 0.593335712367307 | 0.678941362410351 | 7.5592583541153 | 8.2439483077042 | 2.19458573102736 | 8.7124833155766 | 10.9989753403642 | 4.68246845710479 |
| YDR414C | 1287.32421620471 | 0.978446471829186 | 0.361751986199626 | 2.70474388297967 | 0.0068357070843004 | 0.0173042270530246 | 766.508797107291 | 861.492598155088 | 971.469950268113 | 1116.94036105692 | 2011.24120509517 | 1996.29238554568 |
| YDR415C | 1034.96825601926 | 0.630295316737442 | 0.275742021331766 | 2.28581524750297 | 0.022265069286348 | 0.0464637220363192 | 760.461390423999 | 765.038402954949 | 911.484606953365 | 1211.03518086515 | 1244.45549565264 | 1317.33445926548 |
| YDR416W | 737.084668823732 | -0.453739639490273 | 0.313897386586778 | -1.44550308119509 | 0.148316628170753 | 0.222336844836976 | 952.466552618527 | 956.298003693687 | 647.402790653072 | 615.101322079708 | 622.227747826319 | 629.011596071077 |
| YDR417C | 19.1253312935842 | 0.31286401304622 | 0.57165066608032 | 0.547299306395387 | 0.584173120295743 | 0.670601585508839 | 23.4337008977574 | 14.8391069538676 | 12.4359858091551 | 13.9399733049226 | 23.5692328722091 | 26.5339879235938 |
| YDR418W | 75694.4039759027 | 0.46709590119561 | 0.347429877858448 | 1.34443216016648 | 0.178808680025611 | 0.258770314464031 | 69988.9053232473 | 70843.5453874252 | 49806.854694243 | 63752.7254134002 | 98245.9903045162 | 101528.402732584 |
| YDR419W | 343.865879282036 | -0.994838194225758 | 0.419144000904396 | -2.37349978069392 | 0.0176204035130772 | 0.0381558166187684 | 498.911051371609 | 445.997603446797 | 430.870331858372 | 353.72682261241 | 185.411298594711 | 148.278167808318 |
| YDR420W | 1147.48377431929 | -1.78816335186315 | 0.59343611090405 | -3.01323650348651 | 0.00258477395108813 | 0.00768168933440117 | 1728.04645975076 | 1793.88315175643 | 1819.31157102168 | 1051.5967361901 | 295.401051998354 | 196.663675198401 |
| YDR421W | 289.80030181938 | -2.64510435125789 | 0.522583256856262 | -5.06159414132442 | 4.15765354715978e-07 | 7.99920475004594e-06 | 511.005864738194 | 514.422374400742 | 476.225103632938 | 146.369719701687 | 37.7107725955345 | 53.0679758471876 |
| YDR422C | 784.439796956204 | -0.403019723691127 | 0.383610784783592 | -1.05059539428352 | 0.293444457291754 | 0.387488682583329 | 832.274344788094 | 817.799672124256 | 1031.45529358286 | 977.540628007694 | 507.524147848235 | 540.044695386086 |
| YDR423C | 281.1275681715 | -0.899805833747409 | 0.348074611314655 | -2.58509470239413 | 0.00973522537284887 | 0.0232806726331774 | 364.356252668357 | 319.040799508152 | 416.23976031819 | 253.533264483279 | 171.269758871386 | 162.325573179633 |
| YDR424C | 1016.73129145128 | 0.159857761110135 | 0.355903979272543 | 0.449159802699816 | 0.653316382608287 | 0.732223721544965 | 1025.03543281803 | 908.483103509002 | 946.597978649803 | 702.226155235474 | 1238.17036688672 | 1279.87471160864 |
| YDR425W | 145.024267230629 | -1.49644395311624 | 0.334058078957454 | -4.47959216489067 | 7.47857936044472e-06 | 7.3529848979029e-05 | 242.652193167101 | 191.259600738737 | 209.217173024609 | 84.511088161093 | 70.7076986166272 | 71.7978496756068 |
| YDR426C | 7.90556109614828 | 1.09736846478438 | 0.918383614710681 | 1.19489116226239 | 0.232129556271244 | 0.320659447101417 | 6.04740668329224 | 7.41955347693378 | 1.46305715401824 | 4.3562416577883 | 7.85641095740302 | 20.2906966474541 |
| YDR427W | 2357.06974316332 | -0.690673994000616 | 0.4793446384197 | -1.44087142870237 | 0.149621009176394 | 0.223830445776032 | 2611.72376134683 | 2670.21485686539 | 3452.08335490604 | 3064.18038208829 | 1385.87089288589 | 958.345210887447 |
| YDR428C | 1384.53580302429 | 0.487817020806407 | 0.371946644709881 | 1.31152418698898 | 0.189680727630923 | 0.27254437437375 | 1330.42947032429 | 1403.12000197125 | 724.21329123903 | 1408.80855212874 | 1729.98169282014 | 1710.66180966228 |
| YDR429C | 4258.70126973953 | -0.670432123802076 | 0.309119943350292 | -2.1688413776731 | 0.0300947286255285 | 0.0593780797636041 | 5915.11966209522 | 5487.99638843868 | 4290.41510415849 | 3868.34259211601 | 3032.57462955757 | 2957.75924207119 |
| YDR430C | 968.994304734973 | 0.272967273896984 | 0.362516600782525 | 0.752978686514658 | 0.451462721792292 | 0.548350163126912 | 687.892510224492 | 749.374901170311 | 1196.04922340991 | 1262.43883242705 | 931.770339547998 | 986.440021630076 |
| YDR431W | 6.42921840631272 | 0.586229023261303 | 0.798943378430053 | 0.733755406313349 | 0.463097823411989 | 0.559120731678517 | 5.29148084788071 | 4.1219741538521 | 5.85222861607297 | 6.09873832090362 | 6.28512876592241 | 10.9257597332445 |
| YDR432W | 3286.2409884915 | 0.161486525818577 | 0.541975651778436 | 0.297959004779415 | 0.765734452674876 | 0.820520777907854 | 2589.8019121199 | 2863.94764209644 | 3855.15560083807 | 6587.50863490746 | 1896.53760511709 | 1924.49453587007 |
| YDR433W | 315.60471318958 | 1.82315371386742 | 0.330328028589747 | 5.51922197353618 | 3.40503857290755e-08 | 1.23193530390588e-06 | 161.012202942656 | 150.039859200216 | 106.071643666323 | 438.237910773503 | 516.951840997119 | 521.314821557667 |
| YDR434W | 2320.26767634224 | 0.305001131244259 | 0.314640514056282 | 0.969363821944754 | 0.332363696294331 | 0.428255743124347 | 2123.39567167099 | 2275.32973292636 | 1828.08991394579 | 1978.60496096744 | 2881.73153917543 | 2834.45423936743 |
| YDR435C | 1110.78588144592 | 1.53727657514095 | 0.358716181040655 | 4.2854954874944 | 1.82332349320258e-05 | 0.000146012310477632 | 491.351793017494 | 532.559060677691 | 683.247690926519 | 1144.82030766676 | 1822.6873421175 | 1990.04909426954 |
| YDR436W | 709.980413667647 | 0.0017494626861696 | 0.382642194233503 | 0.00457205899541233 | 0.996352037425819 | 0.996971274242118 | 545.022527331713 | 616.647333416274 | 967.812307383067 | 908.712009814639 | 637.940569741125 | 583.747734319064 |
| YDR437W | 126.231447209117 | 0.601489745095074 | 0.341710570387787 | 1.76023160305659 | 0.0783685456488461 | 0.131388088084053 | 89.1992485785605 | 101.400564184762 | 109.729286551368 | 128.073504738976 | 182.26873421175 | 146.717344989283 |
| YDR438W | 871.849503895295 | 1.00872612433225 | 0.404373987589685 | 2.49453761935795 | 0.0126121356663471 | 0.0288726892729454 | 619.859185037454 | 647.149942154779 | 468.909817862847 | 674.346208625628 | 1470.72013122585 | 1350.11173846521 |
| YDR439W | 186.680551220339 | -3.26820844333898 | 0.609484693093719 | -5.36224860176502 | 8.21923144493012e-08 | 2.45054863450694e-06 | 426.342171172103 | 464.134289723746 | 125.822915245569 | 58.3736382143632 | 21.9979506807285 | 23.412342285524 |
| YDR440W | 296.477442721418 | -1.15139210494015 | 0.317779588386071 | -3.62324122448456 | 0.000290934260379432 | 0.00130565619292233 | 421.806616159633 | 437.753655139093 | 367.958874235588 | 208.228351242281 | 194.838991743595 | 148.278167808318 |
| YDR441C | 3516.10603286364 | 1.38271444944887 | 0.443409788923439 | 3.11836699141438 | 0.00181856204288743 | 0.00577695894068696 | 2201.25603271837 | 2215.14891028012 | 1430.86989662984 | 2605.0325113574 | 6346.40877139016 | 6297.92007480594 |
| YDR443C | 520.354663193976 | 0.0711210691428514 | 0.280899277706216 | 0.253190644431755 | 0.800120895699761 | 0.848473335798857 | 470.185869625971 | 493.812503631481 | 558.156304257959 | 497.482797319424 | 551.520049209692 | 550.97045511933 |
| YDR444W | 443.797158985684 | -0.784353707909861 | 0.600101330754172 | -1.30703544170471 | 0.191200674906029 | 0.27429992122852 | 551.069934015005 | 597.686252308554 | 537.673504101704 | 666.50497364161 | 120.988728744006 | 188.859561103227 |
| YDR445C | 2.7311923697219 | 2.57079811802142 | 1.64829044207498 | 1.55967543850168 | 0.118836599225826 | 0.185397597097294 | 0 | 0.82439483077042 | 1.46305715401824 | 0 | 7.85641095740302 | 6.24329127613972 |
| YDR446W | 1.17596160616246 | -0.912424134866305 | 1.77889711973217 | -0.512915628872162 | 0.608010340378193 | 0.692656393425714 | 1.51185167082306 | 1.64878966154084 | 1.46305715401824 | 0.87124833155766 | 0 | 1.56082281903493 |
| YDR447C | 35042.1600979862 | -0.0170951737599097 | 0.400261209954863 | -0.0427100436783208 | 0.96593267317931 | 0.976238372072192 | 43018.9833674347 | 41265.9076490441 | 21463.7799780246 | 25807.2468290694 | 39211.3470883985 | 39485.6956759457 |
| YDR448W | 202.98643226166 | -1.26846201479941 | 0.419963936033795 | -3.0204070063229 | 0.00252435213011512 | 0.00753677687433537 | 309.929592518727 | 270.401504492698 | 282.370030725521 | 182.962149627109 | 84.8492383399526 | 87.4060778659561 |
| YDR449C | 578.626774772541 | -1.51849866506587 | 0.365764800424452 | -4.15157134667889 | 3.30200290989988e-05 | 0.000234453128332472 | 1069.63505710731 | 917.551446647477 | 586.685918761315 | 319.748137681661 | 329.969260210927 | 248.170828226554 |
| YDR450W | 53186.679161784 | 0.0817004748355092 | 0.325197977365536 | 0.251233035018771 | 0.801633945173471 | 0.849685270949063 | 56936.3339231964 | 57979.6884480836 | 40126.5370346813 | 44248.0890148189 | 58910.5119229908 | 60918.9146269333 |
| YDR451C | 288.735021357279 | -0.971996667694766 | 0.576668972590079 | -1.68553661440999 | 0.0918850867956097 | 0.149504790036313 | 316.732925037431 | 318.216404677382 | 514.264589637412 | 378.121775896024 | 91.134367105875 | 113.94006578955 |
| YDR452W | 1021.48915926345 | -1.58655546360304 | 0.423446375588886 | -3.74676831605092 | 0.000179127387086341 | 0.000886687450296724 | 1561.74277596022 | 1550.68667667916 | 1487.92912563655 | 812.874693343296 | 361.394904040539 | 354.306779920929 |
| YDR453C | 384.740889160768 | 1.20460380128879 | 0.395901108074068 | 3.04268863289826 | 0.00234474808656064 | 0.0071126602343149 | 171.595164638417 | 204.449918031064 | 321.872573884013 | 393.804245864062 | 595.515950571149 | 621.207481975902 |
| YDR454C | 13578.0976081749 | 1.15508993753885 | 0.345168925568665 | 3.34644822281103 | 0.000818539647490572 | 0.003020856922544 | 8637.20859541214 | 8963.64499496677 | 7644.47362974531 | 12779.4705272878 | 20376.3874591205 | 23067.4004425172 |
| YDR455C | 2.58655803342323 | 1.2054279006276 | 1.51263939080877 | 0.796903682366152 | 0.425506972117768 | 0.522851536050072 | 1.51185167082306 | 0.82439483077042 | 2.19458573102736 | 0 | 9.42769314888362 | 1.56082281903493 |
| YDR456W | 3642.04662572711 | 1.37768662020152 | 0.37052488779019 | 3.71820265142794 | 0.000200645285056475 | 0.000970815654217656 | 1901.90940189541 | 2151.6705083108 | 2018.28734396817 | 3236.6875517367 | 6239.56158236948 | 6304.16336608208 |
| YDR457W | 4970.33027244484 | 0.462266482466671 | 0.272266847755267 | 1.69784344395168 | 0.0895373138999363 | 0.146238980856097 | 4124.3313580053 | 4289.32630449849 | 4128.01576006247 | 5034.07285974016 | 6333.83851385831 | 5912.39683850432 |
| YDR458C | 341.467499437064 | -1.99005239928142 | 0.50739637052949 | -3.92208639018192 | 8.77855035870848e-05 | 0.000505217732887244 | 709.058433616015 | 720.521082093347 | 207.022587293581 | 127.202256407418 | 139.844115041774 | 145.156522170248 |
| YDR459C | 970.217834104273 | 0.688961402280414 | 0.392282675676733 | 1.75628811823483 | 0.0790392250416161 | 0.132279784113308 | 761.973242094822 | 841.707122216598 | 623.99387618878 | 713.552383545723 | 1461.29243807696 | 1418.78794250275 |
| YDR460W | 225.415509663655 | -2.34843755155997 | 0.457891376312576 | -5.12880930510652 | 2.91580516299706e-07 | 6.36535093210207e-06 | 491.351793017494 | 450.943972431419 | 188.734372868353 | 83.6398398295353 | 70.7076986166272 | 67.115381218502 |
| YDR461C-A | 63.2129194901594 | -0.52129677921789 | 0.466590140970848 | -1.11724773723939 | 0.263888414683151 | 0.354493406457967 | 73.3248060349184 | 65.9515864616336 | 85.5888435100672 | 79.283598171747 | 31.4256438296121 | 43.703038932978 |
| YDR461W | 1589.59680263675 | 1.57171307038858 | 0.423685813211994 | 3.70961930132449 | 0.000207571130425452 | 0.000993256029590527 | 718.885469476365 | 567.183643570049 | 1114.11802278489 | 1503.77462026852 | 2905.30077204764 | 2728.31828767306 |
| YDR462W | 289.82815226579 | -0.0231049241502006 | 0.52953655590489 | -0.0436323496320628 | 0.965197464966811 | 0.975833665887389 | 233.581083142163 | 237.425711261881 | 406.729888817071 | 510.551522292789 | 158.699501339541 | 191.981206741296 |
| YDR463W | 277.487256721144 | -4.82386699769372 | 0.780600375730485 | -6.17968828567313 | 6.42282912117697e-10 | 7.45161815811373e-08 | 693.939916907784 | 659.515864616336 | 256.766530530201 | 45.3049132409983 | 4.71384657444181 | 4.68246845710479 |
| YDR464W | 171.838765158251 | -2.89915238203382 | 0.583452259940049 | -4.96896246889456 | 6.73120956208453e-07 | 1.13479030313676e-05 | 410.46772862846 | 389.938754954408 | 109.729286551368 | 56.6311415512479 | 40.8533369784957 | 23.412342285524 |
| YDR465C | 3406.25529943976 | 0.558452110048975 | 0.311458139114565 | 1.79302461523909 | 0.0729689882186911 | 0.123826161825658 | 2988.17482738178 | 3193.7055744046 | 2083.39338732198 | 3874.44133043691 | 4058.6219005944 | 4239.19477649887 |
| YDR466W | 225.299240855465 | -2.49201371229209 | 0.648887844135412 | -3.84043827421746 | 0.000122814850953778 | 0.000656371485595292 | 483.792534663379 | 450.943972431419 | 215.069401640682 | 139.399733049226 | 23.5692328722091 | 39.0205704758732 |
| YDR467C | 43.2355576617373 | 0.463264340958896 | 0.412377021640163 | 1.1233999875074 | 0.261267655124767 | 0.351927148923552 | 39.3081434413995 | 40.3953467077506 | 29.2611430803648 | 47.0474099041136 | 58.1374410847823 | 45.263861752013 |
| YDR468C | 392.192951179666 | -1.26853175551431 | 0.308983673102491 | -4.10549768787795 | 4.03445319526646e-05 | 0.000275961864009515 | 653.875847630973 | 547.398167631559 | 461.594532092755 | 228.267062868107 | 237.263610913571 | 224.75848594103 |
| YDR469W | 220.21625484473 | -2.44031378533276 | 0.450104681358881 | -5.42165830838589 | 5.90486705746418e-08 | 1.86408548284653e-06 | 460.358833765621 | 369.328884185148 | 288.222259341594 | 106.292296450034 | 48.7097479358987 | 48.3855073900828 |
| YDR470C | 562.219153997472 | 0.736857575915698 | 0.347856618085087 | 2.11827959454105 | 0.034151395729083 | 0.0659475227871949 | 325.804035062369 | 390.763149785179 | 547.914904179832 | 589.835120464536 | 751.072887527729 | 767.924826965186 |
[truncated: 1,185,938 more chars]
