# Supplementary material for: Growth-phase-dependent control of rRNA synthesis in Saccharomyces cerevisiae
Source: mSphere. 2024 Oct 3;9(10):e00493-24. doi: 10.1128/msphere.00493-24 (PMC11520348; doi:10.1128/msphere.00493-24)
Supplement: Table S2 — Differential gene expression data set. [file msphere.00493-24-s0003.docx]

**Supplemental Table 2. mRNA-seq statistics for two-way comparison between Mid and Late log subphases.** Table includes statistics output from DESeq as described in column headers. Statistics represent gene expression changes in Late log when compared to Mid log. Additionally, the individual sample’s normalized counts were appended on the table and column headers describe which individual sample the respective column values refer to.

|  | baseMean | log2FoldChange | lfcSE | stat | pvalue | padj | Mid_RNAseq_A_i503_i705_counts.txt | Mid_RNAseq_B_i503_i706_counts.txt | Mid_RNAseq_C_i503_i707_counts.txt | Late_RNAseq_A_i504_i712_counts.txt | Late_RNAseq_B_i504_i710_counts.txt | Late_RNAseq_C_i504_i711_counts.txt |
| --- | --- | --- | --- | --- | --- | --- | --- | --- | --- | --- | --- | --- |
| HRA1 | 5.84143559980013 | 0.614308502991777 | 0.907799520259706 | 0.676700625283471 | 0.498595919679782 | NA | 4.05942128802079 | 8.625578039023 | 8.75843664055088 | 6.22729483972658 | 2.22610519789107 | 5.15177759358848 |
| ICR1 | 368.222267344689 | 0.441419010681965 | 0.376274397076543 | 1.1731306039198 | 0.240743435543385 | 0.565569633353458 | 257.77325178932 | 533.55361298528 | 481.714015230298 | 277.114620367833 | 389.568409630937 | 269.609694064464 |
| IRT1 | 842.708617195315 | 0.276166646778893 | 0.332754977453745 | 0.829939942272634 | 0.40657274047871 | 0.711094406081158 | 639.358852863275 | 1142.27297745347 | 988.452135147885 | 719.25255398842 | 904.91176294272 | 662.003420776119 |
| LSR1 | 194.787700814713 | 0.355787803792829 | 0.444243342258286 | 0.800884942887836 | 0.423198257723798 | 0.722588970331446 | 94.7198300538185 | 288.340751590197 | 274.013946325806 | 171.250608092481 | 166.95788984183 | 173.443178984145 |
| NME1 | 138.724679473031 | -0.261478600383413 | 0.413364314554338 | -0.632562103638099 | 0.527019641031224 | 0.791959165822418 | 68.3335916816833 | 177.440462517044 | 133.878960076992 | 168.136960672618 | 148.035995659756 | 136.522106230095 |
| PWR1 | 2.06069233633589 | 0.595357501933594 | 1.53876911602309 | 0.386905024109321 | 0.698826521578277 | NA | 1.35314042934026 | 4.92890173658457 | 1.25120523436441 | 3.11364741986329 | 0 | 1.71725919786283 |
| Q0020 | 23.4671765028346 | 1.41887560496649 | 0.925460652953487 | 1.53315605632539 | 0.125237408645837 | 0.406244144975321 | 84.5712768337665 | 12.3222543414614 | 5.00482093745765 | 10.3788247328776 | 23.3741045778562 | 5.15177759358848 |
| Q0045 | 249.574219172123 | 0.441450709336736 | 0.460415644837083 | 0.958809098446127 | 0.337654924814119 | 0.652164149136947 | 118.399787567273 | 377.06098284872 | 367.854338903137 | 170.212725619193 | 241.532413971181 | 222.385066123236 |
| Q0050 | 4.30864005655612 | 0.751764993496156 | 1.00786155547561 | 0.745901051004367 | 0.455727190310514 | NA | 7.44227236137145 | 3.69667630243843 | 5.00482093745765 | 2.07576494657553 | 3.3391577968366 | 4.29314799465706 |
| Q0055 | 18.8335933174221 | 3.05423930192877 | 0.703668012430706 | 4.34045494178199 | 1.42188023841423e-05 | 0.00121984591200075 | 18.9439660107637 | 43.127890195115 | 38.7873622652968 | 6.22729483972658 | 3.3391577968366 | 2.57588879679424 |
| Q0060 | 3.93056499683964 | 1.1222358516606 | 1.22331402336328 | 0.917373487287606 | 0.358946960869365 | NA | 1.35314042934026 | 11.0900289073153 | 3.75361570309323 | 2.07576494657553 | 4.45221039578214 | 0.858629598931413 |
| Q0065 | 6.65986147075256 | 1.77005198880185 | 0.935423637059996 | 1.89224637765736 | 0.0584581617556384 | NA | 7.44227236137145 | 18.4833815121921 | 5.00482093745765 | 3.11364741986329 | 3.3391577968366 | 2.57588879679424 |
| Q0070 | 5.30334904880828 | 1.21734840179316 | 1.12981269209951 | 1.07747807252102 | 0.281266741718504 | NA | 2.70628085868053 | 13.5544797756076 | 6.25602617182206 | 4.15152989315105 | 0 | 5.15177759358848 |
| Q0075 | 108.956153861526 | 2.35646599694199 | 0.503119624459821 | 4.68370916652682 | 2.81729676291474e-06 | 0.0003734877977309 | 70.3633023256937 | 225.497254448744 | 251.492252107247 | 36.3258865650717 | 32.2785253694205 | 37.7797023529822 |
| Q0085 | 27.8634790504764 | 0.750351924962306 | 0.680929341778492 | 1.10195269747444 | 0.270482236730873 | 0.596597044024965 | 8.79541279071171 | 64.0757225755994 | 32.5313360934747 | 23.8712968856186 | 15.5827363852375 | 22.3243695722167 |
| Q0105 | 1.49470902981807 | 1.81511040411671 | 1.75960336411818 | 1.03154519997542 | 0.302285223379524 | NA | 2.0297106440104 | 2.46445086829228 | 2.50241046872882 | 0 | 1.11305259894553 | 0.858629598931413 |
| Q0120 | 1.24727622296175 | -1.1148326420157 | 2.02473503429404 | -0.550606683409517 | 0.58190332632407 | NA | 0 | 0 | 2.50241046872882 | 1.03788247328776 | 2.22610519789107 | 1.71725919786283 |
| Q0130 | 137.873833902498 | 1.64688167458882 | 0.449609172616687 | 3.66291831860127 | 0.000249358070432096 | 0.0106111440434364 | 96.0729704831587 | 269.857370078005 | 261.501893982162 | 65.3865958171291 | 73.4614715304053 | 60.9627015241303 |
| Q0140 | 3.54790206710046 | 1.03748681286845 | 1.17362866555337 | 0.883999209732382 | 0.376696639438552 | NA | 2.0297106440104 | 3.69667630243843 | 8.75843664055088 | 3.11364741986329 | 1.11305259894553 | 2.57588879679424 |
| Q0158 | 80.9721768260749 | 0.518326422285529 | 0.486301551664984 | 1.06585393468497 | 0.286489675419196 | 0.610753467985257 | 147.492306798089 | 83.7913295219377 | 53.8018250776697 | 49.8183587178127 | 96.8355761082615 | 54.093664732679 |
| Q0160 | 181.787225062411 | 1.91594194838569 | 0.43844577862868 | 4.36984923056655 | 1.24332350369886e-05 | 0.00109948053834785 | 139.373464222047 | 404.169942399935 | 319.057334762925 | 67.4623607637046 | 86.8181027177517 | 73.8421455081015 |
| Q0182 | 1.21905621437278 | -1.08405290431013 | 2.06359631565641 | -0.525322174732271 | 0.599359244790889 | NA | 0 | 1.23222543414614 | 1.25120523436441 | 3.11364741986329 | 0 | 1.71725919786283 |
| Q0275 | 2.35123722805227 | -0.85568096764969 | 1.49770704030982 | -0.571327332128105 | 0.567777777637798 | NA | 1.35314042934026 | 3.69667630243843 | 0 | 6.22729483972658 | 1.11305259894553 | 1.71725919786283 |
| RDN5-1 | 179.657706390072 | 1.29569184992339 | 0.429260958522415 | 3.01842463005107 | 0.00254092597057174 | 0.0463665311021237 | 125.842059928645 | 324.075289180435 | 316.554924294196 | 90.2957751760354 | 122.435785884009 | 98.7424038771124 |
| RME2 | 1811.2102167162 | 0.504197713424889 | 0.347928216449335 | 1.44914292542959 | 0.147297672549795 | 0.440892991564844 | 1342.31530590554 | 2458.28974112155 | 2573.72916708759 | 1370.00486473985 | 1780.88415831286 | 1342.0380631298 |
| RME3 | 249.251284702938 | -0.216673267861752 | 0.39390663031067 | -0.550062505144593 | 0.582276502803136 | 0.820727155005499 | 121.782638640624 | 280.94739898532 | 290.279614372543 | 271.925208001394 | 262.680413351146 | 267.892434866601 |
| RNA170 | 22.8012450994299 | -0.303173521903563 | 0.593197100338686 | -0.51108395798035 | 0.609292268456655 | 0.83595143854625 | 14.2079745080728 | 22.1800578146306 | 25.0241046872882 | 17.644002045892 | 42.2959987599303 | 15.4553327807654 |
| RPR1 | 382.811632401403 | 1.81285976753215 | 0.44458290688413 | 4.07766411947238 | 4.54904182075732e-05 | 0.00301690376529341 | 254.39040071597 | 803.410983063285 | 730.703856868816 | 149.455076153438 | 180.314521029177 | 178.594956577734 |
| RUF20 | 45.5858509349728 | -0.11485525343917 | 0.447606290926068 | -0.256598836449645 | 0.797488473315011 | 0.929585587537483 | 29.0925192308157 | 52.9856936682841 | 50.0482093745765 | 57.083536030827 | 34.5046305673116 | 49.8005167380219 |
| RUF21 | 3.3288791701464 | -0.460774978888605 | 1.12455214308033 | -0.409740874821924 | 0.68199604236749 | NA | 3.38285107335066 | 1.23222543414614 | 3.75361570309323 | 3.11364741986329 | 3.3391577968366 | 5.15177759358848 |
| RUF23 | 14.6135479423009 | 0.447639835443858 | 0.635621992278473 | 0.704254794330248 | 0.481274112962489 | 0.764853084984975 | 12.1782638640624 | 22.1800578146306 | 16.2656680467373 | 13.4924721527409 | 16.695788984183 | 6.8690367914513 |
| SCR1 | 1290.82156430025 | 0.743335381525653 | 0.465942786459242 | 1.59533617243944 | 0.110637080812384 | 0.382393323760946 | 591.322367621695 | 2251.275868185 | 2006.93319592052 | 954.851875424743 | 890.442079156428 | 1050.10399949312 |
| snR10 | 91.5916695933888 | -0.250220006586908 | 0.44354950648924 | -0.564130954777598 | 0.572665012855843 | 0.815365951041003 | 41.270783094878 | 114.596965375591 | 96.3428030460597 | 94.4473050691865 | 91.2703131135338 | 111.621847861084 |
| snR11 | 356.792224493203 | -0.0407216690085997 | 0.429051162441155 | -0.094910986318991 | 0.924385530094642 | 0.978676334770653 | 157.640860018141 | 452.226734331634 | 446.680268668095 | 339.387568765099 | 347.272410871007 | 397.545504305244 |
| snR128 | 142.116234527094 | -0.552882975887684 | 0.449858074906214 | -1.22901645369586 | 0.219065637072258 | 0.540424584502721 | 57.5084682469612 | 152.795953834122 | 136.381370545721 | 126.621661741107 | 224.836624986998 | 154.553327807654 |
| snR13 | 121.540197685528 | -0.463483848127026 | 0.4698119353796 | -0.986530594955062 | 0.323872809434871 | 0.640392469429528 | 48.0364852415794 | 133.080346887783 | 126.371728670806 | 111.053424641791 | 194.784204815469 | 115.914995855741 |
| snR14 | 1.97363562806284 | -0.184169662062854 | 1.45684303214329 | -0.126416956390907 | 0.89940188057233 | NA | 0.676570214670132 | 2.46445086829228 | 2.50241046872882 | 3.11364741986329 | 2.22610519789107 | 0.858629598931413 |
| snR161 | 27.6076863933128 | -1.37048044884941 | 0.55275361618298 | -2.47936948529295 | 0.0131614887795833 | 0.123011768300886 | 7.44227236137145 | 17.251156078046 | 22.5216942185594 | 31.1364741986329 | 40.0698935620392 | 47.2246279412277 |
| snR17a | 265.28038217914 | -0.399427457191814 | 0.466806342265692 | -0.855659876541422 | 0.392185939860251 | 0.701395389644282 | 86.6009874777769 | 292.037427892636 | 309.04769288801 | 295.796504887013 | 290.506728324785 | 317.692951604623 |
| snR17b | 163.593411255239 | 0.115366744451931 | 0.450103803701804 | 0.256311418617476 | 0.79771038090772 | 0.929585587537483 | 73.0695831843742 | 213.175000107283 | 225.216942185594 | 147.379311206862 | 164.731784643939 | 157.98784620338 |
| snR18 | 50.3858808767407 | -0.245755458615451 | 0.413816165846016 | -0.59387592583055 | 0.552595101887484 | 0.803723847583314 | 39.2410724508676 | 35.7345375902381 | 63.811466952585 | 50.8562411911004 | 53.4265247493857 | 59.2454423262675 |
| snR189 | 75.5428216849926 | -0.94817486693125 | 0.422094958486183 | -2.2463543993319 | 0.0246813167104223 | 0.174218279045558 | 30.4456596601559 | 52.9856936682841 | 72.5699035931359 | 104.826129802064 | 86.8181027177517 | 105.611440668564 |
| snR19 | 163.797238624552 | 0.619775122573758 | 0.46033203089464 | 1.34636540796269 | 0.178184701163498 | 0.488181917201042 | 82.5415661897561 | 277.250722682882 | 236.477789294874 | 125.583779267819 | 123.548838482954 | 137.380735829026 |
| snR190 | 61.0435478578516 | -1.12322200690976 | 0.440546311295719 | -2.54961164833314 | 0.0107842974107788 | 0.109537926289576 | 20.9736766547741 | 49.2890173658457 | 46.2945936714832 | 79.9169504431578 | 77.9136819261874 | 91.8733670856611 |
| snR191 | 79.4350601630938 | 0.449267916995899 | 0.473622613997373 | 0.948577841763255 | 0.342835365103534 | 0.655343424880316 | 42.6239235242183 | 112.132514507299 | 121.366907733348 | 67.4623607637046 | 52.3134721504401 | 80.7111822995528 |
| snR24 | 56.1496817878246 | 0.297614134971219 | 0.448024697854641 | 0.664280644340234 | 0.506510706868918 | 0.779869145077428 | 33.8285107335066 | 76.3979769170608 | 76.3235192962291 | 55.0077710842515 | 48.9743143536035 | 46.3659983422963 |
| snR3 | 15.9864115454459 | -0.349074751051791 | 0.63515519794763 | -0.54958969426646 | 0.582600828985356 | 0.820795505840721 | 6.76570214670132 | 22.1800578146306 | 13.7632575780085 | 16.6061195726042 | 21.1479993799652 | 15.4553327807654 |
| snR30 | 1231.75761843487 | 0.920730946111342 | 0.423123707074619 | 2.17603251889871 | 0.029552829062268 | 0.192420915992638 | 714.458146691659 | 2131.75000107283 | 1990.66752787378 | 808.510446691168 | 847.033027797552 | 898.126560482258 |
| snR31 | 1065.18257260061 | -0.736993732535047 | 0.423997580694097 | -1.73820268344118 | 0.0821751064496085 | 0.328700425798434 | 352.493081843139 | 1062.17832423397 | 983.447314210427 | 1392.83827915218 | 1345.68059212515 | 1254.45784403879 |
| snR32 | 82.9556181750974 | 0.44677131512318 | 0.485067256967494 | 0.921050243457516 | 0.357024195932945 | 0.671084067437072 | 43.3004937388884 | 135.544797756076 | 108.854855389704 | 61.235065923978 | 91.2703131135338 | 57.5281831284046 |
| snR33 | 565.28968092819 | -0.260093586384712 | 0.405144832446714 | -0.641976808179877 | 0.520888241365997 | 0.788193890604907 | 249.654409213279 | 645.686127492579 | 649.37551663513 | 639.335603545262 | 625.53556060739 | 582.150868075498 |
| snR34 | 90.4034164243029 | -0.176169955640737 | 0.419686680001807 | -0.419765420336854 | 0.674656828526416 | 0.872424623255307 | 46.006774597569 | 104.739161902422 | 105.101239686611 | 90.2957751760354 | 92.3833657124794 | 103.894181470701 |
| snR35 | 188.052710368333 | -0.204247519174161 | 0.435954798216365 | -0.468506184608599 | 0.639422649290804 | 0.85569265290655 | 79.8352853310756 | 234.122832487767 | 211.453684607586 | 201.349199817826 | 220.384414591216 | 181.170845374528 |
| snR36 | 88.3291605000813 | -0.885032615486996 | 0.484442343776361 | -1.82691010985523 | 0.0677132859409873 | 0.298731159162496 | 27.0628085868053 | 71.4690751804763 | 88.8355716398732 | 147.379311206862 | 99.0616813061526 | 96.1665150803182 |
| snR37 | 475.791378088966 | -0.267821141723997 | 0.430385982284229 | -0.622281284122136 | 0.533756925610755 | 0.795858575463196 | 191.469370751647 | 553.269219931618 | 551.781508354705 | 462.895583086343 | 581.013456649569 | 514.319129759916 |
| snR38 | 40.074456739371 | 0.0301923560957938 | 0.451041302010842 | 0.0669392269869513 | 0.946630084435732 | 0.985486971897494 | 26.3862383721351 | 43.127890195115 | 52.5506198433053 | 38.4016515116473 | 45.6351565567669 | 34.3451839572565 |
| snR39 | 66.9861738763614 | -0.353696201356772 | 0.395814009451873 | -0.89359192173762 | 0.371540270141576 | 0.683833965025226 | 53.4490469589404 | 56.6823699707225 | 66.3138774213138 | 91.3336576493232 | 83.4789449209151 | 50.6591463369533 |
| snR39B | 77.7557168749735 | -1.51217779355026 | 0.46131277568641 | -3.2779881096946 | 0.00104549799480594 | 0.0265475034626496 | 25.0330979427949 | 38.1989884585304 | 58.8066460151273 | 89.2578927027477 | 162.505679446048 | 92.7319966845926 |
| snR4 | 614.927046143553 | -0.375015959712874 | 0.385057773481732 | -0.973921280232681 | 0.330095626745757 | 0.646462981596975 | 286.865771020136 | 681.420665082817 | 639.365874760214 | 659.05537053773 | 778.023766662929 | 644.830828797491 |
| snR40 | 44.9510025982976 | -2.25158369158553 | 0.479620577441918 | -4.69451019719478 | 2.67246280331222e-06 | 0.000371594745455596 | 11.5016936493922 | 23.4122832487767 | 12.5120523436441 | 69.5381257102802 | 93.4964183114249 | 59.2454423262675 |
| snR41 | 206.29453934536 | 0.358542621455322 | 0.41472930127808 | 0.864522039678398 | 0.387301178421582 | 0.69765188767385 | 119.076357781943 | 304.359682234097 | 272.762741091442 | 180.591550352071 | 208.140836002815 | 152.836068609791 |
| snR42 | 173.030075866336 | -0.0455526643484155 | 0.446642982480279 | -0.101988984793748 | 0.918765418351808 | 0.977916809624344 | 75.0992938283846 | 223.032803580452 | 213.956095076314 | 190.970375084949 | 151.375153456593 | 183.746734171322 |
| snR43 | 245.727607431678 | 0.128441336374997 | 0.439932709713163 | 0.29195677779618 | 0.770319671185368 | 0.917720928797231 | 114.340366279252 | 342.558670692628 | 314.052513825467 | 216.917436917143 | 272.697886741656 | 213.798770133922 |
| snR44 | 1401.00177209542 | 0.0842057173030623 | 0.365675940247224 | 0.230274152699609 | 0.817878744113826 | 0.934811497547479 | 810.531117174818 | 1674.59436500461 | 1841.77410498441 | 1339.9062730145 | 1356.81111811461 | 1382.39365427957 |
| snR45 | 97.326431974319 | -0.783952223628573 | 0.419161774768632 | -1.87028558141137 | 0.0614441707207235 | 0.282093525002172 | 41.9473533095482 | 98.5780347316914 | 75.0723140618647 | 107.939777221927 | 151.375153456593 | 109.045959064289 |
| snR46 | 149.872553821282 | -0.215775438100979 | 0.441037268650446 | -0.489245361874386 | 0.624667998133855 | 0.847037427051993 | 64.2741703936625 | 152.795953834122 | 200.192837498306 | 169.174843145905 | 151.375153456593 | 161.422364599106 |
| snR47 | 57.0735132572729 | -1.13741330000504 | 0.485902640510141 | -2.34082551766108 | 0.0192411569597613 | 0.152937438798529 | 17.5908255814234 | 51.753468234138 | 38.7873622652968 | 60.1971834506903 | 96.8355761082615 | 77.2766639038271 |
| snR48 | 58.9986810622464 | -0.852204671685985 | 0.505642765196338 | -1.68538883643491 | 0.091913576060011 | 0.347807264774815 | 18.2673957960936 | 67.7723988780378 | 41.2897727340256 | 64.3487133438414 | 79.026734525133 | 83.287071096347 |
| snR49 | 389.485475360741 | -0.498088828194987 | 0.447169989860889 | -1.11386908667538 | 0.265335353069903 | 0.592894614644195 | 133.960902504686 | 389.383237190181 | 446.680268668095 | 430.721226414422 | 512.004195514946 | 424.163021872118 |
| snR5 | 336.289748927413 | -0.821170877330017 | 0.395586919090514 | -2.07582919884701 | 0.0379097508047006 | 0.22049799731046 | 125.842059928645 | 285.876300721905 | 319.057334762925 | 440.062168674012 | 440.768829182432 | 406.131800294558 |
| snR50 | 39.207728719942 | -0.669919157758507 | 0.513639179480736 | -1.30426023660376 | 0.192144867974821 | 0.506395553012046 | 13.5314042934026 | 39.4312138926766 | 38.7873622652968 | 47.7425937712371 | 54.5395773483312 | 41.2142207487078 |
| snR51 | 233.092561946188 | 0.527394716361759 | 0.449768283449286 | 1.17259205632988 | 0.240959436463983 | 0.565569633353458 | 119.752927996613 | 338.861994390189 | 367.854338903137 | 170.212725619193 | 229.28883538278 | 172.584549385214 |
| snR52 | 10.5110554025987 | -1.23032529182049 | 0.810334882439762 | -1.51829239797282 | 0.128940703255815 | NA | 2.0297106440104 | 12.3222543414614 | 5.00482093745765 | 18.6818845191797 | 15.5827363852375 | 9.44492558824554 |
| snR53 | 3.05315799506259 | 1.35732238767824 | 1.2739902828814 | 1.06541031428306 | 0.286690291288622 | NA | 2.0297106440104 | 3.69667630243843 | 7.50723140618647 | 3.11364741986329 | 1.11305259894553 | 0.858629598931413 |
| snR54 | 40.4554605891021 | -0.148629085025918 | 0.460223660392531 | -0.322949682550329 | 0.746733349300582 | 0.906988469670736 | 25.0330979427949 | 34.502312156092 | 56.3042355463985 | 39.439533984935 | 44.5221039578214 | 42.9314799465706 |
| snR55 | 104.798437997042 | -0.409059895567088 | 0.411373620653007 | -0.994375611439919 | 0.320040037012834 | 0.638537064188392 | 51.41933631493 | 112.132514507299 | 107.603650155339 | 105.864012275352 | 143.583785263974 | 108.187329465358 |
| snR56 | 47.82435048414 | -1.16486587228106 | 0.520437090832428 | -2.23824529957671 | 0.0252050630658304 | 0.176251462898289 | 11.5016936493922 | 41.8956647609688 | 36.2849517965679 | 68.5002432369924 | 71.2353663325142 | 57.5281831284046 |
| snR57 | 274.57399515784 | -0.183447420395714 | 0.440644906898219 | -0.41631576247422 | 0.677178959896955 | 0.87424576384305 | 127.195200357985 | 273.554046380444 | 371.60795460623 | 241.826616276049 | 385.116199235155 | 248.143954091178 |
| snR58 | 4.83351227267594 | -1.2519148254266 | 1.18931813961359 | -1.05263241493429 | 0.29250949488903 | NA | 1.35314042934026 | 7.39335260487685 | 0 | 3.11364741986329 | 11.1305259894553 | 6.01040719251989 |
| snR59 | 11.8969899952333 | -0.300979270028148 | 0.644176976734077 | -0.467230715934753 | 0.640334819976935 | 0.855963847727308 | 7.44227236137145 | 13.5544797756076 | 11.2608471092797 | 13.4924721527409 | 14.469683786292 | 11.1621847861084 |
| snR6 | 2.92422928693753 | -0.749456903896754 | 1.19454618056955 | -0.627398853294578 | 0.530397819225807 | NA | 2.70628085868053 | 1.23222543414614 | 2.50241046872882 | 5.18941236643882 | 3.3391577968366 | 2.57588879679424 |
| snR60 | 6.35689860963088 | -1.54586191531822 | 0.970758952900137 | -1.59242612257138 | 0.111288990562009 | NA | 1.35314042934026 | 1.23222543414614 | 7.50723140618647 | 11.4167072061654 | 8.90442079156428 | 7.72766639038271 |
| snR61 | 56.9117810226231 | -0.93955576593945 | 0.510073072933192 | -1.84200228515594 | 0.0654748156169133 | 0.2931068848645 | 16.2376851520832 | 46.8245664975534 | 55.0530303120341 | 64.3487133438414 | 94.6094709103704 | 64.3972199198559 |
| snR62 | 44.0201012283859 | -1.78233133850033 | 0.532895735033482 | -3.34461550604893 | 0.000823967117300233 | 0.0225531570963892 | 7.44227236137145 | 29.5734104195074 | 23.7728994529238 | 74.727538076719 | 66.7831559367321 | 61.8213311230617 |
| snR63 | 436.094544626265 | -0.429360324331863 | 0.411447594461326 | -1.043535872154 | 0.296700175794469 | 0.621513342006781 | 180.644247316925 | 415.25997130725 | 520.501377495595 | 528.282178903472 | 484.177880541308 | 487.701612193042 |
| snR64 | 65.492056079329 | -1.3205012202262 | 0.512604535557072 | -2.57606230266993 | 0.00999326380684891 | 0.104628925977719 | 15.561114937413 | 49.2890173658457 | 48.797004140212 | 63.3108308705536 | 103.513891701935 | 112.480477460015 |
| snR65 | 5.57101704835659 | -0.889607734588729 | 0.960558127186297 | -0.926136284114947 | 0.354375159691323 | NA | 2.0297106440104 | 7.39335260487685 | 2.50241046872882 | 8.30305978630211 | 8.90442079156428 | 4.29314799465706 |
| snR66 | 55.6204175651864 | -1.44250492613087 | 0.528979895230658 | -2.72695605095137 | 0.00639215523028812 | 0.081468089276488 | 11.5016936493922 | 41.8956647609688 | 37.5361570309323 | 72.6517731301435 | 105.739996899826 | 64.3972199198559 |
| snR67 | 136.010843117583 | 0.0996214679901994 | 0.411821823665411 | 0.241904295171928 | 0.808854321898598 | 0.931050430960128 | 75.7758640430548 | 182.369364253629 | 165.159090936102 | 121.432249374668 | 120.209680686118 | 151.118809411929 |
| snR68 | 212.22507372073 | -0.807907144512818 | 0.436915676568262 | -1.84911457253833 | 0.064441270589609 | 0.289834447065002 | 69.6867321110236 | 213.175000107283 | 181.42475898284 | 267.773678108243 | 298.298096517403 | 242.99217649759 |
| snR69 | 28.0423599730426 | -1.45667989550453 | 0.534949406896759 | -2.72302366677015 | 0.00646874236471929 | 0.0820802011311401 | 7.44227236137145 | 19.7156069463383 | 18.7680785154662 | 46.7047112979494 | 37.8437883641482 | 37.7797023529822 |
| snR7-L | 1.6057654016673 | -1.64632699498878 | 1.85455137609255 | -0.887722505945079 | 0.374690036628471 | NA | 0 | 0 | 2.50241046872882 | 2.07576494657553 | 3.3391577968366 | 1.71725919786283 |
| snR70 | 281.99963233355 | 0.869123059703519 | 0.437879031618779 | 1.98484740520799 | 0.0471614514542633 | 0.24444005677106 | 161.700281306161 | 471.942341277973 | 460.443526246103 | 189.932492611661 | 225.949677585944 | 182.029474973459 |
| snR71 | 23.7970082614412 | -1.62166528553834 | 0.554002235521326 | -2.92718184433376 | 0.00342048824358673 | 0.0552274337756644 | 6.08913193203119 | 13.5544797756076 | 16.2656680467373 | 35.288004091784 | 38.9568409630937 | 32.6279247593937 |
| snR72 | 18.1327597737181 | -1.63316108634263 | 0.635744736293611 | -2.56889438969475 | 0.0102023544937658 | 0.106237560924214 | 4.73599150269092 | 8.625578039023 | 13.7632575780085 | 28.0228267787696 | 35.6176831662571 | 18.0312215775597 |
| snR73 | 6.71341495247346 | 0.792446077308517 | 0.875196106348299 | 0.905449728992681 | 0.365227180730811 | NA | 4.73599150269092 | 12.3222543414614 | 8.75843664055088 | 6.22729483972658 | 2.22610519789107 | 6.01040719251989 |
| snR74 | 7.09562325876034 | 0.30808449760543 | 0.863807817590765 | 0.356658612403745 | 0.721347392909627 | NA | 10.8251234347221 | 2.46445086829228 | 10.0096418749153 | 8.30305978630211 | 6.67831559367321 | 4.29314799465706 |
| snR75 | 122.205894501697 | 0.295616216435401 | 0.478276937397802 | 0.618085868918921 | 0.536518736744807 | 0.79796293666817 | 56.1553278176209 | 188.53049142436 | 160.154269998645 | 132.848956580834 | 107.966102097717 | 87.5802190910041 |
| snR76 | 213.615966370997 | -0.0831455805322538 | 0.40544906104521 | -0.205070349202221 | 0.837517162016995 | 0.941803181712136 | 121.782638640624 | 261.231792038982 | 240.231404997967 | 168.136960672618 | 289.393675725839 | 200.919326149951 |
| snR77 | 75.5031608635535 | -1.00104296770772 | 0.4521154244306 | -2.21413142223238 | 0.0268197396457733 | 0.183062942450835 | 29.0925192308157 | 67.7723988780378 | 55.0530303120341 | 73.6896556034312 | 134.67936447241 | 92.7319966845926 |
| snR78 | 23.3450439487285 | -0.324441603955211 | 0.531235472651748 | -0.610730308229811 | 0.541378137738011 | 0.798861248305202 | 15.561114937413 | 29.5734104195074 | 17.5168732811018 | 18.6818845191797 | 27.8263149736384 | 30.9106655615309 |
| snR79 | 32.9085363700285 | -1.37744559353595 | 0.568398000998399 | -2.42338219190857 | 0.0153767397528644 | 0.134324468236269 | 10.8251234347221 | 28.3411849853613 | 16.2656680467373 | 40.4774164582228 | 72.3484189314597 | 29.193406363668 |
| snR8 | 439.341954307572 | -0.147370633765783 | 0.443404798479272 | -0.332361386866389 | 0.739616391685967 | 0.90301928216742 | 175.908255814234 | 572.984826877956 | 502.984504214493 | 469.122877926069 | 509.778090317055 | 405.273170695627 |
| snR80 | 34.6885388403204 | -0.92451019891519 | 0.496077193218019 | -1.86364181130351 | 0.0623720219118734 | 0.28476122474142 | 14.2079745080728 | 29.5734104195074 | 28.7777203903815 | 53.9698886109637 | 48.9743143536035 | 32.6279247593937 |
| snR81 | 64.1907561975484 | -0.532216656200856 | 0.527464886419162 | -1.00900869404588 | 0.31297046466093 | 0.634104416944316 | 19.6205362254338 | 75.1657514829147 | 63.811466952585 | 86.1442452828844 | 54.5395773483312 | 85.8629598931413 |
| snR82 | 318.63887965478 | -0.156976386425439 | 0.452946415702272 | -0.34656723396752 | 0.728916465464246 | 0.899101253967486 | 123.135779069964 | 410.331069570665 | 371.60795460623 | 317.592036826056 | 381.777041438318 | 307.389396417446 |
| snR83 | 721.01862676138 | -0.154835815377366 | 0.438173701099932 | -0.353366290557118 | 0.723813854025358 | 0.897064153944702 | 292.278332737497 | 846.5388732584 | 909.626205382927 | 782.563384858974 | 643.344402190519 | 851.760562139961 |
| snR84 | 583.905180809213 | 0.176161780688772 | 0.446798468834147 | 0.394275703648759 | 0.693377499168135 | 0.882501670628042 | 249.654409213279 | 830.5199426145 | 779.500861009028 | 538.661003636349 | 529.813037098074 | 575.281831284046 |
| snR85 | 124.847042831851 | -1.41876672347591 | 0.450098817094476 | -3.1521227552529 | 0.00162088115502585 | 0.0351578297324097 | 30.4456596601559 | 86.25578039023 | 88.8355716398732 | 174.364255512344 | 161.392626847103 | 207.788362941402 |
| snR86 | 2382.81654808088 | 0.391195016801635 | 0.39933081686095 | 0.979626415703982 | 0.327270560711884 | 0.643568656507667 | 1322.01819946544 | 3541.41589773601 | 3249.37999364438 | 2206.53813820979 | 1951.18120595152 | 2026.36585347813 |
| snR87 | 54.6358745949011 | -0.726854320344929 | 0.415224530328053 | -1.75050910352206 | 0.0800305046793681 | 0.32372648901971 | 29.7690894454858 | 49.2890173658457 | 45.0433884371188 | 63.3108308705536 | 84.5919975198606 | 55.8109239305418 |
| snR9 | 354.926443023082 | -0.271485422057578 | 0.430852340519922 | -0.630112445785878 | 0.528621017387935 | 0.792067611015232 | 148.168877012759 | 430.046676517004 | 387.873622652968 | 323.819331665782 | 454.125460369778 | 385.524689920204 |
| SRG1 | 1215.00817084563 | -0.875204297230012 | 0.364266590853168 | -2.40264772890687 | 0.0162768586905648 | 0.139640871273682 | 487.130554562495 | 1084.35838204861 | 1002.21539272589 | 1571.35406455767 | 1659.56142502779 | 1485.42920615134 |
| tC(GCA)P1 | 1.50441601622837 | -0.577974486871085 | 1.96816065528491 | -0.293662250243194 | 0.76901600296594 | NA | 0 | 2.46445086829228 | 1.25120523436441 | 0 | 4.45221039578214 | 0.858629598931413 |
| tD(GUC)D | 1.68297331059954 | -2.0685332276764 | 1.7146967850658 | -1.20635510936531 | 0.227680597058191 | NA | 0.676570214670132 | 0 | 1.25120523436441 | 3.11364741986329 | 3.3391577968366 | 1.71725919786283 |
| tD(GUC)G2 | 1.18835505804211 | -0.171057273737978 | 1.83414771178199 | -0.0932625396739641 | 0.925694991344383 | NA | 2.0297106440104 | 1.23222543414614 | 0 | 1.03788247328776 | 1.11305259894553 | 1.71725919786283 |
| tD(GUC)I1 | 1.51299165790282 | 0.611518141688679 | 1.89356778828062 | 0.322944943124505 | 0.746736938672227 | NA | 0.676570214670132 | 2.46445086829228 | 2.50241046872882 | 0 | 0 | 3.43451839572565 |
| tD(GUC)N | 13.3868074375062 | 0.0667481766737604 | 0.665199667964394 | 0.100343069740277 | 0.920071965308507 | 0.978097587681394 | 13.5314042934026 | 11.0900289073153 | 16.2656680467373 | 11.4167072061654 | 21.1479993799652 | 6.8690367914513 |
| tD(GUC)O | 38.1521206359734 | -1.16136543683135 | 0.53099848716865 | -2.18713511412037 | 0.0287326619253354 | 0.189721712869876 | 14.2079745080728 | 29.5734104195074 | 27.5265151560171 | 48.7804762445249 | 77.9136819261874 | 30.9106655615309 |
| tE(UUC)C | 14.2722607535371 | -0.69388585333372 | 0.614266957675939 | -1.12961611342244 | 0.258638018276734 | 0.584375627540796 | 8.11884257604158 | 12.3222543414614 | 12.5120523436441 | 15.5682370993165 | 23.3741045778562 | 13.7380735829026 |
| tE(UUC)E2 | 1.98867899675613 | 0.400429132925568 | 1.57050128324175 | 0.254968994421337 | 0.798747046810532 | NA | 0.676570214670132 | 3.69667630243843 | 2.50241046872882 | 0 | 3.3391577968366 | 1.71725919786283 |
| tE(UUC)G1 | 12.1350688272406 | -0.676724659180543 | 0.739236677504349 | -0.91543707147372 | 0.359962224232575 | 0.674401194553078 | 6.08913193203119 | 18.4833815121921 | 3.75361570309323 | 17.644002045892 | 12.2435785884009 | 14.596703181834 |
| tE(UUC)J | 7.07669519259132 | -1.05744233391658 | 0.938209485113758 | -1.12708552907921 | 0.259706321026691 | NA | 0.676570214670132 | 6.16112717073071 | 7.50723140618647 | 8.30305978630211 | 7.79136819261874 | 12.0208143850398 |
| tE(UUC)K | 7.16097514170689 | -0.131884376562974 | 0.8234382164119 | -0.160163050407904 | 0.872752635099046 | NA | 3.38285107335066 | 7.39335260487685 | 10.0096418749153 | 7.26517731301435 | 8.90442079156428 | 6.01040719251989 |
| tF(GAA)D | 2.79383009971049 | -0.604469364687635 | 1.24674426420779 | -0.484838296065256 | 0.627791054969187 | NA | 0.676570214670132 | 3.69667630243843 | 2.50241046872882 | 3.11364741986329 | 3.3391577968366 | 3.43451839572565 |
| tF(GAA)H1 | 1.99765225870029 | -0.592202736792996 | 1.73317732731306 | -0.34168617801566 | 0.732587075084617 | NA | 0 | 4.92890173658457 | 0 | 3.11364741986329 | 2.22610519789107 | 1.71725919786283 |
| tF(GAA)H2 | 2.90869903082668 | -2.03104207116262 | 1.49864609163703 | -1.35525130482543 | 0.175337503739696 | NA | 0 | 3.69667630243843 | 0 | 4.15152989315105 | 4.45221039578214 | 5.15177759358848 |
| tF(GAA)M | 1.65720835052259 | 3.0498049272469 | 1.86005054659862 | 1.63963551034886 | 0.101080975088661 | NA | 2.70628085868053 | 3.69667630243843 | 2.50241046872882 | 1.03788247328776 | 0 | 0 |
| tF(GAA)P1 | 21.9234329392626 | -0.508968086634733 | 0.655159908331511 | -0.776860855132172 | 0.43724086834301 | 0.735086432066576 | 5.41256171736105 | 22.1800578146306 | 27.5265151560171 | 31.1364741986329 | 17.8088415831286 | 27.4761471658052 |
| tF(GAA)P2 | 1.21060639217878 | -0.409963616932892 | 1.87181749368739 | -0.219019011370219 | 0.826635238348991 | NA | 0.676570214670132 | 2.46445086829228 | 0 | 1.03788247328776 | 2.22610519789107 | 0.858629598931413 |
| tG(CCC)O | 1.11114714910986 | 0.418323818414142 | 1.89374158771748 | 0.220898047086955 | 0.825171818061407 | NA | 1.35314042934026 | 1.23222543414614 | 1.25120523436441 | 0 | 1.11305259894553 | 1.71725919786283 |
| tG(GCC)D2 | 23.7897404599563 | -0.220495715774858 | 0.599634495502759 | -0.367716863236803 | 0.713084365185773 | 0.891399851461647 | 12.8548340787325 | 29.5734104195074 | 23.7728994529238 | 20.7576494657553 | 41.1829461609848 | 14.596703181834 |
| tG(GCC)E | 1.85596971626424 | -0.263458614035424 | 1.63943009288598 | -0.160701340776076 | 0.872328633374199 | NA | 1.35314042934026 | 1.23222543414614 | 2.50241046872882 | 5.18941236643882 | 0 | 0.858629598931413 |
| tG(GCC)G1 | 1.78243255366846 | -3.08693280974546 | 1.88809601929488 | -1.63494482176722 | 0.102060615242263 | NA | 0 | 1.23222543414614 | 0 | 4.15152989315105 | 4.45221039578214 | 0.858629598931413 |
| tG(GCC)G2 | 2.23189985233318 | -2.79682603084509 | 1.66599893322872 | -1.67876820030419 | 0.0931972283611408 | NA | 1.35314042934026 | 0 | 0 | 4.15152989315105 | 4.45221039578214 | 3.43451839572565 |
| tG(GCC)J2 | 1.55703496825885 | -1.61590547253626 | 1.86019778263382 | -0.868674012850573 | 0.385025459337548 | NA | 0 | 2.46445086829228 | 0 | 2.07576494657553 | 2.22610519789107 | 2.57588879679424 |
| tG(GCC)O2 | 2.47774475434063 | -0.286780074219354 | 1.31139809642208 | -0.218682698260568 | 0.826897227578596 | NA | 0.676570214670132 | 3.69667630243843 | 2.50241046872882 | 2.07576494657553 | 3.3391577968366 | 2.57588879679424 |
| tG(GCC)P1 | 1.98341686178211 | 0.58201371140589 | 1.51480702329643 | 0.384216406746878 | 0.700818059027738 | NA | 3.38285107335066 | 2.46445086829228 | 1.25120523436441 | 0 | 2.22610519789107 | 2.57588879679424 |
| tH(GUG)E2 | 3.70606459065648 | -1.38257506504609 | 1.14637863465771 | -1.20603701364245 | 0.227803219067986 | NA | 1.35314042934026 | 3.69667630243843 | 1.25120523436441 | 8.30305978630211 | 3.3391577968366 | 4.29314799465706 |
| tH(GUG)G2 | 1.38926511884679 | 0.105865414460095 | 1.76761165280951 | 0.0598917835214699 | 0.952241823949005 | NA | 0.676570214670132 | 2.46445086829228 | 1.25120523436441 | 0 | 2.22610519789107 | 1.71725919786283 |
| tH(GUG)M | 5.62656307835556 | -0.979025334351285 | 1.01695263233995 | -0.96270495126071 | 0.335695611278903 | NA | 0.676570214670132 | 6.16112717073071 | 5.00482093745765 | 5.18941236643882 | 5.56526299472767 | 11.1621847861084 |
| tK(CUU)E1 | 2.7909487659395 | 0.307835063284342 | 1.32817277336742 | 0.231773357696426 | 0.816714051109774 | NA | 0.676570214670132 | 4.92890173658457 | 3.75361570309323 | 2.07576494657553 | 4.45221039578214 | 0.858629598931413 |
| tK(CUU)M | 4.65754909439349 | -1.28066079426737 | 1.15647429842763 | -1.10738370581049 | 0.268128067695023 | NA | 2.0297106440104 | 6.16112717073071 | 0 | 5.18941236643882 | 11.1305259894553 | 3.43451839572565 |
| tK(CUU)P | 1.20375635235586 | 0.562738140806948 | 1.87428400215642 | 0.300241660367106 | 0.763992829666623 | NA | 0.676570214670132 | 2.46445086829228 | 1.25120523436441 | 0 | 1.11305259894553 | 1.71725919786283 |
| tK(UUU)D | 19.9345945539432 | 0.480347398497827 | 0.64057148450578 | 0.749873214959653 | 0.453331067827081 | 0.745667056750931 | 11.5016936493922 | 29.5734104195074 | 28.7777203903815 | 15.5682370993165 | 25.6002097757473 | 8.58629598931413 |
| tK(UUU)L | 4.7479624878049 | -0.161778902693138 | 1.109314222084 | -0.145836859811654 | 0.884050177044497 | NA | 1.35314042934026 | 11.0900289073153 | 1.25120523436441 | 5.18941236643882 | 4.45221039578214 | 5.15177759358848 |
| tK(UUU)O | 3.19866051688791 | -0.431566617951153 | 1.15686060863578 | -0.37304979937045 | 0.709111380291559 | NA | 2.0297106440104 | 1.23222543414614 | 5.00482093745765 | 4.15152989315105 | 3.3391577968366 | 3.43451839572565 |
| tK(UUU)P | 10.4900106382325 | 0.992799299429641 | 0.837563874701251 | 1.18534159533058 | 0.235882413726711 | NA | 4.73599150269092 | 19.7156069463383 | 17.5168732811018 | 7.26517731301435 | 11.1305259894553 | 2.57588879679424 |
| tL(CAA)A | 4.18372751072658 | -1.48983551543624 | 1.11269617750138 | -1.33894188329266 | 0.180589593297427 | NA | 0.676570214670132 | 2.46445086829228 | 3.75361570309323 | 4.15152989315105 | 8.90442079156428 | 5.15177759358848 |
| tL(CAA)C | 1.54479697398148 | -2.22163218807798 | 1.85269130192988 | -1.19913780874547 | 0.230474364721753 | NA | 1.35314042934026 | 0 | 0 | 3.11364741986329 | 2.22610519789107 | 2.57588879679424 |
| tL(CAA)K | 4.87741445608588 | -1.91677607117326 | 1.02995032807261 | -1.86103739076447 | 0.0627388966356916 | NA | 1.35314042934026 | 3.69667630243843 | 1.25120523436441 | 8.30305978630211 | 7.79136819261874 | 6.8690367914513 |
| tL(CAA)L | 9.45455264686147 | -1.06709281681136 | 0.763235930106725 | -1.39811659110722 | 0.162078058432619 | NA | 2.70628085868053 | 9.85780347316914 | 6.25602617182206 | 11.4167072061654 | 14.469683786292 | 12.0208143850398 |
| tL(CAA)N | 2.208170439378 | -2.55979588727721 | 1.80543575904189 | -1.41782717798591 | 0.156241227318904 | NA | 0.676570214670132 | 0 | 1.25120523436441 | 0 | 4.45221039578214 | 6.8690367914513 |
| tL(GAG)G | 1.3438625259932 | -2.75814238872056 | 2.01956112445844 | -1.36571374607945 | 0.172028828107694 | NA | 0.676570214670132 | 0 | 0 | 2.07576494657553 | 4.45221039578214 | 0.858629598931413 |
| tL(UAA)B2 | 1.81951017019726 | 0.08695277079128 | 1.68964425057876 | 0.0514621765862819 | 0.958957240605976 | NA | 0.676570214670132 | 0 | 5.00482093745765 | 1.03788247328776 | 3.3391577968366 | 0.858629598931413 |
| tL(UAA)J | 1.14629315687681 | -3.62133681494035 | 2.16770097111256 | -1.67058873119465 | 0.0948029398336901 | NA | 0 | 0 | 0 | 2.07576494657553 | 2.22610519789107 | 2.57588879679424 |
| tL(UAA)L | 1.89895112728112 | -0.989453546027346 | 1.48129424525397 | -0.667965564031274 | 0.504155574179488 | NA | 1.35314042934026 | 1.23222543414614 | 1.25120523436441 | 1.03788247328776 | 2.22610519789107 | 4.29314799465706 |
| tM(CAU)E | 1.94440843462282 | -0.753433146187187 | 1.61571589167234 | -0.466315365263474 | 0.640989782045579 | NA | 0.676570214670132 | 0 | 3.75361570309323 | 4.15152989315105 | 2.22610519789107 | 0.858629598931413 |
| tM(CAU)J1 | 1.10798384907348 | 0.41652801605895 | 2.04637319381128 | 0.203544503670508 | 0.83870946473634 | NA | 1.35314042934026 | 2.46445086829228 | 0 | 0 | 1.11305259894553 | 1.71725919786283 |
| tM(CAU)J2 | 4.3239671035609 | -0.140838257325244 | 1.06123644397877 | -0.132711478317891 | 0.894421564782106 | NA | 1.35314042934026 | 4.92890173658457 | 6.25602617182206 | 4.15152989315105 | 6.67831559367321 | 2.57588879679424 |
| tM(CAU)O2 | 1.43900273710628 | 0.85544771738673 | 1.69340323732154 | 0.505164805719748 | 0.613443087232347 | NA | 0.676570214670132 | 3.69667630243843 | 1.25120523436441 | 1.03788247328776 | 1.11305259894553 | 0.858629598931413 |
| tN(GUU)F | 2.22252640435974 | -0.830804430870473 | 1.56676295726833 | -0.530268109171405 | 0.595926054407571 | NA | 0 | 1.23222543414614 | 3.75361570309323 | 4.15152989315105 | 3.3391577968366 | 0.858629598931413 |
| tN(GUU)K | 4.50402480737572 | 0.716889311700009 | 1.18912540856418 | 0.602871073594857 | 0.546594460537471 | NA | 2.0297106440104 | 8.625578039023 | 6.25602617182206 | 0 | 6.67831559367321 | 3.43451839572565 |
| tN(GUU)P | 71.8490824992332 | -0.360954267334857 | 0.48482291941899 | -0.744507433285998 | 0.456569545908285 | 0.747681410222457 | 27.0628085868053 | 82.5591040877915 | 80.0771349993223 | 77.8411854965823 | 95.722523509316 | 67.8317383155816 |
| tP(AGG)C | 3.12929383917956 | -1.30147675032604 | 1.23327840086694 | -1.05529842200363 | 0.29128886259054 | NA | 0.676570214670132 | 3.69667630243843 | 1.25120523436441 | 4.15152989315105 | 5.56526299472767 | 3.43451839572565 |
| tP(UGG)F | 1.22108748368889 | -2.4558390754857 | 2.05946143679387 | -1.19246664764401 | 0.233078320867505 | NA | 0 | 1.23222543414614 | 0 | 1.03788247328776 | 3.3391577968366 | 1.71725919786283 |
| tP(UGG)L | 1.34461224390942 | -1.20790307943194 | 2.56839287564334 | -0.470295292782798 | 0.638144060086915 | NA | 0 | 0 | 2.50241046872882 | 0 | 5.56526299472767 | 0 |
| tP(UGG)M | 11.0172819230238 | -0.706388786942998 | 0.727660903535597 | -0.97076644287299 | 0.331664596626077 | 0.64667846045003 | 3.38285107335066 | 13.5544797756076 | 8.75843664055088 | 12.4545896794532 | 13.3566311873464 | 14.596703181834 |
| tP(UGG)N2 | 9.05876876532596 | -1.08604265152801 | 0.730884473218778 | -1.48592929706816 | 0.137297823367859 | NA | 4.05942128802079 | 7.39335260487685 | 6.25602617182206 | 13.4924721527409 | 11.1305259894553 | 12.0208143850398 |
| tP(UGG)O3 | 9.32619547098374 | -0.469450126821282 | 0.826223308292888 | -0.568187948838241 | 0.569907353558619 | NA | 2.70628085868053 | 11.0900289073153 | 10.0096418749153 | 11.4167072061654 | 15.5827363852375 | 5.15177759358848 |
| tQ(UUG)E1 | 1.94376014900344 | -0.157038177755616 | 1.43218672383104 | -0.109649234378843 | 0.912687562645338 | NA | 0.676570214670132 | 2.46445086829228 | 2.50241046872882 | 2.07576494657553 | 2.22610519789107 | 1.71725919786283 |
| tR(ACG)D | 1.73105545838861 | -1.21655250985904 | 1.67618524867643 | -0.725786431314597 | 0.467969706291632 | NA | 0.676570214670132 | 0 | 2.50241046872882 | 1.03788247328776 | 4.45221039578214 | 1.71725919786283 |
| tR(UCU)G2 | 10.2215488767514 | -0.0727163997349674 | 0.695613237326023 | -0.10453567562126 | 0.916744258304768 | NA | 8.79541279071171 | 11.0900289073153 | 10.0096418749153 | 7.26517731301435 | 15.5827363852375 | 8.58629598931413 |
| tR(UCU)K | 1.25209499346855 | -1.09535298259255 | 2.29375540399766 | -0.477536959992996 | 0.63297980864474 | NA | 0 | 0 | 2.50241046872882 | 4.15152989315105 | 0 | 0.858629598931413 |
| tS(AGA)D2 | 1.81482304714485 | -1.89185986927169 | 1.83850107199405 | -1.02902298948337 | 0.303468871979843 | NA | 0 | 2.46445086829228 | 0 | 3.11364741986329 | 4.45221039578214 | 0.858629598931413 |
| tS(AGA)E | 1.06511226408698 | 1.24193129103316 | 2.11301445067634 | 0.587753335352551 | 0.556697870242771 | NA | 2.0297106440104 | 2.46445086829228 | 0 | 1.03788247328776 | 0 | 0.858629598931413 |
| tS(AGA)J | 2.6384711788278 | -2.24836462868363 | 1.43462751581425 | -1.56721142171007 | 0.117065281593604 | NA | 1.35314042934026 | 0 | 1.25120523436441 | 3.11364741986329 | 6.67831559367321 | 3.43451839572565 |
| tS(GCU)F | 6.92408750819353 | -1.64967545462223 | 0.850545276840655 | -1.93955042669796 | 0.0524343505076508 | NA | 2.70628085868053 | 3.69667630243843 | 3.75361570309323 | 11.4167072061654 | 12.2435785884009 | 7.72766639038271 |
| tS(GCU)O | 4.980444784937 | -0.883526288838999 | 0.952073863183686 | -0.928001831585349 | 0.353406619532674 | NA | 2.0297106440104 | 4.92890173658457 | 3.75361570309323 | 6.22729483972658 | 7.79136819261874 | 5.15177759358848 |
| tS(UGA)E | 5.27645187841682 | -0.0990007631167319 | 0.905998996476069 | -0.109272486505836 | 0.912986368483984 | NA | 5.41256171736105 | 6.16112717073071 | 3.75361570309323 | 4.15152989315105 | 4.45221039578214 | 7.72766639038271 |
| tT(AGU)I1 | 1.99267208999166 | 0.879216704282422 | 1.53680857687089 | 0.572105542300268 | 0.567250474593472 | NA | 2.70628085868053 | 3.69667630243843 | 1.25120523436441 | 2.07576494657553 | 2.22610519789107 | 0 |
| tT(AGU)N1 | 1.35448543043382 | 0.303514426731112 | 1.76188813723947 | 0.172266570343483 | 0.863227964434536 | NA | 2.0297106440104 | 1.23222543414614 | 1.25120523436441 | 1.03788247328776 | 0 | 2.57588879679424 |
| tT(CGU)K | 3.37115718400165 | -1.30522001782681 | 1.24274702714432 | -1.05027007855819 | 0.293593957883988 | NA | 2.0297106440104 | 0 | 3.75361570309323 | 5.18941236643882 | 6.67831559367321 | 2.57588879679424 |
| tV(AAC)E2 | 2.69388711154068 | -1.14378635237132 | 1.27401054159569 | -0.897784056746292 | 0.369300686065532 | NA | 1.35314042934026 | 1.23222543414614 | 2.50241046872882 | 2.07576494657553 | 5.56526299472767 | 3.43451839572565 |
| tV(AAC)G3 | 5.14838677458409 | -0.729252285287491 | 1.1621158874433 | -0.627521138956182 | 0.530317684008845 | NA | 0.676570214670132 | 4.92890173658457 | 6.25602617182206 | 10.3788247328776 | 7.79136819261874 | 0.858629598931413 |
| tV(AAC)L | 2.59050645678565 | -0.125407138303681 | 1.2480194709933 | -0.100484921284016 | 0.919959353317132 | NA | 1.35314042934026 | 3.69667630243843 | 2.50241046872882 | 2.07576494657553 | 3.3391577968366 | 2.57588879679424 |
| tV(AAC)M2 | 1.11594992616659 | -2.47586280861533 | 2.06273421875171 | -1.20028202669447 | 0.23002982767483 | NA | 0.676570214670132 | 0 | 0 | 2.07576494657553 | 2.22610519789107 | 1.71725919786283 |
| tV(AAC)O | 1.52142120884109 | 1.7483618684497 | 1.77833406459939 | 0.983145913500539 | 0.325535629900771 | NA | 2.0297106440104 | 3.69667630243843 | 1.25120523436441 | 1.03788247328776 | 1.11305259894553 | 0 |
| tV(CAC)H | 1.68547233156648 | -4.17198403820647 | 2.25019766171959 | -1.85405225024466 | 0.0637316851232897 | NA | 0 | 0 | 0 | 0 | 6.67831559367321 | 3.43451839572565 |
| tV(UAC)D | 1.56224553106141 | -0.237956752961455 | 1.58101544885283 | -0.150508809470593 | 0.880363200956247 | NA | 0.676570214670132 | 2.46445086829228 | 1.25120523436441 | 1.03788247328776 | 2.22610519789107 | 1.71725919786283 |
| tW(CCA)G1 | 2.37194291774888 | -1.21958063423294 | 1.66962272488915 | -0.730452823894041 | 0.465113439566522 | NA | 0.676570214670132 | 3.69667630243843 | 0 | 0 | 5.56526299472767 | 4.29314799465706 |
| tW(CCA)K | 3.18025732073961 | -1.82848166745148 | 1.25055879917847 | -1.46213170356537 | 0.14370512246235 | NA | 0.676570214670132 | 2.46445086829228 | 1.25120523436441 | 3.11364741986329 | 5.56526299472767 | 6.01040719251989 |
| tW(CCA)P | 5.064225681486 | 0.562922082717315 | 0.995935123118401 | 0.565219630928101 | 0.571924385787672 | NA | 6.76570214670132 | 2.46445086829228 | 8.75843664055088 | 6.22729483972658 | 4.45221039578214 | 1.71725919786283 |
| tX(XXX)D | 4.95642815429954 | -0.703580677991865 | 0.961822453926442 | -0.731507852743135 | 0.464469009770323 | NA | 2.70628085868053 | 2.46445086829228 | 6.25602617182206 | 6.22729483972658 | 7.79136819261874 | 4.29314799465706 |
| tY(GUA)D | 2.83623393304617 | -0.630428784325039 | 1.24871947612801 | -0.50486021590682 | 0.613657018722061 | NA | 0.676570214670132 | 3.69667630243843 | 2.50241046872882 | 3.11364741986329 | 4.45221039578214 | 2.57588879679424 |
| tY(GUA)F1 | 4.08069718187545 | -3.37393873838662 | 1.3767609033587 | -2.45063520481709 | 0.0142604395059221 | NA | 0 | 1.23222543414614 | 1.25120523436441 | 6.22729483972658 | 8.90442079156428 | 6.8690367914513 |
| tY(GUA)J1 | 2.16581390040812 | -0.784162531167601 | 1.72675024009903 | -0.454126203638245 | 0.649737994146798 | NA | 0 | 4.92890173658457 | 0 | 1.03788247328776 | 4.45221039578214 | 2.57588879679424 |
| YAL001C | 200.581290896108 | 0.367182044886629 | 0.300068150266661 | 1.22366217327739 | 0.221079694911777 | 0.542830451240022 | 191.469370751647 | 230.426156185329 | 256.497073044704 | 192.008257558236 | 172.523152836558 | 160.563735000174 |
| YAL002W | 797.543894988851 | 0.183296763690989 | 0.262935689322033 | 0.697116333517186 | 0.485729993164031 | 0.766605162192985 | 761.141491503898 | 931.562428214484 | 852.070764602164 | 720.290436461708 | 816.980607626022 | 703.217641524827 |
| YAL003W | 40000.2122648788 | -0.0965271870323418 | 0.493995492222697 | -0.195400947077522 | 0.845079075241106 | 0.945820131308182 | 71970.1565855353 | 20387.1698079479 | 23629.0108509719 | 40694.3338951399 | 30866.0616213586 | 52454.5408283189 |
| YAL005C | 15188.974913625 | -0.232819512890946 | 0.278561726826985 | -0.835791461888625 | 0.403272230609394 | 0.708823635381182 | 16355.4083694358 | 12540.3582433053 | 13001.2735902806 | 14288.5280097526 | 19079.9476511244 | 15868.3336178514 |
| YAL007C | 4315.41646182493 | 0.0163513258630624 | 0.273991158211542 | 0.0596782975399445 | 0.952411856975237 | 0.986211737518731 | 3449.831524603 | 4608.52312370657 | 4962.27995948926 | 4239.74990338051 | 4418.81881781377 | 4213.29544195644 |
| YAL008W | 824.084400203209 | 0.522810158841843 | 0.412774606608046 | 1.26657539119959 | 0.20530715632278 | 0.523757231214639 | 453.302043828988 | 1175.54306417542 | 1287.49018616098 | 687.0781973165 | 694.544821742014 | 646.548087995354 |
| YAL009W | 807.174048059948 | 0.184042954022192 | 0.336372062482489 | 0.547141021950278 | 0.584281851344724 | 0.821941282802123 | 549.375014312147 | 1022.7471103413 | 1004.71780319462 | 755.578440553492 | 801.397871240785 | 709.228048717347 |
| YAL010C | 90.4974204316194 | 0.485204610370184 | 0.555286714353028 | 0.873791138575144 | 0.382232007123998 | 0.692792642172087 | 202.97106440104 | 65.3079480097455 | 47.5457989058476 | 88.2200102294599 | 55.6526299472767 | 83.287071096347 |
| YAL011W | 57.6049249579577 | -0.35000418376471 | 0.411262456409706 | -0.851048225554609 | 0.394742564178917 | 0.702080855414639 | 33.8285107335066 | 66.5401734438917 | 52.5506198433053 | 63.3108308705536 | 58.9917877441133 | 70.4076271123758 |
| YAL012W | 24326.7506609176 | 0.0527705171048005 | 0.281635735327236 | 0.187371524581161 | 0.851369347290765 | 0.948177537979727 | 30298.8439235725 | 20215.8904726016 | 23799.1747628455 | 23351.3177665014 | 22511.4888136734 | 25783.7882263114 |
| YAL013W | 348.127612364788 | 0.210372508035959 | 0.317145868240866 | 0.663330439090525 | 0.507118945774311 | 0.779869145077428 | 471.569439625082 | 332.700867219458 | 315.303719059832 | 269.849443054819 | 336.141884881551 | 363.200320347987 |
| YAL014C | 1891.24232880313 | 0.362893055270265 | 0.326965584963093 | 1.10988150422996 | 0.26705009160519 | 0.593596753967041 | 1394.41121243514 | 2407.76849832156 | 2582.48760372815 | 1555.78582745836 | 1735.24900175609 | 1671.75182911946 |
| YAL015C | 592.675691830621 | 0.45515255372697 | 0.286079424098685 | 1.59100066410216 | 0.111609426311826 | 0.384150288886454 | 812.560827818828 | 606.254913599902 | 636.863464291485 | 526.206413956896 | 434.090513588759 | 540.078017727859 |
| YAL016C-A | 31.3358076894824 | -0.750887239810179 | 0.490635101976786 | -1.53043929548625 | 0.125908028595887 | 0.406812449898347 | 14.8845447227429 | 22.1800578146306 | 33.7825413278391 | 44.6289463513738 | 35.6176831662571 | 36.9210727540507 |
| YAL016W | 5436.68407367071 | 0.555105215658079 | 0.269582770394154 | 2.05912720181066 | 0.0394820546278969 | 0.22284687967803 | 5390.23490027694 | 6891.83685317937 | 7128.11622017405 | 4696.41819162713 | 4583.55060245771 | 3929.94767430908 |
| YAL017W | 508.834612496357 | 0.0778584511239776 | 0.356645103002682 | 0.218307921427964 | 0.827189202898359 | 0.937992412361367 | 765.200912791919 | 381.989884585304 | 419.153753512078 | 498.183587178127 | 474.160407150798 | 514.319129759916 |
| YAL018C | 21.0025061251814 | -0.747078757723824 | 0.574001987263401 | -1.30152643074562 | 0.193078323528859 | 0.507162802399533 | 10.148553220052 | 14.7867052097537 | 22.5216942185594 | 24.9091793589063 | 35.6176831662571 | 18.0312215775597 |
| YAL019W | 43.7103703426195 | 0.570170733666076 | 0.542835220645275 | 1.05035692597158 | 0.293554041689013 | 0.61783002848447 | 85.2478470484366 | 30.8056358536536 | 40.0385674996612 | 49.8183587178127 | 21.1479993799652 | 35.2038135561879 |
| YAL019W-A | 1.34878272879684 | -0.28464841147133 | 2.02035247524706 | -0.140890470825652 | 0.887956470234896 | NA | 0 | 2.46445086829228 | 1.25120523436441 | 1.03788247328776 | 3.3391577968366 | 0 |
| YAL020C | 2093.45974216303 | 1.08372278293917 | 0.410519180995265 | 2.63988342837426 | 0.00829345485873847 | 0.093864475948495 | 1342.99187612021 | 3728.71416372623 | 3463.33608872069 | 1180.07237212819 | 1428.04648444712 | 1417.59746783576 |
| YAL021C | 940.262822585411 | 0.866909056728734 | 0.423516112577755 | 2.04693288161398 | 0.040664675053558 | 0.225450220569656 | 2031.74035465441 | 766.4442200389 | 844.563533195978 | 583.289949987723 | 663.379348971539 | 752.159528663917 |
| YAL022C | 3288.83831797292 | 0.564578783467197 | 0.345797635157038 | 1.63268549598612 | 0.10253515942904 | 0.366753015804682 | 2358.52376834008 | 4709.56560930656 | 4705.78288644455 | 2667.35795634955 | 2633.48244910513 | 2658.31723829165 |
| YAL023C | 13279.0158604825 | 0.30603108932073 | 0.253309033152018 | 1.20813334413176 | 0.22699597575516 | 0.550072110812317 | 16398.7088631747 | 12797.8933590418 | 14849.3037214368 | 12115.2021106881 | 11495.6072419095 | 12017.379866644 |
| YAL024C | 119.681407380342 | -0.0781378457926673 | 0.308431745473575 | -0.253339180999972 | 0.800006121322196 | 0.929962020180481 | 118.399787567273 | 121.990317980468 | 108.854855389704 | 135.962604000697 | 113.531365092445 | 119.349514251466 |
| YAL025C | 31.7512304842106 | 0.640204448238104 | 0.687789974607144 | 0.93081387032979 | 0.351949853579962 | 0.665025561596851 | 81.864995975086 | 22.1800578146306 | 11.2608471092797 | 20.7576494657553 | 27.8263149736384 | 26.6175175668738 |
| YAL026C | 451.622332065459 | 0.689519260554477 | 0.362166041857795 | 1.90387607026177 | 0.0569263263253336 | 0.27020026731463 | 782.791738373342 | 532.321387551134 | 356.593491793857 | 376.751337803458 | 316.106938100532 | 345.169098770428 |
| YAL026C-A | 23.5995754191045 | 0.3530538620568 | 0.529948467901161 | 0.666204137649564 | 0.505280628592343 | 0.779064660179395 | 25.0330979427949 | 35.7345375902381 | 18.7680785154662 | 26.9849443054819 | 14.469683786292 | 20.6071103743539 |
| YAL027W | 96.8271499207074 | -0.216720470309727 | 0.35652874962649 | -0.607862537135559 | 0.54327864812035 | 0.800368921142664 | 116.370076923263 | 80.0946532194992 | 71.3186983587715 | 102.750364855489 | 97.948628707207 | 112.480477460015 |
| YAL028W | 164.552874781549 | 0.236903950780786 | 0.438671231837325 | 0.540048978795669 | 0.589163255337218 | 0.82583660270659 | 280.776639088105 | 125.686994282907 | 126.371728670806 | 116.24283700823 | 202.575573008087 | 135.663476631163 |
| YAL029C | 758.118515477915 | -0.060500910287765 | 0.40184656459528 | -0.150557241539936 | 0.880324993116046 | 0.960354537944778 | 1181.29159481405 | 532.321387551134 | 511.742940855044 | 806.434681744592 | 641.118296992628 | 875.802190910041 |
| YAL030W | 1686.66886361986 | -0.689302632598861 | 0.352875248490344 | -1.9533890108411 | 0.0507735173814463 | 0.254221409328008 | 788.204300090704 | 1450.32933599001 | 1636.57644654865 | 1908.6658683762 | 2389.72392993606 | 1946.51330077751 |
| YAL031C | 150.614211477036 | -0.0385238766900846 | 0.533725805316708 | -0.0721791532399766 | 0.942459335198887 | 0.983779795011854 | 282.806349732115 | 59.1468208390148 | 102.598829217882 | 175.402137985632 | 136.905469670301 | 146.825661417272 |
| YAL031W-A | 2.42766520391075 | 0.62392392517229 | 1.38565915278509 | 0.450272293816443 | 0.652514114081593 | NA | 2.70628085868053 | 3.69667630243843 | 2.50241046872882 | 0 | 2.22610519789107 | 3.43451839572565 |
| YAL032C | 53.176993735431 | -0.258536581783406 | 0.412136122645554 | -0.627308715683127 | 0.530456891387753 | 0.792788926598233 | 63.5976001789924 | 34.502312156092 | 46.2945936714832 | 52.932006137676 | 66.7831559367321 | 54.9522943316104 |
| YAL033W | 1084.70952059433 | -0.836375280041512 | 0.288892757979066 | -2.89510642597042 | 0.00379030002682974 | 0.0587241093105589 | 612.296044276469 | 808.339884799869 | 917.133436789114 | 1510.1189986337 | 1271.1060679958 | 1389.26269107103 |
| YAL034C | 438.737600648715 | -0.328824354265392 | 0.471993786075121 | -0.69667093925058 | 0.486008749528634 | 0.766813881410433 | 684.689057246173 | 234.122832487767 | 246.487431169789 | 362.22098317743 | 626.648613206336 | 478.256686604797 |
| YAL034C-B | 2.4737760482887 | -0.803259959010379 | 1.37750603702198 | -0.583126271262622 | 0.559808299496906 | NA | 0.676570214670132 | 2.46445086829228 | 2.50241046872882 | 2.07576494657553 | 1.11305259894553 | 6.01040719251989 |
| YAL034W-A | 10.934924453542 | 0.241715541747861 | 0.880017895262152 | 0.274671166403787 | 0.783568885287753 | 0.92445688682964 | 26.3862383721351 | 4.92890173658457 | 3.75361570309323 | 10.3788247328776 | 5.56526299472767 | 14.596703181834 |
| YAL035W | 6604.36665370497 | 0.89351823745845 | 0.32430644957064 | 2.7551664132533 | 0.00586622902048376 | 0.0782345345933658 | 5639.21273927555 | 9867.66127664231 | 10253.6268956164 | 4755.57749260453 | 4808.38722744471 | 4301.73429064638 |
| YAL036C | 8118.16541924367 | 0.906741552745063 | 0.335542306571295 | 2.70231662293351 | 0.00688581558302612 | 0.0844394516952211 | 6722.40165296243 | 11537.3267399103 | 13506.7605049638 | 5968.86210387793 | 5399.41815748479 | 5574.22335626273 |
| YAL037C-A | 6.04180445566199 | -1.89726922334687 | 0.971036869356398 | -1.95385909971099 | 0.0507178826896251 | NA | 0.676570214670132 | 3.69667630243843 | 3.75361570309323 | 10.3788247328776 | 10.0174733905098 | 7.72766639038271 |
| YAL037C-B | 45.6456304835817 | 1.51500625775866 | 0.409803813251925 | 3.69690619942406 | 0.000218242934745648 | 0.00950348779483322 | 60.2147491056417 | 77.630202351207 | 65.0626721869494 | 26.9849443054819 | 23.3741045778562 | 20.6071103743539 |
| YAL037W | 103.150469358033 | -0.334683588818207 | 0.376373927229566 | -0.889231598165592 | 0.373878624204913 | 0.686309610048537 | 123.812349284634 | 87.4880058243761 | 61.3090564838562 | 116.24283700823 | 112.418312493499 | 117.632255053604 |
| YAL038W | 350404.47356551 | 0.335674727250041 | 0.251857131128051 | 1.33279818501297 | 0.182598041516642 | 0.493684639058164 | 440909.307206875 | 346161.697862071 | 385886.708740797 | 302535.47578607 | 318624.663079347 | 308308.988717901 |
| YAL039C | 2543.61595082344 | 0.144262550151057 | 0.261402467543897 | 0.551879067961864 | 0.58103121047839 | 0.819373748240869 | 2448.50760689121 | 2840.27962570686 | 2723.87379521132 | 2081.99224141525 | 2512.15971582007 | 2654.88271989593 |
| YAL040C | 3156.09569840374 | 0.101165384405042 | 0.329966605049961 | 0.306592797139954 | 0.759153336825937 | 0.912866087924729 | 2844.30118247323 | 4110.70404831153 | 2845.24070294467 | 2467.04663900501 | 3998.08493541236 | 2671.19668227562 |
| YAL041W | 103.890753013731 | 0.777960127120952 | 0.579550636189477 | 1.34235057049719 | 0.179482353650319 | 0.489636719877566 | 261.156102862671 | 57.9145954048687 | 73.8211088275003 | 103.788247328776 | 54.5395773483312 | 72.1248863102387 |
| YAL042C-A | 4.17123114335042 | 0.11545143123046 | 1.09918570792162 | 0.105033599325777 | 0.916349147758972 | NA | 2.0297106440104 | 4.92890173658457 | 6.25602617182206 | 7.26517731301435 | 1.11305259894553 | 3.43451839572565 |
| YAL042W | 11287.4863340721 | 0.631939903734696 | 0.32974922264486 | 1.91642575732558 | 0.0553109248749721 | 0.266717446460855 | 8785.94080770633 | 15431.1591118121 | 16946.3236942316 | 8999.4789258782 | 8953.39510591788 | 8608.62035888634 |
| YAL043C | 625.697964954022 | 1.17609476093381 | 0.552406047066253 | 2.12904034483308 | 0.0332509226133996 | 0.205566991375157 | 1791.55792844651 | 372.132081112135 | 437.921832027544 | 365.334630597293 | 405.151146016175 | 382.090171524479 |
| YAL044C | 2420.75961313527 | -1.15004432086446 | 0.276533270566507 | -4.15879188246853 | 3.199352259408e-05 | 0.00240328996461688 | 1653.5376046538 | 1217.43872893639 | 1640.33006225174 | 3322.26179699413 | 3037.52054252236 | 3653.46894345316 |
| YAL044W-A | 1543.14557691626 | -0.84302910547018 | 0.328537596253157 | -2.56600497198676 | 0.0102877353522717 | 0.106547572621366 | 714.458146691659 | 1299.99783302418 | 1301.25344373899 | 1937.72657762825 | 2016.85130928931 | 1988.58615112515 |
| YAL045C | 2.20139208110449 | -0.399439863959576 | 1.47079750012404 | -0.271580461569924 | 0.785944624390824 | NA | 2.0297106440104 | 3.69667630243843 | 0 | 2.07576494657553 | 1.11305259894553 | 4.29314799465706 |
| YAL046C | 1130.48386166969 | -0.426740383630479 | 0.318796306650968 | -1.33859889442726 | 0.180701286244061 | 0.491329703562377 | 662.362240162059 | 1031.37268838032 | 1201.15702498984 | 1231.96649579258 | 1342.34143432831 | 1313.70328636506 |
| YAL047C | 5.81512012301773 | -0.407514814759703 | 1.03402208072261 | -0.394106491879667 | 0.693502418269363 | NA | 10.8251234347221 | 2.46445086829228 | 1.25120523436441 | 3.11364741986329 | 7.79136819261874 | 9.44492558824554 |
| YAL047W-A | 18.6499073378935 | -0.0353276948015897 | 0.622685402826633 | -0.0567344194054049 | 0.954756755441253 | 0.986770496029859 | 7.44227236137145 | 24.6445086829228 | 23.7728994529238 | 16.6061195726042 | 22.2610519789107 | 17.1725919786283 |
| YAL048C | 319.392498466852 | -0.570873538172929 | 0.492855570164918 | -1.15829783151665 | 0.246742516015467 | 0.570275827123806 | 481.041422630464 | 128.151445151199 | 160.154269998645 | 402.698399635652 | 375.098725844645 | 369.210727540507 |
| YAL049C | 3268.90677600261 | -0.114178706704204 | 0.301456424534648 | -0.378756919446846 | 0.704868381227181 | 0.88888988716914 | 2257.71480635423 | 3379.99436586287 | 3782.39342348362 | 3308.76932484139 | 3505.00263407949 | 3379.56610139404 |
| YAL051W | 398.47367093088 | 0.409281583370628 | 0.295708679358344 | 1.38407024189728 | 0.166336947770368 | 0.470061345026585 | 370.083907424562 | 518.766907775526 | 475.457989058476 | 335.236038871948 | 387.342304433046 | 303.95487802172 |
| YAL053W | 4542.44692181577 | 0.319579405113379 | 0.256141616595642 | 1.24766685461302 | 0.212153084338134 | 0.531943568048539 | 4999.1773161976 | 5356.48396223328 | 4774.59917433459 | 3827.71056148527 | 4608.03775963451 | 3688.67275700935 |
| YAL054C | 530.731742272208 | -0.101280383485475 | 0.40712978228932 | -0.248766825448063 | 0.803541153974304 | 0.930747663909662 | 253.713830501299 | 648.150578360871 | 635.612259057121 | 495.069939758263 | 636.666086596846 | 515.177759358848 |
| YAL055W | 38.4776313126613 | 0.319376094904726 | 0.419909160500278 | 0.760583776082005 | 0.446905713168537 | 0.740443446642452 | 42.6239235242183 | 48.0567919316995 | 37.5361570309323 | 40.4774164582228 | 27.8263149736384 | 34.3451839572565 |
| YAL056C-A | 4.08500108595987 | 0.119265243715624 | 1.19586838513302 | 0.0997310784349889 | 0.920557826514713 | NA | 0.676570214670132 | 8.625578039023 | 3.75361570309323 | 5.18941236643882 | 1.11305259894553 | 5.15177759358848 |
| YAL056W | 1159.78822824284 | 0.125611096391685 | 0.259904126281115 | 0.483297815194447 | 0.628884291129333 | 0.850071340307741 | 1291.57253980528 | 1043.69494272178 | 1294.99741756717 | 1132.32977835695 | 1140.87891391917 | 1055.25577708671 |
| YAL058W | 591.752335486695 | -0.104549802585677 | 0.552045595941223 | -0.189386172726227 | 0.849790160932545 | 0.947472766798606 | 1169.11333094999 | 242.74841052679 | 297.78684577873 | 612.350659239781 | 517.569458509674 | 710.94530791521 |
| YAL059C-A | 5.15566315936248 | 1.07827180602031 | 0.959306296234188 | 1.12401201811468 | 0.261007927533924 | NA | 6.08913193203119 | 3.69667630243843 | 11.2608471092797 | 3.11364741986329 | 3.3391577968366 | 3.43451839572565 |
| YAL059W | 977.552592258084 | -0.0695115363435587 | 0.343996941931734 | -0.202070215953702 | 0.83986183177009 | 0.942531379971263 | 1336.22617397351 | 691.278468555986 | 833.302686086698 | 1069.0189474864 | 857.050501188062 | 1078.43877625785 |
| YAL060W | 3903.77612666214 | 0.543276351005853 | 0.390438319788811 | 1.39145243555938 | 0.164088273517206 | 0.465647345704174 | 2355.8174874814 | 5947.95217062343 | 5587.88257667146 | 2975.60905091602 | 3496.09821328792 | 3059.29726099262 |
| YAL061W | 678.200383557122 | 1.02855480560944 | 0.348988297480524 | 2.94724726598272 | 0.00320616717311811 | 0.052504412852088 | 755.728929786537 | 947.581358858384 | 1027.23949741318 | 336.273921345235 | 628.874718404227 | 373.503875535164 |
| YAL062W | 434.904133526246 | 0.447131709768196 | 0.404074673337139 | 1.10655712736327 | 0.268485452015217 | 0.594800638350329 | 252.360690071959 | 639.525000321848 | 614.341770072926 | 333.160273925372 | 416.28167200563 | 353.755394759742 |
| YAL063C | 279.049674608737 | -0.178665012347273 | 0.352114552758126 | -0.507405930677344 | 0.611870025280675 | 0.83752876516356 | 171.848834526213 | 305.591907668243 | 309.04769288801 | 250.129676062351 | 340.594095277334 | 297.085841230269 |
| YAL063C-A | 63.7491550640488 | 0.467910255682522 | 0.449802473417922 | 1.04025718695365 | 0.298220429123799 | 0.623561668462566 | 46.006774597569 | 88.7202312585222 | 87.5843664055088 | 57.083536030827 | 67.8962085356776 | 35.2038135561879 |
| YAL064C-A | 7.91440293940536 | 0.935877969823843 | 0.772490844574627 | 1.21150687596716 | 0.225701201012887 | NA | 8.79541279071171 | 11.0900289073153 | 11.2608471092797 | 6.22729483972658 | 6.67831559367321 | 3.43451839572565 |
| YAL064W | 17.1066400149021 | 0.00382041187604456 | 0.699886750071972 | 0.00545861437675694 | 0.995644677494293 | 0.998254726866075 | 4.73599150269092 | 24.6445086829228 | 22.5216942185594 | 18.6818845191797 | 20.0349467810196 | 12.0208143850398 |
| YAL064W-B | 9.39732214132556 | 1.94112331605586 | 0.870286564348872 | 2.23044155290179 | 0.0257181435615424 | NA | 16.2376851520832 | 12.3222543414614 | 16.2656680467373 | 7.26517731301435 | 0 | 4.29314799465706 |
| YAL065C | 11.2064334568463 | -0.054852446547455 | 0.692657321528817 | -0.0791913184811012 | 0.936880449755475 | 0.982462572950796 | 6.08913193203119 | 17.251156078046 | 10.0096418749153 | 12.4545896794532 | 11.1305259894553 | 10.303555187177 |
| YAL067C | 109.206321405221 | 0.845767786049224 | 0.393521746932936 | 2.1492275652897 | 0.0316163651891819 | 0.199569930871589 | 98.1026811271691 | 131.848121453637 | 191.434400857755 | 95.4851875424743 | 75.6875767282963 | 62.6799607219931 |
| YAL068C | 1.49868612960353 | 0.191522677703488 | 1.90667273275537 | 0.100448637258642 | 0.919988158042026 | NA | 3.38285107335066 | 1.23222543414614 | 0 | 1.03788247328776 | 3.3391577968366 | 0 |
| YAR002C-A | 11008.9896342057 | 0.487907130878978 | 0.375872672967414 | 1.29806491923736 | 0.19426502779303 | 0.508717712872136 | 6874.62995126321 | 15599.9739962902 | 16085.4944929889 | 8606.12146850214 | 9745.8885563671 | 9141.82933982275 |
| YAR002W | 310.574129071629 | -0.405145616594056 | 0.270767955505094 | -1.49628347209068 | 0.134579800281961 | 0.421331531601694 | 287.542341234806 | 272.321820946297 | 241.482610232331 | 345.614863604825 | 347.272410871007 | 369.210727540507 |
| YAR003W | 912.904966779036 | 0.603376506206833 | 0.358152380137613 | 1.68469215805573 | 0.092047980278375 | 0.347874387493819 | 654.919967800688 | 1272.88887347297 | 1376.32575780085 | 808.510446691168 | 696.770926939905 | 668.013827968639 |
| YAR007C | 863.988504595825 | 0.601788475276415 | 0.39465572290873 | 1.52484416250461 | 0.127297971778308 | 0.408229274475004 | 1652.86103443913 | 686.349566819401 | 784.505681946486 | 745.199615820614 | 625.53556060739 | 689.479567941924 |
| YAR008W | 484.277464165828 | 0.319740663405424 | 0.416221766365505 | 0.768197843657807 | 0.442369654405309 | 0.738149912216149 | 251.007549642619 | 691.278468555986 | 671.897210853689 | 448.365228460314 | 442.994934380323 | 400.121393102038 |
| YAR009C | 18.4985603601232 | -0.3511526900863 | 0.677066494994119 | -0.518638409495289 | 0.604012919034261 | 0.831560644975248 | 30.4456596601559 | 9.85780347316914 | 7.50723140618647 | 20.7576494657553 | 27.8263149736384 | 14.596703181834 |
| YAR014C | 263.673031776378 | -0.0516265101845029 | 0.451448357428994 | -0.114357510299776 | 0.9089543948352 | 0.973299154529197 | 451.272333184978 | 158.957081004852 | 165.159090936102 | 287.49344510071 | 261.567360752201 | 257.588879679424 |
| YAR015W | 845.013778658306 | -0.0302730746613315 | 0.49057491147495 | -0.0617093821009175 | 0.950794268375081 | 0.985866799441858 | 1573.70231932273 | 410.331069570665 | 523.003787964324 | 871.821277561721 | 791.380397850275 | 899.84381968012 |
| YAR018C | 87.2984685256973 | 0.619265928905322 | 0.688976632971519 | 0.898819929834866 | 0.368748582682364 | 0.681769945236982 | 246.948128354598 | 36.9667630243843 | 32.5313360934747 | 68.5002432369924 | 58.9917877441133 | 79.8525527006214 |
| YAR019C | 283.085548617749 | -0.078333718758093 | 0.342358218924454 | -0.228806304122579 | 0.819019466780604 | 0.935454344307926 | 376.849609571263 | 241.516185092644 | 206.448863670128 | 275.038855421257 | 323.898306293151 | 274.761471658052 |
| YAR019W-A | 4.28965928272663 | 0.189079473510238 | 1.0052681597151 | 0.188088592762973 | 0.85080720316504 | NA | 6.08913193203119 | 4.92890173658457 | 2.50241046872882 | 5.18941236643882 | 4.45221039578214 | 2.57588879679424 |
| YAR020C | 83.2175975762132 | 0.585871991729764 | 0.439543811267839 | 1.3329092042949 | 0.182561601662876 | 0.493684639058164 | 52.0959065296001 | 124.45476884876 | 123.869318202077 | 57.083536030827 | 75.6875767282963 | 66.1144791177188 |
| YAR023C | 308.197463737034 | -0.376759172982743 | 0.318352340060772 | -1.18346600785413 | 0.236624510557142 | 0.562729700737464 | 202.294494186369 | 319.146387443851 | 284.023588200721 | 319.667801772631 | 402.925040818284 | 321.127470000348 |
| YAR027W | 2288.03380176739 | 0.0501821783068469 | 0.30411160336986 | 0.165012376215765 | 0.868934240784432 | 0.955727902033853 | 1722.54776655016 | 2650.51690884835 | 2611.26532411853 | 2130.77271765978 | 2586.73423994942 | 2026.36585347813 |
| YAR028W | 1470.97926947027 | 0.666804484512799 | 0.310939417717743 | 2.1444836084375 | 0.031994157431689 | 0.200631623980001 | 1349.75757826691 | 1914.8783246631 | 2150.82179787242 | 1075.24624232612 | 1343.45448692726 | 991.717186765782 |
| YAR029W | 100.603984497549 | -0.167150896445475 | 0.318791415765405 | -0.524326842503437 | 0.600051230161582 | 0.829907235555527 | 87.277557692447 | 94.881358429253 | 102.598829217882 | 97.5609524890498 | 107.966102097717 | 113.339107058946 |
| YAR030C | 31.5091853349696 | 0.566662059255281 | 0.511404678206886 | 1.1080502064278 | 0.267840133694237 | 0.594189536269576 | 25.0330979427949 | 41.8956647609688 | 46.2945936714832 | 15.5682370993165 | 34.5046305673116 | 25.7588879679424 |
| YAR031W | 141.316587864574 | 0.725901976783796 | 0.31488865418259 | 2.30526558242672 | 0.0211517038675857 | 0.159553797678323 | 169.142553667533 | 165.118208175583 | 193.936811326484 | 111.053424641791 | 120.209680686118 | 88.4388486899355 |
| YAR033W | 166.403912803755 | 0.21526668551318 | 0.294236805894429 | 0.731610326107253 | 0.464406443757843 | 0.753219028984221 | 183.350528175606 | 168.814884478021 | 183.927169451568 | 145.303546260287 | 173.636205435503 | 143.391143021546 |
| YAR035W | 356.084841661926 | 0.0521527212912134 | 0.324862429185839 | 0.160537866511425 | 0.872457395265247 | 0.957251259156579 | 254.39040071597 | 414.027745873104 | 420.404958746442 | 329.008744032221 | 330.576621886824 | 388.100578716998 |
| YAR042W | 212.828678630679 | 0.154047074607246 | 0.504569204417347 | 0.305304155026924 | 0.760134511663037 | 0.912866087924729 | 424.209524598173 | 123.222543414614 | 123.869318202077 | 201.349199817826 | 183.653678826013 | 220.667806925373 |
| YAR050W | 106.296469801856 | 0.0203839205617203 | 0.363521736242381 | 0.0560734573190121 | 0.955283288651687 | 0.986770496029859 | 93.3666896244782 | 129.383670585345 | 98.8452135147885 | 75.7654205500068 | 120.209680686118 | 120.208143850398 |
| YAR053W | 4.67967529503412 | -0.47212668034715 | 1.01653218859325 | -0.464448332915568 | 0.642326568534731 | NA | 2.0297106440104 | 4.92890173658457 | 5.00482093745765 | 9.34094225958987 | 3.3391577968366 | 3.43451839572565 |
| YAR068W | 2.72916653850461 | 0.803708027168234 | 1.32396208994991 | 0.607047613575278 | 0.543819314336844 | NA | 0.676570214670132 | 4.92890173658457 | 5.00482093745765 | 2.07576494657553 | 1.11305259894553 | 2.57588879679424 |
| YAR071W | 218.450019015681 | 3.20635793155092 | 0.322081360752374 | 9.9551179368497 | 2.39538691203137e-23 | 4.5895613234521e-20 | 442.476920394266 | 357.345375902381 | 382.86880171551 | 47.7425937712371 | 35.6176831662571 | 44.6487391444335 |
| YAR073W | 22.6780627471503 | 1.87160128667931 | 0.537355590340675 | 3.4829846759252 | 0.000495856747088581 | 0.0161954729868796 | 29.7690894454858 | 33.2700867219458 | 43.7921832027544 | 10.3788247328776 | 11.1305259894553 | 7.72766639038271 |
| YAR075W | 41.3016799253393 | 1.07184460779415 | 0.536587003705827 | 1.99752249009326 | 0.0457684538256447 | 0.239681256250554 | 30.4456596601559 | 71.4690751804763 | 66.3138774213138 | 41.5152989315106 | 20.0349467810196 | 18.0312215775597 |
| YBL001C | 4586.6803324123 | -0.83474015677024 | 0.36012919746497 | -2.3178908087602 | 0.0204552560998961 | 0.156769082749603 | 1877.48234570962 | 3855.63338344328 | 4155.25258332421 | 5902.43762558751 | 6079.49329544051 | 5649.7827609687 |
| YBL002W | 18382.3297567759 | -0.46272787626741 | 0.320344794971126 | -1.44446822152711 | 0.148607312540824 | 0.443201711129469 | 10363.0259781024 | 16850.6828119485 | 19165.9617799941 | 21952.2521925095 | 19934.7720471145 | 22027.2837309865 |
| YBL003C | 20121.580326287 | -0.273663926618357 | 0.28656890670256 | -0.95496727041082 | 0.339594248701956 | 0.653494389534263 | 13846.0094432242 | 19578.8299231481 | 21232.9528271641 | 23533.9850818 | 21284.9048496355 | 21252.7998327503 |
| YBL004W | 1416.9258982856 | 0.540130768583165 | 0.334866893647742 | 1.61297153833113 | 0.106750691417966 | 0.37506294270811 | 2318.60612567454 | 1454.02601229245 | 1263.71728670806 | 1283.86061945696 | 997.295128655199 | 1184.05021692642 |
| YBL005W | 479.266786208584 | 0.526895305948126 | 0.32162847903875 | 1.63821098033003 | 0.101377689860986 | 0.364883507401972 | 403.235847943399 | 656.776156399894 | 638.11466952585 | 391.281692429487 | 441.881881781377 | 344.310469171496 |
| YBL005W-B | 6.8233616272679 | 0.107980595228916 | 1.13672672516476 | 0.0949925719510687 | 0.92432072696521 | NA | 18.2673957960936 | 2.46445086829228 | 0 | 7.26517731301435 | 7.79136819261874 | 5.15177759358848 |
| YBL006C | 941.461450775849 | -0.177374479463926 | 0.348695033556657 | -0.508680831082459 | 0.61097596079912 | 0.836562606639673 | 1242.18291413436 | 648.150578360871 | 759.481577259198 | 976.647407363786 | 853.711343391225 | 1168.59488414565 |
| YBL006W-A | 12.9747076587098 | 0.0769163942809492 | 0.711819721996083 | 0.108056003372962 | 0.913951267958278 | 0.975779548922733 | 6.76570214670132 | 19.7156069463383 | 13.7632575780085 | 7.26517731301435 | 20.0349467810196 | 10.303555187177 |
| YBL007C | 737.895986473978 | 0.52575077805439 | 0.695861399384828 | 0.755539506170592 | NA | NA | 2115.6350612735 | 203.317196634113 | 292.782024841272 | 584.327832461011 | 559.865457269604 | 671.448346364365 |
| YBL008W | 338.986396918793 | 0.554957915938235 | 0.406594566066112 | 1.36489260372456 | 0.172286807260459 | 0.480730372880155 | 635.299431575254 | 264.928468341421 | 309.04769288801 | 270.887325528106 | 232.627993179617 | 321.127470000348 |
| YBL009W | 783.189029805146 | 0.233403497805752 | 0.417583235788564 | 0.558938860093348 | 0.576203447250637 | 0.817311997560742 | 1398.47063372316 | 590.235982956002 | 549.279097885977 | 748.313263240478 | 617.744192414772 | 795.091008610488 |
| YBL010C | 17.3851017036156 | 1.40939552003151 | 1.10162203765236 | 1.27938210371593 | NA | NA | 71.716442755034 | 2.46445086829228 | 1.25120523436441 | 8.30305978630211 | 11.1305259894553 | 9.44492558824554 |
| YBL011W | 939.054367281764 | 0.309041571625622 | 0.399615638766801 | 0.773347040619614 | 0.43931702324905 | 0.736637762437438 | 1658.95016637116 | 730.709682448662 | 726.950241165723 | 815.775624004182 | 736.840820501944 | 965.099669198908 |
| YBL012C | 1.20691965239224 | 0.566763018907968 | 1.87363797496369 | 0.30249334528937 | 0.762276005688223 | NA | 0.676570214670132 | 1.23222543414614 | 2.50241046872882 | 0 | 1.11305259894553 | 1.71725919786283 |
| YBL013W | 28.198648888997 | 1.32185830042309 | 0.492039118877443 | 2.68649026004035 | 0.00722070533828598 | 0.0864679464259746 | 45.3302043828988 | 40.6634393268227 | 35.0337465622035 | 15.5682370993165 | 11.1305259894553 | 21.4657399732853 |
| YBL014C | 197.415000373237 | 0.233611356444234 | 0.541471217153177 | 0.431438179987594 | 0.66614979372742 | 0.868260547470569 | 405.942128802079 | 129.383670585345 | 103.850034452246 | 198.235552397963 | 113.531365092445 | 233.547250909344 |
| YBL015W | 2050.57239441279 | 0.319545516053619 | 0.306912929546029 | 1.04116016397965 | 0.297801220590976 | 0.622937210406412 | 1668.42214937654 | 2344.92500118011 | 2817.71418778865 | 1749.86984996317 | 2022.41657228404 | 1700.0866058842 |
| YBL016W | 843.549525782641 | 0.350404230179899 | 0.666887651353094 | 0.525432176572681 | 0.599282790339618 | 0.829727654263775 | 2231.32856798209 | 255.070664868251 | 349.086260387671 | 713.025259148694 | 645.57050738841 | 867.215894920727 |
| YBL017C | 1975.01467323366 | 0.114757101722063 | 0.410259030660397 | 0.279718648818864 | 0.779693369554952 | 0.921494475945177 | 3392.99962657071 | 1479.90274640952 | 1286.23898092661 | 1812.14279836044 | 1891.07636560846 | 1987.72752152622 |
| YBL018C | 1157.77314598956 | -0.308334813126501 | 0.351669253152253 | -0.876775010504 | 0.380608854873116 | 0.691392829405806 | 616.35546556449 | 1221.13540523883 | 1267.47090241115 | 1346.13356785423 | 1259.97554200635 | 1235.5679928623 |
| YBL019W | 148.993744332474 | -0.347171592359384 | 0.464897910817305 | -0.746769525698761 | 0.455202693734232 | 0.74691026444433 | 229.357302773175 | 87.4880058243761 | 75.0723140618647 | 162.947548306179 | 172.523152836558 | 166.574142192694 |
| YBL020W | 2105.64941792553 | 0.618701873598433 | 0.322144439016094 | 1.9205728818045 | 0.0547855767042273 | 0.265865137443021 | 1706.31008139807 | 2842.74407657515 | 3102.98898122374 | 1776.85479426865 | 1602.79574248157 | 1602.20283160602 |
| YBL021C | 248.469662107031 | -0.765066265863097 | 0.346703276315851 | -2.20668888391499 | 0.0273358023793675 | 0.184267290042603 | 248.977838998608 | 138.009248624368 | 163.907885701738 | 350.804275971264 | 267.132623746928 | 321.98609959928 |
| YBL022C | 684.523756811097 | 0.200043760389513 | 0.327119368217044 | 0.611531385255012 | 0.540847847074239 | 0.798861248305202 | 995.234785779764 | 601.326011863317 | 598.076102026189 | 576.024772674709 | 625.53556060739 | 710.94530791521 |
| YBL023C | 531.862808578057 | 0.459629328216734 | 0.267381019575649 | 1.71900506979215 | 0.0856134487874834 | 0.335135365366292 | 587.262946333674 | 591.468208390148 | 669.39480038496 | 460.819818139767 | 396.24672522461 | 485.98435299518 |
| YBL024W | 2520.02767008113 | 0.562444174153356 | 0.399081363020504 | 1.40934713136293 | 0.158732548748141 | 0.458409271254452 | 4832.06447317408 | 2229.09581037037 | 1953.13137084285 | 2067.46188678923 | 1804.25826289071 | 2234.15421641954 |
| YBL025W | 212.574387027388 | -0.196909164704866 | 0.346154276955067 | -0.56884799008399 | 0.5694593036538 | 0.814202409341303 | 136.667183363367 | 194.69161859509 | 264.004304450891 | 227.29626165002 | 230.401887981726 | 222.385066123236 |
| YBL026W | 1189.02269620891 | -0.715334480198055 | 0.395810654445141 | -1.80726433754248 | 0.0707210736831705 | 0.303672139518666 | 466.156877907721 | 1152.13078092664 | 1083.54373295958 | 1733.26373039057 | 1440.29006303552 | 1258.75099203345 |
| YBL027W | 14040.9377537495 | 0.270569046520522 | 0.544644769984291 | 0.496780766899361 | 0.619343659525997 | 0.842642554747841 | 31020.7443426255 | 6748.89870281842 | 8290.48588289859 | 12984.9476233032 | 9468.73845922966 | 15731.8115116213 |
| YBL028C | 109.645959742607 | -0.241178131316691 | 0.404884205015435 | -0.595671869460794 | 0.551394454202165 | 0.803400588784296 | 140.726604651387 | 96.1135838633991 | 63.811466952585 | 114.167072061654 | 97.948628707207 | 145.108402219409 |
| YBL029C-A | 1120.96020940117 | -0.863004629745869 | 0.280397147598442 | -3.07779389746783 | 0.00208539090076114 | 0.0409106720053755 | 657.626248659368 | 883.505636282784 | 845.814738430342 | 1418.78534098437 | 1629.50900485626 | 1290.52028719391 |
| YBL029W | 106.00169108876 | -0.534234204268257 | 0.393386232231369 | -1.3580399121697 | 0.174451019934948 | 0.483236622893869 | 119.752927996613 | 81.3268786536454 | 57.5554407807629 | 149.455076153438 | 106.853049498771 | 121.066773449329 |
| YBL030C | 36134.6000635299 | 0.897774741473235 | 0.388875899857244 | 2.30864073037904 | 0.0209635257955961 | 0.159076280224254 | 23685.370075172 | 58969.3803764978 | 58431.284444818 | 26124.5397351263 | 25205.0761031216 | 24391.9496464436 |
| YBL031W | 22.4983664267994 | -0.877253776648297 | 0.532116639070317 | -1.64861181221655 | 0.09922718722973 | 0.36144351850221 | 20.9736766547741 | 9.85780347316914 | 16.2656680467373 | 29.0607092520574 | 24.4871571768018 | 34.3451839572565 |
| YBL032W | 1245.29511291927 | 0.634179392421955 | 0.533042119873897 | 1.18973598666459 | 0.234150176412192 | 0.560322736893122 | 2880.83597406542 | 595.164884692587 | 1067.27806491284 | 964.192817684332 | 690.092611346231 | 1274.20632481422 |
| YBL033C | 404.700140535354 | 0.758321833182049 | 0.694549801712117 | 1.09181779522898 | 0.274913195688421 | 0.600152316299675 | 1220.53266726492 | 117.061416243884 | 187.680785154662 | 306.17532961989 | 240.419361372235 | 356.331283556536 |
| YBL034C | 254.947447664823 | -0.211620355107262 | 0.349698731185895 | -0.605150480213688 | 0.545079015492717 | 0.800898308039913 | 151.55172808611 | 288.340751590197 | 270.260330622713 | 284.379797680847 | 252.662939960636 | 282.489138048435 |
| YBL035C | 339.517335324587 | 0.244227903602707 | 0.410710319661474 | 0.594647594450539 | 0.552079058771203 | 0.803626278288227 | 595.381788909716 | 245.212861395082 | 262.753099216526 | 302.023799726739 | 288.280623126893 | 343.451839572565 |
| YBL036C | 901.881240428382 | 0.311081291929964 | 0.283225296171801 | 1.09835278181249 | 0.272050472311847 | 0.598448570550516 | 792.940291593394 | 1078.19725487787 | 1126.08471092797 | 851.063628095966 | 741.293030897726 | 821.708526177362 |
| YBL037W | 504.283230494471 | -0.143510976681668 | 0.337452283608611 | -0.425277835274979 | 0.670634149302605 | 0.87058290537205 | 659.655959303378 | 357.345375902381 | 419.153753512078 | 540.736768582925 | 576.561246253787 | 472.246279412277 |
| YBL038W | 325.410445204285 | -0.555564836802863 | 0.324724113976968 | -1.71088260122951 | 0.0871027870791666 | 0.338288391980439 | 336.255396691056 | 221.800578146306 | 231.472968357416 | 408.925694475379 | 316.106938100532 | 437.90109545502 |
| YBL039C | 6817.91691385112 | 0.496344802872056 | 0.280514691167168 | 1.76940751590179 | 0.0768258922675869 | 0.317010214468119 | 9179.70467264435 | 6594.87052355015 | 8162.86294899342 | 6320.70426232248 | 4878.50954117828 | 5770.84953441802 |
| YBL039W-B | 271.925902593175 | -0.585943780262449 | 0.363792101727473 | -1.61065558454976 | 0.107254811454939 | 0.375384467649401 | 129.901481216665 | 267.392919209713 | 256.497073044704 | 342.501216184962 | 314.993885501586 | 320.268840401417 |
| YBL040C | 6119.00427302247 | -0.620446706520377 | 0.36835840179199 | -1.68435606057043 | 0.0921128771338887 | 0.347874387493819 | 2675.1586288057 | 5637.4313612186 | 6158.43216354163 | 7871.3006774144 | 6866.421482895 | 7505.28132425948 |
| YBL041W | 2425.4936595119 | -0.238882404714488 | 0.280961662949126 | -0.850231316995525 | 0.395196493631528 | 0.702080855414639 | 2701.54486717784 | 1934.59393160944 | 2038.21332677963 | 2878.04809842697 | 2358.55845716559 | 2642.00327591196 |
| YBL042C | 12536.0247811094 | 0.467298153159145 | 0.355276795630638 | 1.31530727282558 | 0.188406654860169 | 0.501366434388531 | 8443.59627908325 | 17082.341193568 | 18121.2054092998 | 11014.0088065297 | 10624.0870569351 | 9930.90994124072 |
| YBL043W | 86.2379419009125 | -0.680091839497658 | 0.363555156154953 | -1.87067031778747 | 0.0613907914145975 | 0.282093525002172 | 82.5415661897561 | 64.0757225755994 | 51.2994146089409 | 122.470131847956 | 95.722523509316 | 101.318292673907 |
| YBL044W | 8.94447976019062 | 0.220570136883378 | 0.788072661752577 | 0.279885532880758 | 0.779565326909961 | NA | 5.41256171736105 | 8.625578039023 | 15.0144628123729 | 11.4167072061654 | 8.90442079156428 | 4.29314799465706 |
| YBL045C | 6240.62784976887 | -0.467122719546063 | 0.29913901082687 | -1.56155734504456 | 0.118392304669083 | 0.396341856282987 | 6840.8014405297 | 4461.88829704318 | 4413.00086160328 | 7354.43520571709 | 6758.45538079729 | 7615.1859129227 |
| YBL046W | 105.569396495089 | 0.0487276718872878 | 0.370587142824731 | 0.131487756201876 | 0.895389471618748 | 0.966310161494771 | 134.637472719356 | 76.3979769170608 | 110.106060624068 | 95.4851875424743 | 125.774943680845 | 91.0147374867297 |
| YBL047C | 1357.66462888288 | -0.260195563917993 | 0.242466522758079 | -1.07311954227018 | 0.283217483908762 | 0.608756980267008 | 1234.74064177299 | 1242.08323761931 | 1229.93474538022 | 1408.4065162515 | 1498.16879818069 | 1532.65383409257 |
| YBL048W | 109.077510360727 | 0.179894029215063 | 0.457186341207055 | 0.393480760470905 | 0.693964431321982 | 0.882501670628042 | 54.1256171736105 | 133.080346887783 | 161.405475233009 | 80.9548329164456 | 112.418312493499 | 112.480477460015 |
| YBL049W | 200.858590124309 | -0.0431486575925372 | 0.415736599795514 | -0.103788450701142 | 0.917337231973388 | 0.97754067656341 | 99.4558215565094 | 264.928468341421 | 230.221763123052 | 203.424964764402 | 225.949677585944 | 181.170845374528 |
| YBL050W | 1544.24229713553 | 0.150765961765573 | 0.270187654798609 | 0.558004627850039 | 0.576841224025684 | 0.817369867844094 | 1448.53682960875 | 1590.80303548267 | 1835.51807881259 | 1409.44439872478 | 1664.01363542357 | 1317.13780476079 |
| YBL051C | 133.739591078068 | -0.12901072398247 | 0.464287996189372 | -0.277867885970176 | 0.78111377673567 | 0.922507086229018 | 220.561889982463 | 78.8624277853531 | 82.5795454680512 | 138.038368947273 | 123.548838482954 | 158.846475802311 |
| YBL052C | 11.068173905669 | -0.319540228884756 | 0.776327128868283 | -0.411605130108716 | 0.680628870832311 | 0.87491979660087 | 18.9439660107637 | 4.92890173658457 | 5.00482093745765 | 16.6061195726042 | 8.90442079156428 | 12.0208143850398 |
| YBL053W | 10.525913671891 | -0.15367795395079 | 0.743906615445268 | -0.206582319285877 | 0.836336069575789 | NA | 5.41256171736105 | 12.3222543414614 | 12.5120523436441 | 17.644002045892 | 6.67831559367321 | 8.58629598931413 |
| YBL054W | 193.552739958071 | 0.850970103483078 | 0.646471732311213 | 1.31632995063954 | 0.188063322436267 | 0.501366434388531 | 567.642410108241 | 98.5780347316914 | 80.0771349993223 | 147.379311206862 | 119.096628087172 | 148.542920615134 |
| YBL055C | 1987.8187087895 | -0.012098676602361 | 0.255120086290429 | -0.0474234576284514 | 0.962175733566586 | 0.988087915299789 | 1960.02391189937 | 1811.37138819483 | 2167.08746591916 | 2187.85625369061 | 1894.4155234053 | 1906.15770962774 |
| YBL056W | 5729.76548988151 | 0.405344600206334 | 0.303581445533142 | 1.33520874272958 | 0.181808036233722 | 0.492551224978628 | 4669.68762165325 | 7218.3765932281 | 7701.16821751295 | 5221.58672311074 | 5018.75416864542 | 4549.01961513862 |
| YBL057C | 2382.84536083235 | -0.103059014643039 | 0.270540825818172 | -0.380937015074775 | 0.703249985510084 | 0.887726241505533 | 1908.60457558444 | 2293.17153294597 | 2692.59366435221 | 2432.79651738652 | 2389.72392993606 | 2580.18194478889 |
| YBL058W | 388.075062305903 | 1.35117364368165 | 0.705127931187612 | 1.91621063911897 | NA | NA | 1363.96555277499 | 136.777023190222 | 171.415117107924 | 207.576494657553 | 212.593046398597 | 236.123139706138 |
| YBL059C-A | 173.645341091457 | -0.283148292848371 | 0.299611454104837 | -0.945051629265466 | 0.34463251832649 | 0.656807762433896 | 148.845447227429 | 173.743786214606 | 147.642217655001 | 215.879554443855 | 170.297047638667 | 185.463993369185 |
| YBL059W | 83.054347577868 | -0.790226800762609 | 0.406425693690019 | -1.94433278464258 | 0.0518553335957734 | 0.25838576017822 | 89.3072683364574 | 43.127890195115 | 48.797004140212 | 99.6367174356253 | 102.400839102989 | 115.056366256809 |
| YBL060W | 53.832352891511 | 0.380695160274769 | 0.491908507709088 | 0.773914568072302 | 0.43898131400031 | 0.73650455133502 | 98.7792513418392 | 46.8245664975534 | 36.2849517965679 | 53.9698886109637 | 35.6176831662571 | 51.5177759358848 |
| YBL061C | 271.670354001767 | 0.783125531109 | 0.641858473711033 | 1.22009066357137 | 0.222430507477152 | 0.544983187117933 | 778.055746870652 | 121.990317980468 | 130.125344373899 | 212.765907023992 | 145.809890461865 | 241.274917299727 |
| YBL062W | 1.31113010034642 | -0.736870742561369 | 1.93505093804421 | -0.380801728819675 | 0.703350376338149 | NA | 2.70628085868053 | 0 | 0 | 2.07576494657553 | 2.22610519789107 | 0.858629598931413 |
| YBL063W | 25.8427936141349 | -0.886454482323235 | 0.587638223784483 | -1.50850378080297 | 0.131425639760553 | 0.414618319068967 | 31.1222298748261 | 9.85780347316914 | 12.5120523436441 | 32.1743566719207 | 25.6002097757473 | 43.790109545502 |
| YBL064C | 1485.86345651218 | -0.760607065846385 | 0.290793800394748 | -2.61562338954226 | 0.00890647343688461 | 0.0978860598761238 | 875.48185778315 | 1232.22543414614 | 1202.4082302242 | 1651.27101500083 | 2151.53067376172 | 1802.26352815703 |
| YBL065W | 15.3996426535427 | 0.653128087726159 | 0.747592834420304 | 0.873641449804164 | 0.382313545749802 | 0.692792642172087 | 4.73599150269092 | 30.8056358536536 | 21.270488984195 | 11.4167072061654 | 15.5827363852375 | 8.58629598931413 |
| YBL066C | 268.388679319334 | 0.0288689008766612 | 0.459245034538567 | 0.0628616505471151 | 0.949876672849303 | 0.985770615313908 | 477.658571557113 | 157.724855570706 | 176.419938045382 | 242.864498749337 | 286.054517929002 | 269.609694064464 |
| YBL067C | 489.067395037239 | 0.579478184672447 | 0.266894598071944 | 2.1711873858018 | 0.0299170104906025 | 0.193652000337819 | 628.533729428552 | 603.79046273161 | 525.506198433053 | 400.622634689077 | 359.515989459408 | 416.435355481735 |
| YBL068W | 4113.6723619789 | 0.674556670180263 | 0.319684237328627 | 2.11007172520313 | 0.0348521783041955 | 0.209331578780058 | 3393.67619678538 | 5564.73006060398 | 6217.23880955676 | 3318.11026710098 | 3122.11254004222 | 3066.16629778407 |
| YBL069W | 1184.61179074084 | 0.652732389573437 | 0.275571852709987 | 2.3686468090062 | 0.0178532933208222 | 0.147663696225841 | 1639.32963014573 | 1191.56199481932 | 1512.70712834657 | 919.563871332959 | 996.182076056254 | 848.326043744236 |
| YBL070C | 8.55893332797503 | 0.832123651222073 | 0.824632810085519 | 1.00908385046646 | 0.312934422369639 | NA | 4.73599150269092 | 13.5544797756076 | 15.0144628123729 | 4.15152989315105 | 4.45221039578214 | 9.44492558824554 |
| YBL071C | 5.6148035777622 | -0.200922201378445 | 0.967297471257207 | -0.207715007377518 | 0.835451499054329 | NA | 3.38285107335066 | 2.46445086829228 | 10.0096418749153 | 9.34094225958987 | 3.3391577968366 | 5.15177759358848 |
| YBL071C-B | 3.41962261820599 | -0.338954703809669 | 1.22023517028741 | -0.277778179209368 | 0.781182642692867 | NA | 4.05942128802079 | 4.92890173658457 | 0 | 4.15152989315105 | 2.22610519789107 | 5.15177759358848 |
| YBL071W-A | 632.854685100572 | -0.860453846127374 | 0.337932170496849 | -2.54623241362987 | 0.0108892661376126 | 0.109838670351362 | 295.661183810848 | 499.051300829188 | 555.535124057799 | 831.343861103499 | 715.692821121979 | 899.84381968012 |
| YBL072C | 18316.7859012816 | 0.00181706283758751 | 0.242264317783329 | 0.00750033209270469 | 0.994015656930574 | 0.998254726866075 | 18669.2785036076 | 16781.6781876363 | 19533.8161188972 | 18165.0190474824 | 18856.2240787363 | 17894.6994713296 |
| YBL073W | 2.19851074733351 | 0.704220447080548 | 1.38414144196969 | 0.508777806752474 | 0.610907977232437 | NA | 2.0297106440104 | 4.92890173658457 | 1.25120523436441 | 1.03788247328776 | 2.22610519789107 | 1.71725919786283 |
| YBL074C | 43.9607897891404 | 0.102187300530257 | 0.466057261743941 | 0.219259110238691 | 0.826448212194332 | 0.937978507797441 | 66.3038810376729 | 36.9667630243843 | 32.5313360934747 | 51.8941236643882 | 32.2785253694205 | 43.790109545502 |
| YBL075C | 373.891085880126 | -0.267692424460543 | 0.289955877396209 | -0.923217790459741 | 0.355893719506862 | 0.670054732959529 | 409.32497987543 | 282.179624419467 | 325.313360934747 | 418.266636734969 | 410.716409010902 | 397.545504305244 |
| YBL076C | 15117.663579257 | 0.518139286652759 | 0.310965460079784 | 1.66622777500698 | 0.0956680558601587 | 0.355692099019529 | 23956.6747312547 | 14166.8958163782 | 15285.97434823 | 13204.9787076402 | 12185.6998532557 | 11905.758018783 |
| YBL077W | 1.54730359466496 | 0.966303555812569 | 1.91782859136647 | 0.503852930424856 | 0.614364729277008 | NA | 0 | 3.69667630243843 | 2.50241046872882 | 0 | 2.22610519789107 | 0.858629598931413 |
| YBL078C | 136.88036913756 | -0.0789839185099287 | 0.366753512230344 | -0.215359678574317 | 0.829486904233904 | 0.939300773352341 | 96.7495406978288 | 134.31257232193 | 168.912706639196 | 145.303546260287 | 166.95788984183 | 109.045959064289 |
| YBL079W | 1184.97821006353 | 0.507708654643194 | 0.279241442447673 | 1.8181708638693 | 0.0690380217320999 | 0.300857125789318 | 1639.32963014573 | 1365.30578103393 | 1168.62568889636 | 1086.66294953229 | 926.059762322685 | 923.8854484502 |
| YBL080C | 74.0974572283909 | 0.48908467768765 | 0.777092702281548 | 0.629377519891379 | 0.529101931615142 | 0.792240662757681 | 211.766477191751 | 20.9478323804844 | 26.2753099216526 | 65.3865958171291 | 30.0524201715294 | 90.1561078877983 |
| YBL081W | 468.722958173151 | 0.0211367031627361 | 0.292176262838327 | 0.0723423010391226 | 0.94232950150248 | 0.983779795011854 | 366.024486136541 | 542.179191024303 | 509.240530386315 | 466.009230506206 | 476.386512348689 | 452.497798636854 |
| YBL082C | 2242.84441191575 | 1.11711736513307 | 0.385757672617191 | 2.89590446135247 | 0.00378067481497288 | 0.0587241093105589 | 1582.49773211344 | 3867.95563778474 | 3761.12293449942 | 1483.13405432821 | 1367.94164410406 | 1394.41446866461 |
| YBL083C | 57.7425349225124 | 0.448997508429881 | 0.463852027079288 | 0.967975738420417 | 0.333056486615239 | 0.647455782833059 | 36.5347915921871 | 75.1657514829147 | 88.8355716398732 | 41.5152989315106 | 62.3309455409499 | 42.0728503476392 |
| YBL084C | 236.230785722665 | 0.115948907706517 | 0.279610494857951 | 0.414680099062167 | 0.678376102757821 | 0.87424576384305 | 253.713830501299 | 218.103901843867 | 265.255509685255 | 221.068966810294 | 224.836624986998 | 234.405880508276 |
| YBL085W | 71.3614864302529 | -0.216134604237142 | 0.548619196080484 | -0.393961067679145 | 0.69360978306372 | 0.882501670628042 | 127.195200357985 | 30.8056358536536 | 38.7873622652968 | 74.727538076719 | 76.8006293272419 | 79.8525527006214 |
| YBL086C | 12.0300136814357 | -0.731269982525 | 0.771271627402531 | -0.948135464269252 | 0.343060496012869 | 0.65555576166289 | 17.5908255814234 | 4.92890173658457 | 3.75361570309323 | 15.5682370993165 | 20.0349467810196 | 10.303555187177 |
| YBL087C | 73926.8644371805 | -0.0664876337419928 | 0.388463116189681 | -0.171155589735649 | 0.864101426398945 | 0.953608271002311 | 36422.4809365519 | 88547.7196977418 | 91702.0828318021 | 73247.5176698106 | 76009.2489293916 | 77632.1365577847 |
| YBL088C | 503.94722110701 | 0.485107026948795 | 0.317329120468933 | 1.52871890935168 | 0.126334142520309 | 0.40681717154439 | 481.041422630464 | 712.22630093647 | 570.549586870172 | 431.75910888771 | 497.534511728654 | 330.572395588594 |
| YBL089W | 1288.61926061643 | 0.453764765516683 | 0.372530862592059 | 1.2180595249462 | 0.223201353391644 | 0.546316906244176 | 824.739091682891 | 1768.24349799971 | 1876.80785154662 | 1099.11753921174 | 1175.38354448648 | 987.424038771124 |
| YBL090W | 443.501277231716 | -1.25120041316009 | 0.371059727123384 | -3.37196500105237 | 0.000746339319934366 | 0.0213717267707159 | 378.202750000604 | 198.388294897529 | 208.951274138857 | 692.267609682938 | 480.838722744471 | 702.359011925896 |
| YBL091C | 2246.82765494882 | 0.503913779533831 | 0.301234517805286 | 1.67282880861467 | 0.094361001823569 | 0.353346604874186 | 3372.02594991594 | 2199.52239995086 | 2333.49776208963 | 1652.30889747412 | 1835.42373566119 | 2088.1871846012 |
| YBL091C-A | 154.904029934603 | -0.65477520032424 | 0.310706319932992 | -2.107376510608 | 0.0350849560447662 | 0.210071174318038 | 138.020323792707 | 117.061416243884 | 105.101239686611 | 174.364255512344 | 213.706098997543 | 181.170845374528 |
| YBL092W | 45432.9944272127 | -0.509418777999095 | 0.369716439747309 | -1.37786347382137 | 0.168245444145557 | 0.472860254906382 | 56971.2714965131 | 26268.5818051275 | 29240.6663270963 | 51764.3883552272 | 48325.4046884183 | 60027.653890894 |
| YBL093C | 216.460873079726 | 0.504072943392566 | 0.604631208523383 | 0.83368661141988 | 0.404457604672817 | 0.710086228362661 | 552.081295170828 | 101.042485599984 | 107.603650155339 | 180.591550352071 | 151.375153456593 | 206.071103743539 |
| YBL094C | 19.1557747972092 | 0.445741967310056 | 0.561743235845834 | 0.793497702983266 | 0.427487907063113 | 0.72698239343159 | 15.561114937413 | 24.6445086829228 | 26.2753099216526 | 12.4545896794532 | 22.2610519789107 | 13.7380735829026 |
| YBL095W | 1090.5308335644 | 0.54970878174068 | 0.379393397288272 | 1.44891499343358 | 0.147361323452579 | 0.440892991564844 | 693.484470036885 | 1576.01633027292 | 1619.05957326755 | 846.912098202815 | 861.502711583844 | 946.209818022417 |
| YBL096C | 20.4086961539334 | 0.620715880022339 | 0.575266974036419 | 1.07900489344456 | 0.280585546220196 | 0.605862404084782 | 16.2376851520832 | 23.4122832487767 | 35.0337465622035 | 19.7197669924675 | 10.0174733905098 | 18.0312215775597 |
| YBL097W | 22.5868086466631 | 0.142293693878903 | 0.493036748827256 | 0.288606669213531 | 0.772882391296154 | 0.919042235736281 | 21.6502468694442 | 28.3411849853613 | 21.270488984195 | 23.8712968856186 | 18.9218941820741 | 21.4657399732853 |
| YBL098W | 1612.33226967037 | -0.289841366273952 | 0.288481236333456 | -1.00471479517276 | 0.315034201873441 | 0.635421652543671 | 1157.6116373006 | 1426.91705274123 | 1769.20420139128 | 1927.34775289538 | 1737.47510695398 | 1655.43786673976 |
| YBL099W | 26389.6076283071 | 0.72462908850215 | 0.334860037617964 | 2.16397601116221 | 0.0304661905258564 | 0.195446052614534 | 20557.585972752 | 38893.9636033888 | 39192.7527612308 | 20706.7932245642 | 19606.4215304256 | 19380.1286774809 |
| YBL100W-B | 1.83860659803107 | -2.22741468235815 | 1.67824261613228 | -1.32723043792768 | 0.184432466561397 | NA | 0.676570214670132 | 0 | 1.25120523436441 | 2.07576494657553 | 4.45221039578214 | 2.57588879679424 |
| YBL100W-C | 3.16801093272871 | -0.89093391216778 | 1.20458767219303 | -0.73961732527594 | 0.459532226024854 | NA | 0.676570214670132 | 2.46445086829228 | 3.75361570309323 | 3.11364741986329 | 5.56526299472767 | 3.43451839572565 |
| YBL101C | 227.841056882569 | -0.0368163630293176 | 0.423794733613845 | -0.0868731017853187 | 0.930772380334444 | 0.980512845043758 | 359.25878398984 | 162.653757307291 | 151.395833358094 | 203.424964764402 | 289.393675725839 | 200.919326149951 |
| YBL102W | 2725.43270236406 | 0.315434956868421 | 0.372833130369128 | 0.846048623833727 | 0.397525619594952 | 0.70393630974485 | 1642.71248121908 | 3792.78988630183 | 3632.24879535989 | 2572.91065128037 | 2312.92330060882 | 2399.01109941437 |
| YBL103C | 73.2056887296977 | 0.398627925593172 | 0.55803620967982 | 0.714340608509776 | 0.47501658741806 | 0.76034401127235 | 163.053421735502 | 41.8956647609688 | 43.7921832027544 | 65.3865958171291 | 58.9917877441133 | 66.1144791177188 |
| YBL104C | 226.462517624169 | 0.244564703309918 | 0.58097819689132 | 0.420953324270217 | 0.673789165665188 | 0.871790963764147 | 520.959065296001 | 113.364739941445 | 101.347623983517 | 206.538612184265 | 195.897257414414 | 220.667806925373 |
| YBL105C | 664.300159751302 | 0.211819543151026 | 0.276117400868064 | 0.767135799790606 | 0.44300077878646 | 0.738636525059224 | 596.734929339056 | 798.4820813267 | 744.467114446825 | 563.570182995256 | 660.040191174702 | 622.506459225274 |
| YBL106C | 430.93430943719 | 0.372444797721093 | 0.319461445514752 | 1.16585210187404 | 0.243674247564247 | 0.567946943988665 | 632.593150716573 | 361.04205220482 | 464.197141949197 | 373.637690383595 | 361.742094657299 | 392.393726711656 |
| YBL107C | 121.349741372388 | -0.481271658791633 | 0.47372374945101 | -1.01593314531807 | 0.309661223766181 | 0.633323413761106 | 171.172264311543 | 78.8624277853531 | 52.5506198433053 | 147.379311206862 | 112.418312493499 | 165.715512593763 |
| YBL109W | 5.22946977444787 | -1.27071081299603 | 0.937862495015895 | -1.35490097935359 | 0.175449107597572 | NA | 4.05942128802079 | 2.46445086829228 | 2.50241046872882 | 6.22729483972658 | 6.67831559367321 | 9.44492558824554 |
| YBL113C | 129.660171650425 | 0.677852801353614 | 0.338103726949121 | 2.00486639845771 | 0.0449773317770822 | 0.237910811254985 | 196.205362254338 | 126.919219717053 | 155.149449061187 | 89.2578927027477 | 97.948628707207 | 112.480477460015 |
| YBL113W-A | 2.41261344148392 | 1.83096218564093 | 1.51020489081085 | 1.21239323007215 | 0.225361891151604 | NA | 1.35314042934026 | 4.92890173658457 | 5.00482093745765 | 2.07576494657553 | 1.11305259894553 | 0 |
| YBR001C | 609.267076397399 | 0.37482794799148 | 0.276671359486728 | 1.35477683229247 | 0.175488670080056 | 0.484257741536325 | 576.437822898952 | 708.529624634032 | 779.500861009028 | 540.736768582925 | 560.97850986855 | 489.418871390905 |
| YBR002C | 758.286169739812 | 0.616186440392953 | 0.339988820862917 | 1.81237265045664 | 0.0699286358127792 | 0.301839866639432 | 598.764639983067 | 985.780347316914 | 1169.87689413072 | 658.017488064442 | 535.378300092802 | 601.89934885092 |
| YBR003W | 1626.49557117799 | -0.376736995432432 | 0.257151691704304 | -1.46503798180584 | 0.142910555440359 | 0.436245285539661 | 1603.47140876821 | 1276.5855497754 | 1365.06491069157 | 1920.08257558236 | 1759.73615893289 | 1834.0328233175 |
| YBR004C | 3461.32533951876 | 0.490966062887916 | 0.411647094200251 | 1.1926868179205 | 0.232992050148498 | 0.558714353046961 | 2236.74112969946 | 5390.98627438937 | 4506.84125418061 | 2282.30355875979 | 3995.85883021447 | 2355.22098986886 |
| YBR005W | 5282.09673735121 | 0.761214408739683 | 0.415086615362152 | 1.83386883741251 | 0.0666734663662151 | 0.29570917027238 | 3694.07337209892 | 8804.25072697419 | 7434.66150259333 | 2711.98690270093 | 5383.83542109955 | 3663.77249864034 |
| YBR006W | 1821.83428470726 | 0.45502297823022 | 0.36506838119512 | 1.24640478789375 | 0.212615824376429 | 0.532130859454992 | 1418.0911699486 | 2566.72557932641 | 2336.00017255836 | 1223.66343600627 | 2081.40836002815 | 1305.11699037575 |
| YBR007C | 148.657751955617 | -0.168678511024758 | 0.319720963082341 | -0.527580391972347 | 0.597790605284788 | 0.828750365770883 | 168.465983452863 | 126.919219717053 | 123.869318202077 | 162.947548306179 | 138.018522269246 | 171.725919786283 |
| YBR008C | 1081.64980106768 | 0.812199897654629 | 0.366575730445869 | 2.21564012616641 | 0.0267161584546157 | 0.18281485571087 | 784.821449017353 | 1693.0777465168 | 1657.84693553285 | 771.146677652808 | 862.615764182789 | 720.390233503455 |
| YBR009C | 11320.6531452292 | -0.257736290266858 | 0.404119635351325 | -0.637772252869606 | 0.523621944201445 | 0.78996822448029 | 4958.5831033174 | 13383.2004402613 | 12595.8830943465 | 13841.2006637656 | 11741.5918662764 | 11403.4597034081 |
| YBR010W | 23778.4463533257 | -0.535923940910684 | 0.320934143208381 | -1.6698875836427 | 0.0949416073758815 | 0.353927182029034 | 13130.8747263179 | 21000.8180741527 | 24105.7200452647 | 30950.6932359144 | 27140.6745726879 | 26341.8974656168 |
| YBR011C | 28606.8171380666 | -0.13625878289302 | 0.369661597089285 | -0.368604107015503 | 0.712422833867205 | 0.891399851461647 | 41372.9451972932 | 19014.4706743091 | 21381.8462500534 | 28319.6611661299 | 27651.5657156039 | 33900.41382501 |
| YBR014C | 477.116382990995 | -0.622070045237286 | 0.329364043829824 | -1.88870053331838 | 0.0589319671328769 | 0.275670539211498 | 479.688282201123 | 330.236416351166 | 316.554924294196 | 611.312776766493 | 447.447144776105 | 677.458753556885 |
| YBR015C | 6467.65038930648 | 0.0248485571786616 | 0.272854599808763 | 0.0910688593708054 | 0.92743787641652 | 0.980139995010912 | 7366.4964973284 | 5299.80159226256 | 6902.89927798846 | 6626.87959194237 | 6765.13369639096 | 5844.69167992613 |
| YBR016W | 397.577126722936 | 0.31610433075404 | 0.402403354382344 | 0.785540993412528 | 0.432136445228163 | 0.731159519481642 | 707.015874330288 | 311.753034838974 | 302.791666716188 | 332.122391452084 | 372.872620646754 | 358.90717235333 |
| YBR017C | 4860.14843065981 | 0.797206095686059 | 0.298981263216148 | 2.66640821271038 | 0.00766665384088543 | 0.0893653692943858 | 4541.13928086592 | 6461.79017666237 | 7507.23140618647 | 3774.7785553476 | 3546.18558024047 | 3329.76558465602 |
| YBR018C | 76.5836916941195 | 1.14041072076787 | 0.417281747808106 | 2.73295136141997 | 0.00627696009185734 | 0.0808967861165829 | 74.4227236137145 | 139.241474058514 | 102.598829217882 | 39.439533984935 | 63.4439981398955 | 40.3555911497764 |
| YBR019C | 77.8322255833792 | -0.856864102884106 | 0.450392976735714 | -1.90248104909261 | 0.057108294064516 | 0.270749921086218 | 27.0628085868053 | 72.7013006146224 | 67.5650826556782 | 102.750364855489 | 110.192207295608 | 86.7215894920727 |
| YBR020W | 168.024181418394 | 0.491629614919362 | 0.36125963252632 | 1.3608761418522 | 0.173552834324059 | 0.482212867295063 | 124.488919499304 | 234.122832487767 | 231.472968357416 | 137.000486473985 | 140.244627467137 | 140.815254224752 |
| YBR021W | 1883.6440125199 | 0.0941943316891769 | 0.36087999325769 | 0.261012894726797 | 0.794082563299741 | 0.929585587537483 | 1139.3442415045 | 2179.80679300453 | 2517.4249315412 | 2025.94658785771 | 1655.10921463201 | 1784.23230657948 |
| YBR022W | 398.18090480684 | -0.592329370914181 | 0.271566921189426 | -2.18115434795909 | 0.0291720021548336 | 0.191304294304882 | 293.631473166837 | 317.914162009705 | 341.579028981484 | 533.47159126991 | 458.57767076556 | 443.91150264754 |
| YBR023C | 4272.73295214878 | 0.515017118658509 | 0.310528100508608 | 1.65852017197469 | 0.0972125194315935 | 0.357503238447089 | 3482.30689490717 | 5547.47890452593 | 6053.33092385502 | 3658.53571833937 | 3519.47231786578 | 3375.27295339938 |
| YBR024W | 302.518659986472 | -0.152442892181563 | 0.437448148918418 | -0.348482197395223 | 0.727478081374417 | 0.899022777228632 | 455.331754472999 | 216.871676409721 | 186.429579920297 | 329.008744032221 | 217.045256794379 | 410.424948289215 |
| YBR025C | 23828.3817749312 | 0.590947872291318 | 0.315442524333281 | 1.87339317531885 | 0.0610141117380966 | 0.281467988981203 | 19669.2492808901 | 32025.5390334582 | 34230.4728017416 | 20136.9957467292 | 19859.0844703862 | 17048.9493163821 |
| YBR026C | 807.380808179007 | 0.0797682508824339 | 0.559249967468269 | 0.142634341569202 | 0.886578974120759 | 0.962704561588727 | 1724.57747719417 | 402.937716965789 | 360.34710749695 | 789.828562171988 | 655.58798077892 | 911.006004466229 |
| YBR027C | 43.5834451855037 | 0.0267102103825061 | 0.450691520817143 | 0.0592649498576725 | 0.952741077985106 | 0.986211737518731 | 27.0628085868053 | 49.2890173658457 | 56.3042355463985 | 38.4016515116473 | 50.0873669525491 | 40.3555911497764 |
| YBR028C | 338.331932285053 | 0.00588110802770814 | 0.299782710572824 | 0.0196179026351137 | 0.984348182343793 | 0.995606783760712 | 399.852996870048 | 287.108526156051 | 329.06697663784 | 367.410395543868 | 360.629042058353 | 285.92365644416 |
| YBR029C | 4516.25709890767 | 0.400995662486654 | 0.328423961328379 | 1.22096956892166 | 0.222097541939591 | 0.544612629596031 | 3371.34937970127 | 5920.84321107221 | 6128.40323791689 | 4201.34825186887 | 4005.87630360498 | 3469.72220928184 |
| YBR030W | 277.785642645229 | 0.5001702163008 | 0.715069315799683 | 0.699470953723479 | NA | NA | 799.029423525426 | 76.3979769170608 | 100.096418749153 | 229.372026596596 | 194.784204815469 | 267.033805267669 |
| YBR031W | 69036.9875092949 | 0.734059347209173 | 0.36488504505161 | 2.01175509153944 | 0.044245760040534 | 0.236141716539452 | 47886.2866539226 | 102373.289068862 | 108434.450430957 | 52254.268882619 | 50933.2869277477 | 52340.343091661 |
| YBR032W | 28.7645441980517 | -1.10593631764732 | 0.619627110873196 | -1.78484171889897 | 0.0742869942494029 | 0.31168003134713 | 10.8251234347221 | 22.1800578146306 | 22.5216942185594 | 34.2501216184962 | 16.695788984183 | 66.1144791177188 |
| YBR033W | 42.3550991905824 | -0.128062619277014 | 0.415410228874466 | -0.308279889072527 | 0.757869367540188 | 0.912866087924729 | 46.6833448122391 | 34.502312156092 | 40.0385674996612 | 47.7425937712371 | 34.5046305673116 | 50.6591463369533 |
| YBR034C | 7720.4074968822 | 0.314242219534224 | 0.277015755799619 | 1.13438392205219 | 0.256633540259341 | 0.582364622744056 | 6729.8439253238 | 9396.95116079848 | 9547.94714343482 | 7288.01072742668 | 6514.69686162821 | 6844.99516268122 |
| YBR035C | 2716.52018050289 | -0.60034167910814 | 0.309078253663105 | -1.94236143110383 | 0.0520933609896656 | 0.259145279251368 | 2842.27147182922 | 1656.11098349242 | 1978.15547553013 | 3218.47354966536 | 3093.17317246964 | 3510.93643003055 |
| YBR036C | 8771.10103326561 | 0.710234235538434 | 0.361725958510308 | 1.96345940574291 | 0.0495928169359266 | 0.250917806760842 | 6264.36361763075 | 13495.3329547686 | 12903.6795820002 | 6270.88590360467 | 7504.20062209079 | 6188.14351949869 |
| YBR037C | 1282.98467633085 | -0.0809792689839497 | 0.361237086554124 | -0.224172079772924 | 0.822623407748662 | 0.936610490543666 | 714.458146691659 | 1468.8127175022 | 1559.00172201806 | 1262.06508751792 | 1371.2808019009 | 1322.28958235438 |
| YBR038W | 2213.5209892037 | 0.0579596707276881 | 0.271996410629001 | 0.213089836713854 | 0.831256891731776 | 0.939639058736332 | 1931.60796288323 | 2214.30910516062 | 2628.78219739963 | 2397.50851329473 | 2011.28604629458 | 2097.63211018944 |
| YBR039W | 2131.61946748377 | -0.240485042402643 | 0.460923224004182 | -0.52174642083225 | 0.6018468990832 | 0.830907968110565 | 3480.27718426316 | 1360.37687929734 | 1020.98347124136 | 2325.89462263788 | 1986.79888911778 | 2615.38575834508 |
| YBR040W | 25.7242289875263 | -0.340420082603883 | 0.477765901138743 | -0.712524861637258 | 0.476139821760381 | 0.760668954287083 | 17.5908255814234 | 24.6445086829228 | 26.2753099216526 | 31.1364741986329 | 28.9393675725839 | 25.7588879679424 |
| YBR041W | 3048.31893048064 | 0.412011421003267 | 0.294853913789483 | 1.39734085842059 | 0.162311094645621 | 0.463699886691368 | 2638.62383721351 | 3800.1832389067 | 4003.85674996612 | 2681.88831097558 | 2861.65823188897 | 2303.70321393298 |
| YBR042C | 1906.19445505411 | 0.365403458683304 | 0.322616213688695 | 1.13262583583569 | 0.25737141431088 | 0.58288845132346 | 1466.12765519018 | 2263.59812252646 | 2710.11053763332 | 1732.22584791728 | 1753.05784333922 | 1512.04672371822 |
| YBR043C | 965.818291790285 | -0.243970291005492 | 0.252931322634779 | -0.964571285454326 | 0.334759587494309 | 0.64984738565258 | 805.795125672127 | 901.989017794976 | 945.911157179495 | 1089.77659695215 | 996.182076056254 | 1055.25577708671 |
| YBR044C | 254.266905118888 | -0.273413645074529 | 0.527361242937761 | -0.518456084393743 | 0.604140092800389 | 0.831560644975248 | 454.655184258329 | 123.222543414614 | 111.357265858433 | 271.925208001394 | 279.376202335329 | 285.065026845229 |
| YBR045C | 30.9036397251312 | 0.377183420264895 | 0.493751530377095 | 0.763913420130217 | 0.444918839471284 | 0.738826432810841 | 46.006774597569 | 23.4122832487767 | 35.0337465622035 | 31.1364741986329 | 18.9218941820741 | 30.9106655615309 |
| YBR046C | 305.215042646114 | -0.0140436116463828 | 0.34476071804433 | -0.0407343728892484 | 0.967507658724212 | 0.989895696394939 | 209.060196333071 | 338.861994390189 | 364.100723200044 | 278.152502841121 | 370.646515448863 | 270.468323663395 |
| YBR047W | 46.0103415315258 | -0.927270905477706 | 0.427392092990789 | -2.16960238779544 | 0.0300369803430895 | 0.193912357894162 | 27.0628085868053 | 33.2700867219458 | 35.0337465622035 | 68.5002432369924 | 70.1223137335687 | 42.0728503476392 |
| YBR048W | 47995.6988140536 | -0.224354590745307 | 0.239736537476107 | -0.935838120910825 | 0.349356540837812 | 0.661865984421801 | 42987.9182997108 | 42424.2894722175 | 47401.9103038957 | 50059.1474516154 | 51429.7083868774 | 53671.2189700047 |
| YBR049C | 119.496411824895 | 0.7612824012072 | 0.357192904963509 | 2.13129205711679 | 0.0330650890238189 | 0.204884291413191 | 161.023711091491 | 142.938150360953 | 146.391012420636 | 111.053424641791 | 94.6094709103704 | 60.9627015241303 |
| YBR050C | 21.5884418672404 | -0.347408086617502 | 0.5378099684516 | -0.645968105830612 | 0.518300010963447 | 0.78502989802843 | 24.3565277281247 | 19.7156069463383 | 12.5120523436441 | 17.644002045892 | 27.8263149736384 | 27.4761471658052 |
| YBR051W | 3.62829697726828 | -1.07234598114154 | 1.15554932395616 | -0.92799671888539 | 0.353409271614569 | NA | 2.0297106440104 | 2.46445086829228 | 2.50241046872882 | 4.15152989315105 | 8.90442079156428 | 1.71725919786283 |
| YBR052C | 3869.30682795634 | -0.323202493174438 | 0.281851140440939 | -1.14671344834301 | 0.251500070491895 | 0.575146686152046 | 2732.66709705266 | 3625.20722725795 | 3956.31095106027 | 3869.22586041678 | 4682.61228376386 | 4349.81754818654 |
| YBR053C | 1732.39224835585 | 0.00765101177153683 | 0.364507759196093 | 0.0209899832815928 | 0.983253646100222 | 0.995375476890468 | 996.587926209104 | 2098.47991435088 | 2117.03925654458 | 1604.56630370288 | 1952.29425855047 | 1625.38583077716 |
| YBR054W | 7627.00240431758 | 0.56129697534967 | 0.351283721496466 | 1.5978451063959 | 0.110077453279781 | 0.382080435659531 | 5982.91040832798 | 10472.6839648081 | 10821.6740720178 | 5661.64889178475 | 7825.87282318605 | 4997.22426578082 |
| YBR055C | 734.649940072476 | 0.339154669731439 | 0.268483379827952 | 1.26322407721764 | 0.206508662312849 | 0.524108379826666 | 698.897031754246 | 840.377746087669 | 923.389462960936 | 638.297721071975 | 627.761665805281 | 679.176012754747 |
| YBR056C-B | 3.99344035436252 | -0.589410222362069 | 1.02994834814018 | -0.572271632287572 | 0.567137965000313 | NA | 3.38285107335066 | 2.46445086829228 | 3.75361570309323 | 4.15152989315105 | 3.3391577968366 | 6.8690367914513 |
| YBR056W | 1510.55115735048 | 0.206098018083035 | 0.272020134534961 | 0.757657216938646 | 0.448656217456826 | 0.742035731635397 | 1848.3898264788 | 1536.58511638024 | 1468.91494514382 | 1267.25449988436 | 1606.13490027841 | 1336.02765593728 |
| YBR056W-A | 1215.58220651071 | 0.88895189010698 | 0.407943759865542 | 2.17910402747668 | 0.0293239411450245 | 0.191538651933637 | 888.336691861883 | 1990.04407614602 | 1858.03977303115 | 663.206900430881 | 1182.06186008016 | 711.803937514141 |
| YBR057C | 18.2139201107571 | -0.51072152074689 | 0.648344489645507 | -0.787731721181336 | 0.430853646759662 | 0.730544767426118 | 25.709668157465 | 6.16112717073071 | 12.5120523436441 | 17.644002045892 | 18.9218941820741 | 28.3347767647366 |
| YBR058C | 1280.4621764144 | 0.0550081016459895 | 0.408377161622849 | 0.13469926042728 | 0.892849657954835 | 0.965832153362604 | 2132.54931664026 | 959.903613199845 | 820.790633743054 | 1230.92861331929 | 1155.34859770546 | 1383.25228387851 |
| YBR058C-A | 1479.71473620027 | -0.892690429675989 | 0.363397749387726 | -2.45651061730582 | 0.0140293656457687 | 0.128088851068012 | 602.147491056417 | 1301.23005845833 | 1206.16184592729 | 2196.15931347691 | 1779.77110571391 | 1792.81860256879 |
| YBR059C | 148.776311747544 | -0.0311075650887234 | 0.308617544599592 | -0.100796489483717 | 0.919712013635419 | 0.977974644292995 | 148.168877012759 | 129.383670585345 | 163.907885701738 | 171.250608092481 | 139.131574868192 | 140.815254224752 |
| YBR060C | 302.637640916339 | -0.177524612686972 | 0.347603311563648 | -0.510710360866245 | 0.609553884689827 | 0.836009479646177 | 401.206137299388 | 220.568352712159 | 228.970557888687 | 302.023799726739 | 323.898306293151 | 339.158691577908 |
| YBR061C | 1449.31294683357 | 0.152924216044951 | 0.257647161395963 | 0.59354124150404 | 0.552818991131187 | 0.803846081715676 | 1712.3992133301 | 1462.65159033147 | 1402.60106772251 | 1385.57310183916 | 1275.55827839158 | 1457.09442938661 |
| YBR062C | 360.572198884869 | -0.868314463645438 | 0.295378512192056 | -2.93966699609096 | 0.00328565160195491 | 0.0533608949011962 | 311.222298748261 | 224.265029014598 | 228.970557888687 | 499.221469651414 | 424.073040198249 | 475.680797808003 |
| YBR063C | 175.095336883949 | -0.0950612855714465 | 0.36819090327691 | -0.258184775140825 | 0.796264307377377 | 0.929585587537483 | 234.093294275866 | 151.563728399976 | 121.366907733348 | 182.667315298646 | 151.375153456593 | 209.505622139265 |
| YBR065C | 22.2807940397192 | 0.712861283732794 | 0.757390667187722 | 0.941206849537411 | 0.346598867302569 | 0.658556137932609 | 58.8616086763015 | 6.16112717073071 | 17.5168732811018 | 13.4924721527409 | 14.469683786292 | 23.1829991711481 |
| YBR066C | 16.9855578521546 | 0.164962885219298 | 0.604417485155506 | 0.272928711148811 | 0.784908015773063 | 0.925243921158455 | 28.4159490161455 | 13.5544797756076 | 11.2608471092797 | 17.644002045892 | 15.5827363852375 | 15.4553327807654 |
| YBR067C | 23978.4777213367 | 0.405060022584939 | 0.391622481847054 | 1.03431248552052 | 0.300990101813993 | 0.625627510682729 | 14239.0967379476 | 33241.7455369605 | 34488.2210800206 | 17552.6683882427 | 24371.3997065114 | 19977.7348783372 |
| YBR068C | 16210.4420845259 | 0.722643530637454 | 0.360425169541696 | 2.00497521179318 | 0.0449656971141411 | 0.237910811254985 | 11890.0449526129 | 24850.2903304253 | 23822.9476622984 | 11459.2603875702 | 14337.2305270174 | 10902.8786472311 |
| YBR069C | 6275.95587274761 | 0.628128636708233 | 0.268309739249731 | 2.34105790741945 | 0.0192291843614848 | 0.152937438798529 | 6187.91118337302 | 8460.45983084741 | 8215.41356883673 | 5168.65471697306 | 4818.40470083522 | 4804.89123562018 |
| YBR070C | 1585.17875671428 | 0.416047898809146 | 0.380628325463312 | 1.09305553732167 | 0.27436941619019 | 0.600152316299675 | 958.699994187577 | 2135.44667737526 | 2343.50740396454 | 1305.65615139601 | 1369.05469670301 | 1398.70761665927 |
| YBR071W | 1199.73402866186 | -0.0986872054522956 | 0.311105483457943 | -0.317214612726801 | 0.751080762720673 | 0.908603445448746 | 1349.08100805224 | 1118.8606942047 | 1007.22021366335 | 1024.39000113502 | 1597.23047948684 | 1101.621775429 |
| YBR072W | 1740.20269995496 | -1.02188002303282 | 0.268880197372666 | -3.80050309772907 | 0.000144402612809849 | 0.00709424118317104 | 970.878258051639 | 1176.77528960957 | 1298.75103327026 | 2145.30307228581 | 2516.61192621585 | 2332.89662029665 |
| YBR073W | 557.308503562664 | 0.134137859429438 | 0.446630289332697 | 0.300333100179684 | 0.763923087621801 | 0.914988520035447 | 1016.20846243454 | 400.473266097496 | 331.569387106569 | 548.001945895939 | 488.63009093709 | 558.96786890435 |
| YBR074W | 1806.43775372776 | -0.0722453931901014 | 0.294305548808561 | -0.245477509624174 | 0.806086720244083 | 0.930865338863581 | 2263.80393828626 | 1508.24393139488 | 1510.20471787784 | 1928.38563536866 | 1864.36310323377 | 1763.62519620512 |
| YBR076C-A | 517.887301025166 | -0.107184402818825 | 0.366276966367037 | -0.292632113566809 | 0.769803364668711 | 0.917443446011974 | 292.278332737497 | 574.217052312102 | 630.607438119663 | 534.509473743198 | 596.596193034807 | 479.115316203728 |
| YBR076W | 26.9286902694957 | -1.01464881813129 | 0.482148054407189 | -2.10443412320479 | 0.0353405954446424 | 0.211161894611024 | 14.2079745080728 | 16.0189306438998 | 23.7728994529238 | 35.288004091784 | 34.5046305673116 | 37.7797023529822 |
| YBR077C | 646.175601289531 | -0.131330123318247 | 0.259655645967967 | -0.505785741067418 | 0.613007069472607 | 0.8378853783975 | 560.200137746869 | 605.022688165756 | 685.660468431697 | 661.131135484305 | 697.88397953885 | 667.155198369708 |
| YBR078W | 90525.1496170785 | 0.766578898314535 | 0.367591720576126 | 2.08540849917152 | 0.0370322360134681 | 0.21720540061777 | 62969.0664495638 | 142096.540389431 | 137010.726778606 | 68001.021767341 | 70475.1514074344 | 62598.3909100946 |
| YBR079C | 574.303887406014 | -0.000381401531581659 | 0.379148821003815 | -0.00100594149435011 | 0.999197374947952 | 0.999545163801049 | 885.630411003203 | 417.724422175542 | 417.902548277713 | 581.214185041148 | 543.169668285421 | 600.182089653057 |
| YBR080C | 2823.59584408427 | 0.384755689360448 | 0.256593518710447 | 1.49947547893688 | 0.133750325409222 | 0.41896287218104 | 3329.40202639172 | 2991.84335410683 | 3271.90168786294 | 2369.48568651596 | 2774.84012917122 | 2204.10218045694 |
| YBR081C | 311.787042086678 | -0.371983310065256 | 0.362102984208794 | -1.02728595534238 | 0.304285833721689 | 0.629149270587147 | 399.176426655378 | 208.246098370698 | 206.448863670128 | 345.614863604825 | 350.611568667843 | 360.624431551193 |
| YBR082C | 2013.08156337871 | -0.454383401623458 | 0.548156981841206 | -0.828929333522722 | 0.407144395600587 | 0.711205913243079 | 3452.53780546168 | 784.927601551093 | 857.075585539622 | 2408.9252205009 | 1725.23152836558 | 2849.79163885336 |
| YBR083W | 87.8605992784092 | -1.22352295728894 | 0.409519039395666 | -2.98770713834089 | 0.00281078751745604 | 0.0491076189979858 | 75.7758640430548 | 38.1989884585304 | 42.54097796839 | 133.886839054122 | 138.018522269246 | 98.7424038771124 |
| YBR084C-A | 7185.35065999255 | 0.652646671142276 | 0.662602842349774 | 0.984974149564179 | 0.324636768860162 | 0.641084429827143 | 20607.6521686375 | 2595.06676431178 | 3146.7811644265 | 5881.67997612176 | 4035.92872377651 | 6844.99516268122 |
| YBR084W | 7336.35062084383 | -0.0398529993518112 | 0.521492397745236 | -0.0764210552716064 | 0.939084118902696 | 0.983188154154343 | 14243.8327294503 | 3557.43482837991 | 3902.5091259826 | 7724.95924868083 | 5809.02151389675 | 8780.34627867262 |
| YBR085C-A | 3537.45205304448 | -0.343299817641937 | 0.370026564085425 | -0.92777073584015 | 0.353526507089293 | 0.666908553577045 | 1845.00697540545 | 3685.58627353111 | 3826.18560668637 | 3394.91357012427 | 4928.59690813083 | 3544.42298438887 |
| YBR085W | 538.159536576265 | 1.13775900040027 | 0.348134592763207 | 3.26815841933337 | 0.00108249756021163 | 0.0270530259830281 | 464.12716726371 | 816.965462838892 | 939.655131007673 | 318.629919299343 | 357.289884261517 | 332.289654786457 |
| YBR086C | 1342.65435237268 | 1.2255125305912 | 0.678242077697371 | 1.80689545943804 | NA | NA | 4510.69362120577 | 543.411416458449 | 588.066460151273 | 807.47256421788 | 678.962085356776 | 927.319966845926 |
| YBR087W | 1505.94038687416 | -0.388932652657492 | 0.406579911753612 | -0.956595841097989 | 0.338771288800798 | 0.653222867503182 | 2119.69448256152 | 926.633526477899 | 864.582816945808 | 1837.05197771934 | 1453.64669422287 | 1834.0328233175 |
| YBR088C | 3494.03294232565 | -0.0763473207959451 | 0.265861363314097 | -0.287169673111719 | 0.773982406128061 | 0.919754159690737 | 2822.65093560379 | 3668.33511745307 | 3714.82834082794 | 3811.10444191267 | 3373.66242740392 | 3573.61639075254 |
| YBR089C-A | 1561.79280579583 | -0.642734748734312 | 0.273842513494745 | -2.347096294625 | 0.0189203642374671 | 0.15187843287378 | 1002.67705814114 | 1341.89349778515 | 1315.016701317 | 1909.70375084949 | 2063.59951844502 | 1737.86630823718 |
| YBR089W | 10.4889392594016 | 0.906110767573444 | 0.770199216737631 | 1.17646285257404 | 0.239409968307228 | NA | 5.41256171736105 | 18.4833815121921 | 17.5168732811018 | 9.34094225958987 | 4.45221039578214 | 7.72766639038271 |
| YBR090C | 30.9737432027711 | -0.708154177231017 | 0.444694311970762 | -1.59245161938922 | 0.111283265625138 | 0.383716983091359 | 25.709668157465 | 25.876734117069 | 18.7680785154662 | 38.4016515116473 | 36.7307357652026 | 40.3555911497764 |
| YBR091C | 938.848717206882 | -0.74117176048711 | 0.335767094177868 | -2.2073984417737 | 0.0272862351505742 | 0.184267290042603 | 455.331754472999 | 766.4442200389 | 888.355716398732 | 1257.91355762477 | 1093.01765216452 | 1172.02940254138 |
| YBR092C | 13647.1256584128 | -0.889292186465689 | 0.244949665389203 | -3.63050990517862 | 0.000282861848531321 | 0.0114499289109721 | 9897.54567040936 | 8716.76272114981 | 10093.4726256177 | 17752.9797055872 | 16813.7725596712 | 18608.2206680416 |
| YBR093C | 7673.64625946846 | 2.26097855083935 | 0.27826840420804 | 8.12517165674688 | 4.46729728255737e-16 | 6.41950619503494e-13 | 10153.9657817693 | 12928.5092550613 | 15011.9604019042 | 2785.67655830436 | 2676.89150046401 | 2484.87405930751 |
| YBR094W | 599.67750945727 | 0.0413847647621364 | 0.415180593303428 | 0.0996789479798518 | 0.920599214367713 | 0.978384783691908 | 991.851934706413 | 433.743352819442 | 397.883264527883 | 675.661490110334 | 495.308406530763 | 603.616608048783 |
| YBR095C | 54.1025928064776 | 0.830588443904823 | 0.489322737854994 | 1.69742458228246 | 0.0896164183205992 | 0.343639207809743 | 106.898093917881 | 65.3079480097455 | 35.0337465622035 | 37.3637690383595 | 34.5046305673116 | 45.5073687433649 |
| YBR096W | 3760.28827627202 | -0.210220996737572 | 0.330287921084679 | -0.636478003940311 | 0.524464913231686 | 0.790562676876584 | 2240.12398077281 | 3901.22572450669 | 4320.41167426031 | 4118.31765400585 | 4104.93798491113 | 3876.71263917533 |
| YBR097W | 584.0514884047 | -0.0558503940335778 | 0.300477974596491 | -0.185871840052765 | 0.852545265741359 | 0.948409171178891 | 432.328367174214 | 688.814017687694 | 598.076102026189 | 609.237011819917 | 617.744192414772 | 558.109239305418 |
| YBR098W | 10.5107766383465 | 0.402475027632112 | 0.669142665840858 | 0.601478650485325 | 0.547521227426997 | NA | 12.1782638640624 | 9.85780347316914 | 13.7632575780085 | 10.3788247328776 | 10.0174733905098 | 6.8690367914513 |
| YBR099C | 126.986672825061 | 0.123953604725936 | 0.373264846886638 | 0.332079502690436 | 0.739829227213334 | 0.90301928216742 | 86.6009874777769 | 147.867052097537 | 163.907885701738 | 134.924721527409 | 102.400839102989 | 126.218551042918 |
| YBR101C | 1383.96794630672 | -0.0985709577858317 | 0.362816849056256 | -0.271682415086925 | 0.785866223615116 | 0.925456921344373 | 1932.96110331257 | 961.135838633991 | 1114.82386381869 | 1584.84653671042 | 1135.31365092445 | 1574.72668444021 |
| YBR102C | 250.077979106463 | 0.340869809886664 | 0.445010834243651 | 0.765980923736414 | 0.443687652923124 | 0.738706888702244 | 474.952290698433 | 200.852745765821 | 161.405475233009 | 196.159787451387 | 240.419361372235 | 226.678214117893 |
| YBR103C-A | 5.50915886156663 | -0.152842922965116 | 0.901153299160229 | -0.16960812672777 | 0.865318330951569 | NA | 3.38285107335066 | 7.39335260487685 | 5.00482093745765 | 5.18941236643882 | 7.79136819261874 | 4.29314799465706 |
| YBR103W | 454.431917656382 | 0.22680398699894 | 0.349145151919569 | 0.649597984540217 | 0.515951935299493 | 0.783453722438315 | 698.897031754246 | 361.04205220482 | 409.144111637163 | 400.622634689077 | 401.811988219338 | 455.073687433649 |
| YBR104W | 2847.47255504824 | 0.377917896946041 | 0.329862049835894 | 1.14568467980495 | 0.251925645104647 | 0.575522774566069 | 2154.87613372437 | 3398.47774737506 | 4102.7019634809 | 2706.79749033449 | 2191.60056732376 | 2530.38142805087 |
| YBR105C | 680.481134287113 | -0.548134805741799 | 0.439875271394356 | -1.24611416323603 | 0.212722485837952 | 0.532130859454992 | 955.317143114226 | 373.364306546281 | 327.815771403476 | 729.631378721298 | 838.128607005988 | 858.629598931413 |
| YBR106W | 20333.1926278034 | -0.240165985476114 | 0.248738042165949 | -0.965537813937942 | 0.334275505514646 | 0.649346267555993 | 16832.3903707782 | 18459.9692289434 | 20642.3839565441 | 21556.8189701869 | 21398.4362147279 | 23109.15702564 |
| YBR107C | 98.7962092922775 | -0.00549298490046015 | 0.572788898316842 | -0.00958989414180592 | 0.992348488804601 | 0.998253257551426 | 197.558502683678 | 35.7345375902381 | 61.3090564838562 | 96.523070015762 | 104.62694430088 | 97.0251446792496 |
| YBR108W | 117.672879091309 | -0.238827006278122 | 0.547669095452591 | -0.436079027027728 | 0.662779393304797 | 0.866909384383846 | 208.383626118401 | 66.5401734438917 | 47.5457989058476 | 101.712482382201 | 162.505679446048 | 119.349514251466 |
| YBR109C | 6981.68622277936 | 0.0269436836443313 | 0.368464983499334 | 0.0731241362162708 | 0.941707335147389 | 0.983779795011854 | 3901.10385778798 | 8178.28020642795 | 9062.47951250143 | 6635.18265172867 | 7383.99094140468 | 6729.08016682548 |
| YBR109W-A | 9.67153731103646 | 0.730086272899943 | 0.783089379387491 | 0.932315380743631 | 0.351173561274008 | NA | 8.79541279071171 | 9.85780347316914 | 17.5168732811018 | 10.3788247328776 | 8.90442079156428 | 2.57588879679424 |
| YBR110W | 1693.8544310939 | 0.801628877544201 | 0.361028520555683 | 2.22040318673539 | 0.0263914109292086 | 0.18145673447499 | 1267.21601207716 | 2579.04783366788 | 2612.51652935289 | 1258.95144009806 | 1382.41132789035 | 1062.98344347709 |
| YBR111C | 1738.04440301467 | -0.155208927397159 | 0.278980947430165 | -0.556342391216558 | 0.577976813041688 | 0.817875608410542 | 1995.20556306222 | 1391.18251515099 | 1546.48966967441 | 1699.01360877207 | 1861.02394543693 | 1935.3511159914 |
| YBR111W-A | 68.1836435908052 | -0.629625954278002 | 0.433212376509898 | -1.45338865743051 | 0.146115873940947 | 0.440892991564844 | 76.4524342577249 | 41.8956647609688 | 41.2897727340256 | 98.5988349623375 | 58.9917877441133 | 91.8733670856611 |
| YBR112C | 142.478449390909 | -0.0384795514187792 | 0.351306652562396 | -0.109532657973065 | 0.91278002027304 | 0.975578199429051 | 190.792800536977 | 115.829190809737 | 113.859676327161 | 144.265663786999 | 153.601258654484 | 136.522106230095 |
| YBR114W | 495.631561697799 | 0.682923569171452 | 0.285486370452331 | 2.39214071091875 | 0.0167504191804321 | 0.141820699519406 | 575.084682469612 | 670.330636175501 | 586.815254916909 | 346.652746078113 | 457.464618166615 | 337.441432380045 |
| YBR115C | 5044.13658796609 | 0.629749735789834 | 0.268046738586647 | 2.34940271652014 | 0.0188035570719378 | 0.151376535083331 | 5238.68317219083 | 6076.10361577463 | 7069.30957415892 | 4276.07578994559 | 4006.98935620392 | 3597.65801952262 |
| YBR116C | 30.0221876087178 | -0.078704031691198 | 0.591840926418044 | -0.132981732384634 | 0.894207827621522 | 0.965913961099331 | 11.5016936493922 | 51.753468234138 | 25.0241046872882 | 33.2122391452084 | 31.165472770475 | 27.4761471658052 |
| YBR117C | 253.459167815972 | 0.408540120346512 | 0.420525089949668 | 0.971499989204949 | 0.331299358498343 | 0.646462981596975 | 141.403174866058 | 336.397543521897 | 390.376033121696 | 235.599321436322 | 231.514940580671 | 185.463993369185 |
| YBR118W | 115843.165061968 | 0.936725746367213 | 0.350116852429881 | 2.67546603331474 | 0.00746254347085133 | 0.0877610474214523 | 91196.2523758164 | 169231.376674763 | 196122.666870918 | 83539.1602749321 | 81586.7555027077 | 73382.7786726732 |
| YBR119W | 48.5269051010547 | -0.319542692161797 | 0.444514863879611 | -0.718857158955069 | 0.472228935094737 | 0.759052550034829 | 53.4490469589404 | 36.9667630243843 | 38.7873622652968 | 63.3108308705536 | 33.391577968366 | 65.2558495187874 |
| YBR120C | 85.23134398408 | -0.86267796506468 | 0.463982607165944 | -1.85928944693425 | 0.0629861220206064 | 0.286331033652128 | 88.6306981217873 | 49.2890173658457 | 42.54097796839 | 145.303546260287 | 64.557050738841 | 121.066773449329 |
| YBR121C | 25135.0458797374 | 0.604148918133936 | 0.272947817536479 | 2.2134227838374 | 0.0268685113139081 | 0.183062942450835 | 24561.5285031698 | 32771.0354211167 | 33634.8991101841 | 21507.000611469 | 20174.0783558878 | 18161.7332765972 |
| YBR122C | 1882.62024991046 | -0.63629662710888 | 0.248422454725542 | -2.56134908501675 | 0.0104266530201428 | 0.107332944080951 | 1421.47402102195 | 1413.36257296563 | 1587.77944240844 | 2270.88685155363 | 2155.9828841575 | 2446.23572735559 |
| YBR123C | 65.7196994275075 | 0.720016008536022 | 0.610747483826256 | 1.17890949631952 | 0.238434215926759 | 0.564457827907627 | 171.172264311543 | 36.9667630243843 | 36.2849517965679 | 52.932006137676 | 52.3134721504401 | 44.6487391444335 |
| YBR124W | 3.06112504576692 | 1.08436679316929 | 1.22086213539066 | 0.88819757918228 | 0.374434480746335 | NA | 2.70628085868053 | 7.39335260487685 | 2.50241046872882 | 2.07576494657553 | 1.11305259894553 | 2.57588879679424 |
| YBR125C | 841.7073304957 | -0.17160263820572 | 0.270055213242394 | -0.635435384288228 | 0.525144496577243 | 0.79083469891911 | 912.016649375338 | 674.02731247794 | 788.259297649579 | 928.904813592549 | 851.485238193334 | 895.550671685463 |
| YBR126C | 8941.19177193129 | 0.56015680451941 | 0.305739016164709 | 1.83214040375416 | 0.0669305051155476 | 0.296163620788428 | 9910.40050448809 | 11065.3843986324 | 10990.586778657 | 5730.14913502174 | 9555.55656194741 | 6395.07325284116 |
| YBR126W-A | 10.2616486663704 | 0.393413579342447 | 0.748261236374739 | 0.525770359625339 | 0.599047771043435 | NA | 10.148553220052 | 4.92890173658457 | 20.0192837498306 | 7.26517731301435 | 8.90442079156428 | 10.303555187177 |
| YBR126W-B | 430.470858889274 | 0.361415513736232 | 0.317874217431779 | 1.13697649547119 | 0.255548111649921 | 0.580818721140272 | 339.638247764406 | 555.73367079991 | 558.037534526527 | 386.092280063048 | 359.515989459408 | 383.807430722341 |
| YBR127C | 25978.6715798204 | 0.399527693738916 | 0.277123679912612 | 1.4416945309939 | 0.14938856714938 | 0.443994562551517 | 23926.2290715945 | 30066.3005931659 | 34667.1434285347 | 21912.8126585246 | 24494.9485449944 | 20804.5951821081 |
| YBR128C | 37.0248154560005 | -0.359229692354378 | 0.42230299058076 | -0.850644443366023 | 0.394966893352584 | 0.702080855414639 | 38.5645022361975 | 32.0378612877997 | 26.2753099216526 | 39.439533984935 | 41.1829461609848 | 44.6487391444335 |
| YBR129C | 581.200981766067 | -0.408620287079971 | 0.259238504032948 | -1.57623300830357 | 0.114972114052068 | 0.391385537902502 | 462.77402683437 | 485.49682105358 | 550.530303120341 | 691.229727209651 | 648.909665185247 | 648.265347193216 |
| YBR130C | 96.1130283448742 | -0.309773179775777 | 0.338182041131634 | -0.915995357823277 | 0.359669329709595 | 0.674072157538556 | 101.48553220052 | 80.0946532194992 | 75.0723140618647 | 116.24283700823 | 110.192207295608 | 93.590626283524 |
| YBR131W | 80.8075412977958 | 0.37982019157982 | 0.422171788110418 | 0.899681604211032 | 0.368289715816287 | 0.681769945236982 | 136.667183363367 | 62.8434971414533 | 73.8211088275003 | 65.3865958171291 | 64.557050738841 | 81.5698118984842 |
| YBR132C | 462.472383457245 | 0.46874516682427 | 0.454485810540725 | 1.03137470071195 | 0.302365140076929 | 0.62720852586149 | 261.832673077341 | 680.188439648671 | 669.39480038496 | 277.114620367833 | 564.317667665386 | 321.98609959928 |
| YBR133C | 1400.24919705718 | -0.431370461778244 | 0.428169918227401 | -1.00747493790337 | 0.313706595497601 | 0.63492447567613 | 2022.26837164902 | 765.211994604754 | 788.259297649579 | 1701.08937371864 | 1379.07217009352 | 1745.59397462756 |
| YBR134W | 2.28677207404018 | 0.176128641627395 | 1.53538263053762 | 0.114713191437969 | 0.908672457700605 | NA | 3.38285107335066 | 1.23222543414614 | 2.50241046872882 | 1.03788247328776 | 5.56526299472767 | 0 |
| YBR135W | 478.618182567128 | -0.1801811311935 | 0.579440836650595 | -0.310956908448187 | 0.755833380764773 | 0.911525511379585 | 945.845160108844 | 160.189306438998 | 238.980199763603 | 515.827589224019 | 421.846935000358 | 589.019904866949 |
| YBR136W | 417.398659908995 | 0.241773035447832 | 0.31827264884953 | 0.759641258278954 | 0.447469050108011 | 0.740585113740527 | 598.088069768397 | 358.577601336527 | 399.134469762247 | 363.258865650717 | 381.777041438318 | 403.555911497764 |
| YBR137W | 1568.88042140789 | -0.1451194529338 | 0.355266907761627 | -0.408480074454812 | 0.682921256228846 | 0.876016822317207 | 889.689832291223 | 1743.59898931679 | 1838.02048928132 | 1585.8844191837 | 1854.34562984326 | 1501.74316853104 |
| YBR138C | 14.0876979406398 | -1.54048254071073 | 0.740946460595556 | -2.07907402577039 | 0.0376105470507682 | 0.219254994368981 | 12.1782638640624 | 1.23222543414614 | 7.50723140618647 | 28.0228267787696 | 16.695788984183 | 18.8898511764911 |
| YBR139W | 3024.9508061619 | 0.159552046835682 | 0.322811252256689 | 0.494258009038703 | 0.621123976471327 | 0.844223366459491 | 2214.41431261534 | 3704.0696550433 | 3658.52410528154 | 2653.86548419681 | 3366.98411181024 | 2551.84716802416 |
| YBR140C | 3038.62439366987 | 0.663892534642333 | 0.331729990479971 | 2.00130393300215 | 0.0453596461659166 | 0.238761214433781 | 2387.6162875709 | 4428.61821032124 | 4361.70144699434 | 2401.66004318789 | 2472.08982225803 | 2180.06055168686 |
| YBR141C | 136.152122542445 | 0.365257076255604 | 0.411573967636149 | 0.887463991839516 | 0.374829144267987 | 0.687096411016019 | 240.182426207897 | 109.668063639007 | 108.854855389704 | 117.280719481517 | 122.435785884009 | 118.490884652535 |
| YBR141W-A | 6.81782551966751 | -0.805501916560365 | 0.87477263929374 | -0.920812883689067 | 0.357148127331501 | NA | 2.70628085868053 | 7.39335260487685 | 5.00482093745765 | 10.3788247328776 | 11.1305259894553 | 4.29314799465706 |
| YBR142W | 163.994769789999 | 0.887166203755463 | 0.677518483552425 | 1.30943468745501 | 0.190387147982409 | 0.503378715088723 | 501.338529070568 | 70.2368497463301 | 66.3138774213138 | 118.318601954805 | 102.400839102989 | 125.359921443986 |
| YBR143C | 15570.0128971486 | 0.660824387944072 | 0.293984835271528 | 2.24781794385322 | 0.0245878011911284 | 0.173838476318089 | 14335.8462786454 | 20429.0654727089 | 22460.3851620755 | 13250.6455364649 | 11951.9588074772 | 10992.1761255199 |
| YBR144C | 9.12375055750303 | -1.13266404608286 | 0.798814544534474 | -1.41793117543065 | 0.156210859474555 | NA | 2.70628085868053 | 4.92890173658457 | 10.0096418749153 | 15.5682370993165 | 7.79136819261874 | 13.7380735829026 |
| YBR145W | 1938.06094483812 | 0.65902835282441 | 0.407282830816952 | 1.61810983169237 | 0.105638930068017 | 0.373560858745618 | 1184.6744458874 | 2930.23208239953 | 3005.39497294332 | 1267.25449988436 | 1842.10205125486 | 1398.70761665927 |
| YBR146W | 1225.41065493457 | -0.725193805848903 | 0.368762861551697 | -1.96655867892282 | 0.0492341111806683 | 0.250506074429256 | 1401.85348479651 | 648.150578360871 | 719.443009759537 | 1552.67218003849 | 1424.70732665028 | 1605.63735000174 |
| YBR147W | 240.451969283663 | 0.168562202552777 | 0.433430021603438 | 0.388902923542756 | 0.697347959413968 | 0.884846814726597 | 127.195200357985 | 311.753034838974 | 325.313360934747 | 182.667315298646 | 298.298096517403 | 197.484807754225 |
| YBR148W | 9.3151791119321 | -1.49431695844815 | 0.777350991065459 | -1.92231948710839 | 0.0545655694265148 | NA | 2.70628085868053 | 7.39335260487685 | 5.00482093745765 | 15.5682370993165 | 8.90442079156428 | 16.3139623796968 |
| YBR149W | 3613.60356992925 | -0.586107228511375 | 0.347563137497728 | -1.68633311556294 | 0.0917316560819301 | 0.347347535677822 | 4200.14789267218 | 2125.5888739021 | 2341.00499349581 | 3959.52163559282 | 4651.44681099339 | 4403.91121291922 |
| YBR150C | 199.747893202788 | 0.471506869820375 | 0.365547762767242 | 1.28986391887892 | 0.19709791022345 | 0.511983320699798 | 167.112843023523 | 305.591907668243 | 223.96573695123 | 157.75813593974 | 209.25388860176 | 134.804847032232 |
| YBR151W | 2931.08429974798 | 0.125546922086238 | 0.294863959220063 | 0.425779136990221 | 0.670268790629042 | 0.87058290537205 | 2338.90323211465 | 3435.44451039944 | 3402.02703223683 | 2753.50220163244 | 3177.7651699895 | 2478.86365211499 |
| YBR153W | 855.913304431654 | -0.238547556648948 | 0.352920149092951 | -0.675925013808491 | 0.49908825539758 | 0.774921472724282 | 466.156877907721 | 936.491329951068 | 954.669593820046 | 939.283638325426 | 922.720604525848 | 916.157782059817 |
| YBR154C | 639.093388690458 | 0.434016299336582 | 0.511418263627915 | 0.848652326684118 | 0.396074780959062 | 0.702666000294039 | 1403.88319544052 | 383.22211001945 | 415.400137808985 | 592.630892247313 | 404.038093417229 | 635.385903209245 |
| YBR155W | 22.3532548010715 | 1.07797825523185 | 0.515980708483971 | 2.08918325337998 | 0.0366912303273608 | 0.215866112509386 | 33.8285107335066 | 25.876734117069 | 31.2801308591103 | 10.3788247328776 | 15.5827363852375 | 17.1725919786283 |
| YBR156C | 28.3240726275796 | -0.471360253664958 | 0.467149778850563 | -1.00901311528982 | 0.312968344317149 | 0.634104416944316 | 21.6502468694442 | 28.3411849853613 | 21.270488984195 | 31.1364741986329 | 40.0698935620392 | 27.4761471658052 |
| YBR157C | 267.627388676155 | -0.624535693080418 | 0.489878615104604 | -1.27487845728285 | 0.202352265187159 | 0.520420490655842 | 382.262171288624 | 146.634826663391 | 101.347623983517 | 304.099564673315 | 281.60230753322 | 389.817837914861 |
| YBR158W | 2770.76558368569 | 0.125821233155528 | 0.275177259071136 | 0.457237031796301 | 0.647500683726974 | 0.859054305981825 | 3499.89772048859 | 2696.10924991176 | 2477.38636404153 | 2813.69938508313 | 2634.59550170408 | 2502.90528088507 |
| YBR159W | 7576.65435643837 | 0.304248056854686 | 0.306719301809528 | 0.991942975416734 | 0.321225343530144 | 0.638699024600408 | 5871.2763229074 | 9155.43497570584 | 10092.2214203833 | 7012.97187200542 | 6781.82948537514 | 6546.19206225309 |
| YBR160W | 1226.59741570582 | 0.112800387558193 | 0.253233039956131 | 0.4454410355684 | 0.656001062637256 | 0.864280886779837 | 1421.47402102195 | 1200.18757285834 | 1201.15702498984 | 1163.46625255558 | 1221.01870104325 | 1152.28092176596 |
| YBR161W | 402.636590377674 | 0.396415221836778 | 0.299021305871154 | 1.32570895134673 | 0.184936121276847 | 0.496502954273384 | 349.110230769788 | 497.819075395042 | 526.757403667417 | 362.22098317743 | 345.046305673116 | 334.865543583251 |
| YBR162C | 45772.3171580786 | 1.03617741302361 | 0.364856170483487 | 2.83996132407609 | 0.00451190037615382 | 0.0666694173833732 | 34388.7108712535 | 75594.5659339975 | 74630.638614134 | 30344.5698715143 | 31766.5211739056 | 27908.8964836666 |
| YBR162W-A | 807.062597249284 | -1.0177536187947 | 0.299659108020959 | -3.39637138185539 | 0.000682856407964466 | 0.0203370913625894 | 598.764639983067 | 529.856936682841 | 471.704373355383 | 1267.25449988436 | 833.676396610205 | 1141.11873697985 |
| YBR163W | 567.444083010642 | 0.0040614286237627 | 0.271287478627607 | 0.0149709402155555 | 0.988055364132956 | 0.996347598750385 | 644.094844365966 | 548.340318195033 | 511.742940855044 | 587.441479880874 | 512.004195514946 | 601.040719251989 |
| YBR164C | 1805.37400876096 | 0.693367594414987 | 0.351861860907603 | 1.97056763306627 | 0.0487733518730428 | 0.248757077698536 | 1395.08778264981 | 2526.06213999959 | 2772.67079935154 | 1568.24041713781 | 1432.4986948429 | 1137.68421858412 |
| YBR165W | 405.413107569312 | -0.527995637908527 | 0.295971540288523 | -1.78394056872434 | 0.0744333228473225 | 0.311823599060853 | 269.274945438712 | 341.326445258481 | 386.622417418603 | 528.282178903472 | 421.846935000358 | 485.125723396248 |
| YBR166C | 1443.16724577796 | 0.0909733214517197 | 0.254393663881429 | 0.357608440649378 | 0.720636360785175 | 0.895808348138664 | 1373.43753578037 | 1473.74161923879 | 1619.05957326755 | 1483.13405432821 | 1430.27258964501 | 1279.3581024078 |
| YBR167C | 109.159176743949 | -0.0182536148599908 | 0.326914571983922 | -0.0558360392111504 | 0.95547242457605 | 0.986770496029859 | 97.426110912499 | 129.383670585345 | 98.8452135147885 | 117.280719481517 | 112.418312493499 | 99.6010334760439 |
| YBR168W | 318.585491357134 | 0.324129479215708 | 0.316977784463967 | 1.02256213243409 | 0.306514921550423 | 0.631260397374358 | 265.892094365362 | 378.293208282866 | 419.153753512078 | 308.251094566466 | 296.071991319512 | 243.850806096521 |
| YBR169C | 607.78512363961 | 0.18549574224189 | 0.586648601605522 | 0.316195660799721 | 0.751854001106999 | 0.908865783041647 | 1376.82038685372 | 290.80520245849 | 271.511535857077 | 433.834873834285 | 737.95387310089 | 535.784869733201 |
| YBR170C | 484.687676852842 | 0.0562910880530154 | 0.273606680375469 | 0.205737257495933 | 0.836996153799503 | 0.941803181712136 | 564.93612924956 | 442.368930858465 | 474.206783824112 | 473.27440781922 | 512.004195514946 | 441.335613850746 |
| YBR171W | 1361.48250477217 | 0.0295756753857644 | 0.330657073152079 | 0.0894451617315369 | 0.928728133407198 | 0.9802270407366 | 886.306981217873 | 1583.40968287779 | 1657.84693553285 | 1305.65615139601 | 1390.20269608297 | 1345.47258152552 |
| YBR172C | 215.277164121088 | -0.548741853415705 | 0.354666274245332 | -1.54720618582449 | 0.121813534834534 | 0.402173577385928 | 232.740153846525 | 136.777023190222 | 153.898243826823 | 283.34191520756 | 198.123362612305 | 286.782286043092 |
| YBR173C | 1354.66829242377 | -1.28165375856459 | 0.418596562968649 | -3.06178758247613 | 0.00220019527474679 | 0.0425815570344935 | 1312.54621646006 | 517.53468234138 | 536.767045542333 | 1960.55999204059 | 1642.86563604361 | 2157.73618211464 |
| YBR174C | 12.7635298212807 | -0.130792238322691 | 0.611805111572668 | -0.21378088520131 | 0.830717931766084 | 0.939639058736332 | 12.8548340787325 | 13.5544797756076 | 10.0096418749153 | 14.5303546260287 | 14.469683786292 | 11.1621847861084 |
| YBR175W | 1643.33918742283 | 0.115716465132901 | 0.376426331507868 | 0.307407998450509 | 0.758532840868442 | 0.912866087924729 | 921.48863238072 | 2135.44667737526 | 2071.99586810747 | 1599.37689133644 | 1462.55111501443 | 1669.17594032267 |
| YBR176W | 502.281039769792 | 0.0149855267036007 | 0.30459722745092 | 0.0491978434242818 | 0.960761629885404 | 0.988087915299789 | 610.942903847129 | 423.885549346273 | 479.21160476157 | 534.509473743198 | 409.603356411957 | 555.533350508624 |
| YBR177C | 10097.1493432917 | 0.762578564026527 | 0.332937340772209 | 2.2904567035281 | 0.0219948556957343 | 0.162710978814776 | 8205.44356351936 | 14391.1608453928 | 15519.9497270562 | 7407.36721185477 | 8306.71154593053 | 6752.26316599663 |
| YBR178W | 18.4530715810963 | -0.303064027829291 | 0.609122438090382 | -0.497542052102705 | 0.618806856105467 | 0.842468453077742 | 8.11884257604158 | 25.876734117069 | 16.2656680467373 | 19.7197669924675 | 16.695788984183 | 24.0416287700796 |
| YBR179C | 226.627940651559 | -0.00872571638773829 | 0.296276540892074 | -0.0294512564560987 | 0.97650469377263 | 0.992989672947336 | 259.12639221866 | 221.800578146306 | 196.439221795213 | 218.993201863718 | 209.25388860176 | 254.154361283698 |
| YBR180W | 24.2971285416695 | 0.561355870016965 | 0.553490189138918 | 1.01421105745394 | 0.310482053479132 | 0.633323413761106 | 15.561114937413 | 35.7345375902381 | 36.2849517965679 | 16.6061195726042 | 16.695788984183 | 24.900258369011 |
| YBR181C | 21630.0787675893 | -0.0250917367674999 | 0.247417518351065 | -0.101414551947356 | 0.9192213852471 | 0.977916809624344 | 21671.220546099 | 19575.1332468456 | 23079.7317530859 | 21688.6300442944 | 20455.680663421 | 23310.07635179 |
| YBR182C | 535.102961510522 | -0.153405218068735 | 0.432847941557741 | -0.354409027605993 | 0.723032370020096 | 0.896460324175046 | 838.270495976293 | 354.880925034089 | 325.313360934747 | 464.971348032918 | 714.579768523033 | 512.601870562053 |
| YBR182C-A | 16.6741881823587 | -0.190291034049741 | 0.653694541968566 | -0.29110084578141 | 0.770974193085967 | 0.918097350879794 | 7.44227236137145 | 22.1800578146306 | 17.5168732811018 | 12.4545896794532 | 26.7132623746928 | 13.7380735829026 |
| YBR183W | 2623.02756703571 | 0.37643465261144 | 0.390687923887643 | 0.963517502321617 | 0.33528788511417 | 0.650461589593466 | 1587.23372361613 | 3721.32081112135 | 3582.20058598531 | 2025.94658785771 | 2807.11865454064 | 2014.34503909309 |
| YBR184W | 33.3180065228568 | -0.0258206693680104 | 0.447498126721259 | -0.0577000613548797 | 0.953987544825141 | 0.986654799509224 | 26.3862383721351 | 43.127890195115 | 30.0289256247459 | 34.2501216184962 | 30.0524201715294 | 36.0624431551193 |
| YBR185C | 1220.67763135839 | -0.631227084069916 | 0.301176772592085 | -2.09586907594914 | 0.0360938066170379 | 0.213443621846434 | 720.54727862369 | 1003.03150339496 | 1151.10881561526 | 1656.46042736727 | 1430.27258964501 | 1362.64517350415 |
| YBR186W | 15.1024070666872 | 0.899584845642518 | 0.68633443114909 | 1.31070918901212 | 0.189956032972124 | 0.503344331209052 | 15.561114937413 | 25.876734117069 | 17.5168732811018 | 4.15152989315105 | 18.9218941820741 | 8.58629598931413 |
| YBR187W | 9721.45831901281 | 1.24284494859295 | 0.38205192259797 | 3.2530786395251 | 0.0011416189832613 | 0.0275715374612856 | 7094.515271031 | 16779.213736768 | 17130.2508636832 | 5830.82373493066 | 5951.49224656177 | 5542.45406110227 |
| YBR188C | 29.1333633950535 | 0.643806505782923 | 0.702554218958208 | 0.916379815834855 | 0.359467718048035 | 0.673914038923714 | 73.7461533990444 | 22.1800578146306 | 10.0096418749153 | 30.0985917253451 | 15.5827363852375 | 23.1829991711481 |
| YBR189W | 77756.7941156806 | 0.145457836273349 | 0.240757586602809 | 0.604167197079107 | 0.545732487420882 | 0.801244019845526 | 75776.5406132694 | 81257.8740293332 | 87986.0032857398 | 73282.8056739024 | 75124.3721132299 | 73113.1689786087 |
| YBR190W | 2.91509148447489 | 1.09963558231797 | 1.33617205473829 | 0.822974540156324 | 0.410522471814071 | NA | 3.38285107335066 | 3.69667630243843 | 5.00482093745765 | 0 | 1.11305259894553 | 4.29314799465706 |
| YBR191W | 66370.8905343416 | -0.286613661941684 | 0.294018748193909 | -0.974814237875262 | 0.329652413275671 | 0.646188032846309 | 44326.850754543 | 62497.2417944582 | 72576.1596193077 | 74217.9377823347 | 74616.8201281107 | 69990.3331272952 |
| YBR191W-A | 5278.62219849766 | -0.618401535574271 | 0.355547765391249 | -1.73929242641639 | 0.0819833388227179 | 0.328379879990289 | 2455.27330903791 | 4665.2054936773 | 5373.92648159515 | 6858.32738348554 | 6465.72254727461 | 5853.27797591544 |
| YBR192W | 454.082847189277 | -0.280569097849357 | 0.273902233931219 | -1.02434030501486 | 0.305674566962735 | 0.630422658405217 | 422.179813954162 | 351.184248731651 | 456.68991054301 | 464.971348032918 | 522.021668905456 | 507.450092968465 |
| YBR193C | 259.081173208784 | -0.28501053763839 | 0.42298030057679 | -0.673815156993695 | 0.500428837353528 | 0.776386331879394 | 384.968452147305 | 152.795953834122 | 161.405475233009 | 309.288977039754 | 262.680413351146 | 283.347767647366 |
| YBR194W | 54.0612919635468 | -0.236519879905274 | 0.394814003262716 | -0.599066593258319 | 0.54912847781676 | 0.803335259270233 | 60.2147491056417 | 50.5212427999918 | 37.5361570309323 | 67.4623607637046 | 54.5395773483312 | 54.093664732679 |
| YBR195C | 430.824727358241 | -0.0517558706325746 | 0.377508898090583 | -0.137098412499288 | 0.890953007263334 | 0.965832153362604 | 640.711993292615 | 298.198555063366 | 329.06697663784 | 429.683343941134 | 399.585883021447 | 487.701612193042 |
| YBR196C | 107760.209529561 | 0.844835550538683 | 0.342332318052359 | 2.46788137136812 | 0.0135915354006149 | 0.125198951094126 | 85371.6593977212 | 161257.645890403 | 168692.494517947 | 75408.3889791958 | 84912.556668357 | 70918.51172374 |
| YBR196C-A | 91.7047100374413 | 0.488734046880291 | 0.398554945739008 | 1.22626516646048 | 0.220098905197024 | 0.541578984191991 | 74.4227236137145 | 115.829190809737 | 131.376549608263 | 67.4623607637046 | 100.174733905098 | 60.9627015241303 |
| YBR196C-B | 46.9397375633859 | 0.655329680235836 | 0.482140425281278 | 1.35920915541052 | 0.174080320939809 | 0.482921662529934 | 33.8285107335066 | 86.25578039023 | 52.5506198433053 | 35.288004091784 | 44.5221039578214 | 29.193406363668 |
| YBR197C | 181.652080801769 | 0.967122650808439 | 0.470474489790479 | 2.05563249824478 | 0.0398179528498547 | 0.222926776611754 | 422.179813954162 | 157.724855570706 | 140.134986248814 | 112.091307115078 | 143.583785263974 | 114.197736657878 |
| YBR198C | 1106.92385502875 | 0.661047725147099 | 0.261860428371121 | 2.52442772380343 | 0.0115886832185219 | 0.113092956095184 | 1190.76357781943 | 1338.19682148271 | 1540.23364350259 | 864.556100248707 | 822.54587062075 | 885.247116498286 |
| YBR199W | 2833.42613553172 | -0.425228795010418 | 0.243833475919323 | -1.74393115386303 | 0.0811710954568499 | 0.327053880038495 | 2453.2435983939 | 2293.17153294597 | 2509.91770013501 | 3418.78486700989 | 3290.183482483 | 3035.25563222254 |
| YBR200W | 180.235363550999 | 0.793626310343534 | 0.659046615433493 | 1.20420360526625 | 0.228510892862114 | 0.551650824095519 | 528.401337657373 | 72.7013006146224 | 83.8307507024156 | 147.379311206862 | 116.870522889281 | 132.228958235438 |
| YBR201C-A | 476.542920022485 | -1.04524702365048 | 0.418806750633997 | -2.49577405824563 | 0.0125682627007572 | 0.119408882651161 | 157.640860018141 | 407.866618702373 | 369.105544137501 | 554.229240735666 | 830.337238813369 | 540.078017727859 |
| YBR201W | 675.88981776187 | -0.567435128227338 | 0.268465510752598 | -2.11362393119559 | 0.0345474002143596 | 0.208810153977013 | 506.751090787929 | 563.127023404787 | 564.29356069835 | 807.47256421788 | 910.477025937447 | 703.217641524827 |
| YBR202W | 697.039112529091 | -0.0238109777457637 | 0.543316218307121 | -0.0438252659196418 | 0.965043687137617 | 0.989039165251922 | 1407.26604651387 | 348.719797863358 | 316.554924294196 | 736.896556034312 | 596.596193034807 | 776.201157433997 |
| YBR203W | 431.369771945327 | -0.50424551151069 | 0.276870986636464 | -1.82122914949111 | 0.0685720312195516 | 0.300208125976541 | 402.559277728728 | 337.629768956043 | 329.06697663784 | 499.221469651414 | 562.091562467495 | 457.649576230443 |
| YBR204C | 479.734145921516 | 0.22991627496974 | 0.276247138985149 | 0.832284728140118 | 0.405248248726163 | 0.710767715087082 | 543.285882380116 | 559.430347102349 | 450.433884371188 | 425.531814047983 | 495.308406530763 | 404.414541096695 |
| YBR205W | 2058.86259843504 | 0.0174224217044852 | 0.335028439994075 | 0.0520028141634582 | 0.958526451071728 | 0.987637729414339 | 2945.11014445908 | 1594.49971178511 | 1672.86139834522 | 2038.40117753717 | 1982.346678722 | 2119.95647976166 |
| YBR206W | 317.39034332944 | -0.388973281917579 | 0.263298860116452 | -1.47730712448031 | 0.139593329099389 | 0.428994403424904 | 274.687507156073 | 285.876300721905 | 264.004304450891 | 351.842158444552 | 367.307357652026 | 360.624431551193 |
| YBR207W | 2124.83938198018 | 0.198254732850747 | 0.283797716731811 | 0.69857761765609 | 0.484816035476763 | 0.765823312191672 | 1766.52483050371 | 2590.13786257519 | 2456.11587505734 | 1841.20350761249 | 2115.91299059546 | 1979.14122553691 |
| YBR208C | 4516.04767047363 | 0.0993185387056906 | 0.292675728578268 | 0.339346686478413 | 0.734348576177833 | 0.901690475492975 | 3593.94098032774 | 4799.51806599922 | 5621.6651179993 | 4458.74310524423 | 4718.22996693012 | 3904.18878634113 |
| YBR210W | 1377.08696122503 | -0.211757213791812 | 0.357178193176579 | -0.592861540366003 | 0.553273818477575 | 0.804303972839934 | 755.052359571867 | 1417.05924926806 | 1657.84693553285 | 1601.45265628302 | 1405.78543246821 | 1425.32513422614 |
| YBR211C | 221.806467572513 | -0.622828202740558 | 0.375756734133005 | -1.65753038113243 | 0.0974122917799981 | 0.357617462987508 | 255.74354114531 | 147.867052097537 | 118.864497264619 | 279.190385314408 | 241.532413971181 | 287.640915642023 |
| YBR212W | 226.940801651407 | 1.24425734369679 | 0.635274010601606 | 1.95861521631977 | 0.0501578662284218 | 0.252237458513533 | 719.87070840902 | 134.31257232193 | 102.598829217882 | 125.583779267819 | 132.453259274519 | 146.825661417272 |
| YBR213W | 320.14730603918 | 0.0579707510242747 | 0.368627022015539 | 0.15726126290826 | 0.875038948055424 | 0.958285320584837 | 480.364852415794 | 234.122832487767 | 264.004304450891 | 283.34191520756 | 313.880832902641 | 345.169098770428 |
| YBR214W | 1564.65761322785 | 0.251102448714316 | 0.384602761931728 | 0.652887793767038 | 0.513828618668797 | 0.783014890919344 | 2606.82503712402 | 1239.61878675102 | 1253.70764483314 | 1142.70860308983 | 1626.16984705943 | 1518.91576050967 |
| YBR215W | 52.004787008672 | -0.225037638143989 | 0.385159007177686 | -0.584272038171945 | 0.559037301526407 | 0.809182184103241 | 41.270783094878 | 56.6823699707225 | 46.2945936714832 | 48.7804762445249 | 62.3309455409499 | 56.6695535294732 |
| YBR216C | 1237.09903662827 | 0.724972652666331 | 0.32097881460772 | 2.25863084936726 | 0.0239063571933412 | 0.170277250492349 | 1071.68722003749 | 1778.10130147288 | 1775.4602275631 | 944.473050691865 | 1044.04333781091 | 808.829082193391 |
| YBR217W | 33.1943133791407 | -0.83068272183998 | 0.468360925523828 | -1.7735952693127 | 0.0761301207632072 | 0.316114892653127 | 32.4753703041663 | 20.9478323804844 | 17.5168732811018 | 42.5531814047983 | 36.7307357652026 | 48.9418871390905 |
| YBR218C | 8912.9540909991 | 0.534000699186057 | 0.261190590714408 | 2.04448673945512 | 0.040905486093766 | 0.225888757821714 | 10971.9391713055 | 9053.16026467171 | 11606.1797539643 | 7603.52699930616 | 7647.78440735477 | 6595.13394939218 |
| YBR220C | 1422.87633977654 | 0.613471359775412 | 0.271564965281045 | 2.2590224742007 | 0.0238819869207393 | 0.170277250492349 | 1454.62596154078 | 1701.70332455582 | 2006.93319592052 | 1175.92084223504 | 1153.12249250757 | 1044.95222189953 |
| YBR221C | 20581.7503374021 | 0.63732366010237 | 0.339400227046202 | 1.87779385314204 | 0.0604093714908158 | 0.279667472206836 | 15563.144648057 | 29324.5008818099 | 30279.1666716188 | 16515.8237974282 | 17109.8445509908 | 14698.0214745079 |
| YBR221W-A | 2.07784607601702 | -1.00425858904799 | 1.70887852497145 | -0.587671138921221 | 0.556753051187912 | NA | 2.70628085868053 | 1.23222543414614 | 0 | 5.18941236643882 | 3.3391577968366 | 0 |
| YBR222C | 3324.82077309914 | 0.134433305034442 | 0.25151398162174 | 0.534496349537422 | 0.592998174406865 | 0.827869642879033 | 3259.7152942807 | 3331.93757393117 | 3847.45609567056 | 3007.78340758794 | 3420.41063655963 | 3081.62163056484 |
| YBR223C | 314.528129956749 | 0.321255552215771 | 0.294043557704483 | 1.09254409354765 | 0.274594020579924 | 0.600152316299675 | 381.585601073954 | 362.274277638966 | 304.042871950552 | 231.447791543171 | 303.863359512131 | 303.95487802172 |
| YBR225W | 201.767955983309 | 0.0988948762264815 | 0.367588947953055 | 0.269036587680844 | 0.787901527388867 | 0.92631956251959 | 293.631473166837 | 187.298265990214 | 143.888601951907 | 173.326373039057 | 219.27136199227 | 193.191659759568 |
| YBR226C | 2.62878018586758 | 0.435678073857459 | 1.37655272502863 | 0.316499372625482 | 0.7516235012877 | NA | 4.05942128802079 | 1.23222543414614 | 3.75361570309323 | 4.15152989315105 | 0 | 2.57588879679424 |
| YBR227C | 145.636939161223 | 0.478094377743588 | 0.482237399681511 | 0.991408750253175 | 0.321486029250028 | 0.638699024600408 | 292.954902952167 | 104.739161902422 | 110.106060624068 | 138.038368947273 | 84.5919975198606 | 143.391143021546 |
| YBR228W | 173.112963698772 | 0.78179037347695 | 0.390046735289477 | 2.00435051173223 | 0.0450325265562672 | 0.237910811254985 | 130.578051431335 | 248.909537697521 | 277.767562028899 | 140.114133893848 | 139.131574868192 | 102.176922272838 |
| YBR229C | 919.110279661066 | -0.054526030293838 | 0.378069751602039 | -0.144222144360369 | 0.885325055076373 | 0.962338959262291 | 1384.93922942976 | 728.24523158037 | 590.568870620002 | 948.624580585016 | 904.91176294272 | 957.372002808525 |
| YBR230C | 2044.04425934825 | -0.774353056492834 | 0.327161098020833 | -2.36688610344352 | 0.0179384523246518 | 0.147663696225841 | 1000.64734749712 | 1718.95448063387 | 1806.74035842221 | 2375.71298135569 | 2873.90181047737 | 2488.30857770323 |
| YBR230W-A | 807.065820319503 | -1.38988395431824 | 0.264928268457856 | -5.24626519626892 | 1.55213440597929e-07 | 3.7173619023204e-05 | 439.770639535586 | 430.046676517004 | 467.95075765229 | 1136.4813082501 | 1040.70418001407 | 1327.44135994796 |
| YBR231C | 151.28595948241 | -0.0437606791019171 | 0.33913960482955 | -0.129034410840666 | 0.897330420509831 | 0.966604494954798 | 188.086519678297 | 144.170375795099 | 113.859676327161 | 133.886839054122 | 160.279574248157 | 167.432771791625 |
| YBR233W | 16.0563494201015 | 0.806310378617521 | 0.932089981453876 | 0.865056372947852 | 0.387007848436845 | 0.697560712704605 | 52.0959065296001 | 4.92890173658457 | 3.75361570309323 | 16.6061195726042 | 7.79136819261874 | 11.1621847861084 |
| YBR233W-A | 184.177408630455 | -1.12023029593977 | 0.287157374710575 | -3.90110230346286 | 9.57556709334448e-05 | 0.00519248675967397 | 113.663796064582 | 115.829190809737 | 118.864497264619 | 268.811560581531 | 233.741045778562 | 254.154361283698 |
| YBR234C | 7675.10812851314 | 0.735079742082222 | 0.35449641450769 | 2.07358865139178 | 0.0381175300698987 | 0.220866494800179 | 5657.48013507164 | 11219.4125779006 | 11891.4545473994 | 5621.17147532653 | 6371.11307636424 | 5290.01695901643 |
| YBR235W | 765.721982796517 | -0.0149671871822957 | 0.378648356600097 | -0.0395279338241074 | 0.968469482944122 | 0.990124204883325 | 1174.52589266735 | 523.69580951211 | 585.564049682545 | 720.290436461708 | 789.154292652384 | 801.101415803008 |
| YBR236C | 656.828853450847 | 0.171117268251829 | 0.29138534693924 | 0.587254198089481 | 0.557032997199994 | 0.807837748285647 | 875.48185778315 | 605.022688165756 | 605.583333432375 | 618.577954079507 | 615.518087216881 | 620.789200027411 |
| YBR237W | 83.1525418380683 | 0.656525957435541 | 0.500599346681319 | 1.31147985267645 | 0.189695696143003 | 0.50303910933296 | 179.967677102255 | 51.753468234138 | 72.5699035931359 | 68.5002432369924 | 63.4439981398955 | 62.6799607219931 |
| YBR238C | 1013.91688056065 | 0.58146946479434 | 0.283468277324581 | 2.05126820638395 | 0.0402408348790609 | 0.223864582548538 | 1469.51050626353 | 1187.86531851688 | 988.452135147885 | 827.192331210348 | 772.458503668201 | 838.022488557059 |
| YBR239C | 215.583959020987 | 0.103278310231937 | 0.281473107884547 | 0.366920701619209 | 0.713678169176496 | 0.891399851461647 | 234.093294275866 | 236.587283356059 | 198.941632263941 | 202.387082291114 | 213.706098997543 | 207.788362941402 |
| YBR240C | 329.366055813526 | -0.354231245942015 | 0.335949295176847 | -1.05441877993983 | 0.291691227895994 | 0.615507040362032 | 202.97106440104 | 324.075289180435 | 341.579028981484 | 303.061682200027 | 401.811988219338 | 402.697281898833 |
| YBR241C | 1604.59469189409 | 0.806392365642039 | 0.428634325080228 | 1.88130608879983 | 0.0599302932358041 | 0.278704955921846 | 939.079457962143 | 2889.5686430727 | 2297.21281029306 | 984.950467150088 | 1409.12459026505 | 1107.63218262152 |
| YBR242W | 322.440837585216 | 0.498989411552404 | 0.579936213860427 | 0.860421197412057 | 0.389556905171713 | 0.699637138162367 | 786.851159661363 | 172.51156078046 | 172.666322342289 | 315.51627187948 | 181.427573628122 | 305.672137219583 |
| YBR243C | 2771.19960095131 | 0.701669239438253 | 0.360664881485198 | 1.94548811225761 | 0.0517162591031154 | 0.258042584483253 | 1960.02391189937 | 4113.16849917982 | 4224.06887121425 | 2174.36378153786 | 2181.58309393325 | 1973.98944794332 |
| YBR244W | 252.637911325114 | 0.0708781960471517 | 0.422001199880234 | 0.167957332982151 | 0.866616845508051 | 0.954796816764773 | 125.842059928645 | 337.629768956043 | 314.052513825467 | 282.304032734272 | 248.210729564854 | 207.788362941402 |
| YBR245C | 127.943699666307 | 0.817721678652834 | 0.321161294877679 | 2.54614018468284 | 0.0108921437196027 | 0.109838670351362 | 185.380238819616 | 168.814884478021 | 135.130165311356 | 101.712482382201 | 89.0442079156428 | 87.5802190910041 |
| YBR246W | 3428.90803548252 | 0.655895945849619 | 0.33894400525879 | 1.93511593559187 | 0.0529760704298048 | 0.261887176500704 | 2622.38615206143 | 4744.06792146265 | 5220.02823776832 | 2734.82031711326 | 2779.292339567 | 2472.85324492247 |
| YBR247C | 2070.11870718802 | 1.05953735664012 | 0.243754727041787 | 4.34673562846836 | 1.38178599382941e-05 | 0.00120340998371689 | 2672.45234794702 | 2941.32211130684 | 2780.17803075772 | 1357.55027506039 | 1272.21912059475 | 1396.99035746141 |
| YBR248C | 1906.16262323205 | 0.132784424755739 | 0.324004286687713 | 0.409823049297251 | 0.681935756798575 | 0.875534226061695 | 2716.42941190058 | 1500.85057879 | 1762.94817521946 | 1964.71152193374 | 1664.01363542357 | 1828.02241612498 |
| YBR249C | 26777.4287633392 | 0.304241840322555 | 0.299350786961911 | 1.01633886922524 | 0.309468045127155 | 0.633323413761106 | 21539.2893542383 | 31055.7776167852 | 36177.3481464126 | 24551.109905622 | 24546.1489645459 | 22794.8985924311 |
| YBR250W | 47.7980098483086 | -0.196523786043891 | 0.400987655735321 | -0.490099341545839 | 0.624063604010149 | 0.846417554471528 | 41.9473533095482 | 49.2890173658457 | 42.54097796839 | 64.3487133438414 | 42.2959987599303 | 46.3659983422963 |
| YBR251W | 243.171818916672 | -0.127495323754108 | 0.546194765265529 | -0.233424653369072 | 0.815431671237147 | 0.934057641743946 | 467.510018337061 | 112.132514507299 | 116.36208679589 | 255.31908842879 | 211.479993799652 | 296.227211631337 |
| YBR252W | 5055.58033720527 | -0.134609346761041 | 0.332987487184655 | -0.404247462567248 | 0.686030754973953 | 0.877778907010381 | 3066.21621288504 | 5351.5550604967 | 6043.32128198011 | 5303.57943850047 | 5123.3811129463 | 5445.42891642302 |
| YBR253W | 508.791105559373 | -0.939286943917567 | 0.334172970154754 | -2.81078072676731 | 0.00494214543173182 | 0.070841526038889 | 391.057584079336 | 289.572977024343 | 365.351928434408 | 792.942209591851 | 460.803775963451 | 753.018158262849 |
| YBR254C | 702.690862985975 | -0.359976753065716 | 0.308602394473083 | -1.16647427081812 | 0.243422745289379 | 0.567946943988665 | 447.889482111627 | 676.491763346232 | 723.19662546263 | 853.139393042542 | 803.623976438676 | 711.803937514141 |
| YBR255C-A | 600.08298172527 | -0.529151923310732 | 0.403029269590131 | -1.31293671015225 | 0.18920427553065 | 0.502330797113246 | 240.858996422567 | 609.95158990234 | 624.351411947841 | 686.040314843212 | 763.554082876637 | 675.741494359022 |
| YBR255W | 61.8927956576637 | -0.134960739475271 | 0.395895152740015 | -0.340900206888615 | 0.733178709061113 | 0.901172486135829 | 46.006774597569 | 73.9335260487685 | 57.5554407807629 | 79.9169504431578 | 58.9917877441133 | 54.9522943316104 |
| YBR256C | 2639.63549036044 | -0.652583237710361 | 0.278633563427604 | -2.34208409669899 | 0.0191763934546464 | 0.152937438798529 | 1683.30669409929 | 2019.61748656553 | 2456.11587505734 | 3384.5347453914 | 2969.62433398669 | 3324.61380706243 |
| YBR257W | 78.7123615085484 | -0.0686992096236098 | 0.336096621517184 | -0.204403154406887 | 0.838038465363386 | 0.941803181712136 | 82.5415661897561 | 72.7013006146224 | 75.0723140618647 | 87.1821277561722 | 72.3484189314597 | 82.4284414974156 |
| YBR258C | 38.6298593521959 | -0.199579387335165 | 0.435563057365423 | -0.458210089125453 | 0.646801512139529 | 0.859054305981825 | 29.0925192308157 | 40.6634393268227 | 38.7873622652968 | 39.439533984935 | 32.2785253694205 | 51.5177759358848 |
| YBR259W | 292.181193322954 | -0.248324837111449 | 0.278866822752679 | -0.890478238537839 | 0.373209148245809 | 0.685851509845389 | 274.687507156073 | 272.321820946297 | 253.994662575976 | 361.183100704142 | 304.976412111077 | 285.92365644416 |
| YBR260C | 418.707948849332 | 0.183754284255993 | 0.324480896772961 | 0.566302318822069 | 0.571188284379029 | 0.8150306600566 | 584.556665474994 | 351.184248731651 | 399.134469762247 | 340.425451238386 | 438.542723984541 | 398.404133904175 |
| YBR261C | 845.688243641491 | 0.643866288039475 | 0.504339603870008 | 1.27665224602395 | 0.201725047804735 | 0.520188799366767 | 1918.07655858982 | 473.174566712119 | 701.926136478435 | 609.237011819917 | 524.247774103347 | 847.467414145304 |
| YBR262C | 1711.9611709483 | -0.698343851722643 | 0.294832313855164 | -2.36861367938693 | 0.0178548924060849 | 0.147663696225841 | 983.056521915702 | 1399.80809319002 | 1535.22882256513 | 2064.34823936936 | 1977.89446832622 | 2311.43088032336 |
| YBR263W | 7470.84155126115 | 0.0571472151305676 | 0.379123702842608 | 0.150735009977184 | 0.880184754789536 | 0.960354537944778 | 11672.8659137038 | 4704.63670756997 | 6477.48949830456 | 7351.32155829723 | 7015.5705311537 | 7603.16509853766 |
| YBR264C | 272.145188345164 | 0.0464050200009561 | 0.275015830749889 | 0.168735813769058 | 0.866004447353827 | 0.954697653124242 | 275.364077370744 | 258.76734117069 | 295.284435310001 | 280.228267787696 | 279.376202335329 | 243.850806096521 |
| YBR265W | 4159.45698723735 | 0.544419143031559 | 0.348943268277269 | 1.5601938553489 | 0.118714077268785 | 0.396495360918639 | 2934.96159123903 | 5851.83858676003 | 6019.54838252718 | 3241.30696407769 | 3428.20200475225 | 3480.88439406795 |
| YBR267W | 326.086488398957 | 0.550180027521029 | 0.39016838197329 | 1.41010920653917 | 0.15850743920922 | 0.45813700712836 | 600.117780412407 | 269.857370078005 | 291.530819606908 | 272.963090474682 | 240.419361372235 | 281.630508449503 |
| YBR268W | 1285.39356224785 | -0.854439031488246 | 0.30221721981238 | -2.82723476848438 | 0.00469518920728907 | 0.0687530025453474 | 660.332529518049 | 1010.42485599984 | 1077.28770678776 | 1723.92278813098 | 1626.16984705943 | 1614.22364599106 |
| YBR269C | 1358.90806311703 | -0.855539794529066 | 0.344741700884149 | -2.48168351068318 | 0.0130763359341232 | 0.122414949428892 | 592.675508051035 | 1127.48627224372 | 1183.64015170873 | 1646.08160263439 | 1847.66731424959 | 1755.89752981474 |
| YBR270C | 144.871699549843 | -0.0659326994855481 | 0.35735963687829 | -0.184499570408965 | 0.853621564136553 | 0.949027886603389 | 188.763089892967 | 98.5780347316914 | 136.381370545721 | 145.303546260287 | 141.357680066083 | 158.846475802311 |
| YBR271W | 341.161906573338 | 0.766931098539874 | 0.498866178720152 | 1.53734835363553 | 0.124208026419168 | 0.406181701839891 | 804.441985242787 | 258.76734117069 | 225.216942185594 | 252.205441008927 | 209.25388860176 | 297.085841230269 |
| YBR272C | 800.754115014579 | 0.840811398071367 | 0.326491543733843 | 2.57529303349063 | 0.0100155193700244 | 0.104671282434364 | 754.375789357197 | 1184.16864221444 | 1144.85278944344 | 587.441479880874 | 681.188190554667 | 452.497798636854 |
| YBR273C | 187.212147623451 | 0.796833516655321 | 0.517168731789492 | 1.54076120166457 | 0.123374915384569 | 0.406162092571879 | 451.272333184978 | 140.47369949266 | 120.115702498983 | 117.280719481517 | 163.618732044994 | 130.511699037575 |
| YBR274W | 1223.44281931524 | 0.570268201460495 | 0.352313837244784 | 1.6186369684489 | 0.105525396056838 | 0.373497522496741 | 882.924130144522 | 1722.65115693631 | 1781.71625373492 | 1055.52647533366 | 1022.89533843095 | 874.943561311109 |
| YBR275C | 76.890411309678 | -0.152133594938739 | 0.350660500202103 | -0.433848679423707 | 0.664398329318917 | 0.867354439456083 | 79.1587151164054 | 65.3079480097455 | 73.8211088275003 | 94.4473050691865 | 67.8962085356776 | 80.7111822995528 |
| YBR276C | 1614.07349809664 | 0.399320749010351 | 0.259431148343866 | 1.53921667293808 | 0.123751408564087 | 0.406181701839891 | 1719.84148569148 | 1896.39494315091 | 1891.82231435899 | 1432.27781313711 | 1536.01258654484 | 1208.0918456965 |
| YBR277C | 8.24490946707883 | 0.0881092362001108 | 0.789584483586379 | 0.111589371412048 | 0.911148999768908 | NA | 4.73599150269092 | 13.5544797756076 | 7.50723140618647 | 10.3788247328776 | 5.56526299472767 | 7.72766639038271 |
| YBR278W | 63.1132615636925 | -0.604614270877848 | 0.511819705493025 | -1.18130322922099 | 0.237482284941877 | 0.564156198917009 | 89.3072683364574 | 32.0378612877997 | 27.5265151560171 | 78.87906796987 | 66.7831559367321 | 84.1457006952784 |
| YBR279W | 4.98550234327126 | -0.556888970683554 | 0.991836603062338 | -0.561472493517718 | 0.574475476623143 | NA | 6.76570214670132 | 1.23222543414614 | 3.75361570309323 | 8.30305978630211 | 5.56526299472767 | 4.29314799465706 |
| YBR280C | 699.369365460679 | 0.617642685563496 | 0.316036341816309 | 1.95434070023026 | 0.0506609385695577 | 0.254185190954502 | 730.019261629072 | 895.827890624245 | 914.631026320385 | 468.084995452781 | 725.710294512489 | 461.9427242251 |
| YBR281C | 757.278715297822 | 0.202996146465912 | 0.360097250441543 | 0.563725899647949 | 0.572940688283683 | 0.815365951041003 | 1194.82299910745 | 638.292774887702 | 596.824896791824 | 762.843617866506 | 722.371136715652 | 628.516866417794 |
| YBR282W | 240.399527318017 | -0.608009568856772 | 0.280632698133448 | -2.16656709250484 | 0.0302678788711473 | 0.195044582680891 | 190.116230322307 | 177.440462517044 | 203.946453201399 | 315.51627187948 | 266.019571147983 | 289.358174839886 |
| YBR283C | 17562.7177683328 | 0.940378492537256 | 0.36382313272905 | 2.58471330693967 | 0.0097459998606624 | 0.10363332802627 | 13007.0623770333 | 27876.6359966882 | 28393.6003834316 | 12039.4366901381 | 12951.4800413302 | 11108.0911213757 |
| YBR284W | 165.293128527918 | -0.0183662163089932 | 0.313449815807554 | -0.0585938015681252 | 0.953275647834343 | 0.98639575585091 | 131.931191860676 | 168.814884478021 | 192.685606092119 | 171.250608092481 | 172.523152836558 | 154.553327807654 |
| YBR285W | 15.9404507281377 | -0.770242212796874 | 0.560173983504955 | -1.37500532955412 | 0.16912979241621 | 0.473992221749572 | 12.8548340787325 | 11.0900289073153 | 11.2608471092797 | 18.6818845191797 | 21.1479993799652 | 20.6071103743539 |
| YBR286W | 38760.2872159377 | 0.278531299987947 | 0.239410327587577 | 1.16340553389895 | 0.244665005349005 | 0.568215939695386 | 42644.2206306584 | 39643.1566673497 | 45183.5234233676 | 34261.5383257024 | 35324.9503327344 | 35504.3339158139 |
| YBR287W | 11599.9708180103 | 1.35710282400607 | 0.38550818855444 | 3.52029571432678 | 0.000431065909155907 | 0.0152948570730133 | 9237.21314089131 | 21281.765473138 | 19540.072145069 | 5586.92135370803 | 8148.65807688026 | 5805.19471837528 |
| YBR288C | 937.831815284914 | 0.0625157583591506 | 0.262107369104816 | 0.238512021133411 | 0.811483992837946 | 0.932635978500889 | 986.439372989052 | 911.846821268145 | 975.940082804241 | 1052.41282791379 | 842.58081740177 | 857.770969332481 |
| YBR289W | 86.577007888973 | 0.15433239630832 | 0.801434991853916 | 0.192570074774638 | NA | NA | 235.446434705206 | 16.0189306438998 | 21.270488984195 | 90.2957751760354 | 64.557050738841 | 91.8733670856611 |
| YBR290W | 92.1074106512553 | -0.134992456270045 | 0.379443105773537 | -0.355764683073758 | 0.722016799726384 | 0.896293267442601 | 119.752927996613 | 77.630202351207 | 65.0626721869494 | 101.712482382201 | 84.5919975198606 | 103.894181470701 |
| YBR291C | 1208.95614981942 | 0.59789543017841 | 0.347277072271716 | 1.72166687039623 | 0.0851298820223783 | 0.334308124519035 | 888.336691861883 | 1642.55650371681 | 1838.02048928132 | 984.950467150088 | 911.590078536393 | 988.282668370056 |
| YBR292C | 99.3693931741902 | -0.523924998158686 | 0.426801305580463 | -1.2275618450748 | 0.219611492266969 | 0.540842698179322 | 43.3004937388884 | 103.506936468276 | 98.8452135147885 | 129.73530916097 | 124.6618910819 | 96.1665150803182 |
| YBR293W | 1064.34572967569 | 0.78246121483995 | 0.360541918894212 | 2.17023645194925 | 0.0299889379551441 | 0.193899229883204 | 801.059134169436 | 1637.62760198022 | 1600.29149475208 | 779.44973743911 | 889.329026557482 | 678.317383155816 |
| YBR294W | 44.2965018394875 | -0.340278979131307 | 0.406430611609546 | -0.837237573675208 | 0.402459041199306 | 0.708308196207474 | 29.7690894454858 | 43.127890195115 | 45.0433884371188 | 51.8941236643882 | 47.861261754658 | 48.0832575401591 |
| YBR295W | 1431.65886849463 | 0.78527969222571 | 0.358609400507837 | 2.1897911519153 | 0.0285393867293127 | 0.1889912383872 | 1160.99448837395 | 2272.22370056549 | 2003.17958021742 | 884.275867241175 | 1325.64564534413 | 943.633929225622 |
| YBR296C | 181.73366240734 | 2.55888341811773 | 0.410574418149349 | 6.23244728605306 | 4.59204086427998e-10 | 1.75967005919209e-07 | 171.848834526213 | 412.795520438958 | 347.835055153306 | 48.7804762445249 | 56.7656825462223 | 52.3764055348162 |
| YBR296C-A | 2.18533313382662 | 0.111041304099191 | 1.45211437342716 | 0.0764687039335068 | 0.939046211694445 | NA | 0.676570214670132 | 1.23222543414614 | 5.00482093745765 | 3.11364741986329 | 2.22610519789107 | 0.858629598931413 |
| YBR297W | 582.39485721228 | -0.297695617201209 | 0.365314958226423 | -0.814901253007813 | 0.41512883589175 | 0.716134618459117 | 302.426885957549 | 634.596098585263 | 631.858643354028 | 706.797964308967 | 621.083350211608 | 597.606200856263 |
| YBR298C | 480.321504297092 | 0.757150459687418 | 0.409737029591712 | 1.84789366106815 | 0.0646177277320823 | 0.290400859268185 | 348.433660555118 | 739.335260487685 | 723.19662546263 | 332.122391452084 | 486.403985739199 | 252.437102085835 |
| YBR299W | 28.9316494514727 | -0.268140442900794 | 0.50470427699555 | -0.531282287713124 | 0.595223177552247 | 0.82799910756009 | 14.8845447227429 | 33.2700867219458 | 31.2801308591103 | 32.1743566719207 | 34.5046305673116 | 27.4761471658052 |
| YBR300C | 17.9270506589361 | 0.716625260669444 | 0.675302496879151 | 1.0611914867504 | 0.28860288750616 | 0.612185447443212 | 8.79541279071171 | 25.876734117069 | 32.5313360934747 | 8.30305978630211 | 20.0349467810196 | 12.0208143850398 |
| YBR301W | 59.8121341986925 | 0.0629292770884103 | 0.470163849586148 | 0.133845418238349 | 0.893524811725421 | 0.965832153362604 | 33.8285107335066 | 75.1657514829147 | 75.0723140618647 | 40.4774164582228 | 76.8006293272419 | 57.5281831284046 |
| YBR302C | 87.7033958334116 | 0.259787944539431 | 0.48871023714004 | 0.531578683638233 | 0.595017831706609 | 0.82799910756009 | 40.5942128802079 | 135.544797756076 | 111.357265858433 | 66.4244782904169 | 100.174733905098 | 72.1248863102387 |
| YCL001W | 4150.03477204166 | -0.551196938848873 | 0.36034142691872 | -1.52965187367481 | 0.126102922049064 | 0.40681717154439 | 1913.34056708713 | 3941.88916383351 | 4246.59056543281 | 5027.50270060593 | 4881.84869897512 | 4889.03693631546 |
| YCL002C | 2258.81509881951 | 0.454066057917283 | 0.374692057100219 | 1.21183795950026 | 0.225574414542623 | 0.548365637869472 | 1403.88319544052 | 3160.65823858486 | 3270.65048262857 | 1942.91598999469 | 1905.54604939476 | 1869.23663687369 |
| YCL004W | 224.391114045623 | 0.120630916544332 | 0.557874275695203 | 0.216233158257757 | 0.828806008327489 | 0.939195669606348 | 474.275720483762 | 118.29364167803 | 107.603650155339 | 179.553667878783 | 197.01031001336 | 269.609694064464 |
| YCL005W | 1297.6590802581 | 0.174875842851567 | 0.330404914471588 | 0.529277366019944 | 0.59661305445465 | 0.828750365770883 | 891.719542935234 | 1546.44291985341 | 1691.62947686068 | 1262.06508751792 | 1214.34038544958 | 1179.75706893176 |
| YCL005W-A | 5326.98589500326 | -0.786207981136514 | 0.355226405454289 | -2.21325883736333 | 0.0268798057460865 | 0.183062942450835 | 2274.62906172098 | 4696.01112953095 | 4762.08712199095 | 6629.99323936223 | 7198.11115738077 | 6401.08366003368 |
| YCL007C | 6.42026309353494 | 1.12562510248772 | 0.924282498866457 | 1.21783665044853 | 0.223286053653948 | NA | 5.41256171736105 | 11.0900289073153 | 10.0096418749153 | 1.03788247328776 | 6.67831559367321 | 4.29314799465706 |
| YCL008C | 531.24772849634 | 0.8426495230357 | 0.579852013064314 | 1.45321479282721 | 0.146164126195393 | 0.440892991564844 | 1460.71509347281 | 295.734104195074 | 289.028409138179 | 351.842158444552 | 372.872620646754 | 417.293985080667 |
| YCL009C | 26423.4237185495 | 0.536613990853564 | 0.305968326718585 | 1.75382202664106 | 0.0794610075703786 | 0.322102871307854 | 22307.8731181036 | 33104.9685137703 | 38433.2711839716 | 20447.3226062422 | 21119.0600123926 | 23128.0468768165 |
| YCL010C | 197.095617111227 | 0.421384612818751 | 0.363329669545327 | 1.1597858587934 | 0.246136002977023 | 0.56978056082875 | 317.988000894962 | 155.260404702414 | 202.695247967035 | 168.136960672618 | 173.636205435503 | 164.856882994831 |
| YCL011C | 3578.3549333257 | 0.579455441998531 | 0.281767997610736 | 2.05649842037438 | 0.0397344983854529 | 0.22284687967803 | 3460.65664803772 | 4256.10664954078 | 5146.20712894082 | 2925.79069219821 | 3053.1032789076 | 2628.26520232905 |
| YCL012C | 854.306697135511 | -0.457692903091814 | 0.385397989761322 | -1.187585081529 | 0.234996918222819 | 0.561647520143354 | 384.968452147305 | 850.235549560838 | 925.891873429664 | 913.336576493232 | 1127.52228273183 | 923.8854484502 |
| YCL014W | 120.768722307521 | -0.151782575342469 | 0.330569562937325 | -0.459154720700183 | 0.646123063467538 | 0.859054305981825 | 137.343753578037 | 94.881358429253 | 110.106060624068 | 128.697426687683 | 140.244627467137 | 113.339107058946 |
| YCL016C | 176.616890927744 | 0.947383245685904 | 0.740970319934958 | 1.27857111168645 | NA | NA | 579.144103757633 | 52.9856936682841 | 65.0626721869494 | 119.356484428093 | 94.6094709103704 | 148.542920615134 |
| YCL017C | 3641.29500937466 | 0.453426957286667 | 0.273391962936052 | 1.65852336117403 | 0.0972118762760507 | 0.357503238447089 | 3413.29673301081 | 4299.23453973589 | 4914.73416058341 | 3137.51871674891 | 3161.06938100532 | 2921.9165251636 |
| YCL018W | 15524.5367862367 | 0.933000077540903 | 0.367731137646564 | 2.53717997206327 | 0.0111749488770138 | 0.110556981316825 | 11335.2573765834 | 24071.5238560449 | 25723.5284132979 | 10073.687285731 | 11682.6000785323 | 10260.6237072304 |
| YCL021W-A | 407.160195690943 | 0.585072823180044 | 0.33938543755725 | 1.72391846683566 | 0.0847225630698046 | 0.333953621564233 | 317.311430680292 | 598.861560995025 | 550.530303120341 | 332.122391452084 | 335.028832282606 | 309.106655615309 |
| YCL022C | 1.41457832072299 | -2.70161714668206 | 1.99835563382556 | -1.35192009918185 | 0.176400877024564 | NA | 0 | 0 | 1.25120523436441 | 4.15152989315105 | 2.22610519789107 | 0.858629598931413 |
| YCL023C | 7.76756237994943 | 0.801341033345244 | 0.793175961707912 | 1.01029414913149 | 0.312354383481899 | NA | 7.44227236137145 | 14.7867052097537 | 7.50723140618647 | 7.26517731301435 | 4.45221039578214 | 5.15177759358848 |
| YCL024W | 20.6114472371545 | -0.556073452382391 | 0.520643003082114 | -1.0680513309322 | 0.28549735790654 | 0.610456523844073 | 20.9736766547741 | 13.5544797756076 | 15.0144628123729 | 25.9470618321941 | 26.7132623746928 | 21.4657399732853 |
| YCL025C | 16991.1710232416 | 0.556566195905197 | 0.378621357511661 | 1.46998098459899 | 0.14156690411284 | 0.433655580769244 | 10783.8526516272 | 25120.1477005033 | 24782.6220770559 | 12829.26525231 | 15266.629447137 | 13164.5090108164 |
| YCL026C-A | 33.1310094623404 | 0.0504136117410025 | 0.485268707150877 | 0.103888033574207 | 0.917258203544323 | 0.97754067656341 | 25.709668157465 | 46.8245664975534 | 28.7777203903815 | 31.1364741986329 | 42.2959987599303 | 24.0416287700796 |
| YCL026C-B | 195.409096804506 | -0.85495277826256 | 0.304009545596205 | -2.81225636052276 | 0.0049195277075581 | 0.0706936131576099 | 164.406562164842 | 119.525867112176 | 132.627754842628 | 241.826616276049 | 239.30630877329 | 274.761471658052 |
| YCL027W | 33.7881058071939 | 1.2248664164771 | 0.67861362974701 | 1.80495404569716 | 0.0710818734276173 | 0.303855444880757 | 98.1026811271691 | 23.4122832487767 | 20.0192837498306 | 24.9091793589063 | 12.2435785884009 | 24.0416287700796 |
| YCL028W | 970.96266256685 | 0.622217214682336 | 0.698605128988104 | 0.890656522352745 | NA | NA | 2870.68742084537 | 369.667630243843 | 290.279614372543 | 698.494904522665 | 685.640400950449 | 911.006004466229 |
| YCL029C | 33.3067295603137 | -0.546520924814683 | 0.466184610128216 | -1.17232725607216 | 0.241065692713287 | 0.565569633353458 | 34.5050809481767 | 22.1800578146306 | 23.7728994529238 | 45.6668288246616 | 44.5221039578214 | 29.193406363668 |
| YCL030C | 3430.2748577383 | -0.157046867115714 | 0.24409576970463 | -0.643382174569228 | 0.519976146998508 | 0.787361141450849 | 3309.78149016628 | 3002.93338301415 | 3418.29270028357 | 3639.85383382019 | 3767.68304743063 | 3443.10469171496 |
| YCL031C | 16.1048070163726 | 0.179980131679687 | 0.637449987168193 | 0.28234392548854 | 0.777679807381465 | 0.920342502126552 | 28.4159490161455 | 9.85780347316914 | 12.5120523436441 | 15.5682370993165 | 12.2435785884009 | 18.0312215775597 |
| YCL032W | 84.601946293866 | 0.761574196959266 | 0.676377185708592 | 1.12596079975912 | 0.260182112236374 | 0.58657467942951 | 240.182426207897 | 40.6634393268227 | 37.5361570309323 | 44.6289463513738 | 87.9311553166972 | 56.6695535294732 |
| YCL033C | 2197.46675935265 | -0.424321074285065 | 0.368341096593195 | -1.15197863667571 | 0.249329852171304 | 0.572800955347985 | 1057.47924552942 | 2146.53670628258 | 2427.33815466696 | 2255.31861445431 | 2612.33444972517 | 2685.79338545746 |
| YCL034W | 1571.00404353794 | 0.291845368047745 | 0.290588805558232 | 1.00432419441313 | 0.315222375466273 | 0.635421652543671 | 2054.74374195319 | 1322.17789083881 | 1810.4939741253 | 1324.33803591519 | 1420.2551162545 | 1494.01550214066 |
| YCL035C | 3043.43281446576 | -0.833670909052789 | 0.243316222186966 | -3.42628576738377 | 0.000611896243163126 | 0.0188084470893136 | 2087.21911225736 | 2126.82109933624 | 2349.76343013636 | 3769.58914298116 | 3866.74472873679 | 4060.45937334665 |
| YCL036W | 979.67530430764 | 0.427854732729586 | 0.371337924275239 | 1.15219778201931 | 0.249239808912697 | 0.572800955347985 | 1579.11488104009 | 800.946532194993 | 990.954545616614 | 900.881986813779 | 603.27450862848 | 1002.87937155189 |
| YCL037C | 121.631920052448 | -0.315251622135838 | 0.304410260605001 | -1.03561431046802 | 0.300382113051173 | 0.625627510682729 | 100.80896198585 | 109.668063639007 | 115.110881561526 | 139.07625142056 | 126.887996279791 | 138.239365427957 |
| YCL038C | 1105.53433898667 | 0.704281204050837 | 0.38678466639499 | 1.82086123169013 | 0.0686279536418188 | 0.300208125976541 | 723.930129697041 | 1686.91661934607 | 1700.38791350124 | 790.866444645276 | 958.338287692105 | 772.766639038271 |
| YCL039W | 747.457515969567 | -0.058855762230419 | 0.464822487703086 | -0.126619868417412 | 0.899241270790214 | 0.967041875491516 | 1317.95877817742 | 395.544364360912 | 481.714015230298 | 808.510446691168 | 675.62292755994 | 805.394563797665 |
| YCL040W | 11182.6887239762 | 0.872663434842847 | 0.410695977184728 | 2.12484047402863 | 0.0335999263146886 | 0.206338009035075 | 7457.83347630886 | 17608.5014539484 | 18330.1566834386 | 6456.66686632318 | 10423.7375891249 | 6819.23627471328 |
| YCL041C | 11.8865047877953 | -0.291871898702667 | 0.708413481696047 | -0.412007826282304 | 0.680333686882726 | 0.87491979660087 | 4.05942128802079 | 13.5544797756076 | 15.0144628123729 | 12.4545896794532 | 13.3566311873464 | 12.8794439839712 |
| YCL042W | 70.7938680990166 | 0.151843722999081 | 0.378657632857829 | 0.40100531409621 | 0.688416211435399 | 0.879336974073483 | 68.3335916816833 | 66.5401734438917 | 88.8355716398732 | 50.8562411911004 | 82.3658923219696 | 67.8317383155816 |
| YCL043C | 34609.2177291764 | 0.173091357797404 | 0.254882005669923 | 0.679103875310682 | 0.497072047189297 | 0.77425856189831 | 33415.1263323431 | 34669.8948151359 | 41964.172355348 | 30658.0103784473 | 33998.1916347914 | 32949.910858993 |
| YCL044C | 723.256083104381 | -0.701606829116545 | 0.492142649498917 | -1.42561679998857 | 0.153978984185951 | 0.452721841995317 | 1034.47585823063 | 287.108526156051 | 329.06697663784 | 800.207386904866 | 832.56334401126 | 1056.11440668564 |
| YCL045C | 3296.13964348705 | -0.129420719089806 | 0.363326856924045 | -0.35621016344757 | 0.721683181164852 | 0.896293267442601 | 4714.34125582148 | 2619.7112729947 | 2109.5320251384 | 3378.30745055167 | 3606.29042058353 | 3348.65543583251 |
| YCL046W | 1.65784064269189 | -0.20161851194072 | 1.78497974583334 | -0.112952828967027 | 0.910067952824811 | NA | 2.0297106440104 | 0 | 2.50241046872882 | 2.07576494657553 | 3.3391577968366 | 0 |
| YCL047C | 562.548507525018 | 0.0467140205572067 | 0.290954430130252 | 0.160554422685003 | 0.872444354500863 | 0.957251259156579 | 451.272333184978 | 582.842630351125 | 681.906852728604 | 567.721712888407 | 523.134721504401 | 568.412794492595 |
| YCL048W | 52.3547687198961 | -0.903402532889871 | 0.39849410653038 | -2.26704113833864 | 0.0233877127664978 | 0.168251030014805 | 27.0628085868053 | 38.1989884585304 | 45.0433884371188 | 70.5760081835679 | 64.557050738841 | 68.690367914513 |
| YCL048W-A | 87.3237735649154 | -0.531626508588081 | 0.532913710355034 | -0.997584596264006 | 0.318480833545152 | 0.638537064188392 | 23.0033872987845 | 108.435838204861 | 83.8307507024156 | 104.826129802064 | 117.983575488227 | 85.8629598931413 |
| YCL049C | 2152.12505474976 | -0.110979320841743 | 0.32550350564757 | -0.340946622436388 | 0.733143765691349 | 0.901172486135829 | 1388.99865071778 | 2310.42268902402 | 2509.91770013501 | 2048.78000227005 | 2505.4814002264 | 2149.14988612533 |
| YCL050C | 4457.35733386483 | -0.0425066614703706 | 0.265548836458325 | -0.160070976161259 | 0.872825164005955 | 0.957251259156579 | 5196.05924866661 | 4206.81763217493 | 3771.13257637434 | 4515.82664127506 | 4598.020286244 | 4456.28761845403 |
| YCL051W | 42.4885039639418 | 0.892911168640691 | 0.725338552838302 | 1.2310267600511 | 0.218312855911781 | 0.53944178459454 | 127.871770572655 | 22.1800578146306 | 15.0144628123729 | 29.0607092520574 | 25.6002097757473 | 35.2038135561879 |
| YCL052C | 2112.98027267507 | -0.0186148534308543 | 0.262145957198236 | -0.0710095003173274 | 0.943390194420014 | 0.984368137862999 | 2245.53654249017 | 1919.80722639969 | 2132.05371935696 | 1987.54493634607 | 2438.69824428967 | 1954.2409671679 |
| YCL054W | 70.821136252685 | 0.829293668109822 | 0.395417275335631 | 2.0972621072408 | 0.0359703792676372 | 0.213443621846434 | 122.459208855294 | 82.5591040877915 | 66.3138774213138 | 51.8941236643882 | 46.7482091557125 | 54.9522943316104 |
| YCL054W-A | 283.699905981543 | -0.900854009506294 | 0.396085630342923 | -2.27439205185594 | 0.0229424249063336 | 0.165878060832209 | 104.86838327387 | 255.070664868251 | 235.226584060509 | 414.115106841818 | 360.629042058353 | 332.289654786457 |
| YCL055W | 114.866562293757 | 0.122393921401561 | 0.474042355232623 | 0.258191952787637 | 0.796258768179964 | 0.929585587537483 | 209.060196333071 | 80.0946532194992 | 68.8162878900426 | 118.318601954805 | 101.287786504044 | 111.621847861084 |
| YCL056C | 174.725831552759 | 0.497408622454887 | 0.511859837951055 | 0.971767240903261 | 0.331166356572134 | 0.646462981596975 | 66.980451252343 | 283.411849853613 | 264.004304450891 | 128.697426687683 | 174.749258034449 | 130.511699037575 |
| YCL057C-A | 4533.03002575527 | -0.501385649861431 | 0.380491471881421 | -1.31773163635501 | 0.187593500142079 | 0.501062936253101 | 1972.8787459781 | 4683.68887518949 | 4604.43526246103 | 4966.26763468195 | 5635.38530846124 | 5335.5243277598 |
| YCL057W | 3393.88706697083 | 0.109002717615039 | 0.250006822948025 | 0.435998971266878 | 0.662837475957646 | 0.866909384383846 | 3319.93004338634 | 3456.39234277993 | 3789.9006548898 | 3294.23897021536 | 3521.69842306367 | 2981.16196748986 |
| YCL058C | 1233.40937758239 | -0.655325284890228 | 0.401239406784222 | -1.63325255149389 | 0.102415891561677 | 0.366753015804682 | 464.12716726371 | 1195.25867112176 | 1216.17148780221 | 1445.77028528985 | 1510.41237676909 | 1568.71627724769 |
| YCL058W-A | 20.4457155155293 | -0.446791393144086 | 0.727986720333875 | -0.613735636467619 | 0.539390035372845 | 0.798710611079734 | 32.4753703041663 | 4.92890173658457 | 13.7632575780085 | 38.4016515116473 | 13.3566311873464 | 19.7484807754225 |
| YCL059C | 71.4189543187986 | 1.38951733381659 | 0.791885758892908 | 1.75469418184663 | NA | NA | 262.509243292011 | 27.1089595512151 | 20.0192837498306 | 42.5531814047983 | 33.391577968366 | 42.9314799465706 |
| YCL061C | 3.43197358083039 | -0.396709731292611 | 1.22780741129675 | -0.323104199927922 | 0.74661632963408 | NA | 6.08913193203119 | 2.46445086829228 | 0 | 4.15152989315105 | 4.45221039578214 | 3.43451839572565 |
| YCL063W | 229.639047344037 | 0.0833909578914051 | 0.42155570917288 | 0.197817171199089 | 0.843188110972957 | 0.944581029404123 | 377.526179785934 | 173.743786214606 | 156.400654295551 | 247.016028642488 | 175.862310633394 | 247.285324492247 |
| YCL064C | 1917.38449831689 | 0.89391290050517 | 0.247766357019569 | 3.60788652365167 | 0.000308701443705694 | 0.0121535335508242 | 2455.27330903791 | 2416.39407636059 | 2607.51170841543 | 1267.25449988436 | 1464.77722021232 | 1293.09617599071 |
| YCL068C | 2.99856254378623 | 1.09125313651599 | 1.61638019370387 | 0.675121571500714 | 0.499598529560038 | NA | 10.8251234347221 | 0 | 1.25120523436441 | 0 | 3.3391577968366 | 2.57588879679424 |
| YCR001W | 1.19390755301505 | -1.4172228776857 | 1.94263703374975 | -0.72953560189786 | 0.465674098292109 | NA | 0.676570214670132 | 0 | 1.25120523436441 | 1.03788247328776 | 3.3391577968366 | 0.858629598931413 |
| YCR002C | 3014.5392925988 | 0.169177745306278 | 0.338932593100813 | 0.499148647105641 | 0.617674668656312 | 0.841649602952482 | 2000.61812477958 | 3726.24971285793 | 3847.45609567056 | 2936.16951693108 | 3008.58117494978 | 2568.16113040386 |
| YCR003W | 317.062396156121 | -1.80790568144445 | 0.441596039622721 | -4.09402603109629 | 4.23946683322434e-05 | 0.00286687710086747 | 232.063583631855 | 103.506936468276 | 85.08195593678 | 590.555127300738 | 363.96819985519 | 527.198573743887 |
| YCR004C | 5164.82124176345 | 0.19459339273924 | 0.280206882036737 | 0.69446328842747 | 0.487391715865612 | 0.766813881410433 | 4357.11218247565 | 5771.74393354053 | 6409.92441564888 | 4463.93251761067 | 5158.99879611255 | 4827.2156051924 |
| YCR005C | 7829.52982124376 | -0.0978086515424877 | 0.390573109995923 | -0.250423413796994 | 0.802259924742692 | 0.930747663909662 | 5309.72304473119 | 9393.25448449604 | 7990.19662665113 | 5533.98934757036 | 11941.9413340866 | 6808.07408992717 |
| YCR006C | 104.218271972271 | 0.131863098359233 | 0.35924999529765 | 0.367051078873307 | 0.713580917567488 | 0.891399851461647 | 86.6009874777769 | 104.739161902422 | 136.381370545721 | 112.091307115078 | 79.026734525133 | 106.470070267495 |
| YCR007C | 150.248370223435 | 0.568523118548441 | 0.322540175021033 | 1.76264280414484 | 0.0779607529619946 | 0.31959809218616 | 161.023711091491 | 197.156069463383 | 180.173553748475 | 128.697426687683 | 139.131574868192 | 95.3078854813868 |
| YCR008W | 1527.98499896388 | 0.396496871359614 | 0.25402317106805 | 1.56086891480225 | 0.118554683075242 | 0.396495360918639 | 1612.26682155892 | 1828.62254427288 | 1769.20420139128 | 1437.46722550355 | 1323.41954014624 | 1196.92966091039 |
| YCR009C | 1113.3436242248 | 0.118365196095232 | 0.293977464359089 | 0.402633570410864 | 0.687217809655201 | 0.878614641799404 | 1460.71509347281 | 1095.44841095592 | 919.635847257842 | 1048.26129802064 | 1093.01765216452 | 1062.98344347709 |
| YCR010C | 25.6360243266446 | 0.552216397423255 | 0.54092618755912 | 1.0208719971852 | 0.307315087870347 | 0.632461637687716 | 16.2376851520832 | 39.4312138926766 | 36.2849517965679 | 24.9091793589063 | 18.9218941820741 | 18.0312215775597 |
| YCR011C | 3044.75881381927 | 0.694925819492861 | 0.322622225168082 | 2.15399239507078 | 0.0312407683169096 | 0.198861501977404 | 2517.51776878756 | 4309.09234320906 | 4466.80268668095 | 2304.09909069884 | 2532.19466260109 | 2138.84633093815 |
| YCR012W | 424064.530736507 | 1.05108320180553 | 0.397314458562761 | 2.64546929806606 | 0.0081577689574986 | 0.0933159761227517 | 280136.603665027 | 721780.976952839 | 714241.749600284 | 260758.630471291 | 300947.161702894 | 266522.062026706 |
| YCR013C | 46.5364317537538 | 0.970208737068216 | 0.53771821776392 | 1.80430698647851 | 0.0711831951531268 | 0.303855444880757 | 25.0330979427949 | 69.004624312184 | 91.337982108602 | 25.9470618321941 | 37.8437883641482 | 30.0520359625994 |
| YCR014C | 26.7662474589103 | 0.408626026080733 | 0.641512523424081 | 0.636972796570963 | 0.524142563277239 | 0.790546169960003 | 57.5084682469612 | 19.7156069463383 | 13.7632575780085 | 25.9470618321941 | 14.469683786292 | 29.193406363668 |
| YCR015C | 167.600876789226 | -0.0506319296448101 | 0.328112503071234 | -0.154312710338313 | 0.877363175953377 | 0.958809816585114 | 130.578051431335 | 171.279335346314 | 192.685606092119 | 189.932492611661 | 180.314521029177 | 140.815254224752 |
| YCR016W | 21.6429669053602 | 0.17260924066419 | 0.534572149789429 | 0.322892318150471 | 0.746776794204572 | 0.906988469670736 | 29.7690894454858 | 19.7156069463383 | 18.7680785154662 | 16.6061195726042 | 27.8263149736384 | 17.1725919786283 |
| YCR017C | 6258.73750646334 | 0.775522603726287 | 0.354321971173404 | 2.18875109877606 | 0.0286149357611988 | 0.18927347612816 | 4708.25212388945 | 9090.12702769609 | 9907.04304569741 | 4698.49395657371 | 5077.74595638953 | 4070.76292853383 |
| YCR018C | 434.092434628855 | -1.17993372714425 | 0.288048023561834 | -4.09630905483705 | 4.19789429954154e-05 | 0.00286687710086747 | 299.044034884198 | 273.554046380444 | 223.96573695123 | 640.37348601855 | 645.57050738841 | 522.046796150299 |
| YCR018C-A | 47.5137764663385 | -0.226938329464273 | 0.471086943373178 | -0.481733430859494 | 0.62999532417722 | 0.850071340307741 | 23.0033872987845 | 54.2179191024303 | 55.0530303120341 | 42.5531814047983 | 57.8787351451678 | 52.3764055348162 |
| YCR019W | 469.430380734756 | 0.621581688228697 | 0.29857549994358 | 2.08182415618882 | 0.0373585344471688 | 0.218450514753129 | 460.74431619036 | 621.041618809656 | 625.602617182206 | 360.145218230854 | 429.638303192976 | 319.410210802485 |
| YCR020C | 359.402671663759 | -1.74363941937375 | 0.355234520091505 | -4.90841773745583 | 9.18141258093792e-07 | 0.000155219880927151 | 101.48553220052 | 186.066040556067 | 210.202479373221 | 536.585238689774 | 536.491352691748 | 585.585386471223 |
| YCR020C-A | 1148.72645486343 | -0.808469819336471 | 0.369687403827014 | -2.18690117912369 | 0.028749738843794 | 0.189721712869876 | 470.216299195742 | 927.865751912045 | 1108.56783764687 | 1545.40700272548 | 1464.77722021232 | 1375.52461748812 |
| YCR020W-B | 15.0603294044372 | 0.283605840325364 | 0.682611473347469 | 0.41547183339094 | 0.677796530416546 | 0.87424576384305 | 25.709668157465 | 17.251156078046 | 6.25602617182206 | 17.644002045892 | 8.90442079156428 | 14.596703181834 |
| YCR021C | 19194.6713657129 | 1.40139062130102 | 0.377970527267867 | 3.70767168390317 | 0.000209173601785304 | 0.00932038653536377 | 17234.9496485069 | 32681.082964424 | 33626.1406735436 | 8699.53089109803 | 14259.3168450912 | 8667.00717161368 |
| YCR022C | 40.0517346311646 | -0.0984482165707077 | 0.517213489816522 | -0.190343481964539 | 0.849039982748901 | 0.947443568402385 | 17.5908255814234 | 56.6823699707225 | 42.54097796839 | 44.6289463513738 | 44.5221039578214 | 34.3451839572565 |
| YCR023C | 1925.38076974207 | 0.39386660913416 | 0.326704069392149 | 1.20557607337726 | 0.227980989391396 | 0.551065907073905 | 1453.94939132611 | 2480.46979893618 | 2626.2797869309 | 1833.93833029948 | 1647.31784643939 | 1510.32946452035 |
| YCR024C | 325.34769185081 | -0.614819612496111 | 0.277575623817041 | -2.21496255341701 | 0.0267626349000868 | 0.182915131279072 | 288.895481664146 | 258.76734117069 | 222.714531716865 | 407.887812002091 | 384.003146636209 | 389.817837914861 |
| YCR024C-A | 10829.4717611961 | 0.431405689261826 | 0.358667898872434 | 1.20279983410297 | 0.229053789024261 | 0.552497347591882 | 7075.57130502024 | 15015.8991405049 | 15219.6604708087 | 9156.19917934465 | 9199.37973028484 | 9310.12074121331 |
| YCR024C-B | 28645.1796327058 | 0.283656022485816 | 0.368327915108279 | 0.770118176903395 | 0.441229794575425 | 0.737703610738415 | 17260.6593166644 | 38078.2303659841 | 39018.8352336542 | 26870.7772334202 | 24693.0719076067 | 25949.5037389052 |
| YCR025C | 12.1780660562277 | 0.0600284471612728 | 0.728212983027746 | 0.0824325417979889 | 0.934302759485051 | 0.98165448197831 | 5.41256171736105 | 9.85780347316914 | 22.5216942185594 | 13.4924721527409 | 8.90442079156428 | 12.8794439839712 |
| YCR026C | 130.322131694475 | 2.0238553259735 | 0.828692511259597 | 2.4422271210069 | NA | NA | 552.757865385498 | 33.2700867219458 | 41.2897727340256 | 48.7804762445249 | 42.2959987599303 | 63.5385903209245 |
| YCR027C | 581.294049990306 | 0.639142068012982 | 0.316663696136202 | 2.01836230616748 | 0.0435535420710944 | 0.233749542319935 | 498.632248211887 | 755.354191131585 | 870.83884311763 | 459.781935666479 | 465.255986359233 | 437.90109545502 |
| YCR028C | 1286.53994997281 | 1.00056700269886 | 0.348320686592466 | 2.87254544795245 | 0.00407179507218908 | 0.0620813742040924 | 1039.88841994799 | 2031.93974090699 | 2075.74948381056 | 897.768339393916 | 877.085447969081 | 796.808267808351 |
| YCR028C-A | 5997.12461052771 | -0.433166408608951 | 0.372149958192197 | -1.16395662305904 | 0.244441590928047 | 0.567946943988665 | 2763.78932692749 | 6018.18902036976 | 6530.04011814786 | 7002.59304727254 | 6928.75242843595 | 6739.38372201266 |
| YCR030C | 1567.4556653278 | 0.581767090061944 | 0.248546332272762 | 2.34067863622102 | 0.0192487275742602 | 0.152937438798529 | 1867.33379248956 | 1914.8783246631 | 1855.53736256242 | 1154.12531029599 | 1371.2808019009 | 1241.57840005482 |
| YCR031C | 127837.25981706 | 0.187402004585586 | 0.393757538875021 | 0.475932486577909 | 0.634122476987321 | 0.852218519368363 | 67471.6412281936 | 167737.919448578 | 173176.814077909 | 111585.858350587 | 126372.652926479 | 120678.672870612 |
| YCR032W | 367.174354759713 | -0.238862660889421 | 0.271847165386431 | -0.878665262335466 | 0.379582798839836 | 0.690456306244106 | 372.113618068572 | 341.326445258481 | 296.535640544365 | 409.963576948667 | 379.550936240427 | 403.555911497764 |
| YCR033W | 494.967085372717 | -0.154999879657245 | 0.269949983051529 | -0.574179994031184 | 0.565846005863275 | 0.812511326930328 | 437.064358676905 | 485.49682105358 | 482.965220464663 | 456.668288246616 | 582.126509248515 | 525.481314546024 |
| YCR034W | 15296.061756286 | 0.805342761586387 | 0.372143272435705 | 2.16406642612497 | 0.0304592517560177 | 0.195446052614534 | 10564.6439020741 | 22993.326601167 | 24816.4046183837 | 11571.3516946853 | 11355.3626144423 | 10475.2811069632 |
| YCR035C | 148.700193472323 | 1.8580367324397 | 0.729426457872164 | 2.54725711192304 | NA | NA | 571.025261181591 | 61.6112717073071 | 66.3138774213138 | 63.3108308705536 | 50.0873669525491 | 79.8525527006214 |
| YCR036W | 1804.31884786237 | 0.246894227041351 | 0.356156238036477 | 0.693218876082313 | 0.488172204030021 | 0.7672993789348 | 1152.19907558323 | 2237.72138840939 | 2486.14480068209 | 1644.00583768782 | 1769.7536323234 | 1536.0883524883 |
| YCR037C | 1541.4094223869 | 0.689445209801315 | 0.302188321165227 | 2.28150845520052 | 0.0225183769571434 | 0.164467129288005 | 1365.995263419 | 2236.48916297525 | 2107.02961466967 | 1159.31472266243 | 1199.87070166329 | 1179.75706893176 |
| YCR038C | 51.0081230760005 | 0.543947060247029 | 0.6774985914385 | 0.802875558888015 | 0.422046664081832 | 0.722001257482848 | 134.637472719356 | 18.4833815121921 | 27.5265151560171 | 36.3258865650717 | 47.861261754658 | 41.2142207487078 |
| YCR041W | 22.2750366074564 | -1.0247452150051 | 0.53524952252735 | -1.91451869058461 | 0.0555539132819556 | 0.267167980202198 | 8.79541279071171 | 17.251156078046 | 18.7680785154662 | 30.0985917253451 | 27.8263149736384 | 30.9106655615309 |
| YCR042C | 283.503601169585 | 0.68441738113325 | 0.289574716339826 | 2.3635260349528 | 0.0181019547085358 | 0.148349346684515 | 335.578826476385 | 393.079913492619 | 320.308539997289 | 228.334144123308 | 185.879784023904 | 237.840398904001 |
| YCR043C | 2367.89704398525 | -0.0332289209954957 | 0.360380851627051 | -0.0922050126844238 | 0.92653515546728 | 0.97960780060512 | 1342.99187612021 | 2701.03815164834 | 2979.11966302166 | 2521.01652761598 | 2385.27171954028 | 2277.94432596504 |
| YCR044C | 2059.04317648296 | 0.274107798833145 | 0.354118722384929 | 0.774056217607123 | 0.438897547104185 | 0.73650455133502 | 1319.31191860676 | 2757.72052161907 | 2686.33763818039 | 1938.76446010154 | 1800.91910509388 | 1851.20541529613 |
| YCR045C | 25.1154436129804 | 0.754959828944212 | 0.538278080051978 | 1.40254611310071 | 0.160752230444211 | 0.46177102478427 | 16.9142553667533 | 39.4312138926766 | 38.7873622652968 | 19.7197669924675 | 17.8088415831286 | 18.0312215775597 |
| YCR045W-A | 386.409617692684 | 0.618060363535455 | 0.393190517128907 | 1.57191065554825 | 0.11597127301087 | 0.39235013376485 | 245.594987925258 | 609.95158990234 | 549.279097885977 | 318.629919299343 | 281.60230753322 | 313.399803609966 |
| YCR046C | 3193.9304870826 | -0.44793221304438 | 0.30852748453696 | -1.45183893006044 | 0.146546396466091 | 0.440892991564844 | 1957.31763104069 | 2883.40751590197 | 3266.89686692548 | 3358.5876835592 | 3526.15063345945 | 4171.2225916088 |
| YCR047C | 2635.30127739182 | 1.03682499998918 | 0.316514505860218 | 3.27575823790862 | 0.00105378725839519 | 0.0265665314090157 | 2559.46512209711 | 3778.00318109207 | 4294.13636433866 | 1664.76348715357 | 1495.9426929828 | 2019.49681668668 |
| YCR047W-A | 5.4551022113513 | 0.645016320493022 | 0.931751698295898 | 0.692262028255712 | 0.488772791521379 | NA | 4.05942128802079 | 8.625578039023 | 7.50723140618647 | 2.07576494657553 | 4.45221039578214 | 6.01040719251989 |
| YCR048W | 4163.90084875247 | 1.03196188256741 | 0.385352211133548 | 2.67797057536481 | 0.00740697249762346 | 0.0876034525027565 | 2889.63138685613 | 7008.89826942326 | 6880.3775837699 | 2596.78194816598 | 2956.26770279934 | 2651.4482015002 |
| YCR049C | 2.02284980657217 | 0.991574647584184 | 1.66067770363445 | 0.597090359805571 | 0.550447053791483 | NA | 0.676570214670132 | 1.23222543414614 | 6.25602617182206 | 3.11364741986329 | 0 | 0.858629598931413 |
| YCR050C | 2.41643027855655 | -0.011247333741199 | 1.70369558747222 | -0.00660172734137719 | 0.994732621940897 | NA | 0 | 3.69667630243843 | 3.75361570309323 | 1.03788247328776 | 0 | 6.01040719251989 |
| YCR051W | 4921.11754206651 | 1.057260710967 | 0.306608878083945 | 3.44823906461554 | 0.000564254374425751 | 0.0180790272479556 | 4738.02121333493 | 7135.81748914031 | 8070.27376165045 | 3054.48811888589 | 3068.68601529284 | 3459.41865409466 |
| YCR052W | 337.977915950601 | 0.727455329217181 | 0.705845022711885 | 1.03061622000573 | NA | NA | 1032.44614758662 | 115.829190809737 | 115.110881561526 | 264.66003068838 | 267.132623746928 | 232.688621310413 |
| YCR053W | 8109.41902287185 | -0.282391660313081 | 0.52194725102101 | -0.541034864654769 | 0.588483552009327 | 0.825427881149246 | 14562.4973005599 | 4148.90303677006 | 3241.87276223819 | 9109.4944680467 | 8453.63448899134 | 9140.11208062489 |
| YCR054C | 697.192383108105 | 0.451029209959668 | 0.318216324371313 | 1.41736666354483 | 0.156375753973677 | 0.456731622886533 | 601.470920841747 | 829.287717180354 | 985.949724679156 | 698.494904522665 | 517.569458509674 | 550.381572915035 |
| YCR057C | 3166.26617026011 | 0.620485792419145 | 0.317514339124225 | 1.95419770373389 | 0.0506778407852921 | 0.254185190954502 | 5132.46164848762 | 3100.27919231169 | 3276.90650880039 | 2742.08549442627 | 2157.09593675645 | 2588.76824077821 |
| YCR059C | 4777.04225956936 | 0.830471028280285 | 0.406840981286378 | 2.04126689905831 | 0.0412243053157583 | 0.225888757821714 | 2874.74684213339 | 7832.02485943288 | 7639.8591610291 | 3296.31473516194 | 3346.94916502922 | 3672.35879462965 |
| YCR060W | 1719.24802589772 | -0.951939108055587 | 0.306424515661856 | -3.10660230954258 | 0.00189250808439377 | 0.0385693850151712 | 843.006487478984 | 1217.43872893639 | 1456.40289280017 | 2178.51531143102 | 2320.71466880144 | 2299.41006593832 |
| YCR061W | 802.755802157878 | 1.21295323251825 | 0.401635795985033 | 3.02003268793165 | 0.00252747406036709 | 0.0463665311021237 | 590.645797407025 | 1490.99277531683 | 1283.73657045789 | 445.251581040451 | 595.483140435861 | 410.424948289215 |
| YCR063W | 62.9142420671831 | -0.0107547155448783 | 0.539095577588152 | -0.0199495525320271 | 0.984083615792862 | 0.995606783760712 | 115.016936493922 | 38.1989884585304 | 33.7825413278391 | 71.6138906568557 | 46.7482091557125 | 72.1248863102387 |
| YCR064C | 33.9336746111843 | 0.233461727548723 | 0.613457021921302 | 0.380567373436428 | 0.703524294869117 | 0.887726241505533 | 11.5016936493922 | 48.0567919316995 | 51.2994146089409 | 33.2122391452084 | 20.0349467810196 | 39.496961550845 |
| YCR065W | 348.97748991154 | -0.0597216843370932 | 0.316259269236129 | -0.188837735827762 | 0.850219994980378 | 0.947472766798606 | 429.622086315534 | 288.340751590197 | 306.545282419281 | 347.690628551401 | 306.089464710022 | 415.576725882804 |
| YCR066W | 28.3822680181134 | 0.146916851859549 | 0.538228226306663 | 0.272963855626258 | 0.784880999853586 | 0.925243921158455 | 43.9770639535586 | 25.876734117069 | 18.7680785154662 | 28.0228267787696 | 35.6176831662571 | 18.0312215775597 |
| YCR067C | 2328.10357561046 | -0.257025690541487 | 0.25595041883281 | -1.00420109376488 | 0.315281695033246 | 0.635421652543671 | 2382.20372585353 | 1996.20520331675 | 1984.41150170196 | 2614.42595021188 | 2660.19571147983 | 2331.17936109879 |
| YCR068W | 313.072349314143 | -0.217294716639722 | 0.354486287248743 | -0.612984830319393 | 0.539886370192357 | 0.798710611079734 | 378.879320215274 | 209.478323804844 | 279.018767263264 | 288.531327573998 | 426.29914539614 | 296.227211631337 |
| YCR069W | 3664.54465099315 | -0.141736363320136 | 0.265777641919071 | -0.533289265028902 | 0.593833357582746 | 0.827932882774509 | 2945.78671467375 | 3702.83742960916 | 3806.16632293654 | 3624.28559672087 | 4241.84345458143 | 3666.34838743713 |
| YCR071C | 335.057346238235 | -1.09430975695556 | 0.389821410395901 | -2.80720793617823 | 0.00499729726474815 | 0.0711398381752016 | 315.281720036281 | 139.241474058514 | 185.178374685933 | 496.107822231551 | 355.063779063626 | 519.470907353505 |
| YCR072C | 1320.31218733126 | -0.0423009872298878 | 0.322487469341573 | -0.131170948490663 | 0.895640077107445 | 0.966310161494771 | 1544.60980009191 | 1021.51488490715 | 1336.28719030119 | 1555.78582745836 | 992.842918259417 | 1470.83250296951 |
| YCR073C | 456.726523596618 | -0.233207927247793 | 0.356078955667574 | -0.65493319258527 | 0.512510768336896 | 0.782238952841337 | 611.619474061799 | 347.487572429212 | 299.038051013094 | 495.069939758263 | 490.856196134981 | 496.287908182356 |
| YCR073W-A | 2970.75936562115 | 0.401580995101225 | 0.294144710663481 | 1.36524975817313 | 0.172174564132411 | 0.480730372880155 | 2559.46512209711 | 3642.458383336 | 3943.79889871662 | 2272.9626165002 | 2708.05697323449 | 2697.8141998425 |
| YCR075C | 932.33252851921 | 0.396069592425724 | 0.383542327214218 | 1.0326620149137 | 0.301762095560324 | 0.626183583133841 | 559.523567532199 | 1376.39580994124 | 1243.69800295822 | 765.95726528637 | 837.015554407042 | 811.404970990185 |
| YCR075W-A | 427.335645116939 | -1.39532679193113 | 0.2802460811773 | -4.97893417838147 | 6.39353799651689e-07 | 0.000122037152238988 | 206.35391547439 | 227.961705317036 | 272.762741091442 | 603.009716980191 | 670.057664565212 | 583.86812727336 |
| YCR076C | 912.693449516066 | -0.385697896547842 | 0.30897903575974 | -1.24829794875714 | 0.211921965121009 | 0.531943568048539 | 563.58298882022 | 871.183381941323 | 940.906336242037 | 985.988349623375 | 1012.87786504044 | 1101.621775429 |
| YCR077C | 2181.49268595202 | 0.213635562346264 | 0.418808988629545 | 0.510102619920686 | 0.609979569826861 | 0.836161810915756 | 3881.48332156255 | 1632.69870024364 | 1512.70712834657 | 2257.39437940089 | 1660.67447762674 | 2143.99810853174 |
| YCR079W | 1409.49975550639 | 0.401702507167705 | 0.406161677781073 | 0.989021193142274 | 0.32265277272287 | 0.639299599314394 | 783.468308588013 | 2142.84002998014 | 1888.0686986559 | 1084.58718458571 | 1386.86353828614 | 1171.17077294245 |
| YCR081C-A | 4.12743781235964 | 0.00103252834422082 | 1.13464679972305 | 0.000909999785371839 | 0.999273925321128 | NA | 1.35314042934026 | 2.46445086829228 | 8.75843664055088 | 2.07576494657553 | 6.67831559367321 | 3.43451839572565 |
| YCR081W | 145.054749170697 | 0.426978794146569 | 0.484864786102022 | 0.8806141555034 | 0.378526694266632 | 0.689845097858148 | 297.014324240188 | 110.900289073153 | 90.0867768742376 | 140.114133893848 | 106.853049498771 | 125.359921443986 |
| YCR082W | 1040.50008078676 | -1.20440003535667 | 0.254642069858784 | -4.72977633281331 | 2.24767327611833e-06 | 0.00034257492458938 | 665.74509123541 | 600.093786429171 | 623.100206713477 | 1491.43711411452 | 1314.51511935468 | 1548.10916687334 |
| YCR083W | 766.046928855839 | -0.776675052701733 | 0.296167706330329 | -2.62241640834222 | 0.00873086828593552 | 0.0966956279529043 | 426.239235242183 | 619.80939337551 | 649.37551663513 | 898.806221867203 | 1031.79975922251 | 970.251446792496 |
| YCR084C | 3528.7315223586 | 0.993391973912786 | 0.279049700801768 | 3.55991055019434 | 0.000370981136911611 | 0.0136398100628682 | 3687.30766995222 | 5487.09985825277 | 4919.73898152087 | 2309.28850306527 | 2340.74961558246 | 2428.20450577803 |
| YCR085W | 5.56406557623683 | -1.21461461672802 | 0.935340406449824 | -1.29858029050429 | 0.194088005546838 | NA | 2.70628085868053 | 4.92890173658457 | 2.50241046872882 | 11.4167072061654 | 6.67831559367321 | 5.15177759358848 |
| YCR086W | 41.0147928940559 | -0.863475822940742 | 0.57168891550201 | -1.51039455117388 | 0.130942778158644 | 0.414231749507919 | 54.8021873882807 | 13.5544797756076 | 17.5168732811018 | 57.083536030827 | 56.7656825462223 | 46.3659983422963 |
| YCR087C-A | 368.314729443928 | 0.177953931999883 | 0.379572691409137 | 0.46882701529249 | 0.639193288109383 | 0.85569265290655 | 552.081295170828 | 277.250722682882 | 342.830234215849 | 403.73628210894 | 249.3237821638 | 384.666060321273 |
| YCR087W | 6.13496325758434 | 0.121868339561616 | 0.884446052560371 | 0.137790585653948 | 0.890405924945676 | NA | 3.38285107335066 | 8.625578039023 | 7.50723140618647 | 6.22729483972658 | 3.3391577968366 | 7.72766639038271 |
| YCR088W | 222.077123965357 | 0.26077488781654 | 0.492366732719444 | 0.529635473900169 | 0.596364694064946 | 0.828750365770883 | 441.800350179596 | 133.080346887783 | 150.144628123729 | 159.833900886316 | 241.532413971181 | 206.071103743539 |
| YCR089W | 539.966142718837 | -0.657058483070483 | 0.359869369902613 | -1.82582497434637 | 0.0678766301545167 | 0.29874032934775 | 621.091457067181 | 324.075289180435 | 310.298898122374 | 648.676545804852 | 674.509874960994 | 661.144791177188 |
| YCR090C | 1303.35312748959 | -0.505657293630043 | 0.333317647013562 | -1.51704327136771 | 0.129255759373413 | 0.410249643776022 | 717.16442755034 | 1233.45765958029 | 1282.48536522352 | 1785.15785405495 | 1397.99406427559 | 1403.85939425286 |
| YCR091W | 12.0105641298937 | 0.219606115228256 | 0.783989619603671 | 0.280113549640203 | 0.779390389560561 | 0.921494475945177 | 25.709668157465 | 4.92890173658457 | 7.50723140618647 | 15.5682370993165 | 8.90442079156428 | 9.44492558824554 |
| YCR092C | 260.12759047309 | 0.277908952397762 | 0.286010751653608 | 0.971673095472795 | 0.331213205556218 | 0.646462981596975 | 271.981226297393 | 304.359682234097 | 279.018767263264 | 206.538612184265 | 270.471781543765 | 228.395473315756 |
| YCR093W | 1020.18091684974 | 0.609370214850716 | 0.256093602699136 | 2.37948237842792 | 0.0173369719173342 | 0.144844352588426 | 1342.99187612021 | 1266.72774630223 | 1087.29734866267 | 820.965036370621 | 800.284818641839 | 802.818675000871 |
| YCR094W | 382.745647691718 | 0.390340233661959 | 0.286619193449693 | 1.36187751058783 | 0.173236544698543 | 0.481976601610467 | 519.605924866661 | 383.22211001945 | 399.134469762247 | 320.705684245919 | 318.333043298423 | 355.472653957605 |
| YCR095C | 158.112041487484 | -0.0983281530423467 | 0.351695761282961 | -0.279582991514179 | 0.77979745771608 | 0.921494475945177 | 195.528792039668 | 130.615896019491 | 131.376549608263 | 170.212725619193 | 126.887996279791 | 194.050289358499 |
| YCR095W-A | 15.2925032359797 | 0.363605042201582 | 0.62760282416796 | 0.579355331428963 | 0.562349437678346 | 0.810832039687759 | 10.8251234347221 | 20.9478323804844 | 20.0192837498306 | 12.4545896794532 | 18.9218941820741 | 8.58629598931413 |
| YCR097W | 208.465338248158 | -1.20989709839852 | 0.406633716288118 | -2.9753978825043 | 0.00292608752987183 | 0.0499084603017901 | 148.845447227429 | 129.383670585345 | 98.8452135147885 | 367.410395543868 | 160.279574248157 | 346.027728369359 |
| YCR098C | 2711.36542491291 | 1.52444115947037 | 0.348201522527053 | 4.37804277364103 | 1.19749852039863e-05 | 0.00109257484051608 | 2470.15785376065 | 4771.17688101386 | 4830.90340988099 | 1405.29286883163 | 1559.38669112269 | 1231.27484486765 |
| YCR099C | 251.876137940131 | 0.496248333763722 | 0.344005921944443 | 1.44255753202954 | 0.149145153848166 | 0.443682404727836 | 232.063583631855 | 367.20317937555 | 285.274793435086 | 187.856727665085 | 267.132623746928 | 171.725919786283 |
| YCR100C | 311.987490991247 | 0.558460491873919 | 0.330809875047467 | 1.68816149092824 | 0.0913802332153451 | 0.346781091896701 | 338.961677549736 | 410.331069570665 | 365.351928434408 | 223.144731756869 | 338.367990079443 | 195.767548556362 |
| YCR101C | 143.401974831362 | 0.0853671698157872 | 0.375931169255223 | 0.227081914981704 | 0.820360046488029 | 0.935571696768309 | 106.898093917881 | 168.814884478021 | 167.661501404831 | 120.394366901381 | 185.879784023904 | 110.763218262152 |
| YCR102C | 407.605885530796 | 0.554690924358977 | 0.333264384420406 | 1.66441705231617 | 0.0960291146652824 | 0.355730276849832 | 335.578826476385 | 548.340318195033 | 571.800792104536 | 305.137447146603 | 385.116199235155 | 299.661730027063 |
| YCR102W-A | 20.7392567698071 | 0.812242187998141 | 0.559316333479414 | 1.45220537892272 | 0.146444507518949 | 0.440892991564844 | 16.2376851520832 | 25.876734117069 | 37.5361570309323 | 13.4924721527409 | 16.695788984183 | 14.596703181834 |
| YCR104W | 31.813665315589 | -0.0763738533263256 | 0.519008469726838 | -0.147153385312811 | 0.883010953090628 | 0.961277833023661 | 20.297106440104 | 40.6634393268227 | 32.5313360934747 | 18.6818845191797 | 40.0698935620392 | 38.6383319519136 |
| YCR105W | 103.776068783488 | 0.160655011094796 | 0.401659068245229 | 0.39997854846566 | 0.68917231676626 | 0.879716295086045 | 68.3335916816833 | 110.900289073153 | 150.144628123729 | 107.939777221927 | 104.62694430088 | 80.7111822995528 |
| YCR106W | 283.692016489961 | 0.685196446684022 | 0.290414086487675 | 2.35937744952706 | 0.0183056260162234 | 0.148616862063915 | 297.014324240188 | 349.952023297504 | 402.88808546534 | 229.372026596596 | 223.723572388052 | 199.202066952088 |
| YCR107W | 513.081391659443 | 0.554500111767919 | 0.320170677296313 | 1.73188911754944 | 0.0832933067099389 | 0.33103453139585 | 422.179813954162 | 723.316329843786 | 686.911673666062 | 435.910638780861 | 404.038093417229 | 406.131800294558 |
| YDL001W | 160.840385130166 | 0.270285281248457 | 0.290903821061136 | 0.929122485440485 | 0.352825612772492 | 0.666242320044772 | 167.789413238193 | 189.762716858506 | 170.16391187356 | 154.644488519877 | 143.583785263974 | 139.097995026889 |
| YDL002C | 49.5530016011244 | 0.753495781550782 | 0.44539739359579 | 1.69173819242104 | 0.0906959029214503 | 0.345932349032844 | 79.8352853310756 | 67.7723988780378 | 38.7873622652968 | 34.2501216184962 | 31.165472770475 | 45.5073687433649 |
| YDL003W | 46.5908830416519 | -0.421315662899506 | 0.422314837648035 | -0.997634052466417 | 0.318456842420796 | 0.638537064188392 | 52.7724767442703 | 32.0378612877997 | 33.7825413278391 | 62.2729483972658 | 52.3134721504401 | 46.3659983422963 |
| YDL004W | 7482.44943624464 | -0.308735871215703 | 0.342733193366539 | -0.900805282917323 | 0.367691858185715 | 0.681769945236982 | 4058.06814759145 | 7791.36142010606 | 8206.65513219617 | 8163.98353488155 | 8226.57175880645 | 8448.05662388617 |
| YDL005C | 57.3131338018216 | -0.616814580490016 | 0.408751252302535 | -1.50902187336538 | 0.131293193107532 | 0.414618319068967 | 32.4753703041663 | 57.9145954048687 | 46.2945936714832 | 70.5760081835679 | 56.7656825462223 | 79.8525527006214 |
| YDL006W | 711.040117244621 | 0.644236957806758 | 0.352645410201004 | 1.82686897141111 | 0.0677194725617257 | 0.298731159162496 | 543.285882380116 | 1004.26372882911 | 1054.7660125692 | 600.933952033615 | 601.048403430589 | 461.9427242251 |
| YDL007W | 996.908119003428 | 0.247041258012243 | 0.323617906860941 | 0.763373264503554 | 0.445240819770372 | 0.739025189731476 | 1462.74480411682 | 849.003324126692 | 933.399104835851 | 914.37445896652 | 814.754502428131 | 1007.17251954655 |
| YDL008W | 946.5211773051 | -0.358019127371632 | 0.284121763390164 | -1.26009047353402 | 0.207636726469721 | 0.525586142838902 | 658.979389088708 | 853.932225863277 | 977.191288038605 | 1122.98883609736 | 1056.28691639931 | 1009.74840834334 |
| YDL009C | 22.5449546760628 | -0.867588132983551 | 0.691894994988568 | -1.25393034964487 | 0.209867322585678 | 0.528601985043498 | 4.05942128802079 | 19.7156069463383 | 25.0241046872882 | 31.1364741986329 | 16.695788984183 | 38.6383319519136 |
| YDL010W | 115.159839534628 | 0.482511652743447 | 0.47518257875337 | 1.01542370094734 | 0.309903899950716 | 0.633323413761106 | 212.443047406421 | 115.829190809737 | 73.8211088275003 | 110.015542168503 | 61.2178929420044 | 117.632255053604 |
| YDL011C | 1.64818522845306 | 0.398148773023599 | 1.72898220160646 | 0.230279277978491 | 0.817874761732736 | NA | 0.676570214670132 | 1.23222543414614 | 3.75361570309323 | 3.11364741986329 | 1.11305259894553 | 0 |
| YDL012C | 993.630673499074 | -1.08416697851805 | 0.391134500274204 | -2.77185208095425 | 0.00557383585135365 | 0.0764639820371857 | 1005.38333899982 | 405.402167834081 | 497.979683277036 | 1321.22438849532 | 1305.61069856311 | 1426.18376382508 |
| YDL013W | 236.572954362121 | 0.339076678050866 | 0.303611953796699 | 1.11680938056186 | 0.26407583713316 | 0.591375892268453 | 322.723992397653 | 239.051734224352 | 230.221763123052 | 194.084022504812 | 209.25388860176 | 224.102325321099 |
| YDL014W | 52886.7747451864 | 1.30661971162098 | 0.365253447595733 | 3.57729603983687 | 0.000347166916462785 | 0.013042584547896 | 41870.9008752904 | 89593.8790913319 | 94504.7825567784 | 31799.6810990638 | 30175.9690100124 | 29375.4358386415 |
| YDL015C | 8794.25536013498 | 0.384712571689737 | 0.345823142187328 | 1.11245467627306 | 0.2659427071971 | 0.592894614644195 | 6025.53433185219 | 11472.0187919006 | 12383.1782045046 | 7807.98984654385 | 7982.81323963737 | 7093.99774637133 |
| YDL016C | 1733.18189163303 | -0.0847050465860886 | 0.378778279175018 | -0.22362699036116 | 0.823047560212103 | 0.936610490543666 | 895.102394008584 | 2007.29523222407 | 2145.81697693497 | 1811.10491588715 | 1846.55426165064 | 1693.21756909275 |
| YDL017W | 283.364279751924 | 0.492571295650453 | 0.300607694551576 | 1.63858512133308 | 0.101299693099176 | 0.364831225522595 | 339.638247764406 | 357.345375902381 | 296.535640544365 | 196.159787451387 | 283.828412731111 | 226.678214117893 |
| YDL018C | 465.750378795758 | -0.804827480814635 | 0.27762427610092 | -2.89898092529225 | 0.00374377669398205 | 0.0586877766808882 | 389.704443649996 | 340.094219824335 | 286.52599866945 | 621.69160149937 | 574.335141055896 | 582.150868075498 |
| YDL019C | 266.769046486199 | 0.431207194899829 | 0.447913589620065 | 0.962701746257784 | 0.33569722013396 | 0.650515759457194 | 526.371627013363 | 173.743786214606 | 217.709710779408 | 212.765907023992 | 238.193256174344 | 231.829991711481 |
| YDL020C | 137.516265162726 | 0.074614852793575 | 0.519436937858308 | 0.143645642724639 | 0.885780296704122 | 0.96256191460552 | 263.185813506681 | 87.4880058243761 | 71.3186983587715 | 169.174843145905 | 106.853049498771 | 127.077180641849 |
| YDL021W | 29.2035922784773 | -0.392890745237425 | 0.453287231678223 | -0.866758906450575 | 0.386074122175438 | 0.697020358259351 | 27.0628085868053 | 19.7156069463383 | 28.7777203903815 | 34.2501216184962 | 34.5046305673116 | 30.9106655615309 |
| YDL022W | 6099.95665190343 | 1.06958232625784 | 0.405380762565816 | 2.63846345220732 | 0.0083282676989792 | 0.093864475948495 | 4815.826788022 | 10051.2628663301 | 9922.05750850978 | 3050.33658899274 | 5614.23730908128 | 3146.0188504847 |
| YDL023C | 4.52945543499883 | 1.4498627650222 | 1.16266385451863 | 1.24701800902074 | 0.212390894295926 | NA | 1.35314042934026 | 9.85780347316914 | 8.75843664055088 | 1.03788247328776 | 4.45221039578214 | 1.71725919786283 |
| YDL024C | 68.3924110754887 | 0.40950110607553 | 0.394212085672783 | 1.03878374346807 | 0.298905323549574 | 0.624539367416557 | 73.7461533990444 | 82.5591040877915 | 77.5747245305935 | 47.7425937712371 | 82.3658923219696 | 46.3659983422963 |
| YDL025C | 101.121133626957 | 0.229745310146612 | 0.39707311961869 | 0.578596985782459 | 0.562861139059544 | 0.810832039687759 | 158.994000447481 | 91.1846821268145 | 76.3235192962291 | 89.2578927027477 | 87.9311553166972 | 103.03555187177 |
| YDL025W-A | 5.6761065174961 | 0.445349235428266 | 1.16304508142546 | 0.38291657179998 | 0.701781623627836 | NA | 0 | 9.85780347316914 | 10.0096418749153 | 5.18941236643882 | 5.56526299472767 | 3.43451839572565 |
| YDL026W | 25.8978859036559 | 0.370281232744837 | 0.500048360349624 | 0.740490844697388 | 0.459002214383033 | 0.749329870556722 | 20.9736766547741 | 33.2700867219458 | 33.7825413278391 | 16.6061195726042 | 26.7132623746928 | 24.0416287700796 |
| YDL027C | 387.090366509089 | -0.830737862815502 | 0.278492073008189 | -2.98298566936616 | 0.00285451359545854 | 0.0491835141638811 | 323.400562612323 | 266.160693775567 | 245.236225935425 | 521.017001590457 | 484.177880541308 | 482.549834599454 |
| YDL028C | 210.346805739506 | 0.0827277571571494 | 0.288895238664064 | 0.286359019067627 | 0.774603159037169 | 0.919994563269937 | 186.733379248956 | 231.658381619475 | 231.472968357416 | 207.576494657553 | 192.558099617577 | 212.081510936059 |
| YDL029W | 5508.26807022679 | 0.169814847354713 | 0.303475132649574 | 0.559567585891131 | 0.575774419424162 | 0.817173176012366 | 4178.49764580273 | 6348.42543672093 | 6970.46436064414 | 5548.51970219638 | 4867.37901518882 | 5136.32226080771 |
| YDL030W | 222.543230286946 | 0.671199726346745 | 0.273618647385754 | 2.45304818498161 | 0.0141651362609577 | 0.128424610769692 | 266.568664580032 | 282.179624419467 | 271.511535857077 | 173.326373039057 | 172.523152836558 | 169.150030989488 |
| YDL031W | 122.717062027005 | 0.521807495085114 | 0.456137593344337 | 1.14396950108693 | 0.252636285632219 | 0.576133052094532 | 239.505855993227 | 101.042485599984 | 92.5891873429664 | 111.053424641791 | 77.9136819261874 | 114.197736657878 |
| YDL032W | 3.58139672032485 | 0.428144630446138 | 1.0913929734401 | 0.39229190664167 | 0.694842542941861 | NA | 4.73599150269092 | 2.46445086829228 | 5.00482093745765 | 3.11364741986329 | 4.45221039578214 | 1.71725919786283 |
| YDL033C | 344.756015193311 | -0.381898980293053 | 0.339721676646966 | -1.12415252409671 | 0.260948326183255 | 0.586800250699235 | 391.057584079336 | 205.781647502406 | 300.289256247459 | 404.774164582228 | 345.046305673116 | 421.587133075324 |
| YDL035C | 316.61347087169 | -0.20818114917091 | 0.279305792329529 | -0.745352065328078 | 0.456058913717527 | 0.747493694171321 | 297.014324240188 | 327.771965482874 | 256.497073044704 | 317.592036826056 | 365.081252454135 | 335.724173182182 |
| YDL036C | 131.963362516595 | 0.599923586529992 | 0.504443310169533 | 1.18927850649535 | 0.234330090319546 | 0.560519916419789 | 288.895481664146 | 114.596965375591 | 72.5699035931359 | 108.977659695215 | 96.8355761082615 | 109.904588663221 |
| YDL037C | 681.436048879735 | 1.10425896296683 | 0.414979761626524 | 2.66099474017397 | 0.00779101741836478 | 0.0904702386277994 | 1473.56992755155 | 592.700433824294 | 723.19662546263 | 388.168045009624 | 552.074089076985 | 358.90717235333 |
| YDL039C | 2699.64633961548 | 1.08329638431324 | 0.399540216063183 | 2.71135755741275 | 0.00670083247764592 | 0.0839136929880364 | 5495.77985376548 | 2945.01878760928 | 2562.46831997831 | 1351.32298022067 | 2363.01066756137 | 1480.27742855776 |
| YDL040C | 1839.66866395955 | 0.568636921781646 | 0.357298615568157 | 1.59148929496251 | 0.111499502699876 | 0.384001882276144 | 3280.68897093547 | 1693.0777465168 | 1617.80836803318 | 1455.11122754944 | 1498.16879818069 | 1493.15687254173 |
| YDL041W | 7.2480605149692 | -0.681825325029785 | 0.879292620662405 | -0.775424823326891 | 0.438088671648348 | NA | 3.38285107335066 | 4.92890173658457 | 8.75843664055088 | 9.34094225958987 | 3.3391577968366 | 13.7380735829026 |
| YDL042C | 896.956732911332 | 0.289551720270048 | 0.26059440647573 | 1.11112024308555 | 0.266516595560843 | 0.593084549471051 | 860.597313060408 | 1022.7471103413 | 1077.28770678776 | 846.912098202815 | 790.26734525133 | 783.92882382438 |
| YDL043C | 118.353696580724 | 0.520652724674901 | 0.428992998757148 | 1.21366252172717 | 0.22487662153983 | 0.548365637869472 | 217.855609123782 | 107.203612770714 | 92.5891873429664 | 94.4473050691865 | 81.252839723024 | 116.773625454672 |
| YDL044C | 35.194339649324 | -0.409989171710612 | 0.465222567715195 | -0.881275329621592 | 0.378168815587076 | 0.689560554872609 | 23.0033872987845 | 35.7345375902381 | 32.5313360934747 | 50.8562411911004 | 27.8263149736384 | 41.2142207487078 |
| YDL045C | 622.400938948169 | -0.217316469019111 | 0.257067894630025 | -0.845366043596675 | 0.39790649727669 | 0.704186186941373 | 623.797737925861 | 563.127023404787 | 539.269456011061 | 651.790193224716 | 709.014505528306 | 647.406717594285 |
| YDL045W-A | 320.146848970317 | -1.35224212623428 | 0.30263454586211 | -4.46823452485298 | 7.88678119706448e-06 | 0.000809521755727262 | 221.238460197133 | 150.331502965829 | 167.661501404831 | 504.410882017853 | 430.751355791922 | 446.487391444335 |
| YDL046W | 8386.66960473451 | 0.524467452153867 | 0.378084100849117 | 1.38717140174896 | 0.165389503657666 | 0.467843930622179 | 5231.24089982946 | 12036.3780407395 | 12416.9607458324 | 6559.41723117867 | 7218.14610416179 | 6857.87460666519 |
| YDL047W | 3387.08349896259 | 0.572959625378779 | 0.245401736349703 | 2.33478227946317 | 0.0195547973830268 | 0.153898859492293 | 4176.46793515872 | 3823.59552215548 | 4152.75017285548 | 2501.29676062351 | 2816.0230753322 | 2852.36752765015 |
| YDL048C | 1215.99802375339 | 0.57456794762118 | 0.582126484463291 | 0.987015645149558 | 0.323634968899119 | 0.640362754296777 | 3118.98868962931 | 656.776156399894 | 588.066460151273 | 835.49539099665 | 1186.51407047594 | 910.147374867297 |
| YDL049C | 289.024105400702 | -0.217032877403253 | 0.320393725400688 | -0.677394281463626 | 0.498155825640578 | 0.774325205575924 | 320.694281753642 | 256.302890302398 | 223.96573695123 | 259.470618321941 | 381.777041438318 | 291.93406363668 |
| YDL050C | 5.09084859336119 | -0.521081949272781 | 1.08628694498422 | -0.479690888009662 | 0.631447208070966 | NA | 2.70628085868053 | 8.625578039023 | 1.25120523436441 | 6.22729483972658 | 10.0174733905098 | 1.71725919786283 |
| YDL051W | 781.159099766203 | 1.33105410361045 | 0.725531421912778 | 1.83459194655042 | 0.0665661728420007 | 0.295461283008355 | 2773.93788014754 | 316.681936575559 | 262.753099216526 | 440.062168674012 | 321.67220109526 | 571.847312888321 |
| YDL052C | 2778.88632031375 | 0.186177973621212 | 0.268926027894132 | 0.692301801648238 | 0.488747818909945 | 0.767596230573722 | 2445.12475581786 | 3111.36922121901 | 3318.19628153442 | 2413.07675039405 | 2758.14434018703 | 2627.40657273012 |
| YDL053C | 19.1923225891396 | 0.581425839456525 | 0.973910362692134 | 0.597001389172323 | 0.550506453131221 | 0.803400588784296 | 62.2444597496521 | 3.69667630243843 | 2.50241046872882 | 17.644002045892 | 14.469683786292 | 14.596703181834 |
| YDL054C | 1343.55891923509 | 0.285064294610934 | 0.370461653914056 | 0.769483944152197 | 0.441606072415415 | 0.737703610738415 | 821.35624060954 | 1784.26242864361 | 1823.00602646895 | 1236.11802568573 | 1296.70627777155 | 1099.90451623114 |
| YDL055C | 100659.466168298 | 0.280995285763992 | 0.31156983985868 | 0.901869339764861 | 0.367126280161039 | 0.681769945236982 | 75827.9599495844 | 126899.504110106 | 128567.593857115 | 90563.5488541437 | 96470.4948558074 | 85627.695383034 |
| YDL056W | 8.02980941208145 | 0.0822449935400012 | 0.852984873730504 | 0.0964202251093916 | 0.923186829645815 | NA | 15.561114937413 | 2.46445086829228 | 6.25602617182206 | 7.26517731301435 | 8.90442079156428 | 7.72766639038271 |
| YDL057W | 291.274363638707 | -0.299348546558411 | 0.294789742899682 | -1.01546459389627 | 0.309884415770031 | 0.633323413761106 | 304.456596601559 | 229.193930751182 | 248.989841638518 | 280.228267787696 | 355.063779063626 | 329.713765989662 |
| YDL058W | 251.673264842448 | 0.0611742841307454 | 0.273078403904119 | 0.224017290478321 | 0.822743849257941 | 0.936610490543666 | 242.212136851907 | 261.231792038982 | 267.757920153984 | 242.864498749337 | 261.567360752201 | 234.405880508276 |
| YDL059C | 82.960198758323 | -0.000952772044167276 | 0.561380466313357 | -0.00169719486398276 | 0.99864583507146 | 0.999167321147215 | 158.317430232811 | 36.9667630243843 | 52.5506198433053 | 79.9169504431578 | 60.1048403430589 | 109.904588663221 |
| YDL060W | 1679.97450140298 | 0.51174372198721 | 0.251953824182333 | 2.03110122915568 | 0.0422447268083876 | 0.229294324546376 | 1951.22849910866 | 1882.84046337531 | 2090.76394662293 | 1448.88393270972 | 1242.16670042322 | 1463.96346617806 |
| YDL061C | 28215.7590429042 | -0.448159548055717 | 0.386835096045179 | -1.15852866670447 | 0.246648360107521 | 0.570275827123806 | 12445.5090988571 | 28538.3410548247 | 30621.9969058346 | 33139.5873720783 | 36222.0707274845 | 28327.0490983462 |
| YDL062W | 3.19028665404898 | -1.07037460293636 | 1.15586796659671 | -0.926035355134837 | 0.354427606960956 | NA | 1.35314042934026 | 2.46445086829228 | 2.50241046872882 | 5.18941236643882 | 3.3391577968366 | 4.29314799465706 |
| YDL063C | 1303.35665916008 | 0.23060711077087 | 0.385961798433669 | 0.597486880066194 | 0.550182363896319 | 0.803335259270233 | 2148.11043157767 | 1182.9364167803 | 889.606921633096 | 1382.4594544193 | 1040.70418001407 | 1176.32255053604 |
| YDL064W | 2305.7569100774 | -0.250685803605761 | 0.278107721582269 | -0.901398214258514 | 0.367376630536382 | 0.681769945236982 | 1658.27359615649 | 2274.68815143378 | 2386.04838193293 | 2378.82662877555 | 2552.22960938211 | 2584.47509278355 |
| YDL065C | 19.1498031033783 | -0.140583371200825 | 0.674150163455119 | -0.20853420917428 | 0.834811874646116 | 0.941803181712136 | 20.9736766547741 | 25.876734117069 | 7.50723140618647 | 32.1743566719207 | 18.9218941820741 | 9.44492558824554 |
| YDL066W | 4284.58097060431 | -0.240189795599465 | 0.257640871103121 | -0.93226588844799 | 0.351199131861056 | 0.663866153224391 | 3835.47654696498 | 3445.30231387261 | 4505.59004894625 | 4513.75087632848 | 4782.78701766896 | 4624.57901984459 |
| YDL067C | 2242.29928284508 | -0.828293638287066 | 0.36946608112592 | -2.24186652199007 | 0.0249700003082568 | 0.175676330198115 | 886.306981217873 | 1951.84508768749 | 2010.68681162361 | 2874.93445100711 | 2912.85865144046 | 2817.16371409396 |
| YDL068W | 12.713738226968 | -0.783071968356852 | 0.638937542648384 | -1.22558453070551 | 0.220355062081059 | 0.541745464859678 | 6.08913193203119 | 9.85780347316914 | 12.5120523436441 | 17.644002045892 | 15.5827363852375 | 14.596703181834 |
| YDL069C | 59.8357796517283 | -0.413668497705631 | 0.365617146288388 | -1.13142532264978 | 0.257876118305175 | 0.583571625203994 | 60.2147491056417 | 48.0567919316995 | 45.0433884371188 | 70.5760081835679 | 69.0092611346231 | 66.1144791177188 |
| YDL070W | 241.791663401534 | 0.752517781924491 | 0.635246738087905 | 1.18460707754214 | 0.236172837394762 | 0.562356206612219 | 686.042197675514 | 112.132514507299 | 111.357265858433 | 192.008257558236 | 149.149048258702 | 200.060696551019 |
| YDL071C | 33.8479585049066 | -0.321584723473567 | 0.569669385438118 | -0.564511156284525 | 0.572406309797795 | 0.815365951041003 | 11.5016936493922 | 35.7345375902381 | 43.7921832027544 | 32.1743566719207 | 48.9743143536035 | 30.9106655615309 |
| YDL072C | 7703.84590128895 | 0.15534705853101 | 0.390545752558905 | 0.397769166642208 | 0.690800347356879 | 0.880621068220746 | 4443.71316995343 | 10305.1013057642 | 9606.75378944995 | 5987.54398839711 | 9193.81446729012 | 6686.14868687891 |
| YDL073W | 276.611289293434 | 0.233388548169058 | 0.304285943042311 | 0.767004041775947 | 0.443079112457238 | 0.738636525059224 | 235.446434705206 | 300.663005931659 | 361.598312731315 | 262.584265741804 | 242.645466570127 | 256.730250080492 |
| YDL074C | 258.616249668466 | -0.683398584757505 | 0.322507799549784 | -2.11901413147688 | 0.0340892727010426 | 0.208009702214005 | 150.875157871439 | 223.032803580452 | 222.714531716865 | 369.486160490444 | 270.471781543765 | 315.117062807828 |
| YDL075W | 15153.7638068293 | -0.375123869073421 | 0.278410408198817 | -1.34737731789661 | 0.177858741938867 | 0.487986658073799 | 15888.5749213134 | 11517.611132964 | 12176.7293408345 | 19082.5071538688 | 15277.7599731264 | 16979.4003188687 |
| YDL076C | 397.21666389872 | -0.249801708061655 | 0.264233443607061 | -0.945382630796473 | 0.344463566447245 | 0.656807762433896 | 356.552503131159 | 391.847688058473 | 340.32782374712 | 419.304519208257 | 459.690723364506 | 415.576725882804 |
| YDL077C | 246.148884690361 | 0.202857271237767 | 0.310713523189945 | 0.652875578620236 | 0.513836494180196 | 0.783014890919344 | 324.077132826993 | 258.76734117069 | 206.448863670128 | 226.258379176732 | 241.532413971181 | 219.809177326442 |
| YDL078C | 6840.36529899903 | -0.0876722519919777 | 0.274582624487706 | -0.31929278903044 | 0.749504500267783 | 0.908603445448746 | 5378.73320662755 | 6589.94162181357 | 7930.13877540164 | 7083.54788018899 | 6933.20463883174 | 7126.62567113072 |
| YDL079C | 59.4381153932044 | -0.273674494318461 | 0.370343818896234 | -0.73897411096023 | 0.459922718045253 | 0.749329870556722 | 43.9770639535586 | 57.9145954048687 | 60.0578512494917 | 66.4244782904169 | 69.0092611346231 | 59.2454423262675 |
| YDL080C | 637.480397175794 | -0.22863737901114 | 0.524994954721121 | -0.43550395476199 | 0.663196669039204 | 0.866909384383846 | 1167.08362030598 | 271.089595512151 | 321.559745231654 | 670.472077743895 | 662.266296372593 | 732.411047888495 |
| YDL081C | 93891.1205735767 | -0.183016544212381 | 0.36617348271692 | -0.499808295386223 | 0.617210069015653 | 0.841490388212044 | 48928.2047845146 | 103596.888924969 | 111307.217649058 | 98278.1292780916 | 106579.238559431 | 94657.0442453968 |
| YDL082W | 13575.4737188971 | -0.0992115076240395 | 0.346232478681385 | -0.286545930069539 | 0.774460020391041 | 0.919994563269937 | 19127.993109154 | 10063.5851206715 | 10133.5111931174 | 15054.485275039 | 13105.0812999847 | 13968.1863154162 |
| YDL083C | 83075.892348008 | -0.00867076376694566 | 0.352567848365558 | -0.0245931777589527 | 0.98037946101508 | 0.99431092046569 | 48497.2295577697 | 96170.2662333698 | 103812.498295215 | 79506.9868662091 | 84252.5164771822 | 86215.8566583021 |
| YDL084W | 16540.3074881728 | 0.533490034077077 | 0.277594305834385 | 1.92183349176968 | 0.0546267126287751 | 0.265646653291201 | 15925.1097129056 | 20525.1790565723 | 22242.6754512961 | 15115.720340963 | 13299.8655048002 | 12133.2948624998 |
| YDL085C-A | 119.199133943517 | -0.957637425664843 | 0.356364689651162 | -2.68723993559029 | 0.00720451803351488 | 0.0864679464259746 | 86.6009874777769 | 92.4169075609607 | 63.811466952585 | 195.1219049781 | 123.548838482954 | 153.694698208723 |
| YDL085W | 93.7719122619611 | -0.35737483205275 | 0.381219841183561 | -0.937450765792304 | 0.348526738005581 | 0.661165574275933 | 60.8913193203119 | 80.0946532194992 | 106.352444920975 | 87.1821277561722 | 130.227154076628 | 97.883774278181 |
| YDL086C-A | 105.235528156796 | 0.326757614479489 | 0.43593601104008 | 0.749554077213976 | 0.453523317745521 | 0.745667056750931 | 58.1850384616313 | 156.49263013656 | 137.632575780085 | 98.5988349623375 | 83.4789449209151 | 97.0251446792496 |
| YDL086W | 8634.35015907851 | 0.252803973359771 | 0.334371817373341 | 0.756056462370762 | 0.449615343852708 | 0.742427175083414 | 5951.11160823848 | 10855.9060748275 | 11360.9435280289 | 7208.09377698352 | 8212.10207502016 | 8217.94389137255 |
| YDL087C | 73.0213593538407 | 0.874743337712554 | 0.561303335944151 | 1.55841464266585 | 0.119134987758771 | 0.396897218069931 | 183.350528175606 | 50.5212427999918 | 48.797004140212 | 41.5152989315106 | 58.9917877441133 | 54.9522943316104 |
| YDL088C | 546.962443435499 | -0.242229669330695 | 0.267982447242241 | -0.903901251083555 | 0.366047765723322 | 0.681581651239926 | 576.437822898952 | 469.47789040968 | 456.68991054301 | 604.047599453478 | 573.22208845695 | 601.89934885092 |
| YDL089W | 160.319734215021 | 0.195986876856442 | 0.341295130013694 | 0.574244574918424 | 0.565802309395177 | 0.812511326930328 | 228.004162343834 | 134.31257232193 | 150.144628123729 | 150.492958626726 | 154.714311253429 | 144.249772620477 |
| YDL090C | 1017.93981515117 | 0.163117257518568 | 0.254728271841554 | 0.640357885441274 | 0.521939955931166 | 0.78888005960882 | 1000.64734749712 | 1058.48164793154 | 1167.374483662 | 939.283638325426 | 1035.13891701935 | 906.712856471572 |
| YDL091C | 43.4070705248201 | 0.672276443662474 | 0.647439781085774 | 1.03836134773036 | 0.299101857427539 | 0.624722920237461 | 108.251234347221 | 14.7867052097537 | 36.2849517965679 | 32.1743566719207 | 31.165472770475 | 37.7797023529822 |
| YDL092W | 300.713265130351 | -0.312594668058631 | 0.398411155392596 | -0.784603201560957 | 0.432686252362512 | 0.731159519481642 | 402.559277728728 | 198.388294897529 | 202.695247967035 | 344.576981131538 | 248.210729564854 | 407.849059492421 |
| YDL093W | 1180.38943469011 | 0.502573395464245 | 0.261097252028513 | 1.92485134010281 | 0.0542479589646067 | 0.264925461451622 | 1223.91551833827 | 1435.54263078026 | 1492.68784459674 | 1046.18553307407 | 996.182076056254 | 887.823005295081 |
| YDL094C | 15.6393058391629 | -0.611378469510065 | 0.63819343548394 | -0.957983011916221 | 0.338071324953527 | 0.65233191195463 | 8.79541279071171 | 17.251156078046 | 11.2608471092797 | 15.5682370993165 | 28.9393675725839 | 12.0208143850398 |
| YDL095W | 8705.04309614937 | 0.524280366698068 | 0.237020117985391 | 2.21196568103296 | 0.0269690363157975 | 0.18325643916426 | 10473.9834933083 | 9898.46691249596 | 10436.3028598336 | 7215.35895429653 | 6870.87369329079 | 7335.27266367106 |
| YDL097C | 987.620171003423 | 0.609757735869916 | 0.615292067580583 | 0.991005358264362 | 0.321682963851458 | 0.638699024600408 | 2681.24776073773 | 455.923410634073 | 441.675447730637 | 799.169504431578 | 715.692821121979 | 832.012081364539 |
| YDL098C | 18.8375966129254 | 0.41216374448174 | 0.798236446629282 | 0.51634292849216 | 0.605614918634846 | 0.83299223553795 | 46.6833448122391 | 7.39335260487685 | 10.0096418749153 | 14.5303546260287 | 7.79136819261874 | 26.6175175668738 |
| YDL099W | 129.220024638432 | -1.30583119613773 | 0.304581422439973 | -4.28729758261959 | 1.80859934645895e-05 | 0.00152879838874207 | 69.6867321110236 | 76.3979769170608 | 77.5747245305935 | 179.553667878783 | 189.218941820741 | 182.888104572391 |
| YDL100C | 2973.24221862035 | -0.128188160268443 | 0.636492695510172 | -0.201397692656465 | 0.840387619243806 | 0.942840676530536 | 6476.1300948225 | 1176.77528960957 | 869.587637883266 | 3319.14814957427 | 2345.20182597824 | 3652.61031385423 |
| YDL101C | 119.681442562168 | -0.078037052792935 | 0.386238812696634 | -0.202043529100811 | 0.839882694539459 | 0.942531379971263 | 170.495694096873 | 91.1846821268145 | 86.3331611711444 | 122.470131847956 | 129.114101477682 | 118.490884652535 |
| YDL102W | 818.951115991129 | 0.662692670968984 | 0.296600389393452 | 2.23429467616071 | 0.0254636904079662 | 0.176635591820159 | 764.524342577249 | 1112.69956703397 | 1134.84314756852 | 692.267609682938 | 636.666086596846 | 572.705942487252 |
| YDL103C | 497.554436969998 | 0.794915058001386 | 0.648883841301685 | 1.22504985854904 | 0.220556435563811 | 0.542008718093537 | 1463.4213743315 | 219.336127278013 | 210.202479373221 | 363.258865650717 | 316.106938100532 | 413.000837086009 |
| YDL104C | 409.184434721393 | 0.158602385731566 | 0.336836817459177 | 0.470858224252128 | 0.637741982412105 | 0.854485061749365 | 284.836060376125 | 475.639017580411 | 535.515840307968 | 400.622634689077 | 399.585883021447 | 358.90717235333 |
| YDL105W | 27.8807922878942 | -0.175129484268565 | 0.517854424507561 | -0.338182848268795 | 0.735225400308447 | 0.902003168869667 | 39.2410724508676 | 18.4833815121921 | 20.0192837498306 | 23.8712968856186 | 35.6176831662571 | 30.0520359625994 |
| YDL106C | 123.163785846462 | 0.0612998952261548 | 0.355288651169185 | 0.172535472282689 | 0.863016576634122 | 0.953454774053698 | 107.574664132551 | 171.279335346314 | 98.8452135147885 | 126.621661741107 | 121.322733285063 | 113.339107058946 |
| YDL107W | 33.3729864090435 | 0.662295177690051 | 0.433495462105473 | 1.52780187011256 | 0.126561737819184 | 0.407215191710193 | 43.3004937388884 | 41.8956647609688 | 37.5361570309323 | 28.0228267787696 | 21.1479993799652 | 28.3347767647366 |
| YDL108W | 897.031115982735 | 0.0105651110622835 | 0.245522245547286 | 0.0430311764163495 | 0.965676681676898 | 0.989039165251922 | 864.656734348428 | 910.614595833999 | 925.891873429664 | 912.298694019944 | 893.781236953264 | 874.943561311109 |
| YDL109C | 57.0846620161223 | 1.20426652085123 | 0.748414265707261 | 1.60909081511586 | 0.107596485335156 | 0.376195010770364 | 193.499081395658 | 20.9478323804844 | 23.7728994529238 | 33.2122391452084 | 36.7307357652026 | 34.3451839572565 |
| YDL110C | 169.549960095124 | -0.389896721883836 | 0.445869776613651 | -0.874463222973024 | 0.381866040036843 | 0.692418295940622 | 241.535566637237 | 105.971387336568 | 91.337982108602 | 149.455076153438 | 231.514940580671 | 197.484807754225 |
| YDL111C | 3297.27066653953 | 0.196858640086261 | 0.286504410419289 | 0.687105094815698 | 0.492016503891335 | 0.771022591157959 | 2692.74945438712 | 3723.78526198964 | 4150.24776238675 | 3140.63236416877 | 2917.31086183625 | 3158.89829446867 |
| YDL112W | 2433.62767039608 | 0.497887068331465 | 0.263127825291238 | 1.89218706832084 | 0.0584660607331601 | 0.274561206776311 | 3236.03533676724 | 2830.42182223369 | 2481.13997974463 | 2193.04566605704 | 1948.95510075363 | 1912.16811682026 |
| YDL113C | 32.1181747930919 | 0.627636471413395 | 0.568154266126481 | 1.10469375807463 | 0.269292309007308 | 0.595490777175407 | 67.6570214670132 | 20.9478323804844 | 27.5265151560171 | 22.8334144123308 | 32.2785253694205 | 21.4657399732853 |
| YDL114W | 4.91355656931303 | -0.528167115940734 | 0.963164192275595 | -0.548366644209306 | 0.583440175099637 | NA | 3.38285107335066 | 2.46445086829228 | 6.25602617182206 | 7.26517731301435 | 6.67831559367321 | 3.43451839572565 |
| YDL114W-A | 8.25877237519705 | 0.28380606547772 | 0.767460176685288 | 0.369799077658331 | 0.711532202524141 | NA | 10.8251234347221 | 9.85780347316914 | 6.25602617182206 | 8.30305978630211 | 10.0174733905098 | 4.29314799465706 |
| YDL115C | 196.181522733862 | 0.211350671075859 | 0.292623140761338 | 0.722262328693392 | 0.470133209098462 | 0.758293350262115 | 213.119617621092 | 223.032803580452 | 195.188016560848 | 165.023313252754 | 208.140836002815 | 172.584549385214 |
| YDL116W | 1212.61889293466 | 0.112316768227366 | 0.257328755368074 | 0.436471890079724 | 0.662494389768795 | 0.866909384383846 | 1125.13626699643 | 1401.04031862416 | 1253.70764483314 | 1218.47402363983 | 1173.15743928859 | 1104.1976642258 |
| YDL117W | 692.98668832586 | -0.316454235025668 | 0.31814787195809 | -0.994676573123064 | 0.319893591924971 | 0.638537064188392 | 830.151653400252 | 533.55361298528 | 486.718836167756 | 785.677032278837 | 730.162504908271 | 791.656490214762 |
| YDL118W | 1.6454896800675 | -0.080502795136114 | 1.67152438131761 | -0.0481613047562348 | 0.961587688712122 | NA | 0 | 2.46445086829228 | 2.50241046872882 | 2.07576494657553 | 1.11305259894553 | 1.71725919786283 |
| YDL119C | 348.561320411458 | -0.49232107801465 | 0.336944975875847 | -1.46113197484225 | 0.143979226804124 | 0.437185734638195 | 394.440435152687 | 241.516185092644 | 231.472968357416 | 365.334630597293 | 461.916828562397 | 396.686874706313 |
| YDL120W | 1572.58655555811 | -0.358312655183295 | 0.253912223991731 | -1.41116740876155 | 0.158195257566485 | 0.457627750625141 | 1255.71431842776 | 1332.03569431198 | 1547.74087490878 | 1716.65761081796 | 1823.18015707279 | 1760.1906778094 |
| YDL121C | 920.304864713694 | -0.00725387469907979 | 0.306493103374817 | -0.0236673341723088 | 0.981117962257629 | 0.994439436969997 | 667.09823166475 | 996.870376224229 | 1091.05096436577 | 996.367174356253 | 923.833657124794 | 846.608784546373 |
| YDL122W | 87.963767762832 | 0.600893788520501 | 0.460542114083603 | 1.30475318140269 | 0.191976906107132 | 0.506395553012046 | 161.023711091491 | 72.7013006146224 | 83.8307507024156 | 80.9548329164456 | 43.4090513588758 | 85.8629598931413 |
| YDL123W | 937.387204755317 | 0.028345817497434 | 0.34679809845259 | 0.0817357927390974 | 0.934856815082243 | 0.98165448197831 | 594.705218695046 | 1116.3962433364 | 1129.83832663106 | 807.47256421788 | 1045.15639040986 | 930.754485241651 |
| YDL124W | 4884.59626523534 | -0.26231322188793 | 0.328899375663236 | -0.797548555265473 | 0.425132515056269 | 0.724641459965427 | 5821.88669723648 | 3510.61026188236 | 3991.34469762247 | 4458.74310524423 | 6628.22822672066 | 4896.76460270585 |
| YDL125C | 11782.072858242 | 0.096473075810747 | 0.373656912387607 | 0.258186246828139 | 0.796263171632922 | 0.929585587537483 | 6715.63595081573 | 15068.8848341732 | 14744.2024817502 | 10003.1112775475 | 12901.3926743777 | 11259.2099307876 |
| YDL126C | 16496.0036151819 | 0.173391904371329 | 0.255708572878772 | 0.678084048646785 | 0.497718404003872 | 0.77425856189831 | 16429.1545228348 | 16190.2099792462 | 19839.1101960821 | 15264.1375346431 | 16761.4590875208 | 14491.9503707644 |
| YDL127W | 108.25889862452 | -0.326151242015644 | 0.529179406267465 | -0.616333965669852 | 0.537674125988865 | 0.79796293666817 | 181.320817531595 | 60.379046273161 | 45.0433884371188 | 138.038368947273 | 126.887996279791 | 97.883774278181 |
| YDL128W | 5877.3113833992 | 1.02052804598879 | 0.375190139849356 | 2.72002896024544 | 0.00652761991222731 | 0.082282366788339 | 4231.270122547 | 9745.67095866184 | 9644.28994648088 | 3806.95291201952 | 4165.04282525419 | 3670.64153543179 |
| YDL129W | 110.517651201682 | 0.882108507951994 | 0.3905482735017 | 2.25864142233407 | 0.02390569896903 | 0.170277250492349 | 200.941353757029 | 134.31257232193 | 93.8403925773309 | 72.6517731301435 | 82.3658923219696 | 78.99392310169 |
| YDL130W | 89126.3997817917 | 0.129968862839355 | 0.396774676277307 | 0.327563402127307 | 0.743241797335742 | 0.904925619802128 | 45850.4868779802 | 113942.65367006 | 119622.727636644 | 79119.8567036728 | 93240.4162136674 | 82982.2575887264 |
| YDL130W-A | 142.485482504574 | -1.26029028511113 | 0.34997427131647 | -3.60109410434773 | 0.000316880844947958 | 0.0123678982891459 | 108.927804561891 | 66.5401734438917 | 75.0723140618647 | 217.95531939043 | 169.183995039721 | 217.233288529647 |
| YDL131W | 2317.12134311405 | -0.167220020287455 | 0.5669944724171 | -0.294923545858561 | 0.76805228453323 | 0.916686987437086 | 4583.76320439014 | 847.771098692546 | 1116.07506905305 | 2576.02429870023 | 2005.72078329985 | 2773.37360454846 |
| YDL132W | 330.133821663413 | -0.25721348018199 | 0.446213654432723 | -0.576435699864425 | 0.564320719947555 | 0.811537527710419 | 518.252784437321 | 179.904913385337 | 202.695247967035 | 390.243809956199 | 312.767780303695 | 376.93839393089 |
| YDL133C-A | 1731.58394540695 | -0.216781176566875 | 0.343037080421185 | -0.631946774677269 | 0.527421657405744 | 0.791959165822418 | 1127.84254785511 | 1817.53251536556 | 1860.54218349988 | 1886.87033643715 | 2304.01887981726 | 1392.69720946675 |
| YDL133W | 441.015731704008 | -0.087438325510632 | 0.278698850059351 | -0.313737661608619 | 0.753720294094582 | 0.910356009761642 | 490.513405635846 | 432.511127385296 | 359.095902262586 | 441.1000511473 | 476.386512348689 | 446.487391444335 |
| YDL134C | 2583.90888094191 | 0.275982144517201 | 0.245198330276751 | 1.12554659000208 | 0.260357486170989 | 0.58657467942951 | 2815.88523345709 | 2721.98598402883 | 2952.84435310001 | 2386.09180608857 | 2473.20287485698 | 2153.44303411998 |
| YDL135C | 613.540034707597 | -0.454103921696833 | 0.405608986222754 | -1.11956080146471 | 0.262900967433406 | 0.589581187359512 | 837.593925761623 | 347.487572429212 | 366.603133668773 | 741.048085927463 | 627.761665805281 | 760.745824653232 |
| YDL136W | 1126.80325661084 | -0.502166948703665 | 0.46277635008783 | -1.08511800269906 | 0.277869394830576 | 0.60329343886538 | 1648.12504293644 | 591.468208390148 | 556.786329292163 | 1584.84653671042 | 1000.63428645204 | 1378.95913588385 |
| YDL137W | 11477.1451076817 | -0.191325487293952 | 0.345983402137635 | -0.55299036344478 | 0.580270008853894 | 0.819257784137336 | 6454.47984795306 | 12179.3161911005 | 13519.2725573075 | 12670.469233897 | 11946.3935444824 | 12092.93927135 |
| YDL138W | 308.103066537516 | 0.506847871902838 | 0.399077800384622 | 1.27004777367809 | 0.204067613314929 | 0.522231984434412 | 572.378401610932 | 261.231792038982 | 250.241046872882 | 241.826616276049 | 259.34125555431 | 263.599286871944 |
| YDL139C | 3.88149245166658 | -1.26473620414465 | 1.14718397592895 | -1.10247024948245 | 0.270257285505573 | NA | 4.05942128802079 | 2.46445086829228 | 0 | 5.18941236643882 | 5.56526299472767 | 6.01040719251989 |
| YDL140C | 4997.09310274451 | 0.106779032507027 | 0.31878572425818 | 0.334955502651517 | 0.7376586528739 | 0.90301928216742 | 7131.05006262319 | 4210.51430847737 | 4202.79838223006 | 4743.12290292508 | 4970.89290689076 | 4724.18005332063 |
| YDL141W | 1837.43867972137 | 0.535217366706511 | 0.28510049288798 | 1.87729372645037 | 0.0604778475910051 | 0.279667472206836 | 1725.25404740884 | 2344.92500118011 | 2453.61346458861 | 1593.14959649672 | 1590.55216389317 | 1317.13780476079 |
| YDL142C | 955.828833256371 | -0.0219169102360216 | 0.348184017110496 | -0.0629463420460978 | 0.949809232369977 | 0.985770615313908 | 581.173814401643 | 1065.87500053641 | 1199.90581975547 | 900.881986813779 | 1022.89533843095 | 964.241039599976 |
| YDL143W | 9846.34783086421 | 0.562384248472846 | 0.248452257328015 | 2.26355056911545 | 0.02360177102104 | 0.168945180359823 | 10885.3381838278 | 11388.2274623786 | 12951.225380906 | 8108.9757637973 | 8311.16375632631 | 7433.15643794924 |
| YDL144C | 2514.87805235804 | 0.598077453886689 | 0.308626109158359 | 1.93787056940089 | 0.0526390153857803 | 0.26083539692885 | 2119.01791234685 | 3377.52991499458 | 3590.95902262586 | 1904.51433848305 | 2009.05994109669 | 2088.1871846012 |
| YDL145C | 4990.025288782 | -0.110497864212216 | 0.539793152537685 | -0.204704086542672 | 0.837803327421487 | 0.941803181712136 | 9806.20869142889 | 2436.10968330692 | 2153.32420834115 | 5446.80721981418 | 4602.47249663979 | 5495.22943316104 |
| YDL146W | 718.104429187449 | 0.731059219526295 | 0.398327420415125 | 1.83532235557474 | 0.0664579405779069 | 0.295461283008355 | 517.576214222651 | 1089.28728378519 | 1082.29252772522 | 506.486646964429 | 714.579768523033 | 398.404133904175 |
| YDL147W | 227.716015605981 | 0.785278045220017 | 0.633615322469505 | 1.23936088249793 | 0.21521188113544 | 0.534881368592071 | 651.537116727337 | 114.596965375591 | 97.5940082804241 | 170.212725619193 | 158.053469050266 | 174.301808583077 |
| YDL148C | 26.9495434485054 | 0.358707623354575 | 0.632313379301475 | 0.567294058763779 | 0.570514412876607 | 0.8150306600566 | 55.4787576029508 | 16.0189306438998 | 18.7680785154662 | 28.0228267787696 | 13.3566311873464 | 30.0520359625994 |
| YDL149W | 101.654400376945 | 0.139065747372993 | 0.390596108627418 | 0.356034646278682 | 0.721814618990251 | 0.896293267442601 | 147.492306798089 | 96.1135838633991 | 75.0723140618647 | 78.87906796987 | 110.192207295608 | 102.176922272838 |
| YDL150W | 154.297951177592 | -0.552317224665362 | 0.35119838230445 | -1.57266448962901 | 0.115796526420665 | 0.392220644588086 | 167.789413238193 | 89.9524566926684 | 116.36208679589 | 188.894610138373 | 174.749258034449 | 188.039882165979 |
| YDL153C | 131.845867872618 | 0.682683609203666 | 0.349992041016917 | 1.95056895356848 | 0.0511083434096849 | 0.255452832972929 | 214.472758050432 | 131.848121453637 | 140.134986248814 | 108.977659695215 | 104.62694430088 | 91.0147374867297 |
| YDL154W | 56.38884468938 | 0.696296473783383 | 0.417583635198952 | 1.66744195675111 | 0.0954265568948084 | 0.355023850505734 | 96.7495406978288 | 54.2179191024303 | 57.5554407807629 | 48.7804762445249 | 38.9568409630937 | 42.0728503476392 |
| YDL155W | 309.893168848511 | -0.048234434797208 | 0.507150407918958 | -0.0951087370611279 | 0.924228458366466 | 0.978676334770653 | 582.526954830983 | 165.118208175583 | 165.159090936102 | 320.705684245919 | 273.810939340602 | 352.038135561879 |
| YDL156W | 8.32828960993311 | 2.38913759320181 | 1.26838208967984 | 1.88361031950938 | 0.0596177049586923 | NA | 39.2410724508676 | 1.23222543414614 | 1.25120523436441 | 2.07576494657553 | 4.45221039578214 | 1.71725919786283 |
| YDL157C | 2339.24743581486 | -0.236192350551774 | 0.361212914264905 | -0.653886783180063 | 0.513184749642455 | 0.783014890919344 | 1230.68122048497 | 2505.11430761911 | 2710.11053763332 | 2708.87325528106 | 2506.59445282534 | 2374.11084104536 |
| YDL158C | 610.670747200492 | -0.336385763863766 | 0.411403136147535 | -0.81765483611465 | 0.413554306182421 | 0.714920346447085 | 261.832673077341 | 644.453902058433 | 714.438188822079 | 787.752797225413 | 613.29198201899 | 642.254940000697 |
| YDL159W | 172.97190500631 | -0.230226753040709 | 0.518536089440316 | -0.4439936924915 | 0.65704714418618 | 0.864280886779837 | 303.780026386889 | 96.1135838633991 | 76.3235192962291 | 202.387082291114 | 159.166521649211 | 200.060696551019 |
| YDL160C | 807.416659885585 | 0.484656448550007 | 0.308807301850759 | 1.56944620689129 | 0.11654400452452 | 0.393361678218991 | 718.51756797968 | 1113.93179246811 | 993.456956085343 | 601.971834506903 | 795.832608246057 | 620.789200027411 |
| YDL160C-A | 553.91522149048 | -0.418078431769402 | 0.392485429001184 | -1.06520752332984 | 0.286782029845885 | 0.610753467985257 | 244.241847495918 | 577.913728614541 | 601.829717729282 | 638.297721071975 | 675.62292755994 | 585.585386471223 |
| YDL161W | 83.9904668720821 | -0.962795401453872 | 0.365811026840002 | -2.63194745596059 | 0.00848969916029266 | 0.095310138229223 | 66.980451252343 | 44.3601156292611 | 58.8066460151273 | 93.4094225958987 | 131.340206675573 | 109.045959064289 |
| YDL162C | 2.09700424149841 | 0.280201045427715 | 1.38407331032969 | 0.202446679187081 | 0.839567539087649 | NA | 2.0297106440104 | 2.46445086829228 | 2.50241046872882 | 1.03788247328776 | 1.11305259894553 | 3.43451839572565 |
| YDL164C | 740.64020594286 | 0.119671903381945 | 0.319038265286435 | 0.375102037601987 | 0.707584581702398 | 0.889782580534978 | 1032.44614758662 | 617.344942507217 | 663.138774213138 | 673.585725163759 | 673.396822362049 | 783.92882382438 |
| YDL165W | 1214.25867504049 | -0.54686738832367 | 0.25287754767292 | -2.16257786962964 | 0.0305736619019959 | 0.195916843492389 | 1021.6210241519 | 929.097977346191 | 1009.72262413208 | 1344.05780290765 | 1396.88101167665 | 1584.17161002846 |
| YDL166C | 913.057706439427 | 0.13640866659368 | 0.257482727254073 | 0.529777931313731 | 0.596265907940486 | 0.828750365770883 | 977.64396019834 | 869.951156507177 | 1020.98347124136 | 922.677518752822 | 795.832608246057 | 891.257523690806 |
| YDL167C | 459.372359424901 | 0.0956919050172118 | 0.351194811282556 | 0.27247528136235 | 0.785256595118652 | 0.925308509377206 | 650.860546512667 | 327.771965482874 | 444.177858199366 | 485.728997498673 | 372.872620646754 | 474.822168209071 |
| YDL168W | 2948.85048279108 | 0.833615715936055 | 0.316921465470796 | 2.63035422576282 | 0.00852959414842964 | 0.0955713589964397 | 2549.99313909173 | 4236.39104259444 | 4548.13102691464 | 2174.36378153786 | 2070.27783403869 | 2113.94607256914 |
| YDL169C | 24.4184521152317 | -0.650098408050061 | 0.550571395853895 | -1.18077040134242 | 0.237693945344315 | 0.564156198917009 | 23.0033872987845 | 18.4833815121921 | 15.0144628123729 | 21.795531939043 | 46.7482091557125 | 21.4657399732853 |
| YDL170W | 622.135777477413 | -0.0717371195499634 | 0.279318189578183 | -0.256829387510704 | 0.79731048285764 | 0.929585587537483 | 499.308818426557 | 634.596098585263 | 686.911673666062 | 678.775137530197 | 643.344402190519 | 589.87853446588 |
| YDL171C | 8394.56852059325 | 0.900676396787067 | 0.257301666274645 | 3.50046857382447 | 0.000464440995824659 | 0.0155209700232566 | 9965.20269187637 | 10597.1387336568 | 12236.7871920839 | 5801.7630256786 | 6418.9743381189 | 5347.54514214484 |
| YDL172C | 111.411944986644 | 0.0231556988748382 | 0.422158947435895 | 0.0548506646974584 | 0.956257436533097 | 0.986781021449109 | 59.5381788909716 | 126.919219717053 | 151.395833358094 | 113.129189588366 | 121.322733285063 | 96.1665150803182 |
| YDL173W | 15.3816660930171 | 0.48992551127227 | 0.699511266422143 | 0.700382588229263 | 0.48368840708427 | 0.764853084984975 | 25.0330979427949 | 22.1800578146306 | 6.25602617182206 | 9.34094225958987 | 20.0349467810196 | 9.44492558824554 |
| YDL174C | 1449.92198929962 | 0.0549927804542003 | 0.477840675587225 | 0.115086017712115 | 0.908376942510506 | 0.973299154529197 | 2722.51854383261 | 752.889740263293 | 955.92079905441 | 1405.29286883163 | 1274.44522579264 | 1588.46475802311 |
| YDL175C | 122.669501824447 | 0.408072621001524 | 0.616420207446718 | 0.662003964295406 | 0.507968681056697 | 0.780070525972187 | 304.456596601559 | 54.2179191024303 | 60.0578512494917 | 99.6367174356253 | 95.722523509316 | 121.925403048261 |
| YDL176W | 271.048577240173 | 0.0791563648902316 | 0.271167110547128 | 0.291909902828996 | 0.770355511733932 | 0.917720928797231 | 284.159490161455 | 288.340751590197 | 262.753099216526 | 250.129676062351 | 281.60230753322 | 259.306138877287 |
| YDL177C | 49.7704398230514 | -0.153845055217952 | 0.50558945433513 | -0.304288497117219 | 0.760908107897494 | 0.912866087924729 | 75.7758640430548 | 25.876734117069 | 38.7873622652968 | 65.3865958171291 | 37.8437883641482 | 54.9522943316104 |
| YDL178W | 3245.36061779604 | 0.04831631485486 | 0.316549428225835 | 0.152634345687049 | 0.87868663840043 | 0.959113330331498 | 2253.65538506621 | 3619.04610008722 | 4027.62964941904 | 3412.55757217017 | 3025.27696393396 | 3133.99803609966 |
| YDL179W | 410.755999181613 | -0.251403163463235 | 0.363915466375254 | -0.69082846620209 | 0.489673344288763 | 0.768398138949443 | 557.493856888189 | 287.108526156051 | 279.018767263264 | 444.213698567163 | 440.768829182432 | 455.93231703258 |
| YDL180W | 1016.47035297383 | -0.0345656755215912 | 0.277236733004983 | -0.124679277334328 | 0.900777463661383 | 0.968150497592676 | 871.42243649513 | 1070.803902273 | 1071.03168061594 | 998.442939302829 | 1189.85322827278 | 897.267930883326 |
| YDL181W | 3104.96163572996 | -1.2548773118529 | 0.337477966227687 | -3.7183977546145 | 0.00020049041067794 | 0.00907416441399053 | 1143.40366279252 | 2081.22875827283 | 2278.44473177759 | 4450.44004545793 | 4296.38303192976 | 4379.86958414914 |
| YDL182W | 1724.58602225561 | 0.318059381970307 | 0.550278244062141 | 0.577997377512876 | 0.563265889783094 | 0.810832039687759 | 3936.9620791655 | 768.908670907193 | 1034.74672881937 | 1552.67218003849 | 1360.15027591144 | 1694.07619869168 |
| YDL183C | 40.523925178302 | -0.456441291942587 | 0.514466536096412 | -0.887212792120359 | 0.374964346578418 | 0.687096411016019 | 55.4787576029508 | 27.1089595512151 | 18.7680785154662 | 38.4016515116473 | 57.8787351451678 | 45.5073687433649 |
| YDL184C | 1842.3383917207 | -0.0892083784904947 | 0.421044639417552 | -0.211873920575026 | 0.832205397062504 | 0.940112098796406 | 1012.82561136119 | 2124.35664846795 | 2219.63808576247 | 2030.09811775087 | 2508.82055802324 | 1158.29132895848 |
| YDL185C-A | 1.33228566846862 | -1.29258775891235 | 2.01339476938917 | -0.641994197344867 | 0.520876950622111 | NA | 0 | 1.23222543414614 | 1.25120523436441 | 2.07576494657553 | 0 | 3.43451839572565 |
| YDL185W | 37045.0338307955 | 1.02588580822062 | 0.347707425383341 | 2.95042824319786 | 0.0031733374739096 | 0.0522951511407256 | 30104.6682719622 | 58644.0728618832 | 60315.5995277708 | 24156.7145657727 | 27011.5604712102 | 22037.5872861736 |
| YDL186W | 7.097169352705 | -1.21095882079615 | 0.85659763428305 | -1.41368452623581 | 0.157454550652357 | NA | 2.0297106440104 | 6.16112717073071 | 5.00482093745765 | 8.30305978630211 | 13.3566311873464 | 7.72766639038271 |
| YDL187C | 157.168735620227 | 0.351458857232746 | 0.446772494607902 | 0.786661805447971 | 0.431479868438284 | 0.731159519481642 | 79.1587151164054 | 247.677312263375 | 202.695247967035 | 137.000486473985 | 150.262100857647 | 126.218551042918 |
| YDL188C | 2506.08061504197 | 0.413500528817144 | 0.314436438680796 | 1.31505283087408 | 0.188492147653726 | 0.501366434388531 | 1972.8787459781 | 3165.58714032144 | 3450.82403637705 | 2122.46965787348 | 2301.79277461937 | 2022.93133508241 |
| YDL189W | 118.101126631577 | 0.316817888435559 | 0.33142846571993 | 0.955916347581571 | 0.339114499135036 | 0.653227259057704 | 143.432885510068 | 118.29364167803 | 131.376549608263 | 99.6367174356253 | 87.9311553166972 | 127.93581024078 |
| YDL190C | 996.455047294713 | 0.223930669032362 | 0.334635709753236 | 0.669177444324428 | 0.503382292732645 | 0.778225233627554 | 1499.95616592368 | 938.95578081936 | 780.752066243393 | 920.601753806246 | 887.102921359591 | 951.361595616005 |
| YDL191W | 3986.88969936057 | -0.861399347618786 | 0.473484181391793 | -1.8192779853526 | 0.068869030274554 | 0.300604895539155 | 5176.43871244118 | 1564.9263013656 | 1749.18491764145 | 5937.7256296793 | 4207.33882401412 | 5285.72381102178 |
| YDL192W | 12413.8542147244 | 0.295104637346113 | 0.299879996565597 | 0.984075766059175 | 0.325078261342736 | 0.641231930747442 | 9854.24517667047 | 15179.7851232463 | 16004.1661527552 | 11308.7674289435 | 11711.5394461049 | 10424.6219606263 |
| YDL193W | 490.753811678527 | 0.33743815304684 | 0.353664602928999 | 0.954119101126395 | 0.340023358378131 | 0.65358631663837 | 773.996325582631 | 487.961271921872 | 380.366391246781 | 436.948521254148 | 479.725670145525 | 385.524689920204 |
| YDL194W | 172.91560696723 | 0.138133005122572 | 0.43560440747345 | 0.317106536923621 | 0.751162764922137 | 0.908603445448746 | 296.337754025518 | 107.203612770714 | 138.88378101445 | 152.568723573301 | 153.601258654484 | 188.898511764911 |
| YDL195W | 2434.4822925169 | 0.845840550037596 | 0.553822567267435 | 1.52727714620042 | 0.126692109888059 | 0.407215191710193 | 6511.31174598535 | 1452.7937868583 | 1420.11794100361 | 1618.05877585562 | 1630.62205745521 | 1973.98944794332 |
| YDL196W | 4.13957534795139 | 0.123447535530556 | 1.08857180887588 | 0.113403208244052 | 0.909710896310489 | NA | 2.0297106440104 | 8.625578039023 | 2.50241046872882 | 2.07576494657553 | 4.45221039578214 | 5.15177759358848 |
| YDL197C | 17.6642664168174 | -0.640365576103743 | 0.76282123954028 | -0.839470039520221 | 0.401205594936732 | 0.707577598073053 | 30.4456596601559 | 4.92890173658457 | 5.00482093745765 | 22.8334144123308 | 25.6002097757473 | 17.1725919786283 |
| YDL198C | 8356.0948039836 | 0.105142487299576 | 0.370756206365646 | 0.283589284533466 | 0.776725149826532 | 0.920342502126552 | 4722.46009839752 | 10260.7411901349 | 10999.3452152975 | 8139.07435552264 | 8345.66838689362 | 7669.27957765538 |
| YDL199C | 261.863768429591 | 0.235377930713813 | 0.358940936649561 | 0.65575671839185 | 0.511980668684268 | 0.781637419282118 | 184.027098390276 | 362.274277638966 | 304.042871950552 | 220.031084337006 | 282.715360132166 | 218.091918128579 |
| YDL200C | 287.964739942191 | -0.561834527166471 | 0.419337893603799 | -1.33981339567968 | 0.180306019465253 | 0.49089338447945 | 114.340366279252 | 292.037427892636 | 292.782024841272 | 406.849929528803 | 286.054517929002 | 335.724173182182 |
| YDL201W | 526.56581409154 | 0.894463185064498 | 0.501484980162823 | 1.78362906257747 | 0.0744839599009309 | 0.311823599060853 | 1299.01481216665 | 409.098844136519 | 345.332644684578 | 337.311803818523 | 335.028832282606 | 433.607947460363 |
| YDL202W | 74.2446316139105 | 0.45434108064903 | 0.744075488794934 | 0.610611540752212 | 0.541456780592223 | 0.798861248305202 | 208.383626118401 | 25.876734117069 | 22.5216942185594 | 59.1593009774025 | 44.5221039578214 | 85.0043302942098 |
| YDL203C | 282.194518597451 | 0.451822786584902 | 0.557191362570285 | 0.810893378714047 | 0.417426903034082 | 0.7183742031856 | 666.42166145008 | 140.47369949266 | 170.16391187356 | 244.940263695912 | 198.123362612305 | 273.044212460189 |
| YDL204W | 129.624171635935 | 1.01493190357748 | 0.667052743500256 | 1.52151672182889 | 0.128130218259408 | 0.408708376556648 | 397.823286226038 | 64.0757225755994 | 57.5554407807629 | 68.5002432369924 | 109.079154696662 | 80.7111822995528 |
| YDL205C | 1135.15807613607 | 0.118299417086566 | 0.273937162861366 | 0.431848734399117 | 0.665851356662126 | 0.868260547470569 | 987.115943203722 | 1230.993208712 | 1327.52875366064 | 1140.63283814325 | 1147.55722951285 | 977.120483583947 |
| YDL206W | 574.617690216125 | 0.663930851366654 | 0.359046652878016 | 1.84914925691347 | 0.0644362635065094 | 0.289834447065002 | 477.658571557113 | 826.823266312061 | 809.529786633774 | 413.07722436853 | 575.448193654841 | 345.169098770428 |
| YDL207W | 304.590180955331 | 0.00182306949114643 | 0.348267542915912 | 0.00523468100381264 | 0.995823347921029 | 0.998254726866075 | 194.175651610328 | 367.20317937555 | 354.091081325128 | 287.49344510071 | 328.350516688933 | 296.227211631337 |
| YDL208W | 1398.32864408877 | 0.486327933268372 | 0.546085537211332 | 0.890570982252852 | 0.373159372315356 | 0.685851509845389 | 3290.83752415552 | 837.913295219377 | 765.73760343102 | 1174.88295976175 | 851.485238193334 | 1469.11524377165 |
| YDL209C | 68.0480374466769 | -0.092088480454874 | 0.369672777572697 | -0.249108092458241 | 0.803277169866004 | 0.930747663909662 | 77.8055746870652 | 49.2890173658457 | 70.067493124407 | 71.6138906568557 | 65.6701033377865 | 73.8421455081015 |
| YDL210W | 481.170229494934 | 0.831908606191753 | 0.451357662782861 | 1.84312503096235 | 0.0653107574677568 | 0.292600338210963 | 326.106843471004 | 814.5010119706 | 708.182162650257 | 272.963090474682 | 523.134721504401 | 242.133546898658 |
| YDL211C | 86.2688134111508 | -0.844225493314551 | 0.419119985739648 | -2.01428116539156 | 0.0439800222678998 | 0.234941605944134 | 92.0135491951379 | 49.2890173658457 | 42.54097796839 | 121.432249374668 | 91.2703131135338 | 121.066773449329 |
| YDL212W | 11194.5489256912 | 0.071785749361034 | 0.347607112954823 | 0.206514040379731 | 0.836389398478532 | 0.941803181712136 | 6842.83115117371 | 13510.1196599783 | 14067.3004499591 | 11411.517793799 | 10733.1662116318 | 10602.3582876051 |
| YDL213C | 53.663164702992 | 0.569250190691719 | 0.573906332438233 | 0.991886930874708 | 0.321252685087115 | 0.638699024600408 | 119.752927996613 | 41.8956647609688 | 30.0289256247459 | 33.2122391452084 | 37.8437883641482 | 59.2454423262675 |
| YDL214C | 14.7827660554684 | 0.689487597213042 | 0.695161630716912 | 0.991837821230125 | 0.321276644675736 | 0.638699024600408 | 33.1519405188365 | 12.3222543414614 | 8.75843664055088 | 9.34094225958987 | 12.2435785884009 | 12.8794439839712 |
| YDL215C | 1654.270329015 | 0.00811017152581208 | 0.327893495609392 | 0.024734164094165 | 0.980267004403716 | 0.99431092046569 | 1096.72031798028 | 1875.44711077043 | 2005.68199068615 | 1699.01360877207 | 1764.18836932867 | 1484.57057655241 |
| YDL216C | 43.5099202149858 | 0.451901486768154 | 0.532695697259747 | 0.848329523014344 | 0.396254480192189 | 0.702767896372941 | 81.864995975086 | 46.8245664975534 | 21.270488984195 | 38.4016515116473 | 40.0698935620392 | 32.6279247593937 |
| YDL217C | 1115.38893124137 | 0.166524142199915 | 0.280999325275003 | 0.592614028652717 | 0.553439488420328 | 0.804341385446282 | 936.373177103462 | 1228.5287578437 | 1375.07455256649 | 1031.65517844804 | 1096.35680996135 | 1024.34511152518 |
| YDL218W | 109.730601603749 | -0.352901906975631 | 0.351591985281341 | -1.00372568701542 | 0.315510852215862 | 0.635421652543671 | 73.7461533990444 | 108.435838204861 | 107.603650155339 | 115.204954534942 | 146.922943060811 | 106.470070267495 |
| YDL219W | 636.000186646175 | -0.548069150213265 | 0.27263467877773 | -2.01026939298536 | 0.0444026844397131 | 0.236320953851362 | 441.800350179596 | 538.482514721864 | 570.549586870172 | 819.927153897333 | 695.657874340959 | 749.583639867123 |
| YDL220C | 214.041773352541 | 0.602062849950876 | 0.39179959837828 | 1.53666020190656 | 0.12437654178697 | 0.406181701839891 | 389.704443649996 | 197.156069463383 | 186.429579920297 | 184.743080245222 | 142.470732665028 | 183.746734171322 |
| YDL221W | 5.03154168569404 | 0.66811479729455 | 0.992852009589232 | 0.672924857724734 | 0.5009950969983 | NA | 2.70628085868053 | 8.625578039023 | 7.50723140618647 | 3.11364741986329 | 2.22610519789107 | 6.01040719251989 |
| YDL222C | 373.122194284137 | 1.29946528073698 | 0.38669945231972 | 3.36040114083893 | 0.000778293787178016 | 0.0219141653034008 | 294.308043381507 | 690.04624312184 | 608.085743901104 | 223.144731756869 | 235.967150976453 | 187.181252567048 |
| YDL223C | 87.596584399583 | -0.0425313155833158 | 0.328223467606741 | -0.129580361494061 | 0.896898441487197 | 0.966421109619065 | 79.1587151164054 | 86.25578039023 | 93.8403925773309 | 80.9548329164456 | 93.4964183114249 | 91.8733670856611 |
| YDL224C | 1597.92596713104 | -0.303924863250133 | 0.242548893006414 | -1.25304576525977 | 0.210189051858489 | 0.528738148832645 | 1445.1539785354 | 1370.23468277051 | 1475.17097131564 | 1831.8625653529 | 1764.18836932867 | 1700.94523548313 |
| YDL225W | 21.8512105117979 | -1.24566291381102 | 0.603068036026589 | -2.06554292284875 | 0.038871658618867 | 0.222765995753986 | 21.6502468694442 | 7.39335260487685 | 8.75843664055088 | 34.2501216184962 | 36.7307357652026 | 22.3243695722167 |
| YDL226C | 989.003578675995 | 0.149407841667098 | 0.599550234360347 | 0.249199872011558 | 0.803206178423926 | 0.930747663909662 | 2266.51021914494 | 354.880925034089 | 497.979683277036 | 980.798937256937 | 757.988819881909 | 1075.86288746106 |
| YDL227C | 310.87050109128 | 0.213364583255151 | 0.445887789660276 | 0.47851631778864 | 0.632282762976984 | 0.851680835678705 | 540.579601521435 | 195.923844029237 | 264.004304450891 | 336.273921345235 | 197.01031001336 | 331.431025187525 |
| YDL228C | 7.77556580750652 | 1.36826924088786 | 0.888792673365961 | 1.53946953197315 | 0.123689710520787 | NA | 4.05942128802079 | 14.7867052097537 | 15.0144628123729 | 2.07576494657553 | 5.56526299472767 | 5.15177759358848 |
| YDL229W | 167371.552435872 | 1.00775293453377 | 0.341296327498467 | 2.95272129624159 | 0.00314986207678717 | 0.0522125638442146 | 137194.231560595 | 254619.670359354 | 278871.125045609 | 114781.49848584 | 114040.030130163 | 104722.75903367 |
| YDL230W | 497.667168857931 | -0.227812587437381 | 0.327716160045635 | -0.695152132277083 | 0.486959966878115 | 0.766813881410433 | 569.672120752251 | 320.378612877997 | 484.216425699027 | 512.713941804155 | 612.178929420044 | 486.842982594111 |
| YDL231C | 1067.1925142263 | 0.245150407881436 | 0.243911548560993 | 1.00507913351275 | 0.31485874686738 | 0.635421652543671 | 1152.19907558323 | 1155.82745722908 | 1164.87207319327 | 1020.23847124187 | 967.24270848367 | 942.775299626691 |
| YDL232W | 570.736809324696 | -1.41641807234252 | 0.382997252608305 | -3.69824603883284 | 0.000217094407967765 | 0.00950348779483322 | 185.380238819616 | 332.700867219458 | 416.651343043349 | 785.677032278837 | 1035.13891701935 | 668.87245756757 |
| YDL233W | 193.018589165455 | 0.708776894190686 | 0.453636573322335 | 1.56243331308091 | 0.118185944385319 | 0.395918961445079 | 406.618699016749 | 128.151445151199 | 182.675964217204 | 154.644488519877 | 146.922943060811 | 139.097995026889 |
| YDL234C | 672.157430559137 | 0.398757753082746 | 0.400562485807149 | 0.995494503883044 | 0.319495816049332 | 0.638537064188392 | 1014.17875179053 | 763.979769170608 | 514.245351323773 | 400.622634689077 | 835.902501808097 | 504.015574572739 |
| YDL235C | 566.706214329822 | -0.510011512549214 | 0.430222668142003 | -1.18545941512518 | 0.235835852052473 | 0.562210636613656 | 760.464921289228 | 396.776589795058 | 243.98502070106 | 661.131135484305 | 586.578719644297 | 751.300899064986 |
| YDL236W | 8204.83636995057 | 0.949698490078384 | 0.34083259290758 | 2.78640749106968 | 0.0053295827844662 | 0.0743554413716304 | 6634.44752505531 | 12443.0124340077 | 13359.1182873088 | 5426.04957034843 | 5817.92593468831 | 5548.46446829479 |
| YDL237W | 3721.87339010417 | 0.474570392575238 | 0.342846399576586 | 1.38420701854046 | 0.166295074904357 | 0.470061345026585 | 2701.54486717784 | 4946.15289266262 | 5338.89273503294 | 2929.94222209136 | 3511.68094967316 | 2903.02667398711 |
| YDL238C | 431.090914161784 | 0.461991909720065 | 0.345496899644255 | 1.33718105776278 | 0.181163543437834 | 0.491656302021091 | 388.351303220656 | 524.928034946257 | 585.564049682545 | 296.8343873603 | 488.63009093709 | 302.237618823857 |
| YDL239C | 60.4188251025737 | 0.0686064953944471 | 0.399697788316486 | 0.171645921993753 | 0.863715902661379 | 0.953454774053698 | 44.6536341682287 | 70.2368497463301 | 71.3186983587715 | 71.6138906568557 | 52.3134721504401 | 52.3764055348162 |
| YDL240W | 218.313841469171 | 0.292860517532936 | 0.32534858468108 | 0.900143819036465 | 0.368043719398369 | 0.681769945236982 | 305.8097370309 | 207.013872936552 | 207.700068904492 | 186.818845191797 | 175.862310633394 | 226.678214117893 |
| YDL241W | 774.23440426469 | 0.551415592552102 | 0.350207745572379 | 1.57453854040512 | 0.115362998779564 | 0.391747850004865 | 712.428436047649 | 1003.03150339496 | 1046.00757592865 | 485.728997498673 | 869.294079776463 | 528.91583294175 |
| YDL242W | 9.8004786907007 | 0.446229942478059 | 0.776408886639501 | 0.574735748336753 | 0.565470026590611 | NA | 6.76570214670132 | 6.16112717073071 | 21.270488984195 | 9.34094225958987 | 6.67831559367321 | 8.58629598931413 |
| YDL243C | 286.687320391527 | 0.639705095934142 | 0.410994683778735 | 1.55648022026128 | 0.119593942536033 | 0.396897218069931 | 181.997387746265 | 446.065607160904 | 420.404958746442 | 181.629432825359 | 269.358728944819 | 220.667806925373 |
| YDL244W | 81.0863443371418 | 0.409397889017784 | 0.476459292840943 | 0.859250507166524 | 0.39020232344563 | 0.699637138162367 | 42.6239235242183 | 133.080346887783 | 102.598829217882 | 56.0456535575392 | 83.4789449209151 | 68.690367914513 |
| YDL248W | 378.768028067555 | 0.565461443840211 | 0.371411309021746 | 1.52246695268803 | 0.127892119847114 | 0.408360464021731 | 271.304656082723 | 516.302456907234 | 569.298381635807 | 281.266150260984 | 363.96819985519 | 270.468323663395 |
| YDR001C | 2184.78804072291 | 0.293929356089321 | 0.256890062982292 | 1.14418344048435 | 0.252547569127301 | 0.576133052094532 | 2459.33273032593 | 2400.37514571669 | 2359.77307201128 | 1724.96067060426 | 2227.21825049001 | 1937.06837518927 |
| YDR002W | 2812.86714987127 | 0.215953563687723 | 0.275349048168109 | 0.784290213183802 | 0.432869840778711 | 0.731159519481642 | 3574.99701431698 | 2887.10419220441 | 2606.26050318107 | 2643.48665946393 | 2320.71466880144 | 2844.63986125977 |
| YDR003W | 1101.89224808538 | 0.377451551643348 | 0.352508539764744 | 1.07075860316817 | 0.284277984930668 | 0.608839674843081 | 795.646572452075 | 1430.61372904367 | 1510.20471787784 | 817.851388950758 | 1170.9313340907 | 886.105746097218 |
| YDR003W-A | 254.093805115753 | 0.66715589987712 | 0.404972114226647 | 1.6474119487243 | 0.0994734010247773 | 0.361652820424048 | 181.997387746265 | 359.809826770674 | 394.12964882479 | 167.09907819933 | 262.680413351146 | 158.846475802311 |
| YDR004W | 351.655783133293 | -0.342839035215344 | 0.353864049263489 | -0.968843927290462 | 0.332623065795363 | 0.647455782833059 | 431.651796959544 | 213.175000107283 | 284.023588200721 | 441.1000511473 | 356.176831662571 | 383.807430722341 |
| YDR005C | 1167.66593539008 | -0.0835456611765517 | 0.263972890228985 | -0.31649333802452 | 0.751628080987306 | 0.908783805114648 | 983.733092130372 | 1302.46228389247 | 1116.07506905305 | 1213.2846112734 | 1232.14922703271 | 1158.29132895848 |
| YDR006C | 1124.93186301674 | -0.0529136158874135 | 0.249928823367254 | -0.211714740118871 | 0.832329587817486 | 0.940112098796406 | 1175.20246288202 | 1030.14046294617 | 1107.3166324125 | 1138.55707319668 | 1088.56544176873 | 1209.80910489436 |
| YDR007W | 6393.16660614673 | 0.134767984606382 | 0.388578104176873 | 0.346823413768673 | 0.728723986019525 | 0.89906903300167 | 3400.44189893208 | 8000.8397439109 | 8674.60588984846 | 6446.2880415903 | 5821.26509248515 | 6015.55897011348 |
| YDR008C | 22.7749322541174 | 0.327707168936346 | 0.593036474602421 | 0.552591928103655 | 0.580542870492048 | 0.819257784137336 | 10.8251234347221 | 27.1089595512151 | 38.7873622652968 | 18.6818845191797 | 18.9218941820741 | 22.3243695722167 |
| YDR009W | 86.235105309986 | 0.0470450217172836 | 0.4671657125489 | 0.100703070566976 | 0.919786173807921 | 0.977974644292995 | 146.139166368748 | 57.9145954048687 | 57.5554407807629 | 77.8411854965823 | 102.400839102989 | 75.5594047059643 |
| YDR010C | 30.5737480250004 | 0.974975482936468 | 0.547957974742828 | 1.7792887919809 | 0.0751924275332164 | 0.313647368259019 | 18.9439660107637 | 54.2179191024303 | 48.797004140212 | 21.795531939043 | 23.3741045778562 | 16.3139623796968 |
| YDR011W | 2872.18009449253 | 0.430566883989843 | 0.309112441587197 | 1.3929134711596 | 0.163645958285639 | 0.465094118506563 | 2328.75467889459 | 3641.22615790185 | 3923.77961496679 | 2621.69112752489 | 2484.33340084643 | 2233.2955868206 |
| YDR012W | 71259.32047619 | 0.778081658984785 | 0.376953570498729 | 2.06413128798685 | 0.039005270150313 | 0.22284687967803 | 47621.0711297719 | 108699.534447768 | 113748.319061303 | 53463.4019639993 | 51010.0875570749 | 53013.5086972233 |
| YDR013W | 376.479336556292 | 0.303669257718418 | 0.339294979549684 | 0.895000739832493 | 0.370786692489117 | 0.683760637929883 | 272.657796512063 | 467.013439541388 | 509.240530386315 | 312.402624459617 | 358.402936860462 | 339.158691577908 |
| YDR014W | 49.4312472596201 | 0.279341640019343 | 0.457604935384237 | 0.610442804304083 | 0.541568520615412 | 0.798861248305202 | 30.4456596601559 | 69.004624312184 | 63.811466952585 | 41.5152989315106 | 52.3134721504401 | 39.496961550845 |
| YDR014W-A | 3.88167298563513 | -1.384674902429 | 1.22113728755285 | -1.13392238247337 | 0.256827107466315 | NA | 4.73599150269092 | 0 | 1.25120523436441 | 8.30305978630211 | 5.56526299472767 | 3.43451839572565 |
| YDR016C | 544.267382712573 | -0.66705007103602 | 0.365836440038072 | -1.82335600840256 | 0.0682494885474921 | 0.299391505268986 | 242.888707066577 | 521.231358643818 | 499.2308885114 | 664.244782904169 | 722.371136715652 | 615.637422433823 |
| YDR017C | 268.590071649576 | 0.619807286271911 | 0.301266418492386 | 2.05733944517807 | 0.039653585576097 | 0.22284687967803 | 374.143328712583 | 320.378612877997 | 281.521177731993 | 215.879554443855 | 179.201468430231 | 240.416287700796 |
| YDR018C | 77.247480143111 | -0.0623714522816756 | 0.425743486673059 | -0.146500073950802 | 0.883526631544123 | 0.961475024255134 | 46.006774597569 | 77.630202351207 | 103.850034452246 | 68.5002432369924 | 97.948628707207 | 69.5489975134444 |
| YDR019C | 350.054005134856 | -1.28971325634583 | 0.400612498392025 | -3.21935351873061 | 0.00128479983474992 | 0.0295401178005701 | 320.694281753642 | 128.151445151199 | 158.90306476428 | 524.130649010321 | 454.125460369778 | 514.319129759916 |
| YDR020C | 233.597364501702 | -0.159578105444859 | 0.462949630720359 | -0.344698634269461 | 0.730320944979627 | 0.899838143479963 | 381.585601073954 | 149.099277531683 | 130.125344373899 | 257.394853375365 | 191.445047018632 | 291.93406363668 |
| YDR021W | 84.959727255877 | 0.429398111838937 | 0.402250189702256 | 1.06749014128937 | 0.285750563142554 | 0.610456523844073 | 135.314042934026 | 76.3979769170608 | 80.0771349993223 | 88.2200102294599 | 56.7656825462223 | 72.9835159091701 |
| YDR022C | 93.4960143528319 | -0.244490243933709 | 0.36882673482839 | -0.662886447338117 | 0.507403282225889 | 0.779869145077428 | 71.0398725403638 | 108.435838204861 | 77.5747245305935 | 102.750364855489 | 121.322733285063 | 79.8525527006214 |
| YDR023W | 6036.80829220081 | 0.443217573867215 | 0.305599997808548 | 1.45031929661492 | 0.146969500413603 | 0.440892991564844 | 9128.28533632942 | 6416.19783559896 | 5325.12947745494 | 5088.73776652991 | 4925.25775033399 | 5337.24158695766 |
| YDR024W | 199.120911067924 | 0.0275183898792865 | 0.290271483665973 | 0.0948022504027746 | 0.924471899362234 | 0.978676334770653 | 175.231685599564 | 204.54942206826 | 223.96573695123 | 201.349199817826 | 186.99283662285 | 202.636585347813 |
| YDR025W | 33960.2795058203 | -0.68043095564435 | 0.331389097600807 | -2.05326898371292 | 0.0400464972429851 | 0.223693556136616 | 36825.7167844953 | 19424.8017438798 | 22038.7289980947 | 41158.2673606996 | 40663.1505972772 | 43651.0115504752 |
| YDR026C | 30.4592530497463 | -0.281095649473232 | 0.507516074692196 | -0.553865509863336 | 0.579670890842614 | 0.819257784137336 | 41.9473533095482 | 19.7156069463383 | 20.0192837498306 | 36.3258865650717 | 27.8263149736384 | 36.9210727540507 |
| YDR027C | 350.844044106859 | -0.0324160385948777 | 0.277725379491754 | -0.116719756236178 | 0.907082134072782 | 0.9727440497482 | 301.750315742879 | 384.454335453596 | 355.342286559493 | 346.652746078113 | 333.91577968366 | 382.94880112341 |
| YDR028C | 84.3857019655196 | 0.439962193276674 | 0.54344554746543 | 0.809579166355505 | 0.418182085964798 | 0.719121113252534 | 186.056809034286 | 48.0567919316995 | 56.3042355463985 | 60.1971834506903 | 80.1397871240785 | 75.5594047059643 |
| YDR030C | 89.6317757656268 | 0.102232075568437 | 0.347956771322726 | 0.293806828876505 | 0.768905516593015 | 0.917134033902605 | 115.016936493922 | 85.0235549560838 | 77.5747245305935 | 93.4094225958987 | 83.4789449209151 | 83.287071096347 |
| YDR031W | 313.055566369663 | -0.726425275188779 | 0.373135170720914 | -1.9468153425079 | 0.0515568769001238 | 0.257470832686283 | 146.139166368748 | 263.696242907274 | 299.038051013094 | 482.61535007881 | 352.837673865734 | 334.00691398432 |
| YDR032C | 8676.41727427131 | 0.123265474015742 | 0.388258315163146 | 0.317483152843605 | 0.750877020860438 | 0.908603445448746 | 4582.4100639608 | 11199.6969709543 | 11359.6923227945 | 7879.6037372007 | 8482.57385656392 | 8554.52669415366 |
| YDR033W | 50982.5694324564 | 0.614234584673084 | 0.340322470269787 | 1.8048605024116 | 0.0710965138709524 | 0.303855444880757 | 38555.7068234068 | 73825.0902105637 | 72643.7247019634 | 41331.5937337386 | 43931.0730277813 | 35608.2280972846 |
| YDR034C | 441.923574401441 | 0.125546899485501 | 0.296387757493455 | 0.42359003134019 | 0.67186482664812 | 0.87058290537205 | 562.90641860555 | 410.331069570665 | 409.144111637163 | 479.501702658947 | 400.698935620392 | 388.95920831593 |
| YDR034C-A | 3.63991009949866 | -1.3170498316262 | 1.24788087997945 | -1.05542913010086 | 0.291229106036908 | NA | 1.35314042934026 | 0 | 5.00482093745765 | 6.22729483972658 | 6.67831559367321 | 2.57588879679424 |
| YDR034W-B | 279.556395699205 | -0.672407152769374 | 0.392771028227227 | -1.7119571059105 | 0.0869045735548915 | 0.337746780793453 | 134.637472719356 | 243.980635960936 | 269.009125388348 | 315.51627187948 | 457.464618166615 | 256.730250080492 |
| YDR035W | 5885.44418120416 | 0.35148019411453 | 0.290415487620668 | 1.21026670097437 | 0.226176570819728 | 0.549013061263428 | 5290.77907872043 | 6674.96517676965 | 7831.29356188685 | 5285.93543645458 | 5803.45625090202 | 4426.23558249143 |
| YDR036C | 360.411957223936 | -0.0982601459556664 | 0.740343755522344 | -0.132722326922768 | NA | NA | 878.188138641831 | 81.3268786536454 | 83.8307507024156 | 381.940750169897 | 313.880832902641 | 423.304392273186 |
| YDR037W | 13174.9095292459 | 0.51074220535298 | 0.432494938015898 | 1.18092065469262 | 0.23763424525969 | 0.564156198917009 | 26532.3775385039 | 10160.9309299691 | 9754.39600710495 | 11483.1316844558 | 9168.21425751437 | 11950.4067579274 |
| YDR038C | 606.878346552402 | 0.959566348068015 | 0.39715018918621 | 2.41612965118873 | 0.0156864747422227 | 0.136202200631867 | 418.796962880812 | 1072.03612770714 | 914.631026320385 | 359.107335757566 | 469.708196755016 | 406.99042989349 |
| YDR040C | 198.550441098975 | 1.20329168116511 | 0.354904888531996 | 3.39046240287736 | 0.00069774824445541 | 0.0204590750188728 | 184.703668604946 | 348.719797863358 | 297.78684577873 | 107.939777221927 | 130.227154076628 | 121.925403048261 |
| YDR041W | 1100.39593588112 | -1.33982768854137 | 0.287389636630976 | -4.66205985799613 | 3.13060100745295e-06 | 0.000391189012844338 | 594.028648480376 | 575.449277746249 | 700.67493124407 | 1844.31715503236 | 1271.1060679958 | 1616.79953478785 |
| YDR042C | 70.7611787821456 | -0.492892611718101 | 0.358270232651782 | -1.37575652900297 | 0.168897024122261 | 0.473570777880368 | 46.6833448122391 | 62.8434971414533 | 67.5650826556782 | 83.0305978630211 | 84.5919975198606 | 79.8525527006214 |
| YDR043C | 460.060753059167 | -0.035597291038965 | 0.374825998276614 | -0.0949701760353746 | 0.924338515898236 | 0.978676334770653 | 625.150878355202 | 427.582225648711 | 309.04769288801 | 431.75910888771 | 601.048403430589 | 365.776209144782 |
| YDR044W | 9035.55995414597 | 0.449376830713571 | 0.354996607495049 | 1.2658623243881 | 0.205562377028388 | 0.523757231214639 | 7578.26297452015 | 11369.7440808665 | 12346.893252708 | 5855.73291428956 | 10521.6862178321 | 6541.0402846595 |
| YDR045C | 788.7597120532 | 0.0599577621089093 | 0.391836948767405 | 0.153017121783735 | 0.878384773066668 | 0.959113330331498 | 422.179813954162 | 977.154769277891 | 1017.22985553827 | 893.616809500764 | 734.614715304053 | 687.762308744061 |
| YDR046C | 10176.0014771767 | 0.8522538246338 | 0.348431359015536 | 2.44597336772951 | 0.0144461671097764 | 0.129542228622457 | 7942.25775001268 | 15439.7846898512 | 15910.3257601779 | 6900.88056489034 | 8162.0147080676 | 6700.74539006074 |
| YDR047W | 6195.18530009822 | 1.19355109602856 | 0.35544575974023 | 3.35789937936196 | 0.000785371964596481 | 0.0219141653034008 | 5013.38529070568 | 9886.1446581545 | 10964.3114687353 | 3703.16466469074 | 3925.7365164809 | 3678.36920182217 |
| YDR048C | 18.7878714981553 | -0.287718592550824 | 0.526942455865238 | -0.546015204029044 | 0.585055486111826 | 0.822828219762852 | 14.8845447227429 | 16.0189306438998 | 20.0192837498306 | 18.6818845191797 | 23.3741045778562 | 19.7484807754225 |
| YDR049W | 20.4641861005158 | -0.0916641014036477 | 0.547922505125096 | -0.16729391573854 | 0.867138791215375 | 0.955032337977769 | 27.7393788014754 | 12.3222543414614 | 18.7680785154662 | 19.7197669924675 | 24.4871571768018 | 19.7484807754225 |
| YDR050C | 251860.179856659 | 0.640382813610607 | 0.375302674795954 | 1.70631028398285 | 0.0879503243612355 | 0.340200850893931 | 164937.669783358 | 370899.855677989 | 384736.851130416 | 185094.922403666 | 214602.106339694 | 190889.673804833 |
| YDR051C | 736.087736313983 | -0.308539045937 | 0.282261527374343 | -1.09309635219187 | 0.274351497440781 | 0.600152316299675 | 560.200137746869 | 651.847254663309 | 761.983987727927 | 862.480335302132 | 706.788400330415 | 873.226302113247 |
| YDR052C | 56.5890681799345 | -0.152928636690443 | 0.371933645168342 | -0.411171827763055 | 0.680946544337709 | 0.87491979660087 | 57.5084682469612 | 55.4501445365764 | 47.5457989058476 | 69.5381257102802 | 54.5395773483312 | 54.9522943316104 |
| YDR053W | 1.23604541353542 | -0.851541137254219 | 1.97775031882678 | -0.430560485389966 | 0.66678797802114 | NA | 1.35314042934026 | 1.23222543414614 | 0 | 3.11364741986329 | 0 | 1.71725919786283 |
| YDR054C | 22.6121933666321 | 1.38977741002169 | 0.754978840761032 | 1.84081637125191 | 0.0656484728816474 | 0.29342723337769 | 71.716442755034 | 11.0900289073153 | 15.0144628123729 | 13.4924721527409 | 8.90442079156428 | 15.4553327807654 |
| YDR055W | 16990.2990913601 | 0.245891201285193 | 0.348610981104195 | 0.705345541630256 | 0.480595225536038 | 0.764853084984975 | 13071.3365474269 | 22981.0043468256 | 19252.2949411652 | 12862.4774914553 | 20598.1513960861 | 13176.5298252015 |
| YDR056C | 1269.23392953924 | 0.130646935098924 | 0.347624728189783 | 0.375827507379159 | 0.707045136534577 | 0.88970915496033 | 845.712768337665 | 1484.8316481461 | 1650.33970412666 | 1215.36037621997 | 1399.10711687454 | 1020.05196353052 |
| YDR057W | 70.8455039030284 | 0.49359302627169 | 0.633881854401509 | 0.77868300353498 | 0.436166470614866 | 0.734586647329215 | 174.555115384894 | 48.0567919316995 | 25.0241046872882 | 72.6517731301435 | 48.9743143536035 | 55.8109239305418 |
| YDR058C | 240.937793752093 | -0.820159656198873 | 0.314395895297284 | -2.60868436409881 | 0.00908910357599978 | 0.099135042419064 | 130.578051431335 | 209.478323804844 | 183.927169451568 | 298.910152306876 | 312.767780303695 | 309.96528521424 |
| YDR059C | 869.714372862585 | -0.24534317397619 | 0.399062793468145 | -0.614798417672517 | 0.538687854406941 | 0.798710611079734 | 399.852996870048 | 985.780347316914 | 1003.46659796026 | 843.798450782952 | 1064.07828459193 | 921.309559653406 |
| YDR060W | 90.8398823429798 | 0.333847467651216 | 0.47342126534452 | 0.705180548677438 | 0.480697884598861 | 0.764853084984975 | 172.525404740884 | 70.2368497463301 | 60.0578512494917 | 69.5381257102802 | 86.8181027177517 | 85.8629598931413 |
| YDR061W | 570.664955990348 | -0.252747688378288 | 0.433259303906752 | -0.583363556418133 | 0.559648585621325 | 0.809182184103241 | 887.660121647213 | 321.610838312143 | 351.5886708564 | 666.320547850744 | 555.413246873822 | 641.396310401765 |
| YDR062W | 4733.69881671953 | 0.725666700202044 | 0.242421138030105 | 2.99341347086626 | 0.00275875724231316 | 0.0483455385024879 | 5668.98182872103 | 6019.4212458039 | 6010.78994588663 | 3798.64985223321 | 3584.02936860462 | 3320.32065906777 |
| YDR063W | 698.805084357158 | -0.623949052758449 | 0.27011631321845 | -2.30992732472935 | 0.0208921779924215 | 0.159076280224254 | 643.418274151295 | 515.070231473087 | 490.472451870849 | 826.15444873706 | 835.902501808097 | 881.812598102561 |
| YDR064W | 96042.4053132851 | -0.0134440612087846 | 0.335631793611026 | -0.0400559823732472 | 0.96804849459273 | 0.990124204883325 | 59797.9818534049 | 107352.712048246 | 119635.239688988 | 97015.0263081004 | 97876.2802882756 | 94577.1916926962 |
| YDR065W | 31.2094954022288 | 0.472811260592266 | 0.588168203005695 | 0.803870828406017 | 0.421471579021178 | 0.721522771990525 | 62.2444597496521 | 32.0378612877997 | 13.7632575780085 | 29.0607092520574 | 27.8263149736384 | 22.3243695722167 |
| YDR066C | 246.90441448119 | 0.112071080308699 | 0.325953326236981 | 0.343825545830505 | 0.730977487382787 | 0.900098242818393 | 190.116230322307 | 276.018497248736 | 304.042871950552 | 250.129676062351 | 259.34125555431 | 201.777955748882 |
| YDR067C | 280.298512495443 | -0.175943160737244 | 0.271281217718937 | -0.648563738458044 | 0.516620400043658 | 0.783931905874062 | 252.360690071959 | 279.715173551174 | 257.748278279069 | 282.304032734272 | 319.446095897368 | 290.216804438817 |
| YDR068W | 12.808511200698 | -0.314466690042681 | 0.649558838895908 | -0.4841234869149 | 0.628298233639499 | 0.850071340307741 | 14.2079745080728 | 9.85780347316914 | 10.0096418749153 | 8.30305978630211 | 15.5827363852375 | 18.8898511764911 |
| YDR069C | 335.855799151737 | 0.0777158838015141 | 0.298532548911296 | 0.260326333208665 | 0.794612061696758 | 0.929585587537483 | 290.925192308157 | 420.188873043835 | 324.062155700383 | 319.667801772631 | 360.629042058353 | 299.661730027063 |
| YDR070C | 160.650546943889 | -1.16292723181287 | 0.344825197405639 | -3.37251233541628 | 0.000744857498776628 | 0.0213717267707159 | 75.0992938283846 | 115.829190809737 | 107.603650155339 | 203.424964764402 | 270.471781543765 | 191.474400561705 |
| YDR071C | 665.255961307596 | -0.132809153383709 | 0.47031181657304 | -0.282385321192719 | 0.777648069185719 | 0.920342502126552 | 1077.77635196952 | 450.994508897488 | 374.110365074959 | 801.245269378154 | 429.638303192976 | 857.770969332481 |
| YDR072C | 4864.87261557756 | 0.434996833447606 | 0.369680366413895 | 1.17668362447083 | 0.239321806276245 | 0.564935230380965 | 3194.76455367236 | 7220.8410440964 | 6363.6298219774 | 3767.51337803458 | 4847.3440684078 | 3795.14282727684 |
| YDR073W | 77.2067641184931 | -0.29285045223599 | 0.527983890333646 | -0.554657931042045 | 0.579128656299824 | 0.818699339992963 | 129.901481216665 | 43.127890195115 | 33.7825413278391 | 87.1821277561722 | 86.8181027177517 | 82.4284414974156 |
| YDR074W | 3140.47267654656 | 0.75999770196934 | 0.333060422140368 | 2.28186134241143 | 0.0224975277864925 | 0.164467129288005 | 4958.5831033174 | 3603.02716944332 | 3284.41374020658 | 1971.97669924675 | 3092.0601198707 | 1932.77522719461 |
| YDR075W | 444.885338632453 | 0.894420974774988 | 0.359568044404261 | 2.48748738575165 | 0.0128649015791295 | 0.12122533488006 | 344.374239267097 | 685.117341385255 | 706.930957415892 | 322.781449192495 | 313.880832902641 | 296.227211631337 |
| YDR076W | 27.5269293004757 | -0.288773689043449 | 0.514200895459407 | -0.561597017028622 | 0.57439061325353 | 0.816126268518437 | 35.1816511628469 | 23.4122832487767 | 15.0144628123729 | 37.3637690383595 | 26.7132623746928 | 27.4761471658052 |
| YDR077W | 16202.9519536668 | 0.568503771280208 | 0.590323526628447 | 0.963037632139007 | 0.335528638334138 | 0.650461589593466 | 41916.2310796733 | 9096.28815486682 | 7050.54149564346 | 10250.12730619 | 14805.8256711735 | 14098.6980144538 |
| YDR078C | 191.529003946447 | 0.241661938666739 | 0.415247381818912 | 0.581971011131209 | 0.560586207267641 | 0.809610432003619 | 106.898093917881 | 247.677312263375 | 269.009125388348 | 207.576494657553 | 159.166521649211 | 158.846475802311 |
| YDR079C-A | 184.523424062414 | -0.894879382039172 | 0.333579308357542 | -2.68265854511578 | 0.00730395219124984 | 0.086741977676248 | 94.0432598391483 | 135.544797756076 | 158.90306476428 | 259.470618321941 | 217.045256794379 | 242.133546898658 |
| YDR079W | 542.07450931296 | -0.539519528048904 | 0.324067139598507 | -1.66483873902588 | 0.0959449329037228 | 0.355730276849832 | 300.397175313539 | 529.856936682841 | 496.728478042671 | 651.790193224716 | 670.057664565212 | 603.616608048783 |
| YDR080W | 274.952865399719 | -0.0316454114766931 | 0.278304522593864 | -0.113707859224674 | 0.909469382173544 | 0.973410285303699 | 291.601762522827 | 257.535115736544 | 266.50671491962 | 286.455562627423 | 247.097676965909 | 300.520359625994 |
| YDR081C | 232.877589996256 | 0.140229614431949 | 0.412043524796803 | 0.34032718873838 | 0.733610144199105 | 0.901216308795993 | 391.057584079336 | 166.350433609729 | 173.917527576653 | 229.372026596596 | 197.01031001336 | 239.557658101864 |
| YDR082W | 161.795570824036 | -0.00483670366276588 | 0.438061036960267 | -0.0110411637983786 | 0.991190604860602 | 0.997612256476753 | 262.509243292011 | 125.686994282907 | 95.0915978116953 | 190.970375084949 | 140.244627467137 | 156.270587005517 |
| YDR083W | 418.537339797962 | 0.592951827795742 | 0.319368093646222 | 1.85664078407463 | 0.06336227743316 | 0.287228998963568 | 356.552503131159 | 600.093786429171 | 554.283918823434 | 361.183100704142 | 320.559148496314 | 318.551581203554 |
| YDR084C | 2159.55238337099 | 0.0361748325882259 | 0.363296028144457 | 0.0995739831591052 | 0.920682549580642 | 0.978384783691908 | 1237.44692263167 | 2613.55014582397 | 2710.11053763332 | 2040.47694248374 | 2268.401196651 | 2087.32855500226 |
| YDR085C | 86.2412030323703 | -0.443461378828537 | 0.364366086897063 | -1.21707643706649 | 0.223575134849093 | 0.546855266005357 | 90.6604087657977 | 71.4690751804763 | 56.3042355463985 | 84.0684803363089 | 110.192207295608 | 104.752811069632 |
| YDR086C | 2810.5754436972 | -0.350821918090694 | 0.323622829740288 | -1.08404564156439 | 0.278344562607195 | 0.603364250395172 | 1630.53421735502 | 2968.43107085806 | 2813.96057208556 | 3282.8222630092 | 3201.13927456736 | 2966.56526430803 |
| YDR087C | 452.680878087355 | 0.91196380514796 | 0.427031498436464 | 2.13558908063464 | 0.0327129222311659 | 0.203720343428756 | 964.789126119608 | 423.885549346273 | 384.120006949874 | 342.501216184962 | 239.30630877329 | 361.483061150125 |
| YDR088C | 2.27548557651452 | 0.451031081109155 | 1.44955872075693 | 0.311150610631101 | 0.755686127607307 | NA | 4.05942128802079 | 0 | 3.75361570309323 | 1.03788247328776 | 2.22610519789107 | 2.57588879679424 |
| YDR089W | 531.189351002564 | -0.164733281898758 | 0.281389538099408 | -0.585427884104779 | 0.558260044074468 | 0.808281796811093 | 541.256171736105 | 467.013439541388 | 494.226067573942 | 619.615836552795 | 459.690723364506 | 605.333867246646 |
| YDR090C | 880.488039742394 | 0.286552730472076 | 0.300212527981753 | 0.954499575345815 | 0.339830824156849 | 0.653512070007885 | 723.253559482371 | 1080.66170574617 | 1099.80940100632 | 803.321034324729 | 861.502711583844 | 714.379826310935 |
| YDR091C | 9041.88098837526 | 0.668542711687434 | 0.306681603724568 | 2.1799244022731 | 0.0292630657572895 | 0.191538651933637 | 8085.69063552274 | 12015.430208359 | 13200.2152225445 | 8015.5663412014 | 6464.60949467567 | 6469.77402794819 |
| YDR092W | 1948.98197039306 | -0.944465238582278 | 0.245780526245618 | -3.84271794437626 | 0.000121679278957133 | 0.0062623115683545 | 1268.5691525065 | 1276.5855497754 | 1453.90048233145 | 2529.31958740228 | 2595.63866074099 | 2569.87838960172 |
| YDR093W | 2637.74669381373 | 0.812875958431797 | 0.245492148003444 | 3.31120960504364 | 0.000928935994216708 | 0.0246061018191596 | 3336.16772853842 | 3163.12268945315 | 3585.9542016884 | 1916.9689281625 | 2016.85130928931 | 1807.41530575062 |
| YDR094W | 6.97101392406314 | -0.578822468766836 | 0.821435649562371 | -0.704647368391194 | 0.481029712482599 | NA | 3.38285107335066 | 4.92890173658457 | 8.75843664055088 | 7.26517731301435 | 8.90442079156428 | 8.58629598931413 |
| YDR095C | 12.8572865245904 | -0.844992210971129 | 0.701144309826931 | -1.20516161812638 | 0.228140916258943 | 0.551220675349477 | 3.38285107335066 | 11.0900289073153 | 13.7632575780085 | 14.5303546260287 | 18.9218941820741 | 15.4553327807654 |
| YDR096W | 184.394008067722 | 0.794247798375265 | 0.397628004045843 | 1.99746443986298 | 0.0457747536846402 | 0.239681256250554 | 360.61192441918 | 177.440462517044 | 162.656680467373 | 140.114133893848 | 132.453259274519 | 133.087587834369 |
| YDR097C | 612.555369516433 | 0.457104748402014 | 0.293643000051833 | 1.55666829558794 | 0.119549259697812 | 0.396897218069931 | 571.701831396261 | 712.22630093647 | 843.312327961613 | 567.721712888407 | 457.464618166615 | 522.90542574923 |
| YDR098C | 2796.94680456504 | -0.255761296162707 | 0.257820067511623 | -0.992014697037409 | 0.321190356063995 | 0.638699024600408 | 2886.92510599745 | 2240.18583927769 | 2521.17854724429 | 2949.66198908382 | 2970.73738658563 | 3212.99195920135 |
| YDR099W | 1649.9971918651 | 0.103518730012175 | 0.591870497250757 | 0.174900980016777 | 0.861157440202548 | 0.953240541779596 | 3676.4825465175 | 914.311272136438 | 535.515840307968 | 1566.16465219124 | 1444.7422734313 | 1762.76656660619 |
| YDR100W | 1873.3992858864 | -0.364311493440346 | 0.382235276467849 | -0.953107983142915 | 0.340535361724869 | 0.65358631663837 | 864.656734348428 | 1970.32846919968 | 2080.75430474802 | 1934.61293020839 | 2279.53172264045 | 2110.51155417341 |
| YDR101C | 4689.38964366237 | 0.701689196328563 | 0.304875803744037 | 2.30155751198175 | 0.0213601385380088 | 0.160243867202626 | 7209.53220752492 | 5140.84451125771 | 5072.38602011332 | 4302.02285177778 | 2960.71991319512 | 3450.83235810535 |
| YDR102C | 17.9392342235413 | -0.133474038831384 | 0.568963179192534 | -0.234591698922959 | 0.814525653079838 | 0.93404506551552 | 10.8251234347221 | 20.9478323804844 | 20.0192837498306 | 22.8334144123308 | 16.695788984183 | 16.3139623796968 |
| YDR103W | 854.157788406024 | -0.27491814258536 | 0.278878287779804 | -0.985799736415583 | 0.324231395142487 | 0.640660728524929 | 926.90119409808 | 745.496387658416 | 645.621900932036 | 932.018461012412 | 903.798710343774 | 971.110076391428 |
| YDR104C | 154.007401132057 | 0.182536017860002 | 0.30145354460824 | 0.605519560558559 | 0.544833831784892 | 0.800898308039913 | 173.201974955554 | 149.099277531683 | 168.912706639196 | 144.265663786999 | 128.001048878736 | 160.563735000174 |
| YDR105C | 2110.12616235869 | 0.45056156296193 | 0.336867763690062 | 1.33750275783726 | 0.181058582671396 | 0.491603558429469 | 1537.84409794521 | 2778.66835399955 | 2995.3853310684 | 1815.2564457803 | 1872.15447142639 | 1661.44827393228 |
| YDR106W | 39.596604757621 | -0.937028176792653 | 0.422538782583843 | -2.21761460820871 | 0.0265811209939378 | 0.182324920612356 | 23.6799575134546 | 32.0378612877997 | 26.2753099216526 | 52.932006137676 | 43.4090513588758 | 59.2454423262675 |
| YDR107C | 566.06412843454 | 0.352386285039393 | 0.366859059714957 | 0.960549496346609 | 0.336778732586345 | 0.65110931789534 | 368.730766995222 | 793.553179590116 | 743.21590921246 | 530.357943850047 | 520.90861630651 | 439.618354652883 |
| YDR108W | 394.655418775154 | -0.1549264437801 | 0.321106796341454 | -0.482476377159444 | 0.629467575685941 | 0.850071340307741 | 472.922580054422 | 346.255346995066 | 300.289256247459 | 481.577467605522 | 346.159358272061 | 420.728503476392 |
| YDR109C | 236.772937377489 | 0.498917478704848 | 0.325252912635288 | 1.53393700509084 | 0.125045151121242 | 0.406181701839891 | 353.169652057809 | 229.193930751182 | 248.989841638518 | 187.856727665085 | 174.749258034449 | 226.678214117893 |
| YDR110W | 64.1764611079369 | 0.850971012169003 | 0.503642435772423 | 1.68963326305873 | 0.0910981381503118 | 0.346317525190471 | 142.756315295398 | 55.4501445365764 | 48.797004140212 | 39.439533984935 | 44.5221039578214 | 54.093664732679 |
| YDR111C | 1150.90743011142 | 1.70422578168258 | 0.330649553584703 | 5.15417535939908 | 2.54749470291613e-07 | 5.85719982094476e-05 | 1185.35101610207 | 1874.21488533628 | 2224.64290669992 | 556.305005682241 | 583.23956184746 | 481.691205000522 |
| YDR113C | 18.8051500795913 | -1.58893711927721 | 0.678919278358366 | -2.34039181081922 | 0.0192635187352357 | 0.152937438798529 | 18.2673957960936 | 4.92890173658457 | 3.75361570309323 | 26.9849443054819 | 32.2785253694205 | 26.6175175668738 |
| YDR114C | 9.04394425467641 | -0.201275674682946 | 0.770380212330443 | -0.261267970622034 | 0.79388586469908 | NA | 3.38285107335066 | 12.3222543414614 | 10.0096418749153 | 9.34094225958987 | 8.90442079156428 | 10.303555187177 |
| YDR115W | 1643.33854393423 | -1.12782340479074 | 0.346078995866987 | -3.25886117984524 | 0.00111860388688619 | 0.027331400297148 | 623.797737925861 | 1229.76098327785 | 1243.69800295822 | 2343.53862468377 | 2219.4268822974 | 2199.80903246228 |
| YDR116C | 1055.34556676229 | -0.941828862469169 | 0.322992638381816 | -2.91594528961312 | 0.00354612677792339 | 0.0569564557266088 | 983.056521915702 | 606.254913599902 | 576.805613041994 | 1411.52016367136 | 1254.41027901162 | 1500.02590933318 |
| YDR117C | 130.644010676045 | 0.421967519215217 | 0.634127798128996 | 0.665429776868699 | 0.505775644754716 | 0.779563554470966 | 333.549115832375 | 51.753468234138 | 62.5602617182206 | 103.788247328776 | 106.853049498771 | 125.359921443986 |
| YDR118W | 191.800524815384 | -0.180852520005785 | 0.326916938936376 | -0.55320633000599 | 0.580122132963741 | 0.819257784137336 | 225.297881485154 | 150.331502965829 | 162.656680467373 | 237.675086382898 | 193.671152216523 | 181.170845374528 |
| YDR118W-A | 7.9417558043312 | -0.0516456591893851 | 0.764919207076224 | -0.0675177962739254 | 0.946169494974505 | NA | 9.47198300538184 | 6.16112717073071 | 7.50723140618647 | 9.34094225958987 | 10.0174733905098 | 5.15177759358848 |
| YDR119W | 5451.34042178052 | 0.332176588111828 | 0.303506592614012 | 1.09446251315627 | 0.273752179760576 | 0.600152316299675 | 4501.22163820039 | 6333.63873151117 | 7394.62293509367 | 5430.20110024158 | 4365.39229306439 | 4682.96583257192 |
| YDR119W-A | 330.68956853453 | -0.985177876766306 | 0.43163565556466 | -2.28242932219654 | 0.0224640058222328 | 0.164467129288005 | 109.604374776561 | 273.554046380444 | 284.023588200721 | 391.281692429487 | 578.787351451678 | 346.886357968291 |
| YDR120C | 417.869095185953 | 2.13628015353436 | 0.760723747010478 | 2.80822067396948 | NA | NA | 1736.75574105823 | 144.170375795099 | 161.405475233009 | 135.962604000697 | 128.001048878736 | 200.919326149951 |
| YDR121W | 108.021432638394 | 0.607795232557434 | 0.657881599173023 | 0.923867202428904 | 0.355555460950057 | 0.669637218067146 | 293.631473166837 | 43.127890195115 | 53.8018250776697 | 81.9927153897333 | 65.6701033377865 | 109.904588663221 |
| YDR122W | 417.259689198741 | 0.229150082783284 | 0.316888300921985 | 0.723125726372898 | 0.469602645041367 | 0.758293350262115 | 593.352078265706 | 359.809826770674 | 396.632059293518 | 364.296748124005 | 407.377251214066 | 382.090171524479 |
| YDR123C | 145.985096116221 | 1.36289755829934 | 0.762729769362476 | 1.78686818457147 | 0.0739587957605639 | 0.310529699073573 | 529.754478086713 | 64.0757225755994 | 36.2849517965679 | 76.8033030232945 | 85.7050501188062 | 83.287071096347 |
| YDR124W | 234.650794043319 | -0.145612293812419 | 0.432998829014949 | -0.336287962126087 | 0.736653726708448 | 0.902255619245719 | 338.285107335066 | 182.369364253629 | 146.391012420636 | 201.349199817826 | 350.611568667843 | 188.898511764911 |
| YDR125C | 151.396639323655 | 0.0170379738304443 | 0.312487674383597 | 0.054523666778387 | 0.95651795326968 | 0.986781021449109 | 127.195200357985 | 172.51156078046 | 157.651859529916 | 155.682370993165 | 161.392626847103 | 133.9462174333 |
| YDR126W | 356.046140117243 | -0.222521527511221 | 0.311249090651627 | -0.714930691188054 | 0.474651871317592 | 0.760313273862685 | 268.598375224042 | 337.629768956043 | 380.366391246781 | 319.667801772631 | 460.803775963451 | 369.210727540507 |
| YDR127W | 9617.46621439619 | 0.289517964410257 | 0.280086018934959 | 1.03367517418814 | 0.301288042763976 | 0.625627510682729 | 8906.37030591762 | 9833.15896448622 | 12998.7711798119 | 8816.81161057955 | 9170.44036271226 | 7979.24486286962 |
| YDR128W | 601.844814631792 | -0.0419850182023471 | 0.328847621379522 | -0.127673169798885 | 0.898407623694132 | 0.967031487040734 | 815.267108677509 | 454.691185199927 | 507.989325151951 | 585.365714934299 | 605.500613826371 | 642.254940000697 |
| YDR129C | 10905.5549386333 | 0.533630562528138 | 0.280558133801206 | 1.90203205053485 | 0.0571669646413266 | 0.270749921086218 | 10243.2730501058 | 14265.4738511099 | 14191.1697681612 | 8446.28756761582 | 9980.74265474461 | 8306.38274006249 |
| YDR130C | 16.7521317601686 | -0.914154004321913 | 0.609334708681112 | -1.50024935605683 | 0.133549822622793 | 0.418919028611575 | 13.5314042934026 | 14.7867052097537 | 6.25602617182206 | 30.0985917253451 | 17.8088415831286 | 18.0312215775597 |
| YDR131C | 205.056870612919 | 0.0902622283129878 | 0.319012415594841 | 0.282942681540096 | 0.777220775681511 | 0.920342502126552 | 242.888707066577 | 223.032803580452 | 167.661501404831 | 187.856727665085 | 233.741045778562 | 175.160438182008 |
| YDR132C | 294.973049281546 | 0.0105868874623652 | 0.276465873255772 | 0.0382936502711521 | 0.969453553424383 | 0.990124204883325 | 276.717217800084 | 333.933092653605 | 277.767562028899 | 281.266150260984 | 311.65472770475 | 288.499545240955 |
| YDR133C | 22197.6557336754 | -0.707729171951799 | 0.37271593930161 | -1.89884332094284 | 0.0575850793947538 | 0.271149598582117 | 9133.02132783211 | 20711.2450971284 | 20736.2243491214 | 25420.8554182372 | 28607.6778980981 | 28576.9103116353 |
| YDR135C | 3726.80100833725 | 0.338504474936581 | 0.290469904372165 | 1.16536849374547 | 0.243869864804628 | 0.567946943988665 | 5311.7527553752 | 3408.33555084823 | 3764.87655020251 | 3322.26179699413 | 3228.965589541 | 3324.61380706243 |
| YDR136C | 14.5652480719571 | -0.549351304044251 | 0.614653570035651 | -0.893757607252468 | 0.371451595597649 | 0.683833965025226 | 7.44227236137145 | 17.251156078046 | 11.2608471092797 | 18.6818845191797 | 15.5827363852375 | 17.1725919786283 |
| YDR137W | 195.832621754688 | 0.154514882823459 | 0.351389355216856 | 0.43972556518709 | 0.660135884157139 | 0.866117567252964 | 286.189200805466 | 165.118208175583 | 166.410296170467 | 203.424964764402 | 176.97536323234 | 176.877697379871 |
| YDR138W | 106.487767012896 | -0.120919001866363 | 0.37714014298476 | -0.320620872944967 | 0.748497717737658 | 0.908246755658868 | 126.518630143315 | 85.0235549560838 | 93.8403925773309 | 147.379311206862 | 85.7050501188062 | 100.459663074975 |
| YDR139C | 1324.73398458141 | -0.90550079359133 | 0.25782545186925 | -3.51206906465748 | 0.000444632436256312 | 0.0154126817810676 | 857.891032201727 | 906.917919531561 | 1002.21539272589 | 1873.37786428441 | 1553.82142812797 | 1754.18027061688 |
| YDR140W | 1168.39681343295 | 0.0152277409380887 | 0.270644486955094 | 0.0562647372182215 | 0.955130909938185 | 0.986770496029859 | 969.525117622299 | 1212.5098271998 | 1342.54321647301 | 1147.89801545627 | 1197.6445964654 | 1140.26010738092 |
| YDR141C | 1663.68493981594 | 0.219866216550806 | 0.273996805744418 | 0.802440802013932 | 0.422298018893164 | 0.722127029121936 | 1488.45447227429 | 1930.897255307 | 1951.88016560848 | 1666.83925210015 | 1580.53469050266 | 1363.50380310308 |
| YDR142C | 945.584218869992 | 0.25636909574189 | 0.288830200190436 | 0.88761180642764 | 0.37474960064473 | 0.687096411016019 | 844.359627908324 | 1080.66170574617 | 1163.6208679589 | 803.321034324729 | 1006.19954944676 | 775.342527835066 |
| YDR143C | 503.728881306452 | 0.500973935736515 | 0.593044856037691 | 0.844748808856839 | 0.398251101652106 | 0.704186186941373 | 1286.83654830259 | 237.819508790205 | 245.236225935425 | 426.569696521271 | 394.020620026719 | 431.890688262501 |
| YDR144C | 4965.1335081816 | 0.583541388782677 | 0.26849369885984 | 2.17338951066893 | 0.0297510136311015 | 0.193279597385414 | 4850.33186897017 | 6316.38757543313 | 6701.45523525579 | 4148.41624573119 | 3952.44977885559 | 3821.76034484372 |
| YDR145W | 126.212104097364 | -0.0566589679908759 | 0.327745151038444 | -0.172875076294355 | 0.862749622902363 | 0.953454774053698 | 104.86838327387 | 145.402601229245 | 121.366907733348 | 147.379311206862 | 125.774943680845 | 112.480477460015 |
| YDR146C | 22.8916064146004 | 0.3121448867773 | 0.562383407640001 | 0.555039289098503 | 0.578867787168857 | 0.818699339992963 | 37.2113618068572 | 25.876734117069 | 12.5120523436441 | 20.7576494657553 | 17.8088415831286 | 23.1829991711481 |
| YDR147W | 261.62554131405 | -0.01989286712092 | 0.284785821540115 | -0.0698520277917624 | 0.944311436084314 | 0.984861822652717 | 230.033872987845 | 283.411849853613 | 266.50671491962 | 292.682857467149 | 251.549887361691 | 245.568065294384 |
| YDR148C | 2963.68220605369 | -0.10609113748782 | 0.319625180187295 | -0.331923590706003 | 0.73994695649654 | 0.90301928216742 | 3929.51980680413 | 2273.45592599963 | 2359.77307201128 | 3084.58671061123 | 3132.13001343273 | 3002.62770746315 |
| YDR150W | 75.6878568980074 | -0.138940949280848 | 0.535199526136966 | -0.259605889944848 | 0.79516779255235 | 0.929585587537483 | 136.667183363367 | 39.4312138926766 | 38.7873622652968 | 79.9169504431578 | 73.4614715304053 | 85.8629598931413 |
| YDR151C | 350.30288499943 | 0.409790290124718 | 0.401858308438992 | 1.01973825480065 | 0.307852613616596 | 0.632655281754807 | 636.652572004594 | 280.94739898532 | 280.269972497628 | 280.228267787696 | 328.350516688933 | 295.368582032406 |
| YDR152W | 97.1309748156867 | 0.778030716859161 | 0.505786002148905 | 1.53826067458092 | 0.123984890927066 | 0.406181701839891 | 221.915030411803 | 65.3079480097455 | 80.0771349993223 | 68.5002432369924 | 64.557050738841 | 82.4284414974156 |
| YDR153C | 119.179637756248 | -1.0436656426176 | 0.375677344820643 | -2.77809044651301 | 0.00546793955779556 | 0.0755522033130021 | 104.1918130592 | 55.4501445365764 | 72.5699035931359 | 143.227781313711 | 193.671152216523 | 145.96703181834 |
| YDR154C | 5133.54819473472 | 0.138019964330822 | 0.4271321201926 | 0.323131784770921 | 0.746595439594213 | 0.906988469670736 | 2300.33872987845 | 6954.68035032083 | 6882.87999423863 | 4950.69939758263 | 4965.32764389603 | 4747.36305249178 |
| YDR155C | 25717.0152043021 | 0.253900292919995 | 0.408460562661199 | 0.621602955413335 | 0.534202977204835 | 0.796317093613431 | 13077.425679359 | 35879.9401914674 | 34966.1814795478 | 22002.0705512273 | 25631.3752485178 | 22745.0980756931 |
| YDR156W | 2941.94518254418 | -0.421920473606944 | 0.285146749226327 | -1.47966082289809 | 0.138963785786908 | 0.428521373767782 | 2161.64183587107 | 2364.64060812645 | 3019.15823052132 | 3122.98836212288 | 3076.47738348546 | 3906.76467513793 |
| YDR157W | 192.605322151313 | -0.00165765958317673 | 0.33891534700212 | -0.00489107264642802 | 0.996097504209292 | 0.998355441010463 | 132.607762075346 | 213.175000107283 | 232.724173591781 | 200.311317344538 | 194.784204815469 | 182.029474973459 |
| YDR158W | 32776.7709045529 | 0.222824652484492 | 0.320486714129767 | 0.695269546787731 | 0.486886395203846 | 0.766813881410433 | 23394.4448828638 | 39248.8445284229 | 43266.6770043213 | 30856.2459308452 | 30301.7439536932 | 29592.6691271711 |
| YDR159W | 102.704216994313 | -0.210725692842125 | 0.407704564839751 | -0.516858801728037 | 0.605254728703029 | 0.832696070029921 | 144.109455724738 | 75.1657514829147 | 65.0626721869494 | 116.24283700823 | 105.739996899826 | 109.904588663221 |
| YDR160W | 748.212014500766 | 0.850011239336143 | 0.291916583147177 | 2.91182922933703 | 0.00359319020460838 | 0.0570807968717871 | 740.844385063794 | 1127.48627224372 | 1019.732266007 | 558.380770628817 | 535.378300092802 | 507.450092968465 |
| YDR161W | 718.29207562256 | 1.1725732393927 | 0.464722254244616 | 2.5231699766534 | 0.0116302169584198 | 0.113114191331636 | 1782.7625156558 | 603.79046273161 | 598.076102026189 | 462.895583086343 | 365.081252454135 | 497.146537781288 |
| YDR162C | 111.612893276498 | 0.124031189227948 | 0.389494580661562 | 0.318441373477596 | 0.750150158836764 | 0.908603445448746 | 167.112843023523 | 101.042485599984 | 80.0771349993223 | 105.864012275352 | 97.948628707207 | 117.632255053604 |
| YDR163W | 19.621641889564 | -0.146696586660658 | 0.518484827704663 | -0.282933229329168 | 0.77722802154229 | 0.920342502126552 | 17.5908255814234 | 20.9478323804844 | 17.5168732811018 | 21.795531939043 | 16.695788984183 | 23.1829991711481 |
| YDR164C | 265.417846887946 | 0.714601533122384 | 0.713981042302051 | 1.00086905783707 | NA | NA | 809.854546960148 | 85.0235549560838 | 93.8403925773309 | 218.993201863718 | 165.844837242885 | 218.95054772751 |
| YDR165W | 915.44718004397 | 0.285280959920235 | 0.368197048168057 | 0.77480512497217 | 0.43845482046894 | 0.736481095282136 | 1418.0911699486 | 969.761416673014 | 628.105027650935 | 952.776110478167 | 705.675347731469 | 818.274007781636 |
| YDR166C | 355.561774920578 | 0.254034015752706 | 0.271076967644797 | 0.937128734911839 | 0.34869234211414 | 0.661232771005165 | 364.671345707201 | 410.331069570665 | 385.371212184239 | 327.970861558933 | 353.95072646468 | 291.075434037749 |
| YDR167W | 854.855254779095 | -0.658383771331333 | 0.343744654382008 | -1.91532802892598 | 0.0554506832276419 | 0.267167248275344 | 909.310368516657 | 476.871243014557 | 601.829717729282 | 1075.24624232612 | 871.520184974354 | 1194.35377211359 |
| YDR168W | 264.874077425596 | -0.780615304907642 | 0.339808384380946 | -2.2972220250826 | 0.021606107302007 | 0.161095469079792 | 260.479532648001 | 151.563728399976 | 171.415117107924 | 336.273921345235 | 288.280623126893 | 381.231541925547 |
| YDR169C | 48.9867538351319 | -0.505860806337471 | 0.489048748073576 | -1.03437706022175 | 0.300959924308215 | 0.625627510682729 | 65.6273108230028 | 32.0378612877997 | 22.5216942185594 | 53.9698886109637 | 65.6701033377865 | 54.093664732679 |
| YDR169C-A | 1.64753694283368 | 1.14274917107152 | 1.61520182435969 | 0.707496211208491 | 0.479258166371466 | NA | 0.676570214670132 | 3.69667630243843 | 2.50241046872882 | 1.03788247328776 | 1.11305259894553 | 0.858629598931413 |
| YDR170C | 2201.02295560459 | 0.51809796299769 | 0.272692398968189 | 1.89993547659585 | 0.0574415876625252 | 0.271149598582117 | 3075.01162567575 | 2554.40332498495 | 2145.81697693497 | 1815.2564457803 | 1855.45868244221 | 1760.1906778094 |
| YDR170W-A | 7.58734476378546 | 1.58304247652513 | 1.08009420162759 | 1.46565223120321 | 0.142743054066207 | NA | 27.7393788014754 | 4.92890173658457 | 1.25120523436441 | 3.11364741986329 | 3.3391577968366 | 5.15177759358848 |
| YDR171W | 169.191527160787 | 0.231306674494474 | 0.549615810625916 | 0.420851565807498 | 0.673863474600575 | 0.871790963764147 | 359.25878398984 | 70.2368497463301 | 117.613292030255 | 149.455076153438 | 139.131574868192 | 179.453586176665 |
| YDR172W | 1427.50194683807 | 0.31156229405095 | 0.46011419218632 | 0.677141238722725 | 0.498316346074289 | 0.774350461539609 | 2808.44296109572 | 1028.90823751203 | 904.621384445469 | 1403.21710388506 | 1015.10397023833 | 1404.71802385179 |
| YDR173C | 170.9561635167 | 0.00370986478632944 | 0.361843754210176 | 0.0102526705053325 | 0.991819695812231 | 0.998070660281636 | 115.016936493922 | 223.032803580452 | 176.419938045382 | 197.197669924675 | 156.94041645132 | 157.129216604448 |
| YDR174W | 2065.3809677296 | 0.192849345344002 | 0.679701628239237 | 0.283726472516444 | NA | NA | 5270.48197228033 | 690.04624312184 | 648.124311400765 | 1718.73337576454 | 1578.30858530477 | 2486.59131850537 |
| YDR175C | 496.502710079417 | -0.557287803733407 | 0.316109892524031 | -1.76295591157762 | 0.0779079258662905 | 0.31959809218616 | 518.252784437321 | 310.520809404828 | 375.361570309323 | 609.237011819917 | 524.247774103347 | 641.396310401765 |
| YDR176W | 5.9346220392553 | -0.440975987003665 | 0.987271525393616 | -0.446661304070177 | 0.655119625248237 | NA | 10.8251234347221 | 2.46445086829228 | 1.25120523436441 | 7.26517731301435 | 7.79136819261874 | 6.01040719251989 |
| YDR177W | 751.57901108249 | -0.322290793050189 | 0.633089407355315 | -0.509076268384484 | 0.610698765530252 | 0.836562606639673 | 1478.98248926891 | 195.923844029237 | 327.815771403476 | 920.601753806246 | 542.056615686475 | 1044.0935923006 |
| YDR178W | 3177.76740140472 | -0.0514688345273011 | 0.383102092157666 | -0.13434756839208 | 0.893127740017738 | 0.965832153362604 | 1673.83471109391 | 3773.07427935549 | 3917.52358879497 | 2860.40409638108 | 3761.00473183696 | 3080.76300096591 |
| YDR179W-A | 779.287437967595 | -0.51552251655194 | 0.325624571036402 | -1.5831806393207 | 0.113380297943408 | 0.387922590820661 | 445.183201252947 | 777.534248946216 | 703.177341712799 | 1050.33706296722 | 812.52839723024 | 886.964375696149 |
| YDR180W | 347.721669149598 | 0.318016173672216 | 0.312135381586989 | 1.01884051739129 | 0.308278686675228 | 0.63307820329018 | 299.044034884198 | 421.421098477981 | 437.921832027544 | 364.296748124005 | 287.167570527948 | 276.478730855915 |
| YDR181C | 70.4095898301843 | -0.69059078154539 | 0.380285795820793 | -1.81597837503988 | 0.0693736894216535 | 0.30095091833635 | 53.4490469589404 | 38.1989884585304 | 70.067493124407 | 88.2200102294599 | 82.3658923219696 | 90.1561078877983 |
| YDR182W | 1335.28996933103 | -0.271079957839461 | 0.320857821835892 | -0.844860057605545 | 0.398188977796998 | 0.704186186941373 | 1657.59702594182 | 1005.49595426325 | 965.930440929326 | 1422.93687087752 | 1540.46479694062 | 1419.31472703362 |
| YDR182W-A | 55.2825547049161 | -0.863339934843524 | 0.370289123332814 | -2.33152928466537 | 0.0197254677439536 | 0.154261208969041 | 40.5942128802079 | 43.127890195115 | 33.7825413278391 | 70.5760081835679 | 72.3484189314597 | 71.2662567113072 |
| YDR183C-A | 74.6000258764408 | -0.539499875147429 | 0.420405953457703 | -1.28328314742029 | 0.199392894708275 | 0.515239459624559 | 41.9473533095482 | 77.630202351207 | 63.811466952585 | 107.939777221927 | 60.1048403430589 | 96.1665150803182 |
| YDR183W | 89.4082346236409 | 0.128873377430599 | 0.355552701424873 | 0.362459283572143 | 0.717008844876204 | 0.893811936748736 | 120.429498211283 | 80.0946532194992 | 78.8259297649579 | 84.0684803363089 | 84.5919975198606 | 88.4388486899355 |
| YDR184C | 73.024324695047 | 0.54399784068367 | 0.488826641871109 | 1.11286454969267 | 0.265766607319367 | 0.592894614644195 | 136.667183363367 | 81.3268786536454 | 41.2897727340256 | 56.0456535575392 | 48.9743143536035 | 73.8421455081015 |
| YDR185C | 503.184947494174 | 0.0519240784840288 | 0.359306363166131 | 0.144511992569474 | 0.885096187151986 | 0.96227215505005 | 322.723992397653 | 639.525000321848 | 575.554407807629 | 399.584752215789 | 566.543772863277 | 515.177759358848 |
| YDR186C | 29.9276850666647 | 0.494691569130894 | 0.56014121548057 | 0.883155096356329 | 0.377152479117091 | 0.688869542410245 | 59.5381788909716 | 19.7156069463383 | 25.0241046872882 | 30.0985917253451 | 21.1479993799652 | 24.0416287700796 |
| YDR188W | 6594.3429974828 | 0.442027342725664 | 0.35435443808625 | 1.24741585039235 | 0.212245057672401 | 0.531943568048539 | 10474.660063523 | 4693.54667866266 | 7621.09108251363 | 5925.27103999984 | 5056.59795700956 | 5794.8911631881 |
| YDR189W | 226.079078654773 | 0.299055235158196 | 0.729098313952332 | 0.410171343747955 | NA | NA | 615.67889534982 | 59.1468208390148 | 72.5699035931359 | 217.95531939043 | 144.69683786292 | 246.426694893315 |
| YDR190C | 1290.77212151292 | 0.0480074203779213 | 0.495779846855132 | 0.0968321336223034 | 0.922859704894795 | 0.97858007507751 | 2466.7750026873 | 745.496387658416 | 723.19662546263 | 1423.97475335081 | 965.016603285779 | 1420.17335663256 |
| YDR191W | 191.420778216482 | -0.131386365538854 | 0.295589970969888 | -0.444488576888281 | 0.656689385842892 | 0.864280886779837 | 165.759702594182 | 172.51156078046 | 210.202479373221 | 214.841671970567 | 201.462520409142 | 183.746734171322 |
| YDR192C | 172.772341698554 | 0.285489537801758 | 0.785411428367839 | 0.363490429971248 | 0.716238560546 | 0.893196615263775 | 487.807124777165 | 33.2700867219458 | 47.5457989058476 | 157.75813593974 | 110.192207295608 | 200.060696551019 |
| YDR193W | 3.34527925557768 | 1.24380145131795 | 1.28535887683083 | 0.967668620599295 | 0.333209894489266 | NA | 0.676570214670132 | 8.625578039023 | 5.00482093745765 | 2.07576494657553 | 1.11305259894553 | 2.57588879679424 |
| YDR194C | 425.415611653472 | -0.0917173026601959 | 0.542339475103899 | -0.169114192992544 | 0.865706821330644 | 0.954552620181958 | 805.795125672127 | 274.78627181459 | 153.898243826823 | 447.327345987026 | 349.498516068898 | 521.188166551367 |
| YDR195W | 37.405516674288 | -0.284812455590578 | 0.533894997123308 | -0.533461555409177 | 0.59371411674517 | 0.827932882774509 | 56.8318980322911 | 23.4122832487767 | 20.0192837498306 | 38.4016515116473 | 33.391577968366 | 52.3764055348162 |
| YDR196C | 365.000938406136 | 0.124205952574788 | 0.269372599515882 | 0.461093492055287 | 0.644731530661694 | 0.858865749623948 | 336.931966905726 | 406.634393268227 | 399.134469762247 | 363.258865650717 | 340.594095277334 | 343.451839572565 |
| YDR197W | 608.981053667504 | -0.281349651224448 | 0.25488153149081 | -1.1038447924368 | 0.269660470635797 | 0.595604728404763 | 556.817286673518 | 569.288150575518 | 523.003787964324 | 674.623607637046 | 706.788400330415 | 623.365088824205 |
| YDR198C | 310.758289790583 | 0.231567461541407 | 0.267476981006944 | 0.865747252977241 | 0.386628780787569 | 0.697440084467597 | 326.783413685674 | 346.255346995066 | 334.071797575298 | 307.213212093178 | 266.019571147983 | 284.206397246298 |
| YDR199W | 1.81520535994891 | -2.22187590303178 | 1.91805927864622 | -1.15839793262281 | 0.246701682447953 | NA | 0.676570214670132 | 1.23222543414614 | 0 | 7.26517731301435 | 0 | 1.71725919786283 |
| YDR200C | 9.13218679012874 | -0.462492382731637 | 0.755255050664366 | -0.612365825723113 | 0.540295746911817 | NA | 11.5016936493922 | 3.69667630243843 | 7.50723140618647 | 11.4167072061654 | 7.79136819261874 | 12.8794439839712 |
| YDR201W | 228.390636059318 | 0.27892398187325 | 0.407962678370672 | 0.683699751622431 | 0.494164780354864 | 0.772703796920499 | 129.901481216665 | 301.895231365805 | 320.308539997289 | 229.372026596596 | 213.706098997543 | 175.160438182008 |
| YDR202C | 87.3675772611239 | 1.27137014403501 | 0.745694926504096 | 1.70494675348717 | 0.0882043555477561 | 0.340724889575606 | 303.103456172219 | 36.9667630243843 | 30.0289256247459 | 60.1971834506903 | 38.9568409630937 | 54.9522943316104 |
| YDR203W | 2.66661432189777 | 1.59005000791634 | 1.46087455687191 | 1.08842336971152 | 0.276408248943027 | NA | 3.38285107335066 | 7.39335260487685 | 1.25120523436441 | 3.11364741986329 | 0 | 0.858629598931413 |
| YDR204W | 1311.69804944889 | 0.223329813798869 | 0.282273119133399 | 0.791183427187501 | 0.42883696103623 | 0.728414554384234 | 1192.79328846344 | 1455.25823772659 | 1591.53305811153 | 1070.05682995968 | 1409.12459026505 | 1151.42229216702 |
| YDR205W | 1019.33351545197 | 0.0853106141959669 | 0.308229399693905 | 0.27677636942059 | 0.78195182706571 | 0.923117498865004 | 750.316368069176 | 1185.40086764859 | 1213.66907733348 | 1010.89752898228 | 1050.72165340458 | 904.995597273709 |
| YDR206W | 1293.81918878833 | -0.153375205123966 | 0.383854365336658 | -0.399566134904963 | 0.689476103618882 | 0.879721793562251 | 1880.1886265683 | 845.306647824254 | 948.413567648224 | 1562.01312229808 | 1130.86144052866 | 1396.13172786248 |
| YDR207C | 9.1524888100074 | -0.894538378433005 | 0.874172655932974 | -1.02329713971469 | 0.306167375723289 | NA | 13.5314042934026 | 3.69667630243843 | 1.25120523436441 | 9.34094225958987 | 13.3566311873464 | 13.7380735829026 |
| YDR208W | 558.553447944149 | -0.131635279396321 | 0.267535643000192 | -0.492028942088387 | 0.622698883650861 | 0.845963881641491 | 580.497244186973 | 503.980202565772 | 514.245351323773 | 656.979605591154 | 540.94356308753 | 554.674720909692 |
| YDR209C | 2.19166910124412 | -0.289424935486441 | 1.52008180448054 | -0.190400894631684 | 0.848994996681743 | NA | 3.38285107335066 | 2.46445086829228 | 0 | 1.03788247328776 | 1.11305259894553 | 5.15177759358848 |
| YDR210W | 1024.70282837385 | -0.634930310142951 | 0.251436146052606 | -2.52521493075267 | 0.0115627548263202 | 0.113031827791987 | 800.382563954766 | 805.875433931577 | 802.022555227588 | 1366.89121731998 | 1219.90564844431 | 1153.13955136489 |
| YDR211W | 1062.12381864372 | -0.200594521690029 | 0.424277991009953 | -0.472790307158127 | 0.636362790020388 | 0.853279471134959 | 1603.47140876821 | 703.600722897447 | 656.882748041316 | 1226.77708342614 | 823.658923219696 | 1358.35202550949 |
| YDR212W | 11718.7070828329 | 0.729718491732009 | 0.314805712451319 | 2.3179963478104 | 0.0204495195471568 | 0.156769082749603 | 9975.35124509642 | 16152.0109907876 | 17735.8341971155 | 9364.81355647549 | 8696.27995556146 | 8387.95255196097 |
| YDR213W | 531.053527586524 | 0.777019706495439 | 0.283990217408789 | 2.73607912830659 | 0.00621760730657635 | 0.0806694463415985 | 573.731542040272 | 741.799711355978 | 696.921315540977 | 346.652746078113 | 448.56019737505 | 378.655653128753 |
| YDR214W | 4869.27946788494 | -0.39076164065734 | 0.269175161576195 | -1.45170021769162 | 0.14658497876912 | 0.440892991564844 | 4901.07463507043 | 3515.53916361894 | 4224.06887121425 | 5350.28414979842 | 5307.03479177231 | 5917.6751958353 |
| YDR215C | 14.6580752676682 | -1.26677449120981 | 0.691379815156822 | -1.83224106842412 | 0.0669155127351983 | 0.296163620788428 | 4.05942128802079 | 13.5544797756076 | 8.75843664055088 | 13.4924721527409 | 30.0524201715294 | 18.0312215775597 |
| YDR216W | 403.32594785148 | -0.298607381656707 | 0.314346567198921 | -0.949930467883067 | 0.342147584311893 | 0.655118026190794 | 290.248622093487 | 420.188873043835 | 375.361570309323 | 405.812047055516 | 545.395773483312 | 382.94880112341 |
| YDR217C | 167.198795026544 | 0.0981617681351175 | 0.329886486729279 | 0.297562258788957 | 0.76603728346402 | 0.91618441642763 | 127.195200357985 | 187.298265990214 | 205.197658435763 | 166.061195726042 | 149.149048258702 | 168.291401390557 |
| YDR218C | 2.56989459966933 | -1.23402114315348 | 1.33898278065901 | -0.921610913133722 | 0.35673156402049 | NA | 2.0297106440104 | 2.46445086829228 | 0 | 4.15152989315105 | 3.3391577968366 | 3.43451839572565 |
| YDR219C | 27.3432503689777 | -0.294607151047341 | 0.616129827336992 | -0.478157586235808 | 0.632538047798374 | 0.851681587899989 | 45.3302043828988 | 13.5544797756076 | 13.7632575780085 | 41.5152989315106 | 26.7132623746928 | 23.1829991711481 |
| YDR221W | 320.238822458427 | 0.536635527065863 | 0.504707012518934 | 1.06326148390049 | 0.287663386747197 | 0.611271403705319 | 725.283270126381 | 207.013872936552 | 203.946453201399 | 269.849443054819 | 244.871571768018 | 270.468323663395 |
| YDR222W | 1171.58130322363 | 0.640733006549401 | 0.319059273248264 | 2.00819427696382 | 0.04462265093795 | 0.23705267799569 | 1004.03019857048 | 1548.9073707217 | 1730.41683912598 | 1014.01117640215 | 932.738077916358 | 799.384156605145 |
| YDR223W | 29.1818264611841 | 0.100529066774804 | 0.467781353458783 | 0.214906100962535 | 0.829840528146113 | 0.939516122864656 | 29.7690894454858 | 22.1800578146306 | 38.7873622652968 | 26.9849443054819 | 25.6002097757473 | 31.7692951604623 |
| YDR224C | 11750.2637847436 | -0.43646909779688 | 0.254777269087621 | -1.71313987060115 | 0.086686810507539 | 0.337585221407408 | 11271.6597764044 | 10029.0828085155 | 8657.08901656736 | 13708.3517071848 | 13416.7360276895 | 13418.6633721001 |
| YDR225W | 51382.646261034 | -0.251808817787021 | 0.362853848799616 | -0.693967608777057 | 0.487702522495352 | 0.766980601724564 | 26252.9540398451 | 56288.0578317958 | 58189.8018345857 | 54517.8905568597 | 57235.3907429773 | 55811.7825601407 |
| YDR226W | 15105.9831047003 | -0.156326741209799 | 0.251852214654389 | -0.62070822535479 | 0.534791616477274 | 0.796624869342804 | 15002.2679400955 | 12704.2442260467 | 15158.3514143248 | 14650.7489929301 | 16232.7591030217 | 16887.526951783 |
| YDR227W | 81.6056002663697 | -0.0477059794413952 | 0.399563715320311 | -0.119395174316946 | 0.904962284286332 | 0.971014226260563 | 95.3964002684886 | 97.3458092975452 | 47.5457989058476 | 89.2578927027477 | 76.8006293272419 | 83.287071096347 |
| YDR228C | 104.512684228563 | -0.697853456213306 | 0.370734277982047 | -1.88235482300641 | 0.0597878554904105 | 0.278267686930267 | 57.5084682469612 | 85.0235549560838 | 97.5940082804241 | 157.75813593974 | 112.418312493499 | 116.773625454672 |
| YDR229W | 14.9031898795881 | 0.293168502680986 | 0.713135021705069 | 0.411098170413837 | 0.681000551494832 | 0.87491979660087 | 27.7393788014754 | 9.85780347316914 | 11.2608471092797 | 18.6818845191797 | 5.56526299472767 | 16.3139623796968 |
| YDR230W | 1.42406080070982 | 2.75224279033418 | 2.23325253011445 | 1.2323921066791 | 0.217802648037803 | NA | 0 | 4.92890173658457 | 2.50241046872882 | 0 | 1.11305259894553 | 0 |
| YDR231C | 452.715783733796 | -0.638219611638793 | 0.289869230657643 | -2.20175011397666 | 0.0276829646633966 | 0.184842135868471 | 329.489694544354 | 304.359682234097 | 429.163395386993 | 497.145704704839 | 576.561246253787 | 579.574979278703 |
| YDR232W | 8364.61408093326 | 0.645760385496693 | 0.33739414921596 | 1.91396438556305 | 0.0556247065182867 | 0.267167980202198 | 6352.99431575254 | 11688.8904683103 | 12577.1150158311 | 6249.09037166562 | 6786.28169577092 | 6533.31261826912 |
| YDR233C | 29255.838850862 | -0.0552891432211846 | 0.284844928635919 | -0.194102606937593 | 0.846095540407851 | 0.945953694112353 | 21751.7324016447 | 31707.6248714485 | 32627.6788965208 | 29271.3993941348 | 31344.6742389052 | 28831.9233025179 |
| YDR234W | 7470.05093555817 | 0.578993483788074 | 0.278217216926558 | 2.08108430593967 | 0.0374261900833546 | 0.218623720121059 | 7268.39381620123 | 8869.55867498393 | 10710.3168061594 | 6297.87084791015 | 6135.14592538779 | 5539.01954270654 |
| YDR235W | 512.904527998937 | 0.272314531796986 | 0.309859597614337 | 0.878832006152409 | 0.379492369599485 | 0.690456306244106 | 421.503243739492 | 637.060549453556 | 625.602617182206 | 489.880527391824 | 507.551985119164 | 395.828245107381 |
| YDR236C | 1243.63069964493 | -0.0575667328257546 | 0.370643371853302 | -0.155315694809023 | 0.87657244308827 | 0.95870019275626 | 676.570214670132 | 1410.89812209733 | 1570.26256912734 | 1212.24672880011 | 1346.7936447241 | 1245.01291845055 |
| YDR237W | 1341.11092331653 | -1.39082087801062 | 0.501552222702931 | -2.77303302638218 | 0.00555364840818144 | 0.0763693087326003 | 1414.70831887525 | 391.847688058473 | 412.897727340256 | 1914.89316321592 | 1609.47405807524 | 2302.84458433405 |
| YDR238C | 5156.06302604721 | 0.205153799160236 | 0.335149663271282 | 0.612125929525931 | 0.540454443234627 | 0.798710611079734 | 7837.38936673881 | 4698.47558039924 | 4028.8808546534 | 4791.9033791696 | 4860.70069959515 | 4719.02827572704 |
| YDR239C | 119.86609590896 | 0.420089456954138 | 0.340575045564251 | 1.23347104382868 | 0.217400073505659 | 0.537931822001948 | 172.525404740884 | 118.29364167803 | 120.115702498983 | 90.2957751760354 | 104.62694430088 | 113.339107058946 |
| YDR240C | 202.717823001307 | -0.0874030581435319 | 0.404651768583222 | -0.215995739866774 | 0.828991068284602 | 0.939195669606348 | 303.103456172219 | 165.118208175583 | 120.115702498983 | 229.372026596596 | 203.688625607033 | 194.908918957431 |
| YDR241W | 50.7260040365237 | -1.21078476754058 | 0.398604747393556 | -3.03755731826528 | 0.00238504041162437 | 0.0445104295000548 | 26.3862383721351 | 34.502312156092 | 31.2801308591103 | 70.5760081835679 | 82.3658923219696 | 59.2454423262675 |
| YDR242W | 527.792796019806 | -0.448793469386347 | 0.269977317191293 | -1.66233768842274 | 0.0964450850773894 | 0.356047751461037 | 449.919192755638 | 450.994508897488 | 437.921832027544 | 546.964063422651 | 705.675347731469 | 575.281831284046 |
| YDR243C | 111.962712822112 | 0.138525348301615 | 0.447797138724339 | 0.309348444468044 | 0.757056483532723 | 0.912468162580435 | 190.792800536977 | 91.1846821268145 | 68.8162878900426 | 124.545896794532 | 96.8355761082615 | 99.6010334760439 |
| YDR244W | 99.6214083047537 | -0.181498261161884 | 0.383895140978199 | -0.472780824209992 | 0.636369556228338 | 0.853279471134959 | 128.548340787325 | 61.6112717073071 | 88.8355716398732 | 103.788247328776 | 110.192207295608 | 104.752811069632 |
| YDR245W | 1996.98499835835 | 0.442881988242008 | 0.295074699963385 | 1.50091481342509 | 0.13337759621252 | 0.41870804097737 | 2949.84613596177 | 1950.61286225334 | 2001.92837498306 | 1767.51385200906 | 1563.83890151848 | 1748.16986342436 |
| YDR246W | 38.4222425722492 | 0.869425726880424 | 0.718833750392563 | 1.20949486081534 | 0.226472784292913 | 0.549500027064442 | 110.957515205902 | 12.3222543414614 | 25.0241046872882 | 20.7576494657553 | 32.2785253694205 | 29.193406363668 |
| YDR246W-A | 21.3023821520119 | -0.21583287695312 | 0.498026199453013 | -0.433376551655658 | 0.664741232567615 | 0.867605041961546 | 18.2673957960936 | 19.7156069463383 | 21.270488984195 | 25.9470618321941 | 21.1479993799652 | 21.4657399732853 |
| YDR247W | 835.985899230006 | 1.02001760210864 | 0.379288484555843 | 2.68929230293694 | 0.00716036897574219 | 0.0864679464259746 | 835.564215117613 | 1296.30115672174 | 1227.43233491149 | 434.872756307573 | 814.754502428131 | 406.99042989349 |
| YDR248C | 80.2330609103689 | -0.36260499781397 | 0.524781911208231 | -0.690963217423266 | 0.489588656587541 | 0.768398138949443 | 125.165489713974 | 46.8245664975534 | 37.5361570309323 | 88.2200102294599 | 63.4439981398955 | 120.208143850398 |
| YDR249C | 775.754335524873 | 0.350789386082135 | 0.368571211088647 | 0.951754709886348 | 0.341221395922049 | 0.653998193984641 | 502.015099285238 | 1091.75173465348 | 1015.9786503039 | 632.070426232248 | 782.475977058711 | 630.234125615657 |
| YDR250C | 9.87299965617479 | 1.4161160216029 | 0.808345139920718 | 1.75187052122537 | 0.0797960745020694 | NA | 6.08913193203119 | 20.9478323804844 | 16.2656680467373 | 8.30305978630211 | 3.3391577968366 | 4.29314799465706 |
| YDR251W | 523.470449062142 | 0.481591731319035 | 0.28811493746332 | 1.67152642469412 | 0.0946177437575932 | 0.353762749548089 | 583.203525045654 | 503.980202565772 | 743.21590921246 | 426.569696521271 | 429.638303192976 | 454.215057834717 |
| YDR252W | 92.8384096325284 | 0.320050566337672 | 0.34073965922776 | 0.939281817276636 | 0.347586071526289 | 0.660034601629703 | 111.634085420572 | 113.364739941445 | 83.8307507024156 | 89.2578927027477 | 86.8181027177517 | 72.1248863102387 |
| YDR253C | 4.31273629413457 | -0.0918212997180985 | 1.0120147101266 | -0.0907311907616558 | 0.927706186195203 | NA | 2.70628085868053 | 6.16112717073071 | 3.75361570309323 | 6.22729483972658 | 4.45221039578214 | 2.57588879679424 |
| YDR254W | 108.639766785404 | -0.0626505551843188 | 0.376129812904131 | -0.166566310446355 | 0.867711303684134 | 0.955296796318024 | 138.020323792707 | 93.6491329951068 | 86.3331611711444 | 141.152016367136 | 87.9311553166972 | 104.752811069632 |
| YDR255C | 80.6347296745437 | 0.708870768747826 | 0.367966415804694 | 1.92645507388934 | 0.0540475763510161 | 0.264203320180409 | 81.864995975086 | 117.061416243884 | 101.347623983517 | 59.1593009774025 | 74.5745241293508 | 49.8005167380219 |
| YDR256C | 131.88763165474 | 0.00194424293186993 | 0.31345741690109 | 0.00620257434356203 | 0.99505109342639 | 0.998254726866075 | 110.957515205902 | 147.867052097537 | 137.632575780085 | 134.924721527409 | 138.018522269246 | 121.925403048261 |
| YDR257C | 100.533535046263 | 0.889207246152333 | 0.63270821104875 | 1.4053986191809 | 0.159902777126084 | 0.460250957897212 | 285.512630590796 | 64.0757225755994 | 41.2897727340256 | 72.6517731301435 | 70.1223137335687 | 69.5489975134444 |
| YDR258C | 561.748365405774 | 0.235483897763149 | 0.324818763703641 | 0.724970118961478 | 0.468470362112438 | 0.757246243369599 | 809.177976745478 | 478.103468448703 | 534.264635073604 | 528.282178903472 | 449.673249973996 | 570.988683289389 |
| YDR259C | 6.80647145652032 | -0.786816513765709 | 0.809875577795992 | -0.971527646144071 | 0.331285592994272 | NA | 6.08913193203119 | 3.69667630243843 | 5.00482093745765 | 8.30305978630211 | 10.0174733905098 | 7.72766639038271 |
| YDR260C | 455.612112948926 | 0.23001840899969 | 0.268464422247371 | 0.856792892980597 | 0.391559354137842 | 0.70049273812148 | 441.800350179596 | 503.980202565772 | 530.51101937051 | 423.456049101408 | 384.003146636209 | 449.92190984006 |
| YDR261C | 1926.61473632061 | -0.498518300366588 | 0.401443001086378 | -1.24181589669643 | 0.214304496140978 | 0.534463272773969 | 2602.08904562133 | 1052.32052076081 | 1134.84314756852 | 2163.98495680499 | 2358.55845716559 | 2247.89229000244 |
| YDR261C-D | 52.1670999782251 | -0.371622744839564 | 0.443641407620989 | -0.837664695981328 | 0.402219046497598 | 0.708308196207474 | 55.4787576029508 | 55.4501445365764 | 25.0241046872882 | 67.4623607637046 | 51.2004195514946 | 58.3868127273361 |
| YDR261W-B | 2.04627954776581 | 1.69246398587143 | 1.6443410536949 | 1.02926578526297 | 0.303354796510708 | NA | 6.76570214670132 | 0 | 2.50241046872882 | 1.03788247328776 | 1.11305259894553 | 0.858629598931413 |
| YDR262W | 1442.21078830671 | 0.585270520169171 | 0.380238293722051 | 1.53922035163822 | 0.123750510782715 | 0.406181701839891 | 952.610862255545 | 2097.24768891673 | 2143.31456646624 | 1098.07965673845 | 1355.69806551566 | 1006.31388994762 |
| YDR263C | 45.7040564565923 | 0.109665898768259 | 0.442389406081218 | 0.247894495801117 | 0.80421603873207 | 0.93085658289004 | 66.980451252343 | 41.8956647609688 | 32.5313360934747 | 41.5152989315106 | 50.0873669525491 | 41.2142207487078 |
| YDR264C | 2823.62993181675 | 0.343369163071223 | 0.308968367145524 | 1.11134083480299 | 0.266421668809757 | 0.593084549471051 | 2284.77761494104 | 3275.25520396045 | 3915.02117832624 | 2658.01701408996 | 2582.28202955364 | 2226.42655002915 |
| YDR265W | 532.307526026808 | 0.071183471104438 | 0.33475791644744 | 0.212641636260197 | 0.831606493010698 | 0.939849414436786 | 363.318205277861 | 633.363873151117 | 640.617079994579 | 494.032057284976 | 587.691772243242 | 474.822168209071 |
| YDR266C | 89.4338189967153 | 0.930075937239854 | 0.707508910134885 | 1.31457840871931 | 0.18865163046408 | 0.501558543898026 | 276.717217800084 | 38.1989884585304 | 36.2849517965679 | 79.9169504431578 | 44.5221039578214 | 60.9627015241303 |
| YDR267C | 1714.44312221518 | 1.17720560970822 | 0.351562138822627 | 3.34849939658079 | 0.000812504577439031 | 0.0223458196704285 | 1443.12426789139 | 2792.22283377516 | 2897.79132278798 | 1024.39000113502 | 1182.06186008016 | 947.068447621348 |
| YDR268W | 527.968046159342 | -0.602924726427471 | 0.257764334523569 | -2.33905411135279 | 0.019332633184471 | 0.153274449026675 | 415.414111807461 | 394.312138926766 | 447.931473902459 | 624.805248919234 | 672.283769763103 | 613.061533637029 |
| YDR269C | 1.59779932457419 | 0.462512809727965 | 1.67503969226374 | 0.276120507391022 | 0.78245551011878 | NA | 0.676570214670132 | 2.46445086829228 | 2.50241046872882 | 0 | 2.22610519789107 | 1.71725919786283 |
| YDR270W | 300.691264360963 | 0.375630923694177 | 0.309770229522365 | 1.21261143872141 | 0.225278413462099 | 0.548365637869472 | 268.598375224042 | 391.847688058473 | 359.095902262586 | 305.137447146603 | 238.193256174344 | 241.274917299727 |
| YDR271C | 8.38909497092983 | 0.892476234441486 | 0.839805683851859 | 1.06271754478732 | 0.28791006197677 | NA | 5.41256171736105 | 8.625578039023 | 18.7680785154662 | 5.18941236643882 | 8.90442079156428 | 3.43451839572565 |
| YDR272W | 274.390805653327 | 0.218904237340554 | 0.345612167186057 | 0.633381165723569 | 0.526484761271818 | 0.791959165822418 | 399.852996870048 | 211.942774673136 | 272.762741091442 | 266.735795634955 | 223.723572388052 | 271.326953262326 |
| YDR273W | 21.7976892385515 | -0.112266392659097 | 0.525964776349355 | -0.213448500179654 | 0.83097715433322 | 0.939639058736332 | 26.3862383721351 | 13.5544797756076 | 22.5216942185594 | 18.6818845191797 | 25.6002097757473 | 24.0416287700796 |
| YDR274C | 4.97564628568593 | -1.38418387020956 | 1.17450423732259 | -1.17852607612975 | 0.238586942974881 | NA | 0 | 2.46445086829228 | 6.25602617182206 | 4.15152989315105 | 6.67831559367321 | 10.303555187177 |
| YDR275W | 290.953917434628 | 0.546926265094768 | 0.355308054547758 | 1.53930162318134 | 0.123730677878522 | 0.406181701839891 | 485.777414133155 | 296.96632962922 | 252.743457341611 | 243.902381222624 | 207.027783403869 | 259.306138877287 |
| YDR276C | 10893.6049549085 | -0.217135866675371 | 0.396656796482098 | -0.547414965786854 | 0.584093675823039 | 0.821877710803141 | 5072.92346959665 | 12500.9270294126 | 12653.4385351273 | 11024.3876312626 | 13652.7031786659 | 10457.2498853857 |
| YDR277C | 31.4020873727587 | -1.03687079491092 | 0.624250740463435 | -1.6609844854027 | 0.0967165626869341 | 0.356820797384145 | 40.5942128802079 | 12.3222543414614 | 7.50723140618647 | 46.7047112979494 | 40.0698935620392 | 41.2142207487078 |
| YDR279W | 98.0753235528692 | 0.662315640491383 | 0.752028417084356 | 0.880705602933472 | NA | NA | 299.044034884198 | 30.8056358536536 | 30.0289256247459 | 72.6517731301435 | 62.3309455409499 | 93.590626283524 |
| YDR280W | 841.82517818449 | 0.518483618545401 | 0.264341792395394 | 1.96141372064948 | 0.0498307807293457 | 0.251252041782701 | 1111.60486270303 | 945.116907990091 | 917.133436789114 | 762.843617866506 | 652.248822982083 | 662.003420776119 |
| YDR281C | 228.436838273727 | 1.44024901197612 | 0.409742009960333 | 3.5150142698708 | 0.000439730359223624 | 0.0154126817810676 | 179.967677102255 | 401.705491531642 | 420.404958746442 | 151.530841100014 | 107.966102097717 | 109.045959064289 |
| YDR282C | 162.043994389189 | -0.070927926431713 | 0.296990382233681 | -0.238822300905033 | 0.811243376906633 | 0.932605386091865 | 148.168877012759 | 181.137138819483 | 145.139807186272 | 159.833900886316 | 171.410100237612 | 166.574142192694 |
| YDR283C | 678.675645600567 | 0.0763746658814716 | 0.301277122993991 | 0.253503037743078 | 0.799879514037966 | 0.929962020180481 | 900.514955725945 | 598.861560995025 | 589.317665385638 | 678.775137530197 | 640.005244393682 | 664.579309572913 |
| YDR284C | 3669.65283015867 | 0.203547201473887 | 0.346270690447229 | 0.58782682765034 | 0.556648534902472 | 0.80757591585548 | 2403.85397272298 | 4716.95896191143 | 4664.49311371053 | 3440.58039894894 | 3738.74367985805 | 3053.2868538001 |
| YDR285W | 6.49626363867977 | -0.897402669469341 | 0.909751167476416 | -0.986426510403578 | 0.323923861308397 | NA | 2.70628085868053 | 8.625578039023 | 2.50241046872882 | 12.4545896794532 | 6.67831559367321 | 6.01040719251989 |
| YDR286C | 238.189050659886 | -0.645763939134325 | 0.413319097088243 | -1.56238592333046 | 0.118197101224731 | 0.395918961445079 | 94.0432598391483 | 216.871676409721 | 247.738636404153 | 338.349686291811 | 258.228202955364 | 273.902842059121 |
| YDR287W | 824.636651467168 | 0.305334079356494 | 0.352734300775846 | 0.865620606459041 | 0.386698251426276 | 0.697440084467597 | 559.523567532199 | 1115.16401790226 | 1061.02203874102 | 664.244782904169 | 840.354712203879 | 707.510789519484 |
| YDR288W | 208.078241065863 | -0.0730298845979609 | 0.30095410477874 | -0.242661201287327 | 0.808267866051259 | 0.931015474312912 | 240.182426207897 | 179.904913385337 | 187.680785154662 | 216.917436917143 | 193.671152216523 | 230.112732513619 |
| YDR289C | 30.4411962997331 | -0.165236707891341 | 0.46186216690123 | -0.357761946599702 | 0.720521470385429 | 0.895808348138664 | 23.0033872987845 | 25.876734117069 | 37.5361570309323 | 34.2501216184962 | 34.5046305673116 | 27.4761471658052 |
| YDR291W | 525.149924861956 | 0.594598066604645 | 0.289019258924653 | 2.0572956584864 | 0.0396577947264934 | 0.22284687967803 | 782.115168158672 | 554.501445365764 | 558.037534526527 | 414.115106841818 | 427.412197995085 | 414.718096283872 |
| YDR292C | 1285.0652327685 | 0.649824487660607 | 0.244745180422374 | 2.65510637038555 | 0.00792834037436954 | 0.091694367146632 | 1663.68615787385 | 1489.76054988269 | 1555.24810631496 | 993.25352693639 | 1018.44312803516 | 989.999927567919 |
| YDR293C | 1961.621065708 | 0.399442964259192 | 0.510765088897146 | 0.782048289795368 | 0.434186194630536 | 0.733167522542986 | 4348.99333989961 | 1339.42904691686 | 1004.71780319462 | 1631.55124800836 | 1736.36205435503 | 1708.67290187351 |
| YDR294C | 4436.99103306518 | -0.108690548653745 | 0.324839064519681 | -0.334598145744751 | 0.737928243848866 | 0.90301928216742 | 5857.744918614 | 3159.42601315071 | 3791.15186012417 | 4314.47744145723 | 4936.38827632345 | 4562.75768872153 |
| YDR295C | 15.3252950540148 | -0.252334395639299 | 0.685012535200085 | -0.368364639583704 | 0.712601360126638 | 0.891399851461647 | 25.0330979427949 | 4.92890173658457 | 11.2608471092797 | 16.6061195726042 | 17.8088415831286 | 16.3139623796968 |
| YDR296W | 297.605992377713 | 0.492360776704714 | 0.56071105673288 | 0.878100709434151 | 0.379889068024026 | 0.690794800063937 | 718.51756797968 | 155.260404702414 | 168.912706639196 | 236.63720390961 | 220.384414591216 | 285.92365644416 |
| YDR297W | 7935.54245382496 | 0.54370676721072 | 0.345485928552316 | 1.57374504220477 | 0.115546404136519 | 0.391875784790183 | 5728.520007612 | 11228.0381559396 | 11284.6200087326 | 6199.27201294781 | 7105.72779166829 | 6067.07674604936 |
| YDR298C | 7489.55084805283 | 0.26643754122532 | 0.341127305023472 | 0.781050174822524 | 0.434772984922947 | 0.733511921730878 | 5031.65268650177 | 9293.4442243302 | 10213.5883281167 | 7158.27541826571 | 6664.95896248586 | 6575.38546861676 |
| YDR299W | 128.953294416857 | -0.669531575355918 | 0.491600392076678 | -1.36194272044333 | 0.173215962641444 | 0.481976601610467 | 170.495694096873 | 51.753468234138 | 75.0723140618647 | 201.349199817826 | 110.192207295608 | 164.856882994831 |
| YDR300C | 1167.08382589366 | 0.120121995419741 | 0.254047287025635 | 0.472833214737775 | 0.636332175278354 | 0.853279471134959 | 1269.24572272117 | 1133.64739941445 | 1243.69800295822 | 1231.96649579258 | 1072.9827053835 | 1050.96262909205 |
| YDR301W | 567.000082447338 | 0.567423470472928 | 0.266645891739471 | 2.12800379848842 | 0.0333367686435141 | 0.205619094534352 | 775.349466011971 | 624.738295112094 | 630.607438119663 | 456.668288246616 | 444.107986979268 | 470.529020214414 |
| YDR302W | 1942.45689110482 | -0.588119751127481 | 0.340386042619127 | -1.72780219365679 | 0.0840236939792843 | 0.332622722446919 | 974.93767933966 | 1860.66040556067 | 1821.75482123458 | 2507.52405546324 | 2081.40836002815 | 2408.45602500261 |
| YDR303C | 505.650581032068 | 0.473280613334911 | 0.288727323665371 | 1.63919578973909 | 0.101172490406979 | 0.364698390898198 | 587.939516548344 | 682.652890516963 | 492.974862339578 | 479.501702658947 | 379.550936240427 | 411.283577888147 |
| YDR304C | 7322.47418308546 | -0.276021038206519 | 0.248799433530347 | -1.10941184346729 | 0.267252555471292 | 0.593596753967041 | 5993.05896154803 | 6636.76618831112 | 7243.22710173558 | 7545.40558080204 | 8315.61596672209 | 8200.77129939392 |
| YDR305C | 338.124747786405 | -0.574757228782661 | 0.347924268083051 | -1.65196073257374 | 0.0985425559923346 | 0.360090662329269 | 341.667958408417 | 190.994942292652 | 281.521177731993 | 424.493931574695 | 316.106938100532 | 473.96353861014 |
| YDR306C | 440.346515005697 | 0.667594453498753 | 0.293870161257969 | 2.27173269528619 | 0.0231026601614122 | 0.166617428617061 | 441.800350179596 | 641.98945119014 | 538.018250776697 | 320.705684245919 | 378.437883641482 | 321.127470000348 |
| YDR307W | 1175.37273782362 | -0.0977589542748887 | 0.306249545667356 | -0.319213385482286 | 0.749564707376756 | 0.908603445448746 | 809.854546960148 | 1267.95997173638 | 1330.03116412937 | 1234.04226073915 | 1266.65385760002 | 1143.69462577664 |
| YDR308C | 315.877418079284 | -0.267420586361861 | 0.297709815192384 | -0.898259219935528 | 0.369047368091136 | 0.682084974851399 | 274.687507156073 | 317.914162009705 | 267.757920153984 | 405.812047055516 | 280.489254934275 | 348.603617166154 |
| YDR309C | 406.364095996332 | 0.279661289195512 | 0.373985456063239 | 0.747786537314498 | 0.454588923763351 | 0.746351652039915 | 672.510793382111 | 322.843063746289 | 340.32782374712 | 372.599807910307 | 338.367990079443 | 391.535097112724 |
| YDR310C | 165.297011879071 | 0.305138461408929 | 0.739507895701996 | 0.412623669310885 | NA | NA | 449.242622540967 | 48.0567919316995 | 50.0482093745765 | 124.545896794532 | 103.513891701935 | 216.374658930716 |
| YDR311W | 97.2922448411707 | -0.513422027210708 | 0.334454165015202 | -1.53510430102544 | 0.12475820998548 | 0.406181701839891 | 85.9244172631067 | 67.7723988780378 | 86.3331611711444 | 121.432249374668 | 123.548838482954 | 98.7424038771124 |
| YDR312W | 14.8307478508676 | 0.286440265180326 | 0.597928539153042 | 0.479054345835482 | 0.631899967198487 | 0.851680835678705 | 18.9439660107637 | 11.0900289073153 | 18.7680785154662 | 15.5682370993165 | 10.0174733905098 | 14.596703181834 |
| YDR313C | 408.620344037474 | -0.453065008359765 | 0.288394290091936 | -1.5709916039438 | 0.116184599039454 | 0.392840632516929 | 297.690894454858 | 332.700867219458 | 405.390495934069 | 415.152989315105 | 494.195353931817 | 506.591463369533 |
| YDR314C | 19.0282418745042 | 0.904051753189863 | 0.663159036310338 | 1.36325029697219 | 0.172803640553608 | 0.481704813725577 | 41.9473533095482 | 19.7156069463383 | 12.5120523436441 | 12.4545896794532 | 7.79136819261874 | 19.7484807754225 |
| YDR315C | 89.557763171715 | -0.902472406940504 | 0.379537172644975 | -2.37782349657932 | 0.0174151596959861 | 0.145075852076127 | 60.8913193203119 | 82.5591040877915 | 43.7921832027544 | 133.886839054122 | 115.757470290336 | 100.459663074975 |
| YDR316W | 42.3358871730599 | -0.0603367042313001 | 0.460929807590801 | -0.130902153077644 | 0.895852711430979 | 0.966310161494771 | 30.4456596601559 | 43.127890195115 | 51.2994146089409 | 60.1971834506903 | 31.165472770475 | 37.7797023529822 |
| YDR317W | 58.9283957614999 | 0.602349862170654 | 0.416176325862221 | 1.44734292831944 | 0.147800901555535 | 0.441506511091813 | 46.006774597569 | 76.3979769170608 | 91.337982108602 | 48.7804762445249 | 48.9743143536035 | 42.0728503476392 |
| YDR318W | 148.259731175101 | 0.121516580918581 | 0.374584393244348 | 0.324403747486921 | 0.745632382944872 | 0.906683930012085 | 104.1918130592 | 190.994942292652 | 168.912706639196 | 149.455076153438 | 166.95788984183 | 109.045959064289 |
| YDR319C | 1192.03898302965 | 0.389226038684943 | 0.362328344723647 | 1.07423568802438 | 0.282717060987618 | 0.608180264429951 | 787.527729876033 | 1649.94985632168 | 1619.05957326755 | 999.480821776116 | 1162.02691329914 | 934.189003637377 |
| YDR320C | 179.063426529028 | 0.0965117040970625 | 0.356050724504837 | 0.271061670303529 | 0.786343600961325 | 0.925456921344373 | 258.44982200399 | 149.099277531683 | 146.391012420636 | 158.796018413028 | 184.766731424959 | 176.877697379871 |
| YDR320C-A | 897.719894218933 | -1.98019580476383 | 0.301424000565908 | -6.56946958784341 | 5.04948062300176e-11 | 2.63858314736492e-08 | 294.308043381507 | 381.989884585304 | 414.14893257462 | 1724.96067060426 | 1236.60143742849 | 1334.31039673942 |
| YDR320W-B | 30.8104479756367 | 0.885762615046992 | 0.621609671848303 | 1.42494986027687 | 0.154171695258821 | 0.453044608668868 | 13.5314042934026 | 43.127890195115 | 63.811466952585 | 29.0607092520574 | 15.5827363852375 | 19.7484807754225 |
| YDR321W | 10799.2366795073 | 1.27923744214096 | 0.359767301234778 | 3.55573571514259 | 0.000376922832349687 | 0.0137123572173798 | 8716.25407559531 | 17884.5199511971 | 19288.5798929618 | 6349.76497157454 | 6534.73180840923 | 6021.569377306 |
| YDR322C-A | 3324.4154075344 | -1.32773665322315 | 0.319012310461682 | -4.16202325014235 | 3.1544022430399e-05 | 0.00240328996461688 | 1275.3348546532 | 2204.45130168745 | 2204.62362295009 | 5139.594007721 | 4535.68934070305 | 4586.79931749161 |
| YDR322W | 651.204770154414 | -0.639811999635468 | 0.396769028785386 | -1.61255529846697 | 0.106841156815299 | 0.375151477931789 | 816.620249106849 | 357.345375902381 | 351.5886708564 | 812.661976584319 | 771.345451069256 | 797.666897407282 |
| YDR323C | 83.2022758759481 | -0.252760065718775 | 0.38858679890265 | -0.650459733661968 | 0.515395303456713 | 0.783453722438315 | 104.86838327387 | 52.9856936682841 | 68.8162878900426 | 96.523070015762 | 90.1572605145883 | 85.8629598931413 |
| YDR324C | 394.70711172804 | 1.22993490888569 | 1.16037641557605 | 1.05994476652226 | NA | NA | 1428.23972316865 | 134.31257232193 | 97.5940082804241 | 276.076737894545 | 135.792417071355 | 296.227211631337 |
| YDR325W | 131.4009263533 | -0.0457694076397645 | 0.415133834985047 | -0.110252173594603 | 0.912209385891362 | 0.975149628064636 | 198.235072898349 | 101.042485599984 | 87.5843664055088 | 115.204954534942 | 125.774943680845 | 160.563735000174 |
| YDR326C | 175.980579020493 | 0.423644422359598 | 0.380529790817438 | 1.11330159315396 | 0.265578922449697 | 0.592894614644195 | 281.453209302775 | 193.459393160944 | 128.874139139534 | 161.909665832891 | 161.392626847103 | 128.794439839712 |
| YDR327W | 2.39879593609119 | 0.103719121417597 | 1.34574473032653 | 0.0770719134768079 | 0.93856633632182 | NA | 1.35314042934026 | 1.23222543414614 | 5.00482093745765 | 3.11364741986329 | 1.11305259894553 | 2.57588879679424 |
| YDR328C | 1403.61628470767 | -0.0765462997008949 | 0.542105792410689 | -0.141201774215511 | 0.887710544498493 | 0.963474359852217 | 2769.87845885952 | 619.80939337551 | 708.182162650257 | 1433.3156956104 | 1169.81828149176 | 1720.69371625855 |
| YDR329C | 330.579849401907 | -0.278640734742636 | 0.400744392230079 | -0.695307882393673 | 0.486862375497951 | 0.766813881410433 | 476.305431127773 | 214.407225541429 | 203.946453201399 | 347.690628551401 | 376.211778443591 | 364.91757954585 |
| YDR330W | 52.9841299733242 | 0.823378085130248 | 0.632662383621007 | 1.30144940879477 | 0.193104670773505 | 0.507162802399533 | 142.756315295398 | 27.1089595512151 | 32.5313360934747 | 39.439533984935 | 32.2785253694205 | 43.790109545502 |
| YDR331W | 1586.63669157755 | 0.297500029471259 | 0.297646182688311 | 0.999508969959796 | 0.317548195993772 | 0.637790742294482 | 1286.83654830259 | 1917.3427755314 | 2045.72055818581 | 1476.90675948849 | 1456.9858520197 | 1336.02765593728 |
| YDR332W | 63.7200407900245 | 0.762688726065266 | 0.58682595370562 | 1.29968472125189 | 0.19370904906719 | 0.507956028302102 | 158.994000447481 | 33.2700867219458 | 47.5457989058476 | 47.7425937712371 | 38.9568409630937 | 55.8109239305418 |
| YDR333C | 816.320730515765 | 0.272592390454449 | 0.254556388469433 | 1.07085267862834 | 0.284235676197818 | 0.608839674843081 | 901.868096155286 | 837.913295219377 | 939.655131007673 | 793.980092065139 | 740.17997829878 | 684.327790348336 |
| YDR334W | 217.361674445722 | 0.000278301403645555 | 0.36627607966431 | 0.000759813209480175 | 0.999393756829394 | 0.999567655168846 | 308.51601788958 | 177.440462517044 | 165.159090936102 | 212.765907023992 | 186.99283662285 | 253.295731684767 |
| YDR335W | 1156.85737767683 | 0.083824999793462 | 0.257618253910377 | 0.325384550671723 | 0.74489004668586 | 0.906158398733353 | 1253.00803756908 | 1239.61878675102 | 1078.53891202212 | 1208.09519890696 | 1017.33007543622 | 1144.55325537557 |
| YDR336W | 347.820995225996 | -0.264759785213606 | 0.275158370537908 | -0.962208726182041 | 0.335944766648169 | 0.650515759457194 | 328.813124329684 | 346.255346995066 | 272.762741091442 | 373.637690383595 | 366.194305053081 | 399.262763503107 |
| YDR337W | 123.530345146366 | -0.0548422280575027 | 0.626263901606133 | -0.0875704761472806 | 0.930218068626487 | 0.980512845043758 | 255.74354114531 | 51.753468234138 | 55.0530303120341 | 153.606606046589 | 67.8962085356776 | 157.129216604448 |
| YDR338C | 757.756138400962 | 0.556540715498536 | 0.264298128749413 | 2.10573082046375 | 0.0352277410413103 | 0.21070661342919 | 813.237398033498 | 951.278035160822 | 942.157541476402 | 562.532300521968 | 682.301243153613 | 595.030312059469 |
| YDR339C | 578.926545927881 | -0.0990216723703312 | 0.333261379686001 | -0.297129155690435 | 0.766367905953029 | 0.916279634187422 | 767.23062343593 | 464.548988673096 | 444.177858199366 | 639.335603545262 | 522.021668905456 | 636.244532808177 |
| YDR340W | 1.08321750051981 | -0.360360145773075 | 2.27436330008443 | -0.15844440761056 | 0.874106623484364 | NA | 2.70628085868053 | 0 | 0 | 2.07576494657553 | 0 | 1.71725919786283 |
| YDR341C | 12644.1784843539 | 0.105423970941698 | 0.248997382946976 | 0.423393891509889 | 0.67200790090153 | 0.87058290537205 | 12444.8325286424 | 12600.7372895785 | 14272.4981083948 | 13291.1229529231 | 11869.5929151552 | 11386.2871114295 |
| YDR342C | 6519.53439457625 | -0.436281320129912 | 0.435646418671375 | -1.00145737789025 | 0.316605736228526 | 0.63675639322658 | 2521.57719007558 | 7500.55621764757 | 6602.610021741 | 6202.38566036768 | 9755.90602975761 | 6534.17124786805 |
| YDR343C | 1359.61878470065 | 0.078820016100405 | 0.549934379726049 | 0.143326220375001 | 0.886032548743529 | 0.96256191460552 | 553.434435600168 | 1864.35708186311 | 1772.95781709437 | 702.646434415816 | 2331.8451947909 | 932.471744439514 |
| YDR344C | 13.113200172781 | 0.491485865730257 | 0.681506319698681 | 0.721175800611153 | 0.470801358675054 | 0.758293350262115 | 18.2673957960936 | 11.0900289073153 | 16.2656680467373 | 7.26517731301435 | 18.9218941820741 | 6.8690367914513 |
| YDR345C | 50175.2769012244 | 0.315827391914845 | 0.370168905313221 | 0.853198060079104 | 0.393549478995604 | 0.701925497964819 | 30687.1952267932 | 67647.9441091891 | 68602.3317949663 | 45611.8210535773 | 48336.5352144077 | 40165.8340084125 |
| YDR346C | 1754.13400265748 | 0.582507317330022 | 0.34064401890248 | 1.71001774581805 | 0.0872625915719355 | 0.338451670955118 | 3002.61861270605 | 1797.81690841922 | 1508.95351264348 | 1416.7095760378 | 1418.02901105661 | 1380.67639508171 |
| YDR347W | 1931.52029457675 | -0.380029671486895 | 0.280662189808951 | -1.35404655591686 | 0.175721525141598 | 0.484435168591802 | 1970.84903533409 | 1318.48121453637 | 1745.43130193835 | 2251.16708456116 | 2173.79172574063 | 2129.4014053499 |
| YDR348C | 59.0559127639487 | 2.2864478601082 | 0.900788783089736 | 2.53827301475225 | NA | NA | 265.215524150692 | 14.7867052097537 | 13.7632575780085 | 23.8712968856186 | 17.8088415831286 | 18.8898511764911 |
| YDR349C | 3560.81059882731 | 0.176838954001208 | 0.245226791986942 | 0.721124117672364 | 0.470833153633522 | 0.758293350262115 | 3700.83907424562 | 3786.6287591311 | 3848.70730090493 | 3441.61828142222 | 3559.54221142782 | 3027.52796583216 |
| YDR350C | 1118.46546833261 | 0.180378285858846 | 0.299613545958804 | 0.602036484303841 | 0.547149851936006 | 0.801891215942927 | 952.610862255545 | 1366.53800646807 | 1246.20041342695 | 1012.97329392886 | 1245.50585822005 | 886.964375696149 |
| YDR351W | 183.221240347005 | -0.0374275706159173 | 0.289074348217259 | -0.12947385628208 | 0.89698271047629 | 0.966421109619065 | 190.792800536977 | 168.814884478021 | 182.675964217204 | 204.462847237689 | 171.410100237612 | 181.170845374528 |
| YDR352W | 2851.82721770192 | 0.353694811637371 | 0.317600475898855 | 1.11364698253793 | 0.265430662287465 | 0.592894614644195 | 2201.55947853661 | 3493.35910580431 | 3905.01153645133 | 2694.34290065503 | 2553.34266198106 | 2263.3476227832 |
| YDR353W | 13291.3340541486 | -0.117275897484409 | 0.25261351109301 | -0.464250296735826 | 0.642468429611053 | 0.858125316890341 | 13933.2870009167 | 11082.6355547104 | 13237.7513795755 | 13230.9257694724 | 14104.6025338378 | 14158.802086379 |
| YDR354W | 4580.2618073985 | 0.251496507715055 | 0.352546224833829 | 0.71337172262615 | 0.475615766279382 | 0.760667619525288 | 2921.43018694563 | 5775.44060984297 | 6239.76050377532 | 4314.47744145723 | 4081.56388033328 | 4148.89822203659 |
| YDR356W | 166.727458620079 | -0.091068108424876 | 0.338436073277599 | -0.269085111238063 | 0.787864187536932 | 0.92631956251959 | 117.723217352603 | 170.047109912168 | 197.690427029577 | 150.492958626726 | 178.088415831286 | 186.322622968117 |
| YDR357C | 203.086684131276 | -1.17989149136396 | 0.329056140847663 | -3.58568446200247 | 0.000336195264692448 | 0.0127134893516592 | 112.987225849912 | 134.31257232193 | 126.371728670806 | 349.766393497976 | 212.593046398597 | 282.489138048435 |
| YDR358W | 58.5092928194982 | 0.14539897286192 | 0.537505572384607 | 0.270506912545794 | 0.786770299649204 | 0.925574228895543 | 110.280944991231 | 39.4312138926766 | 33.7825413278391 | 57.083536030827 | 40.0698935620392 | 70.4076271123758 |
| YDR359C | 103.498394888651 | -0.781782883196818 | 0.348594822439092 | -2.24266923337169 | 0.0249181520489677 | 0.175526394580229 | 98.1026811271691 | 66.5401734438917 | 62.5602617182206 | 134.924721527409 | 125.774943680845 | 133.087587834369 |
| YDR360W | 2.43639699237014 | 1.42493538841673 | 1.36874966968035 | 1.041048936837 | 0.29785283669646 | NA | 2.0297106440104 | 2.46445086829228 | 6.25602617182206 | 1.03788247328776 | 1.11305259894553 | 1.71725919786283 |
| YDR361C | 1128.0869538734 | 0.136401110558449 | 0.370594614266691 | 0.368060153352069 | 0.71282838153645 | 0.891399851461647 | 1730.6666091262 | 935.259104516922 | 877.094869289452 | 1234.04226073915 | 831.450291412314 | 1160.00858815634 |
| YDR362C | 472.479967514234 | 0.250607671976185 | 0.265357591768247 | 0.944414931964921 | 0.344957653985327 | 0.656996883733486 | 454.655184258329 | 559.430347102349 | 526.757403667417 | 447.327345987026 | 417.394724604576 | 429.314799465706 |
| YDR363W | 38.2332869322445 | 0.448710452303699 | 0.413555921396119 | 1.08500550732995 | 0.277919216060589 | 0.60329343886538 | 40.5942128802079 | 48.0567919316995 | 43.7921832027544 | 29.0607092520574 | 37.8437883641482 | 30.0520359625994 |
| YDR363W-A | 114.265452509634 | -0.65531086930305 | 0.489418874565343 | -1.33895708432789 | 0.18058464432819 | 0.491244929294102 | 138.696894007377 | 64.0757225755994 | 62.5602617182206 | 165.023313252754 | 72.3484189314597 | 182.888104572391 |
| YDR364C | 391.758539541675 | 0.206691064921605 | 0.28082034311629 | 0.736025968161478 | 0.461714894432974 | 0.750658008972072 | 473.599150269092 | 372.132081112135 | 412.897727340256 | 408.925694475379 | 347.272410871007 | 335.724173182182 |
| YDR365C | 16.9593096005958 | 0.982088559595913 | 0.644542284632817 | 1.52369919400306 | 0.127583870906829 | 0.408325217133884 | 20.297106440104 | 20.9478323804844 | 26.2753099216526 | 4.15152989315105 | 18.9218941820741 | 11.1621847861084 |
| YDR366C | 3.94768743560503 | 0.220178859466098 | 1.00889557225203 | 0.218237511910793 | 0.827244059119367 | NA | 4.05942128802079 | 3.69667630243843 | 5.00482093745765 | 4.15152989315105 | 3.3391577968366 | 3.43451839572565 |
| YDR367W | 2944.06090434767 | 0.370574114889976 | 0.321924354615984 | 1.15112171408101 | 0.249682166003599 | 0.572923389296881 | 2240.80055098748 | 3622.74277638966 | 4097.69714254345 | 2579.13794612009 | 2767.0487609786 | 2356.93824906673 |
| YDR368W | 3127.04477019668 | 0.219601593502385 | 0.285397539285861 | 0.769458608689779 | 0.44162110726355 | 0.737703610738415 | 2613.59073927072 | 3489.66242950188 | 3991.34469762247 | 2920.60127983177 | 3047.53801591287 | 2699.53145904036 |
| YDR369C | 134.855564049305 | 0.071205490043375 | 0.3916570903248 | 0.181805696366494 | 0.855735213973376 | 0.950353507634853 | 91.3369789804678 | 181.137138819483 | 142.637396717543 | 133.886839054122 | 161.392626847103 | 98.7424038771124 |
| YDR370C | 292.408994035894 | -0.136161265478733 | 0.282679802014148 | -0.481680206751802 | 0.630033138931958 | 0.850071340307741 | 318.664571109632 | 259.999566604836 | 256.497073044704 | 330.046626505509 | 282.715360132166 | 306.530766818514 |
| YDR371C-A | 3.42101222800805 | 0.841086208742557 | 1.1724913734426 | 0.717349592324088 | 0.4731584143602 | NA | 2.0297106440104 | 4.92890173658457 | 6.25602617182206 | 3.11364741986329 | 3.3391577968366 | 0.858629598931413 |
| YDR371W | 139.284185743691 | -0.04305997324929 | 0.379811070485331 | -0.113372085743228 | 0.909735569354814 | 0.973410285303699 | 195.528792039668 | 96.1135838633991 | 118.864497264619 | 155.682370993165 | 123.548838482954 | 145.96703181834 |
| YDR372C | 1874.38079097517 | -0.0379635531240122 | 0.260830504579394 | -0.145548747011899 | 0.88427763093 | 0.961928051208485 | 2023.62151207836 | 1923.50390270213 | 1601.54269998645 | 2026.984470331 | 1754.17089593816 | 1916.46126481491 |
| YDR373W | 1562.5522904041 | 0.212337601615504 | 0.38013083543872 | 0.558590837205928 | 0.576440994800148 | 0.817311997560742 | 891.042972720564 | 1991.27630158017 | 2150.82179787242 | 1533.99029551931 | 1436.95090523869 | 1371.23146949347 |
| YDR374C | 24.2229991608001 | -1.0199706109036 | 0.595772074955845 | -1.71201480193444 | 0.0868939406975454 | 0.337746780793453 | 8.79541279071171 | 9.85780347316914 | 30.0289256247459 | 33.2122391452084 | 33.391577968366 | 30.0520359625994 |
| YDR374W-A | 490.973876914249 | -0.919286491617217 | 0.362279588260515 | -2.53750562108997 | 0.0111645575176136 | 0.110556981316825 | 202.294494186369 | 417.724422175542 | 400.385674996612 | 599.896069560327 | 727.93639971038 | 597.606200856263 |
| YDR375C | 943.229748851836 | -0.907463530853654 | 0.404983194783526 | -2.24074367169413 | 0.0250426835051724 | 0.175757441743261 | 1064.24494767612 | 423.885549346273 | 477.960399527205 | 1280.7469720371 | 1099.69596775819 | 1312.84465676613 |
| YDR376W | 1270.67028239901 | 0.205337946460253 | 0.282876976001503 | 0.725891337509072 | 0.467905387350067 | 0.756950824254128 | 1065.59808810546 | 1447.86488512172 | 1570.26256912734 | 1209.13308138024 | 1213.22733285063 | 1117.9357378087 |
| YDR377W | 4707.30687435318 | -0.834862245105638 | 0.378588648334797 | -2.20519619058241 | 0.0274403303963338 | 0.184267290042603 | 1807.11904338392 | 3888.90347016523 | 4451.78822386858 | 6488.8412229951 | 5894.72656401555 | 5712.46272169069 |
| YDR378C | 1528.11198417347 | -0.154498118625477 | 0.373260089890962 | -0.41391545147677 | 0.678936027484964 | 0.874417272234724 | 806.471695886797 | 1679.52326674119 | 1854.28615732806 | 1630.51336553508 | 1781.9972109118 | 1415.8802086379 |
| YDR379C-A | 359.776222057126 | -1.89942947576279 | 0.32800382600811 | -5.79087597507424 | 7.00202692339109e-09 | 2.0123825377826e-06 | 107.574664132551 | 157.724855570706 | 192.685606092119 | 616.502189132932 | 550.96103647804 | 533.208980936407 |
| YDR379W | 58.1018124855737 | -0.222590382233517 | 0.462030842455105 | -0.481765202190255 | 0.629972751690111 | 0.850071340307741 | 83.8947066190963 | 38.1989884585304 | 37.5361570309323 | 72.6517731301435 | 65.6701033377865 | 50.6591463369533 |
| YDR380W | 2878.59237113121 | -0.289974281196524 | 0.334672415143223 | -0.866442132891142 | 0.386247747142443 | 0.697065008029752 | 3612.8849463385 | 2114.49884499478 | 2041.96694248272 | 3565.12629574347 | 2748.12686679653 | 3188.95033043127 |
| YDR381C-A | 187.268996055301 | -0.253274564032308 | 0.422799907022197 | -0.599041200874746 | 0.549145410152162 | 0.803335259270233 | 105.544953488541 | 224.265029014598 | 183.927169451568 | 249.091793589063 | 124.6618910819 | 236.123139706138 |
| YDR381W | 2460.62181506972 | 0.160573749485116 | 0.506255506204449 | 0.317179265246883 | 0.751107582208973 | 0.908603445448746 | 5049.24351208319 | 1441.70375795099 | 1300.00223850462 | 2281.2656762865 | 2125.93046398597 | 2565.58524160706 |
| YDR382W | 79915.5758119555 | 0.113500739883279 | 0.370490726097355 | 0.306352445252447 | 0.759336311904138 | 0.912866087924729 | 45344.4123574069 | 96633.5829966088 | 107195.757248937 | 74909.1675095443 | 80255.5445943688 | 75154.9901648676 |
| YDR383C | 27.354664739502 | -0.284678132576975 | 0.506304781872173 | -0.562266331999305 | 0.573934573589295 | 0.815967966795959 | 32.4753703041663 | 23.4122832487767 | 17.5168732811018 | 40.4774164582228 | 24.4871571768018 | 25.7588879679424 |
| YDR384C | 2130.02256119171 | 1.22896316181381 | 0.366635443193965 | 3.35200315361666 | 0.000802291048739395 | 0.0221710045584329 | 1660.97987701517 | 3587.00823879942 | 3711.07472512484 | 1274.51967719737 | 1287.80185697998 | 1258.75099203345 |
| YDR385W | 37710.3987111085 | 1.0903467468057 | 0.383136960374645 | 2.84584067728552 | 0.00442943505674682 | 0.066303106005679 | 26516.8164235665 | 63006.1508987605 | 64434.5671592985 | 24010.3731370391 | 25205.0761031216 | 23089.4085448646 |
| YDR386W | 55.250604844954 | -0.232214847833866 | 0.440587294081343 | -0.527057522886699 | 0.598153643141098 | 0.828750365770883 | 68.3335916816833 | 28.3411849853613 | 55.0530303120341 | 65.3865958171291 | 53.4265247493857 | 60.9627015241303 |
| YDR387C | 1825.80489912913 | 0.849328070091196 | 0.370510577416723 | 2.29231801157443 | 0.0218872992248704 | 0.162123963846076 | 1354.4935697696 | 2979.52109976537 | 2711.36174286768 | 1168.65566492202 | 1551.59532293008 | 1189.20199452001 |
| YDR388W | 3205.59742459851 | 0.52688003140929 | 0.245167922999069 | 2.1490577762544 | 0.031629820289495 | 0.199569930871589 | 3572.96730367297 | 4074.96951072129 | 3706.06990418739 | 2497.14523073036 | 2616.78666012095 | 2765.64593815808 |
| YDR389W | 184.917763982537 | -0.244185277344322 | 0.467093953015018 | -0.52277550537348 | 0.601130487205636 | 0.830800202081749 | 299.044034884198 | 99.8102601658375 | 107.603650155339 | 216.917436917143 | 179.201468430231 | 206.92973334247 |
| YDR390C | 519.975101233985 | -0.241244940577268 | 0.464371930364759 | -0.519508016748067 | 0.603406523906149 | 0.831183519646816 | 851.125330055026 | 296.96632962922 | 280.269972497628 | 584.327832461011 | 467.482091557125 | 639.679051203902 |
| YDR391C | 1336.35016797373 | 0.145641010157262 | 0.329642022981786 | 0.441815666703723 | 0.658622600692282 | 0.86511223916506 | 1026.35701565459 | 1547.67514528755 | 1637.82765178301 | 1107.42059899804 | 1608.3610054763 | 1090.45959064289 |
| YDR392W | 98.2186293899107 | 0.327451562196958 | 0.459261354447926 | 0.71299611653714 | 0.475848160219989 | 0.760668954287083 | 184.027098390276 | 71.4690751804763 | 71.3186983587715 | 93.4094225958987 | 82.3658923219696 | 86.7215894920727 |
| YDR393W | 46.3871224736162 | -0.901186590699344 | 0.400198371347239 | -2.25184972059123 | 0.0243317704044336 | 0.172665452203314 | 39.2410724508676 | 29.5734104195074 | 27.5265151560171 | 64.3487133438414 | 60.1048403430589 | 57.5281831284046 |
| YDR394W | 2036.04346799156 | 0.0609855927253863 | 0.435401563078226 | 0.140067463915901 | 0.888606687165111 | 0.963909826437756 | 3593.94098032774 | 1261.79884456565 | 1380.07937350395 | 1974.05246419333 | 1833.1976304633 | 2173.19151489541 |
| YDR395W | 4943.9015584268 | 0.449367354952909 | 0.248626250580951 | 1.80740108457131 | 0.0706997649797847 | 0.303672139518666 | 5792.117607791 | 5373.73511831133 | 5956.98812080896 | 4595.74359171822 | 4101.5988271143 | 3843.226084817 |
| YDR396W | 48.7839764263296 | 0.185967438131105 | 0.440211183684153 | 0.422450507901078 | 0.672696218267633 | 0.870957457133399 | 31.1222298748261 | 61.6112717073071 | 63.811466952585 | 49.8183587178127 | 43.4090513588758 | 42.9314799465706 |
| YDR397C | 442.918276358392 | -0.305353085965957 | 0.483184787336003 | -0.631959229613776 | 0.527413518600447 | 0.791959165822418 | 718.51756797968 | 193.459393160944 | 275.265151560171 | 528.282178903472 | 398.472830422501 | 543.512536123584 |
| YDR398W | 218.033685231561 | 1.69238032050344 | 1.18606766461186 | 1.42688344940022 | NA | NA | 873.45214713914 | 60.379046273161 | 65.0626721869494 | 116.24283700823 | 74.5745241293508 | 118.490884652535 |
| YDR399W | 4580.44264811396 | 0.10263269956268 | 0.257154693729994 | 0.399108793520377 | 0.689813043176195 | 0.879721793562251 | 4737.34464312026 | 4735.44234342363 | 4757.08230105349 | 5124.02577062169 | 3974.7108308345 | 4154.04999963017 |
| YDR400W | 2440.59043492611 | 0.367453576551888 | 0.344133636497671 | 1.06776419850018 | 0.285626891297846 | 0.610456523844073 | 1673.83471109391 | 3244.44956810679 | 3331.95953911243 | 2170.21225164471 | 2202.73109331321 | 2020.35544628561 |
| YDR401W | 130.473510998576 | -0.306192294362607 | 0.449646384027102 | -0.680962430121871 | 0.495895263266898 | 0.773875893211537 | 52.0959065296001 | 155.260404702414 | 143.888601951907 | 126.621661741107 | 154.714311253429 | 150.260179812997 |
| YDR402C | 17.9082728024715 | 0.0402610326219641 | 0.551172390803744 | 0.0730461708418552 | 0.941769376599101 | 0.983779795011854 | 19.6205362254338 | 13.5544797756076 | 21.270488984195 | 20.7576494657553 | 13.3566311873464 | 18.8898511764911 |
| YDR403W | 145.172437610739 | 0.719051792300582 | 0.375705640922387 | 1.91387009930235 | 0.0556367557936728 | 0.267167980202198 | 115.016936493922 | 184.833815121921 | 242.733815466696 | 107.939777221927 | 115.757470290336 | 104.752811069632 |
| YDR404C | 3790.65594223198 | 0.354711732023831 | 0.348614564452249 | 1.01748970982082 | 0.308920525001532 | 0.633323413761106 | 2539.84458587167 | 4997.90636089675 | 5226.28426394015 | 3296.31473516194 | 3497.21126588687 | 3186.37444163447 |
| YDR405W | 108.958655259229 | -0.298385478558841 | 0.517091561232474 | -0.577045732186477 | 0.563908562408347 | 0.811311596596349 | 182.673957960936 | 60.379046273161 | 48.797004140212 | 122.470131847956 | 104.62694430088 | 134.804847032232 |
| YDR406W | 847.926872276662 | 0.28760574602034 | 0.351696371351372 | 0.817767169206872 | 0.413490148008582 | 0.714920346447085 | 605.530342129768 | 1083.12615661446 | 1108.56783764687 | 704.722199362392 | 941.642498707922 | 643.97219919856 |
| YDR406W-A | 40.1368175081326 | -0.722640659644461 | 0.483743940043023 | -1.49384953448759 | 0.135214951218065 | 0.422244782545739 | 16.2376851520832 | 44.3601156292611 | 31.2801308591103 | 49.8183587178127 | 46.7482091557125 | 52.3764055348162 |
| YDR407C | 296.376291302884 | 0.533972045770463 | 0.311781266580368 | 1.71264954956112 | 0.0867770317481744 | 0.337707771488495 | 442.476920394266 | 288.340751590197 | 320.308539997289 | 245.9781461692 | 219.27136199227 | 261.882027674081 |
| YDR408C | 1592.33942674998 | -0.384201784392857 | 0.335400792792444 | -1.14550052548806 | 0.252001877882599 | 0.575522774566069 | 1912.66399687246 | 991.941474487645 | 1238.69318202077 | 1761.28655716933 | 1653.99616203306 | 1995.4551879166 |
| YDR409W | 112.787340803038 | -0.172376302277154 | 0.440666382990779 | -0.391171890869563 | 0.695670184127087 | 0.883320378312284 | 170.495694096873 | 73.9335260487685 | 72.5699035931359 | 126.621661741107 | 95.722523509316 | 137.380735829026 |
| YDR410C | 2025.56848183297 | 0.369768275356428 | 0.402300682738773 | 0.919134098503458 | 0.358025435854552 | 0.672306502872252 | 1095.36717755094 | 2916.67760262392 | 2840.23588200721 | 1740.52890770358 | 1828.74542006751 | 1731.85590104466 |
| YDR411C | 2690.41796220485 | 0.187168336792403 | 0.326493662934312 | 0.573267900853745 | 0.56646331577958 | 0.812941172023297 | 1870.71664356291 | 3290.0419091702 | 3434.55836833031 | 2360.14474425637 | 2573.37760876208 | 2613.66849914722 |
| YDR412W | 33.2714995529535 | 0.504307148824367 | 0.673393407248349 | 0.748904196857363 | 0.453914950585804 | 0.745884258424013 | 79.1587151164054 | 24.6445086829228 | 12.5120523436441 | 17.644002045892 | 35.6176831662571 | 30.0520359625994 |
| YDR413C | 4.15160037185548 | 1.7140241724032 | 1.08840597342607 | 1.57480224681956 | 0.115302097654701 | NA | 6.76570214670132 | 8.625578039023 | 3.75361570309323 | 2.07576494657553 | 1.11305259894553 | 2.57588879679424 |
| YDR414C | 1175.54641239514 | 0.426299865953632 | 0.334993929515044 | 1.2725599731636 | 0.203174229781347 | 0.520894501687415 | 867.363015207109 | 1577.24855570706 | 1600.29149475208 | 1096.00389179188 | 985.051550066798 | 927.319966845926 |
| YDR415C | 1056.67515649306 | -0.180326422504197 | 0.249505708668948 | -0.722734655917072 | 0.469842919621042 | 0.758293350262115 | 940.432598391483 | 975.922543843745 | 1056.01721780356 | 1140.63283814325 | 1170.9313340907 | 1056.11440668564 |
| YDR416W | 430.403238791639 | 0.401298031709358 | 0.255845648152657 | 1.56851615263713 | 0.116760723322439 | 0.393861876559495 | 477.658571557113 | 487.961271921872 | 504.235709448858 | 371.561925437019 | 360.629042058353 | 380.372912326616 |
| YDR417C | 18.4776079922154 | -0.281913166851689 | 0.552848126441884 | -0.509928773144398 | 0.61010136307945 | 0.836161810915756 | 10.8251234347221 | 18.4833815121921 | 21.270488984195 | 20.7576494657553 | 18.9218941820741 | 20.6071103743539 |
| YDR418W | 78794.8697207839 | -0.348888855169936 | 0.29956282042935 | -1.16466006919647 | 0.244156618337486 | 0.567946943988665 | 49507.3488882722 | 77046.1274954217 | 81388.3980849362 | 87984.4109080236 | 86604.3966187542 | 90238.5363292957 |
| YDR419W | 160.078738143063 | 0.36053683482119 | 0.403885516809552 | 0.892670867896452 | 0.372033455601624 | 0.684522504096715 | 274.687507156073 | 145.402601229245 | 118.864497264619 | 134.924721527409 | 126.887996279791 | 159.705105401243 |
| YDR420W | 343.422213925108 | 0.499828913423622 | 0.552313161621328 | 0.904973750682244 | 0.365479293575979 | 0.680743674489542 | 816.620249106849 | 231.658381619475 | 157.651859529916 | 290.607092520574 | 254.889045158527 | 309.106655615309 |
| YDR421W | 51.4464890449706 | 0.615715022285485 | 0.56360649597117 | 1.09245551051452 | 0.274632935241283 | 0.600152316299675 | 113.663796064582 | 29.5734104195074 | 42.54097796839 | 46.7047112979494 | 47.861261754658 | 28.3347767647366 |
| YDR422C | 449.997047619242 | 0.521039145805356 | 0.349547120597175 | 1.49061203798561 | 0.136063381952979 | 0.423528462169476 | 759.111780859888 | 398.008815229204 | 432.917011090086 | 395.433222322638 | 346.159358272061 | 368.352097941576 |
| YDR423C | 166.396328371254 | -0.213411555146804 | 0.348268304317018 | -0.612779149010764 | 0.540022379647308 | 0.798710611079734 | 196.881932469008 | 134.31257232193 | 130.125344373899 | 140.114133893848 | 211.479993799652 | 185.463993369185 |
| YDR424C | 1324.37326453024 | -1.08925123871489 | 0.334335462131533 | -3.25795903243541 | 0.0011221660525621 | 0.027331400297148 | 545.315593024126 | 970.99364210716 | 1025.98829217882 | 1949.14328483442 | 1805.37131548966 | 1649.42745954724 |
| YDR425W | 70.5356883408073 | -0.447678446888311 | 0.352937015267317 | -1.26843722115471 | 0.204641856535675 | 0.522559480838321 | 65.6273108230028 | 55.4501445365764 | 57.5554407807629 | 94.4473050691865 | 74.5745241293508 | 75.5594047059643 |
| YDR426C | 8.12403307302501 | 0.131662027511055 | 0.85248240491941 | 0.154445448669996 | 0.877258520667688 | NA | 3.38285107335066 | 6.16112717073071 | 16.2656680467373 | 5.18941236643882 | 10.0174733905098 | 7.72766639038271 |
| YDR427W | 1957.07591425284 | -0.825219816648449 | 0.430076902451 | -1.91877269377997 | 0.0550131070460916 | 0.265865137443021 | 2379.49744499485 | 1086.8228329169 | 768.240013899749 | 2610.27442031873 | 2295.11445902569 | 2602.50631436111 |
| YDR428C | 1092.94806521437 | 0.482016307026631 | 0.265362116513891 | 1.81644732623844 | 0.0693017810409986 | 0.30095091833635 | 1094.0140371216 | 1356.6802029949 | 1371.3209368634 | 885.313749714462 | 991.729865660471 | 858.629598931413 |
| YDR429C | 2230.1794463884 | 0.46249411733588 | 0.261551549022179 | 1.76827137543223 | 0.0770155468658693 | 0.317337178053775 | 3003.97175313538 | 2378.19508790205 | 2371.03391912056 | 1912.81739826935 | 1771.97973752129 | 1943.07878238179 |
| YDR430C | 883.913262158357 | -0.162102233700433 | 0.271036126176355 | -0.598083495315891 | 0.549784222382171 | 0.803335259270233 | 980.350241057021 | 730.709682448662 | 790.761708118308 | 934.094225958987 | 980.599339671016 | 886.964375696149 |
| YDR431W | 6.65253938375671 | -0.250967774182559 | 0.819326725887841 | -0.306309761726135 | 0.759368807426941 | NA | 4.73599150269092 | 4.92890173658457 | 8.75843664055088 | 6.22729483972658 | 6.67831559367321 | 8.58629598931413 |
| YDR432W | 1690.9766401707 | 2.02625466235345 | 0.487869992472571 | 4.1532676606819 | 3.27760982086326e-05 | 0.00241534631414385 | 5115.54739312087 | 1487.29609901439 | 1542.73605397132 | 595.744539667176 | 667.831559367321 | 736.704195883152 |
| YDR433W | 229.666839755838 | 2.43916208940999 | 0.30575354696168 | 7.97754306907741 | 1.49274824344395e-15 | 1.61996520030551e-12 | 340.314817979076 | 405.402167834081 | 417.902548277713 | 81.9927153897333 | 64.557050738841 | 67.8317383155816 |
| YDR434W | 1695.59209139481 | 0.563707661644409 | 0.304312605560741 | 1.85239668467132 | 0.0639688810341034 | 0.288745036193243 | 1536.49095751587 | 2259.90144622403 | 2272.18870560577 | 1462.37640486246 | 1512.63848196698 | 1129.95655219374 |
| YDR435C | 1219.23574485485 | 0.201580992343158 | 0.330337023173348 | 0.610228276584594 | 0.541710600983307 | 0.798861248305202 | 889.013262076553 | 1429.38150360953 | 1595.28667381462 | 1066.94318253982 | 1326.75869794308 | 1008.03114914548 |
| YDR436W | 461.139526707133 | 0.617388041764839 | 0.309283412006719 | 1.9961886664372 | 0.045913390626968 | 0.23995373849736 | 705.662733900948 | 500.283526263334 | 467.95075765229 | 340.425451238386 | 418.507777203521 | 334.00691398432 |
| YDR437W | 114.011926684946 | 0.146609629425185 | 0.325578445870114 | 0.450305084027809 | 0.652490473643304 | 0.862003656689277 | 99.4558215565094 | 142.938150360953 | 117.613292030255 | 104.826129802064 | 110.192207295608 | 109.045959064289 |
| YDR438W | 723.897708613983 | 0.799476484198828 | 0.367415912627754 | 2.175944091482 | 0.0295594413398573 | 0.192420915992638 | 523.665346154682 | 1153.36300636079 | 1082.29252772522 | 504.410882017853 | 568.769878061168 | 510.88461136419 |
| YDR439W | 27.8278801780606 | -0.044336506437764 | 0.546477963966374 | -0.0811313709997869 | 0.935337477464794 | 0.98165448197831 | 45.3302043828988 | 17.251156078046 | 18.7680785154662 | 24.9091793589063 | 28.9393675725839 | 31.7692951604623 |
| YDR440W | 105.947281378521 | 1.10097765225089 | 0.330847895102056 | 3.32774567573198 | 0.00087551754023372 | 0.0236266423533494 | 161.700281306161 | 152.795953834122 | 118.864497264619 | 65.3865958171291 | 65.6701033377865 | 71.2662567113072 |
| YDR441C | 3289.15569354007 | 0.648160656440229 | 0.389818660636029 | 1.66272352222105 | 0.0963677914622378 | 0.356047751461037 | 2022.94494186369 | 4976.95852851627 | 5048.6131206604 | 2599.89559558585 | 2505.4814002264 | 2581.04057438783 |
| YDR443C | 407.240942310935 | 0.0906089398035331 | 0.262764093609373 | 0.344829990121226 | 0.730222185489935 | 0.899838143479963 | 386.321592576645 | 432.511127385296 | 441.675447730637 | 374.675572856883 | 410.716409010902 | 397.545504305244 |
| YDR444W | 200.83933609308 | 0.796215778029292 | 0.567376964000325 | 1.40332764378646 | 0.160519158508274 | 0.46177102478427 | 517.576214222651 | 94.881358429253 | 151.395833358094 | 156.720253466452 | 121.322733285063 | 163.139623796968 |
| YDR445C | 3.5686295782211 | 0.0693204926219939 | 1.31505460410962 | 0.0527130146576146 | 0.957960569246472 | NA | 0 | 6.16112717073071 | 5.00482093745765 | 5.18941236643882 | 3.3391577968366 | 1.71725919786283 |
| YDR447C | 33702.6076410933 | -0.538843917783807 | 0.300435884039995 | -1.79354047372076 | 0.072886544983578 | 0.307826495639681 | 20040.686328744 | 30750.185709117 | 31652.9900189509 | 44517.892926732 | 37256.0965919049 | 37997.7942711107 |
| YDR448W | 95.4547000437499 | -0.0667430099885562 | 0.424276534688184 | -0.157310161019412 | 0.875000412627706 | 0.958285320584837 | 142.079745080728 | 66.5401734438917 | 70.067493124407 | 107.939777221927 | 77.9136819261874 | 108.187329465358 |
| YDR449C | 195.73001433563 | 0.591567485162741 | 0.348470417054111 | 1.69761178054573 | 0.0895810579065023 | 0.343639207809743 | 248.301268783938 | 258.76734117069 | 198.941632263941 | 194.084022504812 | 106.853049498771 | 167.432771791625 |
| YDR450W | 51237.818425133 | -0.460393851657282 | 0.27227367076721 | -1.69092314493719 | 0.0908514821884533 | 0.346194418545876 | 34360.971492452 | 46198.5959770072 | 48834.540297243 | 61964.6973026994 | 58709.0723839812 | 57359.0330974152 |
| YDR451C | 168.107509417149 | -0.267699626495542 | 0.538916223679209 | -0.496736996834021 | 0.619374529194437 | 0.842642554747841 | 293.631473166837 | 71.4690751804763 | 91.337982108602 | 196.159787451387 | 130.227154076628 | 225.819584518962 |
| YDR452W | 348.452958396295 | 0.430041971708564 | 0.397742005953125 | 1.0812083342267 | 0.279604451566454 | 0.604425117564489 | 631.240010287233 | 283.411849853613 | 284.023588200721 | 304.099564673315 | 318.333043298423 | 269.609694064464 |
| YDR453C | 487.129761396074 | -0.38089360294816 | 0.310386829253755 | -1.22715774977927 | 0.219763305850616 | 0.540984788877662 | 305.8097370309 | 467.013439541388 | 497.979683277036 | 534.509473743198 | 587.691772243242 | 529.774462540681 |
| YDR454C | 14424.9901351082 | 0.0746122532781568 | 0.319118067895869 | 0.23380767428845 | 0.815134291471845 | 0.93405541082149 | 9923.93190878149 | 15979.4994300072 | 18491.5621586716 | 14001.0345646519 | 14050.0629564895 | 14103.8497920474 |
| YDR455C | 1.81177717285539 | 1.9818508429938 | 2.14176152838725 | 0.925336839198032 | 0.354790721842905 | NA | 0 | 7.39335260487685 | 1.25120523436441 | 0 | 2.22610519789107 | 0 |
| YDR456W | 3665.90745968707 | 0.385743780429075 | 0.348505457692256 | 1.10685147654044 | 0.26835814798271 | 0.594800638350329 | 2513.45834749954 | 4893.16719899433 | 5053.61794159786 | 3218.47354966536 | 3454.91526712694 | 2861.8124532384 |
| YDR457W | 3841.4990319065 | 0.529350244376634 | 0.256529207266566 | 2.06350867418606 | 0.0390643245275172 | 0.22284687967803 | 3909.22270036402 | 4967.1007250431 | 4739.56542777239 | 3151.01118890165 | 3284.61821948827 | 2997.47592986956 |
| YDR458C | 82.9040215505109 | 0.907633295429502 | 0.335487928988985 | 2.70541267509897 | 0.00682195819839763 | 0.0842906805496992 | 98.7792513418392 | 109.668063639007 | 116.36208679589 | 60.1971834506903 | 52.3134721504401 | 60.1040719251989 |
| YDR459C | 800.353580392746 | 0.528976195322847 | 0.359723175424542 | 1.47050907881749 | 0.141423928632966 | 0.433549195617219 | 554.111005814838 | 1145.96965375591 | 1137.34555803725 | 709.91161172883 | 609.952824222153 | 644.830828797491 |
| YDR460W | 68.4472264418109 | -0.432771446332081 | 0.358478656539576 | -1.20724466697588 | 0.227337933203989 | 0.550365795198408 | 64.9507406083327 | 55.4501445365764 | 53.8018250776697 | 73.6896556034312 | 92.3833657124794 | 70.4076271123758 |
| YDR461C-A | 72.0626055161237 | -1.33982229487335 | 0.460159990725033 | -2.91164447557101 | 0.00359531595922501 | 0.0570807968717871 | 61.567889534982 | 24.6445086829228 | 35.0337465622035 | 106.90189474864 | 74.5745241293508 | 129.653069438643 |
| YDR461W | 3650.37540083818 | -1.53071071950184 | 0.342067481609938 | -4.47487937847107 | 7.64544619270244e-06 | 0.000799018631193702 | 1167.76019052065 | 2278.38482773622 | 2187.10674966899 | 5899.32397816765 | 5521.8539433688 | 4847.82271556676 |
| YDR462W | 387.472966459714 | -1.2839763207871 | 0.456346052561027 | -2.81360233879834 | 0.00489897896445607 | 0.0705747646308108 | 396.470145796697 | 124.45476884876 | 153.898243826823 | 567.721712888407 | 486.403985739199 | 595.8889416584 |
| YDR463W | 13.5301649862739 | 0.196808665167395 | 0.898034436398952 | 0.219154920112621 | 0.826529370379585 | 0.937978507797441 | 35.1816511628469 | 3.69667630243843 | 3.75361570309323 | 16.6061195726042 | 13.3566311873464 | 8.58629598931413 |
| YDR464W | 37.8470537066257 | -0.460450661806174 | 0.467010491491687 | -0.985953571054561 | 0.324155896814377 | 0.640660728524929 | 43.9770639535586 | 32.0378612877997 | 18.7680785154662 | 39.439533984935 | 45.6351565567669 | 47.2246279412277 |
| YDR465C | 2529.17283810248 | 0.779812732306407 | 0.245200802355037 | 3.18030253089173 | 0.00147121370972574 | 0.0327715644637421 | 3008.70774463808 | 3182.83829639949 | 3398.27341653374 | 1974.05246419333 | 1832.08457786435 | 1779.08052898589 |
| YDR466W | 68.0412805377911 | -0.644181005005831 | 0.604486808675074 | -1.06566594301331 | 0.286574678218257 | 0.610753467985257 | 108.251234347221 | 18.4833815121921 | 31.2801308591103 | 69.5381257102802 | 76.8006293272419 | 103.894181470701 |
| YDR467C | 34.3056539351833 | 0.430765017253043 | 0.439709387493235 | 0.979658450570767 | 0.327254742152494 | 0.643568656507667 | 36.5347915921871 | 45.5923410634073 | 36.2849517965679 | 34.2501216184962 | 22.2610519789107 | 30.9106655615309 |
| YDR468C | 178.880316066099 | 0.0344390884960054 | 0.309464401299344 | 0.111286107065648 | 0.911389471926557 | 0.974551772807771 | 177.261396243575 | 186.066040556067 | 180.173553748475 | 156.720253466452 | 155.827363852375 | 217.233288529647 |
| YDR469W | 57.6906604321136 | -0.207271674694758 | 0.453462435177806 | -0.457086758715714 | 0.647608687301447 | 0.859054305981825 | 82.5415661897561 | 38.1989884585304 | 38.7873622652968 | 73.6896556034312 | 54.5395773483312 | 58.3868127273361 |
| YDR470C | 592.874413272148 | -0.18959313059824 | 0.282359248293583 | -0.671460671977391 | 0.50192710519082 | 0.777149832544209 | 458.038035331679 | 589.003757521856 | 615.59297530729 | 623.767366445946 | 698.997032137796 | 571.847312888321 |
| YDR471W | 26775.9634788862 | -0.881865109453782 | 0.253654039529834 | -3.47664524124426 | 0.000507729143224027 | 0.0164882887867328 | 17028.5957330325 | 19288.0247206896 | 20198.2060983447 | 38560.4475300603 | 32327.4996837741 | 33253.0071074157 |
| YDR472W | 1402.81641522239 | 0.0707565409694291 | 0.26346524873203 | 0.268561190934883 | 0.788267380503691 | 0.926336024233623 | 1244.88919499304 | 1507.01170596073 | 1560.25292725242 | 1408.4065162515 | 1454.75974682181 | 1241.57840005482 |
| YDR473C | 40.6976752411901 | -0.523186996260325 | 0.455945555450721 | -1.14747690816535 | 0.251184571501704 | 0.574993594978811 | 21.6502468694442 | 45.5923410634073 | 33.7825413278391 | 55.0077710842515 | 40.0698935620392 | 48.0832575401591 |
| YDR475C | 630.526696774868 | 0.749617493734241 | 0.36796981882839 | 2.03717113572252 | 0.0416328952879648 | 0.226830220014428 | 1102.13287969764 | 698.671821160863 | 570.549586870172 | 346.652746078113 | 565.430720264332 | 499.722426578082 |
| YDR476C | 2494.31239035718 | 0.579762317526167 | 0.397247155135515 | 1.45944989166351 | 0.144441322622001 | 0.437901963472622 | 1468.83393604886 | 3881.51011756035 | 3617.23433254751 | 1893.09763127688 | 2081.40836002815 | 2023.78996468134 |
| YDR477W | 341.657383483003 | 0.535183815151246 | 0.484852030685811 | 1.10380854627801 | 0.26967619679317 | 0.595604728404763 | 749.639797854506 | 216.871676409721 | 245.236225935425 | 287.49344510071 | 279.376202335329 | 271.326953262326 |
| YDR478W | 72.0761227878855 | 0.243650141798896 | 0.397161930507281 | 0.613478088113052 | 0.539560266901225 | 0.798710611079734 | 103.51524284453 | 71.4690751804763 | 58.8066460151273 | 76.8033030232945 | 52.3134721504401 | 69.5489975134444 |
| YDR479C | 787.13955421655 | 0.0888395286090742 | 0.287587759289023 | 0.3089127605038 | 0.757387889762565 | 0.912676224393128 | 850.448759840356 | 793.553179590116 | 789.510502883944 | 675.661490110334 | 951.659972098432 | 662.003420776119 |
| YDR480W | 41.3183113001469 | 0.0494845967531841 | 0.751202150233235 | 0.0658738752782058 | 0.947478239901987 | 0.985486971897494 | 96.7495406978288 | 22.1800578146306 | 6.25602617182206 | 42.5531814047983 | 38.9568409630937 | 41.2142207487078 |
| YDR481C | 3261.21014743609 | 0.571176964678493 | 0.270911229074171 | 2.10835470582179 | 0.0350003192305056 | 0.209782935283573 | 4280.65974821792 | 3282.64855656532 | 4131.47968387129 | 2458.74357921871 | 2964.05907099196 | 2449.67024575132 |
| YDR482C | 105.961998890306 | 0.662187238710146 | 0.443302800634624 | 1.49375830191502 | 0.135238803873818 | 0.422244782545739 | 206.35391547439 | 89.9524566926684 | 92.5891873429664 | 76.8033030232945 | 67.8962085356776 | 102.176922272838 |
| YDR483W | 9795.18023263567 | 0.440882366519217 | 0.270030368190612 | 1.63271401462521 | 0.102529158512115 | 0.366753015804682 | 9327.87354965711 | 11693.8193700469 | 12819.8488312978 | 8338.34779039389 | 8973.4300526989 | 7617.76180171949 |
| YDR484W | 75.0686259420846 | -0.12953938441402 | 0.387529347948528 | -0.334269869107373 | 0.738175925003023 | 0.90301928216742 | 94.0432598391483 | 66.5401734438917 | 53.8018250776697 | 83.0305978630211 | 64.557050738841 | 88.4388486899355 |
| YDR485C | 96.2339071936146 | -0.0729284825459566 | 0.321701045930082 | -0.226696442142768 | 0.820659793859745 | 0.935571696768309 | 89.3072683364574 | 104.739161902422 | 87.5843664055088 | 103.788247328776 | 92.3833657124794 | 99.6010334760439 |
| YDR486C | 156.505987614348 | -0.371894310957243 | 0.32699342895852 | -1.13731432506682 | 0.255406908388865 | 0.580727416700632 | 155.61114937413 | 149.099277531683 | 103.850034452246 | 174.364255512344 | 198.123362612305 | 157.98784620338 |
| YDR487C | 9774.63143472202 | 0.0779841208522678 | 0.312360157212539 | 0.249660909215144 | 0.802849590934875 | 0.930747663909662 | 6898.30990877666 | 11009.9342540958 | 12209.2606769279 | 9073.16858148163 | 9641.26161206622 | 9815.85357498391 |
| YDR488C | 342.260365384379 | 1.06765608573101 | 0.310302924336667 | 3.44068973250358 | 0.000580233469760189 | 0.0182360535584058 | 520.959065296001 | 415.25997130725 | 454.187500074281 | 236.63720390961 | 168.070942440776 | 258.447509278355 |
| YDR489W | 380.045617933469 | 0.0273424064694084 | 0.257495412379952 | 0.106185994603519 | 0.915434782806345 | 0.976237315690329 | 377.526179785934 | 385.686560887743 | 387.873622652968 | 380.902867696609 | 366.194305053081 | 382.090171524479 |
| YDR490C | 110.589415346813 | 0.912843928098882 | 0.544381055033455 | 1.67684734738387 | 0.0935723363891299 | 0.351079497104908 | 274.687507156073 | 61.6112717073071 | 96.3428030460597 | 84.0684803363089 | 60.1048403430589 | 86.7215894920727 |
| YDR491C | 2.41665050717437 | -1.89131021990443 | 1.4911833789566 | -1.26832839380747 | 0.20468070132056 | NA | 0.676570214670132 | 2.46445086829228 | 0 | 5.18941236643882 | 4.45221039578214 | 1.71725919786283 |
| YDR492W | 6712.18366290485 | 0.711790110977946 | 0.353202705795876 | 2.01524535145919 | 0.0438789477986657 | 0.234941605944134 | 5012.03215027634 | 10069.7462478423 | 9924.55991897851 | 5110.53329846895 | 5729.99477937161 | 4426.23558249143 |
| YDR493W | 551.180721328646 | -1.28829936552357 | 0.274616588511675 | -4.69126563877913 | 2.71520168913275e-06 | 0.000371594745455596 | 337.608537120396 | 266.160693775567 | 356.593491793857 | 792.942209591851 | 753.536609486127 | 800.242786204077 |
[truncated: 1,160,342 more chars]
