# Supplementary material for: Growth-phase-dependent control of rRNA synthesis in Saccharomyces cerevisiae
Source: mSphere. 2024 Oct 3;9(10):e00493-24. doi: 10.1128/msphere.00493-24 (PMC11520348; doi:10.1128/msphere.00493-24)
Supplement: Table S3 — Differential gene expression data set. [file msphere.00493-24-s0004.docx]

**Supplemental Table 3. mRNA-seq statistics for two-way comparison between Early and Late log subphases.** Table includes statistics output from DESeq as described in column headers. Statistics represent gene expression changes in Late log when compared to Early log. Additionally, the individual sample’s normalized counts were appended on the table and column headers describe which individual sample the respective column values refer to.

|  | baseMean | log2FoldChange | lfcSE | stat | pvalue | padj | Early_RNAseq_A_i502_i706_counts.txt | Early_RNAseq_B_i502_i705_counts.txt | Early_RNAseq_C_i504_i709_counts.txt | Late_RNAseq_A_i504_i712_counts.txt | Late_RNAseq_B_i504_i710_counts.txt | Late_RNAseq_C_i504_i711_counts.txt |
| --- | --- | --- | --- | --- | --- | --- | --- | --- | --- | --- | --- | --- |
| HRA1 | 5.7190265059775 | 0.0840345220407371 | 0.889709257841049 | 0.0944516664293834 | 0.924750375332001 | 0.940114566520902 | 7.60044013874454 | 2.46423245740569 | 6.61729749168058 | 8.0021902440675 | 2.85396098699187 | 6.77603771697479 |
| ICR1 | 348.269039365922 | 0.45864461351761 | 0.21362315805475 | 2.14697983914301 | 0.0317948866875859 | 0.0464492982687816 | 265.255360842185 | 289.958019154736 | 324.247577092349 | 356.097465861004 | 499.443172723578 | 354.612640521681 |
| IRT1 | 978.327782773631 | 0.0187086089613201 | 0.16962499316079 | 0.110293940843882 | 0.912176262454435 | 0.929516926673915 | 965.255897620557 | 908.480365963563 | 1041.12147202441 | 924.252973189797 | 1160.1351412122 | 870.72084663126 |
| LSR1 | 201.259349137795 | 0.280584986936323 | 0.482125183455347 | 0.581975380181131 | 0.560583264331344 | 0.622166846757888 | 298.69729745266 | 178.246147752345 | 68.378740747366 | 220.060231711856 | 214.04707402439 | 228.126603138151 |
| NME1 | 151.610614300282 | 0.853228659148198 | 0.410156448042536 | 2.08025172643321 | 0.0375024500233263 | 0.0541980594588446 | 134.527790455778 | 140.461250072124 | 49.2621035491777 | 216.059136589823 | 189.788405634959 | 179.564999499832 |
| PWR1 | 2.55491916314883 | -0.525320064005248 | 1.36306998060073 | -0.385394786387805 | 0.699944944694991 | 0.748778312929525 | 2.28013204162336 | 1.64282163827046 | 5.14678693797379 | 4.00109512203375 | 0 | 2.2586792389916 |
| Q0020 | 26.5008764940609 | -1.13547214314708 | 0.727687957145057 | -1.56038330990372 | 0.118669326545901 | 0.156695694620666 | 64.6037411793286 | 22.9995029357864 | 21.3224030287486 | 13.3369837401125 | 29.9665903634147 | 6.77603771697479 |
| Q0045 | 209.870652641682 | 0.905527085150298 | 0.36260705151343 | 2.49726827255801 | 0.0125154233615726 | 0.0194617683824407 | 207.492015787726 | 147.032536625206 | 83.8191015612874 | 218.726533337845 | 309.654767088618 | 292.498961449412 |
| Q0050 | 5.81539320100442 | -0.812285856640156 | 0.870420456171376 | -0.93321089926249 | 0.350711088352784 | 0.414651570001214 | 11.4006602081168 | 5.7498757339466 | 5.14678693797379 | 2.6673967480225 | 4.28094148048781 | 5.64669809747899 |
| Q0055 | 16.5377039283778 | -2.42296130366301 | 0.784984195427872 | -3.0866370530458 | 0.00202434602368866 | 0.0035248075999046 | 38.7622447075972 | 34.4992544036796 | 10.2935738759476 | 8.0021902440675 | 4.28094148048781 | 3.38801885848739 |
| Q0060 | 3.51686804943664 | -0.315468846451383 | 1.17596189546096 | -0.268264514070605 | 0.78849571906868 | 0.825800619066601 | 6.84039612487009 | 3.28564327654092 | 1.4705105537068 | 2.6673967480225 | 5.70792197398374 | 1.1293396194958 |
| Q0065 | 8.14572671828766 | -1.67832681000345 | 0.845084867234683 | -1.98598611225323 | 0.0470348659908363 | 0.0667545165900578 | 19.0011003468614 | 12.3211622870284 | 5.88204221482719 | 4.00109512203375 | 4.28094148048781 | 3.38801885848739 |
| Q0070 | 6.48686619385354 | -1.11882092133789 | 1.17323708778325 | -0.953618780882408 | 0.340276645584897 | 0.403996764280366 | 10.6406161942424 | 13.9639839252989 | 2.20576583056019 | 5.334793496045 | 0 | 6.77603771697479 |
| Q0075 | 64.6653397533137 | -0.859427943315113 | 0.598030680985195 | -1.43709674209238 | 0.150690506819722 | 0.195241435723675 | 123.127130247662 | 104.319174030174 | 22.7929135824553 | 46.6794430903938 | 41.3824343113821 | 49.6909432578151 |
| Q0085 | 17.4526346917125 | 1.70049757476742 | 0.555648187689957 | 3.06038535253223 | 0.00221052373501692 | 0.00383149570959907 | 12.1607042219913 | 7.39269737221706 | 5.14678693797379 | 30.6750626022588 | 19.9777269089431 | 29.3628301068907 |
| Q0105 | 1.33932331481236 | -1.09172955752137 | 1.74085362834261 | -0.627123119225568 | 0.530578533530322 | 0.592720008349581 | 2.28013204162336 | 2.46423245740569 | 0.735255276853398 | 0 | 1.42698049349594 | 1.1293396194958 |
| Q0120 | 1.73431172468804 | 0.71646126142958 | 1.42725438865199 | 0.501985677624198 | 0.615677595739174 | 0.673176818267748 | 0.760044013874454 | 2.46423245740569 | 0.735255276853398 | 1.33369837401125 | 2.85396098699187 | 2.2586792389916 |
| Q0130 | 99.3727056876553 | -0.38685491416008 | 0.512838643081283 | -0.754340413654758 | 0.450644841172616 | 0.515681446691176 | 164.169506996882 | 134.711374338178 | 38.9685296732301 | 84.0229975627088 | 94.1807125707318 | 80.1831129842017 |
| Q0140 | 3.71485732620496 | -0.598419132610914 | 1.41058096095785 | -0.424235934819763 | 0.671393756223334 | 0.723966733643315 | 6.08035211099563 | 7.39269737221706 | 0 | 4.00109512203375 | 1.42698049349594 | 3.38801885848739 |
| Q0158 | 120.758165812404 | -0.844915396973931 | 0.581727187930771 | -1.4524254917143 | 0.146383332513908 | 0.189926444225887 | 281.216285133548 | 128.140087785096 | 55.8794010408583 | 64.01752195254 | 124.147302934146 | 71.1483960282353 |
| Q0160 | 100.066655382567 | -0.0501265843259003 | 0.263696083082058 | -0.190092259771268 | 0.849236835305593 | 0.877932756422071 | 123.887174261536 | 96.1050658388218 | 85.2896121149942 | 86.6903943107313 | 111.304478492683 | 97.1232072766386 |
| Q0250 | 1.4742822887531 | -1.2322696627935 | 1.96009296664938 | -0.628679192140545 | 0.529559103231421 | 0.591989028534815 | 3.80022006937227 | 1.64282163827046 | 0.735255276853398 | 2.6673967480225 | 0 | 0 |
| Q0275 | 2.97769116017519 | 0.917174217650237 | 1.28427400811368 | 0.714157735697984 | 0.475129647977615 | 0.53922850337407 | 3.80022006937227 | 1.64282163827046 | 0.735255276853398 | 8.0021902440675 | 1.42698049349594 | 2.2586792389916 |
| RDN5-1 | 77.9010604738244 | 2.63426093265736 | 0.368582342491911 | 7.14700795173109 | 8.86897422177584e-13 | 4.11474726769943e-12 | 22.8013204162336 | 12.3211622870284 | 29.4102110741359 | 116.031758538979 | 156.967854284553 | 129.874056242017 |
| RME2 | 1658.92728137888 | 0.486612626238898 | 0.166282161885421 | 2.92642710872502 | 0.00342879844715895 | 0.00579145487715441 | 1417.48208587586 | 1453.07573905022 | 1274.19739478694 | 1760.48185369485 | 2283.1687895935 | 1765.15782527193 |
| RME3 | 258.428785169295 | 1.02052668992166 | 0.150546877387084 | 6.77879679495247 | 1.21180824690149e-11 | 5.07083115445357e-11 | 163.409462983008 | 169.210628741857 | 179.402287552229 | 349.428973990948 | 336.767396465041 | 352.353961282689 |
| RNA170 | 24.4414983190298 | 0.966832188105258 | 0.540398416913974 | 1.78910995636608 | 0.0735971090266471 | 0.101133654480261 | 14.4408362636146 | 18.071038020975 | 16.9108713676282 | 22.6728723581913 | 54.2252587528456 | 20.3281131509244 |
| RPR1 | 204.147905366764 | 0.216188412560046 | 0.526286332713063 | 0.410780974390065 | 0.681233145143075 | 0.732627786336923 | 266.015404856059 | 245.601834921433 | 55.1441457640049 | 192.05256585762 | 231.170839946342 | 234.902640855126 |
| RUF20 | 63.4486230490524 | -0.105493997785459 | 0.349603579933591 | -0.301753196593405 | 0.76284021228891 | 0.803472169033106 | 77.5244894151943 | 72.2841520839002 | 47.7915929954709 | 73.3534105706188 | 44.236395298374 | 65.5016979307563 |
| RUF21 | 2.88556960401644 | 2.72609999060781 | 1.35468294100866 | 2.01235278609032 | 0.0441827617239364 | 0.0630238382541129 | 1.52008802774891 | 0 | 0.735255276853398 | 4.00109512203375 | 4.28094148048781 | 6.77603771697479 |
| RUF23 | 14.3792051547308 | 0.295636588662811 | 0.690578296653895 | 0.428100028766149 | 0.668578295844322 | 0.721290556694323 | 12.1607042219913 | 5.7498757339466 | 20.5871477518952 | 17.3380788621463 | 21.404707402439 | 9.03471695596638 |
| SCR1 | 918.5589140526 | 1.09018618065437 | 0.446266276177743 | 2.44290514172791 | 0.0145695662060388 | 0.0224674765602395 | 811.727006817917 | 735.16268312603 | 214.694540841192 | 1227.00250409035 | 1141.58439479675 | 1381.18235464336 |
| snR10 | 79.9299736110462 | 2.03592327225373 | 0.333673589687272 | 6.1015415519156 | 1.05050322041257e-09 | 3.68898965219054e-09 | 23.5613644301081 | 44.3561842333024 | 26.4691899667223 | 121.366552035024 | 117.012400466667 | 146.814150534454 |
| snR11 | 320.666520213821 | 1.43585193531763 | 0.221770990666332 | 6.47448041334658 | 9.51387509800575e-11 | 3.67522298306523e-10 | 185.450739385367 | 201.245650688131 | 133.081205110465 | 436.119368301679 | 445.217913970732 | 522.884243826554 |
| snR128 | 129.558974794043 | 2.4113767594851 | 0.408185871107767 | 5.90754587595331 | 3.47241648440515e-09 | 1.15261480644073e-08 | 51.6829929434629 | 50.106059967249 | 21.3224030287486 | 162.711201629373 | 288.250059686179 | 203.281131509244 |
| snR13 | 108.572207900698 | 2.35261752831655 | 0.326724589756316 | 7.20061361182281 | 5.99421875824351e-13 | 2.83164623932756e-12 | 39.5222887214716 | 36.1420760419501 | 30.8807216278427 | 142.705726019204 | 249.721586361789 | 152.460848631933 |
| snR14 | 1.71685097306408 | 1.76929943281756 | 1.49185783351257 | 1.18597053490798 | 0.23563393588028 | 0.292389842762483 | 0.760044013874454 | 0.821410819135229 | 0.735255276853398 | 4.00109512203375 | 2.85396098699187 | 1.1293396194958 |
| snR161 | 30.1810583582383 | 2.47570656989016 | 0.483996698546077 | 5.11513111004098 | 3.13523105773746e-07 | 8.56936731583867e-07 | 5.32030809712118 | 9.03551901048752 | 13.2345949833612 | 40.0109512203375 | 51.3712977658537 | 62.1136790722689 |
| snR17a | 255.052614440696 | 1.70256164191808 | 0.298192462717655 | 5.70960656215567 | 1.13237638620166e-08 | 3.56670157393324e-08 | 155.048978830389 | 130.604320242501 | 74.2607829621932 | 380.104036593206 | 372.441908802439 | 417.855659213445 |
| snR17b | 165.153894391352 | 0.670254610387297 | 0.347376141700942 | 1.92947796329755 | 0.0536715532260363 | 0.0753003881081703 | 164.929551010757 | 147.032536625206 | 70.5845065779262 | 189.385169109598 | 211.193113037399 | 207.798489987227 |
| snR18 | 49.8269764731778 | 1.2869902759929 | 0.337996873022609 | 3.80769876503211 | 0.00014026598828879 | 0.000283946691648281 | 24.3214084439825 | 38.6063084993558 | 24.2634241361621 | 65.3512203265513 | 68.4950636878049 | 77.92443374521 |
| snR189 | 77.8736734183893 | 2.23156843497072 | 0.296917691354118 | 7.51578130893268 | 5.65720516989879e-14 | 2.95908328483577e-13 | 28.8816725272293 | 32.8564327654092 | 20.5871477518952 | 134.703535775136 | 111.304478492683 | 138.908773197983 |
| snR19 | 131.336826683841 | 0.801696603159149 | 0.314658128431999 | 2.54783376216644 | 0.0108394109853276 | 0.0170146223743549 | 116.286734122792 | 111.711871402391 | 59.5556774251253 | 161.377503255361 | 158.394834778049 | 180.694339119328 |
| snR190 | 68.3361312662951 | 1.90597321491798 | 0.411274398454086 | 4.63431038275716 | 3.58129323790935e-06 | 8.77200450947131e-06 | 36.4821126659738 | 36.1420760419501 | 13.9698502602146 | 102.694774798866 | 99.8886345447155 | 120.83933928605 |
| snR191 | 57.3996511378341 | 1.6234923738257 | 0.405130252991508 | 4.00733433713659 | 6.14079055955838e-05 | 0.000130075661558771 | 41.802420763095 | 21.356681297516 | 21.3224030287486 | 86.6903943107313 | 67.068083194309 | 106.157924232605 |
| snR24 | 57.5635435963711 | 0.367221600322951 | 0.358819429298676 | 1.02341615402682 | 0.306111124769907 | 0.369108896682955 | 58.523389068333 | 60.784400616007 | 31.6159769046961 | 70.6860138225963 | 62.7871417138212 | 60.9843394527731 |
| snR3 | 14.0620960471697 | 2.14540214674993 | 0.594555869446147 | 3.60841134870715 | 0.000308077749825825 | 0.000600614839935879 | 6.08035211099563 | 6.57128655308183 | 2.94102110741359 | 21.33917398418 | 27.1126293764228 | 20.3281131509244 |
| snR30 | 780.560530412193 | 1.26416814803825 | 0.204971799824716 | 6.16752230852887 | 6.93683287219913e-10 | 2.4789144908586e-09 | 503.149137184889 | 524.060102608276 | 349.981511782218 | 1038.95103335476 | 1085.93215555041 | 1181.2892419926 |
| snR31 | 1059.21317331007 | 2.11790577659395 | 0.173242505274537 | 12.2250932196905 | 2.28319633049309e-34 | 5.63072676790044e-33 | 438.54539600556 | 430.41926922686 | 321.306555984935 | 1789.8232179231 | 1725.21941663659 | 1649.96518408336 |
| snR32 | 60.1844569554102 | 1.59378393778957 | 0.335235490542187 | 4.75422198053044 | 1.99212065820152e-06 | 5.03738580471541e-06 | 33.441936610476 | 32.0350219462739 | 24.2634241361621 | 78.6882040666638 | 117.012400466667 | 75.6657545062185 |
| snR33 | 509.508255842454 | 1.83968126801753 | 0.312293325198351 | 5.89087604369727 | 3.8415363640241e-09 | 1.27123494168675e-08 | 263.735272814436 | 277.636856867707 | 126.463907618785 | 821.55819839093 | 801.963037344716 | 765.692262018151 |
| snR34 | 78.7802001751741 | 1.87595143352943 | 0.339969023620385 | 5.51800694531555 | 3.42865885445672e-08 | 1.02812211419354e-07 | 32.6818925966015 | 46.8204166907081 | 22.057658305602 | 116.031758538979 | 118.439380960163 | 136.650093958992 |
| snR35 | 165.444396293804 | 1.87286267468384 | 0.269310427516329 | 6.9542894865082 | 3.54343324667783e-12 | 1.55920678683531e-11 | 76.7644454013199 | 87.0695468283343 | 49.2621035491777 | 258.737484558183 | 282.542137712195 | 238.290659713613 |
| snR36 | 88.6995718770717 | 2.31235142726447 | 0.333960037939184 | 6.92403630546228 | 4.38953336279732e-12 | 1.92368333723672e-11 | 32.6818925966015 | 35.3206652228149 | 21.3224030287486 | 189.385169109598 | 127.001263921138 | 126.486037383529 |
| snR37 | 417.65429671579 | 2.0409897358802 | 0.165274079655481 | 12.3491217747798 | 4.92460702625312e-35 | 1.29315794219748e-33 | 155.048978830389 | 162.639342188775 | 172.049734783695 | 594.829474809018 | 744.883817604879 | 676.474432077983 |
| snR38 | 40.8929233579774 | 0.730329110727025 | 0.447009575736858 | 1.63381088542262 | 0.102298565964291 | 0.136693139440542 | 33.441936610476 | 42.7133625950319 | 16.1756160907748 | 49.3468398384163 | 58.5062002333334 | 45.1735847798319 |
| snR39 | 82.3881205562291 | 0.514331172758458 | 0.331538486737925 | 1.55134680687924 | 0.120818595380224 | 0.159299964593549 | 57.7633450544585 | 83.7839035517934 | 61.7614432556855 | 117.36545691299 | 107.023537012195 | 66.6310375502521 |
| snR39B | 88.0317238545688 | 2.42000525651481 | 0.39668932074561 | 6.10050518114836 | 1.05733754746442e-09 | 3.7109801584709e-09 | 32.6818925966015 | 32.8564327654092 | 17.6461266444816 | 114.698060164968 | 208.339152050407 | 121.968678905546 |
| snR4 | 609.430179809035 | 1.48217764668425 | 0.236352262183294 | 6.27105335482174 | 3.58613696530087e-10 | 1.32760755461995e-09 | 335.179410118634 | 395.098604004045 | 233.811178039381 | 846.898467497144 | 997.459364953659 | 848.134054241344 |
| snR40 | 59.2089575351452 | 2.07391751905574 | 0.337024568053797 | 6.15360930816383 | 7.57391319211124e-10 | 2.6902738753578e-09 | 25.081452457857 | 24.6423245740569 | 18.381381921335 | 89.3577910587538 | 119.866361453659 | 77.92443374521 |
| snR41 | 185.104195849132 | 0.766039655082109 | 0.197656410134687 | 3.87561250636959 | 0.00010635682349622 | 0.000218093694972015 | 145.928450663895 | 133.889963519042 | 130.875439279905 | 232.063517077958 | 266.84535228374 | 201.022452270252 |
| snR42 | 157.142393991885 | 1.38181063026327 | 0.38470589732619 | 3.59186235476823 | 0.000328323259920065 | 0.000636623217889849 | 114.006602081168 | 104.319174030174 | 43.3800613343505 | 245.40050081807 | 194.069347115447 | 241.678678572101 |
| snR43 | 210.218028378411 | 1.37138906495408 | 0.284801593459232 | 4.81524365189477 | 1.47020390098879e-06 | 3.75570795660233e-06 | 127.687394330908 | 145.389714986936 | 78.6723146233136 | 278.742960168351 | 349.610220906504 | 281.205565254454 |
| snR44 | 1218.58267124994 | 1.37796185203462 | 0.175719745875205 | 7.84181564326437 | 4.44078020297741e-15 | 2.54667554345813e-14 | 750.163441694086 | 738.448326402571 | 543.353649594661 | 1721.80460084852 | 1739.48922157155 | 1818.23678738823 |
| snR45 | 95.4739002829837 | 2.30378429658772 | 0.323531263484335 | 7.12074707024185 | 1.07343621235073e-12 | 4.95537884221129e-12 | 30.4017605549782 | 42.7133625950319 | 23.5281688593087 | 138.70463089717 | 194.069347115447 | 143.426131675966 |
| snR46 | 137.282123172959 | 1.64311470245396 | 0.476968901110426 | 3.44490950799653 | 0.000571250601005032 | 0.0010680690107001 | 94.2454577204323 | 82.1410819135229 | 23.5281688593087 | 217.392834963834 | 194.069347115447 | 212.31584846521 |
| snR47 | 62.2607072407554 | 2.10679658782747 | 0.335577142482667 | 6.27812899365245 | 3.42671570263111e-10 | 1.27149187913418e-09 | 25.8414964717314 | 25.4637353931921 | 19.1166371981884 | 77.3545056926525 | 124.147302934146 | 101.640565754622 |
| snR48 | 61.1072485884104 | 2.0067150128658 | 0.302191601369667 | 6.64053866411399 | 3.12538834755034e-11 | 1.26379481435234e-10 | 28.1216285133548 | 22.1780921166512 | 22.7929135824553 | 82.6892991886975 | 101.315615038211 | 109.545943091092 |
| snR49 | 349.032211633697 | 2.43780017869291 | 0.193054079444144 | 12.6275507138312 | 1.48839229392286e-36 | 4.36819566442701e-35 | 119.326910178289 | 116.640336317203 | 90.436399052968 | 553.484825214669 | 656.41102700813 | 557.893772030924 |
| snR5 | 343.614383479924 | 2.06792843343506 | 0.154894936249162 | 13.3505231578947 | 1.1761996157007e-40 | 4.68026423769001e-39 | 119.326910178289 | 137.175606795583 | 140.433757878999 | 565.48811058077 | 565.084275424391 | 534.177640021512 |
| snR50 | 45.9175425941119 | 1.04528547333348 | 0.404010819637801 | 2.58727099009523 | 0.0096739477503328 | 0.0152850731080776 | 25.081452457857 | 44.3561842333024 | 20.5871477518952 | 61.3501252045175 | 69.9220441813009 | 54.2083017357983 |
| snR51 | 187.223523559939 | 0.94576697413949 | 0.236055168194626 | 4.00655059312115 | 6.16119326915447e-05 | 0.000130431834766616 | 149.728670733267 | 127.31867696596 | 106.612015143743 | 218.726533337845 | 293.957981660163 | 226.997263518655 |
| snR52 | 10.5697550984082 | 3.01088882268337 | 0.763117862973829 | 3.94550955857605 | 7.96304551602488e-05 | 0.000165858423946491 | 1.52008802774891 | 3.28564327654092 | 2.20576583056019 | 24.0065707322025 | 19.9777269089431 | 12.4227358144538 |
| snR53 | 3.9676493417649 | -1.403000440741 | 1.30179325727763 | -1.07774443668193 | 0.281147821832647 | 0.342270236753451 | 9.12052816649345 | 7.39269737221706 | 0.735255276853398 | 4.00109512203375 | 1.42698049349594 | 1.1293396194958 |
| snR54 | 45.905600135085 | 0.565486857976248 | 0.37124030054499 | 1.52323672065263 | 0.127699492217223 | 0.167055043671019 | 43.3225087908439 | 44.3561842333024 | 23.5281688593087 | 50.6805382124275 | 57.0792197398374 | 56.4669809747899 |
| snR55 | 120.972106366983 | 0.808965997561431 | 0.233296241880749 | 3.46754834557061 | 0.00052522925371982 | 0.000987717291860467 | 97.2856337759301 | 83.7839035517934 | 82.3485910075806 | 136.037234149148 | 184.080483660976 | 142.296792056471 |
| snR56 | 49.0933303868557 | 2.69141353095539 | 0.35453125253436 | 7.5914704605472 | 3.16295282185697e-14 | 1.68569531656239e-13 | 9.8805721803679 | 16.4282163827046 | 13.2345949833612 | 88.0240926847425 | 91.3267515837399 | 75.6657545062185 |
| snR57 | 289.504035111244 | 0.899421918440083 | 0.326504799183743 | 2.75469739093766 | 0.00587464417478386 | 0.0095808252747418 | 228.773248176211 | 247.244656559704 | 130.140184003052 | 310.751721144621 | 493.735250749594 | 326.379150034286 |
| snR58 | 5.5395586670593 | 1.89402425534893 | 0.999595775122696 | 1.89479017667561 | 0.0581202051402058 | 0.0810683119439516 | 3.04017605549782 | 3.28564327654092 | 0.735255276853398 | 4.00109512203375 | 14.2698049349594 | 7.90537733647059 |
| snR59 | 15.3189001222741 | 0.282774518979904 | 0.519947522635734 | 0.543852036348697 | 0.586543297020992 | 0.645346874381366 | 10.6406161942424 | 12.3211622870284 | 18.381381921335 | 17.3380788621463 | 18.5507464154472 | 14.6814150534454 |
| snR6 | 3.02077881210022 | 1.90150562736911 | 1.18291138137555 | 1.60747935754742 | 0.107949254080531 | 0.143622330659758 | 0.760044013874454 | 0.821410819135229 | 2.20576583056019 | 6.66849187005625 | 4.28094148048781 | 3.38801885848739 |
| snR60 | 7.32482794163308 | 2.23217923012607 | 0.907178106587319 | 2.4605744053097 | 0.0138714805854494 | 0.0214572914565287 | 5.32030809712118 | 1.64282163827046 | 0.735255276853398 | 14.6706821141238 | 11.4158439479675 | 10.1640565754622 |
| snR61 | 67.1890438466277 | 1.33230736844144 | 0.299088065038467 | 4.4545654747878 | 8.40632729867325e-06 | 1.98918054940513e-05 | 38.0022006937227 | 41.8919517758967 | 34.5569980121097 | 82.6892991886975 | 121.293341947155 | 84.7004714621848 |
| snR62 | 58.8876375613616 | 1.54425762092606 | 0.358263197390792 | 4.31039981826988 | 1.62959642719685e-05 | 3.714907575096e-05 | 34.2019806243504 | 37.7848976802205 | 18.381381921335 | 96.02628292881 | 85.6188296097561 | 81.3124526036975 |
| snR63 | 468.634463987258 | 1.15719417181298 | 0.224248370222564 | 5.16032366551642 | 2.46523264627147e-07 | 6.81564319851523e-07 | 332.899278077011 | 326.100095196686 | 211.753519733779 | 678.852472371726 | 620.736514670732 | 641.464903873613 |
| snR64 | 65.4646012387253 | 3.55655927507731 | 0.412800039714305 | 8.61569508941611 | 6.95185393046898e-18 | 5.25521265652935e-17 | 12.9207482358657 | 9.03551901048752 | 8.82306332224078 | 81.3556008146863 | 132.709185895122 | 147.94349015395 |
| snR65 | 6.18911708221879 | 1.55965096183251 | 0.923424190707075 | 1.68898646746331 | 0.0912220233201802 | 0.123109871671804 | 4.56026408324673 | 4.10705409567614 | 0.735255276853398 | 10.66958699209 | 11.4158439479675 | 5.64669809747899 |
| snR66 | 75.8179332698924 | 1.14849349031557 | 0.320715290219833 | 3.58103752873255 | 0.000342232487211738 | 0.00066141832897954 | 44.0825528047183 | 57.498757339466 | 39.7037849500835 | 93.3588861807875 | 135.563146882114 | 84.7004714621848 |
| snR67 | 144.301615765962 | 0.513599637476602 | 0.495384470723646 | 1.03676975728841 | 0.2998431731352 | 0.362426914080304 | 148.968626719393 | 166.746396284451 | 41.1742955037903 | 156.042709759316 | 154.113893297561 | 198.76377303126 |
| snR68 | 222.976700298455 | 1.84304833886873 | 0.270723374483142 | 6.80786556531157 | 9.90573603929733e-12 | 4.17741248055153e-11 | 117.046778136666 | 109.247638944985 | 65.4377196399524 | 344.094180494903 | 382.430772256911 | 319.603112317311 |
| snR69 | 33.1006184521897 | 1.97387561738251 | 0.445633351410798 | 4.42937139047954 | 9.45081517204144e-06 | 2.2193333528552e-05 | 18.2410563329869 | 14.7853947444341 | 7.35255276853398 | 60.0164268305063 | 48.5173367788618 | 49.6909432578151 |
| snR7-L | 2.31696741541481 | 0.968014690343665 | 1.24508609852739 | 0.77746807348389 | 0.436882663579458 | 0.502236964901872 | 0.760044013874454 | 2.46423245740569 | 1.4705105537068 | 2.6673967480225 | 4.28094148048781 | 2.2586792389916 |
| snR70 | 225.395378703477 | 0.415423573949172 | 0.218656851999607 | 1.89988820450922 | 0.0574477922946696 | 0.0802166589501027 | 231.813424231709 | 185.638845124562 | 161.756160907748 | 244.066802444059 | 289.677040179675 | 239.419999333109 |
| snR71 | 29.2285807059124 | 1.89562221340929 | 0.409777204469856 | 4.62598258939692 | 3.72826618572383e-06 | 9.10792259156488e-06 | 15.2008802774891 | 13.1425731061637 | 8.82306332224078 | 45.3457447163825 | 49.9443172723578 | 42.9149055408403 |
| snR72 | 25.4225906742705 | 1.16059667086441 | 0.548084012254617 | 2.11755250092069 | 0.0342129845131451 | 0.0497545779265155 | 13.6807922497402 | 24.6423245740569 | 8.82306332224078 | 36.0098560983038 | 45.6633757918699 | 23.7161320094118 |
| snR73 | 10.0367349765776 | -1.12690921545422 | 0.821733344849326 | -1.37138065835804 | 0.170256323656332 | 0.218410307601853 | 18.2410563329869 | 18.071038020975 | 5.14678693797379 | 8.0021902440675 | 2.85396098699187 | 7.90537733647059 |
| snR74 | 9.78333852258268 | -0.446475199643979 | 0.770814251480716 | -0.579225408438299 | 0.562437088578043 | 0.62369070892754 | 12.1607042219913 | 17.2496272018398 | 4.41153166112039 | 10.66958699209 | 8.56188296097562 | 5.64669809747899 |
| snR75 | 103.275264421588 | 1.12000173996402 | 0.451042740213154 | 2.48313882501407 | 0.0130230323437212 | 0.0202220224518496 | 83.60484152619 | 83.7839035517934 | 27.9397005204291 | 170.71339187344 | 138.417107869106 | 115.192641188571 |
| snR76 | 211.957705994089 | 1.01761953999012 | 0.28589235838663 | 3.55944994729075 | 0.000371632351088039 | 0.00071525443001692 | 119.326910178289 | 171.674861199263 | 129.404928726198 | 216.059136589823 | 371.014928308943 | 264.265470962017 |
| snR77 | 103.555572313785 | 0.747300644845244 | 0.490197332042558 | 1.52448941680565 | 0.127386498587635 | 0.166679207149365 | 91.965325678809 | 108.42622812585 | 31.6159769046961 | 94.6925845547988 | 172.664639713008 | 121.968678905546 |
| snR78 | 28.5745460644234 | 0.500514434650049 | 0.499915558897414 | 1.00119795381835 | 0.316731115606674 | 0.380159136677445 | 34.9620246382249 | 22.1780921166512 | 13.9698502602146 | 24.0065707322025 | 35.6745123373984 | 40.6562263018487 |
| snR79 | 35.2611282300003 | 2.68115235462842 | 0.524713303551147 | 5.10974724003176 | 3.22590091308834e-07 | 8.79125769844157e-07 | 12.9207482358657 | 7.39269737221706 | 8.08780804538738 | 52.0142365864388 | 92.7537320772358 | 38.3975470628571 |
| snR8 | 346.144360111143 | 2.6396040187083 | 0.249524228716167 | 10.5785479521944 | 3.74720324857366e-26 | 5.26068404117938e-25 | 107.166205956298 | 113.354693040662 | 66.9082301936593 | 602.831665053085 | 653.557066021139 | 533.048300402017 |
| snR80 | 36.7452609447887 | 1.94999895103507 | 0.455850782957788 | 4.27771328675252 | 1.88823006990119e-05 | 4.26281247246053e-05 | 13.6807922497402 | 22.1780921166512 | 9.55831859909418 | 69.352315448585 | 62.7871417138212 | 42.9149055408403 |
| snR81 | 61.8743356788676 | 1.9231978735118 | 0.401958860369234 | 4.78456395200538 | 1.71358932513141e-06 | 4.34833347527478e-06 | 32.6818925966015 | 29.5707894888682 | 15.4403608139214 | 110.696965042934 | 69.9220441813009 | 112.93396194958 |
| snR82 | 308.103819780298 | 1.25193179728368 | 0.283719906717442 | 4.41256241681514 | 1.02154330653569e-05 | 2.38937247969364e-05 | 219.652720009717 | 209.459758879483 | 117.640844296544 | 408.111702447443 | 489.454309269106 | 404.303583779496 |
| snR83 | 599.5484907099 | 2.19062255771591 | 0.181074459845737 | 12.0979102165052 | 1.08333976517833e-33 | 2.58328739593627e-32 | 210.532191843224 | 203.709883145537 | 232.340667485674 | 1005.60857400448 | 824.794725240651 | 1120.30490253983 |
| snR84 | 473.969799015854 | 1.57340988101076 | 0.241754341300039 | 6.50830041996234 | 7.60058018429733e-11 | 2.96614699611026e-10 | 248.534392536947 | 297.350716526953 | 169.843968953135 | 692.189456111839 | 679.242714904065 | 756.657545062185 |
| snR85 | 135.787526739783 | 2.67251861277182 | 0.248090661507547 | 10.7723466757354 | 4.64995758618229e-27 | 6.88575911049733e-26 | 37.2421566798483 | 32.0350219462739 | 41.1742955037903 | 224.06132683389 | 206.912171556911 | 273.300187917983 |
| snR86 | 1996.96239056478 | 1.00796982823637 | 0.167146547681769 | 6.03045556259674 | 1.63498125025371e-09 | 5.62572328336634e-09 | 1472.96529888869 | 1410.36237645519 | 1096.26561778842 | 2835.44274314792 | 2501.49680509838 | 2665.24150201008 |
| snR87 | 76.8850582014778 | 0.409304716276713 | 0.433479534420467 | 0.944230773948592 | 0.345051732528153 | 0.409141780105594 | 83.60484152619 | 82.1410819135229 | 32.3512321815495 | 81.3556008146863 | 108.450517505691 | 73.4070752672269 |
| snR9 | 306.600220270329 | 2.17270524105557 | 0.266062097298359 | 8.16615843864119 | 3.18365087750267e-16 | 2.06491595914823e-15 | 120.086954192164 | 134.711374338178 | 79.407569900167 | 416.11389269151 | 582.208041346342 | 507.073489153613 |
| SRG1 | 1322.7559834892 | 1.73234069736324 | 0.128776558637754 | 13.4522984282899 | 2.98449145840398e-41 | 1.22515263286128e-39 | 664.278468126273 | 613.593881894016 | 558.058755131729 | 2019.21933825303 | 2127.62791580244 | 1953.75754172773 |
| tC(GCA)P1 | 1.3989879482447 | 2.12216674519862 | 2.10476801261589 | 1.00826634217094 | 0.313326616509923 | 0.376828561966135 | 0 | 0.821410819135229 | 0.735255276853398 | 0 | 5.70792197398374 | 1.1293396194958 |
| tD(GUC)D | 2.43106718865475 | 1.39731345393745 | 1.46130438557305 | 0.956209717655434 | 0.338966291349029 | 0.402809704230451 | 0.760044013874454 | 3.28564327654092 | 0 | 4.00109512203375 | 4.28094148048781 | 2.2586792389916 |
| tD(GUC)G2 | 1.59443713065719 | 0.148844994096178 | 1.42933018780076 | 0.104136185862833 | 0.917061274790014 | 0.933761291724966 | 1.52008802774891 | 0.821410819135229 | 2.20576583056019 | 1.33369837401125 | 1.42698049349594 | 2.2586792389916 |
| tD(GUC)N | 10.5145427997733 | 2.03586432707859 | 0.768835598476275 | 2.64798395276362 | 0.00809733687744497 | 0.0129805553601355 | 2.28013204162336 | 4.10705409567614 | 5.88204221482719 | 14.6706821141238 | 27.1126293764228 | 9.03471695596638 |
| tD(GUC)O | 42.6526418587123 | 1.94886266083957 | 0.547679547068591 | 3.55839956279306 | 0.000373121415545755 | 0.000717481619101621 | 15.2008802774891 | 27.9279678505978 | 9.55831859909418 | 62.6838235785288 | 99.8886345447155 | 40.6562263018487 |
| tE(UUC)C | 13.2791046514789 | 2.54651704398882 | 0.653638555363996 | 3.8959100914277 | 9.78306776837189e-05 | 0.000201886660978874 | 5.32030809712118 | 4.10705409567614 | 2.20576583056019 | 20.0054756101688 | 29.9665903634147 | 18.0694339119328 |
| tE(UUC)E2 | 1.36374039295831 | 2.01733007600256 | 2.39049761302079 | 0.84389545716941 | 0.398727826966294 | 0.464275470479598 | 0 | 1.64282163827046 | 0 | 0 | 4.28094148048781 | 2.2586792389916 |
| tE(UUC)G1 | 26.3113418788291 | -0.798298224335607 | 0.602113195737935 | -1.3258274855731 | 0.184896846609581 | 0.235283685915194 | 50.9229489295884 | 36.1420760419501 | 13.2345949833612 | 22.6728723581913 | 15.6967854284553 | 19.1987735314286 |
| tE(UUC)I | 1.23525226513277 | -0.00686588816477103 | 1.98429088020406 | -0.00346012181644708 | 0.997239227733026 | 0.998316658601081 | 1.52008802774891 | 0 | 2.20576583056019 | 0 | 1.42698049349594 | 2.2586792389916 |
| tE(UUC)J | 8.79952002797453 | 1.17448818905821 | 0.679064596072655 | 1.72956769628519 | 0.0837075417644163 | 0.113749657633355 | 6.08035211099563 | 6.57128655308183 | 3.67627638426699 | 10.66958699209 | 9.98886345447155 | 15.8107546729412 |
| tE(UUC)K | 9.47137536693432 | 0.023439473261831 | 0.900736669971302 | 0.0260225591377087 | 0.979239344952414 | 0.983830291080704 | 15.2008802774891 | 11.4997514678932 | 1.4705105537068 | 9.33588861807875 | 11.4158439479675 | 7.90537733647059 |
| tE(UUC)P | 1.58546746622864 | 0.488781465126001 | 1.66087448699896 | 0.294291633083714 | 0.768535065558206 | 0.808464740154445 | 1.52008802774891 | 1.64282163827046 | 0.735255276853398 | 1.33369837401125 | 4.28094148048781 | 0 |
| tF(GAA)D | 2.3824490618721 | 3.06879084752528 | 1.49816286515468 | 2.0483693187846 | 0.0405238254686278 | 0.0581756378905534 | 0.760044013874454 | 0 | 0.735255276853398 | 4.00109512203375 | 4.28094148048781 | 4.51735847798319 |
| tF(GAA)F | 1.31576621848178 | 0.0890135895075194 | 2.55859319557179 | 0.0347900516821419 | 0.972247153438499 | 0.978735843116888 | 3.80022006937227 | 0 | 0 | 2.6673967480225 | 1.42698049349594 | 0 |
| tF(GAA)H1 | 1.5189558913362 | 4.41681587184818 | 2.0522104853515 | 2.15222361613247 | 0.0313797464773379 | 0.0459329802870715 | 0 | 0 | 0 | 4.00109512203375 | 2.85396098699187 | 2.2586792389916 |
| tF(GAA)H2 | 3.4886808964968 | 2.52871238495674 | 1.21310219038481 | 2.08450071642737 | 0.0371146451231674 | 0.0536854567950187 | 0 | 1.64282163827046 | 1.4705105537068 | 5.334793496045 | 5.70792197398374 | 6.77603771697479 |
| tF(GAA)P1 | 20.0069995260488 | 2.24104545821754 | 0.640536086291836 | 3.49870289305838 | 0.000467527229751517 | 0.000886661290107702 | 10.6406161942424 | 8.21410819135229 | 2.20576583056019 | 40.0109512203375 | 22.831687895935 | 36.1388678238655 |
| tF(GAA)P2 | 1.28251264927046 | 1.16104900211588 | 1.76382182148912 | 0.658257533709192 | 0.510372660720951 | 0.573208151936985 | 0 | 1.64282163827046 | 0.735255276853398 | 1.33369837401125 | 2.85396098699187 | 1.1293396194958 |
| tG(GCC)D2 | 20.8641884085726 | 1.89244021622947 | 0.602365187922859 | 3.14168257756592 | 0.00167980039931307 | 0.00295502722808369 | 6.84039612487009 | 12.3211622870284 | 7.35255276853398 | 26.673967480225 | 52.7982782593496 | 19.1987735314286 |
| tG(GCC)E | 2.0718752848797 | 0.748787288397031 | 1.55059318138749 | 0.48290376701322 | 0.629164066950671 | 0.685611246344431 | 1.52008802774891 | 1.64282163827046 | 1.4705105537068 | 6.66849187005625 | 0 | 1.1293396194958 |
| tG(GCC)G1 | 3.2013901602326 | 0.77342059794931 | 1.13786810923982 | 0.679710233258944 | 0.49668795517218 | 0.560556477683445 | 2.28013204162336 | 3.28564327654092 | 1.4705105537068 | 5.334793496045 | 5.70792197398374 | 1.1293396194958 |
| tG(GCC)G2 | 3.37384527363004 | 1.7361797534789 | 1.22967530156569 | 1.41190097196252 | 0.157979121522702 | 0.203910961631094 | 3.04017605549782 | 1.64282163827046 | 0 | 5.334793496045 | 5.70792197398374 | 4.51735847798319 |
| tG(GCC)J2 | 1.7443404482484 | 2.53006002099322 | 1.61916205971699 | 1.56257368174464 | 0.118152902621462 | 0.156109131473376 | 0 | 0.821410819135229 | 0.735255276853398 | 2.6673967480225 | 2.85396098699187 | 3.38801885848739 |
| tG(GCC)O2 | 1.85962798435549 | 3.63761754454431 | 1.78045854827643 | 2.04307904166918 | 0.0410446142548909 | 0.0588451299861235 | 0 | 0.821410819135229 | 0 | 2.6673967480225 | 4.28094148048781 | 3.38801885848739 |
| tG(GCC)P1 | 1.42644832589039 | 1.44313410342848 | 1.70941786927636 | 0.844225469597671 | 0.398543424639676 | 0.464168190377615 | 0.760044013874454 | 0.821410819135229 | 0.735255276853398 | 0 | 2.85396098699187 | 3.38801885848739 |
| tH(GUG)E2 | 3.84357650457708 | 3.1083963243709 | 1.4800028796871 | 2.1002637001815 | 0.0357056505831263 | 0.0517859681757954 | 0 | 2.46423245740569 | 0 | 10.66958699209 | 4.28094148048781 | 5.64669809747899 |
| tH(GUG)M | 5.53406419216488 | 2.6226643933482 | 0.997432902301392 | 2.62941435689246 | 0.00855320729318518 | 0.0136606999516373 | 1.52008802774891 | 2.46423245740569 | 0.735255276853398 | 6.66849187005625 | 7.13490246747968 | 14.6814150534454 |
| tK(CUU)E1 | 1.71078372589608 | 3.50196528786066 | 1.92494114133096 | 1.81925837246083 | 0.0688720210341106 | 0.0950029622346324 | 0.760044013874454 | 0 | 0 | 2.6673967480225 | 5.70792197398374 | 1.1293396194958 |
| tK(CUU)M | 5.91788807889111 | 1.32502653792501 | 0.889506413507977 | 1.48961999351917 | 0.136324181934323 | 0.177656950778786 | 4.56026408324673 | 3.28564327654092 | 2.20576583056019 | 6.66849187005625 | 14.2698049349594 | 4.51735847798319 |
| tK(CUU)P | 1.26397077922672 | -0.0482304115995078 | 1.69959852151851 | -0.0283775320988254 | 0.97736104377154 | 0.982663731189306 | 1.52008802774891 | 1.64282163827046 | 0.735255276853398 | 0 | 1.42698049349594 | 2.2586792389916 |
| tK(UUU)D | 17.7415487901077 | 0.594450814700337 | 0.765474022918309 | 0.776578690984235 | 0.437407377587374 | 0.502572940838212 | 19.7611443607358 | 18.8924488401103 | 3.67627638426699 | 20.0054756101688 | 32.8205513504065 | 11.293396194958 |
| tK(UUU)L | 4.20939705574129 | 1.64545229015951 | 0.955069539319365 | 1.72286123933148 | 0.084913621184025 | 0.115243721908262 | 1.52008802774891 | 1.64282163827046 | 2.94102110741359 | 6.66849187005625 | 5.70792197398374 | 6.77603771697479 |
| tK(UUU)O | 2.87440427441554 | 2.19043385704634 | 1.28336993506779 | 1.70678289805086 | 0.0878624122866037 | 0.1189222884163 | 0 | 1.64282163827046 | 1.4705105537068 | 5.334793496045 | 4.28094148048781 | 4.51735847798319 |
| tK(UUU)P | 5.94960144311468 | 1.63110358955953 | 1.02158033083328 | 1.59664741022283 | 0.110344324459751 | 0.14659837944407 | 3.04017605549782 | 4.92846491481137 | 0.735255276853398 | 9.33588861807875 | 14.2698049349594 | 3.38801885848739 |
| tL(CAA)A | 4.97128429266749 | 1.90809137446324 | 0.981432818927262 | 1.94418949281607 | 0.0518726043792518 | 0.0729817162698107 | 2.28013204162336 | 3.28564327654092 | 0.735255276853398 | 5.334793496045 | 11.4158439479675 | 6.77603771697479 |
| tL(CAA)C | 2.09329751289602 | 2.1481355677925 | 1.36851524697407 | 1.56968332836791 | 0.116488801682363 | 0.154161674701451 | 0.760044013874454 | 0.821410819135229 | 0.735255276853398 | 4.00109512203375 | 2.85396098699187 | 3.38801885848739 |
| tL(CAA)G2 | 1.3208546608832 | -1.00447171726058 | 2.06594396452947 | -0.486204725058628 | 0.626822021587522 | 0.683462224171408 | 0.760044013874454 | 0.821410819135229 | 3.67627638426699 | 2.6673967480225 | 0 | 0 |
| tL(CAA)K | 5.32888324069188 | 3.68854022106273 | 1.27211333698059 | 2.89953741843288 | 0.00373713735959972 | 0.00628117981714531 | 2.28013204162336 | 0 | 0 | 10.66958699209 | 9.98886345447155 | 9.03471695596638 |
| tL(CAA)L | 11.6370695767671 | 1.23576164050403 | 0.72606954292416 | 1.70198798799237 | 0.0887576200532204 | 0.119983727316629 | 12.9207482358657 | 4.92846491481137 | 2.94102110741359 | 14.6706821141238 | 18.5507464154472 | 15.8107546729412 |
| tL(CAA)N | 2.8288655829219 | 2.7121391550154 | 1.74828190346056 | 1.55131683834682 | 0.120825773527768 | 0.159299964593549 | 0.760044013874454 | 0 | 1.4705105537068 | 0 | 5.70792197398374 | 9.03471695596638 |
| tL(GAG)G | 2.3317593689476 | 1.04764473065349 | 1.47021814512851 | 0.712577745095047 | 0.476107087097281 | 0.539959882306866 | 2.28013204162336 | 0 | 2.20576583056019 | 2.6673967480225 | 5.70792197398374 | 1.1293396194958 |
| tL(UAA)B2 | 1.62852602061505 | 1.12584892075021 | 1.68755826097685 | 0.667146697559647 | 0.504678434351214 | 0.568190301198051 | 0 | 0.821410819135229 | 2.20576583056019 | 1.33369837401125 | 4.28094148048781 | 1.1293396194958 |
| tL(UAA)J | 1.62179790210617 | 3.42893684002274 | 1.83539290614122 | 1.86823040916717 | 0.0617299618710262 | 0.0857529519587655 | 0 | 0.821410819135229 | 0 | 2.6673967480225 | 2.85396098699187 | 3.38801885848739 |
| tL(UAA)L | 2.14358901802959 | 1.70497833050639 | 1.54225768747767 | 1.10550807711962 | 0.268939496466218 | 0.328632445639107 | 0 | 0.821410819135229 | 2.20576583056019 | 1.33369837401125 | 2.85396098699187 | 5.64669809747899 |
| tM(CAU)E | 1.93913403539929 | 1.99391905944284 | 1.47171371218909 | 1.3548280775865 | 0.17547233871343 | 0.224524282678103 | 0.760044013874454 | 0.821410819135229 | 0.735255276853398 | 5.334793496045 | 2.85396098699187 | 1.1293396194958 |
| tM(CAU)J2 | 3.64888779970219 | 1.88971254730192 | 1.09525329615843 | 1.72536577057565 | 0.0844615759085213 | 0.114678204174727 | 0.760044013874454 | 1.64282163827046 | 2.20576583056019 | 5.334793496045 | 8.56188296097562 | 3.38801885848739 |
| tN(GUU)F | 1.92774756919397 | 3.68303758909412 | 1.86526738786915 | 1.97453599041452 | 0.0483208292574225 | 0.0684149527534692 | 0 | 0.821410819135229 | 0 | 5.334793496045 | 4.28094148048781 | 1.1293396194958 |
| tN(GUU)K | 4.00228204228377 | 0.254511986925976 | 1.2153526359207 | 0.209414106987286 | 0.834124981120079 | 0.865067896953123 | 3.80022006937227 | 4.92846491481137 | 2.20576583056019 | 0 | 8.56188296097562 | 4.51735847798319 |
| tN(GUU)P | 58.658187426644 | 2.95886312948327 | 0.343902401845414 | 8.60378733502794 | 7.71278309646297e-18 | 5.80337716515763e-17 | 11.4006602081168 | 13.1425731061637 | 15.4403608139214 | 100.027378050844 | 122.72032244065 | 89.217829940168 |
| tP(AGG)C | 3.09475154575293 | 3.4447339810761 | 1.42708414199749 | 2.41382682331156 | 0.0157859633288537 | 0.0242567538855591 | 0.760044013874454 | 0.821410819135229 | 0 | 5.334793496045 | 7.13490246747968 | 4.51735847798319 |
| tP(UGG)F | 1.56143639736975 | 2.3609753791196 | 1.71450197932248 | 1.3770619151181 | 0.168493106202971 | 0.21640520531336 | 0.760044013874454 | 0 | 0.735255276853398 | 1.33369837401125 | 4.28094148048781 | 2.2586792389916 |
| tP(UGG)M | 10.4025285333504 | 2.38350006975649 | 0.662691588588474 | 3.59669582472492 | 0.000322284980734659 | 0.000625663090405567 | 3.04017605549782 | 4.10705409567614 | 2.94102110741359 | 16.004380488135 | 17.1237659219512 | 19.1987735314286 |
| tP(UGG)N2 | 8.93891892046377 | 2.94026000713591 | 0.769733348328083 | 3.81984230451021 | 0.000133537021548414 | 0.000270916835083834 | 2.28013204162336 | 2.46423245740569 | 1.4705105537068 | 17.3380788621463 | 14.2698049349594 | 15.8107546729412 |
| tP(UGG)O3 | 9.77665469163386 | 1.25935563866681 | 0.757210929469243 | 1.66315037152137 | 0.0962823390156972 | 0.129454239397971 | 6.08035211099563 | 8.21410819135229 | 2.94102110741359 | 14.6706821141238 | 19.9777269089431 | 6.77603771697479 |
| tQ(UUG)E1 | 1.67256337976805 | 1.76356770807949 | 1.56598190483814 | 1.12617374608922 | 0.260091983859932 | 0.318955682986485 | 1.52008802774891 | 0 | 0.735255276853398 | 2.6673967480225 | 2.85396098699187 | 2.2586792389916 |
| tR(ACG)D | 2.05871082895051 | 1.58382022062222 | 1.39608622231561 | 1.13447163599626 | 0.256596764962489 | 0.315205798777784 | 0.760044013874454 | 0.821410819135229 | 1.4705105537068 | 1.33369837401125 | 5.70792197398374 | 2.2586792389916 |
| tR(UCU)G2 | 9.87940426953474 | 1.11735058231626 | 0.692265803276835 | 1.61404850135207 | 0.106516906444964 | 0.141949590138081 | 7.60044013874454 | 7.39269737221706 | 3.67627638426699 | 9.33588861807875 | 19.9777269089431 | 11.293396194958 |
| tR(UCU)K | 1.33070352388162 | 2.08106036097049 | 2.47268980934668 | 0.841618044084688 | 0.400001785872895 | 0.465168655824141 | 1.52008802774891 | 0 | 0 | 5.334793496045 | 0 | 1.1293396194958 |
| tS(AGA)D2 | 2.32941294075193 | 1.77753218125919 | 1.3626061586708 | 1.30450913490157 | 0.192060046961097 | 0.243206064933557 | 0.760044013874454 | 1.64282163827046 | 0.735255276853398 | 4.00109512203375 | 5.70792197398374 | 1.1293396194958 |
| tS(AGA)D3 | 1.32305032605555 | 0.113744370237738 | 1.98933987997336 | 0.0571769416492376 | 0.954404246003316 | 0.964516350822298 | 2.28013204162336 | 0 | 1.4705105537068 | 1.33369837401125 | 2.85396098699187 | 0 |
| tS(AGA)J | 3.88666672512491 | 1.44977188912548 | 1.05404190493535 | 1.37544046620652 | 0.168994930721433 | 0.216963800605545 | 3.04017605549782 | 2.46423245740569 | 0.735255276853398 | 4.00109512203375 | 8.56188296097562 | 4.51735847798319 |
| tS(CGA)C | 1.41503276214645 | 1.42033718914606 | 1.70944742063652 | 0.830875037160955 | 0.406044226980531 | 0.470874817842969 | 1.52008802774891 | 0.821410819135229 | 0 | 1.33369837401125 | 1.42698049349594 | 3.38801885848739 |
| tS(GCU)F | 9.08022704187697 | 1.53453845713219 | 0.686945929097188 | 2.23385625000929 | 0.0254925330084521 | 0.0379317662520808 | 6.08035211099563 | 4.92846491481137 | 2.94102110741359 | 14.6706821141238 | 15.6967854284553 | 10.1640565754622 |
| tS(GCU)O | 4.78167418256794 | 2.66734303936593 | 1.10058158532821 | 2.42357593014832 | 0.0153685401047236 | 0.0236601830332868 | 2.28013204162336 | 1.64282163827046 | 0 | 8.0021902440675 | 9.98886345447155 | 6.77603771697479 |
| tS(UGA)E | 5.71013885808552 | 0.713799438864398 | 0.80552908651212 | 0.886124971545219 | 0.375550188084213 | 0.440951940607206 | 3.80022006937227 | 4.10705409567614 | 5.14678693797379 | 5.334793496045 | 5.70792197398374 | 10.1640565754622 |
| tT(AGU)I1 | 1.55969264543773 | 0.488348811742719 | 1.65678625918168 | 0.294756676690404 | 0.768179763754624 | 0.808439712431038 | 2.28013204162336 | 0.821410819135229 | 0.735255276853398 | 2.6673967480225 | 2.85396098699187 | 0 |
| tT(AGU)N1 | 1.32433228396313 | 0.598769331832493 | 2.03463630873079 | 0.294288138505701 | 0.768537735663766 | 0.808464740154445 | 0.760044013874454 | 2.46423245740569 | 0 | 1.33369837401125 | 0 | 3.38801885848739 |
| tT(CGU)K | 3.97325780642604 | 1.804157642466 | 1.18234886168983 | 1.52590973859226 | 0.127032345022765 | 0.166303134710801 | 2.28013204162336 | 0 | 2.94102110741359 | 6.66849187005625 | 8.56188296097562 | 3.38801885848739 |
| tV(AAC)E2 | 3.06089083065012 | 1.8455557353882 | 1.38645050171813 | 1.33113712541568 | 0.183143895116915 | 0.233311998892481 | 0.760044013874454 | 3.28564327654092 | 0 | 2.6673967480225 | 7.13490246747968 | 4.51735847798319 |
| tV(AAC)G1 | 1.3845995936591 | 0.795468404269062 | 1.6790822971462 | 0.473751885551445 | 0.635676848795429 | 0.691314560913339 | 1.52008802774891 | 0 | 1.4705105537068 | 1.33369837401125 | 2.85396098699187 | 1.1293396194958 |
| tV(AAC)G3 | 5.73678407702431 | 1.27685994429434 | 1.04387547526923 | 1.2231918217688 | 0.221257253769826 | 0.276188327165337 | 4.56026408324673 | 2.46423245740569 | 2.94102110741359 | 13.3369837401125 | 9.98886345447155 | 1.1293396194958 |
| tV(AAC)L | 1.97194272962093 | 2.75783830004402 | 1.57513771696434 | 1.75085535083181 | 0.0799708294022979 | 0.109313129505438 | 0.760044013874454 | 0 | 0.735255276853398 | 2.6673967480225 | 4.28094148048781 | 3.38801885848739 |
| tV(AAC)M2 | 1.68692263681502 | 1.73675205250455 | 1.56893768920758 | 1.10696050228848 | 0.268311005607259 | 0.32804244719485 | 1.52008802774891 | 0.821410819135229 | 0 | 2.6673967480225 | 2.85396098699187 | 2.2586792389916 |
| tV(AAC)O | 1.12416655877696 | -0.558204024510379 | 2.11093741375816 | -0.264434189698023 | 0.791445369256668 | 0.828611393600894 | 1.52008802774891 | 2.46423245740569 | 0 | 1.33369837401125 | 1.42698049349594 | 0 |
| tV(CAC)H | 2.1798735731598 | 4.938956361798 | 2.53209704539522 | 1.95053991740949 | 0.0511118004722532 | 0.072067638665877 | 0 | 0 | 0 | 0 | 8.56188296097562 | 4.51735847798319 |
| tV(UAC)D | 2.07125596048435 | 0.0940826195216484 | 1.49268123194732 | 0.0630292774559177 | 0.949743190626113 | 0.96040447995026 | 3.04017605549782 | 0 | 2.94102110741359 | 1.33369837401125 | 2.85396098699187 | 2.2586792389916 |
| tW(CCA)G1 | 3.95877157465704 | 0.226288990692319 | 1.21576370705505 | 0.186129088554929 | 0.85234353111344 | 0.88016241725868 | 2.28013204162336 | 5.7498757339466 | 2.94102110741359 | 0 | 7.13490246747968 | 5.64669809747899 |
| tW(CCA)K | 3.80889738776249 | 2.31624648380465 | 1.08261359554094 | 2.13949510087881 | 0.032395592271223 | 0.0473025239691923 | 1.52008802774891 | 0.821410819135229 | 1.4705105537068 | 4.00109512203375 | 7.13490246747968 | 7.90537733647059 |
| tW(CCA)P | 5.54014182250735 | -0.12448481697905 | 0.912043109695859 | -0.136490058041842 | 0.891433883533357 | 0.913547190487811 | 4.56026408324673 | 9.03551901048752 | 3.67627638426699 | 8.0021902440675 | 5.70792197398374 | 2.2586792389916 |
| tX(XXX)D | 4.20320110483795 | 3.92186210921625 | 1.35290655358535 | 2.89884182970574 | 0.00374543787106219 | 0.00629350000821486 | 0.760044013874454 | 0.821410819135229 | 0 | 8.0021902440675 | 9.98886345447155 | 5.64669809747899 |
| tY(GUA)D | 3.45350912594779 | 0.762713388475393 | 1.00727935656604 | 0.75720144913482 | 0.448929182168097 | 0.513990233988045 | 3.04017605549782 | 1.64282163827046 | 2.94102110741359 | 4.00109512203375 | 5.70792197398374 | 3.38801885848739 |
| tY(GUA)F1 | 4.74212519133356 | 6.06064492231804 | 1.511955817768 | 4.00848017587244 | 6.11107676095832e-05 | 0.000129533345720274 | 0 | 0 | 0 | 8.0021902440675 | 11.4158439479675 | 9.03471695596638 |
| tY(GUA)J1 | 2.27152115679025 | 1.72043360445966 | 1.52530892468823 | 1.12792469552443 | 0.25935172335124 | 0.31816820080502 | 0 | 2.46423245740569 | 0.735255276853398 | 1.33369837401125 | 5.70792197398374 | 3.38801885848739 |
| YAL001C | 361.181931917362 | -1.13287011780182 | 0.152288315952643 | -7.43898250312326 | 1.01463760457904e-13 | 5.17369457806575e-13 | 514.549797393006 | 504.346242949031 | 469.092866632468 | 246.734199192081 | 221.18197649187 | 211.186508845714 |
| YAL002W | 844.28241647727 | 0.418351944910031 | 0.241745684677053 | 1.73054565780111 | 0.0835328311464847 | 0.113559828718529 | 573.833230475213 | 630.022098276721 | 963.919667954805 | 925.586671563808 | 1047.40368222602 | 924.929148367058 |
| YAL003W | 51862.2178362743 | 0.0977958150472309 | 0.234241513132236 | 0.417499928768059 | 0.676312776397135 | 0.728302285856188 | 49188.5284899269 | 46090.1824724968 | 55037.5337488611 | 52292.9795466071 | 39571.5960651358 | 68992.4866946178 |
| YAL005C | 13377.8195577627 | 1.94226631721335 | 0.546164995223449 | 3.55618967564687 | 0.000376272464009178 | 0.000722897867761708 | 2805.32245521061 | 2400.16241351314 | 11367.7818354304 | 18361.0255150129 | 24461.2996195073 | 20871.3255079018 |
| YAL007C | 4404.62244914792 | 0.769037736314885 | 0.0888599681574343 | 8.65449034319225 | 4.95119098931038e-18 | 3.7869604665881e-17 | 3177.74402200909 | 3229.78734083972 | 3365.263402158 | 5448.15785783596 | 5665.11255917887 | 5541.66951286588 |
| YAL008W | 617.70190543533 | 1.27651942734071 | 0.153972864534104 | 8.29054802093368 | 1.12727870575051e-16 | 7.68825413827319e-16 | 326.818925966015 | 341.706900760255 | 413.948720868463 | 882.908323595448 | 890.435827941464 | 850.392733480336 |
| YAL009W | 736.548104984191 | 0.977368169158062 | 0.162593249782222 | 6.01112389639271 | 1.84241526909536e-09 | 6.32270128854631e-09 | 433.985131922313 | 481.346740013244 | 572.763860668797 | 970.93241628019 | 1027.42595531707 | 932.834525703529 |
| YAL010C | 281.666017167845 | -2.24111643927981 | 0.240718743263876 | -9.31010360428433 | 1.27707717151073e-20 | 1.16828244491095e-19 | 453.746276283049 | 414.812463663291 | 527.178033503887 | 113.364361790956 | 71.3490246747968 | 109.545943091092 |
| YAL011W | 102.789823359759 | -0.554857166206239 | 0.482815095353933 | -1.14921254854199 | 0.250468347766676 | 0.308437004673374 | 155.809022844263 | 164.282163827046 | 47.0563377186175 | 81.3556008146863 | 75.6299661552846 | 92.6058487986554 |
| YAL012W | 23780.9480193519 | 0.894628398043832 | 0.266923295463626 | 3.35163102377381 | 0.000803370132856565 | 0.00147027615172339 | 12748.2182447162 | 13740.5601824941 | 23416.410057227 | 30006.8797168791 | 28860.6804809553 | 33912.9394338393 |
| YAL013W | 462.289187600654 | -0.273730304345139 | 0.381237656208113 | -0.718004373092968 | 0.472754590421148 | 0.537002849995021 | 614.875607224433 | 657.128655308183 | 246.310517745888 | 346.761577242925 | 430.948109035773 | 477.710659046723 |
| YAL014C | 1462.83092607007 | 1.44760321902784 | 0.253559875384842 | 5.70911788322476 | 1.1356321653692e-08 | 3.57385260775576e-08 | 658.958160029152 | 615.236703532287 | 1080.09000169764 | 1999.21386264286 | 2224.66258936016 | 2198.82423915832 |
| YAL015C | 616.8743678588 | 0.144954607950706 | 0.192359121738078 | 0.753562433852659 | 0.451112008653262 | 0.51612497585554 | 507.709401268135 | 565.952054384173 | 684.522662750514 | 676.185075623704 | 556.522392463415 | 710.354620662857 |
| YAL016C-A | 33.3804536816025 | 1.63173426156623 | 0.423582667335357 | 3.8522215080967 | 0.000117051064815223 | 0.000239266689691628 | 13.6807922497402 | 11.4997514678932 | 23.5281688593087 | 57.3490300824838 | 45.6633757918699 | 48.5616036383193 |
| YAL016C-B | 1.48625384156192 | 0.386778320368318 | 1.46534424415175 | 0.263950482565422 | 0.791818075094017 | 0.828744882210714 | 1.52008802774891 | 1.64282163827046 | 0.735255276853398 | 1.33369837401125 | 1.42698049349594 | 2.2586792389916 |
| YAL016W | 4978.97416277589 | 0.416739200790439 | 0.114825792253012 | 3.62931700808279 | 0.000284172092924815 | 0.000556167831837763 | 4190.88269250374 | 4369.08414698028 | 4233.59988412187 | 6034.98514240091 | 5876.30567221626 | 5168.98743843227 |
| YAL017W | 766.871462118362 | -0.475665552034281 | 0.190612028250947 | -2.49546451186203 | 0.0125792337378343 | 0.0195516199433485 | 752.44357373571 | 841.94608961361 | 1082.2957675282 | 640.1752195254 | 607.893690229269 | 676.474432077983 |
| YAL018C | 26.2545778115891 | 0.841882208678438 | 0.434268208203763 | 1.93862270544893 | 0.0525472967190914 | 0.0738348714298152 | 17.4810123191124 | 18.071038020975 | 20.5871477518952 | 32.00876097627 | 45.6633757918699 | 23.7161320094118 |
| YAL019W | 340.84306189999 | -3.79247404767588 | 0.474271280908106 | -7.99642356672807 | 1.28085220671208e-15 | 7.87450939595694e-15 | 829.20801913703 | 790.19720800809 | 288.220068526532 | 64.01752195254 | 27.1126293764228 | 46.3029243993277 |
| YAL020C | 1248.9833404561 | 1.19133688274083 | 0.148483742932341 | 8.02334894860297 | 1.02900500421187e-15 | 6.36844127606696e-15 | 773.724806124194 | 704.770482818026 | 803.634017600764 | 1516.41505125079 | 1830.81597315529 | 1864.53971178756 |
| YAL021C | 1081.03312022869 | -0.589327379449509 | 0.210485103220301 | -2.79985315080802 | 0.00511258589419515 | 0.00841630256592634 | 1108.90421624283 | 1197.61697429916 | 1590.3571638339 | 749.538486194323 | 850.480374123578 | 989.301506678319 |
| YAL022C | 2797.41018290861 | 0.667919050697683 | 0.097497392126928 | 6.85063503881361 | 7.35228703977008e-12 | 3.15181320158287e-11 | 2168.40557158382 | 2255.59410934534 | 2060.18528574322 | 3427.60482120891 | 3376.23584761138 | 3496.43546195899 |
| YAL023C | 12418.5678613634 | 0.699317842021176 | 0.113046875704751 | 6.18608729928646 | 6.16758607305547e-10 | 2.21992027024627e-09 | 8995.12090420417 | 9097.12482192266 | 10306.8084709309 | 15568.2611198333 | 14737.854536826 | 15806.2373144632 |
| YAL024C | 222.244486459736 | -0.842275399242462 | 0.190327919748065 | -4.42539066447726 | 9.62679059117156e-06 | 2.25983944170607e-05 | 304.777649563656 | 301.457770622629 | 249.986794130155 | 174.714486995474 | 145.552010336585 | 156.978207109916 |
| YAL025C | 394.314155055364 | -4.54190711037866 | 0.677141839964225 | -6.70746783365007 | 1.98030593153922e-11 | 8.1210581980786e-11 | 1061.78148738261 | 1088.36933535418 | 118.376099573397 | 26.673967480225 | 35.6745123373984 | 35.0095282043697 |
| YAL026C | 879.108717580964 | -1.54826810294611 | 0.151724164971076 | -10.2044924962432 | 1.89308232027379e-24 | 2.38881944149724e-23 | 1367.31918096014 | 1389.00569515767 | 1174.93793241173 | 484.132509766084 | 405.262460152846 | 453.994527037311 |
| YAL026C-A | 42.1093745161364 | -1.09808832675273 | 0.379084186151968 | -2.89668724485522 | 0.00377125514397306 | 0.00633195984048906 | 55.4832130128352 | 48.4632383289785 | 68.378740747366 | 34.6761577242925 | 18.5507464154472 | 27.1041508678992 |
| YAL027W | 191.29792710666 | -0.869544700657149 | 0.197139748508845 | -4.41080354030246 | 1.02987726376348e-05 | 2.40799709184207e-05 | 275.135933022552 | 219.316688709106 | 247.781028299595 | 132.036139027114 | 125.574283427642 | 147.94349015395 |
| YAL028W | 200.25236145268 | -0.0654607793647851 | 0.365899468116778 | -0.178903729217483 | 0.858013294538358 | 0.884749479868965 | 158.849198899761 | 154.425233997423 | 300.71940823304 | 149.37421788926 | 259.71044981626 | 178.435659880336 |
| YAL029C | 1615.47279434907 | -1.14978494775727 | 0.203860207522502 | -5.64006562011549 | 1.69985371052318e-08 | 5.26767853151139e-08 | 2448.10176868962 | 2392.76971614092 | 1841.81446851776 | 1036.28363660674 | 821.940764253659 | 1151.92641188571 |
| YAL030W | 1672.11778592527 | 2.04535849189766 | 0.257353254622677 | 7.94766903141171 | 1.90053590854676e-15 | 1.14137739841058e-14 | 544.191513934109 | 520.774459331735 | 891.129395546319 | 2452.67130980669 | 3063.72711953577 | 2560.21291739697 |
| YAL031C | 376.051392272618 | -1.48395056535415 | 0.167768876925619 | -8.84520771997576 | 9.13582058657289e-19 | 7.45345060685683e-18 | 519.110061476252 | 573.34475175639 | 569.822839561384 | 225.395025207901 | 175.5186007 | 193.117074933781 |
| YAL031W-A | 2.13159540593279 | 0.464540400897724 | 1.4937709718038 | 0.31098502358549 | 0.755812006976979 | 0.798143386071749 | 3.04017605549782 | 1.64282163827046 | 0.735255276853398 | 0 | 2.85396098699187 | 4.51735847798319 |
| YAL032C | 178.387803388459 | -1.90246118108743 | 0.54183150192859 | -3.51116753883047 | 0.000446143125763198 | 0.000848587775278623 | 389.142535103721 | 372.920511887394 | 82.3485910075806 | 68.0186170745738 | 85.6188296097561 | 72.2777356477311 |
| YAL033W | 1247.80747903633 | 1.36911350483667 | 0.13610510347389 | 10.0592370887791 | 8.36440005158093e-24 | 9.88187590793331e-23 | 711.401196986489 | 646.450314659425 | 731.579000469131 | 1940.53113418637 | 1629.61172357236 | 1827.2715043442 |
| YAL034C | 654.442345145959 | -0.096599329567956 | 0.392967794950567 | -0.245819964916228 | 0.805821601423054 | 0.840417897866205 | 477.307640713157 | 463.275701992269 | 1088.17780974303 | 465.460732529926 | 803.390017838212 | 629.042168059159 |
| YAL034C-B | 3.13785854950945 | 0.834909518418369 | 1.22704813636341 | 0.680421161709906 | 0.496237825476991 | 0.560145933874654 | 3.80022006937227 | 0.821410819135229 | 2.20576583056019 | 2.6673967480225 | 1.42698049349594 | 7.90537733647059 |
| YAL034W-A | 46.3890515243969 | -2.56431371453454 | 0.451474849089765 | -5.6798595086848 | 1.34805422677006e-08 | 4.21169543103595e-08 | 91.2052816649345 | 59.9629897968717 | 87.4953779455544 | 13.3369837401125 | 7.13490246747968 | 19.1987735314286 |
| YAL035W | 5252.42282927252 | 0.401051451260226 | 0.126396633849963 | 3.17295990442504 | 0.0015089336173874 | 0.00267329785369426 | 4515.42148642813 | 4954.7500610237 | 4110.81225288735 | 6111.00594971955 | 6164.55573190244 | 5657.99149367395 |
| YAL036C | 5403.45310211028 | 1.06256978932906 | 0.11011314303496 | 9.64979983353764 | 4.92517707278145e-22 | 4.99917034335845e-21 | 3373.07533357483 | 3389.96245057109 | 3733.62629586156 | 7670.0993489387 | 6922.28237394878 | 7331.67280976672 |
| YAL037C-A | 7.59352128707472 | 1.97276691464355 | 0.77310930290426 | 2.55173092243575 | 0.0107189265517026 | 0.0168458826300807 | 1.52008802774891 | 3.28564327654092 | 4.41153166112039 | 13.3369837401125 | 12.8428244414634 | 10.1640565754622 |
| YAL037C-B | 42.1178623222086 | -0.812854298587317 | 0.647032092400393 | -1.25628126971531 | 0.209014010342957 | 0.262522244594194 | 107.166205956298 | 22.1780921166512 | 31.6159769046961 | 34.6761577242925 | 29.9665903634147 | 27.1041508678992 |
| YAL037W | 150.287514991308 | -0.0158007035695888 | 0.177339085272249 | -0.0890988218718492 | 0.929003373682493 | 0.943403144152912 | 151.248758761016 | 147.853947444341 | 154.403608139214 | 149.37421788926 | 144.12502984309 | 154.719527870924 |
| YAL038W | 310271.068183938 | 0.868314764096881 | 0.268324430826699 | 3.23606300559972 | 0.00121190631007882 | 0.00217198793234905 | 167515.220745957 | 178664.245859285 | 312679.805821614 | 388763.740135661 | 408490.290029134 | 405513.106511976 |
| YAL039C | 2222.813267032 | 1.24929291584078 | 0.248585204104548 | 5.02561252726597 | 5.01828206352079e-07 | 1.34387190189909e-06 | 1112.7044363122 | 1075.22676224801 | 1760.93638806389 | 2675.39893826657 | 3220.69497382033 | 3491.91810348101 |
| YAL040C | 4712.4171107932 | -0.479644630474775 | 0.225804104729007 | -2.12416258353854 | 0.0336565507775339 | 0.0490003116370561 | 5191.8606587764 | 6109.65367272783 | 5163.69780934142 | 3170.20103502474 | 5125.7139326374 | 3513.37555625143 |
| YAL041W | 519.801836506307 | -3.24025314458979 | 0.254673912335253 | -12.7231451187051 | 4.39795028828398e-37 | 1.34552384763254e-35 | 1007.05831838365 | 971.728999036976 | 841.867291997141 | 133.369837401125 | 69.9220441813009 | 94.864528037647 |
| YAL042C-A | 5.03670351517234 | 0.0287165666554477 | 1.17390108744681 | 0.0244625096292441 | 0.980483687741386 | 0.984734778366465 | 4.56026408324673 | 0.821410819135229 | 9.55831859909418 | 9.33588861807875 | 1.42698049349594 | 4.51735847798319 |
| YAL042W | 8394.54075883313 | 1.10272179215353 | 0.142127669738594 | 7.75867073724411 | 8.58241462009514e-15 | 4.83207823141815e-14 | 4931.16556201746 | 4908.75105515213 | 6161.43922003148 | 11564.4986010516 | 11478.6310896813 | 11322.7590250649 |
| YAL043C | 904.69095438473 | -1.4004790861343 | 0.229591967437053 | -6.0998609915143 | 1.06160747191351e-09 | 3.72395135902165e-09 | 1067.10179547973 | 1155.72502252327 | 1713.88005034527 | 469.46182765196 | 519.420899632521 | 502.55613067563 |
| YAL044C | 2578.19164725264 | 2.37462248337765 | 0.320812215541971 | 7.40190793348076 | 1.34241564554726e-13 | 6.73909278407085e-13 | 613.355519196685 | 613.593881894016 | 1273.46213951009 | 4269.16849521001 | 3894.22976675041 | 4805.34008095462 |
| YAL044W-A | 1698.83561543332 | 1.62011218925784 | 0.126722581216678 | 12.7847158233597 | 1.99591006272171e-37 | 6.31486471551854e-36 | 769.924586054822 | 809.089656848201 | 922.745372451015 | 2490.014864279 | 2585.68865421464 | 2615.55055875227 |
| YAL045C | 1.87272910495421 | 2.69808530244852 | 1.66494437744796 | 1.62052578992709 | 0.105119378937827 | 0.140231240598672 | 0.760044013874454 | 0 | 0.735255276853398 | 2.6673967480225 | 1.42698049349594 | 5.64669809747899 |
| YAL046C | 1088.84379141717 | 1.74470674765593 | 0.130904443849864 | 13.3280941146425 | 1.5890550680787e-40 | 6.13488760211813e-39 | 479.587772754781 | 472.311221002757 | 549.235691809489 | 1583.09996995135 | 1720.9384751561 | 1727.88961782857 |
| YAL047C | 25.7013930164619 | -2.26344140949188 | 0.68650182806719 | -3.29706537834644 | 0.000977007559524607 | 0.00177106512886434 | 60.0434770960819 | 50.106059967249 | 17.6461266444816 | 4.00109512203375 | 9.98886345447155 | 12.4227358144538 |
| YAL047W-A | 16.6069644274702 | 1.41609122809781 | 0.506989529652702 | 2.79313702803264 | 0.00521995745738042 | 0.00857999089421424 | 9.8805721803679 | 10.678340648758 | 6.61729749168058 | 21.33917398418 | 28.5396098699187 | 22.586792389916 |
| YAL048C | 491.964077365831 | 0.0153934579580357 | 0.209053033256079 | 0.073634224379703 | 0.941301437931476 | 0.953534661056606 | 411.943855519954 | 441.919020694753 | 613.938156172588 | 517.474969116365 | 480.89242630813 | 485.616036383193 |
| YAL049C | 2969.37438965739 | 1.51155397046294 | 0.161874520120986 | 9.33781282769639 | 9.8344582317788e-21 | 9.09932897165725e-20 | 1403.04124961224 | 1389.00569515767 | 1833.72666047238 | 4251.83041634787 | 4493.5615740187 | 4445.08074233546 |
| YAL051W | 628.746326909208 | -0.883494784669507 | 0.155397283871376 | -5.6853939956942 | 1.30511418322006e-08 | 4.08541051755082e-08 | 878.610880038869 | 804.982602752524 | 761.724466820121 | 430.784574805634 | 496.589211736586 | 399.786225301512 |
| YAL053W | 6950.03597276352 | -0.731148661091163 | 0.140445268681341 | -5.20593301544448 | 1.93024270546485e-07 | 5.40334664982522e-07 | 8442.56890611744 | 9327.11985128053 | 8252.50522740254 | 4918.67960335349 | 5907.69924307317 | 4851.64300535395 |
| YAL054C | 473.586496693823 | 1.58077062629408 | 0.21818421142214 | 7.24511923200357 | 4.32058945174157e-13 | 2.07120053096791e-12 | 202.931751704479 | 215.20963461343 | 293.366855464506 | 636.174124403366 | 816.232842279675 | 677.603771697479 |
| YAL055W | 58.9921380941708 | -0.734343071821632 | 0.38167739292759 | -1.92398891165385 | 0.0543559733599473 | 0.0761617721349359 | 61.5635651238308 | 55.8559357011956 | 103.670994036329 | 52.0142365864388 | 35.6745123373984 | 45.1735847798319 |
| YAL056C-A | 3.62061592276929 | 1.12581762947601 | 1.18862524222958 | 0.94715944898179 | 0.343557535285304 | 0.407593593170016 | 4.56026408324673 | 0.821410819135229 | 1.4705105537068 | 6.66849187005625 | 1.42698049349594 | 6.77603771697479 |
| YAL056W | 1469.78708042972 | -0.0677984498897164 | 0.167792074094271 | -0.404062291116475 | 0.686166913551896 | 0.736834205512847 | 1565.69066858138 | 1707.71309298214 | 1239.64039677483 | 1455.06492604627 | 1462.65500583333 | 1387.95839236034 |
| YAL058W | 770.415177633081 | 0.093501427832986 | 0.214668396310455 | 0.43556214813178 | 0.663154438841315 | 0.716750489972466 | 651.357719890407 | 690.806498892728 | 894.805671930586 | 786.882040666638 | 663.54592947561 | 935.093204942521 |
| YAL059C-A | 4.68696648232194 | -0.26010790085684 | 0.921062895781055 | -0.282399716727565 | 0.777637032178065 | 0.816141390082027 | 8.360484152619 | 3.28564327654092 | 3.67627638426699 | 4.00109512203375 | 4.28094148048781 | 4.51735847798319 |
| YAL059W | 1366.04556562262 | -0.145478089831555 | 0.202062887141 | -0.719964422412914 | 0.471546901049119 | 0.535818710617481 | 1602.93282526122 | 1552.46644816558 | 1149.93925299871 | 1373.70932523159 | 1098.77497999187 | 1418.45056208672 |
| YAL060W | 2708.00493816229 | 1.65357753559416 | 0.152503782027366 | 10.8428624760102 | 2.15617056646962e-27 | 3.29489888899779e-26 | 1200.86954192164 | 1226.3663529689 | 1491.09770145869 | 3823.71323829026 | 4482.14573007073 | 4023.83706426353 |
| YAL061W | 493.189055820718 | 0.49128475719629 | 0.320546656186169 | 1.53264664508295 | 0.125362941940402 | 0.164595959802722 | 377.741874895604 | 318.707397824469 | 533.060075718714 | 432.118273179645 | 806.243978825204 | 491.262734480672 |
| YAL062W | 348.486498398264 | 1.10389490889653 | 0.188311411430323 | 5.862071238869 | 4.57128813113491e-09 | 1.50275594620076e-08 | 250.054480564695 | 222.602331985647 | 191.166371981884 | 428.117178057611 | 533.69070456748 | 465.287923232269 |
| YAL063C | 280.423587706954 | 1.10518302144462 | 0.333067782321353 | 3.31819251247276 | 0.000906020162109626 | 0.00164790991908105 | 120.086954192164 | 151.139590720882 | 262.486133836663 | 321.421308136711 | 436.656031009756 | 390.751508345546 |
| YAL063C-A | 61.5970638319591 | 0.336138653619539 | 0.357212830710454 | 0.941003863021941 | 0.346702879539179 | 0.410660394798863 | 66.883873220952 | 45.9990058715728 | 49.9973588260311 | 73.3534105706188 | 87.0458101032521 | 46.3029243993277 |
| YAL064C-A | 9.75027306762304 | -0.846173464563655 | 0.707803757592896 | -1.19549162530774 | 0.231895006333409 | 0.288357172369343 | 8.360484152619 | 10.678340648758 | 18.381381921335 | 8.0021902440675 | 8.56188296097562 | 4.51735847798319 |
| YAL064W | 15.4789879949337 | 1.24716202803676 | 0.627438030891963 | 1.98770550497839 | 0.0468442697406062 | 0.06651312030157 | 14.4408362636146 | 4.10705409567614 | 8.82306332224078 | 24.0065707322025 | 25.6856488829268 | 15.8107546729412 |
| YAL064W-B | 7.43087355849107 | -0.966726180619944 | 1.00058263945719 | -0.966163255785035 | 0.333962496067166 | 0.397664907195087 | 6.08035211099563 | 13.9639839252989 | 9.55831859909418 | 9.33588861807875 | 0 | 5.64669809747899 |
| YAL065C | 11.771975032853 | 0.705683162628053 | 0.670311067387769 | 1.05276967211377 | 0.292446567891037 | 0.354676222763887 | 3.80022006937227 | 9.03551901048752 | 13.9698502602146 | 16.004380488135 | 14.2698049349594 | 13.5520754339496 |
| YAL067C | 95.2777983004122 | 0.161285800839815 | 0.271672860706437 | 0.593676528529277 | 0.55272848464425 | 0.6147114114202 | 80.5646654706921 | 83.7839035517934 | 105.141504590036 | 122.700250409035 | 97.0346735577236 | 82.4417922231932 |
| YAL068C | 1.8407803593899 | 0.00626404070416735 | 1.59208497282362 | 0.00393448893186766 | 0.996860740126012 | 0.998091812358338 | 0.760044013874454 | 2.46423245740569 | 2.20576583056019 | 1.33369837401125 | 4.28094148048781 | 0 |
| YAL069W | 2.19809994257459 | -2.08393678573114 | 1.51575567143217 | -1.37485006654279 | 0.169177932544003 | 0.217155763008194 | 5.32030809712118 | 2.46423245740569 | 2.94102110741359 | 1.33369837401125 | 0 | 1.1293396194958 |
| YAR002C-A | 8570.06534590129 | 1.16720701641532 | 0.179887920393769 | 6.48852359769537 | 8.6681581878504e-11 | 3.36656730577232e-10 | 5723.13142447464 | 5918.26495186933 | 4201.24865194032 | 11059.0269173013 | 12494.6412010504 | 12024.0789287718 |
| YAR002W | 771.687531728698 | -1.24296401515601 | 0.301006967436214 | -4.12935297060658 | 3.63785551554554e-05 | 7.93113642817759e-05 | 1324.75671618317 | 1286.32934276577 | 644.083622523577 | 444.121558545746 | 445.217913970732 | 485.616036383193 |
| YAR003W | 827.23174932176 | 0.385017347139856 | 0.146654507458721 | 2.62533592599073 | 0.00865635172763559 | 0.0138084351464448 | 696.960360722874 | 782.804510635873 | 672.75857832086 | 1038.95103335476 | 893.289788928456 | 878.626223967731 |
| YAR007C | 1337.45380903949 | -1.00630561434497 | 0.125122785232428 | -8.04254486883151 | 8.79916523162059e-16 | 5.48236173797225e-15 | 1757.98180409161 | 1764.39043950247 | 1835.93242630294 | 957.595432540078 | 801.963037344716 | 906.859714455126 |
| YAR008W | 540.188545244108 | 0.0887831470001541 | 0.223525695488587 | 0.397194366428835 | 0.691224136184804 | 0.741650909395308 | 589.794154766576 | 597.165665511312 | 383.803254517474 | 576.15769757286 | 567.938236411382 | 526.272262685042 |
| YAR009C | 58.0577783391542 | -1.72056548687448 | 0.404207003415724 | -4.25664442311726 | 2.07518002805152e-05 | 4.66376218362515e-05 | 96.5255897620557 | 109.247638944985 | 61.0261879788321 | 26.673967480225 | 35.6745123373984 | 19.1987735314286 |
| YAR014C | 820.68672950303 | -1.89468597451869 | 0.183894727018286 | -10.3031011559689 | 6.82256992507975e-25 | 8.86797365412169e-24 | 1438.00327425047 | 1388.18428433854 | 1054.35606700777 | 369.434449601116 | 335.340415971545 | 338.801885848739 |
| YAR015W | 1093.98640756968 | 0.0322515612552131 | 0.187307909557411 | 0.172184726909932 | 0.863292304715281 | 0.889627246327187 | 950.815061356942 | 980.764518047464 | 1313.90117973702 | 1120.30663416945 | 1014.58313087561 | 1183.5479212316 |
| YAR018C | 347.470203416113 | -2.75234520028902 | 0.278749771681047 | -9.87389221411931 | 5.40266024149395e-23 | 5.91919836593408e-22 | 687.079788542507 | 698.199196264945 | 430.859592236091 | 88.0240926847425 | 75.6299661552846 | 105.028584613109 |
| YAR019C | 479.93660700271 | -0.63142791843578 | 0.233747104612621 | -2.70132936825985 | 0.00690629075824924 | 0.0111901578461166 | 475.787552685408 | 521.59587015087 | 752.166148221026 | 353.430069112981 | 415.251323607317 | 361.388678238655 |
| YAR019W-A | 6.20848733667738 | -0.461480317461791 | 0.84031751371302 | -0.549173746745676 | 0.582886219253441 | 0.641867575225436 | 3.80022006937227 | 7.39269737221706 | 10.2935738759476 | 6.66849187005625 | 5.70792197398374 | 3.38801885848739 |
| YAR020C | 70.4757438449983 | 0.633383318704196 | 0.32496705625824 | 1.9490693179707 | 0.0512871471893444 | 0.0722991603282087 | 53.2030809712118 | 40.2491301376262 | 72.055017131633 | 73.3534105706188 | 97.0346735577236 | 86.9591507011764 |
| YAR023C | 295.939127291337 | 1.66233808354848 | 0.222172240019008 | 7.48220427271319 | 7.30862236579751e-14 | 3.78020132891249e-13 | 119.326910178289 | 133.068552699907 | 173.520245337402 | 410.779099195465 | 516.566938645529 | 422.373017691428 |
| YAR027W | 2489.58864253498 | 0.487462893950269 | 0.139274883225888 | 3.5000057631329 | 0.000465248099399794 | 0.00088285522899563 | 2132.68350293172 | 2027.24190162575 | 2057.97951991266 | 2738.0827618451 | 3316.30266688456 | 2665.24150201008 |
| YAR028W | 1334.45616548394 | 0.292582688358051 | 0.183042911543486 | 1.59843768814036 | 0.109945602670199 | 0.146173640293046 | 1273.07372323971 | 1292.90062931885 | 1032.29840870217 | 1381.71151547566 | 1722.36545564959 | 1304.38726051765 |
| YAR029W | 121.092922899899 | 0.39577594035683 | 0.245597801100447 | 1.61147998305963 | 0.107075146629888 | 0.142576350039305 | 103.365985886926 | 84.6053143709286 | 125.728652341931 | 125.367647157058 | 138.417107869106 | 149.072829773445 |
| YAR030C | 31.2104866542999 | 0.137737713103925 | 0.455861028313552 | 0.302148471900488 | 0.7625388828974 | 0.803415723598527 | 34.9620246382249 | 32.8564327654092 | 21.3224030287486 | 20.0054756101688 | 44.236395298374 | 33.8801885848739 |
| YAR031W | 118.464375015866 | 0.468340048037136 | 0.242845163633233 | 1.92855414960813 | 0.053786235754364 | 0.0754449665014717 | 91.2052816649345 | 89.53377928574 | 116.90558901969 | 142.705726019204 | 154.113893297561 | 116.321980808067 |
| YAR033W | 151.280561484555 | 0.946301810708041 | 0.209504291009526 | 4.51686123538642 | 6.27630049908882e-06 | 1.50380809150684e-05 | 92.7253696926834 | 99.3907091153627 | 117.640844296544 | 186.717772361575 | 222.608956985366 | 188.599716455798 |
| YAR035W | 428.725852390901 | 0.16119880553707 | 0.151746575881645 | 1.06228957457859 | 0.288104245814701 | 0.349802347127321 | 416.504119603201 | 402.491301376262 | 396.302594223982 | 422.782384561566 | 423.813206568293 | 510.461508012101 |
| YAR042W | 593.134644909637 | -1.82156069134615 | 0.332115875115193 | -5.48471430555479 | 4.1413815956309e-08 | 1.23498855306952e-07 | 1118.02474440932 | 1140.93962777883 | 515.413949074232 | 258.737484558183 | 235.451781426829 | 290.24028221042 |
| YAR050W | 137.456234994063 | -0.0188346978114889 | 0.277541107256442 | -0.0678627321108363 | 0.945894905798221 | 0.957408607835091 | 126.167306303159 | 126.497266146825 | 162.491416184601 | 97.3599813028213 | 154.113893297561 | 158.107546729412 |
| YAR053W | 4.36584159302881 | 1.94305177117628 | 1.03134474873381 | 1.88399831730542 | 0.0595652030792629 | 0.0829411565418848 | 2.28013204162336 | 1.64282163827046 | 1.4705105537068 | 12.0032853661013 | 4.28094148048781 | 4.51735847798319 |
| YAR068W | 3.03465962699105 | -0.501240544588385 | 1.10867145421678 | -0.452109182284744 | 0.651190329045774 | 0.705583106279801 | 5.32030809712118 | 2.46423245740569 | 2.94102110741359 | 2.6673967480225 | 1.42698049349594 | 3.38801885848739 |
| YAR071W | 72.794536535994 | -0.705469943094151 | 0.338192689578995 | -2.08599997821469 | 0.036978625035317 | 0.0535110552574837 | 104.1260299008 | 105.140584849309 | 61.7614432556855 | 61.3501252045175 | 45.6633757918699 | 58.7256602137815 |
| YAR073W | 61.544165321572 | -3.13783204202621 | 0.725867609943421 | -4.3228710016015 | 1.54011792488344e-05 | 3.51980439069556e-05 | 165.689595024631 | 143.746893348665 | 22.057658305602 | 13.3369837401125 | 14.2698049349594 | 10.1640565754622 |
| YAR075W | 94.8412428064843 | -2.1837752173492 | 0.605679088940677 | -3.60549878181957 | 0.00031155395257618 | 0.00060664633335608 | 225.733072120713 | 190.567310039373 | 49.9973588260311 | 53.34793496045 | 25.6856488829268 | 23.7161320094118 |
| YBL001C | 4686.6502427773 | 2.10269959324294 | 0.106696483130726 | 19.7072999179053 | 1.8666475174009e-86 | 1.00892298315519e-83 | 1706.29881114815 | 1697.85616315252 | 1905.78167760401 | 7584.74265300198 | 7794.1674554748 | 7431.05469628235 |
| YBL002W | 18985.3500493994 | 1.408214216538 | 0.196408756620495 | 7.1698138146609 | 7.50998311100984e-13 | 3.51694949155305e-12 | 8870.47368592876 | 9274.54955885587 | 13028.7235058422 | 28209.054308712 | 25557.2206385122 | 28972.0785985452 |
| YBL003C | 20564.4430527268 | 1.17332505491351 | 0.154452634794088 | 7.5966658417822 | 3.03857820093224e-14 | 1.6220755729421e-13 | 14035.7328042195 | 13219.7857231624 | 10647.9669193909 | 30241.6106307051 | 27288.1479771228 | 27953.41426176 |
| YBL004W | 3010.73497344 | -1.59768622205748 | 0.21728367567257 | -7.35299703078047 | 1.93811244341433e-13 | 9.58124794815958e-13 | 5096.09511302822 | 5046.74807276685 | 3435.84790873593 | 1649.78488865192 | 1278.57452217236 | 1557.35933528471 |
| YBL005W | 634.2441741324 | -0.586516939971415 | 0.163751509541776 | -3.58174982088813 | 0.00034130055320172 | 0.000660207392802372 | 680.239392417637 | 786.91156473155 | 816.133357307272 | 502.804287002241 | 566.511255917887 | 452.865187417815 |
| YBL005W-B | 87.3367585043894 | -4.26110752908238 | 0.704449816435163 | -6.04884468654634 | 1.45888215314776e-09 | 5.05735416639035e-09 | 235.613644301081 | 222.602331985647 | 39.7037849500835 | 9.33588861807875 | 9.98886345447155 | 6.77603771697479 |
| YBL006C | 1101.44386435241 | 0.514500537266039 | 0.184984270973067 | 2.78132045800233 | 0.00541382672601034 | 0.00888289404120999 | 1005.5382303559 | 860.017127634585 | 856.572397534209 | 1255.01016994459 | 1094.49403851138 | 1537.03122213378 |
| YBL006W-A | 11.7254654144529 | 1.14329098963193 | 0.808870176028182 | 1.41344188908765 | 0.157525836319739 | 0.203366356363422 | 6.08035211099563 | 2.46423245740569 | 13.2345949833612 | 9.33588861807875 | 25.6856488829268 | 13.5520754339496 |
| YBL007C | 1724.98179670893 | -1.76452934078932 | 0.139703380854163 | -12.6305414371562 | 1.43288891243374e-36 | 4.22441703911147e-35 | 2574.26907499278 | 2606.33652911608 | 2817.49822090222 | 750.872184568334 | 717.771188228456 | 883.143582445714 |
| YBL008W | 472.791620413726 | -0.723810792648996 | 0.190776406726813 | -3.79402676183892 | 0.000148223687844865 | 0.000299494965533269 | 554.072086114477 | 568.416286841579 | 645.554133077284 | 348.095275616936 | 298.238923140651 | 422.373017691428 |
| YBL009W | 1050.23789437568 | -0.322477653308022 | 0.186184759189965 | -1.7320303483004 | 0.0832681591348201 | 0.113318774684944 | 1061.02144336874 | 1086.72651371591 | 1354.34021996396 | 961.596527662111 | 791.974173890244 | 1045.76848765311 |
| YBL010C | 88.4928535309306 | -3.72784399791327 | 0.368227549900541 | -10.1237509222766 | 4.33477857625028e-24 | 5.28484470781191e-23 | 180.130431288246 | 135.532785157313 | 177.931776998522 | 10.66958699209 | 14.2698049349594 | 12.4227358144538 |
| YBL011W | 1188.40770996151 | -0.245594072529095 | 0.3192872215821 | -0.769194806206624 | 0.441777673241899 | 0.507234906823678 | 972.096293745427 | 965.979123303029 | 1930.04510174017 | 1048.28692197284 | 944.66108669431 | 1269.37773231328 |
| YBL013W | 26.2460182535133 | -0.592978662045782 | 0.50321053168768 | -1.17839080207053 | 0.238640842910868 | 0.295658180041814 | 21.2812323884847 | 29.5707894888682 | 44.1153166112039 | 20.0054756101688 | 14.2698049349594 | 28.2334904873949 |
| YBL014C | 429.491884086082 | -1.39865859380247 | 0.287026717195983 | -4.8729212648433 | 1.09960054444109e-06 | 2.84597331653828e-06 | 652.117763904282 | 621.807990085368 | 595.556774251253 | 254.736389436149 | 145.552010336585 | 307.180376502857 |
| YBL015W | 1618.9394674718 | 1.42452376114855 | 0.251570249244248 | 5.66252871882908 | 1.49158402835344e-08 | 4.63556013794942e-08 | 697.720404736749 | 738.448326402571 | 1199.93661182475 | 2248.61545858297 | 2592.82355668212 | 2236.09244660168 |
| YBL016W | 1272.50918494589 | -0.719190953870092 | 0.227301561162558 | -3.16403877822798 | 0.00155596062456541 | 0.00275210270273555 | 1399.24102954287 | 1366.82760304102 | 1984.45399222732 | 916.250782945729 | 827.648686227643 | 1140.63301569076 |
| YBL017C | 2534.84321097275 | -0.0899107893537275 | 0.167011916243475 | -0.538349546403939 | 0.590335751498887 | 0.648969099020641 | 2321.93446238646 | 2419.05486235325 | 3100.57150249078 | 2328.63736102364 | 2424.4398584496 | 2614.42121913277 |
| YBL018C | 1119.89932237134 | 1.50696751978963 | 0.215489938878938 | 6.99321521751527 | 2.68656894960251e-12 | 1.19513622819766e-11 | 687.079788542507 | 634.129152372397 | 427.918571128678 | 1729.80679109259 | 1615.3419186374 | 1625.11971245445 |
| YBL019W | 296.147686675982 | -0.794950965688739 | 0.143678895928924 | -5.53283041708497 | 3.15104093009752e-08 | 9.47943018210229e-08 | 367.861302715236 | 377.02756598307 | 382.332743963767 | 209.390644719766 | 221.18197649187 | 219.091886182185 |
| YBL020W | 1887.76829933985 | 0.401261945168896 | 0.130983322721902 | 3.06345828484468 | 0.0021879470696689 | 0.00379540644393487 | 1642.4551139827 | 1765.21185032161 | 1473.45157481421 | 2283.29161630726 | 2054.85191063415 | 2107.34772997916 |
| YBL021C | 348.735388168119 | 0.475761902761588 | 0.186872929448421 | 2.54591130007894 | 0.0108992879356317 | 0.017092065171786 | 324.538793924392 | 284.208143420789 | 266.897665497784 | 450.790050415803 | 342.475318439025 | 423.502357310924 |
| YBL022C | 999.459336580166 | -0.506007260000716 | 0.175884189326325 | -2.8769343164888 | 0.00401559171348669 | 0.00671439233144488 | 1072.42210357685 | 1094.94062190726 | 1352.1344541334 | 740.202597576244 | 801.963037344716 | 935.093204942521 |
| YBL023C | 682.620105975912 | -0.436685238245096 | 0.183277612008245 | -2.38264364894416 | 0.0171888242776403 | 0.0262940363832017 | 855.049515608761 | 840.303267975339 | 660.994493891205 | 592.162078060995 | 508.005055684553 | 639.206224634622 |
| YBL024W | 3473.77061195876 | -0.709273866920409 | 0.143936068398406 | -4.92770071332771 | 8.32029344597748e-07 | 2.17866060922931e-06 | 4199.24317665636 | 4599.90058715728 | 4135.07567702351 | 2656.72716103041 | 2313.13537995691 | 2938.54168992807 |
| YBL025W | 236.235557908699 | 0.710211171034749 | 0.175824787331373 | 4.03931198674638 | 5.36082196916821e-05 | 0.000114791321531941 | 167.20968305238 | 165.103574646181 | 205.136222242098 | 292.079943908464 | 295.384962153659 | 292.498961449412 |
| YBL026W | 1728.9525764628 | 0.302846798993857 | 0.206894969317429 | 1.46377072382661 | 0.14325660411506 | 0.186092997053932 | 1673.61691855155 | 1733.99823919447 | 1236.69937566742 | 2227.27628459879 | 1846.51275858374 | 1655.61188218084 |
| YBL027W | 32756.0010574066 | -1.56997487625658 | 0.325679339136547 | -4.82061551837753 | 1.43115953483772e-06 | 3.66029209107155e-06 | 60626.4308547236 | 56599.312492513 | 29793.2790733766 | 16685.9003572548 | 12139.3230581699 | 20691.760508402 |
| YBL028C | 388.124091358733 | -2.0060707440751 | 0.260459992359359 | -7.70203026539028 | 1.33921156169363e-14 | 7.35489093068999e-14 | 736.482649444346 | 649.735957935966 | 479.386440508416 | 146.706821141238 | 125.574283427642 | 190.85839569479 |
| YBL029C-A | 1243.9172551439 | 1.59666726645332 | 0.190846701772485 | 8.36622929096652 | 5.94913765546451e-17 | 4.14014021816983e-16 | 603.474947016317 | 515.845994416924 | 734.520021576545 | 1823.16567727338 | 2089.09944247805 | 1697.39744810218 |
| YBL029W | 180.086375264558 | -0.278788019664773 | 0.252184075447157 | -1.10549414815525 | 0.268945528671921 | 0.328632445639107 | 215.852499940345 | 153.603823178288 | 222.78234888658 | 192.05256585762 | 136.99012737561 | 159.236886348907 |
| YBL030C | 22964.5421265284 | 1.29875534886627 | 0.0991265377377176 | 13.101994465929 | 3.20717477641547e-39 | 1.1429525054852e-37 | 12948.8698643791 | 12725.296410043 | 14146.3115266594 | 33570.5217722372 | 32313.9732752155 | 32082.2799106366 |
| YBL031W | 97.9838582037135 | -2.05153705257327 | 0.416011545982278 | -4.9314425822707 | 8.1624561420599e-07 | 2.14078813333605e-06 | 217.372587968094 | 168.389217922722 | 88.2306332224078 | 37.343554472315 | 31.3935708569106 | 45.1735847798319 |
| YBL032W | 1711.9433417321 | -0.767892223481911 | 0.314311800131268 | -2.44309066080628 | 0.0145620786158636 | 0.0224666132023052 | 1757.98180409161 | 1797.24687226788 | 2916.75768327743 | 1239.00578945645 | 884.72790596748 | 1675.93999533176 |
| YBL033C | 659.299201077932 | -1.24858029347396 | 0.200155091295356 | -6.23806412014528 | 4.43019074714916e-10 | 1.62340210090449e-09 | 985.777085995167 | 860.017127634585 | 939.656243818643 | 393.441020333319 | 308.227786595122 | 468.675942090756 |
| YBL034C | 386.122987875098 | -0.242219373999834 | 0.203955007216071 | -1.18761180373093 | 0.234986385273548 | 0.291697932035259 | 483.387992824153 | 438.633377418212 | 333.805895691443 | 365.433354479083 | 323.924572023577 | 371.552734814118 |
| YBL035C | 509.347710754601 | -0.608696106307119 | 0.153828842798913 | -3.95696993640403 | 7.59064795896088e-05 | 0.000158662399812505 | 618.675827293806 | 575.808984213796 | 652.171430568964 | 388.106226837274 | 369.587947815447 | 451.735847798319 |
| YBL036C | 1359.80711908203 | -0.687627774850835 | 0.212643647022896 | -3.23370946876582 | 0.00122193691447884 | 0.00218815097385691 | 1905.43034278326 | 1872.81666762832 | 1255.8160128656 | 1093.63266668923 | 950.369008668293 | 1080.77801585748 |
| YBL037W | 719.052407322346 | -0.137927849066338 | 0.141385993139004 | -0.975541112695191 | 0.329291918605299 | 0.392969159903214 | 715.201417055861 | 735.16268312603 | 808.780804538738 | 694.856852859861 | 739.175895630895 | 621.136790722689 |
| YBL038W | 535.235937311918 | -0.176484221374368 | 0.186462552863411 | -0.946486137104689 | 0.343900689715694 | 0.407926092446231 | 599.674726946944 | 532.274210799628 | 572.763860668797 | 525.477159360433 | 405.262460152846 | 575.963205942857 |
| YBL039C | 6834.59391120646 | 0.206348324878902 | 0.177026697662443 | 1.16563392755804 | 0.243762484409099 | 0.301093786683948 | 6784.91291185725 | 6866.99444797052 | 5388.68592405856 | 8122.22309772852 | 6254.45550299269 | 7590.29158263126 |
| YBL039W-B | 294.177064322498 | 1.3388064724457 | 0.204298144662296 | 6.55319936780993 | 5.6317247517412e-11 | 2.21646642838552e-10 | 144.408362636146 | 150.318179901747 | 205.136222242098 | 440.120463423713 | 403.83547965935 | 421.243678071933 |
| YBL040C | 6012.68468957898 | 1.98230171428553 | 0.117903407828527 | 16.8129297599989 | 1.96228742776028e-63 | 2.82831027921182e-61 | 2290.01261380373 | 2529.94532293651 | 2466.78145384315 | 10114.7684685013 | 8803.04266437643 | 9871.55761401277 |
| YBL041W | 3109.46179202692 | 0.269478432673254 | 0.182345720971748 | 1.47784346809545 | 0.139449680603868 | 0.181402051423323 | 2657.11387250509 | 2438.7687220125 | 3363.7928916043 | 3698.3455911332 | 3023.77166571789 | 3474.97800918857 |
| YBL042C | 9834.84395527976 | 1.16795184777652 | 0.103488666831014 | 11.2857947014011 | 1.54241249755547e-29 | 2.71113481277637e-28 | 5723.13142447464 | 6065.29748849453 | 6384.95682419491 | 14153.2071450074 | 13620.5288104187 | 13061.9420390884 |
| YBL043W | 235.235598348007 | -1.27191952295616 | 0.198176029845815 | -6.41813000263322 | 1.37958425500195e-10 | 5.25116401287714e-10 | 354.180510465496 | 292.422251612142 | 351.452022335924 | 157.376408133328 | 122.72032244065 | 133.262075100504 |
| YBL044W | 6.84160701217744 | 1.76230850474754 | 0.860540088716903 | 2.04790982762373 | 0.0405688357313833 | 0.0582273663540058 | 4.56026408324673 | 3.28564327654092 | 1.4705105537068 | 14.6706821141238 | 11.4158439479675 | 5.64669809747899 |
| YBL045C | 7241.58250891936 | 0.876924765664901 | 0.21257678455468 | 4.12521417849998 | 3.703899086343e-05 | 8.06157364900024e-05 | 4263.84691783569 | 4484.08166165922 | 6570.24115396197 | 9450.58667824372 | 8664.62555650732 | 10016.1130853082 |
| YBL046W | 306.064348458338 | -1.82982484409811 | 0.270812530333901 | -6.75679534415196 | 1.41077240723666e-11 | 5.86931997006863e-11 | 547.231689989607 | 542.131140629251 | 343.364214290537 | 122.700250409035 | 161.248795765041 | 119.709999666555 |
| YBL047C | 1758.51456888421 | 0.258617913494061 | 0.136289865578029 | 1.89755791743735 | 0.0577543424409659 | 0.0806273493482792 | 1595.33238512248 | 1766.03326114074 | 1443.30610846322 | 1809.82869353327 | 1920.71574424553 | 2015.8712208 |
| YBL048W | 128.291016864852 | 0.0852043666049695 | 0.314347075140139 | 0.271051882913129 | 0.786351128496261 | 0.823820613701623 | 164.929551010757 | 118.283157955473 | 90.436399052968 | 104.028473172878 | 144.12502984309 | 147.94349015395 |
| YBL049W | 254.501010540535 | 0.096664204976829 | 0.223782487868484 | 0.431956074389692 | 0.665773338622091 | 0.718981997053427 | 294.897077383288 | 246.423245740569 | 196.313158919857 | 261.404881306205 | 289.677040179675 | 238.290659713613 |
| YBL050W | 1631.31046895741 | 0.465435902799839 | 0.176472685977636 | 2.63743876408626 | 0.0083534706087941 | 0.0133680262444211 | 1492.72644324943 | 1471.96818789033 | 1146.26297661445 | 1811.16239190728 | 2133.33583777642 | 1732.40697630655 |
| YBL051C | 502.748383511688 | -2.17975010460646 | 0.2655226428403 | -8.20928144315542 | 2.22516099861301e-16 | 1.47119207308909e-15 | 996.41770218941 | 894.516382038265 | 580.851668714185 | 177.381883743496 | 158.394834778049 | 208.927829606723 |
| YBL052C | 47.611550450632 | -2.2833082174384 | 0.514179133329277 | -4.44068626949936 | 8.96724471876001e-06 | 2.11112701436942e-05 | 85.1249295539389 | 110.069049764121 | 41.9095507806437 | 21.33917398418 | 11.4158439479675 | 15.8107546729412 |
| YBL053W | 17.3230133715311 | -0.53230380562941 | 0.546415806978525 | -0.974173511877065 | 0.329970394200457 | 0.393706397495247 | 21.2812323884847 | 18.071038020975 | 22.057658305602 | 22.6728723581913 | 8.56188296097562 | 11.293396194958 |
| YBL054W | 1045.85367310182 | -3.41446059879107 | 0.255773979942591 | -13.3495228856252 | 1.19209986275422e-40 | 4.71460957916088e-39 | 2176.76605573644 | 2208.77369265463 | 1352.1344541334 | 189.385169109598 | 152.686912804065 | 195.375754172773 |
| YBL055C | 2035.12601896175 | 0.795704690374595 | 0.14229328398985 | 5.59200454205101 | 2.24462946932737e-08 | 6.85113728849757e-08 | 1507.16727951304 | 1624.75060024948 | 1331.5473063815 | 2811.43617241572 | 2428.72079993008 | 2507.13395528067 |
| YBL056W | 4674.5573660599 | 1.10038594265558 | 0.147399955296955 | 7.46530716674044 | 8.3105514817476e-14 | 4.27117566645126e-13 | 2681.43528094907 | 2831.40309355913 | 3407.17295293865 | 6709.8365196506 | 6434.25504517317 | 5983.24130408874 |
| YBL057C | 2376.94192848181 | 1.03496149494652 | 0.133724348538866 | 7.73951420406964 | 9.97973895909902e-15 | 5.57044637596525e-14 | 1676.65709460705 | 1613.25084878159 | 1388.16196269922 | 3126.18898868237 | 3063.72711953577 | 3393.66555658487 |
| YBL058W | 943.028834860031 | -2.49833759439311 | 0.156008433017216 | -16.0141188913641 | 1.01839126063394e-57 | 1.04845805023361e-55 | 1557.33018442876 | 1476.07524198601 | 1774.9062383241 | 266.73967480225 | 272.553274257724 | 310.568395361344 |
| YBL059C-A | 268.104988451899 | -0.231557810932313 | 0.186877962956234 | -1.23908569672574 | 0.215313763399544 | 0.269547398071693 | 318.458441813396 | 292.422251612142 | 258.074602175543 | 277.40926179434 | 218.328015504878 | 243.937357811092 |
| YBL059W | 146.07937249179 | -0.178373269253814 | 0.189026695078911 | -0.943640628004157 | 0.345353323352296 | 0.409424539437579 | 151.248758761016 | 160.17510973137 | 154.403608139214 | 128.03504390508 | 131.282205401626 | 151.331509012437 |
| YBL060W | 180.939493748225 | -2.2970372490378 | 0.239265846143567 | -9.60035578023744 | 7.96689437216702e-22 | 7.93752333300695e-21 | 319.978529841145 | 313.778932909657 | 269.103431328344 | 69.352315448585 | 45.6633757918699 | 67.7603771697479 |
| YBL061C | 931.172619723578 | -2.62568504418436 | 0.249200586396788 | -10.5364320451623 | 5.8683215991692e-26 | 8.15030704330009e-25 | 1773.94272838298 | 1720.03425526917 | 1315.37169029073 | 273.408166672306 | 186.934444647968 | 317.344433078319 |
| YBL062W | 4.19130002752918 | -1.50561624734374 | 1.01655310267622 | -1.48109945597529 | 0.13858007283143 | 0.18041556651639 | 7.60044013874454 | 5.7498757339466 | 5.14678693797379 | 2.6673967480225 | 2.85396098699187 | 1.1293396194958 |
| YBL063W | 91.4441052410024 | -1.65428124657207 | 0.41229087451038 | -4.0124129561117 | 6.01012431040431e-05 | 0.000127724987802367 | 189.250959454739 | 144.5683041678 | 83.083846284434 | 41.3446495943488 | 32.8205513504065 | 57.5963205942857 |
| YBL064C | 1555.02594563732 | 1.80165038533678 | 0.32648551616305 | 5.51831642184401 | 3.42262755612827e-08 | 1.02678826683848e-07 | 512.269665351382 | 507.631886225572 | 1059.50285394575 | 2121.9141130519 | 2758.35329392764 | 2370.48386132168 |
| YBL065W | 11.8427009200617 | 0.855593350776994 | 0.616237119013477 | 1.38841579706639 | 0.165010468800631 | 0.212352758063669 | 7.60044013874454 | 5.7498757339466 | 11.7640844296544 | 14.6706821141238 | 19.9777269089431 | 11.293396194958 |
| YBL066C | 501.178348694347 | -0.933722986195271 | 0.203789839314347 | -4.5817936229637 | 4.6100473692535e-06 | 1.11904069000667e-05 | 582.953758641706 | 584.844503224283 | 805.839783431325 | 312.085419518633 | 366.733986828456 | 354.612640521681 |
| YBL067C | 779.247603954919 | -1.04720761542408 | 0.135360670310378 | -7.73642456869386 | 1.02251602847405e-14 | 5.7025270513179e-14 | 1092.94329195147 | 1062.08418914185 | 997.006155413208 | 514.807572368343 | 460.914699399187 | 547.729715455462 |
| YBL068W | 3295.17692450184 | 0.719188449106848 | 0.106418444185672 | 6.75811843153853 | 1.39795243780243e-11 | 5.82345504918853e-11 | 2462.54260495323 | 2648.22848089198 | 2360.90469397626 | 4263.83370171397 | 4002.6802842561 | 4032.87178121949 |
| YBL069W | 1358.51139239439 | -0.35720794323458 | 0.12107169169428 | -2.95038367958524 | 0.00317379527460383 | 0.00539304064738812 | 1612.81339744159 | 1491.68204754958 | 1471.9810642605 | 1181.65675937397 | 1277.14754167886 | 1115.78754406185 |
| YBL070C | 5.46974121103611 | 1.35110053556745 | 0.882667316326555 | 1.53070189705267 | 0.125843084722988 | 0.165192926029812 | 3.04017605549782 | 4.10705409567614 | 2.20576583056019 | 5.334793496045 | 5.70792197398374 | 12.4227358144538 |
| YBL071C | 6.13766704683369 | 0.742584520021057 | 0.913764497158631 | 0.812665103897272 | 0.416410093735165 | 0.481690006771229 | 8.360484152619 | 2.46423245740569 | 2.94102110741359 | 12.0032853661013 | 4.28094148048781 | 6.77603771697479 |
| YBL071C-B | 5.56045667709873 | -0.281124503309126 | 0.836077206872886 | -0.336242276428746 | 0.736688174910723 | 0.782305079272295 | 4.56026408324673 | 5.7498757339466 | 8.08780804538738 | 5.334793496045 | 2.85396098699187 | 6.77603771697479 |
| YBL071W-A | 686.756687180699 | 1.73614504526344 | 0.240296541001765 | 7.2250105558144 | 5.01062916227766e-13 | 2.38787220768059e-12 | 288.056681258418 | 251.35171065538 | 411.742955037903 | 1068.29239758301 | 917.548457317887 | 1183.5479212316 |
| YBL072C | 21749.7879403009 | 0.257350792070554 | 0.216093437549829 | 1.19092368092489 | 0.233683547078084 | 0.290302908704932 | 22823.361692636 | 22357.9810860418 | 14263.9523709559 | 23342.3889419449 | 24174.4765403147 | 23536.5670099119 |
| YBL074C | 78.8239141995512 | -0.886315116468631 | 0.287613600419802 | -3.08161754233793 | 0.00205879174288419 | 0.00357997942207691 | 101.845897859177 | 114.997514678932 | 90.436399052968 | 66.6849187005625 | 41.3824343113821 | 57.5963205942857 |
| YBL075C | 537.690524511317 | -0.0474013579076284 | 0.145612399644534 | -0.325531053834314 | 0.744779183789281 | 0.788804341289562 | 536.591073795365 | 495.310723938543 | 607.320858680907 | 537.480444726534 | 526.5558021 | 522.884243826554 |
| YBL076C | 16567.1993279951 | -0.084250063871882 | 0.111434712204881 | -0.756048651312368 | 0.449620026864205 | 0.514599522541245 | 17354.8450128093 | 18003.682333806 | 15794.0186020879 | 16968.6444125451 | 15622.5824427935 | 15659.4231639287 |
| YBL077W | 1.67314399674371 | -0.622939761076473 | 2.05762089828093 | -0.30274758659232 | 0.762082229679196 | 0.80318889910055 | 5.32030809712118 | 0 | 0.735255276853398 | 0 | 2.85396098699187 | 1.1293396194958 |
| YBL078C | 161.688358602865 | 0.351138237694479 | 0.426125056174565 | 0.824026263197798 | 0.409924635337015 | 0.47478056871355 | 119.326910178289 | 77.2126169987115 | 229.39964637826 | 186.717772361575 | 214.04707402439 | 143.426131675966 |
| YBL079W | 1932.97835431339 | -1.03775171787652 | 0.131467341169302 | -7.89360847071606 | 2.93572179449371e-15 | 1.71386962728048e-14 | 2571.22889893728 | 2770.61869294313 | 2457.22313524406 | 1396.38219758978 | 1187.24777058862 | 1215.16943057748 |
| YBL080C | 141.555819777208 | -1.32843056888904 | 0.420351367890476 | -3.16028606152929 | 0.00157614307936725 | 0.00278476273842985 | 163.409462983008 | 188.924488401103 | 255.868836344983 | 84.0229975627088 | 38.5284733243903 | 118.580660047059 |
| YBL081W | 853.435624221193 | -0.877520600824614 | 0.148344578934717 | -5.91542075299424 | 3.31028262659517e-09 | 1.10161586024096e-08 | 1150.70663700592 | 1204.18826085225 | 960.978646847392 | 598.830569931051 | 610.747651216261 | 595.161979474285 |
| YBL082C | 1484.6090780499 | 0.6864589532559 | 0.104240777057592 | 6.58532076057568 | 4.53904377573135e-11 | 1.80614956622046e-10 | 1175.78808946378 | 1129.43987631094 | 1108.76495749492 | 1905.85497646208 | 1753.75902650651 | 1834.04754206118 |
| YBL083C | 46.8056851057718 | 1.02573473405163 | 0.329604907764202 | 3.11201292787012 | 0.0018581636168278 | 0.003256430483314 | 30.4017605549782 | 28.749378669733 | 33.0864874584029 | 53.34793496045 | 79.9109076357724 | 55.3376413552941 |
| YBL084C | 359.499507331246 | -0.535149222703441 | 0.174438345808514 | -3.06784165042983 | 0.0021561083669794 | 0.00374618774932451 | 411.943855519954 | 376.206155163935 | 488.209503830657 | 284.077753664396 | 288.250059686179 | 308.309716122353 |
| YBL085W | 438.206328795775 | -2.95885939410046 | 0.410272330237793 | -7.21193991411878 | 5.51603153357974e-13 | 2.61336599903566e-12 | 1059.50135534099 | 922.444349888862 | 347.775745951657 | 96.02628292881 | 98.4616540512196 | 105.028584613109 |
| YBL086C | 98.0551041734507 | -3.16397129519865 | 0.724221369602811 | -4.36879030086323 | 1.24936659710518e-05 | 2.89097101278066e-05 | 250.054480564695 | 248.887478197974 | 30.1454663509893 | 20.0054756101688 | 25.6856488829268 | 13.5520754339496 |
| YBL087C | 68209.1987255203 | 1.34541386499458 | 0.207866391646226 | 6.47249348169944 | 9.63986809578398e-11 | 3.71725234656688e-10 | 43948.7850582764 | 43241.5297517359 | 28385.2652182023 | 94124.42904747 | 97447.070920344 | 102108.112357093 |
| YBL088C | 751.33914076808 | -0.825735746570819 | 0.186634331698707 | -4.42435075612909 | 9.67327477971989e-06 | 2.26992981987204e-05 | 941.694533190449 | 932.301279718485 | 1006.5644740123 | 554.81852358868 | 637.860280592683 | 434.795753505882 |
| YBL089W | 1096.85286913352 | 0.835282167459923 | 0.121689939968109 | 6.86401988265276 | 6.69493063488734e-12 | 2.87953051046945e-11 | 800.3263466098 | 802.518370295119 | 760.253956266414 | 1412.38657807791 | 1506.89140113171 | 1298.74056242017 |
| YBL090W | 687.090047411053 | 0.522356448478759 | 0.211885433012363 | 2.46527777324022 | 0.0136907070670341 | 0.0211978816034336 | 623.996135390927 | 522.417280970006 | 546.294670702075 | 889.576815465504 | 616.455573190244 | 923.799808747563 |
| YBL091C | 2765.1472357369 | -0.375028892393945 | 0.207625090119478 | -1.80627925159784 | 0.0708747310865621 | 0.0976824279276332 | 2725.51783375379 | 2770.61869294313 | 3871.85428791 | 2123.24781142591 | 2353.0908337748 | 2746.55395461378 |
| YBL091C-A | 205.885351350771 | 0.559816490810407 | 0.297642285658955 | 1.88083655375452 | 0.059994156030717 | 0.0835205185694849 | 133.007702428029 | 131.425731061637 | 234.546433316234 | 224.06132683389 | 273.98025475122 | 238.290659713613 |
| YBL092W | 75918.9218013729 | -0.2582337935076 | 0.227290943271412 | -1.13613762955455 | 0.255898968588554 | 0.314467735935081 | 94041.0058807001 | 93782.1160423074 | 60263.7282567351 | 66518.2064038111 | 61955.212086113 | 78953.2621385707 |
| YBL093C | 718.732522613834 | -2.37174270873078 | 0.225070167063513 | -10.5377924567919 | 5.78406645903036e-26 | 8.05052683546586e-25 | 1351.35825666878 | 1310.97166733983 | 952.890838802004 | 232.063517077958 | 194.069347115447 | 271.041508678991 |
| YBL094C | 22.7606745616371 | -0.249401749197495 | 0.43946869752068 | -0.567507425681345 | 0.570369482981089 | 0.630761545884969 | 26.6015404856059 | 23.8209137549216 | 23.5281688593087 | 16.004380488135 | 28.5396098699187 | 18.0694339119328 |
| YBL095W | 813.954778510154 | 1.24973559182024 | 0.137023412347947 | 9.12059895754715 | 7.47102739041602e-20 | 6.55711551478191e-19 | 506.189313240387 | 496.132134757678 | 444.094187219453 | 1088.29787319318 | 1104.48290196585 | 1244.53226068437 |
| YBL096C | 19.1760079772319 | 0.221653391519599 | 0.593601657827043 | 0.373404266307123 | 0.70884758470551 | 0.757427583920913 | 12.1607042219913 | 12.3211622870284 | 28.6749557972825 | 25.3402691062138 | 12.8428244414634 | 23.7161320094118 |
| YBL097W | 67.3243669524457 | -1.94477890664865 | 0.442980346009464 | -4.39021488011366 | 1.13238704644908e-05 | 2.63249547787409e-05 | 126.167306303159 | 137.997017614718 | 56.6146563177117 | 30.6750626022588 | 24.2586683894309 | 28.2334904873949 |
| YBL098W | 1526.38781796467 | 1.59547397335078 | 0.188965750722042 | 8.44319125161275 | 3.08789258461404e-17 | 2.20088695646227e-16 | 634.636751585169 | 717.91305592419 | 924.215883004722 | 2476.67788053889 | 2227.51655034716 | 2177.3667863879 |
| YBL099W | 19657.7636730087 | 0.923830868729411 | 0.0823335696684885 | 11.2205856304927 | 3.23123512068992e-29 | 5.55909575405698e-28 | 13579.7063958949 | 13648.562170751 | 13483.1112669376 | 26608.6162598985 | 25136.2613929309 | 25490.3245516397 |
| YBL100C | 1.05529190755716 | -0.567596554155292 | 2.18815292086353 | -0.259395286656336 | 0.795330265998891 | 0.831749775115899 | 3.04017605549782 | 0 | 0.735255276853398 | 0 | 1.42698049349594 | 1.1293396194958 |
| YBL100W-B | 10.991370624595 | -2.21146635394424 | 1.00334016010088 | -2.20410429272749 | 0.0275170103341785 | 0.0407477920154068 | 26.6015404856059 | 24.6423245740569 | 2.94102110741359 | 2.6673967480225 | 5.70792197398374 | 3.38801885848739 |
| YBL100W-C | 4.38822337589916 | 0.536940947231577 | 0.903523177274421 | 0.59427468020391 | 0.552328410661566 | 0.614371818135983 | 3.80022006937227 | 2.46423245740569 | 4.41153166112039 | 4.00109512203375 | 7.13490246747968 | 4.51735847798319 |
| YBL101C | 414.205196155386 | -0.826611290577995 | 0.275603161319095 | -2.99928087407147 | 0.00270617705755198 | 0.00463487309091157 | 422.584471714197 | 468.204166907081 | 697.757257733875 | 261.404881306205 | 371.014928308943 | 264.265470962017 |
| YBL102W | 2391.26243313182 | 0.938165938341398 | 0.113503271880942 | 8.26554092048974 | 1.39062730397296e-16 | 9.35644055349441e-16 | 1644.73524602432 | 1728.24836346052 | 1547.7123577764 | 3306.23826917389 | 2965.26546548455 | 3155.37489687126 |
| YBL103C | 364.992184913196 | -2.97456812833691 | 0.193300359950434 | -15.3883217242826 | 1.96051590536231e-53 | 1.56986495829382e-51 | 633.11666355742 | 722.020110019866 | 588.204221482719 | 84.0229975627088 | 75.6299661552846 | 86.9591507011764 |
| YBL104C | 485.79613007498 | -1.38291322812666 | 0.141970017550629 | -9.74088227912977 | 2.01794637681488e-22 | 2.11103229032602e-21 | 693.920184667377 | 737.626915583436 | 676.434854705126 | 265.405976428239 | 251.148566855285 | 290.24028221042 |
| YBL105C | 1058.21679788016 | -0.728910711732968 | 0.170448448964891 | -4.27642912657486 | 1.89914979934257e-05 | 4.28448194731683e-05 | 1460.8045946667 | 1389.82710597681 | 1109.50021277178 | 724.198217088109 | 846.19943264309 | 818.771224134454 |
| YBL106C | 472.049509098066 | 0.0897224236580448 | 0.160521559717934 | 0.5589431339672 | 0.576200530349585 | 0.635692573540977 | 441.585572061058 | 412.348231205885 | 518.354970181646 | 480.13141464405 | 463.768660386179 | 516.10820610958 |
| YBL107C | 316.051378939395 | -1.28135014627382 | 0.207689625190293 | -6.16954335152659 | 6.84874974823403e-10 | 2.44878670711389e-09 | 450.706100227551 | 477.239685917568 | 416.889741975877 | 189.385169109598 | 144.12502984309 | 217.962546562689 |
| YBL109W | 10.772004603843 | -0.284797887457086 | 0.6188684119469 | -0.460191345945642 | 0.645378882141585 | 0.700104938881137 | 8.360484152619 | 14.7853947444341 | 12.4993397065078 | 8.0021902440675 | 8.56188296097562 | 12.4227358144538 |
| YBL113C | 200.839779498342 | -1.06974577307876 | 0.235486186707661 | -4.54271134980318 | 5.55352714725304e-06 | 1.33605998060398e-05 | 290.336813300042 | 308.850467994846 | 217.635561948606 | 114.698060164968 | 125.574283427642 | 147.94349015395 |
| YBL113W-A | 1.58327180105629 | -0.426377538091258 | 1.92977192346131 | -0.220947114478943 | 0.825133611779352 | 0.857130280857766 | 0 | 2.46423245740569 | 2.94102110741359 | 2.6673967480225 | 1.42698049349594 | 0 |
| YBR001C | 682.247459623141 | 0.0143641997031967 | 0.129848067708335 | 0.110623130222173 | 0.911915204794576 | 0.929396923051166 | 719.001637125234 | 636.593384829803 | 680.111131089393 | 694.856852859861 | 719.198168721952 | 643.723583112605 |
| YBR002C | 739.418279246379 | 0.137756000527009 | 0.160956772790644 | 0.855857123242575 | 0.3920768139258 | 0.457375937971715 | 771.444674082571 | 711.341769371108 | 630.113772263362 | 845.564769123133 | 686.377617371545 | 791.667073266554 |
| YBR003W | 1666.91213696544 | 1.31590760330883 | 0.266851347647665 | 4.93123836513755 | 8.17099539419543e-07 | 2.14216152492933e-06 | 770.684630068697 | 746.662434593923 | 1348.45817774913 | 2467.34199192081 | 2256.05616021707 | 2412.26942724302 |
| YBR004C | 3273.87621436317 | 0.393479471964346 | 0.252439215910753 | 1.55870976918046 | 0.119065088523758 | 0.157154286561884 | 2670.79466475483 | 2791.97537424064 | 3027.04597480544 | 2932.80272445074 | 5122.85997165041 | 3097.77857627697 |
| YBR005W | 3944.25454214643 | 0.845933616953227 | 0.325342034992247 | 2.6001362442249 | 0.00931867550068903 | 0.01476329489435 | 2283.17221767886 | 2327.87826142924 | 3848.32611905069 | 3484.9538512914 | 6902.30464703984 | 4818.89215638857 |
| YBR006W | 1703.68348228772 | 0.481913978058157 | 0.236798929452349 | 2.03511890519392 | 0.0418389101826755 | 0.0598648072898374 | 1451.68406650021 | 1386.54146270027 | 1426.39523709559 | 1572.43038295926 | 2668.4535228374 | 1716.59622163361 |
| YBR007C | 334.292528334728 | -1.18539726385875 | 0.329916373452596 | -3.59302344243631 | 0.000326863168842102 | 0.000633981612772092 | 580.673626600083 | 543.773962267522 | 269.103431328344 | 209.390644719766 | 176.945581193496 | 225.86792389916 |
| YBR008C | 827.111839568943 | 0.66555319487968 | 0.194632204417161 | 3.41954301382303 | 0.000627264146657821 | 0.00116374006156254 | 576.873406530711 | 556.916535373685 | 784.517380402576 | 990.937891890359 | 1105.90988245935 | 947.515940756974 |
| YBR009C | 12143.5540472245 | 0.934856702891636 | 0.177469970790816 | 5.26768950671407 | 1.38151515219374e-07 | 3.93349748776497e-07 | 7978.18201364015 | 7166.80939695487 | 9878.15464452541 | 17786.201515814 | 15053.2172258886 | 14998.7594865237 |
| YBR010W | 24852.8239014808 | 1.45262516068778 | 0.127902084151589 | 11.3573220508755 | 6.82033709628328e-30 | 1.23222023416416e-28 | 14328.3497495612 | 13264.9633182148 | 12308.9085898027 | 39772.2192113895 | 34795.4923534049 | 34647.0101865116 |
| YBR011C | 29665.2778221847 | 0.91937219089642 | 0.259259441405242 | 3.54614738777969 | 0.000390907504797264 | 0.00074879683287509 | 16311.3045817597 | 17050.02437279 | 28199.9808884352 | 36391.293833271 | 35450.4763999195 | 44588.5868569331 |
| YBR012W-B | 1.40935663136069 | -2.35393404370377 | 1.97181520438219 | -1.19379039094148 | 0.232559971390857 | 0.289017814608373 | 2.28013204162336 | 4.10705409567614 | 0.735255276853398 | 1.33369837401125 | 0 | 0 |
| YBR014C | 887.210150483984 | -0.448534138003125 | 0.213302428323041 | -2.10280839992985 | 0.0354825214604674 | 0.0514853767768661 | 1083.0627197711 | 907.658955144428 | 1082.2957675282 | 785.548342292626 | 573.646158385366 | 891.048959782185 |
| YBR015C | 6747.91147067993 | 0.672087942651204 | 0.156647133302491 | 4.29045797699584 | 1.78305025213707e-05 | 4.0351932782139e-05 | 4624.10778041218 | 4936.67902300273 | 6050.41567322661 | 8515.66411806183 | 8673.1874394683 | 7687.4147899079 |
| YBR016W | 595.639444018732 | -0.674315780480737 | 0.38853755026683 | -1.73552280858735 | 0.0826482451247611 | 0.112522358916709 | 520.630149504001 | 455.061593800917 | 1221.25901485349 | 426.7834796836 | 478.038465321139 | 472.063960949243 |
| YBR017C | 3852.60734049413 | 0.560710562694219 | 0.120609053761333 | 4.64899230371028 | 3.33560657481169e-06 | 8.2136462582493e-06 | 2980.89262241561 | 2962.00741380164 | 3396.14412378585 | 4850.66098627892 | 4546.35985227805 | 4379.5790444047 |
| YBR018C | 55.4629620226077 | 0.32120659055415 | 0.467764939045223 | 0.686683767299405 | 0.492282028398842 | 0.555874170646742 | 30.4017605549782 | 38.6063084993558 | 78.6723146233136 | 50.6805382124275 | 81.3378881292684 | 53.0789621163025 |
| YBR019C | 80.0503394556646 | 2.05342393471618 | 0.344079310377042 | 5.96787970908812 | 2.40356457854601e-09 | 8.13224822975973e-09 | 25.081452457857 | 22.9995029357864 | 44.8505718880573 | 132.036139027114 | 141.271068856098 | 114.063301569076 |
| YBR020W | 140.084962495021 | 0.853666657023557 | 0.194803387054968 | 4.38219617189036 | 1.17488945973654e-05 | 2.72544099994679e-05 | 89.6851936371856 | 100.212119934498 | 109.553036251156 | 176.048185369485 | 179.799542180488 | 185.211697597311 |
| YBR021W | 1828.00528722245 | 0.860306055924206 | 0.235862196754477 | 3.64749445974062 | 0.000264810019624832 | 0.000520314385727555 | 1564.9306245675 | 1404.61250072124 | 926.421648835282 | 2603.37922606996 | 2121.91999382846 | 2346.76772931227 |
| YBR022W | 517.549110074486 | 0.573404365057393 | 0.140519854307988 | 4.08059322208389 | 4.49209086643609e-05 | 9.70219825497984e-05 | 407.383591436707 | 429.597858407725 | 411.00769976105 | 685.520964241783 | 587.915963320326 | 583.868583279327 |
| YBR023C | 4558.96412359926 | -0.00503488206504079 | 0.123331793491579 | -0.0408238777893435 | 0.967436303500628 | 0.974649248913494 | 4419.65594067995 | 5055.78359177733 | 4225.51207607648 | 4701.28676838966 | 4512.11232043415 | 4439.43404423798 |
| YBR024W | 348.386836288158 | 0.547508184385492 | 0.356297937600059 | 1.53665830364717 | 0.124377006881396 | 0.163367611711773 | 214.332411912596 | 229.995029357864 | 405.125657546222 | 422.782384561566 | 278.261196231708 | 539.824338118991 |
| YBR025C | 21661.628788958 | 0.392018338140268 | 0.114796086969651 | 3.41491028560849 | 0.000638030226418463 | 0.00118202343574697 | 18732.8048099637 | 19510.9711869191 | 17965.2274346359 | 25876.4158525663 | 25460.1859649545 | 22424.1674847086 |
| YBR026C | 1119.89159483215 | -0.263011074980483 | 0.226692331559046 | -1.16021161003401 | 0.24596266117986 | 0.303522416364644 | 1061.78148738261 | 1090.01215699245 | 1513.89061504115 | 1014.94446262256 | 840.491510669106 | 1198.22933628504 |
| YBR027C | 41.1304983397411 | 1.05814187609069 | 0.357335729667119 | 2.96119807855882 | 0.00306444784392322 | 0.00521816978621319 | 24.3214084439825 | 34.4992544036796 | 21.3224030287486 | 49.3468398384163 | 64.2141222073171 | 53.0789621163025 |
| YBR028C | 434.810207423512 | 0.0119446455152041 | 0.202033616266903 | 0.0591220695640283 | 0.95285488041605 | 0.963250741019093 | 376.221786867855 | 407.419766291074 | 514.678693797379 | 472.129224399983 | 462.341679892683 | 376.070093292101 |
| YBR029C | 3689.75630769599 | 1.10043701425881 | 0.140340861563621 | 7.84117328337723 | 4.46355972129008e-15 | 2.5574777696367e-14 | 2188.92675995843 | 2252.3084660688 | 2599.12740367676 | 5398.81101799754 | 5135.70279609187 | 4563.66140238252 |
| YBR030W | 445.647410450679 | -0.986501065585218 | 0.191143008055791 | -5.16106278549973 | 2.45551802026407e-07 | 6.7916801191611e-07 | 551.031910058979 | 574.98757339466 | 652.171430568964 | 294.747340656486 | 249.721586361789 | 351.224621663193 |
| YBR031W | 57777.903219065 | 0.469456481037148 | 0.176745436134405 | 2.65611656688071 | 0.007904628572127 | 0.0126998813274252 | 53776.914201687 | 53290.6697130363 | 38311.2114557232 | 67147.7120363444 | 65298.627382374 | 68842.2845252249 |
| YBR032W | 29.3104731627873 | 2.71094977405272 | 0.661811968215792 | 4.0962537763729 | 4.19889630305938e-05 | 9.0871009081225e-05 | 6.84039612487009 | 11.4997514678932 | 5.14678693797379 | 44.0120463423713 | 21.404707402439 | 86.9591507011764 |
| YBR033W | 87.9550839409564 | -1.03731565019049 | 0.265439962563522 | -3.90791062571166 | 9.30977298427447e-05 | 0.000192548429770422 | 120.846998206038 | 127.31867696596 | 107.347270420596 | 61.3501252045175 | 44.236395298374 | 66.6310375502521 |
| YBR034C | 6895.50322805075 | 0.866842624540218 | 0.10936231213327 | 7.92633776326783 | 2.25703178013094e-15 | 1.34057766720964e-14 | 4606.62676809307 | 4978.57097477862 | 5067.37936807362 | 9365.229982307 | 8352.11682843171 | 9003.0954466205 |
| YBR035C | 3289.88792403083 | 0.85748131034039 | 0.171070070789856 | 5.01245662891979 | 5.37394906254637e-07 | 1.43615301275961e-06 | 2123.56297476523 | 2114.31144845408 | 2782.20596761326 | 4135.79865780889 | 3965.57879142521 | 4617.86970411832 |
| YBR036C | 6763.66480511696 | 0.806231763359383 | 0.129299994084254 | 6.23535808388384 | 4.50745824387161e-10 | 1.64799177958012e-09 | 4950.16666236432 | 5127.2463330421 | 4686.51713466356 | 8058.20557577597 | 9620.7024871496 | 8139.15063770622 |
| YBR037C | 1038.36590245852 | 2.20323417874089 | 0.25451981653607 | 8.65643472766158 | 4.86749579394697e-18 | 3.72734093501063e-17 | 288.056681258418 | 312.136111271387 | 511.002417413112 | 1621.77722279768 | 1758.03996798699 | 1739.18301402353 |
| YBR038W | 2706.22278784619 | 0.106664373983243 | 0.203029427680761 | 0.525364107073976 | 0.59933010008305 | 0.657519456890843 | 2945.17055376351 | 2879.04492106898 | 1994.74756610327 | 3080.84324396599 | 2578.55375174716 | 2758.97669042823 |
| YBR039W | 3783.46030545614 | -0.612233352597675 | 0.153904862235966 | -3.97799876951906 | 6.94977313443933e-05 | 0.000146066845592915 | 4633.22830857867 | 4571.97261930669 | 4519.61418681784 | 2988.81805615921 | 2547.16018089025 | 3439.9684809842 |
| YBR040W | 23.1929647084307 | 1.97299159750821 | 0.627783100146586 | 3.14279182897329 | 0.00167344806444867 | 0.00294545024315172 | 3.04017605549782 | 8.21410819135229 | 16.9108713676282 | 40.0109512203375 | 37.1014928308943 | 33.8801885848739 |
| YBR041W | 2832.04758762964 | 0.566819629090711 | 0.143145314657982 | 3.95974978604796 | 7.50283322659319e-05 | 0.000156877422010585 | 2182.84640784743 | 2151.27493531516 | 2513.10253628492 | 3446.27659844507 | 3668.76684877805 | 3030.01819910723 |
| YBR042C | 1665.43964284451 | 0.872009176295836 | 0.127764172999872 | 6.82514632874982 | 8.78357379889688e-12 | 3.72842013479353e-11 | 1190.98896974127 | 1253.47291000036 | 1085.97204391247 | 2225.94258622478 | 2247.4942772561 | 1988.7670699321 |
| YBR043C | 1345.55203060863 | 0.0207212314589946 | 0.128542575300873 | 0.161201309453257 | 0.871934849812132 | 0.896707885179262 | 1292.83486760045 | 1251.83008836209 | 1463.15800093826 | 1400.38329271181 | 1277.14754167886 | 1387.95839236034 |
| YBR044C | 459.279475236466 | -0.628014772761227 | 0.186095817479294 | -3.37468504809949 | 0.000739002133553914 | 0.00135822268014471 | 487.9482569074 | 518.31022687433 | 666.876536106032 | 349.428973990948 | 358.17210386748 | 374.940753672605 |
| YBR045C | 99.2336268630834 | -2.2150677211706 | 0.317249919571424 | -6.98209072570593 | 2.90819211552912e-12 | 1.28930513064401e-11 | 192.291135510237 | 165.103574646181 | 133.081205110465 | 40.0109512203375 | 24.2586683894309 | 40.6562263018487 |
| YBR046C | 329.744385952593 | 0.587396954778043 | 0.260235915820477 | 2.25717097090955 | 0.0239973935740259 | 0.0358386126458973 | 209.012103815475 | 240.673370006622 | 340.423193183123 | 357.431164235015 | 475.184504334147 | 355.741980141176 |
| YBR047W | 86.8547480736347 | -0.308606900870769 | 0.322469238114297 | -0.957011908098303 | 0.338561244571286 | 0.402402094976976 | 112.486514053419 | 101.854941572768 | 73.5255276853398 | 88.0240926847425 | 89.899771090244 | 55.3376413552941 |
| YBR048W | 56356.9189560784 | 0.548974182736947 | 0.136434552406639 | 4.02371813483689 | 5.72864743050337e-05 | 0.00012210321141717 | 46903.0761402064 | 50242.4141632254 | 40141.2618398113 | 64326.9399753106 | 65935.0606824732 | 70592.7609354433 |
| YBR049C | 527.165955074616 | -3.03531137888253 | 0.492680057917235 | -6.16081639617008 | 7.23708620914713e-10 | 2.57627558466127e-09 | 1298.15517569757 | 1186.11722283127 | 334.541150968296 | 142.705726019204 | 121.293341947155 | 80.1831129842017 |
| YBR050C | 36.2058850456598 | -0.374002394130428 | 0.356613099752215 | -1.04876235446846 | 0.294287507707032 | 0.356508923232688 | 41.802420763095 | 42.7133625950319 | 38.2332743963767 | 22.6728723581913 | 35.6745123373984 | 36.1388678238655 |
| YBR051W | 3.90347472402075 | 2.0816005258248 | 1.64506115130125 | 1.26536361531499 | 0.205741011822399 | 0.25876211027343 | 0 | 0 | 4.41153166112039 | 5.334793496045 | 11.4158439479675 | 2.2586792389916 |
| YBR052C | 3519.72029036404 | 1.91669501130846 | 0.216280373127915 | 8.86208481883313 | 7.85318133386565e-19 | 6.44756128246235e-18 | 1292.07482358657 | 1237.86610443679 | 1891.81182734379 | 4972.02753831394 | 6003.3069361374 | 5721.23451236571 |
| YBR053C | 1435.65588367094 | 1.80983428208215 | 0.276306282092556 | 6.55010182314963 | 5.74978657589036e-11 | 2.25881985046789e-10 | 484.148036838027 | 525.702924246547 | 901.422969422266 | 2061.89768622139 | 2502.92378559187 | 2137.83989970555 |
| YBR054W | 7946.08010554851 | 0.00514284539264178 | 0.277436528036954 | 0.0185370161205188 | 0.985210448038958 | 0.988257804822252 | 10044.7416873648 | 8164.82354220418 | 5585.73433825527 | 7275.32463023137 | 10033.0998497699 | 6572.75658546554 |
| YBR055C | 817.245521629608 | 0.079048590938348 | 0.179372236112671 | 0.440695799146388 | 0.659433238211835 | 0.71320393247323 | 895.331848344107 | 842.767500432745 | 647.024643630991 | 820.224500016919 | 804.816998331708 | 893.307639021176 |
| YBR056C-B | 6.69578856547659 | -0.186983576456676 | 0.746699669718027 | -0.250413364354756 | 0.802267695526977 | 0.837789127867972 | 7.60044013874454 | 6.57128655308183 | 7.35255276853398 | 5.334793496045 | 4.28094148048781 | 9.03471695596638 |
| YBR056W | 1465.39989806101 | 0.701389264881828 | 0.19681825727183 | 3.56363924060725 | 0.000365748540662491 | 0.000704348288223551 | 1021.49915464727 | 971.728999036976 | 1354.34021996396 | 1628.44571466774 | 2059.13285211464 | 1757.25244793546 |
| YBR056W-A | 1104.86407509662 | -0.00983456505321944 | 0.26781592869922 | -0.0367213597077136 | 0.970707177543492 | 0.977495403966292 | 1267.75341514259 | 1089.19074617331 | 968.331199615926 | 852.233260993189 | 1515.45328409268 | 936.222544562016 |
| YBR057C | 158.326481561678 | -3.35758475912277 | 0.643133720930025 | -5.22066352587985 | 1.78283202714708e-07 | 5.01015967420969e-07 | 445.38579213043 | 355.670884685554 | 64.702464363099 | 22.6728723581913 | 24.2586683894309 | 37.2682074433613 |
| YBR058C | 1603.21334784786 | 0.044199770376101 | 0.146397176230525 | 0.301916823221383 | 0.762715470784254 | 0.803471259299443 | 1452.44411051408 | 1585.32288093099 | 1699.1749448082 | 1581.76627157734 | 1481.20575224878 | 1819.36612700773 |
| YBR058C-A | 1895.70395972548 | 0.931456585313322 | 0.155119308511003 | 6.00477525495963 | 1.91597113610177e-09 | 6.56124012077936e-09 | 1444.84367037534 | 1240.3303368942 | 1227.14105706832 | 2822.10575940781 | 2281.7418091 | 2358.06112550723 |
| YBR059C | 432.72842422583 | -1.78586437608525 | 0.424433764474 | -4.20763974397387 | 2.58051824422285e-05 | 5.73389562590935e-05 | 901.412200455103 | 830.446338145717 | 280.867515757998 | 220.060231711856 | 178.372561686992 | 185.211697597311 |
| YBR060C | 539.936189088707 | -0.670911257735159 | 0.245191044384584 | -2.73627961991478 | 0.0062138200486081 | 0.0101187137422225 | 750.163441694086 | 774.590402444521 | 465.416590248201 | 388.106226837274 | 415.251323607317 | 446.08914970084 |
| YBR061C | 1594.50828545529 | 0.333078347957082 | 0.118883182358534 | 2.80172806068201 | 0.00508296999028521 | 0.0083696733579563 | 1375.67966511276 | 1412.82660891259 | 1446.24712957063 | 1780.48732930502 | 1635.31964554634 | 1916.48933428437 |
| YBR062C | 515.268141337714 | 0.49943894208744 | 0.217068571271869 | 2.30083488899881 | 0.0214009655690825 | 0.0322356392663886 | 407.383591436707 | 342.52831157939 | 530.854309888154 | 641.508917899411 | 543.679568021952 | 625.654149200672 |
| YBR063C | 354.18309420126 | -1.00842084969652 | 0.195343939466438 | -5.16228377727469 | 2.43955086497267e-07 | 6.75039543951057e-07 | 489.468344935149 | 501.882010491625 | 429.389081682385 | 234.73091382598 | 194.069347115447 | 275.558867156975 |
| YBR065C | 117.466362282934 | -3.24568348216489 | 0.328465915191921 | -9.88134029148339 | 5.01574970646256e-23 | 5.51392416883325e-22 | 216.612543954219 | 243.137602464028 | 178.667032275376 | 17.3380788621463 | 18.5507464154472 | 30.4921697263865 |
| YBR066C | 100.285859101775 | -3.09673608293484 | 0.416686909510854 | -7.4318055409278 | 1.07125212118537e-13 | 5.44952255530065e-13 | 249.294436550821 | 179.888969390615 | 109.553036251156 | 22.6728723581913 | 19.9777269089431 | 20.3281131509244 |
| YBR067C | 15843.7171197807 | 2.41782873847056 | 0.161295268389147 | 14.9900785225591 | 8.5252377681795e-51 | 5.58532244084972e-49 | 5040.61190001538 | 5043.46242949031 | 4901.21167550475 | 22555.5069012783 | 31245.164885587 | 26276.3449268087 |
| YBR068C | 15814.1755858378 | 0.000219654954879172 | 0.187452585223501 | 0.001171789413399 | 0.999065047532499 | 0.999527363611875 | 17625.4206817486 | 16731.3169749655 | 13081.6618857757 | 14725.3637474582 | 18380.9357367211 | 14340.3544883576 |
| YBR069C | 6492.35308205972 | -0.0500504120458988 | 0.223446958704591 | -0.22399236192813 | 0.822763246546803 | 0.855453744005618 | 7532.79622150972 | 7590.65737962865 | 4691.66392160153 | 6641.81790257603 | 6177.39855634391 | 6319.78451069849 |
| YBR070C | 1396.57992365414 | 0.763513733877526 | 0.177512548179221 | 4.30118175705903 | 1.6988963500256e-05 | 3.85820788734804e-05 | 1063.30157541036 | 1200.9026175757 | 842.602547273994 | 1677.79255450615 | 1755.186007 | 1839.69424015865 |
| YBR071W | 2064.32649352662 | -0.654371199272911 | 0.207860857339301 | -3.14812133293933 | 0.00164323470869687 | 0.00289620117407824 | 2514.22559789669 | 2405.91228924709 | 2652.80103888706 | 1316.3602951491 | 2047.71700816667 | 1448.94273181311 |
| YBR072C-A | 1.70972466676428 | 0.748117599854028 | 1.38244912838181 | 0.541153800523363 | 0.588401578057429 | 0.647062162645046 | 1.52008802774891 | 0.821410819135229 | 1.4705105537068 | 1.33369837401125 | 2.85396098699187 | 2.2586792389916 |
| YBR072W | 1731.18031593087 | 2.76031048618866 | 0.28447145787203 | 9.70329503999094 | 2.91915796333583e-22 | 3.01491378187838e-21 | 347.340114340626 | 343.349722398526 | 644.81887780043 | 2756.75453908125 | 3226.40289579431 | 3068.41574617008 |
| YBR073W | 1297.49145361938 | -1.4683284712517 | 0.181587298679253 | -8.0860747526471 | 6.16183759409999e-16 | 3.9105360699934e-15 | 2112.16231455711 | 2051.8842261998 | 1555.06491054494 | 704.19274147794 | 626.444436644716 | 735.200092291764 |
| YBR074W | 2335.65545745535 | 0.0742996558435512 | 0.0922069858855945 | 0.805792046339506 | 0.42036276488641 | 0.485656019425232 | 2280.89208563724 | 2269.55809327064 | 2275.61508186127 | 2478.0115789129 | 2390.19232660569 | 2319.66357844437 |
| YBR076C-A | 476.683225432114 | 1.4181210734845 | 0.150412355349813 | 9.42822197143571 | 4.17099128737885e-21 | 3.93212928632838e-20 | 271.33571295318 | 248.066067378839 | 258.809857452396 | 686.854662615794 | 764.861544513822 | 630.171507678655 |
| YBR076W | 59.1798036780775 | -0.629837195883217 | 0.275137178697399 | -2.28917516296815 | 0.0220691770732547 | 0.0331958911171451 | 76.0044013874454 | 64.0700438925479 | 75.7312935159 | 45.3457447163825 | 44.236395298374 | 49.6909432578151 |
| YBR077C | 687.555775697572 | 0.802064348970895 | 0.139407030794581 | 5.75339955524019 | 8.74664266107203e-09 | 2.7891211553448e-08 | 446.905880158179 | 512.560351140383 | 544.088904871515 | 849.565864245166 | 894.716769421952 | 877.496884348235 |
| YBR078W | 64866.7670847421 | 1.01004735128926 | 0.175075675686003 | 5.76920435880983 | 7.96466723534424e-09 | 2.54853634378109e-08 | 37283.1990565975 | 39600.2155905094 | 52247.9752284793 | 87382.5837668431 | 90352.1239066822 | 82334.5049593411 |
| YBR079C | 3164.78952853141 | -2.90782220514051 | 0.849866854441863 | -3.42150325070646 | 0.000622759805132317 | 0.00115670678582136 | 8029.10496256973 | 7867.47282567722 | 859.513418641623 | 746.8710894463 | 696.366480826017 | 789.408394027563 |
| YBR080C | 2749.33264778307 | 0.441569944063239 | 0.166161610142162 | 2.65747270795851 | 0.00787289647355966 | 0.0126551689039673 | 2119.00271068198 | 2189.05983299539 | 2686.62278162232 | 3044.83338786768 | 3557.46237028537 | 2899.01480324571 |
| YBR081C | 539.350218905021 | -0.448495729555963 | 0.139075265449252 | -3.22484180135982 | 0.0012604229722496 | 0.00225271518269795 | 614.875607224433 | 676.842514967429 | 576.440137053064 | 444.121558545746 | 449.49885545122 | 474.322640188235 |
| YBR082C | 4983.13863956281 | -1.20250266471445 | 0.276124226665069 | -4.35493357188484 | 1.33107276599654e-05 | 3.06799501075109e-05 | 6192.07858103518 | 5633.2353976294 | 9017.90597060693 | 3095.51392608011 | 2211.8197649187 | 3748.27819710655 |
| YBR083W | 372.225207781815 | -1.87379879532125 | 0.582412734547781 | -3.21730395674844 | 0.00129401427397972 | 0.00230956978014103 | 836.0484152619 | 786.090153912414 | 132.345949833612 | 172.047090247451 | 176.945581193496 | 129.874056242017 |
| YBR084C-A | 21030.6874796369 | -2.26460554110772 | 0.323985042583586 | -6.98984596032227 | 2.75188218655478e-12 | 1.2233521495541e-11 | 43105.8962468897 | 39716.0345160075 | 21626.7987133659 | 7558.06868552176 | 5174.23126941626 | 9003.0954466205 |
| YBR084W | 10067.050024211 | -0.122095414008823 | 0.194467754727729 | -0.627844005191333 | 0.530106135560199 | 0.592396346527128 | 10680.1384829638 | 10533.7723445902 | 10265.6341754271 | 9926.71699776574 | 7447.41119555529 | 11548.626948964 |
| YBR085C-A | 3444.89192341723 | 1.52618515039383 | 0.212687857219095 | 7.1757042002716 | 7.19359382829186e-13 | 3.37365506654382e-12 | 1716.17938332852 | 1541.78810751682 | 2068.27309378861 | 4362.5273813908 | 6318.6696252 | 4661.91394927865 |
| YBR085W | 339.976351682608 | 0.826948576525826 | 0.140568998383503 | 5.88286596643259 | 4.032224585857e-09 | 1.33229794517924e-08 | 237.893776342704 | 248.887478197974 | 248.516283576449 | 409.445400821454 | 458.060738412195 | 437.054432744874 |
| YBR086C | 3980.61544227272 | -2.72916614241896 | 0.164909526394001 | -16.549475352312 | 1.6150392790469e-61 | 2.09502895277964e-59 | 7013.68616003346 | 7055.09752555248 | 6687.14674298166 | 1037.61733498075 | 870.458101032521 | 1219.68678905546 |
| YBR087W | 2021.74586840378 | 0.273068173864012 | 0.15773772455898 | 1.73115324585469 | 0.083424435722442 | 0.113464727755198 | 1859.06765793691 | 1925.38696005298 | 1709.46851868415 | 2360.64612199991 | 1863.63652450569 | 2412.26942724302 |
| YBR088C | 3910.88282783059 | 0.545183387661274 | 0.125877292643869 | 4.3310701732655 | 1.48386378394934e-05 | 3.39962575157026e-05 | 3246.90802727167 | 3395.71232630504 | 2899.8468119098 | 4897.34042936931 | 4325.17787578618 | 4700.31149634151 |
| YBR089C-A | 2094.79710204081 | 0.51028950689298 | 0.114720535502584 | 4.44810952683781 | 8.66293564401133e-06 | 2.04735414978022e-05 | 1697.93832699553 | 1699.49898479079 | 1785.9350674769 | 2454.0050081807 | 2645.62183494147 | 2285.7833898595 |
| YBR089W | 8.59262195540427 | 0.239065588992306 | 0.698299150068294 | 0.34235411709856 | 0.732084413717794 | 0.778282168066483 | 6.08035211099563 | 6.57128655308183 | 11.028829152801 | 12.0032853661013 | 5.70792197398374 | 10.1640565754622 |
| YBR090C | 32.7642692856999 | 1.67063658839954 | 0.356778212235635 | 4.68256337160005 | 2.83309560020852e-06 | 7.04310389534399e-06 | 15.2008802774891 | 16.4282163827046 | 15.4403608139214 | 49.3468398384163 | 47.0903562853659 | 53.0789621163025 |
| YBR091C | 915.802334497396 | 2.28430759675635 | 0.170150693915136 | 13.4252029433137 | 4.30403657692903e-41 | 1.73145325755059e-39 | 293.376989355539 | 275.994035229437 | 366.157127872992 | 1616.44242930164 | 1401.29484461301 | 1541.54858061176 |
| YBR092C | 19733.2789554361 | 0.474297492475423 | 0.256545470258605 | 1.84878529329447 | 0.0644888217830518 | 0.0893176378571159 | 19469.287459408 | 19282.6189791995 | 10803.8410380838 | 22812.9106874624 | 21555.9673347496 | 24475.0482337129 |
| YBR093C | 3559.04647611461 | -0.10738749205244 | 0.395026741309458 | -0.271848664463742 | 0.785738384913833 | 0.823444686468108 | 4664.39011314753 | 4830.71702733428 | 1579.3283346811 | 3579.6464358462 | 3431.88808685773 | 3268.30885882084 |
| YBR094W | 873.966727878308 | -0.358539410863056 | 0.168851977434923 | -2.12339480004751 | 0.0337207825860919 | 0.0490828087642262 | 932.574005023955 | 983.228750504869 | 1030.82789814846 | 868.237641481324 | 635.006319605691 | 793.925752505546 |
| YBR095C | 242.48509475851 | -3.09228550797251 | 0.305102421831442 | -10.1352375029028 | 3.85435779250541e-24 | 4.74371245582355e-23 | 516.069885420754 | 483.81097247065 | 302.9251740636 | 48.013141464405 | 44.236395298374 | 59.8549998332773 |
| YBR096W | 3654.5657693722 | 1.31926520852923 | 0.142325016403289 | 9.26938385020797 | 1.8722431210405e-20 | 1.69837327035926e-19 | 2165.36539552832 | 2309.80722340826 | 1798.43440718341 | 5292.11514807664 | 5262.70406001301 | 5098.96838202353 |
| YBR097W | 677.484502150759 | 0.39401480340632 | 0.118360009808994 | 3.32895210166145 | 0.000871733949128932 | 0.00158956041440828 | 571.55309843359 | 574.166162575525 | 610.261879788321 | 782.880945544604 | 791.974173890244 | 734.070752672269 |
| YBR098W | 54.1230993715189 | -3.04673228887463 | 0.57104507918927 | -5.335362127978 | 9.53540599686195e-08 | 2.75854787224115e-07 | 118.566866164415 | 129.782909423366 | 41.1742955037903 | 13.3369837401125 | 12.8428244414634 | 9.03471695596638 |
| YBR099C | 113.130343633006 | 1.17819714096243 | 0.237698189515725 | 4.95669379460916 | 7.17028452882036e-07 | 1.89050672576946e-06 | 65.3637851932031 | 64.0700438925479 | 78.6723146233136 | 173.380788621463 | 131.282205401626 | 166.012924065882 |
| YBR101C | 1966.6981317308 | -0.164730352927869 | 0.246283818528098 | -0.668863890093839 | 0.503582306589409 | 0.567054659815782 | 1903.15021074163 | 1683.89217922722 | 2649.86001777965 | 2036.55741711518 | 1455.52010336585 | 2071.20886215529 |
| YBR102C | 511.467276072776 | -1.36407281645107 | 0.17593080305159 | -7.75346211573345 | 8.94204757499748e-15 | 5.02148230055703e-14 | 754.723705777333 | 811.553889305606 | 644.083622523577 | 252.068992688126 | 308.227786595122 | 298.145659546891 |
| YBR103C-A | 4.59776222938409 | 2.05331903398672 | 0.996210595523075 | 2.06112948729339 | 0.0392906883379852 | 0.0565932499578442 | 1.52008802774891 | 0.821410819135229 | 2.94102110741359 | 6.66849187005625 | 9.98886345447155 | 5.64669809747899 |
| YBR103W | 650.19126481715 | -0.479082356980505 | 0.131940746443446 | -3.63104173573744 | 0.000282279527159345 | 0.000552797407353717 | 751.683529721835 | 761.447829338357 | 759.51870098956 | 514.807572368343 | 515.139958152033 | 598.549998332773 |
| YBR104W | 2472.38680507923 | 0.882170217178171 | 0.191820119035171 | 4.59894520770485 | 4.24635437017513e-06 | 1.03230338999085e-05 | 1855.26743786754 | 1968.10032264801 | 1394.7792601909 | 3478.28535942134 | 2809.7245916935 | 3328.16385865412 |
| YBR105C | 1213.11318146244 | -0.396785196420723 | 0.168511581954769 | -2.35464643924134 | 0.018540336857201 | 0.0282283156938511 | 1479.04565099969 | 1488.39640427304 | 1169.79114547376 | 937.589956929909 | 1074.51631160244 | 1129.3396194958 |
| YBR106W | 20439.3388289566 | 1.20481044378591 | 0.10885500478276 | 11.0680298640409 | 1.79299073126544e-28 | 2.95160859974305e-27 | 12567.3277694141 | 12904.3639686144 | 11634.6795009282 | 27700.9152282137 | 27433.6999874594 | 30395.0465191099 |
| YBR107C | 172.57219990694 | -0.753208276040704 | 0.180392711652707 | -4.17538086289641 | 2.9748791381635e-05 | 6.5607161136105e-05 | 214.332411912596 | 203.709883145537 | 231.60541220882 | 124.033948783046 | 134.136166388618 | 127.615377003025 |
| YBR108W | 286.52967536779 | -1.3043828226793 | 0.231337110620378 | -5.63845039467008 | 1.7158723313022e-08 | 5.31224245385492e-08 | 441.585572061058 | 420.562339397237 | 361.010340935019 | 130.702440653103 | 208.339152050407 | 156.978207109916 |
| YBR109C | 6777.48444794682 | 0.957667345869761 | 0.102258550610978 | 9.36515665582836 | 7.59375579191111e-21 | 7.07659483711717e-20 | 4592.18593182945 | 4783.07519982444 | 4446.0886591325 | 8526.33370505392 | 9466.58859385204 | 8850.63459798857 |
| YBR109W-A | 11.6383065321474 | -0.588442530267865 | 0.674099809119323 | -0.872930866182317 | 0.382700761069698 | 0.447646011956369 | 15.9609242913635 | 13.9639839252989 | 11.7640844296544 | 13.3369837401125 | 11.4158439479675 | 3.38801885848739 |
| YBR110W | 1630.45470960311 | -0.0613884635311111 | 0.15171109304193 | -0.404640572421059 | 0.68574173082085 | 0.736743559069742 | 1584.69176892824 | 1812.85367783145 | 1596.97446132558 | 1617.77612767565 | 1772.30977292195 | 1398.1224489358 |
| YBR111C | 2279.11785706455 | 0.117379250226159 | 0.114322482799148 | 1.02673811268062 | 0.30454379769096 | 0.367520223043073 | 2207.92786030529 | 2138.95377302814 | 2213.11838332873 | 2183.26423825642 | 2385.9113851252 | 2545.53150234353 |
| YBR111W-A | 211.49355200002 | -1.54609523420009 | 0.31633464781984 | -4.88753048347908 | 1.02108688998393e-06 | 2.65122881042264e-06 | 408.143635450582 | 303.1005922609 | 234.546433316234 | 126.701345531069 | 75.6299661552846 | 120.83933928605 |
| YBR112C | 356.857364163922 | -1.49094544370229 | 0.440340678723005 | -3.3858907789897 | 0.000709476151567023 | 0.00130877767891459 | 670.358820237269 | 702.306250360621 | 206.606732795805 | 185.384073987564 | 196.923308102439 | 179.564999499832 |
| YBR114W | 643.202661599584 | -0.693318079483272 | 0.172807786756917 | -4.01207661121508 | 6.01869597470042e-05 | 0.000127865254149712 | 769.924586054822 | 774.590402444521 | 838.926270889727 | 445.455256919758 | 586.48898282683 | 443.830470461849 |
| YBR115C | 5631.00239615629 | -0.262007586476765 | 0.117550158830008 | -2.22890031867725 | 0.0258205379200514 | 0.0383933995757572 | 6095.55299127312 | 6470.2530223282 | 5856.30828013732 | 5494.83730092635 | 5137.12977658537 | 4731.93300568739 |
| YBR116C | 25.3889516012264 | 1.815767469536 | 0.469028911416751 | 3.87133378207173 | 0.000108241455188276 | 0.000221818034234174 | 15.9609242913635 | 6.57128655308183 | 11.028829152801 | 42.67834796836 | 39.9554538178862 | 36.1388678238655 |
| YBR117C | 190.19630388104 | 1.50074544066161 | 0.194302897241496 | 7.7237419614817 | 1.1296340882183e-14 | 6.25688018461474e-14 | 105.646117928549 | 99.3907091153627 | 92.6421648835282 | 302.749530900554 | 296.811942647155 | 243.937357811092 |
| YBR118W | 82432.3070354394 | 0.7288074768808 | 0.0988949876880436 | 7.36950874780192 | 1.71257928650874e-13 | 8.49219361796306e-13 | 60610.4699304322 | 61411.1370710071 | 64106.172333571 | 107349.382124166 | 104597.670173252 | 96519.0105802084 |
| YBR119W | 155.772078774858 | -1.7833825100969 | 0.48092387374705 | -3.70824283727469 | 0.000208702465054116 | 0.00041599391159834 | 339.739674201881 | 276.815446048572 | 108.08252569745 | 81.3556008146863 | 42.8094148048781 | 85.8298110816806 |
| YBR120C | 186.493851615891 | -0.685486890497517 | 0.37151141564882 | -1.84513008651527 | 0.0650186175465789 | 0.0899170049908552 | 202.931751704479 | 180.71038020975 | 306.601450447867 | 186.717772361575 | 82.7648686227643 | 159.236886348907 |
| YBR121C | 21176.9031098001 | 0.639636848395866 | 0.113498309857427 | 5.63565086739491 | 1.74398386954575e-08 | 5.38641875136843e-08 | 15762.5528037423 | 16657.3900012433 | 17252.765071365 | 27636.8977062611 | 25864.0214446138 | 23887.7916315751 |
| YBR122C | 1929.30847948212 | 1.73380069760618 | 0.136299803371425 | 12.720493021413 | 4.54980356324253e-37 | 1.38544722587751e-35 | 815.527226887289 | 894.516382038265 | 966.125433785365 | 2918.13204233662 | 2764.06121590163 | 3217.48857594353 |
| YBR123C | 209.800631170623 | -2.46291491802857 | 0.20431256992327 | -12.054642154193 | 1.83326968368422e-33 | 4.24663827441994e-32 | 354.180510465496 | 327.742916834956 | 383.06799924062 | 68.0186170745738 | 67.068083194309 | 58.7256602137815 |
| YBR124W | 1.89262871764292 | 0.970724223389076 | 1.34079157155622 | 0.723993381210161 | 0.469069798535544 | 0.533564839232118 | 0.760044013874454 | 1.64282163827046 | 1.4705105537068 | 2.6673967480225 | 1.42698049349594 | 3.38801885848739 |
| YBR125C | 989.838290367193 | 0.484253651180993 | 0.146893933554631 | 3.29662117054612 | 0.000978553934416138 | 0.00177337267913469 | 742.563001555342 | 801.696959475984 | 931.568435773256 | 1193.66004474007 | 1091.64007752439 | 1177.90122313412 |
| YBR126C | 7015.13577739678 | 0.994490504742817 | 0.285251066828965 | 3.48636909862675 | 0.000489624941665538 | 0.000924514518673268 | 4007.71208516 | 3836.80993618065 | 6220.9948974566 | 7363.34872291611 | 12250.6275366626 | 8411.3214860047 |
| YBR126W-A | 7.66421723713529 | 1.56479238207235 | 0.780633722089913 | 2.00451548247632 | 0.0450148701093615 | 0.0640980126299273 | 1.52008802774891 | 5.7498757339466 | 4.41153166112039 | 9.33588861807875 | 11.4158439479675 | 13.5520754339496 |
| YBR126W-B | 322.078964555528 | 1.63582827180479 | 0.149196932152962 | 10.9642219059014 | 5.67869033702354e-28 | 8.96155365594517e-27 | 166.449639038505 | 151.961001540017 | 152.197842308653 | 496.135795132185 | 460.914699399187 | 504.814809914622 |
| YBR127C | 19369.5877821638 | 1.56926907136103 | 0.282318053809442 | 5.55851476795832 | 2.72079940885191e-08 | 8.22325487689353e-08 | 7212.81769166857 | 7969.32776724999 | 14109.5487628167 | 28158.3737704995 | 31403.5597203651 | 27363.8989803832 |
| YBR128C | 74.8842276017155 | -0.819460804380704 | 0.264965500404808 | -3.0927075529786 | 0.00198339484713562 | 0.00345721552768655 | 85.1249295539389 | 110.069049764121 | 91.9069096066748 | 50.6805382124275 | 52.7982782593496 | 58.7256602137815 |
| YBR129C | 721.253612733939 | 0.552335639630143 | 0.131690629781311 | 4.19419088926348 | 2.73847302151859e-05 | 6.0661666726672e-05 | 633.11666355742 | 584.844503224283 | 536.736352102981 | 888.243117091493 | 831.929627708131 | 852.651412719327 |
| YBR130C | 337.16751527532 | -1.96008143980459 | 0.414308594378102 | -4.73096978050086 | 2.23449786654544e-06 | 5.62178167665389e-06 | 749.403397680212 | 620.165168447098 | 239.693220254208 | 149.37421788926 | 141.271068856098 | 123.098018525042 |
| YBR131W | 143.970764963906 | -1.10113163363265 | 0.244301067522405 | -4.5072731150946 | 6.56660870082e-06 | 1.56988662121336e-05 | 171.769947135627 | 185.638845124562 | 232.340667485674 | 84.0229975627088 | 82.7648686227643 | 107.287263852101 |
| YBR132C | 401.199927719355 | 0.732311766200551 | 0.350841911432167 | 2.08729841657512 | 0.0368611678359372 | 0.0533545044820105 | 224.212984092964 | 278.458267686843 | 401.449381161956 | 356.097465861004 | 723.47911020244 | 423.502357310924 |
| YBR133C | 2016.44798147001 | 0.0958810945253306 | 0.199130592781381 | 0.481498564264284 | 0.630162199895598 | 0.686301434937609 | 1739.74074775863 | 1749.60504475804 | 2359.43418342256 | 2185.93163500444 | 1768.02883144146 | 2295.94744643496 |
| YBR134W | 2.42049235681363 | 0.457612288630034 | 1.90584667177473 | 0.24010970840792 | 0.810245208120791 | 0.844623982621576 | 0 | 1.64282163827046 | 4.41153166112039 | 1.33369837401125 | 7.13490246747968 | 0 |
| YBR135W | 709.192100790753 | -0.20183820374748 | 0.221607425375061 | -0.910791700259492 | 0.36240513641027 | 0.427141507315466 | 727.362121277853 | 639.879028106343 | 909.510777467654 | 662.848091883591 | 540.82560703496 | 774.726978974117 |
| YBR136W | 643.955218360962 | -0.67567264109871 | 0.136175889004471 | -4.96176412754337 | 6.98557950065847e-07 | 1.84405651775624e-06 | 777.525026193567 | 749.948077870464 | 849.219844765675 | 466.794430903938 | 489.454309269106 | 530.789621163025 |
| YBR137W | 1452.66452625864 | 1.45780786739215 | 0.15200919290812 | 9.59026121711799 | 8.78622828720971e-22 | 8.70037811768583e-21 | 720.521725152983 | 742.555380498247 | 862.454439749036 | 2037.89111548919 | 2377.34950216423 | 1975.21499449815 |
| YBR138C | 73.5591971926922 | -2.12542984377288 | 0.574157919602095 | -3.7018209994314 | 0.000214057618633043 | 0.000425752135680441 | 163.409462983008 | 155.246644816558 | 40.4390402269369 | 36.0098560983038 | 21.404707402439 | 24.8454716289076 |
| YBR139W | 2716.45830471351 | 1.08725114171367 | 0.206550953550026 | 5.26383985659181 | 1.41077347952677e-07 | 4.00800560149391e-07 | 1561.890448512 | 1522.07424785758 | 2131.505047598 | 3410.26674234677 | 4316.61599282521 | 3356.39734914151 |
| YBR140C | 2818.05284011013 | 0.228478159062698 | 0.11086837699291 | 2.06080548177692 | 0.0393216013042843 | 0.0566252011677593 | 2438.22119650925 | 2695.87030840182 | 2651.33052833335 | 3086.17803746203 | 3169.32367605447 | 2867.39329389983 |
| YBR141C | 253.842410253109 | -1.19233052001337 | 0.18080578607145 | -6.59453740900855 | 4.2658384494862e-11 | 1.70266019589954e-10 | 386.102359048223 | 363.884992876906 | 309.542471555281 | 150.707916263271 | 156.967854284553 | 155.84886749042 |
| YBR141W-A | 7.59341721125017 | 1.41938962544253 | 0.794956643855578 | 1.78549312898175 | 0.0741813648546838 | 0.101807095312628 | 6.08035211099563 | 3.28564327654092 | 2.94102110741359 | 13.3369837401125 | 14.2698049349594 | 5.64669809747899 |
| YBR142W | 1348.13253971676 | -4.08961319421449 | 0.27706854207703 | -14.760294198529 | 2.64126133795963e-49 | 1.63154486076249e-47 | 2945.17055376351 | 3008.00641967321 | 1687.41086037855 | 152.041614637283 | 131.282205401626 | 164.883584446386 |
| YBR143C | 13542.2286926515 | 0.442432062964157 | 0.124786197493944 | 3.54552083362928 | 0.000391838011750967 | 0.000750357645177672 | 11485.7851376708 | 12194.6650208816 | 10764.8725084106 | 17027.3271410016 | 15322.9165391594 | 14457.8058087852 |
| YBR144C | 9.28129862962582 | 2.65329040988761 | 0.767138173991278 | 3.45868645290199 | 0.000542816415627612 | 0.0010184284847442 | 3.04017605549782 | 1.64282163827046 | 2.94102110741359 | 20.0054756101688 | 9.98886345447155 | 18.0694339119328 |
| YBR145W | 1519.41008924114 | 0.826809132601049 | 0.240960263800513 | 3.43130904473756 | 0.00060067586066995 | 0.00111857124097195 | 1176.54813347766 | 1282.22228867009 | 827.897441736927 | 1628.44571466774 | 2361.65271673577 | 1839.69424015865 |
| YBR146W | 1629.69127029993 | 0.626092461144673 | 0.198033431932815 | 3.1615493153554 | 0.00156932242782317 | 0.00277497962564369 | 1133.98566870069 | 1111.36883828996 | 1599.18022715614 | 1995.21276752083 | 1826.5350316748 | 2111.86508845714 |
| YBR147W | 299.505753716896 | -0.0703785493812405 | 0.278918893654247 | -0.252326217342892 | 0.800788925089044 | 0.836648996154565 | 311.618045688526 | 368.813457791718 | 239.693220254208 | 234.73091382598 | 382.430772256911 | 259.748112484034 |
| YBR148W | 18.1450746788327 | -0.0731559149372466 | 0.487210571139112 | -0.15015256086543 | 0.880644252420951 | 0.904275826096057 | 18.2410563329869 | 16.4282163827046 | 21.3224030287486 | 20.0054756101688 | 11.4158439479675 | 21.4574527704202 |
| YBR149W | 3954.04530347036 | 1.291555827709 | 0.325023922254944 | 3.97372543764924 | 7.07571236155861e-05 | 0.000148569344050078 | 1651.57564214919 | 1687.17782250376 | 3541.72466860282 | 5088.05929685292 | 5963.35148231952 | 5792.38290839395 |
| YBR150C | 318.977122489391 | -0.968314199199726 | 0.224829842098306 | -4.30687576952677 | 1.65576538577606e-05 | 3.76817343583982e-05 | 367.101258701361 | 462.454291173134 | 436.006379174065 | 202.72215284971 | 268.272332777236 | 177.30632026084 |
| YBR151W | 3045.25589874451 | 0.555059984507644 | 0.162900477455372 | 3.40735640053424 | 0.000655954141899762 | 0.00121418908800281 | 2259.61085324875 | 2331.98531552492 | 2807.20464702627 | 3538.30178625185 | 4074.0293089309 | 3260.40348148437 |
| YBR152W | 12.0176742299415 | -6.66038069404377 | 1.45788351779354 | -4.56852732934662 | 4.91163034151149e-06 | 1.19007706721398e-05 | 31.1618045688526 | 32.8564327654092 | 8.08780804538738 | 0 | 0 | 0 |
| YBR153W | 840.764381044262 | 1.31001224941801 | 0.128706108777227 | 10.178322240209 | 2.47796770956551e-24 | 3.09674346131828e-23 | 451.466144241426 | 466.56134526881 | 531.589565165007 | 1206.99702848018 | 1182.96682910813 | 1205.00537400202 |
| YBR154C | 1987.26156511004 | -2.21216429151269 | 0.242140896309823 | -9.13585571551776 | 6.48915219530724e-20 | 5.71856537211451e-19 | 3749.29712044268 | 3390.78386139023 | 2668.24139970098 | 761.541771560424 | 517.993919139025 | 835.71131842689 |
| YBR155W | 90.9616629395413 | -3.12736029643344 | 0.569638117558124 | -5.49008256301305 | 4.01745902764514e-08 | 1.19968873173602e-07 | 236.373688314955 | 194.674364135049 | 58.8204221482719 | 13.3369837401125 | 19.9777269089431 | 22.586792389916 |
| YBR156C | 66.5411069916469 | -1.0962138680485 | 0.339603787357358 | -3.22792003168969 | 0.00124693827378271 | 0.00223107355689785 | 100.325809831428 | 105.961995668445 | 65.4377196399524 | 40.0109512203375 | 51.3712977658537 | 36.1388678238655 |
| YBR157C | 387.352871537156 | 0.255553293745025 | 0.310838675799988 | 0.822141238014613 | 0.41099651773144 | 0.475767162949513 | 274.375889008678 | 280.101089325113 | 505.120375198285 | 390.773623585296 | 361.026064854472 | 512.720187251092 |
| YBR158W | 4621.85731468476 | -0.762288793475974 | 0.193217300455864 | -3.94524088514579 | 7.9719796809637e-05 | 0.00016599120452883 | 6604.02243655513 | 6369.21949157457 | 4472.55784909922 | 3615.6562919445 | 3377.66282810488 | 3292.02499083025 |
| YBR159W | 6751.21237588965 | 0.891055936250631 | 0.128724919185512 | 6.92217126170017 | 4.44773061167731e-12 | 1.94542065753782e-11 | 4997.28939122454 | 5031.96267802241 | 4161.54486699023 | 9011.79991319402 | 8694.59214687074 | 8610.08525903596 |
| YBR160W | 1630.36873448107 | -0.186024427315158 | 0.137449391634593 | -1.35340306059485 | 0.175926900226731 | 0.225061513781179 | 1785.34338859109 | 1896.63758138324 | 1524.18418891709 | 1495.07587726661 | 1565.39760136504 | 1515.57376936336 |
| YBR161W | 446.801753868493 | 0.0163604923746299 | 0.176884749786942 | 0.092492384981386 | 0.926306841192057 | 0.941007369918271 | 395.222887214716 | 412.348231205885 | 524.972267673326 | 465.460732529926 | 442.36395298374 | 440.442451603361 |
| YBR162C | 33456.2256740788 | 0.465649195601033 | 0.123865369379523 | 3.75931705474744 | 0.000170377811106206 | 0.000342445144975163 | 28924.2349920062 | 30055.421872158 | 25330.2795428764 | 38993.3393609669 | 40726.023284374 | 36708.0549920914 |
| YBR162W-A | 1414.07199530565 | -0.0297725156462491 | 0.273042390223283 | -0.109039902638936 | 0.913170841276313 | 0.930384240734868 | 1905.43034278326 | 1206.65249330965 | 1174.20267713488 | 1628.44571466774 | 1068.80838962846 | 1500.89235430992 |
| YBR163W | 711.909639571023 | 0.0898933225354051 | 0.176967756532777 | 0.507964412820906 | 0.611478300631749 | 0.669488227194045 | 598.154638919195 | 675.199693329158 | 796.28146483223 | 754.873279690368 | 656.41102700813 | 790.537733647059 |
| YBR164C | 1724.33475149224 | 0.0975643584899356 | 0.229245177355789 | 0.425589578874829 | 0.670406935194811 | 0.723022843643756 | 1881.10893433927 | 1871.99525680919 | 1244.7871837128 | 2015.218243131 | 1836.52389512927 | 1496.37499583193 |
| YBR165W | 533.08471514541 | 0.471515742378208 | 0.158095926190512 | 2.9824661124412 | 0.00285936301127274 | 0.00488562394391859 | 465.90698050504 | 461.632880353999 | 413.21346559161 | 678.852472371726 | 540.82560703496 | 638.076885015126 |
| YBR166C | 1745.44039951628 | 0.102267645485634 | 0.128353826010897 | 0.796763514294864 | 0.425588388700157 | 0.490993648009466 | 1756.46171606386 | 1759.46197458766 | 1534.47776279304 | 1905.85497646208 | 1833.66993414228 | 1682.71603304874 |
| YBR167C | 320.43854422898 | -1.81421794178265 | 0.27447260466998 | -6.60983249663122 | 3.84755120010764e-11 | 1.54044549900606e-10 | 594.354418849823 | 563.487821926767 | 338.952682629417 | 150.707916263271 | 144.12502984309 | 131.003395861513 |
| YBR168W | 368.494334557849 | -0.0251032755914032 | 0.2261155694855 | -0.111019668608061 | 0.911600749852382 | 0.929269246753664 | 311.618045688526 | 336.778435845444 | 466.151845525055 | 396.108417081341 | 379.576811269919 | 320.732451936807 |
| YBR169C | 1113.8080645139 | -1.01936130384427 | 0.270669053907768 | -3.76607997525873 | 0.000165830692454643 | 0.000333823051291375 | 1365.03904891852 | 1228.00917460717 | 1881.51825346785 | 557.485920336703 | 946.088067187806 | 704.707922565378 |
| YBR170C | 572.204959802846 | 0.215658131437109 | 0.25061942329034 | 0.860500469619514 | 0.389513224757335 | 0.454712522637882 | 414.984031575452 | 459.990058715728 | 713.197618547796 | 608.16645854913 | 656.41102700813 | 580.48056442084 |
| YBR171W | 1486.16670906002 | 0.503995056826784 | 0.151412431573655 | 3.32862402108387 | 0.00087276137133779 | 0.00159009276811711 | 1149.94659299205 | 1113.83307074737 | 1423.45421598818 | 1677.79255450615 | 1782.29863637642 | 1769.67518374992 |
| YBR172C | 465.21075798117 | -0.850803706863118 | 0.416903982830856 | -2.04076655993066 | 0.0412740359019007 | 0.0591479003225205 | 799.566302595926 | 730.234218211219 | 266.16241022093 | 364.099656105071 | 254.002527842277 | 377.199432911596 |
| YBR173C | 2183.80895701387 | 0.404565053257572 | 0.195571361157934 | 2.06863137251914 | 0.0385806916274326 | 0.0556447333545759 | 1744.30101184187 | 1664.17831956797 | 2230.76450997321 | 2519.35622850725 | 2106.2232084 | 2838.03046379294 |
| YBR174C | 13.5045747049285 | 0.825087568172216 | 0.526047194881991 | 1.56846681476423 | 0.116772228738419 | 0.154473725392084 | 9.8805721803679 | 8.21410819135229 | 11.028829152801 | 18.6717772361575 | 18.5507464154472 | 14.6814150534454 |
| YBR175W | 1381.7299186262 | 1.50143295162863 | 0.124720189693412 | 12.0384113856743 | 2.23212680029691e-33 | 5.13389164068288e-32 | 721.281769166857 | 750.769488689599 | 692.610470795901 | 2055.22919435134 | 1875.05236845366 | 2195.43622029983 |
| YBR176W | 509.230737430269 | 0.804765343291697 | 0.194107760747151 | 4.14597201159826 | 3.38375161120949e-05 | 7.41202733883983e-05 | 365.581170673612 | 337.599846664579 | 409.537189207343 | 686.854662615794 | 525.128821606504 | 730.682733813781 |
| YBR177C | 6750.12301804048 | 1.34290237316385 | 0.293984344441146 | 4.56793839044955 | 4.92544749629636e-06 | 1.19292204858022e-05 | 2829.64386365459 | 3016.22052786456 | 5605.58623073031 | 9518.60529531829 | 10649.5554229602 | 8881.12676771496 |
| YBR178W | 17.6657826813881 | 1.50863496075901 | 0.48816339511148 | 3.09043032694922 | 0.00199866690031603 | 0.0034828999235491 | 9.12052816649345 | 8.21410819135229 | 10.2935738759476 | 25.3402691062138 | 21.404707402439 | 31.6215093458823 |
| YBR179C | 415.518768273927 | -0.862958832457123 | 0.213502734631917 | -4.04190997340096 | 5.30175885180575e-05 | 0.000113639153710549 | 516.829929434629 | 446.026074790429 | 646.289388354137 | 281.410356916374 | 268.272332777236 | 334.284527370756 |
| YBR180W | 28.5926926278201 | -0.337051454238524 | 0.39746965815851 | -0.847992915484655 | 0.396441916214053 | 0.462135562286907 | 29.6417165411037 | 30.3922003080035 | 36.0275085658165 | 21.33917398418 | 21.404707402439 | 32.7508489653781 |
| YBR181C | 23884.443885678 | 0.533595824588315 | 0.228742900754448 | 2.332731738683 | 0.0196622293057751 | 0.0297826294435445 | 22330.8531716453 | 22406.4443243708 | 13814.7113967985 | 27870.2949217131 | 26225.0475094683 | 30659.3119900719 |
| YBR182C | 1119.43395963759 | -1.05038188095712 | 0.205890031092278 | -5.10166458951259 | 3.36679041988072e-07 | 9.1598165534171e-07 | 1530.72864394315 | 1609.14379468591 | 1388.89721797607 | 597.49687155704 | 916.121476824391 | 674.215752838991 |
| YBR182C-A | 15.2541709018068 | 1.55169713285708 | 0.585693597822427 | 2.6493325838394 | 0.00806509200195336 | 0.0129352588339934 | 6.08035211099563 | 9.03551901048752 | 8.08780804538738 | 16.004380488135 | 34.2475318439025 | 18.0694339119328 |
| YBR183W | 2329.92214031498 | 0.787425157179798 | 0.205001140186245 | 3.84107696408135 | 0.00012249569699809 | 0.000249844997084784 | 1903.15021074163 | 1786.56853161912 | 1438.15932152525 | 2603.37922606996 | 3598.84480459675 | 2649.43074733714 |
| YBR184W | 74.8965235965968 | -1.29232304905453 | 0.305940012076826 | -4.22410602745875 | 2.39891099720461e-05 | 5.34771034018891e-05 | 126.167306303159 | 110.890460583256 | 82.3485910075806 | 44.0120463423713 | 38.5284733243903 | 47.4322640188235 |
| YBR185C | 1273.31914298273 | 1.60931982877183 | 0.169744521495108 | 9.48083516685518 | 2.52255923325936e-21 | 2.43110240518874e-20 | 594.354418849823 | 560.202178650226 | 730.843745192278 | 2128.58260492196 | 1833.66993414228 | 1792.26197613983 |
| YBR186W | 20.7269296953307 | -1.04215897593576 | 0.596479655559565 | -1.7471827684686 | 0.0806056397592647 | 0.11006487989023 | 24.3214084439825 | 31.2136111271387 | 27.9397005204291 | 5.334793496045 | 24.2586683894309 | 11.293396194958 |
| YBR187W | 6177.67525597007 | 0.613112649952456 | 0.205969427189996 | 2.97671677936401 | 0.00291353023762758 | 0.0049742450964076 | 5529.32020093665 | 5512.48800721652 | 3611.57391990389 | 7492.7174651952 | 7630.06469872277 | 7289.88724384538 |
| YBR188C | 109.876618823264 | -2.67008195492713 | 0.302644108720951 | -8.82251422706213 | 1.11917972450503e-18 | 9.0398501782561e-18 | 194.57126755186 | 208.638348060348 | 166.902947845721 | 38.6772528463263 | 19.9777269089431 | 30.4921697263865 |
| YBR189W | 87884.955235102 | 0.252263439766594 | 0.22243874937417 | 1.13408046249287 | 0.256760798150862 | 0.315347573718328 | 91330.6889272238 | 92556.5711001576 | 56775.6772233426 | 94169.7747921864 | 96312.6214280147 | 96164.3979396867 |
| YBR190W | 5.54308724482369 | -1.83633961701811 | 1.25044114827448 | -1.46855341377091 | 0.141953958512836 | 0.184474729495943 | 6.08035211099563 | 16.4282163827046 | 3.67627638426699 | 0 | 1.42698049349594 | 5.64669809747899 |
| YBR191W | 72483.7302722457 | 0.898979860332028 | 0.164055149644323 | 5.47974179585977 | 4.25946979601464e-08 | 1.26903633885857e-07 | 55566.0578103475 | 55122.4158397078 | 41123.5628896874 | 95371.4370271705 | 95661.9183229805 | 92056.9897435805 |
| YBR191W-A | 6167.91749915402 | 1.02277686443131 | 0.177207802748146 | 5.77162432223662 | 7.85110012284536e-09 | 2.51467829119877e-08 | 4608.90690013469 | 4318.97808701303 | 3278.5032794893 | 8813.07885546634 | 8289.32968671789 | 7698.70818610285 |
| YBR192W | 664.709033894509 | -0.0866377257895281 | 0.123549336347555 | -0.701239912335979 | 0.483153305576982 | 0.546613001913886 | 680.239392417637 | 669.449817595212 | 704.374555225556 | 597.49687155704 | 669.253851449594 | 667.439715122017 |
| YBR193C | 426.329073110206 | -0.390361378087571 | 0.163530709398529 | -2.38708301042252 | 0.0169826561932184 | 0.0260093289419632 | 459.06658438017 | 450.133128886106 | 541.883139040955 | 397.442115455353 | 336.767396465041 | 372.682074433613 |
| YBR194W | 124.394210607514 | -1.18741669747702 | 0.228257353428651 | -5.20209614122325 | 1.97053234938669e-07 | 5.50899690436295e-07 | 182.410563329869 | 183.996023486291 | 152.197842308653 | 86.6903943107313 | 69.9220441813009 | 71.1483960282353 |
| YBR195C | 546.291626046899 | 0.118707220407205 | 0.203624594744636 | 0.58297093509787 | 0.559912866430913 | 0.621635544620147 | 463.626848463417 | 471.489810183622 | 636.731069755043 | 552.151126840658 | 512.285997165041 | 641.464903873613 |
| YBR196C | 75332.7135854349 | 0.967214976538735 | 0.133099228402747 | 7.2668714022295 | 3.67908738913299e-13 | 1.78078811984452e-12 | 47319.5802598096 | 49685.4976278517 | 55950.7208027131 | 96901.1890621614 | 108861.487887818 | 93277.8058722554 |
| YBR196C-A | 76.2796547339365 | 0.859601592743513 | 0.328981531061791 | 2.61291747889051 | 0.00897729753171956 | 0.014253794808013 | 41.0423767492205 | 63.2486330734126 | 58.0851668714185 | 86.6903943107313 | 128.428244414634 | 80.1831129842017 |
| YBR196C-B | 37.8000770475734 | 0.707964453853875 | 0.374654533343124 | 1.88964603614043 | 0.0588053158170345 | 0.0819006395510598 | 33.441936610476 | 31.2136111271387 | 21.3224030287486 | 45.3457447163825 | 57.0792197398374 | 38.3975470628571 |
| YBR197C | 241.835901526632 | -1.02633096756648 | 0.223358752664683 | -4.59498880309048 | 4.32772506539029e-06 | 1.05169069966734e-05 | 274.375889008678 | 313.778932909657 | 384.538509794327 | 144.039424393215 | 184.080483660976 | 150.202169392941 |
| YBR198C | 1209.04168940969 | -0.236674065715444 | 0.204750769728785 | -1.15591294737961 | 0.24771676617582 | 0.305540607359735 | 1530.72864394315 | 1398.8626249873 | 994.800389582648 | 1110.97074555137 | 1054.5385846935 | 1164.34914770017 |
| YBR199W | 3479.07751643488 | 0.607600136805638 | 0.103589168710947 | 5.86547941610646 | 4.4783682424571e-09 | 1.47295620794e-08 | 2632.03242004724 | 2847.83130994184 | 2791.0290309355 | 4393.20244399306 | 4218.15433877399 | 3992.21555491765 |
| YBR200W | 456.63461094088 | -2.11544635324456 | 0.157576915576232 | -13.4248493537821 | 4.32462885789693e-41 | 1.73145325755059e-39 | 755.483749791208 | 756.519364423546 | 714.668129101503 | 189.385169109598 | 149.832951817073 | 173.918301402353 |
| YBR201C-A | 593.575012614814 | 1.21057657092539 | 0.245115615821067 | 4.9387982355604 | 7.86054902897957e-07 | 2.06327482808424e-06 | 381.542094964976 | 401.669890557127 | 291.161089633946 | 712.194931722008 | 1064.52744814797 | 710.354620662857 |
| YBR201W | 822.166801040367 | 0.794653631392723 | 0.150301458847188 | 5.28706532516529 | 1.24294261929947e-07 | 3.54829481900369e-07 | 630.836531515797 | 606.201184521799 | 566.146563177117 | 1037.61733498075 | 1167.27004367968 | 924.929148367058 |
| YBR202W | 1107.78111632181 | -0.517156670084752 | 0.16556285203785 | -3.12362745458458 | 0.0017863647863097 | 0.00313738478310444 | 1313.35605597506 | 1388.18428433854 | 1212.43595153125 | 946.925845547988 | 764.861544513822 | 1020.9230160242 |
| YBR203W | 646.296990346943 | 0.0369078730677569 | 0.163527594211234 | 0.225698135203296 | 0.821436208559903 | 0.854504450476268 | 595.874506877572 | 724.484342477272 | 593.351008420692 | 641.508917899411 | 720.625149215448 | 601.93801719126 |
| YBR204C | 465.739762674693 | 0.664116779948148 | 0.238244336968357 | 2.78754487262526 | 0.00531090985390661 | 0.00872506618856086 | 306.297737591405 | 303.1005922609 | 471.298632463028 | 546.816333344613 | 635.006319605691 | 531.918960782521 |
| YBR205W | 2146.85214522249 | 0.688913226740989 | 0.117369792811437 | 5.86959566204381 | 4.36859231500074e-09 | 1.43903960157922e-08 | 1519.32798373503 | 1678.14230349327 | 1734.46719809717 | 2619.3836065581 | 2541.45225891626 | 2788.33952053512 |
| YBR206W | 319.875433150245 | 1.41988673782851 | 0.269085773561268 | 5.2767068248787 | 1.31526052541654e-07 | 3.7481457679489e-07 | 134.527790455778 | 146.211125806071 | 241.163730807915 | 452.123748789814 | 470.903562853659 | 474.322640188235 |
| YBR207W | 2131.24675088743 | 0.589454609977587 | 0.126821976578826 | 4.64789010452943 | 3.35347481549835e-06 | 8.25137998987948e-06 | 1621.17388159421 | 1844.88869977772 | 1639.61926738308 | 2365.98091549596 | 2712.68991813577 | 2603.12782293781 |
| YBR208C | 4051.46012791752 | 1.19345547328045 | 0.389180163085897 | 3.06658865605399 | 0.00216516589525481 | 0.00375997973053332 | 1597.6125171641 | 1612.42943796245 | 4185.07303584954 | 5729.56821475233 | 6048.97031192927 | 5135.10724984739 |
| YBR209W | 1.44806259297251 | 0.351128219460587 | 1.6480087935604 | 0.213062103086234 | 0.831278523297699 | 0.86266760033742 | 0.760044013874454 | 1.64282163827046 | 1.4705105537068 | 0 | 1.42698049349594 | 3.38801885848739 |
| YBR210W | 1358.64207978078 | 1.24685840498705 | 0.145013244008996 | 8.59823813685389 | 8.09491939847506e-18 | 6.07681102066079e-17 | 849.72920751164 | 859.19571681545 | 708.050831609823 | 2057.89659109936 | 1802.27636328537 | 1874.70376836302 |
| YBR211C | 395.609313127611 | -0.340289517399396 | 0.191049319420482 | -1.78116058424919 | 0.0748862207374006 | 0.102730970326307 | 467.427068532789 | 490.382259023732 | 369.098148980406 | 358.764862609026 | 309.654767088618 | 378.328772531092 |
| YBR212W | 939.346405462034 | -3.2828479917284 | 0.17580026331974 | -18.6737376255101 | 8.09820041172749e-78 | 2.38749672138475e-75 | 1606.7330453306 | 1563.96619963348 | 1941.07393089297 | 161.377503255361 | 169.810678726016 | 193.117074933781 |
| YBR213W | 325.014567284858 | 0.742013983829026 | 0.387343151383114 | 1.91565019590372 | 0.0554096357509635 | 0.0775376262094389 | 152.008802774891 | 179.06755857148 | 398.508360054542 | 364.099656105071 | 402.408499165854 | 453.994527037311 |
| YBR214W | 1625.92334166551 | 0.400718171760447 | 0.462067303132995 | 0.86722901413587 | 0.385816541965221 | 0.45088398039395 | 805.646654706921 | 818.125175858688 | 2580.74602175543 | 1468.40190978639 | 2084.81850099756 | 1997.80178688807 |
| YBR215W | 79.1633315406846 | -0.246555172048044 | 0.299065269754467 | -0.824419272256075 | 0.409701368034298 | 0.474706041436054 | 91.2052816649345 | 102.676352391904 | 63.9672090862457 | 62.6838235785288 | 79.9109076357724 | 74.5364148867227 |
| YBR216C | 1077.97481564603 | 0.341592584199321 | 0.161295389459951 | 2.11780749185107 | 0.0341913745956921 | 0.0497343026749627 | 899.892112427354 | 888.766506304318 | 1063.17913033001 | 1213.66552035024 | 1338.50770289919 | 1063.83792156504 |
| YBR217W | 94.5824290990412 | -1.26437345448345 | 0.241329561172491 | -5.23919841539735 | 1.61275569105887e-07 | 4.55192924813222e-07 | 129.967526372532 | 133.889963519042 | 137.492736771585 | 54.6816333344613 | 47.0903562853659 | 64.3723583112605 |
| YBR218C | 8656.43440347302 | 0.253973618656289 | 0.169851388557899 | 1.49526960487411 | 0.134844094302939 | 0.175869454182358 | 6906.51995407717 | 7381.19762074917 | 9400.97396984755 | 9770.67428800642 | 9804.78297081058 | 8674.45761734722 |
| YBR220C | 1350.84502882178 | 0.221795666708524 | 0.112672557840329 | 1.96849766225096 | 0.049010804828968 | 0.0693010857032235 | 1216.830466213 | 1313.43589979723 | 1210.96544097755 | 1511.08025775475 | 1478.35179126179 | 1374.40631692639 |
| YBR221C | 15902.2694633564 | 0.924493743420582 | 0.108630313662731 | 8.51045819761598 | 1.73247358624944e-17 | 1.26256445847347e-16 | 10495.4477875923 | 11019.2261386991 | 11408.2208756573 | 21223.142225641 | 21935.5441460195 | 19332.0356065291 |
| YBR221W-A | 2.32943500003991 | 1.83363333620969 | 1.82066884425982 | 1.00712073037925 | 0.313876760258902 | 0.377341266100088 | 0 | 0.821410819135229 | 2.20576583056019 | 6.66849187005625 | 4.28094148048781 | 0 |
| YBR222C | 3361.31943245253 | 0.645478716249911 | 0.112476934633682 | 5.73876518196487 | 9.53693424902026e-09 | 3.02329205958678e-08 | 2502.82493768858 | 2617.01486976484 | 2744.70794849374 | 3865.0578878846 | 4385.11105651301 | 4053.19989437042 |
| YBR223C | 336.671194804476 | 0.219437477194764 | 0.32486014881296 | 0.675482905479754 | 0.499369008505068 | 0.563063069846043 | 228.773248176211 | 246.423245740569 | 458.064037479667 | 297.414737404509 | 389.56567472439 | 399.786225301512 |
| YBR224W | 1.35411526834516 | -0.932309901544758 | 2.43929044304663 | -0.382205368041502 | 0.702309040439757 | 0.750812005322609 | 3.80022006937227 | 0 | 1.4705105537068 | 0 | 2.85396098699187 | 0 |
| YBR225W | 378.337077488261 | -0.997225968515981 | 0.217482513496214 | -4.58531563059823 | 4.53301126360781e-06 | 1.10075294106178e-05 | 446.145836144305 | 446.847485609565 | 619.084943110561 | 222.727628459879 | 281.115157218699 | 254.101414386555 |
| YBR226C | 3.26185127116573 | -0.294838850754623 | 1.18481012159537 | -0.248849031064672 | 0.803477562488981 | 0.838852945505784 | 3.80022006937227 | 4.10705409567614 | 2.94102110741359 | 5.334793496045 | 0 | 3.38801885848739 |
| YBR227C | 329.817588982775 | -1.66107662709211 | 0.256311204308467 | -6.48070236170022 | 9.12966146127672e-11 | 3.53522293957259e-10 | 484.908080851902 | 453.418772162646 | 566.146563177117 | 177.381883743496 | 108.450517505691 | 188.599716455798 |
| YBR228W | 196.779873957377 | -0.483800276327376 | 0.232508260123157 | -2.08078747856576 | 0.0374533627700434 | 0.0541391822880547 | 251.574568592444 | 248.066067378839 | 188.22535087447 | 180.049280491519 | 178.372561686992 | 134.39141472 |
| YBR229C | 1353.98716007285 | -0.301758723953234 | 0.124088806108411 | -2.4317964965317 | 0.0150241460050988 | 0.0231519626963818 | 1441.04345030597 | 1414.46943055086 | 1630.06094878398 | 1219.00031384628 | 1160.1351412122 | 1259.21367573781 |
| YBR230C | 2221.73788887598 | 1.59172040325081 | 0.153415297868486 | 10.3752391408535 | 3.21406651989018e-25 | 4.25437458122606e-24 | 1096.74351202084 | 989.800037057951 | 1233.75835456 | 3052.83557811175 | 3684.46363420651 | 3272.82621729882 |
| YBR230W-A | 937.645490041578 | 2.06494450193 | 0.186849525113227 | 11.0513767732548 | 2.15878452459985e-28 | 3.5269210142455e-27 | 412.703899533829 | 313.778932909657 | 358.804575104458 | 1460.39971954232 | 1334.2267614187 | 1745.9590517405 |
| YBR231C | 181.186834270497 | 0.289462375994531 | 0.196336553181674 | 1.47431729499033 | 0.140396180695392 | 0.1825600697655 | 163.409462983008 | 149.496769082612 | 176.461266444816 | 172.047090247451 | 205.485191063415 | 220.221225801681 |
| YBR233W | 120.718874969269 | -3.87689518053604 | 0.331118044285896 | -11.7084986681929 | 1.1530319796527e-31 | 2.34437787461674e-30 | 211.292235857098 | 218.495277889971 | 248.516283576449 | 21.33917398418 | 9.98886345447155 | 14.6814150534454 |
| YBR233W-A | 292.006023113728 | 0.343431809550262 | 0.207300221614528 | 1.65668809649837 | 0.0975825508446189 | 0.131039425419917 | 276.656021050301 | 291.600840793006 | 204.400966965245 | 345.427878868914 | 299.665903634147 | 334.284527370756 |
| YBR234C | 6010.28518720535 | 0.70460598784562 | 0.116168864261298 | 6.06536004570683 | 1.31658374614169e-09 | 4.57874647585789e-09 | 4514.66144241426 | 4701.75552873005 | 4496.08601795853 | 7223.31039364493 | 8168.03634477074 | 6957.86139571361 |
| YBR235W | 1042.30595833105 | -0.12547028856957 | 0.180207286773216 | -0.696255355797402 | 0.486268926388458 | 0.549754271667341 | 943.974665232072 | 1015.26377245114 | 1303.60760586108 | 925.586671563808 | 1011.72916988862 | 1053.67386498958 |
| YBR236C | 1031.07545973347 | -0.656905143683884 | 0.155258356010033 | -4.23104533994572 | 2.32607774669286e-05 | 5.19343898969016e-05 | 1333.87724434967 | 1380.79158696632 | 1071.2669383754 | 794.884230910705 | 789.120212903253 | 816.512544895462 |
| YBR237W | 298.410564949742 | -2.61118103638107 | 0.241462012978924 | -10.8140448436044 | 2.95353240167844e-27 | 4.46541052617397e-26 | 601.954858988568 | 539.666908171846 | 397.037849500835 | 88.0240926847425 | 81.3378881292684 | 82.4417922231932 |
| YBR238C | 1240.057725045 | -0.441219088169514 | 0.179201430465543 | -2.46214043617443 | 0.0138110576611959 | 0.0213740205178994 | 1258.6328869761 | 1302.75755914847 | 1723.43836894437 | 1062.95760408697 | 990.32446248618 | 1102.2354686279 |
| YBR239C | 381.037996579812 | -0.87302414330961 | 0.142179262695537 | -6.14030574331438 | 8.23627997049605e-10 | 2.91755936038434e-09 | 460.586672407919 | 504.346242949031 | 513.943438520525 | 260.071182932194 | 273.98025475122 | 273.300187917983 |
| YBR240C | 329.409426995399 | 1.40383024302559 | 0.18991742654744 | 7.39179267825076 | 1.44862278346048e-13 | 7.23307726984193e-13 | 186.210783399241 | 176.603326114074 | 179.402287552229 | 389.439925211285 | 515.139958152033 | 529.660281543529 |
| YBR241C | 1050.88840333895 | 1.34994879891706 | 0.281086471627285 | 4.80261035368171 | 1.56610456643263e-06 | 3.98969136601809e-06 | 474.267464657659 | 482.168150832379 | 819.809633691539 | 1265.67975693668 | 1806.55730476585 | 1456.84810914958 |
| YBR242W | 1053.81932883973 | -2.34306390092186 | 0.282799765959535 | -8.28523988685735 | 1.17871257456084e-16 | 8.0053714749755e-16 | 1964.71377586546 | 1968.92173346714 | 1349.19343302599 | 405.44430569942 | 232.597820439838 | 402.044904540504 |
| YBR243C | 2237.74650113267 | 0.643873482587929 | 0.101743950758277 | 6.32837114923563 | 2.47762621780977e-10 | 9.272869964636e-10 | 1755.70167204999 | 1799.71110472529 | 1683.73458399428 | 2794.09809355357 | 2796.88176725203 | 2596.35178522084 |
| YBR244W | 280.963655357847 | 0.382427916281281 | 0.374755340412772 | 1.02047355978986 | 0.30750392267292 | 0.370581650400698 | 183.930651357618 | 159.353698912234 | 388.214786178594 | 362.76595773106 | 318.216650049594 | 273.300187917983 |
| YBR245C | 626.140872478728 | -3.23801358497837 | 0.260207300499466 | -12.4439767015108 | 1.50804701428382e-35 | 4.10172786339916e-34 | 1285.99447147558 | 1316.72154307377 | 794.07569900167 | 130.702440653103 | 114.158439479675 | 115.192641188571 |
| YBR246W | 2489.53341672313 | 1.16465204883405 | 0.199307832152584 | 5.84348360149957 | 5.11203328396523e-09 | 1.67373285612309e-08 | 1292.07482358657 | 1379.97017614718 | 1935.19188867814 | 3514.29521551964 | 3563.17029225935 | 3252.4981041479 |
| YBR247C | 2248.29807795452 | -0.666526749072561 | 0.136004159698616 | -4.90078208305966 | 9.54558914294147e-07 | 2.48745243797181e-06 | 2860.80566822345 | 2959.54318134423 | 2456.4878799672 | 1744.47747320672 | 1631.03870406585 | 1837.43556091966 |
| YBR248C | 2254.38135302459 | 0.128068659943786 | 0.124008297167278 | 1.03274267020239 | 0.301724339123938 | 0.364564840454147 | 2106.08196244611 | 2263.80821753669 | 2094.00702847848 | 2524.6910220033 | 2133.33583777642 | 2404.36404990655 |
| YBR249C | 26077.460934029 | 0.551242085433298 | 0.0860829220000206 | 6.40361726374909 | 1.51738212344383e-10 | 5.74868017094432e-10 | 20676.2373534407 | 21766.5652962644 | 21022.4188757924 | 31548.6350372361 | 31469.2008230659 | 29981.7082183744 |
| YBR250W | 65.1320725304968 | 0.0352129599181256 | 0.312689061507053 | 0.112613341024503 | 0.910337107727813 | 0.928518081572983 | 66.883873220952 | 51.7488816055194 | 74.2607829621932 | 82.6892991886975 | 54.2252587528456 | 60.9843394527731 |
| YBR251W | 378.565066133208 | -0.37217957755486 | 0.186614376876157 | -1.9943778383262 | 0.0461107785540708 | 0.0655433946310987 | 419.544295658699 | 432.883501684266 | 430.124336959238 | 328.089800006768 | 271.126293764228 | 389.62216872605 |
| YBR252W | 4416.48191204984 | 1.78734243996165 | 0.108109450638562 | 16.5327122597006 | 2.13324716667819e-61 | 2.71298845550486e-59 | 2072.64002583564 | 2023.13484753007 | 1857.25482933168 | 6815.19869119749 | 6568.39121156179 | 7162.27186684235 |
| YBR253W | 703.186623970227 | 0.684332293635619 | 0.252117109387149 | 2.71434293094629 | 0.00664073830113578 | 0.0107814339477263 | 567.752878364217 | 565.952054384173 | 485.268482723243 | 1018.9455577446 | 590.769924307318 | 990.430846297815 |
| YBR254C | 763.974927243962 | 1.00895400491225 | 0.127699356631519 | 7.90101087058431 | 2.76650522242434e-15 | 1.6253218181743e-14 | 512.269665351382 | 497.774956395949 | 511.002417413112 | 1096.30006343725 | 1030.27991630407 | 936.222544562016 |
| YBR255C-A | 605.342674129386 | 1.63921340238909 | 0.194526826602695 | 8.42667014630865 | 3.5564054648422e-17 | 2.52372492833332e-16 | 338.219586174132 | 312.957522090522 | 231.60541220882 | 881.574625221436 | 978.908618538212 | 888.790280543193 |
| YBR255W | 168.79720782017 | -1.60504826126234 | 0.494823318313782 | -3.24367951520937 | 0.00117996429605086 | 0.00211825309277217 | 373.181610812357 | 294.886484069547 | 94.112675437235 | 102.694774798866 | 75.6299661552846 | 72.2777356477311 |
| YBR256C | 3447.74720388061 | 0.619360579265689 | 0.124023196905884 | 4.99390916149093 | 5.91692361005748e-07 | 1.57283469405052e-06 | 2845.60478794596 | 2778.01139031534 | 2533.68968403681 | 4349.19039765069 | 3807.18395664716 | 4372.80300668773 |
| YBR257W | 230.502695587727 | -1.7695050196064 | 0.202544309798138 | -8.73638475141535 | 2.40689220061583e-18 | 1.8763344727397e-17 | 389.902579117595 | 369.634868610853 | 310.277726832134 | 112.030663416945 | 92.7537320772358 | 108.416603471597 |
| YBR258C | 69.6847967035965 | -0.68630691231044 | 0.338607811930961 | -2.02684902157654 | 0.0426778553397943 | 0.0609979219334301 | 93.4854137065579 | 64.0700438925479 | 100.729972928916 | 50.6805382124275 | 41.3824343113821 | 67.7603771697479 |
| YBR259W | 443.704322574843 | -0.217925052742738 | 0.166758489288932 | -1.30683033692607 | 0.191270339542678 | 0.242348002006995 | 440.065484033309 | 468.204166907081 | 522.766501842766 | 464.127034155915 | 390.992655217886 | 376.070093292101 |
| YBR260C | 585.733905119042 | -0.385991854686087 | 0.282627846072222 | -1.36572478632361 | 0.172025361552899 | 0.220592426854904 | 494.78865303227 | 571.701930118119 | 924.215883004722 | 437.45306667569 | 562.230314437399 | 524.01358344605 |
| YBR261C | 981.488169661736 | -0.368259393245011 | 0.235840238847749 | -1.56147820679044 | 0.118410961943897 | 0.15641822793648 | 1061.78148738261 | 1011.9781291746 | 1245.52243898966 | 782.880945544604 | 672.107812436586 | 1114.65820444235 |
| YBR262C | 1698.94564604815 | 2.06654740778499 | 0.136891248247091 | 15.0962711951814 | 1.71353382647437e-51 | 1.18233834026731e-49 | 696.200316709 | 649.735957935966 | 619.084943110561 | 2652.72606590838 | 2535.74433694228 | 3040.18225568269 |
| YBR263W | 7848.67867114621 | 0.608721899720156 | 0.19869308386253 | 3.0636290296914 | 0.00218669883647662 | 0.00379425592653487 | 5370.47100203689 | 5421.31140629251 | 7859.14365428597 | 9446.58558312169 | 8994.25805050488 | 10000.3023306353 |
| YBR264C | 390.903787982843 | -0.330845270705657 | 0.209203622512713 | -1.58145096500684 | 0.113774962270114 | 0.150783955025039 | 499.348917115516 | 467.382756087945 | 339.68793790627 | 360.098560983038 | 358.17210386748 | 320.732451936807 |
| YBR265W | 3003.33864079213 | 1.4283183820777 | 0.19092134070733 | 7.48118768067536 | 7.36539025679635e-14 | 3.80652758610208e-13 | 1413.68186580648 | 1439.93316594406 | 2027.83405356167 | 4165.14002203714 | 4395.09991996748 | 4578.34281743596 |
| YBR267W | 1192.57112663105 | -2.57280735565672 | 0.433890443548074 | -5.92962438770942 | 3.03628429738792e-09 | 1.01407517779907e-08 | 2781.7610907805 | 2554.58764751056 | 789.66416734055 | 350.762672364959 | 308.227786595122 | 370.423395194622 |
| YBR268W | 1315.80685775135 | 2.1255256474071 | 0.110888747381601 | 19.1680914213282 | 6.83715340451785e-82 | 2.60857511657075e-79 | 488.708300921274 | 468.204166907081 | 514.678693797379 | 2215.27299923269 | 2084.81850099756 | 2123.1584846521 |
| YBR269C | 1372.28619001104 | 2.23744252357428 | 0.277798869033233 | 8.05418154278597 | 8.00123875752495e-16 | 5.0141096213823e-15 | 392.942755173093 | 359.77793878123 | 687.463683857927 | 2115.24562118184 | 2368.78761920325 | 2309.49952186891 |
| YBR270C | 196.842749609243 | -0.0661894608978169 | 0.221330082808854 | -0.299053161042639 | 0.764899483781665 | 0.805379553864915 | 188.490915440865 | 170.853450380128 | 244.840007192182 | 186.717772361575 | 181.226522673984 | 208.927829606723 |
| YBR271W | 639.544549252154 | -1.53442839439726 | 0.185012577479096 | -8.29364368252547 | 1.09831127768436e-16 | 7.51439551377717e-16 | 905.972464538349 | 993.085680334492 | 955.096604632564 | 324.088704884734 | 268.272332777236 | 390.751508345546 |
| YBR272C | 733.125419148485 | 0.0301254718641869 | 0.191275567324169 | 0.157497751990096 | 0.874852579437059 | 0.899396707913895 | 765.364321971575 | 734.341272306895 | 675.699599428273 | 754.873279690368 | 873.312062019513 | 595.161979474285 |
| YBR273C | 512.026345382432 | -2.2574395270935 | 0.176292746719694 | -12.8050618593109 | 1.53603364564525e-37 | 4.98135711282756e-36 | 889.251496233111 | 835.374803060528 | 815.398102030419 | 150.707916263271 | 209.766132543903 | 171.659622163361 |
| YBR274W | 1039.88088516535 | 0.656919276058952 | 0.152703205832606 | 4.3019350672904 | 1.69312933859127e-05 | 3.84645775485218e-05 | 734.202517402723 | 783.625921455009 | 902.893479975973 | 1356.37124636944 | 1311.39507352277 | 1150.79707226622 |
| YBR275C | 202.629338644943 | -1.51750130254423 | 0.409873476154348 | -3.70236521958492 | 0.000213598838424743 | 0.000424969958902724 | 408.903679464456 | 347.456776494202 | 144.845289540119 | 121.366552035024 | 87.0458101032521 | 106.157924232605 |
| YBR276C | 1880.40530968812 | -0.124671210952154 | 0.157665215907155 | -0.790733772410334 | 0.429099363912236 | 0.494604313903458 | 1802.06435689633 | 1881.03077581967 | 2200.61904362222 | 1840.50375613553 | 1969.23308102439 | 1588.98084463059 |
| YBR277C | 6.75255081480642 | 1.62655399409304 | 0.809934633313331 | 2.00825341600597 | 0.0446163693773245 | 0.0635724454704145 | 4.56026408324673 | 1.64282163827046 | 3.67627638426699 | 13.3369837401125 | 7.13490246747968 | 10.1640565754622 |
| YBR278W | 203.284736109646 | -1.62668172264417 | 0.228995065492283 | -7.1035667041436 | 1.21577626154761e-12 | 5.59257080311902e-12 | 339.739674201881 | 329.385738473227 | 252.927815237569 | 101.361076424855 | 85.6188296097561 | 110.675282710588 |
| YBR279W | 28.92088473403 | -2.68275021810952 | 0.792081999789292 | -3.38696021222951 | 0.000706716362002083 | 0.00130405756015519 | 68.4039612487009 | 70.6413304456297 | 11.028829152801 | 10.66958699209 | 7.13490246747968 | 5.64669809747899 |
| YBR280C | 558.524081201164 | 0.81934330234695 | 0.300681017324047 | 2.72495852794038 | 0.00643095643680882 | 0.0104486932487831 | 313.89817773015 | 350.742419770743 | 547.029925978928 | 601.497966679074 | 930.39128175935 | 607.584715288739 |
| YBR281C | 1062.01109175833 | -0.413689022880408 | 0.15558216048404 | -2.65897466389051 | 0.00783788568958197 | 0.0126020145222183 | 1073.18214759073 | 1224.72353133063 | 1341.1056249806 | 980.268304898269 | 926.110340278862 | 826.676601470924 |
| YBR282W | 368.115768179358 | 0.0594660281487812 | 0.170928447074411 | 0.347900125266414 | 0.727915191193271 | 0.774738747961857 | 387.622447075972 | 314.600343728793 | 379.391722856354 | 405.44430569942 | 341.048337945529 | 380.587451770084 |
| YBR283C | 12467.566651704 | 0.731329553138254 | 0.10977813393568 | 6.6618872713463 | 2.70333355103697e-11 | 1.10067931023388e-10 | 8896.31518240049 | 9732.89679593333 | 9490.67511362367 | 15470.9011385305 | 16604.3450223187 | 14610.2666574171 |
| YBR284W | 177.696233683795 | 0.610057579504798 | 0.275751371531354 | 2.21234649212047 | 0.0269427330050803 | 0.0399521184890148 | 131.487614400281 | 102.676352391904 | 187.490095597617 | 220.060231711856 | 221.18197649187 | 203.281131509244 |
| YBR285W | 26.1616920267661 | -0.0124237368488458 | 0.586079317264484 | -0.0211980468903653 | 0.983087672289169 | 0.986738879985693 | 14.4408362636146 | 17.2496272018398 | 47.0563377186175 | 24.0065707322025 | 27.1126293764228 | 27.1041508678992 |
| YBR286W | 36300.1102411595 | 0.733783202396037 | 0.17582546094777 | 4.17336145994242 | 3.00138196782591e-05 | 6.61691483457474e-05 | 23885.1431800186 | 24588.1114599939 | 33314.4165942275 | 44026.7170244854 | 45288.0799220805 | 46698.1932661512 |
| YBR287W | 8069.95575821715 | 0.125366624837671 | 0.194320517968609 | 0.645153821882685 | 0.51882750802128 | 0.581447406817565 | 7759.2893376443 | 8330.74852766949 | 7068.00897639172 | 7179.29834730256 | 10446.9241928837 | 7635.46516741109 |
| YBR288C | 1245.25403552978 | -0.135288833418285 | 0.144539808954722 | -0.935997040515427 | 0.349274711467356 | 0.413242571794468 | 1245.71213874023 | 1315.0787214355 | 1349.92868830284 | 1352.37015124741 | 1080.22423357642 | 1128.2102798763 |
| YBR289W | 299.518467516722 | -2.20327622162149 | 0.224831978984134 | -9.79965675513167 | 1.1296887113638e-22 | 1.20314630244755e-21 | 544.951557947984 | 506.810475406436 | 425.712805298118 | 116.031758538979 | 82.7648686227643 | 120.83933928605 |
| YBR290W | 347.947124377561 | -2.18516381365983 | 0.253609800213799 | -8.61624358292816 | 6.91864902531989e-18 | 5.23621441986287e-17 | 726.602077263978 | 533.095621618764 | 452.18199526484 | 130.702440653103 | 108.450517505691 | 136.650093958992 |
| YBR291C | 1142.97116752339 | 0.257963164142223 | 0.232612769436252 | 1.10898109664146 | 0.267438336878212 | 0.327098821986061 | 1186.42870565802 | 1203.36685003311 | 733.784766299692 | 1265.67975693668 | 1168.69702417317 | 1299.86990203966 |
| YBR292C | 107.042893208261 | 1.25611885412482 | 0.309445141491664 | 4.05926183901214 | 4.92280883020636e-05 | 0.000105761305308773 | 46.3626848463417 | 58.3201681586013 | 84.5543568381408 | 166.712296751406 | 159.821815271545 | 126.486037383529 |
| YBR293W | 877.126259023188 | 0.444223894527114 | 0.158366172047931 | 2.80504282437708 | 0.00503098950140311 | 0.00828829004472964 | 719.761681139108 | 809.089656848201 | 699.963023564435 | 1001.60747888245 | 1140.15741430325 | 892.17829940168 |
| YBR294W | 50.4348918268598 | 0.779453732469505 | 0.293838824647441 | 2.65265739952752 | 0.00798608837832019 | 0.0128148860024208 | 33.441936610476 | 34.4992544036796 | 43.3800613343505 | 66.6849187005625 | 61.3601612203252 | 63.2430186917647 |
| YBR295W | 1573.98699876094 | -0.397061759439948 | 0.215370392355403 | -1.84362277050932 | 0.0652381351744291 | 0.0901821280352402 | 1570.25093266462 | 1757.81915294939 | 2038.86288271447 | 1136.31101465759 | 1699.53376775366 | 1241.14424182588 |
| YBR296C | 109.430608699363 | -1.14512487222055 | 0.273112839436733 | -4.19286355991995 | 2.754551295994e-05 | 6.0996994557245e-05 | 140.608142566774 | 188.103077581967 | 123.522886511371 | 62.6838235785288 | 72.7760051682927 | 68.8897167892437 |
| YBR296C-A | 1.8332971744997 | 1.37815544032651 | 1.59167832069277 | 0.865850481475854 | 0.386572161495734 | 0.451604293851104 | 2.28013204162336 | 0 | 0.735255276853398 | 4.00109512203375 | 2.85396098699187 | 1.1293396194958 |
| YBR297W | 552.032113673873 | 1.6004329896094 | 0.163245363285703 | 9.80384960036144 | 1.0837482053819e-22 | 1.15611691778076e-21 | 279.696197105799 | 302.279181441764 | 239.693220254208 | 908.248592701662 | 796.255115370732 | 786.020375169075 |
| YBR298C | 432.240025011622 | 0.190130755824221 | 0.338645381871528 | 0.561444998226349 | 0.574494215595769 | 0.634457599583545 | 447.665924172054 | 499.417778034219 | 263.95664439037 | 426.7834796836 | 623.590475657724 | 332.025848131765 |
| YBR299W | 31.3019381751391 | 0.875723192976555 | 0.364493401387965 | 2.40257625965755 | 0.0162800397163815 | 0.0249922692545444 | 19.0011003468614 | 21.356681297516 | 25.7339346898689 | 41.3446495943488 | 44.236395298374 | 36.1388678238655 |
| YBR300C | 10.9412104711577 | 1.93659510003304 | 0.865094282270676 | 2.23859426622254 | 0.0251823276596626 | 0.0374960002756133 | 2.28013204162336 | 1.64282163827046 | 9.55831859909418 | 10.66958699209 | 25.6856488829268 | 15.8107546729412 |
| YBR301W | 50.3841744659108 | 1.56561288920055 | 0.457793087813918 | 3.41991377955653 | 0.000626409863033777 | 0.001162487659982 | 17.4810123191124 | 19.7138596592455 | 38.9685296732301 | 52.0142365864388 | 98.4616540512196 | 75.6657545062185 |
| YBR302C | 80.0426618570956 | 0.842931922056037 | 0.272319126449082 | 3.09538273366064 | 0.00196559080120277 | 0.00343079169445671 | 60.0434770960819 | 54.2131140629251 | 57.3499115945651 | 85.35669593672 | 128.428244414634 | 94.864528037647 |
| YCL001W | 4388.98963425796 | 1.41431236852846 | 0.123311565152048 | 11.4694219214926 | 1.87910353027528e-30 | 3.54298415621089e-29 | 2239.84970888802 | 2276.95079064285 | 2667.50614442413 | 6460.4349237105 | 6258.73644447317 | 6430.45979340907 |
| YCL001W-A | 3.09937629312486 | -4.70602780501562 | 1.78210521893377 | -2.64071265546891 | 0.00827318538496991 | 0.0132460825492261 | 10.6406161942424 | 5.7498757339466 | 2.20576583056019 | 0 | 0 | 0 |
| YCL001W-B | 5.74319558138406 | -5.59458567150096 | 1.55952126038574 | -3.58737377528098 | 0.000334025341265507 | 0.000647099272236582 | 14.4408362636146 | 15.6068055635694 | 4.41153166112039 | 0 | 0 | 0 |
| YCL002C | 1851.78299316254 | 0.995037576688705 | 0.200877866715383 | 4.95344555853203 | 7.29107640177298e-07 | 1.92157340682241e-06 | 1320.19645209993 | 1455.53997150763 | 936.71522271123 | 2496.68335614906 | 2442.99060486504 | 2458.57235164235 |
| YCL004W | 338.463122318211 | -0.508379380680306 | 0.310303071642178 | -1.63833177025891 | 0.101352503691583 | 0.135624579934724 | 326.818925966015 | 314.600343728793 | 551.441457640049 | 230.729818703946 | 252.575547348781 | 354.612640521681 |
| YCL005W | 1271.56356506993 | 0.706605775039483 | 0.155545178193501 | 4.54276875211426 | 5.55201458524209e-06 | 1.33605998060398e-05 | 1023.77928668889 | 1062.08418914185 | 813.192336199859 | 1621.77722279768 | 1556.83571840407 | 1551.71263718723 |
| YCL005W-A | 5553.58729322775 | 1.87088088675513 | 0.16303669963471 | 11.4752131940043 | 1.75743980010128e-30 | 3.32325205348598e-29 | 2740.71871403128 | 2417.41204071498 | 1996.21807665698 | 8519.66521318387 | 9228.28285143822 | 8419.22686334117 |
| YCL007C | 6.30856527395362 | -0.527941203208235 | 0.861536701718456 | -0.612790148295694 | 0.540015105792406 | 0.602535347698185 | 9.12052816649345 | 6.57128655308183 | 6.61729749168058 | 1.33369837401125 | 8.56188296097562 | 5.64669809747899 |
| YCL008C | 745.785661322028 | -1.0180871758868 | 0.318866567538993 | -3.19283135809557 | 0.00140885199645756 | 0.00250419677967216 | 741.802957541467 | 753.233721147005 | 1500.65602005779 | 452.123748789814 | 478.038465321139 | 548.859055074958 |
| YCL009C | 23694.7578454267 | 0.520530110410382 | 0.182447067075631 | 2.85304729066761 | 0.00433021770130083 | 0.0071904229417914 | 17189.1554177847 | 17468.1224797298 | 23740.6576343194 | 26275.1916663956 | 27075.5278835919 | 30419.8919907388 |
| YCL010C | 281.698000948515 | -0.658633247322398 | 0.149063177017085 | -4.41848389724652 | 9.93956841606221e-06 | 2.3298894378959e-05 | 354.94055447937 | 354.028063047284 | 325.718087646055 | 216.059136589823 | 222.608956985366 | 216.833206943193 |
| YCL011C | 3483.50465455968 | 0.187773696089071 | 0.106051124378841 | 1.77059599498725 | 0.07662790843872 | 0.105009214902501 | 3187.62459418946 | 3237.18003821194 | 3345.41150968296 | 3759.69571633771 | 3914.20749365935 | 3456.90857527664 |
| YCL012C | 823.442116063365 | 1.79261111978191 | 0.254985727240345 | 7.03024102244055 | 2.06177072698664e-12 | 9.26811060942892e-12 | 286.536593230669 | 321.99304110101 | 497.767822429751 | 1173.6545691299 | 1445.53123991138 | 1215.16943057748 |
| YCL014W | 397.047684888242 | -1.9342808361667 | 0.34679993169654 | -5.57751216012134 | 2.43982734219352e-08 | 7.41899678456032e-08 | 808.686830762419 | 735.984093945165 | 343.364214290537 | 165.378598377395 | 179.799542180488 | 149.072829773445 |
| YCL016C | 317.398953077467 | -1.60360933811669 | 0.2186222650124 | -7.33506872241826 | 2.21607743419135e-13 | 1.08643070583258e-12 | 490.228388949023 | 491.203669842867 | 452.917250541693 | 153.375313011294 | 121.293341947155 | 195.375754172773 |
| YCL017C | 3108.44765562352 | 0.826941452128282 | 0.156015468459055 | 5.30038117563519 | 1.15561156644144e-07 | 3.31503609904431e-07 | 1965.47381987934 | 2136.48954057073 | 2621.18506198236 | 4031.77018463601 | 4052.62460152846 | 3843.1427251442 |
| YCL018W | 9921.98463486924 | 1.19308767644447 | 0.120664688739925 | 9.88762900649416 | 4.7105203440094e-23 | 5.20484411435179e-22 | 6220.20020954853 | 5623.37846779978 | 6270.25700100578 | 12944.8764181532 | 14977.5872597333 | 13495.6084529748 |
| YCL019W | 8.85889862187319 | -4.31848379058583 | 1.39472054590503 | -3.09630757449235 | 0.00195946994497551 | 0.00342102881914163 | 28.1216285133548 | 18.8924488401103 | 3.67627638426699 | 1.33369837401125 | 0 | 1.1293396194958 |
| YCL021W-A | 327.027250151078 | 0.851872129362243 | 0.282465094634391 | 3.01584919887133 | 0.00256260673756838 | 0.00440060029120162 | 171.769947135627 | 199.602829049861 | 327.923853476616 | 426.7834796836 | 429.521128542277 | 406.562263018487 |
| YCL022C | 1.93303769069267 | 2.01398550493269 | 1.85108061856231 | 1.08800528984897 | 0.276592772273504 | 0.337087696536255 | 2.28013204162336 | 0 | 0 | 5.334793496045 | 2.85396098699187 | 1.1293396194958 |
| YCL023C | 6.17364784996604 | 0.517265708003195 | 1.19468047420565 | 0.432974104098526 | 0.665033582944875 | 0.718542032147336 | 0 | 4.92846491481137 | 10.2935738759476 | 9.33588861807875 | 5.70792197398374 | 6.77603771697479 |
| YCL024W | 102.440153101441 | -2.43807853999654 | 0.600093430364729 | -4.06283158026693 | 4.848100973412e-05 | 0.000104294470691709 | 262.215184786687 | 210.281169698619 | 46.3210824417641 | 33.3424593502813 | 34.2475318439025 | 28.2334904873949 |
| YCL025C | 12840.5207606144 | 1.17302712050085 | 0.123443059344864 | 9.50257654603126 | 2.04760096955137e-21 | 1.98516291308075e-20 | 7531.27613348197 | 7833.79498209268 | 8304.70835205913 | 16485.8456011531 | 19572.4644487903 | 17315.0350461096 |
| YCL026C-A | 42.825306945203 | -0.0677048167961025 | 0.357339944830727 | -0.189468929448048 | 0.849725304733938 | 0.878189125306516 | 36.4821126659738 | 46.8204166907081 | 47.7915929954709 | 40.0109512203375 | 54.2252587528456 | 31.6215093458823 |
| YCL026C-B | 241.21548238225 | 1.06306553766914 | 0.277181987572041 | 3.83526197708949 | 0.000125430526556131 | 0.00025542932346721 | 135.287834469653 | 119.104568774608 | 213.959285564339 | 310.751721144621 | 306.800806101626 | 361.388678238655 |
| YCL027W | 64.4242099728389 | -1.94585397789285 | 0.448224253965387 | -4.34125097131204 | 1.41673787186077e-05 | 3.25042866533037e-05 | 78.2845334290688 | 79.6768494561172 | 149.25682120124 | 32.00876097627 | 15.6967854284553 | 31.6215093458823 |
| YCL028W | 1497.43091248151 | -1.0141044311624 | 0.273563780839207 | -3.70701277797613 | 0.000209718365266752 | 0.000417762075282602 | 1555.05005238713 | 1722.49848772658 | 2732.20860878723 | 897.579005709572 | 879.019983993497 | 1198.22933628504 |
| YCL029C | 179.316620983193 | -2.58154028826011 | 0.655345326741309 | -3.93920606880157 | 8.17516909719106e-05 | 0.000169949188347376 | 454.506320296924 | 409.883998748479 | 57.3499115945651 | 58.682728456495 | 57.0792197398374 | 38.3975470628571 |
| YCL030C | 4788.29860114632 | -0.0661468299703201 | 0.129116547991161 | -0.512303271729727 | 0.608438775830346 | 0.666497871987101 | 4500.98065016452 | 4714.89810183621 | 5477.65181255782 | 4677.28019765746 | 4830.32897048374 | 4528.65187417815 |
| YCL031C | 279.572630494345 | -4.75733231160206 | 0.402485271959895 | -11.8198916656908 | 3.08081559750273e-32 | 6.57308222546143e-31 | 759.28396986058 | 518.31022687433 | 340.423193183123 | 20.0054756101688 | 15.6967854284553 | 23.7161320094118 |
| YCL032W | 345.261362434211 | -2.90547428880771 | 0.285296494539695 | -10.1840518352512 | 2.33626214732573e-24 | 2.9252888586013e-23 | 690.119964598004 | 625.093633361909 | 511.737672689965 | 57.3490300824838 | 112.731458986179 | 74.5364148867227 |
| YCL033C | 1978.07678697298 | 2.22696393902906 | 0.225970759077588 | 9.85509783708086 | 6.51532097949944e-23 | 7.10224737361905e-22 | 611.835431168936 | 571.701930118119 | 905.099245806533 | 2898.12656672645 | 3349.12321823496 | 3532.57432978286 |
| YCL034W | 1433.85032028922 | 0.816627979029773 | 0.249912087293137 | 3.26766099181069 | 0.00108440172158594 | 0.00195264563192849 | 891.531628274735 | 810.732478486471 | 1413.16064211223 | 1701.79912523836 | 1820.82710970081 | 1965.05093792269 |
| YCL035C | 3386.69347939517 | 1.54791450185265 | 0.200287972788686 | 7.72844460054411 | 1.08868542394867e-14 | 6.05592938227367e-14 | 1574.05115273399 | 1429.2548252953 | 2174.88510893235 | 4843.99249440886 | 4957.33023440488 | 5340.64706059563 |
| YCL036W | 937.585062799972 | 0.452764938153238 | 0.273064721600003 | 1.65808653531036 | 0.0973000012217499 | 0.130687059002748 | 696.200316709 | 678.485336605699 | 1000.68243179748 | 1157.65018864177 | 773.423427474797 | 1319.06867557109 |
| YCL037C | 191.575673326395 | -0.257847479324826 | 0.306887083786369 | -0.840203100578581 | 0.40079452025464 | 0.465786285320121 | 233.333512259457 | 262.030051304138 | 130.875439279905 | 178.715582117508 | 162.675776258537 | 181.823678738823 |
| YCL038C | 919.235114641237 | 0.532630702896905 | 0.166304898147717 | 3.20273611198033 | 0.00136128657476503 | 0.00242359901484904 | 773.724806124194 | 823.0536407735 | 657.318217506938 | 1016.27816099657 | 1228.6302049 | 1016.40565754622 |
| YCL039W | 940.943635679244 | 0.144861152910567 | 0.29583137158713 | 0.489674749954305 | 0.62436407215123 | 0.681410966174134 | 696.960360722874 | 685.056623158781 | 1299.19607419995 | 1038.95103335476 | 866.177159552033 | 1059.32056308706 |
| YCL040W | 6680.32963895877 | 1.69613484162932 | 0.304713198679798 | 5.56633204264863 | 2.601572968558e-08 | 7.88864061433717e-08 | 2528.66643416031 | 2526.65967965996 | 4396.82655558332 | 8296.93758472399 | 13363.6723215894 | 8969.21525803563 |
| YCL041C | 9.86242656477176 | 2.44899539992634 | 0.802066181784783 | 3.05335825838805 | 0.00226295597909561 | 0.00391400866144377 | 0.760044013874454 | 2.46423245740569 | 5.88204221482719 | 16.004380488135 | 17.1237659219512 | 16.940094292437 |
| YCL042W | 59.1693463330616 | 1.45165946282152 | 0.554495024148663 | 2.61798465198188 | 0.00884507833541449 | 0.014061073059681 | 22.8013204162336 | 13.9639839252989 | 58.0851668714185 | 65.3512203265513 | 105.596556518699 | 89.217829940168 |
| YCL043C | 29426.6862190813 | 1.33022607792947 | 0.263561252293289 | 5.04712307425677 | 4.48512447486471e-07 | 1.20657475503826e-06 | 12961.0305686011 | 13690.4541225269 | 23586.989281457 | 39396.1162699183 | 43587.1191738334 | 43338.4078981512 |
| YCL044C | 1082.4631010335 | 0.211591414588202 | 0.245026051548492 | 0.863546603518306 | 0.387837007752292 | 0.452918767065424 | 804.126566679173 | 912.58742005924 | 1293.31403198513 | 1028.28144636267 | 1067.38140913496 | 1389.08773197983 |
| YCL045C | 5005.60876533242 | -0.317961889103736 | 0.1268888212772 | -2.50583058383937 | 0.0122164138513939 | 0.0190333077684701 | 4985.88873101642 | 5600.37896486399 | 6078.35537374704 | 4341.18820740662 | 4623.41679892683 | 4404.42451603361 |
| YCL046W | 2.17537816699054 | 0.143889546371519 | 1.43668852289268 | 0.100153613033538 | 0.920222372213127 | 0.936391952647371 | 1.52008802774891 | 1.64282163827046 | 2.94102110741359 | 2.6673967480225 | 4.28094148048781 | 0 |
| YCL047C | 526.249764043129 | 1.08889851073837 | 0.149787169059775 | 7.26963809766528 | 3.6045186438287e-13 | 1.74599760447147e-12 | 353.420466451621 | 298.172127346088 | 358.069319827605 | 729.533010584154 | 670.68083194309 | 747.622828106218 |
| YCL048W | 72.9011511744203 | 0.603588983352143 | 0.245168133802806 | 2.46193897220599 | 0.0138188177940322 | 0.0213809284857092 | 61.5635651238308 | 51.7488816055194 | 60.2909327019787 | 90.691489432765 | 82.7648686227643 | 90.3471695596638 |
| YCL048W-A | 76.2064712370641 | 2.76471053003571 | 0.40788480654285 | 6.77816502524046 | 1.21711783022782e-11 | 5.08976547186178e-11 | 15.9609242913635 | 11.4997514678932 | 30.8807216278427 | 134.703535775136 | 151.259932310569 | 112.93396194958 |
| YCL049C | 1908.67766445372 | 1.64070265883612 | 0.271381496311577 | 6.04574254743 | 1.48723423928806e-09 | 5.14525326501245e-09 | 729.642253319476 | 745.019612955653 | 1305.81337169164 | 2632.72059029821 | 3212.13309085935 | 2826.73706759798 |
| YCL050C | 5464.21004169315 | 0.20560656103085 | 0.186788721744633 | 1.10074398020638 | 0.271008099112082 | 0.331027971909786 | 4431.81664490194 | 4473.40332101046 | 6320.98961510867 | 5802.92162532295 | 5894.85641863171 | 5861.27262518319 |
| YCL051W | 290.288524126396 | -3.79227099175002 | 0.262878877969832 | -14.4259250535345 | 3.55471696065546e-47 | 1.98757708679408e-45 | 618.675827293806 | 561.023589469361 | 445.564697773159 | 37.343554472315 | 32.8205513504065 | 46.3029243993277 |
| YCL052C | 2246.18699149679 | 0.658443159942992 | 0.164141335040051 | 4.01144026142063 | 6.03494486497682e-05 | 0.000128168475423182 | 1638.65489391332 | 1607.50097304764 | 1980.0424605662 | 2554.03238623154 | 3126.5142612496 | 2570.37697397244 |
| YCL054W | 326.776222932143 | -3.14586895448379 | 0.535993118244511 | -5.86923385282849 | 4.37813544604919e-09 | 1.44145109152665e-08 | 845.168943428393 | 747.483845413058 | 169.108713676282 | 66.6849187005625 | 59.9331807268293 | 72.2777356477311 |
| YCL054W-A | 322.967694160852 | 1.50012488484456 | 0.194162044857797 | 7.72614898006065 | 1.1084889136227e-14 | 6.15026440868847e-14 | 179.370387274371 | 186.460255943697 | 140.433757878999 | 532.145651230489 | 462.341679892683 | 437.054432744874 |
| YCL055W | 308.648907411962 | -1.72903034771637 | 0.167591888727933 | -10.3169094926978 | 5.90947487173878e-25 | 7.69655703174653e-24 | 506.949357254261 | 470.668399364486 | 445.564697773159 | 152.041614637283 | 129.85522490813 | 146.814150534454 |
| YCL056C | 206.306985024923 | -0.271057809207667 | 0.333943085707238 | -0.811688640396941 | 0.416970314186617 | 0.482252043119544 | 282.736373161297 | 258.744408027597 | 135.286970941025 | 165.378598377395 | 224.035937478862 | 171.659622163361 |
| YCL057C-A | 4147.11758955443 | 2.27578863728238 | 0.11784947329142 | 19.3109784347935 | 4.34267147495213e-83 | 1.8777711457693e-80 | 1482.84587106906 | 1324.93565126512 | 1450.65866123175 | 6381.74671964383 | 7224.80223856992 | 7017.71639554689 |
| YCL057W | 3114.23099272565 | 1.07424646610788 | 0.266868071147759 | 4.02538400898883 | 5.6882413530291e-05 | 0.000121321714619358 | 1611.29330941384 | 1577.10877273964 | 2827.79179477817 | 4233.15863911171 | 4514.96628142114 | 3921.06715888941 |
| YCL058C | 1154.75188501549 | 2.4507212990783 | 0.220257491509437 | 11.1266194955884 | 9.30995986935426e-29 | 1.56032040601116e-27 | 332.139234063136 | 279.279678505978 | 459.534548033374 | 1857.84183499767 | 1936.41252967399 | 2063.30348481882 |
| YCL058W-A | 93.3832839568636 | -2.33848236959745 | 0.517978499030421 | -4.51463212078251 | 6.34267703757092e-06 | 1.51810586038691e-05 | 213.572367898722 | 174.139093656669 | 80.1428251770204 | 49.3468398384163 | 17.1237659219512 | 25.9748112484034 |
| YCL059C | 667.64757458019 | -4.64012881468604 | 0.28259249366543 | -16.4198586965287 | 1.37887460631973e-60 | 1.59703226724818e-58 | 1635.61471785783 | 1250.18726672382 | 966.125433785365 | 54.6816333344613 | 42.8094148048781 | 56.4669809747899 |
| YCL061C | 17.4979593799507 | -2.52705484596887 | 0.770537064602657 | -3.27960193228602 | 0.00103953649416464 | 0.00187550311575851 | 37.2421566798483 | 41.8919517758967 | 10.2935738759476 | 5.334793496045 | 5.70792197398374 | 4.51735847798319 |
| YCL063W | 443.145916190307 | -1.04184849779754 | 0.191160354350829 | -5.45012851297334 | 5.03334353897245e-08 | 1.4900167135452e-07 | 601.954858988568 | 594.701433053906 | 594.086263697546 | 317.420213014678 | 225.462917972358 | 325.24981041479 |
| YCL064C | 3568.6491274227 | -1.63784835281312 | 0.619765606590794 | -2.64268997084655 | 0.00822503100779724 | 0.013178742864766 | 7522.15560531547 | 7787.79597622111 | 894.805671930586 | 1628.44571466774 | 1877.90632944065 | 1700.78546696067 |
| YCL068C | 8.00169600701779 | -2.39075168264353 | 0.909621606452522 | -2.62829254019959 | 0.00858146817068532 | 0.0136990899717118 | 14.4408362636146 | 15.6068055635694 | 10.2935738759476 | 0 | 4.28094148048781 | 3.38801885848739 |
| YCR001W | 1.64092038912557 | 1.10290718552663 | 1.6909439319558 | 0.652243498251874 | 0.51424410311164 | 0.576857013625406 | 2.28013204162336 | 0.821410819135229 | 0 | 1.33369837401125 | 4.28094148048781 | 1.1293396194958 |
| YCR002C | 3226.18090512315 | 0.39865689861732 | 0.118651124152152 | 3.35990831495286 | 0.000779683427278617 | 0.00142853861845455 | 2636.59268413048 | 2939.82932168498 | 2772.64764901416 | 3773.03270007783 | 3857.12827391951 | 3377.85480191193 |
| YCR003W | 594.049307720958 | 0.222470950944955 | 0.234823799060189 | 0.947395246288182 | 0.343437412548687 | 0.407525623452394 | 521.390193517876 | 502.70342131076 | 621.290708941122 | 758.874374812401 | 466.622621373171 | 693.41452637042 |
| YCR004C | 4478.81636820528 | 1.19390051262372 | 0.288392968303693 | 4.13983919110843 | 3.4754936569969e-05 | 7.60528065427865e-05 | 2072.64002583564 | 2120.88273500716 | 3979.93681360744 | 5736.23670662239 | 6614.05458735366 | 6349.14734080538 |
| YCR005C | 9587.03270888642 | 0.263011170718533 | 0.312454358431604 | 0.841758687696799 | 0.399923040610843 | 0.465168655824141 | 9350.06145868354 | 9702.50459562533 | 7093.74291108159 | 7111.27973022799 | 15310.0737147179 | 8954.53384298218 |
| YCR006C | 112.335698814949 | 0.42092643129722 | 0.239188339602921 | 1.75981166973275 | 0.0784397437770201 | 0.107356019864476 | 101.085853845302 | 98.5692982962275 | 88.9658884992612 | 144.039424393215 | 101.315615038211 | 140.038112817479 |
| YCR007C | 249.18424625559 | -1.13252967012007 | 0.266917013623329 | -4.243002927188 | 2.20548557818741e-05 | 4.9362576670006e-05 | 288.056681258418 | 298.993538165223 | 438.947400281479 | 165.378598377395 | 178.372561686992 | 125.356697764034 |
| YCR008W | 1746.56127653199 | -0.067265595747122 | 0.161987548877385 | -0.41525164256938 | 0.677957697320757 | 0.729710193299441 | 1598.37256117798 | 1694.57051987598 | 2068.27309378861 | 1847.17224800558 | 1696.67980676667 | 1574.29942957714 |
| YCR009C | 2053.83567335233 | -0.979524034411748 | 0.13328582151639 | -7.34904900812198 | 1.99621992613644e-13 | 9.8459942516509e-13 | 2964.17165411037 | 2798.54666079373 | 2413.84307390971 | 1347.03535775136 | 1401.29484461301 | 1398.1224489358 |
| YCR010C | 31.1095613435849 | -0.415014282231936 | 0.542748099225308 | -0.764653589435517 | 0.444477849139861 | 0.509974054399635 | 50.162904915714 | 41.0705409567615 | 15.4403608139214 | 32.00876097627 | 24.2586683894309 | 23.7161320094118 |
| YCR011C | 2596.0462561052 | 0.460141731044897 | 0.17513156918917 | 2.62740597355049 | 0.0086038616381952 | 0.0137314583133204 | 1941.15241143536 | 1993.5640580412 | 2621.18506198236 | 2960.81039030498 | 3246.38062270325 | 2813.18499216403 |
| YCR012W | 225910.999492345 | 1.91557199086948 | 0.34437118344376 | 5.56252114858591 | 2.65904794593982e-08 | 8.04411612750263e-08 | 65570.5171649769 | 67340.0803635252 | 151097.165159204 | 335079.713184961 | 385826.985831431 | 350551.535249974 |
| YCR013C | 28.0964895974067 | 1.35833027231421 | 0.591686249692576 | 2.2956934913055 | 0.0216934128651375 | 0.0326609739654786 | 28.1216285133548 | 6.57128655308183 | 12.4993397065078 | 33.3424593502813 | 48.5173367788618 | 39.5268866823529 |
| YCR014C | 109.318573126107 | -2.63143122382506 | 0.312599834155637 | -8.41789065862053 | 3.83323288775389e-17 | 2.70536980521999e-16 | 196.851399593484 | 165.103574646181 | 203.665711688391 | 33.3424593502813 | 18.5507464154472 | 38.3975470628571 |
| YCR015C | 235.862530433458 | -0.194396287823856 | 0.204764848920109 | -0.949363569231072 | 0.342435732376023 | 0.406485754061289 | 238.653820356579 | 286.672375878195 | 229.39964637826 | 244.066802444059 | 231.170839946342 | 185.211697597311 |
| YCR016W | 71.9435961628818 | -2.15087059374924 | 0.453935226218994 | -4.73827645337132 | 2.15543630268347e-06 | 5.42918829483689e-06 | 145.168406650021 | 142.92548252953 | 63.9672090862457 | 21.33917398418 | 35.6745123373984 | 22.586792389916 |
| YCR017C | 4980.68376203871 | 0.579084531841317 | 0.144797002712185 | 3.99928535117795 | 6.35340405376837e-05 | 0.000134272332006327 | 4124.75886329666 | 4275.44331359887 | 3582.16370882976 | 6037.65253914893 | 6509.88501132846 | 5354.19913602958 |
| YCR018C | 567.346646978563 | 1.1301329997104 | 0.166663527217394 | 6.78092572849649 | 1.19408255745001e-11 | 4.9998834523052e-11 | 321.498617868894 | 349.099598132472 | 396.302594223982 | 822.891896764942 | 827.648686227643 | 686.638488653445 |
| YCR018C-A | 48.9364415419731 | 1.04634865102901 | 0.313124210262361 | 3.34164084646248 | 0.000832847496729527 | 0.00152164756726414 | 34.9620246382249 | 33.6778435845444 | 27.2044452435757 | 54.6816333344613 | 74.2029856617887 | 68.8897167892437 |
| YCR019W | 459.563587571265 | 0.113390841540093 | 0.164045742464025 | 0.691214778493624 | 0.489430577979433 | 0.553135864919778 | 446.905880158179 | 451.775950524376 | 424.977550021264 | 462.793335781904 | 550.814470489431 | 420.114338452437 |
| YCR020C | 449.607028823293 | 1.96525811929227 | 0.14402573365598 | 13.6451873523275 | 2.15658548565185e-42 | 9.38766004022677e-41 | 180.130431288246 | 176.603326114074 | 193.372137812444 | 689.522059363816 | 687.804597865041 | 770.209620496134 |
| YCR020C-A | 1221.15666822727 | 1.77840893908156 | 0.215756480384088 | 8.24266754776336 | 1.68413040362607e-16 | 1.1214856055358e-15 | 656.678027987528 | 591.415789777365 | 405.860912823076 | 1985.87687890275 | 1877.90632944065 | 1809.20207043227 |
| YCR020W-B | 71.5438101977837 | -2.8121408237651 | 0.494984890918258 | -5.68126598480256 | 1.33701358933733e-08 | 4.17921452551419e-08 | 179.370387274371 | 128.961498604231 | 67.6434854705126 | 22.6728723581913 | 11.4158439479675 | 19.1987735314286 |
| YCR021C | 12158.1368484325 | 0.348542642219499 | 0.274549655842264 | 1.26950675334208 | 0.204260383431506 | 0.257199154909095 | 13532.5836670347 | 8883.5580089475 | 9673.01842228331 | 11179.0597709623 | 18281.0471021764 | 11399.5541191906 |
| YCR022C | 39.7019794555534 | 1.01799069548628 | 0.366107414966791 | 2.78057928867302 | 0.00542620071758553 | 0.00890094533491648 | 34.2019806243504 | 23.8209137549216 | 20.5871477518952 | 57.3490300824838 | 57.0792197398374 | 45.1735847798319 |
| YCR023C | 1732.19627454422 | 0.712741969380193 | 0.123994969468315 | 5.74815230356845 | 9.0223951904414e-09 | 2.86859094143152e-08 | 1280.67416337846 | 1361.89913812621 | 1295.51979781569 | 2356.64502687788 | 2111.93113037399 | 1986.50839069311 |
| YCR024C | 455.628686422031 | 0.343847500541301 | 0.213270826290571 | 1.61225755309273 | 0.106905905933677 | 0.142382310236046 | 335.939454132509 | 360.599349600366 | 508.061396305698 | 524.143460986421 | 492.308270256098 | 512.720187251092 |
| YCR024C-A | 9578.84018360963 | 0.724683591181461 | 0.134555168462072 | 5.38577298415508 | 7.21339618161491e-08 | 2.10843116872259e-07 | 7682.52489224298 | 7717.15464577548 | 6268.05123517522 | 11765.8870555273 | 11793.9937787439 | 12245.4294941929 |
| YCR024C-B | 24913.0508123086 | 1.02900574992014 | 0.136245050579514 | 7.55261013551167 | 4.2662060722166e-14 | 2.2569830819247e-13 | 15165.158208837 | 15414.5954318917 | 18580.6361013622 | 34529.4509031513 | 31657.5622482073 | 34130.901980402 |
| YCR025C | 14.4125738979084 | 0.170348130901875 | 0.517361613871617 | 0.329263181369591 | 0.741956770913739 | 0.786971646140068 | 15.9609242913635 | 12.3211622870284 | 12.4993397065078 | 17.3380788621463 | 11.4158439479675 | 16.940094292437 |
| YCR026C | 636.846090808791 | -4.16488976039473 | 0.241400123682702 | -17.2530556192634 | 1.06134792632905e-66 | 1.96682932862006e-64 | 1331.59711230804 | 1291.25780768058 | 997.741410690062 | 62.6838235785288 | 54.2252587528456 | 83.571131842689 |
| YCR027C | 597.900541672631 | -0.04885257309957 | 0.138746392826626 | -0.352099770699011 | 0.724763440350196 | 0.771764188821437 | 623.996135390927 | 653.843012031642 | 546.294670702075 | 590.828379686984 | 596.477846281301 | 575.963205942857 |
| YCR028C | 1063.96830359254 | 0.12144214243183 | 0.163127028834973 | 0.744463644677099 | 0.456596027583731 | 0.521057919515962 | 1023.01924267502 | 1165.58195235289 | 869.071737240717 | 1153.64909351973 | 1124.4606288748 | 1048.0271668921 |
| YCR028C-A | 6207.82410339612 | 1.34879157599893 | 0.0877782818126333 | 15.3658917461837 | 2.77198147659599e-53 | 2.19256973868312e-51 | 3516.7236521971 | 3614.20760419501 | 3370.41018909598 | 8998.46292945391 | 8882.9535720122 | 8864.18667342252 |
| YCR030C | 1935.37872188403 | -0.46739087600484 | 0.1667675243496 | -2.80264924377622 | 0.00506847595675397 | 0.00834792662658869 | 2027.79742901704 | 2084.74065896521 | 2625.59659364349 | 1483.07259190051 | 1758.03996798699 | 1633.02508979092 |
| YCR031C | 114042.641078966 | 1.07621710692421 | 0.255506932250077 | 4.21208574439329 | 2.53023456110928e-05 | 5.62794971308464e-05 | 87788.8838225688 | 84181.4663882548 | 48154.0738469596 | 143389.913285072 | 162015.084290048 | 158726.424840895 |
| YCR032W | 680.854890275398 | -0.717516297702516 | 0.137108798501629 | -5.23318930326702 | 1.66609950316665e-07 | 4.69635870384132e-07 | 883.171144122116 | 887.945095485183 | 769.812274865508 | 526.810857734444 | 486.600348282114 | 530.789621163025 |
| YCR033W | 680.551088854008 | -0.024509097266556 | 0.159597634421977 | -0.153568048519778 | 0.877950331626004 | 0.902152384494021 | 710.641152972615 | 721.198699200731 | 627.172751155949 | 586.82728456495 | 746.310798098374 | 691.155847131428 |
| YCR034W | 11970.0462251905 | 0.594457573567527 | 0.163521225807238 | 3.6353541910717 | 0.000277598970325656 | 0.000544124182995529 | 10297.0762999711 | 10511.5942524735 | 7806.20527435253 | 14869.4031718514 | 14558.0549946455 | 13777.9433578487 |
| YCR035C | 769.887109658544 | -4.11648863516743 | 0.268761944214436 | -15.3164862949608 | 5.93397559430294e-53 | 4.58187686960106e-51 | 1650.81559813531 | 1609.14379468591 | 1108.76495749492 | 81.3556008146863 | 64.2141222073171 | 105.028584613109 |
| YCR036W | 1657.26318149112 | 0.853598099289634 | 0.129000848417809 | 6.61699601017346 | 3.66571385434984e-11 | 1.47218700057666e-10 | 1100.54373209021 | 1150.79655760846 | 1290.37301087771 | 2112.57822443382 | 2268.89898465854 | 2020.38857927798 |
| YCR037C | 1839.82446150408 | -0.49595669472744 | 0.175938711670408 | -2.81891739469215 | 0.00481859169733855 | 0.00796264605068479 | 2413.89978806527 | 2319.66415323789 | 1725.64413477493 | 1489.74108377057 | 1538.28497198862 | 1551.71263718723 |
| YCR038C | 148.783050253283 | -2.17306335220593 | 0.253156655440168 | -8.58386815241965 | 9.17351080112992e-18 | 6.82332466240007e-17 | 233.333512259457 | 211.102580517754 | 286.014302695972 | 46.6794430903938 | 61.3601612203252 | 54.2083017357983 |
| YCR041W | 21.8421085283419 | 2.83588153167657 | 0.498587999068331 | 5.68782549314413 | 1.28667202557118e-08 | 4.03352090761464e-08 | 5.32030809712118 | 4.10705409567614 | 6.61729749168058 | 38.6772528463263 | 35.6745123373984 | 40.6562263018487 |
| YCR042C | 573.914982179874 | -1.61943949275661 | 0.198800322399109 | -8.14606069654877 | 3.75971469033159e-16 | 2.42400690670881e-15 | 962.215721565059 | 911.766009240104 | 724.961702977451 | 293.413642282475 | 238.305742413821 | 312.827074600336 |
| YCR043C | 2557.18689534897 | 0.619565040214935 | 0.192319253921186 | 3.22154452860366 | 0.00127501648035343 | 0.00227754252039999 | 2155.48482334795 | 2334.44954798232 | 1559.47644220606 | 3239.55335047333 | 3058.01919756179 | 2996.13801052235 |
| YCR044C | 1682.0795514408 | 1.34058720681036 | 0.10221390701562 | 13.1155069398286 | 2.68381591376358e-39 | 9.61725415285666e-38 | 930.293872982332 | 981.585928866599 | 945.53828603347 | 2491.34856265302 | 2308.85443847642 | 2434.85621963294 |
| YCR045C | 20.7228002064817 | 0.455493572630842 | 0.515599870272708 | 0.883424529160423 | 0.377006942572037 | 0.442262078047067 | 9.12052816649345 | 20.5352704783807 | 22.7929135824553 | 25.3402691062138 | 22.831687895935 | 23.7161320094118 |
| YCR045W-A | 296.649673307654 | 0.985363299989548 | 0.231823158291511 | 4.25049553828648 | 2.1329808494324e-05 | 4.78537315441665e-05 | 167.20968305238 | 174.139093656669 | 255.868836344983 | 409.445400821454 | 361.026064854472 | 412.208961115966 |
| YCR046C | 2780.6253251686 | 2.6007052739501 | 0.362637212671364 | 7.17164478182487 | 7.41020120175443e-13 | 3.47272868457942e-12 | 598.91468293307 | 493.667902300273 | 1268.31535257211 | 4315.84793830041 | 4520.67420339513 | 5486.33187151059 |
| YCR047C | 1860.34479704156 | 0.594096144577006 | 0.171060925468715 | 3.47300906357869 | 0.00051465797480383 | 0.000968399078786667 | 1554.29000837326 | 1399.68403580643 | 1494.77397784296 | 2139.25219191405 | 1917.86178325854 | 2656.20678505412 |
| YCR047W-A | 3.86354148567066 | 1.2459811524562 | 1.07860032373532 | 1.15518336592114 | 0.248015344983041 | 0.305764593719826 | 0.760044013874454 | 2.46423245740569 | 3.67627638426699 | 2.6673967480225 | 5.70792197398374 | 7.90537733647059 |
| YCR048W | 2718.98821105135 | 0.896938441510579 | 0.147115839606524 | 6.09681760923588 | 1.08200853125539e-09 | 3.79346342363377e-09 | 1695.65819495391 | 1854.74562960735 | 2149.15117424248 | 3336.91333177615 | 3790.06019072521 | 3487.40074500302 |
| YCR049C | 1.61904624786893 | 0.162106200163328 | 1.92725664995598 | 0.084112409297968 | 0.932967058416168 | 0.946687162216406 | 0 | 1.64282163827046 | 2.94102110741359 | 4.00109512203375 | 0 | 1.1293396194958 |
| YCR050C | 2.05263830570323 | 1.61924741426906 | 1.67677001738579 | 0.965694399040832 | 0.334197122985081 | 0.397871244434881 | 1.52008802774891 | 0.821410819135229 | 0.735255276853398 | 1.33369837401125 | 0 | 7.90537733647059 |
| YCR051W | 3130.65990032417 | 0.961124518689178 | 0.137514297180112 | 6.98926975884063 | 2.76320673733214e-12 | 1.22670492117291e-11 | 2082.520598016 | 1966.45750100974 | 2325.6124406873 | 3925.07431471511 | 3934.1852205683 | 4550.10932694857 |
| YCR052W | 814.471148535033 | -1.97998598447368 | 0.203775754259384 | -9.71649444591617 | 2.56458943905932e-22 | 2.65717685331289e-21 | 1400.00107355674 | 1484.28935017736 | 1013.91702678084 | 340.093085372869 | 342.475318439025 | 306.051036883361 |
| YCR053W | 12254.7560979858 | -0.172726955883967 | 0.127270820746073 | -1.35716069772652 | 0.174730155681731 | 0.223751192448511 | 11977.5336146475 | 12632.4769874807 | 14352.9182594552 | 11705.8706286967 | 10837.9168481016 | 12021.8202495328 |
| YCR054C | 738.47862907636 | 0.0907424628267438 | 0.166835928550396 | 0.543902405286362 | 0.586508633699842 | 0.645346874381366 | 736.482649444346 | 713.806001828514 | 695.551491903315 | 897.579005709572 | 663.54592947561 | 723.906696096806 |
| YCR057C | 3309.61103236623 | -0.0680348587347922 | 0.147607137725486 | -0.460918487975295 | 0.64485708701308 | 0.699655916086791 | 3293.27071211801 | 3281.53622244524 | 3588.78100632144 | 3523.63110413772 | 2765.48819639512 | 3404.95895277983 |
| YCR059C | 3075.75307039671 | 1.38961432579486 | 0.241431013849099 | 5.75574075443103 | 8.62626833660348e-09 | 2.75208934732957e-08 | 1472.20525487482 | 1337.25681355215 | 2288.11442156778 | 4235.82603585973 | 4290.93034394228 | 4830.18555258353 |
| YCR060W | 1924.91980853291 | 1.67762525900608 | 0.104147595333087 | 16.1081516442186 | 2.23608453426726e-58 | 2.33923294988023e-56 | 946.254797273696 | 882.195219751236 | 922.010117174161 | 2799.43288704961 | 2975.25432893903 | 3024.37150100975 |
| YCR061W | 467.517816107799 | 1.0115813641983 | 0.40495835981348 | 2.4979885948378 | 0.0124900212173957 | 0.0194315849402803 | 203.691795718354 | 206.174115602942 | 519.825480735353 | 572.156602450826 | 763.434564020326 | 539.824338118991 |
| YCR063W | 107.624235363976 | -0.689296513978448 | 0.310280000338437 | -2.22153059567681 | 0.0263150448050833 | 0.0390570665001762 | 130.727570386406 | 103.497763211039 | 164.697182015161 | 92.0251878067763 | 59.9331807268293 | 94.864528037647 |
| YCR064C | 28.6556513391827 | 1.2317273364702 | 0.516054612491691 | 2.38681586532671 | 0.0169950010163137 | 0.0260220907912679 | 18.2410563329869 | 23.8209137549216 | 9.55831859909418 | 42.67834796836 | 25.6856488829268 | 51.9496224968067 |
| YCR065W | 512.009086279573 | -0.282113566067164 | 0.242411942291432 | -1.16377750782592 | 0.244514189462423 | 0.30196478157907 | 471.987332616036 | 501.06059967249 | 713.197618547796 | 446.788955293769 | 392.419635711382 | 546.600375835966 |
| YCR066W | 154.2097188703 | -2.97533597614501 | 0.303881342362845 | -9.79111107319106 | 1.22937445758779e-22 | 1.30502827036242e-21 | 307.817825619154 | 283.386732601654 | 228.664391101407 | 36.0098560983038 | 45.6633757918699 | 23.7161320094118 |
| YCR067C | 2903.88197992502 | 0.374241794492971 | 0.117565543691183 | 3.18326086660236 | 0.00145626347927615 | 0.00258492745664616 | 2416.17992010689 | 2451.91129511866 | 2718.97401380387 | 3359.58620413434 | 3410.48337945529 | 3066.15706693109 |
| YCR068W | 372.753516688017 | 0.490381471726663 | 0.329619827597884 | 1.48771836724852 | 0.136825181681906 | 0.178238226227925 | 231.813424231709 | 245.601834921433 | 452.18199526484 | 370.768147975128 | 546.533529008943 | 389.62216872605 |
| YCR069W | 3595.3417892296 | 1.16456350577405 | 0.243569118069307 | 4.78124449850277 | 1.7421332178159e-06 | 4.41730885486862e-06 | 1727.58004353663 | 1939.35094397828 | 2987.34218985536 | 4657.27472204729 | 5438.22266071301 | 4822.28017524706 |
| YCR071C | 471.399419783774 | 0.754990888278046 | 0.302565253611562 | 2.49529937514675 | 0.0125850900310104 | 0.0195560359226482 | 311.618045688526 | 256.280175570191 | 484.53322744639 | 637.507822777378 | 455.206777425204 | 683.250469794958 |
| YCR072C | 1345.65246439341 | 0.860990683774142 | 0.22766577326813 | 3.78181872230799 | 0.000155686722921628 | 0.000313987588578881 | 843.648855400644 | 955.300782654271 | 1068.32591726799 | 1999.21386264286 | 1272.86660019837 | 1934.5587681963 |
| YCR073C | 806.464394184614 | -0.606149835038177 | 0.118580806459256 | -5.11170275474935 | 3.19267909794766e-07 | 8.71170241030228e-07 | 921.933388829713 | 969.26476657957 | 1029.35738759476 | 636.174124403366 | 629.298397631708 | 652.758300068571 |
| YCR073W-A | 2152.49145391806 | 1.74085068070742 | 0.313471481336838 | 5.5534579199465 | 2.8007311037067e-08 | 8.46089517402964e-08 | 755.483749791208 | 723.662931658137 | 1494.77397784296 | 2920.79943908464 | 3471.84354067561 | 3548.3850844558 |
| YCR075C | 712.303562961267 | 1.44270677509855 | 0.13582857712481 | 10.6215260855811 | 2.36660059686244e-26 | 3.33690684157604e-25 | 357.220686520993 | 372.920511887394 | 419.095507806437 | 984.269400020303 | 1073.08933110894 | 1067.22594042353 |
| YCR075W-A | 519.165022151123 | 1.75039515361106 | 0.181770868505576 | 9.62967921098625 | 5.99162323045187e-22 | 6.03033475216879e-21 | 234.853600287206 | 199.602829049861 | 278.661749927438 | 774.878755300536 | 859.042257084553 | 767.950941257143 |
| YCR076C | 835.679109014387 | 2.00548237272472 | 0.192482644267354 | 10.419029623986 | 2.03013883649495e-25 | 2.73752193212187e-24 | 291.85690132779 | 298.172127346088 | 409.537189207343 | 1267.01345531069 | 1298.5522490813 | 1448.94273181311 |
| YCR077C | 2817.34355470908 | -0.205877966174448 | 0.266256904091827 | -0.773230526647469 | 0.439385962939715 | 0.504578143701662 | 2429.10066834276 | 2514.33851737294 | 4110.81225288735 | 2900.79396347447 | 2129.05489629594 | 2819.96102988101 |
| YCR079W | 1131.25857484012 | 1.18253338611436 | 0.215490750415185 | 5.48762944040976 | 4.07363495554857e-08 | 1.21590411052407e-07 | 613.355519196685 | 592.2372005965 | 869.80699251757 | 1393.71480084176 | 1778.01769489594 | 1540.41924099227 |
| YCR081C-A | 3.51921894726014 | 1.53859172048092 | 1.07646006835156 | 1.42930682309195 | 0.152916065774334 | 0.197848314903717 | 1.52008802774891 | 1.64282163827046 | 2.20576583056019 | 2.6673967480225 | 8.56188296097562 | 4.51735847798319 |
| YCR081W | 321.60707426813 | -1.58463038624185 | 0.171460313515303 | -9.24196599057555 | 2.42009036342019e-20 | 2.18617076561885e-19 | 465.146936491166 | 486.275204928056 | 496.297311876044 | 180.049280491519 | 136.99012737561 | 164.883584446386 |
| YCR082W | 1583.97098073026 | 0.544830032503301 | 0.136259600157154 | 3.99847080040544 | 6.37530441381441e-05 | 0.00013469128478176 | 1222.910818324 | 1265.79407228739 | 1377.13313354642 | 1916.52456345417 | 1685.2639628187 | 2036.19933395092 |
| YCR083W | 788.206938772256 | 1.9438493479321 | 0.316937415997433 | 6.13322772830282 | 8.61137395458871e-10 | 3.04544010193361e-09 | 261.455140772812 | 223.423742804782 | 490.415269661217 | 1154.98279189374 | 1322.81091747073 | 1276.15377003025 |
| YCR084C | 3897.58415326943 | -0.634323408556294 | 0.150430467098091 | -4.21672165747296 | 2.47879626164337e-05 | 5.51732071139977e-05 | 4949.40661835045 | 5244.70808017844 | 4029.19891715662 | 2967.47888217503 | 3000.93997782195 | 3193.77244393412 |
| YCR085W | 6.28862942355671 | 1.95578059688006 | 1.16193858392194 | 1.68320479579792 | 0.0923354527524861 | 0.124508887017178 | 6.08035211099563 | 1.64282163827046 | 0 | 14.6706821141238 | 8.56188296097562 | 6.77603771697479 |
| YCR086W | 117.591288695374 | -1.26985191797105 | 0.279570496252928 | -4.54215282009663 | 5.5682651203264e-06 | 1.33910891992722e-05 | 202.931751704479 | 167.567807103587 | 127.934418172491 | 73.3534105706188 | 72.7760051682927 | 60.9843394527731 |
| YCR087C-A | 768.32367988853 | -1.27935990554986 | 0.284370261577408 | -4.49892298320234 | 6.82985932567825e-06 | 1.63161943227805e-05 | 1365.79909293239 | 1085.08369207764 | 814.662846753565 | 518.808667490376 | 319.64363054309 | 505.944149534117 |
| YCR087W | 5.76561384027044 | 0.89231084376636 | 0.848821743070606 | 1.05123466858711 | 0.293150824176766 | 0.355264619882381 | 3.80022006937227 | 2.46423245740569 | 5.88204221482719 | 8.0021902440675 | 4.28094148048781 | 10.1640565754622 |
| YCR088W | 518.19815524809 | -1.56336577195099 | 0.298502654647238 | -5.23735969383097 | 1.62890043953637e-07 | 4.595497281789e-07 | 897.61198038573 | 916.694474154916 | 508.796651582552 | 205.389549597733 | 309.654767088618 | 271.041508678991 |
| YCR089W | 793.827869621052 | 0.22595916016162 | 0.192267195104738 | 1.17523512026338 | 0.239900663108653 | 0.296889086228339 | 715.201417055861 | 593.880022234771 | 885.982608608345 | 833.561483757032 | 864.750179058537 | 869.591507011764 |
| YCR090C | 1611.56218013987 | 0.66700920085788 | 0.206187722021806 | 3.23496081297867 | 0.00121659426874706 | 0.00217918542587502 | 1441.04345030597 | 1305.22179160588 | 990.388857921528 | 2293.96120329935 | 1792.2874998309 | 1846.47027787563 |
| YCR091W | 75.130838354823 | -3.21684593058352 | 0.391670297941308 | -8.2131475056747 | 2.15464592610008e-16 | 1.4274804368422e-15 | 159.609242913635 | 142.92548252953 | 104.406249313183 | 20.0054756101688 | 11.4158439479675 | 12.4227358144538 |
| YCR092C | 462.642010166503 | -1.0306412583845 | 0.177203762017576 | -5.8161364445653 | 6.0223315004048e-09 | 1.95499710268396e-08 | 657.438072001403 | 652.200190393372 | 553.647223470609 | 265.405976428239 | 346.756259919512 | 300.404338785882 |
| YCR093W | 1794.35762205423 | -1.28210680939189 | 0.125818589553849 | -10.1901222540979 | 2.19488020251476e-24 | 2.75891337083541e-23 | 2321.17441837258 | 2533.23096621305 | 2774.85341484473 | 1054.9554138429 | 1025.99897482358 | 1055.93254422857 |
| YCR094W | 627.689211053592 | -0.942502389768289 | 0.132558688372579 | -7.11007630913805 | 1.15978811575014e-12 | 5.34260349343425e-12 | 840.608679345146 | 844.410322071015 | 793.340443724817 | 412.112797569476 | 408.116421139838 | 467.54660247126 |
| YCR095C | 372.359096239986 | -1.32427375581698 | 0.25782741705991 | -5.13627980653919 | 2.80230338032589e-07 | 7.69180690850348e-07 | 619.43587130768 | 562.666411107632 | 415.41923142217 | 218.726533337845 | 162.675776258537 | 255.23075400605 |
| YCR095W-A | 14.8951419064009 | 0.434310670315578 | 0.553227496469215 | 0.785048959220966 | 0.432424863786517 | 0.497729843215501 | 9.8805721803679 | 13.9639839252989 | 13.9698502602146 | 16.004380488135 | 24.2586683894309 | 11.293396194958 |
| YCR097W | 400.309861239269 | -0.162941436288507 | 0.352517894442846 | -0.462221744930243 | 0.643922311071672 | 0.698875520349877 | 519.870105490127 | 350.742419770743 | 398.508360054542 | 472.129224399983 | 205.485191063415 | 455.123866656807 |
| YCR098C | 1077.86035909909 | 2.37853857046596 | 0.21124054071147 | 11.259858370249 | 2.07091270492853e-29 | 3.6010562477658e-28 | 283.496417175171 | 326.100095196686 | 433.065358066652 | 1805.82759841123 | 1999.19967138781 | 1619.47301435697 |
| YCR099C | 272.318616052092 | -0.0281189347427175 | 0.2501604136245 | -0.112403614685915 | 0.910503389387905 | 0.928541664083326 | 241.693996412076 | 253.815943112786 | 328.659108753469 | 241.399405696036 | 342.475318439025 | 225.86792389916 |
| YCR100C | 332.173701056464 | -0.0559570085859853 | 0.263983827033279 | -0.211971351483328 | 0.832129384658378 | 0.8632743424335 | 313.138133716275 | 305.564824718305 | 396.302594223982 | 286.745150412419 | 433.802070022764 | 257.489433245042 |
| YCR101C | 161.129352924422 | 0.328537781954518 | 0.333436621272689 | 0.985308034553995 | 0.324472787337727 | 0.388146532399944 | 112.486514053419 | 120.747390412879 | 194.842648366151 | 154.709011385305 | 238.305742413821 | 145.684810914958 |
| YCR102C | 289.98330734079 | 1.4755183682018 | 0.449364448466962 | 3.28356720972395 | 0.00102502200356183 | 0.00185189212119833 | 91.2052816649345 | 94.4622442005513 | 274.250218266318 | 392.107321959308 | 493.735250749594 | 394.139527204033 |
| YCR102W-A | 14.2472690812542 | 1.06719820515838 | 0.520449416960271 | 2.05053204092616 | 0.0403125404155028 | 0.0579107723443968 | 9.12052816649345 | 7.39269737221706 | 11.028829152801 | 17.3380788621463 | 21.404707402439 | 19.1987735314286 |
| YCR104W | 27.8434927654203 | 1.62238661648762 | 0.597514730343728 | 2.7152244691178 | 0.00662308625176245 | 0.0107554675585707 | 9.12052816649345 | 8.21410819135229 | 23.5281688593087 | 24.0065707322025 | 51.3712977658537 | 50.8202828773109 |
| YCR105W | 103.351873414929 | 0.651161224338838 | 0.253126224075779 | 2.57247634738903 | 0.0100973855234679 | 0.0159037500012659 | 75.244357373571 | 95.2836550196866 | 70.5845065779262 | 138.70463089717 | 134.136166388618 | 106.157924232605 |
| YCR106W | 299.946987583084 | -0.182155799647271 | 0.173095027846949 | -1.05234565032298 | 0.292640994484508 | 0.35471304246431 | 284.256461189046 | 313.778932909657 | 358.069319827605 | 294.747340656486 | 286.823079192683 | 262.006791723025 |
| YCR107W | 449.598618754214 | 0.570698923438824 | 0.16976676975799 | 3.3616645015534 | 0.00077474193501879 | 0.00142028722174445 | 322.258661882769 | 341.706900760255 | 421.301273636997 | 560.153317084725 | 517.993919139025 | 534.177640021512 |
| YCR108C | 1.01419824170719 | -1.74553186650238 | 2.0745135467482 | -0.841417434578098 | 0.400114121702029 | 0.465168655824141 | 2.28013204162336 | 1.64282163827046 | 0.735255276853398 | 0 | 1.42698049349594 | 0 |
| YDL001W | 280.468977725669 | -0.981947425085623 | 0.17358443075042 | -5.65688651246302 | 1.54143612699515e-08 | 4.78819670483263e-08 | 418.02420763095 | 355.670884685554 | 343.364214290537 | 198.721057727676 | 184.080483660976 | 182.953018358319 |
| YDL002C | 251.957222169954 | -3.24350633047041 | 0.375517458122677 | -8.63743152365182 | 5.7490895905073e-18 | 4.37659566713971e-17 | 563.952658294845 | 542.952551448386 | 261.015623282956 | 44.0120463423713 | 39.9554538178862 | 59.8549998332773 |
| YDL003W | 263.601492380935 | -2.72324843455783 | 0.544734654040015 | -4.9992201053502 | 5.7562663997509e-07 | 1.53327079543262e-06 | 692.400096639628 | 549.523838001468 | 131.610694556758 | 80.021902440675 | 67.068083194309 | 60.9843394527731 |
| YDL004W | 7652.4983034812 | 1.22379065705525 | 0.122202657112547 | 10.0144357411816 | 1.31711336401238e-23 | 1.52278026363357e-22 | 4887.84305322661 | 4770.75403753741 | 4107.13597650308 | 10490.8714099725 | 10546.8128274285 | 11111.5725162192 |
| YDL005C | 137.224703577708 | -1.04542883283288 | 0.519328906472872 | -2.01303801849415 | 0.0441106298971702 | 0.0629347878383296 | 279.696197105799 | 211.923991336889 | 63.2319538093923 | 90.691489432765 | 72.7760051682927 | 105.028584613109 |
| YDL006W | 800.251288806965 | -0.302438420942075 | 0.235637836270633 | -1.28348836387515 | 0.199321033654272 | 0.251418946768107 | 972.856337759301 | 1027.58493473817 | 650.700920015258 | 772.211358552514 | 770.569466487805 | 607.584715288739 |
| YDL007C-A | 1.20000470984639 | -0.107902112129652 | 1.98703578586003 | -0.0543030542768756 | 0.95669371618487 | 0.966528885229762 | 1.52008802774891 | 0.821410819135229 | 1.4705105537068 | 0 | 0 | 3.38801885848739 |
| YDL007W | 1567.82630945228 | -0.725824202554138 | 0.229987892321056 | -3.15592353679606 | 0.00159990796358373 | 0.00282521183005829 | 1715.41933931464 | 1616.53649205813 | 2530.7486629294 | 1174.98826750391 | 1044.54972123903 | 1324.71537366857 |
| YDL008W | 1007.35357723449 | 1.10457882780077 | 0.133926567392408 | 8.24764532763931 | 1.61545981931965e-16 | 1.08242483348215e-15 | 665.038512140147 | 680.949569063105 | 572.763860668797 | 1443.06164068017 | 1354.20448832764 | 1328.10339252706 |
| YDL009C | 22.4098163615102 | 2.34088219668786 | 0.551391681991301 | 4.24540716362273 | 2.18196837183124e-05 | 4.89020278496801e-05 | 9.12052816649345 | 5.7498757339466 | 7.35255276853398 | 40.0109512203375 | 21.404707402439 | 50.8202828773109 |
| YDL010W | 340.073545822745 | -2.14742614519985 | 0.271931465358836 | -7.89693882010362 | 2.8583658335204e-15 | 1.67322750868351e-14 | 592.834330822074 | 535.559854076169 | 537.471607379834 | 141.372027645193 | 78.4839271422765 | 154.719527870924 |
| YDL011C | 1.27230690768165 | 1.27229004882704 | 2.50586504392856 | 0.507724888022069 | 0.611646291984573 | 0.669545787944915 | 0 | 0 | 2.20576583056019 | 4.00109512203375 | 1.42698049349594 | 0 |
| YDL012C | 1398.35344419253 | 0.739548343283825 | 0.217816028166146 | 3.39528890279697 | 0.000685562179501533 | 0.00126646433957475 | 940.1744451627 | 851.803019443233 | 1350.66394357969 | 1697.79803011632 | 1673.84811887073 | 1875.83310798252 |
| YDL013W | 499.366134745645 | -1.42436748215355 | 0.148596599057804 | -9.58546488402115 | 9.20421957842171e-22 | 9.1003914917139e-21 | 689.35992058413 | 774.590402444521 | 719.814916039477 | 249.401595940104 | 268.272332777236 | 294.757640688403 |
| YDL014W | 34568.7527601288 | 0.40555142100375 | 0.17192887708717 | 2.35883248861173 | 0.0183325289314481 | 0.0279381538179916 | 32418.917367801 | 32996.0726046621 | 23810.5068856205 | 40863.1844813307 | 38686.8681591683 | 38636.9670621902 |
| YDL015C | 7426.60487290666 | 0.984210163968407 | 0.0990635025326776 | 9.93514401172875 | 2.92752624747252e-23 | 3.30800265524508e-22 | 4830.83975218603 | 5167.49546317973 | 4962.97311876044 | 10033.4128676866 | 10234.3040993529 | 9330.60393627428 |
| YDL016C | 1577.37287631174 | 1.4444640594556 | 0.112746773111673 | 12.8115778358011 | 1.41233183099454e-37 | 4.62645669486394e-36 | 806.406698720796 | 828.803516507446 | 907.305011637094 | 2327.30366264963 | 2367.36063870976 | 2227.05772964571 |
| YDL017W | 445.414656805771 | -0.944234133909751 | 0.277492813959153 | -3.40273364357731 | 0.000667152854547731 | 0.00123386182338083 | 696.960360722874 | 656.307244489048 | 405.125657546222 | 252.068992688126 | 363.880025841464 | 298.145659546891 |
| YDL018C | 627.837151756208 | 0.650484370740603 | 0.116375187914628 | 5.58954518052243 | 2.27665127240002e-08 | 6.9456068451489e-08 | 481.867904796404 | 495.310723938543 | 488.94475910751 | 798.885326032739 | 736.321934643903 | 765.692262018151 |
| YDL019C | 577.112949764796 | -1.54483850608312 | 0.248295958681329 | -6.22176258642137 | 4.91600541541831e-10 | 1.79029821024162e-09 | 1021.49915464727 | 955.300782654271 | 602.174071742933 | 273.408166672306 | 305.37382560813 | 304.921697263865 |
| YDL020C | 482.381851666898 | -2.18483437034793 | 0.217016424846143 | -10.0675991317104 | 7.68306530292398e-24 | 9.14355257885595e-23 | 843.648855400644 | 818.125175858688 | 710.991852717236 | 217.392834963834 | 136.99012737561 | 167.142263685378 |
| YDL021W | 51.7618550617662 | -0.49861627652678 | 0.361572545040735 | -1.37902139796207 | 0.167888156541802 | 0.215670941439914 | 75.244357373571 | 40.2491301376262 | 66.1729749168059 | 44.0120463423713 | 44.236395298374 | 40.6562263018487 |
| YDL022W | 3604.92208945384 | 1.25884224697697 | 0.329121562659001 | 3.82485497700811 | 0.000130849087691619 | 0.000265712956408216 | 1750.38136395287 | 1708.53450380128 | 2915.28717272372 | 3919.73952121906 | 7197.6896091935 | 4137.9003658326 |
| YDL023C | 4.42636887881826 | -0.908831832136214 | 1.43131738091913 | -0.634961780141734 | 0.525453342296306 | 0.588210282729348 | 10.6406161942424 | 0 | 6.61729749168058 | 1.33369837401125 | 5.70792197398374 | 2.2586792389916 |
| YDL024C | 88.0808045178746 | -0.405224008873441 | 0.319400275549499 | -1.2687027529212 | 0.204547100245912 | 0.257460215834462 | 85.1249295539389 | 105.140584849309 | 110.28829152801 | 61.3501252045175 | 105.596556518699 | 60.9843394527731 |
| YDL025C | 270.885676547177 | -1.79406577982121 | 0.17869027004405 | -10.040086566431 | 1.01584968174749e-23 | 1.19146492510202e-22 | 418.02420763095 | 395.098604004045 | 449.240974157426 | 114.698060164968 | 112.731458986179 | 135.520754339496 |
| YDL025W-A | 3.9667287652336 | 1.74395202895355 | 1.02409098523557 | 1.70292684350931 | 0.08858175979555 | 0.119770959773595 | 2.28013204162336 | 2.46423245740569 | 0.735255276853398 | 6.66849187005625 | 7.13490246747968 | 4.51735847798319 |
| YDL026W | 22.869580613485 | 0.801995041860408 | 0.498423440166189 | 1.60906365397462 | 0.107602423684433 | 0.143195640502235 | 9.8805721803679 | 18.071038020975 | 22.057658305602 | 21.33917398418 | 34.2475318439025 | 31.6215093458823 |
| YDL027C | 622.40681368711 | 0.0887735742560397 | 0.172880433446451 | 0.513496944022512 | 0.607603747929983 | 0.665808060326722 | 580.673626600083 | 520.774459331735 | 708.050831609823 | 669.516583753648 | 620.736514670732 | 634.688866156638 |
| YDL028C | 379.878955061519 | -0.906533054377226 | 0.259636448166085 | -3.49154774216189 | 0.000480230632127113 | 0.000908627736282512 | 565.472746322594 | 581.558859947742 | 339.68793790627 | 266.73967480225 | 246.867625374797 | 278.946886015462 |
| YDL029W | 4736.42905093487 | 1.27905062677327 | 0.206827913480529 | 6.18412962374965 | 6.24460413807615e-10 | 2.24639503269894e-09 | 2505.1050697302 | 2275.30796900458 | 3512.31445752868 | 7129.95150746414 | 6240.18569805773 | 6755.70960382386 |
| YDL030W | 358.586714875675 | -1.15577672862618 | 0.186035801203241 | -6.21265756994546 | 5.20958964525153e-10 | 1.88873104746235e-09 | 551.791954072854 | 521.59587015087 | 411.742955037903 | 222.727628459879 | 221.18197649187 | 222.479905040672 |
| YDL031W | 791.117706661823 | -3.46966404180078 | 0.594962524043421 | -5.83173544817684 | 5.48538401694449e-09 | 1.79145018801118e-08 | 2114.44244659873 | 1933.60106824433 | 305.866195171014 | 142.705726019204 | 99.8886345447155 | 150.202169392941 |
| YDL032W | 3.51037094831629 | 0.368196717455846 | 1.04069647535561 | 0.353798370778599 | 0.723489994558592 | 0.770534664155506 | 3.04017605549782 | 1.64282163827046 | 4.41153166112039 | 4.00109512203375 | 5.70792197398374 | 2.2586792389916 |
| YDL033C | 396.136524861697 | 0.819031319326549 | 0.258667321205934 | 3.16635018102843 | 0.00154364840167207 | 0.00273256100798173 | 249.294436550821 | 226.709386081323 | 383.803254517474 | 520.142365864388 | 442.36395298374 | 554.505753172437 |
| YDL034W | 1.01178143993042 | -0.674020750906256 | 2.05487851466756 | -0.328010023996626 | 0.742904083505093 | 0.787426420705786 | 1.52008802774891 | 0.821410819135229 | 1.4705105537068 | 0 | 0 | 2.2586792389916 |
| YDL035C | 589.149912613039 | -0.75081760044959 | 0.15645584663705 | -4.79891046955474 | 1.59531082491465e-06 | 4.05931189109313e-06 | 814.00713885954 | 745.841023774788 | 657.318217506938 | 408.111702447443 | 468.049601866667 | 441.571791222857 |
| YDL036C | 393.234717661961 | -2.25299969053588 | 0.252613920229405 | -8.91874718736749 | 4.71592543300583e-19 | 3.91645228661662e-18 | 757.003837818956 | 725.305753296407 | 468.357611355615 | 140.038329271181 | 124.147302934146 | 144.555471295462 |
| YDL037C | 1381.26916045207 | -1.9776103870857 | 0.347354360653507 | -5.69335126055436 | 1.24569738240574e-08 | 3.91263594299449e-08 | 2649.51343236635 | 2741.86931427339 | 1217.58273846923 | 498.803191880208 | 707.782324773984 | 472.063960949243 |
| YDL039C | 4811.36982151616 | -1.72278921743519 | 0.320747239453436 | -5.37117395108648 | 7.82257038060131e-08 | 2.28032321296989e-07 | 8749.62668772272 | 8633.84911993039 | 4771.80674677855 | 1736.47528296265 | 3029.47958769187 | 1946.98150401076 |
| YDL040C | 2709.8515611983 | -0.868137152166394 | 0.100200336013848 | -8.66401438061456 | 4.55435897876585e-18 | 3.49167521705382e-17 | 3357.11440928346 | 3477.85340821856 | 3669.65908677531 | 1869.84512036377 | 1920.71574424553 | 1963.92159830319 |
| YDL041W | 9.02895862922516 | 0.806168602761356 | 0.776980535980241 | 1.0375660205494 | 0.299472144258073 | 0.362113409332189 | 8.360484152619 | 4.10705409567614 | 7.35255276853398 | 12.0032853661013 | 4.28094148048781 | 18.0694339119328 |
| YDL042C | 1008.39165845813 | 0.102157678753526 | 0.176276929069568 | 0.579529489722442 | 0.562231954341292 | 0.62356984539289 | 909.012640593847 | 841.94608961361 | 1166.85012436634 | 1088.29787319318 | 1013.15615038211 | 1031.08707259966 |
| YDL043C | 363.606995348417 | -2.24470549322245 | 0.258673553683304 | -8.67775410844921 | 4.03660589511583e-18 | 3.10574446449837e-17 | 702.280668819996 | 640.700438925479 | 459.534548033374 | 121.366552035024 | 104.169576025203 | 153.590188251429 |
| YDL044C | 102.622819124983 | -1.56610075414977 | 0.467029042765561 | -3.35332626184433 | 0.00079846524704515 | 0.00146171199332059 | 204.451839732228 | 183.996023486291 | 72.055017131633 | 65.3512203265513 | 35.6745123373984 | 54.2083017357983 |
| YDL045C | 806.15355174828 | 0.214139353482905 | 0.166949601757954 | 1.28265866601686 | 0.199611687042607 | 0.251736613291533 | 680.999436431511 | 678.485336605699 | 879.365311116664 | 837.562578879065 | 908.986574356911 | 851.522073099832 |
| YDL045W-A | 522.595029039646 | 0.406970237101187 | 0.197734296400202 | 2.05816716932861 | 0.0395740889683997 | 0.056938230046371 | 519.110061476252 | 358.956527962095 | 469.828121909322 | 648.177409769468 | 552.241450982927 | 587.256602137815 |
| YDL046W | 6476.01867583435 | 1.13561711426478 | 0.125524060080202 | 9.04700751026691 | 1.469399286511e-19 | 1.27073650297471e-18 | 3769.05826480342 | 3904.16562334974 | 4479.91040186776 | 8428.9737237511 | 9253.96850032115 | 9020.03554091294 |
| YDL047W | 2984.56243695124 | 0.528715587246839 | 0.131183861675018 | 4.03034016910273 | 5.56961905734038e-05 | 0.000119065752161865 | 2369.05719124667 | 2305.70016931259 | 2656.47731527133 | 3214.21308136711 | 3610.26064854472 | 3751.66621596504 |
| YDL048C | 4650.6520683085 | -2.66893996567796 | 0.28542370166449 | -9.35080005659533 | 8.69872109745107e-21 | 8.07151717282798e-20 | 10059.9425676423 | 8824.41642996977 | 5227.66501842766 | 1073.62719107906 | 1521.16120606667 | 1197.09999666555 |
| YDL049C | 277.211217201182 | 1.40161963876115 | 0.251508739886478 | 5.57284665095056 | 2.50610155988827e-08 | 7.60981962426747e-08 | 122.367086233787 | 149.496769082612 | 184.549074490203 | 333.424593502813 | 489.454309269106 | 383.975470628571 |
| YDL050C | 5.75465546584611 | 0.993264754455034 | 1.01996191649531 | 0.973825334447766 | 0.330143271728444 | 0.393840217110666 | 6.84039612487009 | 1.64282163827046 | 2.94102110741359 | 8.0021902440675 | 12.8428244414634 | 2.2586792389916 |
| YDL051W | 2793.3557357075 | -3.11776286819183 | 0.289289750958863 | -10.7773015043149 | 4.40622555777725e-27 | 6.55476581828973e-26 | 5869.05987513854 | 5493.59555837641 | 3667.45332094475 | 565.48811058077 | 412.397362620325 | 752.140186584201 |
| YDL052C | 2462.00905291603 | 1.10879259655795 | 0.141873296407826 | 7.81537205825281 | 5.4800915811685e-15 | 3.1097002620699e-14 | 1454.72424255571 | 1470.32536625206 | 1754.31909057221 | 3100.84871957616 | 3536.05766288293 | 3455.77923565714 |
| YDL053C | 221.621851254824 | -4.39311417860731 | 0.356564352910663 | -12.3206768785101 | 7.01033928748549e-35 | 1.82606669151128e-33 | 495.548697046144 | 486.275204928056 | 287.484813249679 | 22.6728723581913 | 18.5507464154472 | 19.1987735314286 |
| YDL054C | 1321.94521187371 | 0.538085371188722 | 0.121025151079464 | 4.44606237950839 | 8.74585620259811e-06 | 2.06500266945946e-05 | 1057.98126731324 | 1133.54693040662 | 1042.59198257812 | 1588.4347634474 | 1662.43227492277 | 1446.68405257412 |
| YDL055C | 81586.0549739748 | 1.36589654505052 | 0.179368707918488 | 7.61502137636646 | 2.63647831776214e-14 | 1.41440846724609e-13 | 38673.3195579739 | 42594.2580262573 | 55569.1233140261 | 116375.852719474 | 123679.25333228 | 112624.522893838 |
| YDL056W | 32.9936913602548 | -2.43437528207118 | 0.559661639202835 | -4.34972689130282 | 1.36307186306028e-05 | 3.13729031362986e-05 | 67.6439172348264 | 71.4627412647649 | 27.9397005204291 | 9.33588861807875 | 11.4158439479675 | 10.1640565754622 |
| YDL057W | 342.049953684143 | 0.635978724407 | 0.259212402071633 | 2.45350422790052 | 0.0141471876353568 | 0.0218525027394437 | 224.212984092964 | 221.780921166512 | 357.334064550752 | 360.098560983038 | 455.206777425204 | 433.666413886386 |
| YDL058W | 319.591121249073 | -0.00907237591843664 | 0.173983327226027 | -0.0521450880557678 | 0.958413086738817 | 0.967813682171566 | 304.777649563656 | 368.813457791718 | 288.220068526532 | 312.085419518633 | 335.340415971545 | 308.309716122353 |
| YDL059C | 139.69213453152 | -0.65912823328303 | 0.32241212596892 | -2.0443655191386 | 0.0409174510161915 | 0.0587017445899177 | 147.448538691644 | 149.496769082612 | 216.900306671753 | 102.694774798866 | 77.0569466487805 | 144.555471295462 |
| YDL060W | 2479.85703286849 | -0.819952203521255 | 0.206682486860879 | -3.96720697517627 | 7.2719836515506e-05 | 0.000152394461919086 | 3490.88215572537 | 3587.92245798268 | 2420.46037140139 | 1861.84293011971 | 1592.51023074146 | 1925.52405124034 |
| YDL061C | 32215.1956441142 | 0.914196691834076 | 0.345436463014101 | 2.64649737279402 | 0.00813301369378029 | 0.0130345260236864 | 28557.1337333049 | 27048.236863304 | 11404.5445992731 | 42584.9890821792 | 46438.2261998382 | 37258.0433867859 |
| YDL062W | 4.82132277999904 | 0.431766754178925 | 0.915032728230755 | 0.471859356346477 | 0.637027180260563 | 0.692550836602415 | 6.84039612487009 | 3.28564327654092 | 2.20576583056019 | 6.66849187005625 | 4.28094148048781 | 5.64669809747899 |
| YDL063C | 1987.69191378716 | -0.641691214343727 | 0.187855604433183 | -3.41587474209195 | 0.000635774841256382 | 0.0011781816058254 | 2548.42757852104 | 2686.83478939133 | 2032.98084049965 | 1776.48623418299 | 1334.2267614187 | 1547.19527870924 |
| YDL064W | 2231.05858406323 | 1.4109114304795 | 0.250758630427688 | 5.6265717677317 | 1.83826325493168e-08 | 5.66143184780953e-08 | 998.697834231033 | 981.585928866599 | 1677.85254177945 | 3056.83667323379 | 3272.06627158618 | 3399.31225468235 |
| YDL065C | 62.8496652681466 | -1.94446588022114 | 0.62292364351454 | -3.1215156150607 | 0.00179922705911053 | 0.00315826433163488 | 155.048978830389 | 104.319174030174 | 39.7037849500835 | 41.3446495943488 | 24.2586683894309 | 12.4227358144538 |
| YDL066W | 4363.4074999963 | 1.14141857093064 | 0.22144393952411 | 5.1544358061168 | 2.5439568699858e-07 | 7.0213209611608e-07 | 2218.56847649953 | 2367.30598074773 | 3579.9579429992 | 5800.25422857493 | 6131.73518055204 | 6082.62319060437 |
| YDL067C | 2530.32306492994 | 1.45987200072134 | 0.170397354246551 | 8.56745697241885 | 1.05795372140716e-17 | 7.84215752805352e-17 | 1559.61031647038 | 1389.00569515767 | 1099.20663889583 | 3694.34449601116 | 3734.40795147886 | 3705.36329156571 |
| YDL068W | 13.0863905800986 | 1.87964486196387 | 0.618257053345187 | 3.04023197437655 | 0.0023639599449691 | 0.00407674666393768 | 4.56026408324673 | 3.28564327654092 | 8.82306332224078 | 22.6728723581913 | 19.9777269089431 | 19.1987735314286 |
| YDL069C | 151.006419988189 | -1.26597567142437 | 0.271409609186737 | -4.6644467571277 | 3.09448437420772e-06 | 7.65769769214471e-06 | 271.33571295318 | 205.352704783807 | 163.226671461454 | 90.691489432765 | 88.472790596748 | 86.9591507011764 |
| YDL070W | 848.188071876054 | -2.64300972195769 | 0.191638356978139 | -13.791653005349 | 2.86127633887047e-43 | 1.32558845242242e-41 | 1582.41163688661 | 1274.82959129788 | 1530.80148640878 | 246.734199192081 | 191.215386128455 | 263.136131342521 |
| YDL071C | 36.350020038653 | 0.976739957538647 | 0.385587963470471 | 2.53311838042747 | 0.011305276558914 | 0.0177030477453202 | 19.0011003468614 | 27.1065570314626 | 27.2044452435757 | 41.3446495943488 | 62.7871417138212 | 40.6562263018487 |
| YDL072C | 7175.93500999897 | 0.935764544035906 | 0.199353820360032 | 4.6939885192364 | 2.67929076439904e-06 | 6.68123025678285e-06 | 4726.71372228523 | 4807.7175243985 | 5246.046400349 | 7694.1059196709 | 11786.8588762764 | 8794.16761701378 |
| YDL073W | 540.94996612853 | -1.19517268513702 | 0.185377323648925 | -6.44724317738283 | 1.13902954013336e-10 | 4.35652132088587e-10 | 795.766082526554 | 846.874554528421 | 616.879177280001 | 337.425688624846 | 311.081747582114 | 337.672546229244 |
| YDL074C | 573.636432562856 | -0.835314648534144 | 0.424598851583296 | -1.96730312722072 | 0.0491482747488887 | 0.069480320405687 | 1007.05831838365 | 892.873560399994 | 305.866195171014 | 474.796621148005 | 346.756259919512 | 414.467640354958 |
| YDL075W | 33548.1351853972 | -1.02118553383032 | 0.337652020623651 | -3.02437264241502 | 0.00249149346788673 | 0.0042852894809635 | 58323.497492684 | 52902.9638064044 | 23621.5462794691 | 24521.3783045709 | 19586.7342537252 | 22332.6909755294 |
| YDL076C | 664.347901954004 | -0.465332580522033 | 0.189527685421458 | -2.45522219873714 | 0.0140797529839972 | 0.0217535202130076 | 666.558600167896 | 708.056126094568 | 936.71522271123 | 538.814143100545 | 589.342943813822 | 546.600375835966 |
| YDL077C | 582.559371956121 | -1.55174139348438 | 0.127904140876474 | -12.1320653330764 | 7.14249990053135e-34 | 1.72859158040471e-32 | 835.288371248025 | 883.838041389506 | 886.717863885198 | 290.746245534453 | 309.654767088618 | 289.110942590924 |
| YDL078C | 6151.19671297941 | 1.51980864438346 | 0.197261478892811 | 7.70453842744077 | 1.31317060347272e-14 | 7.21798689332547e-14 | 2770.36043057239 | 2745.15495754994 | 4026.99315132606 | 9102.49140262678 | 8888.66149398618 | 9373.51884181512 |
| YDL079C | 94.6628726716609 | -0.33212093547281 | 0.231160131518618 | -1.43675699304601 | 0.150787054452192 | 0.19532750852345 | 118.566866164415 | 96.926476657957 | 100.729972928916 | 85.35669593672 | 88.472790596748 | 77.92443374521 |
| YDL080C | 955.301299903751 | -0.192921180522973 | 0.137946953456197 | -1.39851715235039 | 0.161957825581514 | 0.208755655151371 | 965.255897620557 | 975.014642313517 | 1117.58802081717 | 861.569149611268 | 849.053393630082 | 963.326695429916 |
| YDL081C | 99441.6176902147 | 0.888908371922159 | 0.303070676486634 | 2.93300685578323 | 0.00335696479128054 | 0.0056789967752336 | 86343.2801081796 | 83019.9914899976 | 39857.4533029459 | 126289.232733499 | 136639.09017421 | 124500.658332456 |
| YDL082W | 22130.0701283406 | -0.521535141016801 | 0.145341721022228 | -3.58833745292611 | 0.000332793374364586 | 0.000644905236369497 | 28666.5800713028 | 26855.2053208072 | 22739.9752025219 | 19345.2949150332 | 16801.2683304211 | 18372.0969299576 |
| YDL083C | 78924.2476288958 | 1.10951753958562 | 0.205700438247431 | 5.39385112175118 | 6.8963365001526e-08 | 2.02030887714498e-07 | 56472.7903188997 | 56359.4605333255 | 37131.8619916503 | 102167.963941132 | 108015.288455175 | 113398.120533193 |
| YDL084W | 14760.848115396 | 0.53720193140353 | 0.152466670208388 | 3.5234056772493 | 0.000426038709180226 | 0.000811299785009673 | 10946.1538878199 | 11627.8915556783 | 13557.3720498998 | 19423.9831190999 | 17050.9899167829 | 15958.6981630951 |
| YDL085C-A | 241.775581896105 | -0.456667029934551 | 0.246558313227214 | -1.85216642650257 | 0.0640019282169783 | 0.0887145266175799 | 329.859102021513 | 252.173121474515 | 257.339346898689 | 250.735294314115 | 158.394834778049 | 202.151791889748 |
| YDL085W | 105.713891112502 | 0.845084065916776 | 0.288235711262144 | 2.93192006714321 | 0.00336873439756263 | 0.00569593621026882 | 60.8035211099563 | 73.1055629030354 | 92.6421648835282 | 112.030663416945 | 166.956717739024 | 128.744716622521 |
| YDL086C-A | 73.5534078680825 | 2.17636569299334 | 0.269276876963297 | 8.08225985660839 | 6.35774498804222e-16 | 4.0269857414494e-15 | 25.8414964717314 | 25.4637353931921 | 28.6749557972825 | 126.701345531069 | 107.023537012195 | 127.615377003025 |
| YDL086W | 6769.01384559029 | 1.611387156174 | 0.234147903214487 | 6.88192007723392 | 5.90511432923145e-12 | 2.55337143595968e-11 | 2809.12267527998 | 2752.54765492215 | 4452.70595662418 | 9262.53520750813 | 10528.262081013 | 10808.9094981943 |
| YDL087C | 136.96274200141 | -1.62384510864509 | 0.236909932661721 | -6.85427196066003 | 7.16766083148646e-12 | 3.07877140086233e-11 | 229.533292190085 | 194.674364135049 | 196.313158919857 | 53.34793496045 | 75.6299661552846 | 72.2777356477311 |
| YDL088C | 1010.79143111704 | -0.707406282996776 | 0.127593237100645 | -5.54423023564152 | 2.95250136056891e-08 | 8.89866348729088e-08 | 1301.19535175307 | 1316.72154307377 | 1144.05721078389 | 776.212453674548 | 734.894954150407 | 791.667073266554 |
| YDL089W | 327.553390153823 | -1.25218011112602 | 0.162854945929375 | -7.68892896669563 | 1.48371804815235e-14 | 8.10732540885943e-14 | 462.106760435668 | 418.098106939832 | 503.649864644578 | 193.386264231631 | 198.350288595935 | 189.729056075294 |
| YDL090C | 985.955604754219 | 0.766858077621924 | 0.115122138801102 | 6.66125634572194 | 2.71496611748209e-11 | 1.10472209774083e-10 | 744.843133596965 | 709.698947732838 | 734.520021576545 | 1206.99702848018 | 1327.09185895122 | 1192.58263818756 |
| YDL091C | 205.321515211731 | -3.06429564104166 | 0.252300094276793 | -12.1454399366172 | 6.06554560718548e-34 | 1.47345051716124e-32 | 426.384691783569 | 360.599349600366 | 313.954003216401 | 41.3446495943488 | 39.9554538178862 | 49.6909432578151 |
| YDL092W | 823.801793263735 | -1.48859189954797 | 0.252032484730906 | -5.90634933880579 | 3.49771873030769e-09 | 1.16041962581973e-08 | 1398.480985529 | 1254.29432081949 | 992.594623752088 | 442.787860171735 | 318.216650049594 | 536.436319260504 |
| YDL093W | 1150.42974529754 | 0.283155060887327 | 0.131085869013891 | 2.16007311098744 | 0.0307670102217818 | 0.0451481511987504 | 1060.26139935486 | 1095.7620327264 | 957.302370463125 | 1344.36796100334 | 1277.14754167886 | 1167.73716655865 |
| YDL094C | 18.8353769202515 | 0.850971208544183 | 0.52781579614011 | 1.61225036228035 | 0.106907470066226 | 0.142382310236046 | 12.1607042219913 | 13.9639839252989 | 13.9698502602146 | 20.0054756101688 | 37.1014928308943 | 15.8107546729412 |
| YDL095W | 7906.071829584 | 0.49266207939471 | 0.106208477342738 | 4.63863235516384 | 3.50722301108957e-06 | 8.60901032052936e-06 | 6284.80395072786 | 6437.39658956279 | 6985.66038538414 | 9271.87109612621 | 8808.75058635041 | 9647.9483693526 |
| YDL096C | 1.2304140568744 | 0.437854400271313 | 2.55021067074876 | 0.171693423329123 | 0.863678556458892 | 0.889883894708876 | 2.28013204162336 | 0.821410819135229 | 0 | 0 | 4.28094148048781 | 0 |
| YDL097C | 1822.18208532339 | -1.37701885416933 | 0.212457325605822 | -6.48139032270486 | 9.08812209709942e-11 | 3.52124013869694e-10 | 2315.09406626159 | 2236.70166050523 | 3342.47048857555 | 1026.94774798866 | 917.548457317887 | 1094.33009129143 |
| YDL098C | 112.653995439757 | -3.24186710674227 | 0.450765206983648 | -7.19191955482907 | 6.38866707328749e-13 | 3.01359233726128e-12 | 250.81452457857 | 214.388223794295 | 147.05105537068 | 18.6717772361575 | 9.98886345447155 | 35.0095282043697 |
| YDL099W | 182.904118665758 | 0.897909354127517 | 0.202069718371599 | 4.44356215945377 | 8.84815774857443e-06 | 2.08687822390014e-05 | 112.486514053419 | 151.961001540017 | 119.111354850251 | 230.729818703946 | 242.586683894309 | 240.549338952605 |
| YDL100C | 4327.5147903871 | -0.201658285322282 | 0.244304551267392 | -0.825438102876627 | 0.409122910138726 | 0.474190706783377 | 4079.1562224642 | 4116.08961468663 | 5693.81686395272 | 4265.16740008798 | 3006.64789979594 | 4804.21074133512 |
| YDL101C | 351.063640237857 | -1.7657686211774 | 0.211259255750631 | -8.35830181689983 | 6.3627493381281e-17 | 4.42323603505883e-16 | 602.714903002442 | 595.522843873041 | 429.389081682385 | 157.376408133328 | 165.529737245529 | 155.84886749042 |
| YDL102W | 882.602484900022 | -0.206918200340464 | 0.135425228384502 | -1.52791472319307 | 0.126533712174824 | 0.165831007711843 | 938.654357134951 | 892.052149580859 | 1005.82921873545 | 889.576815465504 | 816.232842279675 | 753.269526203697 |
| YDL103C | 895.251243542133 | -1.48089748568411 | 0.163022767660693 | -9.08399180638603 | 1.04664163452377e-19 | 9.1121042168069e-19 | 1277.63398732296 | 1276.47241293615 | 1402.13181295943 | 466.794430903938 | 405.262460152846 | 543.212356977479 |
| YDL104C | 375.74437879226 | 0.987892245158128 | 0.14478722617159 | 6.82306216701299 | 8.91200653943708e-12 | 3.77799179181627e-11 | 234.853600287206 | 251.35171065538 | 269.103431328344 | 514.807572368343 | 512.285997165041 | 472.063960949243 |
| YDL105W | 83.826893902932 | -1.74259322690068 | 0.299354595084185 | -5.82116745664327 | 5.84379821678045e-09 | 1.90084630060371e-08 | 152.768846788765 | 110.069049764121 | 124.258141788224 | 30.6750626022588 | 45.6633757918699 | 39.5268866823529 |
| YDL106C | 299.820470651679 | -1.51086888728381 | 0.256957182836344 | -5.87984687023162 | 4.10646198814876e-09 | 1.35544592646987e-08 | 528.99063365662 | 487.918026566326 | 314.689258493254 | 162.711201629373 | 155.540873791057 | 149.072829773445 |
| YDL107W | 92.4820111029263 | -2.17240077436494 | 0.309727908369923 | -7.01390063878371 | 2.31764017643407e-12 | 1.03693165077442e-11 | 185.450739385367 | 125.67585532769 | 143.374778986413 | 36.0098560983038 | 27.1126293764228 | 37.2682074433613 |
| YDL108W | 929.438228748802 | 0.718702003354997 | 0.160492144962606 | 4.47811326543397 | 7.53056452189054e-06 | 1.79044140355506e-05 | 603.474947016317 | 692.449320530998 | 811.721825646152 | 1172.32087075589 | 1145.86533627724 | 1150.79707226622 |
| YDL109C | 122.026448668834 | -2.1470171597423 | 0.302768470919356 | -7.09128382233094 | 1.32873515758613e-12 | 6.08198746090588e-12 | 161.889374955259 | 175.781915294939 | 259.54511272925 | 42.67834796836 | 47.0903562853659 | 45.1735847798319 |
| YDL110C | 331.611932621718 | -0.729960789924175 | 0.213140484387538 | -3.42478713990788 | 0.000615281279382801 | 0.00114379890457921 | 401.303239325712 | 393.455782365775 | 446.299953050013 | 192.05256585762 | 296.811942647155 | 259.748112484034 |
| YDL111C | 3127.61696242134 | 0.803938406405694 | 0.10286528831078 | 7.81544891972508 | 5.47674842954308e-15 | 3.1097002620699e-14 | 2208.68790431916 | 2327.87826142924 | 2298.40799544372 | 4035.77127975804 | 3740.11587345285 | 4154.84046012504 |
| YDL112W | 3013.96657053811 | -0.38851944502083 | 0.112181999933223 | -3.46329576270791 | 0.000533601512765418 | 0.00100171907722041 | 3457.44021911489 | 3519.74535999446 | 3274.82700310504 | 2818.10466428577 | 2498.64284411138 | 2515.03933261714 |
| YDL113C | 194.213028642942 | -3.43753270142129 | 0.326503818403034 | -10.5283078104098 | 6.39741594416614e-26 | 8.86616235338922e-25 | 415.744075589326 | 398.384247280586 | 252.192559960716 | 29.3413642282475 | 41.3824343113821 | 28.2334904873949 |
| YDL114W | 8.74713066853592 | -0.440425656648276 | 0.673998099985245 | -0.653452371242468 | 0.513464685342377 | 0.576082329896325 | 8.360484152619 | 10.678340648758 | 11.028829152801 | 9.33588861807875 | 8.56188296097562 | 4.51735847798319 |
| YDL114W-A | 8.1919186054042 | 0.527278046306195 | 0.703608688617898 | 0.749391039132746 | 0.453621550321357 | 0.518264818633843 | 8.360484152619 | 5.7498757339466 | 5.88204221482719 | 10.66958699209 | 12.8428244414634 | 5.64669809747899 |
| YDL115C | 335.972045406232 | -0.893253975589902 | 0.180963736661611 | -4.93609378358618 | 7.97027917883055e-07 | 2.09123101755238e-06 | 468.947156560538 | 454.240182981782 | 386.744275624888 | 212.058041467789 | 266.84535228374 | 226.997263518655 |
| YDL116W | 1642.22332607398 | -0.23739745024162 | 0.166921672227982 | -1.42220867472129 | 0.154965681230878 | 0.200300400251788 | 1821.82550125707 | 2032.99177735969 | 1476.39259592162 | 1565.76189108921 | 1504.03744014472 | 1452.3307506716 |
| YDL117W | 1609.18769221828 | -1.15819344938852 | 0.176542818403937 | -6.56041100883824 | 5.36596703482618e-11 | 2.11572414516004e-10 | 2434.42097643988 | 2433.84025709768 | 1799.90491773712 | 1009.60966912652 | 936.099203733334 | 1041.25112917513 |
| YDL118W | 1.58186290158472 | 1.03643030206712 | 1.45382266423096 | 0.712900085799235 | 0.475907586079469 | 0.539916803167143 | 0.760044013874454 | 1.64282163827046 | 0.735255276853398 | 2.6673967480225 | 1.42698049349594 | 2.2586792389916 |
| YDL119C | 519.616029415087 | 0.0445859552966307 | 0.17607259589856 | 0.253224842111818 | 0.800094470730354 | 0.836058117795565 | 478.827728740906 | 473.132631821892 | 582.322179267891 | 469.46182765196 | 592.196904800813 | 521.754904207059 |
| YDL120W | 1718.58287223987 | 0.989799926623189 | 0.126880796225271 | 7.80102234593363 | 6.14076365839723e-15 | 3.4785146802065e-14 | 1101.30377610408 | 1076.04817306715 | 1275.66790534065 | 2205.93711061461 | 2337.39404834634 | 2315.14621996639 |
| YDL121C | 1389.45187284075 | -0.411198970122574 | 0.302183852130633 | -1.36075758920703 | 0.173590308591733 | 0.222467247881048 | 1931.27183925499 | 1900.74463547892 | 926.421648835282 | 1280.3504390508 | 1184.39380960163 | 1113.52886482286 |
| YDL122W | 667.48042822304 | -3.77005666863513 | 0.341246014073263 | -11.0479141532939 | 2.24367355090336e-28 | 3.64723474966395e-27 | 1488.16617916618 | 1402.96967908297 | 841.132036720288 | 104.028473172878 | 55.6522392463415 | 112.93396194958 |
| YDL123W | 871.437046256314 | 1.14627309342921 | 0.326922440096963 | 3.50625393928061 | 0.000454461414548797 | 0.000863649790437591 | 376.981830881729 | 427.133625950319 | 822.750654798953 | 1037.61733498075 | 1339.93468339268 | 1224.20414753344 |
| YDL124W | 5658.65567540473 | 0.637614678833762 | 0.308502294444048 | 2.0668069259673 | 0.0387523506053766 | 0.0558550546725495 | 3526.60422437747 | 3335.74933650817 | 6421.71958803758 | 5729.56821475233 | 8497.6688387683 | 6440.62384998454 |
| YDL125C | 10616.7220685353 | 1.18091582422391 | 0.167660119347327 | 7.04351057855034 | 1.87455762029244e-12 | 8.46094692081889e-12 | 6932.3614505489 | 6952.42117316058 | 5612.20352822199 | 12854.1849287204 | 16540.1309001114 | 14809.0304304484 |
| YDL126C | 13837.7948872999 | 1.39592206166176 | 0.307016744264647 | 4.54672941374976 | 5.44859741243893e-06 | 1.31227637642328e-05 | 5492.07804425681 | 5885.40851910392 | 11484.6874244501 | 19614.7019865835 | 21488.8992515553 | 19060.9940978501 |
| YDL127W | 255.292646032883 | -1.18495632465162 | 0.199347106169395 | -5.9441862358649 | 2.77833586973885e-09 | 9.30319383124738e-09 | 386.102359048223 | 347.456776494202 | 329.394364030322 | 177.381883743496 | 162.675776258537 | 128.744716622521 |
| YDL128W | 4492.81456673229 | 0.34007787154136 | 0.185158036482251 | 1.83668977054614 | 0.0662557062519636 | 0.0915435806989894 | 4379.3736079446 | 4407.69045547964 | 3110.12982108987 | 4892.00563587327 | 5339.76100666179 | 4827.92687334454 |
| YDL129W | 240.643081781847 | -1.9139157111515 | 0.204595044398493 | -9.35465331908889 | 8.38743231626453e-21 | 7.79382320964066e-20 | 385.342315034348 | 327.742916834956 | 427.918571128678 | 93.3588861807875 | 105.596556518699 | 103.899244993613 |
| YDL130W | 82975.109777198 | 0.979878856891265 | 0.325602212430753 | 3.00943549976541 | 0.00261733646331066 | 0.00448982922534592 | 72705.8103672303 | 64588.3541194222 | 30202.8162625839 | 101670.494447626 | 119538.155940155 | 109145.027526171 |
| YDL130W-A | 236.724237429091 | 0.295601967595738 | 0.348870702645761 | 0.847310953181097 | 0.396821823076537 | 0.462495299995403 | 171.769947135627 | 144.5683041678 | 321.306555984935 | 280.076658542363 | 216.901035011382 | 285.722923732437 |
| YDL131W | 3784.73239767384 | -0.467496763956068 | 0.183618940963257 | -2.54601601285575 | 0.0108960190003731 | 0.0170910711575381 | 4051.79463796472 | 4280.37177851368 | 4846.8027850176 | 3310.23936429592 | 2571.41884927968 | 3647.76697097143 |
| YDL132W | 900.739801450873 | -1.51693950764782 | 0.144475554744591 | -10.4996274998183 | 8.67216594716489e-26 | 1.18916846370637e-24 | 1330.83706829417 | 1357.79208403053 | 1317.57745612129 | 501.47058862823 | 400.981518672358 | 495.780092958655 |
| YDL133C-A | 2555.85795809516 | -0.172332379888802 | 0.474457454338568 | -0.363219880545553 | 0.716440637778136 | 0.763779417591879 | 3857.22337041286 | 3331.64228241249 | 935.979967434376 | 2424.66364395245 | 2953.84962153659 | 1831.78886282218 |
| YDL133W | 691.662795862865 | -0.434635362949192 | 0.129879654138514 | -3.34644687678074 | 0.000818543621470376 | 0.00149720076955918 | 841.368723359021 | 810.732478486471 | 733.049511022838 | 566.821808954781 | 610.747651216261 | 587.256602137815 |
| YDL134C | 2785.82027276421 | 0.246049431426184 | 0.108943423586961 | 2.25850651030607 | 0.0239140991511903 | 0.0357389048605116 | 2428.34062432888 | 2599.765242563 | 2617.5087855981 | 3066.17256185186 | 3170.75065654797 | 2832.38376569546 |
| YDL135C | 1234.66206717989 | -0.752883164707515 | 0.147609688782314 | -5.10049964144172 | 3.3875802275434e-07 | 9.20865270571941e-07 | 1658.41603827406 | 1543.4309291551 | 1448.45289540119 | 952.260639044033 | 804.816998331708 | 1000.59490287328 |
| YDL136W | 2991.99819658421 | -1.32020333946331 | 0.330841353687174 | -3.99044232152316 | 6.5950186443977e-05 | 0.000138925920518232 | 5490.55795622906 | 4815.93163258985 | 2512.36728100806 | 2036.55741711518 | 1282.85546365285 | 1813.71942891025 |
| YDL137W | 10200.4975388622 | 1.7938227145805 | 0.15085650831333 | 11.8909202833644 | 1.31944656424436e-32 | 2.91086068560849e-31 | 4121.71868724116 | 4240.12264837605 | 5337.95330995567 | 16281.7897499293 | 15315.7816366919 | 15905.6192009788 |
| YDL138W | 583.936090257535 | -1.34351041017601 | 0.179353965909177 | -7.49083190530817 | 6.84384232165098e-14 | 3.5482942684435e-13 | 890.011540246986 | 927.372814803674 | 696.286747180168 | 310.751721144621 | 332.486454984553 | 346.70726318521 |
| YDL139C | 21.3935593598043 | -2.29258164812926 | 0.798257505573301 | -2.87198257720451 | 0.00407905455427667 | 0.0068047190944029 | 50.9229489295884 | 47.6418275098433 | 8.08780804538738 | 6.66849187005625 | 7.13490246747968 | 7.90537733647059 |
| YDL140C | 6830.06293965476 | -0.255395057923804 | 0.0994215479577368 | -2.56880991263957 | 0.0102048417692367 | 0.0160651950765216 | 7011.40602799184 | 7465.8029351201 | 7821.64563516645 | 6095.00156923141 | 6372.89488395285 | 6213.62658646588 |
| YDL141W | 1612.97755523107 | 0.591983662566819 | 0.164295562281817 | 3.60316282646384 | 0.000314368460195823 | 0.000611575834682096 | 1162.10729721404 | 1213.22377986273 | 1483.74514869016 | 2047.22700410727 | 2039.15512520569 | 1732.40697630655 |
| YDL142C | 861.934114603895 | 1.38131506187407 | 0.161655673520373 | 8.54479791394386 | 1.28761390138007e-17 | 9.51191772705142e-17 | 421.064383686448 | 463.275701992269 | 549.970947086342 | 1157.65018864177 | 1311.39507352277 | 1268.24839269378 |
| YDL143W | 8677.80886169495 | 0.540199893327794 | 0.1080995802564 | 4.99724320896067 | 5.81557038384508e-07 | 1.54779604060809e-06 | 6809.99436431511 | 6830.85237192856 | 7573.86460686686 | 10420.1853961499 | 10655.2633449342 | 9776.69308597512 |
| YDL144C | 1929.17981566857 | 1.02963434754689 | 0.25304996256279 | 4.06889745060288 | 4.72361303537955e-05 | 0.000101852906075372 | 1033.65985886926 | 1022.65646982336 | 1749.17230363423 | 2447.33651631064 | 2575.69979076016 | 2746.55395461378 |
| YDL145C | 7098.17203640741 | -0.158196465858698 | 0.161597952535455 | -0.978950929616447 | 0.32760422589878 | 0.391387181650302 | 6796.31357206537 | 7106.02499633887 | 8559.10667785041 | 6999.24906681104 | 5900.5643406057 | 7227.77356477311 |
| YDL146W | 670.766954022453 | 0.111559532676687 | 0.240086026485823 | 0.464664829976159 | 0.642171498134701 | 0.697443286522672 | 608.795255113438 | 657.950066127318 | 666.876536106032 | 650.84480651749 | 916.121476824391 | 524.01358344605 |
| YDL147W | 1252.02641048219 | -3.3962381382046 | 0.139787079841098 | -24.2957943042035 | 2.17161267456034e-130 | 3.52126995179958e-127 | 2362.2167951218 | 2372.23444566254 | 2127.09351593688 | 218.726533337845 | 202.631230076423 | 229.255942757647 |
| YDL148C | 187.290934681635 | -3.46189091249736 | 0.351938709502727 | -9.83663012627638 | 7.82893032245412e-23 | 8.47720234915482e-22 | 395.982931228591 | 377.02756598307 | 258.074602175543 | 36.0098560983038 | 17.1237659219512 | 39.5268866823529 |
| YDL149W | 189.276137236023 | -1.00889833775379 | 0.227363859476011 | -4.4373733806196 | 9.10632557273858e-06 | 2.143092440667e-05 | 231.813424231709 | 234.923494272676 | 291.896344910799 | 101.361076424855 | 141.271068856098 | 134.39141472 |
| YDL150W | 630.959862342222 | -2.10460743029253 | 0.529316622030858 | -3.97608414830743 | 7.0059343719462e-05 | 0.000147199515181221 | 1428.1227020701 | 1369.29183549843 | 274.250218266318 | 242.733104070048 | 224.035937478862 | 247.32537666958 |
| YDL153C | 468.583231332135 | -2.61815046361529 | 0.425026902386725 | -6.15996410794975 | 7.27614336641631e-10 | 2.58875841330643e-09 | 1084.58280779885 | 995.549912791898 | 337.48217207571 | 140.038329271181 | 134.136166388618 | 119.709999666555 |
| YDL154W | 148.449348252675 | -2.1043279909066 | 0.391158586913451 | -5.37973103827633 | 7.45972035808532e-08 | 2.17650680353313e-07 | 313.138133716275 | 280.922500144248 | 128.669673449345 | 62.6838235785288 | 49.9443172723578 | 55.3376413552941 |
| YDL155W | 656.59772186338 | -1.14356640694632 | 0.168421429173489 | -6.78991036092173 | 1.12203238242664e-11 | 4.70731049962431e-11 | 921.173344815839 | 964.336301664759 | 827.897441736927 | 412.112797569476 | 351.0372014 | 463.029243993277 |
| YDL156W | 67.5139759677689 | -5.25435126134035 | 0.60130991556355 | -8.73817498322133 | 2.36906358836535e-18 | 1.84906695958335e-17 | 155.809022844263 | 137.175606795583 | 101.465228205769 | 2.6673967480225 | 5.70792197398374 | 2.2586792389916 |
| YDL157C | 2272.16600062375 | 1.36338526833259 | 0.202025715213261 | 6.74857290762902 | 1.4930637103672e-11 | 6.19975110463615e-11 | 1491.20635522168 | 1357.79208403053 | 966.860689062219 | 3480.95275616936 | 3213.56007135285 | 3122.62404790588 |
| YDL158C | 598.642760815025 | 1.47926210995165 | 0.196011163423321 | 7.54682582418493 | 4.4599440344764e-14 | 2.3575547683467e-13 | 317.698397799522 | 364.706403696042 | 266.16241022093 | 1012.27706587454 | 786.266251916261 | 844.746035382857 |
| YDL159W | 297.556204733624 | -0.539644887701021 | 0.223489383406158 | -2.41463321199602 | 0.015751062123912 | 0.0242145979937646 | 311.618045688526 | 317.064576186198 | 429.389081682385 | 260.071182932194 | 204.058210569919 | 263.136131342521 |
| YDL160C | 830.568835837642 | 0.136850205852361 | 0.210027473954698 | 0.651582401461816 | 0.514670603658298 | 0.577235610466491 | 665.038512140147 | 757.340775242681 | 950.685072971444 | 773.545056926525 | 1020.29105284959 | 816.512544895462 |
| YDL160C-A | 585.67839151373 | 1.21627656689672 | 0.292885331319857 | 4.15273978186516 | 3.28518234015221e-05 | 7.20772500611526e-05 | 447.665924172054 | 395.098604004045 | 214.694540841192 | 820.224500016919 | 866.177159552033 | 770.209620496134 |
| YDL161W | 177.78345959036 | -0.555733067714027 | 0.41449100489311 | -1.34076026054495 | 0.179998302421503 | 0.22990724488103 | 253.854700634068 | 281.743910963384 | 99.2594623752088 | 120.032853661013 | 168.38369823252 | 143.426131675966 |
| YDL162C | 2.23032801982056 | 0.282591669027932 | 1.27908859949546 | 0.220932052040337 | 0.825145340090005 | 0.857130280857766 | 1.52008802774891 | 1.64282163827046 | 2.94102110741359 | 1.33369837401125 | 1.42698049349594 | 4.51735847798319 |
| YDL164C | 913.364602489555 | 0.0221899084049009 | 0.135060876790207 | 0.164295604561853 | 0.869498441729604 | 0.895027280282211 | 903.692332496726 | 922.444349888862 | 894.070416653732 | 865.570244733301 | 863.323198565041 | 1031.08707259966 |
| YDL165W | 1435.81452453095 | 0.8947491452539 | 0.153836883299304 | 5.81621992115559 | 6.01932636951536e-09 | 1.95499710268396e-08 | 948.534929315319 | 940.515387909837 | 1124.20531830885 | 1727.13939434457 | 1790.8605193374 | 2083.63159796975 |
| YDL166C | 1160.07600546849 | -0.0842686032690535 | 0.226371289102176 | -0.372258353094494 | 0.709700503353428 | 0.75806187200111 | 1396.20085348737 | 1319.18577553118 | 866.865971410157 | 1185.657854496 | 1020.29105284959 | 1172.25452503664 |
| YDL167C | 880.945782443333 | -1.04214527551818 | 0.173874783409183 | -5.99365391050225 | 2.05177792179596e-09 | 7.00412189514137e-09 | 1293.59491161432 | 1177.90311463992 | 1087.44255446618 | 624.170839037265 | 478.038465321139 | 624.524809581176 |
| YDL168W | 2291.01588670908 | 0.57658550715276 | 0.188161874300311 | 3.06430571706846 | 0.00218175831857444 | 0.00378669640199996 | 1618.13370553871 | 1614.07225960073 | 2285.17340046036 | 2794.09809355357 | 2654.18371790244 | 2780.43414319865 |
| YDL169C | 41.4419446742937 | -0.200030812574956 | 0.43271375216884 | -0.462270523116875 | 0.643887335281221 | 0.698875520349877 | 45.6026408324673 | 36.1420760419501 | 50.7326141028845 | 28.0076658542363 | 59.9331807268293 | 28.2334904873949 |
| YDL170W | 772.786283360705 | 0.192008637694698 | 0.153305574388602 | 1.25245698638453 | 0.210403392458772 | 0.263909573290968 | 646.037411793286 | 703.127661179756 | 814.662846753565 | 872.238736603358 | 824.794725240651 | 775.856318593613 |
| YDL171C | 8165.31579813209 | -0.209776592230615 | 0.216464889801652 | -0.969102159813698 | 0.332494220206801 | 0.39628032198848 | 9891.97284057602 | 9969.46311184428 | 6412.16126943849 | 7455.37391072289 | 8229.39650599106 | 7033.52715021983 |
| YDL172C | 123.820768132696 | 0.434738814370647 | 0.203025399247931 | 2.1413025955425 | 0.032249643960648 | 0.0471000204298047 | 102.605941873051 | 109.247638944985 | 103.670994036329 | 145.373122767226 | 155.540873791057 | 126.486037383529 |
| YDL173W | 165.922817405161 | -4.24831042451807 | 0.574354776192582 | -7.39666596433701 | 1.39646242388028e-13 | 6.99957904272606e-13 | 443.105660088807 | 386.884495812693 | 115.435078465984 | 12.0032853661013 | 25.6856488829268 | 12.4227358144538 |
| YDL174C | 2016.47874571218 | -0.248677495343555 | 0.23076471982411 | -1.07762354459165 | 0.281201790604887 | 0.342270236753451 | 1814.22506111832 | 1913.88720858508 | 2841.76164503838 | 1805.82759841123 | 1633.89266505285 | 2089.27829606723 |
| YDL175C | 279.081209852954 | -1.614681499083 | 0.191979532708532 | -8.41069605859733 | 4.07584651132987e-17 | 2.86723866295939e-16 | 399.783151297963 | 403.312712195397 | 460.269803310227 | 128.03504390508 | 122.72032244065 | 160.366225968403 |
| YDL176W | 415.37977212633 | -0.521296114449368 | 0.140290349878355 | -3.71583729673054 | 0.000202531884851668 | 0.000404689403927269 | 500.869005143265 | 507.631886225572 | 460.269803310227 | 321.421308136711 | 361.026064854472 | 341.060565087731 |
| YDL177C | 126.304362022012 | -1.42814177090907 | 0.262725812988492 | -5.4358639323028 | 5.45315584718937e-08 | 1.60988478947976e-07 | 197.611443607358 | 184.817434305427 | 170.579224229988 | 84.0229975627088 | 48.5173367788618 | 72.2777356477311 |
| YDL178W | 2901.81622055581 | 1.30163940043395 | 0.117290043792563 | 11.0976120252457 | 1.28840346664802e-28 | 2.14822233539307e-27 | 1736.70057170313 | 1727.42695264139 | 1560.94695275976 | 4385.20025374899 | 3878.53298132195 | 4122.08961115966 |
| YDL179W | 1067.88811090982 | -1.42803626709004 | 0.172582089591473 | -8.27453341462266 | 1.28959603470142e-16 | 8.7128332094515e-16 | 1711.61911924527 | 1682.24935758895 | 1277.87367117121 | 570.822904076815 | 565.084275424391 | 599.679337952269 |
| YDL180W | 1259.12820197501 | 0.16084594649409 | 0.156168182396049 | 1.02995337479294 | 0.303031893257927 | 0.366076524431163 | 1105.86404018733 | 1255.93714245777 | 1204.34814348587 | 1283.01783579882 | 1525.44214754716 | 1180.15990237311 |
| YDL181W | 3382.84015395545 | 2.35966192837557 | 0.15276152156174 | 15.4467034908518 | 7.94017738578373e-54 | 6.68831045768744e-52 | 1059.50135534099 | 965.979123303029 | 1283.75571338603 | 5718.89862776024 | 5508.14470489431 | 5760.76139904806 |
| YDL182W | 3506.47863876841 | -1.3363749018548 | 0.198929864746277 | -6.71781938603972 | 1.84464131853621e-11 | 7.59640862985768e-11 | 5392.51227843925 | 5694.84120906454 | 3984.34834526857 | 1995.21276752083 | 1743.77016305203 | 2228.18706926521 |
| YDL183C | 107.247847081911 | -1.33077692650272 | 0.25680292125624 | -5.18209419111265 | 2.19408358599527e-07 | 6.10241258094569e-07 | 145.928450663895 | 142.104071710395 | 172.049734783695 | 49.3468398384163 | 74.2029856617887 | 59.8549998332773 |
| YDL184C | 2291.01102427791 | 0.199874254132246 | 0.457990035246962 | 0.43641616356231 | 0.66253481374431 | 0.716200133657598 | 2959.61139002712 | 2549.65918259575 | 888.188374438905 | 2608.71401956601 | 3216.41403233984 | 1523.47914669983 |
| YDL185C-A | 1.70612010212036 | 1.26061957137511 | 1.60269482241534 | 0.786562453278098 | 0.431538046022849 | 0.496973680132138 | 0.760044013874454 | 0.821410819135229 | 1.4705105537068 | 2.6673967480225 | 0 | 4.51735847798319 |
| YDL185W | 24710.9165200959 | 0.820231098565425 | 0.122132840485332 | 6.715893082532 | 1.86917815556397e-11 | 7.6779540956225e-11 | 16968.7426537611 | 18310.8899801625 | 18328.4435414015 | 31041.8296551119 | 34629.9626161594 | 28985.6306739791 |
| YDL186W | 11.2924679142695 | 0.33491334651122 | 0.599093288872103 | 0.559033714334795 | 0.576138711265898 | 0.635692573540977 | 10.6406161942424 | 7.39269737221706 | 11.7640844296544 | 10.66958699209 | 17.1237659219512 | 10.1640565754622 |
| YDL187C | 122.836418283977 | 1.40119658144362 | 0.2116448174278 | 6.62050976949446 | 3.57962256134447e-11 | 1.4393944161736e-10 | 72.2041813180732 | 69.8199196264945 | 60.2909327019787 | 176.048185369485 | 192.642366621951 | 166.012924065882 |
| YDL188C | 2074.8404053434 | 1.02054613178623 | 0.177648074685567 | 5.74476325506242 | 9.20496826646557e-09 | 2.92520451623203e-08 | 1170.46778136666 | 1288.79357522317 | 1650.64809653588 | 2727.41317485301 | 2950.9956605496 | 2660.7241435321 |
| YDL189W | 305.605957947081 | -1.79458826140185 | 0.228726178822404 | -7.84601163994992 | 4.29477098792317e-15 | 2.47168452774354e-14 | 517.589973448503 | 501.882010491625 | 405.125657546222 | 128.03504390508 | 112.731458986179 | 168.271603304874 |
| YDL190C | 1518.99029912841 | -0.633263370951154 | 0.107355428369177 | -5.8987550100724 | 3.66254573485364e-09 | 1.21386160634955e-08 | 1788.38356464659 | 1848.17434305427 | 1905.78167760401 | 1182.99045774798 | 1137.30345331626 | 1251.30829840134 |
| YDL191W | 8188.41982061564 | -0.545371382965772 | 0.292462146963965 | -1.86475887094191 | 0.0622152107157271 | 0.0863530615669176 | 12143.2232096722 | 10887.8004076375 | 6123.2059456351 | 7630.08839771836 | 5393.98626541464 | 6952.21469761613 |
| YDL192W | 13101.1027462592 | 0.291315973463786 | 0.216120117010221 | 1.3479354790929 | 0.177679135577181 | 0.227079186867704 | 13536.383887104 | 13285.4985886932 | 8526.75544566886 | 14531.9774832266 | 15014.6887525642 | 13711.3123202985 |
| YDL193W | 592.972615126901 | -0.156382362939133 | 0.142363833852081 | -1.09846973566065 | 0.27199942594377 | 0.332111671329595 | 592.834330822074 | 636.593384829803 | 644.81887780043 | 561.487015458736 | 615.028592696748 | 507.073489153613 |
| YDL194W | 291.990949534969 | -0.788667326826898 | 0.20343580589393 | -3.8767380371482 | 0.000105866234204584 | 0.000217293795902193 | 407.383591436707 | 387.705906631828 | 315.424513770108 | 196.053660979654 | 196.923308102439 | 248.454716289076 |
| YDL195W | 4546.54150087968 | -1.59993899594779 | 0.215997403185971 | -7.40721403289404 | 1.28980211529527e-13 | 6.50012161601021e-13 | 5819.6570142367 | 6057.90479112231 | 8635.57322664316 | 2079.23576508354 | 2090.52642297155 | 2596.35178522084 |
| YDL196W | 3.56103858195281 | 1.29896530046 | 1.03502721325426 | 1.25500593977223 | 0.209476603731787 | 0.262950503542552 | 2.28013204162336 | 2.46423245740569 | 1.4705105537068 | 2.6673967480225 | 5.70792197398374 | 6.77603771697479 |
| YDL197C | 131.385103526072 | -3.05640018293583 | 0.610282270526537 | -5.00817462761755 | 5.49486696938996e-07 | 1.46665461578038e-06 | 325.298837938266 | 318.707397824469 | 59.5556774251253 | 29.3413642282475 | 32.8205513504065 | 22.586792389916 |
| YDL198C | 7945.83834863328 | 0.927313893658853 | 0.161456254988606 | 5.74343740181693 | 9.27736699569886e-09 | 2.94676798893745e-08 | 4775.3565391732 | 5161.74558744578 | 6492.30409461551 | 10458.8626489962 | 10699.4997402325 | 10087.2614813365 |
| YDL199C | 241.74113928096 | 0.844778305067785 | 0.294413719361176 | 2.86935781016183 | 0.00411306216930173 | 0.00685087859016204 | 129.207482358657 | 149.496769082612 | 239.693220254208 | 282.744055290385 | 362.453045347968 | 286.852263351933 |
| YDL200C | 388.557603312318 | 0.412735730047588 | 0.205627948828187 | 2.00719665006458 | 0.0447287281870609 | 0.0637045522664201 | 380.022006937227 | 312.136111271387 | 308.071961001574 | 522.80976261241 | 366.733986828456 | 441.571791222857 |
| YDL201W | 1082.44975916905 | -1.8179310170392 | 0.170437135835637 | -10.6662847162155 | 1.46362173298037e-26 | 2.08180933335761e-25 | 1761.78202416098 | 1708.53450380128 | 1591.09241911075 | 433.451971553656 | 429.521128542277 | 570.316507845378 |
| YDL202W | 236.877062811411 | -2.25450110752203 | 0.27857749793623 | -8.09290457493488 | 5.82585057657578e-16 | 3.70455557251672e-15 | 423.344515728071 | 351.563830589878 | 401.449381161956 | 76.0208073186413 | 57.0792197398374 | 111.804622330084 |
| YDL203C | 506.686476606172 | -1.18495422347479 | 0.224122332674009 | -5.28708678576149 | 1.24279686297607e-07 | 3.54829481900369e-07 | 638.436971654541 | 620.165168447098 | 853.631376426795 | 314.752816266655 | 254.002527842277 | 359.129998999664 |
| YDL204W | 214.355070126045 | -1.51256638765395 | 0.445967689485658 | -3.39165016505661 | 0.000694730818310578 | 0.00128267124610373 | 210.532191843224 | 200.424239868996 | 541.147883764101 | 88.0240926847425 | 139.844088362602 | 106.157924232605 |
| YDL205C | 1380.49040890068 | 0.0557544626521629 | 0.132086732683496 | 0.422104942104678 | 0.672948420800869 | 0.725522516175937 | 1402.28120559837 | 1414.46943055086 | 1244.05192843595 | 1465.73451303836 | 1471.21688879431 | 1285.18848698622 |
| YDL206W | 604.956472972148 | -0.148460831996464 | 0.220911948157191 | -0.672036226355788 | 0.501560634201213 | 0.565315865404727 | 621.716003349303 | 661.235709403859 | 624.231730048535 | 530.811952856478 | 737.748915137399 | 453.994527037311 |
| YDL207W | 416.484884056467 | -0.160376689545075 | 0.307044631626789 | -0.522323704848265 | 0.601444967168429 | 0.659504996966091 | 558.632350197724 | 497.774956395949 | 262.486133836663 | 369.434449601116 | 420.959245581301 | 389.62216872605 |
| YDL208W | 3543.85733404979 | -1.88306959943926 | 0.257264867994766 | -7.31957540147911 | 2.48756776082109e-13 | 1.21768788654231e-12 | 6271.12315847812 | 5924.01482760327 | 4534.31929235491 | 1509.74655938074 | 1091.64007752439 | 1932.30008895731 |
| YDL209C | 126.20841525368 | -0.821978210458413 | 0.217285217268782 | -3.78294584781451 | 0.000154983142190246 | 0.000312763117686974 | 151.248758761016 | 150.318179901747 | 182.343308659643 | 92.0251878067763 | 84.1918491162602 | 97.1232072766386 |
| YDL210W | 347.435587447091 | 0.845849229823923 | 0.379522751282436 | 2.22871811232853 | 0.0258326663614383 | 0.0384026298465021 | 218.892675995843 | 183.174612667156 | 342.628959013684 | 350.762672364959 | 670.68083194309 | 318.473772697815 |
| YDL211C | 173.840489365418 | -0.495523443988251 | 0.224489160932166 | -2.20733794865928 | 0.027290457961577 | 0.040458493791723 | 179.370387274371 | 201.245650688131 | 230.134901655114 | 156.042709759316 | 117.012400466667 | 159.236886348907 |
| YDL212W | 9870.57697660929 | 1.32998461776949 | 0.166808320132586 | 7.97313117662451 | 1.54703815564705e-15 | 9.39521486659812e-15 | 6033.22938213542 | 6259.97185262958 | 4560.78848232163 | 14664.0136222537 | 13760.3728987813 | 13945.0856215341 |
| YDL213C | 289.25552838528 | -3.2079483489517 | 0.56242508220153 | -5.70377895735848 | 1.17180015905822e-08 | 3.68409880351508e-08 | 784.365422318437 | 628.37927663845 | 153.66835286236 | 42.67834796836 | 48.5173367788618 | 77.92443374521 |
| YDL214C | 30.7473657708179 | -1.64311399804198 | 0.41667238326492 | -3.94341949223279 | 8.03279655908297e-05 | 0.00016715020366446 | 39.5222887214716 | 45.1775950524376 | 55.1441457640049 | 12.0032853661013 | 15.6967854284553 | 16.940094292437 |
| YDL215C | 1834.89551365085 | 0.471817422963423 | 0.151354652428739 | 3.11729712560744 | 0.0018251751869701 | 0.00320121315919094 | 1390.88054539025 | 1476.07524198601 | 1744.76077197311 | 2183.26423825642 | 2261.76408219106 | 1952.62820210823 |
| YDL216C | 161.141500424854 | -2.52352610112247 | 0.250833063418471 | -10.0605799998241 | 8.25107977359947e-24 | 9.76578529408142e-23 | 307.05778160528 | 287.49378669733 | 228.664391101407 | 49.3468398384163 | 51.3712977658537 | 42.9149055408403 |
| YDL217C | 924.438577085075 | 1.47356615707836 | 0.169152270598729 | 8.71147724983263 | 2.99939994645255e-18 | 2.32704641778603e-17 | 442.345616074932 | 441.919020694753 | 583.792689821598 | 1325.69618376718 | 1405.5757860935 | 1347.30216605849 |
| YDL218W | 90.817714031353 | 2.79167884677441 | 0.368409572347467 | 7.57765013809529 | 3.51869720215948e-14 | 1.86914578650339e-13 | 17.4810123191124 | 16.4282163827046 | 34.5569980121097 | 148.040519515249 | 188.361425141464 | 140.038112817479 |
| YDL219W | 830.619977344006 | 0.514541078644103 | 0.129815619304147 | 3.96362996534782 | 7.3818645840957e-05 | 0.000154447657072402 | 691.640052625753 | 656.307244489048 | 704.374555225556 | 1053.62171546889 | 891.86280843496 | 985.913487819832 |
| YDL220C | 313.919403212799 | -0.883921623613581 | 0.296179375001524 | -2.98441315709115 | 0.00284122840963475 | 0.00485719753950737 | 330.619146035388 | 322.814451920145 | 568.352329007677 | 237.398310574003 | 182.65350316748 | 241.678678572101 |
| YDL221W | 5.64677286318799 | -0.356043155342965 | 1.02712190377398 | -0.346641575877944 | 0.728860607271685 | 0.775522579382078 | 1.52008802774891 | 6.57128655308183 | 11.028829152801 | 4.00109512203375 | 2.85396098699187 | 7.90537733647059 |
| YDL222C | 198.711771003618 | 1.22571316383952 | 0.417852423444158 | 2.93336377885893 | 0.00335310759364291 | 0.00567395143552516 | 79.0445774429432 | 75.5697953604411 | 202.195201134685 | 286.745150412419 | 302.519864621138 | 246.196037050084 |
| YDL223C | 127.878935181172 | -0.292109034162096 | 0.211388520704943 | -1.38185854741764 | 0.167015137989987 | 0.214670324349124 | 151.248758761016 | 147.032536625206 | 124.258141788224 | 104.028473172878 | 119.866361453659 | 120.83933928605 |
| YDL224C | 2105.68758806662 | 0.245369995522967 | 0.120588170737411 | 2.03477666194372 | 0.0418733503715564 | 0.0599008713078772 | 1744.30101184187 | 2042.84870718931 | 1994.01231082642 | 2353.97763012986 | 2261.76408219106 | 2237.22178622118 |
| YDL225W | 217.30732455507 | -3.29956891247244 | 0.560856980060706 | -5.8830843330421 | 4.026906059251e-09 | 1.33121879206432e-08 | 557.112262169975 | 510.096118682977 | 116.170333742837 | 44.0120463423713 | 47.0903562853659 | 29.3628301068907 |
| YDL226C | 1444.20812178236 | -0.459951322530215 | 0.23915534089498 | -1.92323249319443 | 0.0544508582416528 | 0.0762782433164924 | 1434.96309819497 | 1467.03972297552 | 2116.06468678408 | 1260.34496344063 | 971.773716070732 | 1415.06254322823 |
| YDL227C | 505.537979558306 | -0.770251099514711 | 0.36993078790594 | -2.08214921465406 | 0.0373288423939355 | 0.053971215284678 | 806.406698720796 | 746.662434593923 | 359.539830381312 | 432.118273179645 | 252.575547348781 | 435.925093125378 |
| YDL228C | 5.97258062616939 | -0.212689619698159 | 0.817971207267689 | -0.260020912482504 | 0.794847642446914 | 0.831379101582114 | 8.360484152619 | 5.7498757339466 | 5.14678693797379 | 2.6673967480225 | 7.13490246747968 | 6.77603771697479 |
| YDL229W | 129645.015193842 | 0.316600030960284 | 0.177257772751197 | 1.78609956588291 | 0.0740831384497108 | 0.101693806557635 | 123791.408715788 | 130995.31179241 | 91642.9529622844 | 147496.370578652 | 146204.140422113 | 137739.906691805 |
| YDL230W | 585.00363142346 | 0.546478170887803 | 0.246854445260522 | 2.21376678192312 | 0.0268448262691101 | 0.039816040059787 | 421.064383686448 | 377.02756598307 | 627.908006432802 | 658.846996761558 | 784.839271422765 | 640.335564254117 |
| YDL231C | 1308.55588574274 | -0.0991438975731798 | 0.140009579993583 | -0.708122241190379 | 0.478869348921491 | 0.542618203547308 | 1227.47108240724 | 1309.32884570156 | 1523.44893364024 | 1311.02550165306 | 1240.04604884797 | 1240.01490220639 |
| YDL232W | 736.046028762412 | 1.42243155371909 | 0.256870499044096 | 5.53754346650336 | 3.06743581400227e-08 | 9.23648499982299e-08 | 458.306540366296 | 445.204663971294 | 296.30787657192 | 1009.60966912652 | 1327.09185895122 | 879.755563587227 |
| YDL233W | 350.201973656176 | -1.42666199918737 | 0.16309738610284 | -8.74730143307012 | 2.18515419815287e-18 | 1.71377389712449e-17 | 481.867904796404 | 484.632383289785 | 564.67605262341 | 198.721057727676 | 188.361425141464 | 182.953018358319 |
| YDL234C | 2153.77445841353 | -2.24708805029965 | 0.282145318567279 | -7.96429322914255 | 1.66170787863143e-15 | 1.00727451409378e-14 | 3517.48369621097 | 3689.77739955545 | 3465.99337508692 | 514.807572368343 | 1071.66235061545 | 662.922356644033 |
| YDL235C | 1366.11145260714 | -1.11340662093162 | 0.185443618143148 | -6.00401691942913 | 1.92494628746627e-09 | 6.58849689736477e-09 | 2078.72037794663 | 1945.92223053136 | 1582.26935578851 | 849.565864245166 | 752.018720072358 | 988.172167058823 |
| YDL236W | 5769.67675735765 | 0.753520819341015 | 0.0950290874805077 | 7.92937025198287 | 2.20259947626529e-15 | 1.31064772505107e-14 | 4440.17712905456 | 4323.90655192785 | 4124.78210314757 | 6972.57509933082 | 7458.82703950326 | 7297.79262118185 |
| YDL237W | 2834.79604887068 | 1.29533316096497 | 0.164552437369052 | 7.87185642264201 | 3.49417036621689e-15 | 2.02711887256554e-14 | 1469.92512283319 | 1562.32337799521 | 1891.07657206694 | 3765.03050983376 | 4502.12345697968 | 3818.29725351529 |
| YDL238C | 380.251143006881 | 0.680475720658159 | 0.310977583955198 | 2.18818254358872 | 0.0286563081436849 | 0.042309313594341 | 237.13373232883 | 245.601834921433 | 393.361573116568 | 381.437734967218 | 626.444436644716 | 397.527546062521 |
| YDL239C | 94.8368232000395 | -0.581289876095281 | 0.256321447051391 | -2.26781598957943 | 0.0233404244305309 | 0.0349782793106339 | 118.566866164415 | 123.211622870284 | 99.2594623752088 | 92.0251878067763 | 67.068083194309 | 68.8897167892437 |
| YDL240W | 358.528329147738 | -0.857618470269024 | 0.174758807407781 | -4.90744062053401 | 9.22725784236977e-07 | 2.40546601147952e-06 | 449.946056213677 | 484.632383289785 | 452.917250541693 | 240.065707322025 | 225.462917972358 | 298.145659546891 |
| YDL241W | 893.070836032414 | -0.265294923765228 | 0.25325634525387 | -1.04753515059727 | 0.294852829059515 | 0.357127068026147 | 935.614181079453 | 984.87157214314 | 1003.62345290489 | 624.170839037265 | 1114.47176542033 | 695.673205609412 |
| YDL242W | 10.0411364616171 | 0.169181159440545 | 0.597280112357805 | 0.283252624589642 | 0.776983190772181 | 0.815587146034693 | 9.8805721803679 | 8.21410819135229 | 10.2935738759476 | 12.0032853661013 | 8.56188296097562 | 11.293396194958 |
| YDL243C | 453.387407451133 | -1.09118430980778 | 0.529668431410079 | -2.06012713822275 | 0.0393863881042856 | 0.0567059074904321 | 806.406698720796 | 870.695468283343 | 174.255500614255 | 233.397215451969 | 345.329279426017 | 290.24028221042 |
| YDL244W | 63.5744877323043 | 1.26244231545073 | 0.305193535022814 | 4.13653033428889 | 3.52596762211069e-05 | 7.70533220923516e-05 | 33.441936610476 | 34.4992544036796 | 44.1153166112039 | 72.0197121966075 | 107.023537012195 | 90.3471695596638 |
| YDL248W | 384.975859675859 | 0.071480102192409 | 0.230291078658568 | 0.310390235734603 | 0.756264218490778 | 0.798490919930194 | 400.543195311837 | 432.883501684266 | 292.631600187653 | 361.432259357049 | 466.622621373171 | 355.741980141176 |
| YDR001C | 2399.97273262343 | 0.168307132647366 | 0.221647300985377 | 0.759346637198481 | 0.447645226051609 | 0.513063604200518 | 1924.43144313012 | 1969.74314428628 | 2885.87696164959 | 2216.6066976067 | 2855.38796748537 | 2547.79018158252 |
| YDR002W | 5761.45769313415 | -1.27368027024007 | 0.231430157738774 | -5.50351899979141 | 3.72284388319126e-08 | 1.11376224291414e-07 | 9587.95523502624 | 8996.09129116903 | 5871.01338567439 | 3396.92975860665 | 2975.25432893903 | 3741.50215938958 |
| YDR003W | 866.093806167145 | 1.32923066104904 | 0.271816329844731 | 4.89017956282586 | 1.00744034539142e-06 | 2.61789185905801e-06 | 400.543195311837 | 411.52682038675 | 666.876536106032 | 1050.95431872087 | 1501.18347915772 | 1165.47848731966 |
| YDR003W-A | 186.052055551164 | 1.09226344587787 | 0.277089008722154 | 3.9419226728445 | 8.08310386365158e-05 | 0.000168143077805145 | 110.96642602567 | 105.961995668445 | 138.963247325292 | 214.725438215811 | 336.767396465041 | 208.927829606723 |
| YDR004W | 545.056800841057 | -0.188716849662824 | 0.150933269872223 | -1.25033301022755 | 0.211177924624245 | 0.264789266377896 | 603.474947016317 | 543.773962267522 | 594.821518974399 | 566.821808954781 | 456.6337579187 | 504.814809914622 |
| YDR005C | 1391.75704568058 | 0.337745240846767 | 0.109057108886073 | 3.09695758760298 | 0.00195517845210679 | 0.00341445542282301 | 1171.98786939441 | 1208.29531494792 | 1308.0191375222 | 1559.09339921915 | 1579.6674063 | 1523.47914669983 |
| YDR006C | 1682.61623054199 | -0.342684345820547 | 0.11174793841016 | -3.06658315755909 | 0.00216520571901664 | 0.00375997973053332 | 1928.23166319949 | 1872.81666762832 | 1844.75548962518 | 1463.06711629034 | 1395.58692263903 | 1591.23952386958 |
| YDR007W | 5141.26912976089 | 1.71866966774203 | 0.100762804181152 | 17.05658830864 | 3.12268294017443e-65 | 5.06343038749283e-63 | 2426.06049228726 | 2443.69718692731 | 2318.99514319562 | 8283.60060098388 | 7463.10798098374 | 7912.15337418756 |
| YDR008C | 21.9062596341402 | 0.532094283111245 | 0.438497021951131 | 1.21345016379734 | 0.224957757182016 | 0.280429754580541 | 13.6807922497402 | 18.071038020975 | 22.057658305602 | 24.0065707322025 | 24.2586683894309 | 29.3628301068907 |
| YDR009W | 129.23242484657 | -0.430778742844365 | 0.281843170501231 | -1.5284342071453 | 0.126404767171373 | 0.165705076371395 | 118.566866164415 | 137.175606795583 | 188.960606151323 | 100.027378050844 | 131.282205401626 | 99.3818865156302 |
| YDR010C | 20.6913130473472 | 0.822127426057959 | 0.434230423668708 | 1.89329761630243 | 0.0583183019881425 | 0.081292178528926 | 15.9609242913635 | 14.7853947444341 | 13.9698502602146 | 28.0076658542363 | 29.9665903634147 | 21.4574527704202 |
| YDR011W | 4007.8326056387 | -0.616925927674599 | 0.182403440236905 | -3.38220554871848 | 0.00071906318849091 | 0.0013257088802024 | 4969.16776271118 | 5666.09183039481 | 3920.38113618232 | 3368.92209275242 | 3185.02046148293 | 2937.41235030857 |
| YDR012W | 59185.1318037915 | 0.430082903724407 | 0.182889798976976 | 2.35159591256672 | 0.018693069705743 | 0.0284341580936794 | 55936.1992451043 | 55985.7186106189 | 39362.6265016235 | 68701.4706420675 | 65397.0890364252 | 69727.6867869096 |
| YDR013W | 415.876286379777 | 0.138089866040217 | 0.192295085062677 | 0.718114381317691 | 0.472686763502544 | 0.537002849995021 | 409.663723478331 | 455.061593800917 | 323.512321815495 | 401.443210577386 | 459.487718905691 | 446.08914970084 |
| YDR014W | 135.780407014826 | -1.9006534442871 | 0.331447153810035 | -5.73440870569804 | 9.78531140216573e-09 | 3.09597706119253e-08 | 264.49531682831 | 237.387726730081 | 140.433757878999 | 53.34793496045 | 67.068083194309 | 51.9496224968067 |
| YDR014W-A | 12.190324667833 | -1.20328603611943 | 0.606996515173347 | -1.9823606990162 | 0.047438884932099 | 0.0672837541372391 | 17.4810123191124 | 16.4282163827046 | 16.9108713676282 | 10.66958699209 | 7.13490246747968 | 4.51735847798319 |
| YDR016C | 550.698592951096 | 1.85574564239635 | 0.157566166418089 | 11.7775642105316 | 5.09440141784218e-32 | 1.05279940178404e-30 | 266.015404856059 | 212.745402156024 | 236.016943869941 | 853.5669593672 | 926.110340278862 | 809.736507178487 |
| YDR017C | 683.776720377106 | -1.99200528100446 | 0.258680932893342 | -7.70062663190485 | 1.35400581803377e-14 | 7.42356866928745e-14 | 1251.03244683735 | 1251.83008836209 | 776.429572357189 | 277.40926179434 | 229.743859452846 | 316.215093458823 |
| YDR018C | 90.6749935495389 | 0.348105408802727 | 0.329652618932508 | 1.05597646980622 | 0.290978965203943 | 0.353066006603062 | 67.6439172348264 | 63.2486330734126 | 108.08252569745 | 88.0240926847425 | 125.574283427642 | 91.4765091791596 |
| YDR019C | 450.547579624521 | 1.32481195469412 | 0.222707878105585 | 5.94865330298747 | 2.70357601168014e-09 | 9.07629089635477e-09 | 228.773248176211 | 214.388223794295 | 327.923853476616 | 673.517678875681 | 582.208041346342 | 676.474432077983 |
| YDR020C | 390.295248484259 | -0.522610759941633 | 0.219997938588325 | -2.37552571308215 | 0.0175239713912375 | 0.0267562331552652 | 455.266364310798 | 419.740928578102 | 506.590885751991 | 330.75719675479 | 245.440644881301 | 383.975470628571 |
| YDR021W | 273.024635456336 | -2.26267131982688 | 0.285141421579629 | -7.93526000990004 | 2.10054795777582e-15 | 1.2533720380988e-14 | 520.630149504001 | 505.989064587301 | 329.394364030322 | 113.364361790956 | 72.7760051682927 | 95.9938676571428 |
| YDR022C | 131.479781740968 | -0.0191365753395142 | 0.239276104219168 | -0.0799769596799595 | 0.936255580771322 | 0.949578373242032 | 139.8480985529 | 117.461747136338 | 138.963247325292 | 132.036139027114 | 155.540873791057 | 105.028584613109 |
| YDR023W | 10285.0188678334 | -1.07385870155136 | 0.191934470149792 | -5.59492362530442 | 2.20718909950067e-08 | 6.74638477820988e-08 | 15421.2930415127 | 15672.5184291002 | 10742.814850105 | 6539.12312777716 | 6314.38868371952 | 7019.97507478588 |
| YDR024W | 255.591368414854 | -0.00579341515702085 | 0.194770135256617 | -0.0297448843961 | 0.976270515171579 | 0.981871695053941 | 212.812323884847 | 265.315694580679 | 290.425834357092 | 258.737484558183 | 239.732722907317 | 266.524150201008 |
| YDR025W | 46874.5795039341 | 0.451187475632215 | 0.0938465225782164 | 4.80771650602369 | 1.52664045991576e-06 | 3.89374361895936e-06 | 39624.8946633447 | 40073.3482223313 | 39114.8454733239 | 52889.1427197901 | 52131.878368887 | 57413.3675759274 |
| YDR026C | 184.871726346916 | -2.89953607969206 | 0.556627076169991 | -5.20911792441543 | 1.89740497132243e-07 | 5.31601237321697e-07 | 452.2261882553 | 431.240680045995 | 94.8479307140884 | 46.6794430903938 | 35.6745123373984 | 48.5616036383193 |
| YDR027C | 555.729901706295 | -0.505076854307068 | 0.137457046628826 | -3.67443406281616 | 0.000238377192026221 | 0.000471089112578326 | 678.719304389888 | 643.164671382884 | 635.260559201336 | 445.455256919758 | 428.094148048781 | 503.685470295126 |
| YDR028C | 615.762745374108 | -3.61090842871502 | 0.490403742787764 | -7.36313391123066 | 1.7964199834012e-13 | 8.88755149682701e-13 | 1596.85247315023 | 1436.64752266752 | 381.597488686914 | 77.3545056926525 | 102.742595531707 | 99.3818865156302 |
| YDR030C | 141.253906063792 | -0.603260319732372 | 0.295771657727628 | -2.039615034 | 0.0413886834721261 | 0.0592990945438944 | 154.288934816514 | 126.497266146825 | 230.134901655114 | 120.032853661013 | 107.023537012195 | 109.545943091092 |
| YDR031W | 344.056247361889 | 1.45147576532694 | 0.213461316810474 | 6.79971334860484 | 1.04827517927711e-11 | 4.40928197976092e-11 | 204.451839732228 | 170.853450380128 | 177.196521721669 | 620.169743915231 | 452.352816438212 | 439.313111983865 |
| YDR032C | 6978.12545437535 | 1.74572163894147 | 0.158727932815824 | 10.9982005559606 | 3.89833272562352e-28 | 6.25856090554311e-27 | 2909.44848511141 | 2932.43662431277 | 3774.80059136535 | 10125.4380554934 | 10875.0183409325 | 11251.6106290366 |
| YDR033W | 44819.0929540173 | 0.472215357646585 | 0.159558374202232 | 2.95951472310739 | 0.0030812397825402 | 0.00524400976897291 | 40422.180833899 | 40251.5943700836 | 31972.57571397 | 53111.87034825 | 56321.4930977911 | 46834.8433601102 |
| YDR034C | 999.086253443335 | -1.40717036958373 | 0.184437117976947 | -7.62954000267786 | 2.35592821306269e-14 | 1.27020368993554e-13 | 1579.37146083112 | 1573.00171864396 | 1200.6718671016 | 616.168648793198 | 513.712977658537 | 511.590847631596 |
| YDR034C-A | 4.23448718373198 | 1.86389835959602 | 1.01236425505647 | 1.84113410789287 | 0.065601908467354 | 0.0906656676580563 | 1.52008802774891 | 2.46423245740569 | 1.4705105537068 | 8.0021902440675 | 8.56188296097562 | 3.38801885848739 |
| YDR034C-D | 6.33290436229605 | -3.78792303735889 | 1.50543138527639 | -2.5161711615727 | 0.0118637551943582 | 0.0185194503467165 | 16.720968305238 | 17.2496272018398 | 1.4705105537068 | 0 | 1.42698049349594 | 1.1293396194958 |
| YDR034W-B | 336.063066610396 | 0.951367753183051 | 0.320630489078239 | 2.96717806194314 | 0.00300546846579173 | 0.00512150223723685 | 185.450739385367 | 188.103077581967 | 313.218747939548 | 405.44430569942 | 586.48898282683 | 337.672546229244 |
| YDR035W | 6531.08987199073 | 0.067771566619032 | 0.153699880967921 | 0.440934411869693 | 0.659260480258718 | 0.713136003161782 | 5885.0207994299 | 6265.72172836353 | 6981.24885372302 | 6792.5258188393 | 7440.27629308781 | 5821.74573850084 |
| YDR036C | 604.517583244043 | -0.585778459609251 | 0.270068681634188 | -2.16899810842449 | 0.0300828275164088 | 0.0442141897283996 | 595.114462863698 | 600.451308787852 | 981.565794599287 | 490.80100163614 | 402.408499165854 | 556.764432411428 |
| YDR037W | 17675.7303094213 | -0.59587902880648 | 0.167834085194937 | -3.55040531912439 | 0.000384638462595716 | 0.000738096174081602 | 19770.2648889023 | 20589.4835924437 | 23466.4074160531 | 14756.0388100605 | 11754.038324926 | 15718.1488241425 |
| YDR038C | 473.863374894899 | 0.361611029492645 | 0.163850524429743 | 2.20695680255634 | 0.0273170773989131 | 0.0404794525952365 | 398.263063270214 | 438.633377418212 | 407.331423376783 | 461.459637407893 | 602.185768255285 | 535.306979641008 |
| YDR040C | 183.762595146503 | -0.44911692550319 | 0.314581485195306 | -1.42766483928436 | 0.15338835110066 | 0.198340678875374 | 247.014304509198 | 257.922997208462 | 131.610694556758 | 138.70463089717 | 166.956717739024 | 160.366225968403 |
| YDR041W | 1330.75148903511 | 1.72072252681524 | 0.296433700597262 | 5.8047466376066 | 6.44634060989355e-09 | 2.08950350803446e-08 | 526.710501614997 | 456.704415439187 | 874.953779455544 | 2369.98201061799 | 1629.61172357236 | 2126.54650351059 |
| YDR042C | 125.12876470403 | -0.427686475723599 | 0.202948884030204 | -2.10736056897927 | 0.0350863368149018 | 0.0509561085045797 | 129.207482358657 | 153.603823178288 | 147.786310647533 | 106.6958699209 | 108.450517505691 | 105.028584613109 |
| YDR043C | 1065.07898957544 | -1.34498492667437 | 0.218875239205692 | -6.14498438268019 | 7.99713827890181e-10 | 2.83594526391236e-09 | 1450.16397847246 | 1459.6470256033 | 1674.17626539519 | 554.81852358868 | 770.569466487805 | 481.09867790521 |
| YDR044W | 6610.00010926743 | 1.56023092807542 | 0.249332156975778 | 6.25764019771824 | 3.90846251751862e-10 | 1.44117611646537e-09 | 3515.20356416935 | 3385.03398565628 | 3142.48105327142 | 7524.72622617147 | 13489.2466050171 | 8603.30922131899 |
| YDR045C | 933.347469847708 | 0.201025623341608 | 0.333622508404316 | 0.602554139116974 | 0.546805336996527 | 0.609168570209459 | 1074.70223561848 | 1057.15572422704 | 473.504398293589 | 1148.31430002369 | 941.807125707318 | 904.601035216134 |
| YDR046C | 9114.04096878154 | 0.0847495873323934 | 0.188853906727896 | 0.448757395601575 | 0.653606675675268 | 0.70805353156475 | 9653.31902021944 | 9861.85829453756 | 7023.89365978051 | 8867.7604888008 | 10464.0479588057 | 8813.36639054521 |
| YDR047W | 3989.13003251042 | 0.652767024411452 | 0.124367205126944 | 5.24870703450449 | 1.53170435802134e-07 | 4.33692961109648e-07 | 3437.67907475416 | 3008.00641967321 | 2859.40777168287 | 4758.63579847214 | 5032.96020056017 | 4838.09092992 |
| YDR048C | 17.0304530573689 | 1.84067974474077 | 0.50058953539296 | 3.67702401788294 | 0.000235970830272986 | 0.000466617928399569 | 6.84039612487009 | 6.57128655308183 | 8.82306332224078 | 24.0065707322025 | 29.9665903634147 | 25.9748112484034 |
| YDR049W | 191.890567790278 | -3.69310147248495 | 0.557777268527629 | -6.62110430249994 | 3.56525283939017e-11 | 1.43450557793329e-10 | 504.669225212638 | 455.883004620052 | 108.08252569745 | 25.3402691062138 | 31.3935708569106 | 25.9748112484034 |
| YDR050C | 162065.918968709 | 1.87471199343199 | 0.17348839613471 | 10.8059791617205 | 3.22497717061525e-27 | 4.83076257011791e-26 | 61490.6008984988 | 63405.5225398675 | 83446.3271359227 | 237850.434322792 | 275128.974048484 | 251073.654866686 |
| YDR051C | 1023.55970678955 | 0.0873341616558667 | 0.220341819968789 | 0.396357630468141 | 0.691841217982197 | 0.74186184689696 | 1127.90531658969 | 1099.04767600294 | 751.430892944173 | 1108.30334880335 | 906.132613369919 | 1148.53839302723 |
| YDR052C | 222.417691663591 | -2.25180608181642 | 0.469843682451833 | -4.79267076672307 | 1.64575582448585e-06 | 4.18438740792442e-06 | 489.468344935149 | 472.311221002757 | 141.169013155852 | 89.3577910587538 | 69.9220441813009 | 72.2777356477311 |
| YDR053W | 2.03799535302239 | 0.0705699906084598 | 1.92218598162124 | 0.0367134040530971 | 0.97071352096005 | 0.977495403966292 | 0 | 0.821410819135229 | 5.14678693797379 | 4.00109512203375 | 0 | 2.2586792389916 |
| YDR054C | 314.651865710046 | -5.21017720889659 | 0.359370640712265 | -14.4980602716185 | 1.24622018346897e-47 | 7.09033693857874e-46 | 785.885510346186 | 582.380270766877 | 470.563377186175 | 17.3380788621463 | 11.4158439479675 | 20.3281131509244 |
| YDR055W | 20540.0405720966 | -0.0633866552062809 | 0.26797480813915 | -0.236539604772723 | 0.813013978874054 | 0.847101793891906 | 23264.1872206832 | 24065.6941790239 | 15643.2912703329 | 16528.5239491214 | 26407.7010126358 | 17330.8458007825 |
| YDR056C | 1607.12575818216 | -0.0747772206745622 | 0.191139585153793 | -0.391217866327352 | 0.695636203191186 | 0.744867172085971 | 1812.70497309057 | 1759.46197458766 | 1373.45685716215 | 1561.76079596717 | 1793.71448032439 | 1341.65546796101 |
| YDR057W | 321.772090661326 | -2.8884520866058 | 0.258518918259314 | -11.1730781872933 | 5.52329783907709e-29 | 9.3780392105377e-28 | 630.076487501923 | 628.37927663845 | 442.623676665746 | 93.3588861807875 | 62.7871417138212 | 73.4070752672269 |
| YDR058C | 293.645535254946 | 1.06709445776617 | 0.194789046127864 | 5.47820567418204 | 4.29660541163172e-08 | 1.27951252065396e-07 | 160.36928692751 | 181.531791028886 | 227.1938805477 | 384.10513171524 | 400.981518672358 | 407.691602637983 |
| YDR059C | 820.026892941535 | 1.5379971920905 | 0.193455578294818 | 7.95013101016223 | 1.86314377408088e-15 | 1.12099726518447e-14 | 377.741874895604 | 377.02756598307 | 505.120375198285 | 1084.29677807115 | 1364.19335178211 | 1211.78141171899 |
| YDR060W | 1138.12412412171 | -4.37653114173938 | 0.517821583026952 | -8.45181291238606 | 2.86813937173533e-17 | 2.04650736689498e-16 | 3108.58001674652 | 2777.18997949621 | 629.378516986509 | 89.3577910587538 | 111.304478492683 | 112.93396194958 |
| YDR061W | 772.035691406643 | 0.119766904844229 | 0.177061779090089 | 0.676413088469485 | 0.498778409602932 | 0.562719910364344 | 653.637851932031 | 706.413304456297 | 860.248673918476 | 856.234356115223 | 712.063266254472 | 843.616695763361 |
| YDR062W | 5136.99859757386 | -0.294547216361944 | 0.143080948682047 | -2.05860541934541 | 0.0395320531881194 | 0.0568903698642429 | 5891.1011515409 | 6174.54512743952 | 4912.97575993441 | 4881.33604888118 | 4594.87718905691 | 4367.15630859025 |
| YDR063W | 1026.44001729383 | 0.200646914745379 | 0.163133228991539 | 1.22995735440133 | 0.218713074803222 | 0.273380806161822 | 934.094093051704 | 826.33928405004 | 1105.08868111066 | 1061.62390571296 | 1071.66235061545 | 1159.83178922218 |
| YDR064W | 104897.720245713 | 0.555526165365717 | 0.291374071428528 | 1.90657378208748 | 0.0565758028587757 | 0.0790671530579658 | 102583.900596649 | 102305.896112474 | 49953.2435094199 | 124666.121812328 | 125481.529695565 | 124395.629747843 |
| YDR065W | 163.273156681466 | -3.10559889063649 | 0.275239482366868 | -11.2832609040334 | 1.58750181654125e-29 | 2.78284777894231e-28 | 343.539894271253 | 286.672375878195 | 247.045773022742 | 37.343554472315 | 35.6745123373984 | 29.3628301068907 |
| YDR066C | 256.38073919655 | 0.568777197498754 | 0.187038459586965 | 3.04096386783114 | 0.00235822128388797 | 0.00406901389925443 | 224.973028106838 | 209.459758879483 | 184.549074490203 | 321.421308136711 | 332.486454984553 | 265.394810581513 |
| YDR067C | 441.384494477959 | -0.373013555526212 | 0.177921872613274 | -2.09650196486513 | 0.0360376858033279 | 0.0522091646460542 | 570.793054419715 | 497.774956395949 | 425.712805298118 | 362.76595773106 | 409.543401633334 | 381.71679138958 |
| YDR068W | 39.2659270189738 | -1.69042784331309 | 0.562655838236808 | -3.00437270607619 | 0.0026612909548691 | 0.004560405055028 | 67.6439172348264 | 83.7839035517934 | 28.6749557972825 | 10.66958699209 | 19.9777269089431 | 24.8454716289076 |
| YDR069C | 387.084431546919 | 0.2628139976204 | 0.218388510469597 | 1.20342410438753 | 0.228812244729301 | 0.284909902790953 | 307.05778160528 | 305.564824718305 | 442.623676665746 | 410.779099195465 | 462.341679892683 | 394.139527204033 |
| YDR070C | 195.195202831535 | 1.46332658862744 | 0.237140579464798 | 6.1707135570387 | 6.79824847714548e-10 | 2.43207058040627e-09 | 113.246558067294 | 85.4267251900638 | 112.49405735857 | 261.404881306205 | 346.756259919512 | 251.842735147563 |
| YDR071C | 1721.25904739593 | -1.49125989800026 | 0.292233043199998 | -5.10298179039142 | 3.34343184663199e-07 | 9.10390384435563e-07 | 2868.40610836219 | 2516.80274983034 | 2233.70553108062 | 1029.61514473669 | 550.814470489431 | 1128.2102798763 |
| YDR072C | 4530.79200942579 | 0.52692018729959 | 0.186060911121427 | 2.83197681943905 | 0.00462612022492015 | 0.00765824802930886 | 3726.49580002645 | 4263.12215131184 | 3147.6278402094 | 4841.32509766084 | 6214.5000491748 | 4991.68111817143 |
| YDR073W | 280.130929698624 | -2.0242949548548 | 0.207698159983424 | -9.74633070902676 | 1.91256450781654e-22 | 2.00726430383465e-21 | 529.750677670495 | 429.597858407725 | 389.685296732301 | 112.030663416945 | 111.304478492683 | 108.416603471597 |
| YDR074W | 2887.06907240993 | 0.126173170200758 | 0.299635089031311 | 0.421089434514036 | 0.673689776379355 | 0.726112392968352 | 2229.20909269377 | 2259.70116344102 | 3793.18197328668 | 2534.02691062138 | 3964.15181093171 | 2542.14348348504 |
| YDR075W | 465.827699436891 | -0.396210791972487 | 0.169949868953357 | -2.33133920262821 | 0.0197354806172158 | 0.0298866045489754 | 586.753978711079 | 553.630892097144 | 447.77046360372 | 414.780194317499 | 402.408499165854 | 389.62216872605 |
| YDR076W | 76.9804777142815 | -1.53820593076383 | 0.292366813651566 | -5.26121932770734 | 1.4310320764717e-07 | 4.06022486788953e-07 | 132.247658414155 | 100.212119934498 | 111.023546804863 | 48.013141464405 | 34.2475318439025 | 36.1388678238655 |
| YDR077W | 55259.4350868496 | -2.4698676102082 | 0.202627679463292 | -12.1891916087192 | 3.54924627197955e-34 | 8.68694766794693e-33 | 84677.2636297668 | 89138.6806817359 | 107043.609991256 | 13171.6051417351 | 18981.6945244829 | 18543.756552121 |
| YDR078C | 195.082577477713 | 0.470047650673173 | 0.226865993210055 | 2.07191762865031 | 0.0382731236542372 | 0.0552379795330179 | 185.450739385367 | 170.032039560992 | 135.286970941025 | 266.73967480225 | 204.058210569919 | 208.927829606723 |
| YDR079C-A | 241.27797542627 | 0.84696446040464 | 0.315281798659947 | 2.68637283853531 | 0.00722324370661529 | 0.0116716389340077 | 216.612543954219 | 197.96000741159 | 102.935738759476 | 333.424593502813 | 278.261196231708 | 318.473772697815 |
| YDR079W | 526.716155715861 | 1.89422431707273 | 0.134194417691004 | 14.1155224611084 | 3.04748962171622e-45 | 1.56873156241678e-43 | 208.2520598016 | 229.173618538729 | 232.340667485674 | 837.562578879065 | 859.042257084553 | 793.925752505546 |
| YDR080W | 514.584074154025 | -0.891744890870872 | 0.160191497001385 | -5.56674297677086 | 2.59544785488616e-08 | 7.87374873095961e-08 | 701.520624806121 | 694.092142169269 | 611.732390342027 | 368.100751227105 | 316.789669556098 | 395.268866823529 |
| YDR081C | 633.201134647221 | -1.76526196411391 | 0.162308003120684 | -10.8760007527253 | 1.50000588379229e-27 | 2.32752109145378e-26 | 1063.30157541036 | 975.014642313517 | 898.481948314853 | 294.747340656486 | 252.575547348781 | 315.085753839328 |
| YDR082W | 273.412516753278 | -0.677963553052221 | 0.19435033257065 | -3.48835808040497 | 0.000485996743318289 | 0.000917930948503909 | 360.260862576491 | 345.813954855931 | 303.660429340454 | 245.40050081807 | 179.799542180488 | 205.539810748235 |
| YDR083W | 663.176420304123 | -1.0528594709185 | 0.221756090548581 | -4.74782662480174 | 2.05614171651945e-06 | 5.19117756844888e-06 | 1057.22122329937 | 959.407836749948 | 668.347046659739 | 464.127034155915 | 410.97038212683 | 418.984998832941 |
| YDR084C | 1925.18655757295 | 1.33715610700107 | 0.104989264311113 | 12.7361222671177 | 3.7245163703542e-37 | 1.15034348467225e-35 | 1085.34285181272 | 1119.58294648132 | 1070.53168309855 | 2622.05100330612 | 2908.18624574472 | 2745.42461499428 |
| YDR085C | 346.920981467013 | -2.12949106209151 | 0.281151352784816 | -7.57418038717868 | 3.61401961972e-14 | 1.91821041354369e-13 | 649.837631862658 | 657.128655308183 | 387.479530901741 | 108.029568294911 | 141.271068856098 | 137.779433578487 |
| YDR086C | 4077.23415260847 | -0.00171744994267325 | 0.221977375374041 | -0.00773704950686652 | 0.993826789242422 | 0.995822733666978 | 4770.03623107607 | 4552.25875964744 | 2916.75768327743 | 4218.48795699758 | 4103.99589929431 | 3901.86838535798 |
| YDR087C | 1069.98640765193 | -2.08674469164406 | 0.262787798097914 | -7.94079750562294 | 2.00885401199797e-15 | 1.20308653017718e-14 | 1964.71377586546 | 1954.95774954185 | 1277.87367117121 | 440.120463423713 | 306.800806101626 | 475.451979807731 |
| YDR088C | 12.7877488352233 | -3.172627013156 | 0.884729827329235 | -3.58598400907689 | 0.000335809536880555 | 0.000650167360061875 | 22.8013204162336 | 35.3206652228149 | 11.028829152801 | 1.33369837401125 | 2.85396098699187 | 3.38801885848739 |
| YDR089W | 634.60699094942 | 0.42446752156353 | 0.247707069268238 | 1.71358662801779 | 0.0866046711237409 | 0.117317856497198 | 464.386892477291 | 460.811469534864 | 700.698278841289 | 796.217929284716 | 589.342943813822 | 796.184431744538 |
| YDR090C | 916.959274447518 | 0.34263440144498 | 0.148617440909835 | 2.30547908339273 | 0.0211397568793498 | 0.0318717952392987 | 804.126566679173 | 890.409327942588 | 730.843745192278 | 1032.28254148471 | 1104.48290196585 | 939.610563420504 |
| YDR091C | 9017.22630569818 | 0.00492114996402989 | 0.214829707238019 | 0.0229072134729372 | 0.981724286387205 | 0.985675498685358 | 9679.92056070505 | 10517.3441282075 | 6808.46386366247 | 10300.1525424889 | 8287.9027062244 | 8509.57403290084 |
| YDR092W | 2187.5612391237 | 1.65240618076922 | 0.26585273222995 | 6.21549444652677 | 5.11632831789835e-10 | 1.85699526971957e-09 | 843.648855400644 | 829.624927326581 | 1494.03872256611 | 3250.22293746542 | 3327.71851083252 | 3380.11348115092 |
| YDR093W | 2458.98542142253 | 0.0190061313372644 | 0.0975583925728425 | 0.194818004233448 | 0.845535427004885 | 0.874803442264107 | 2445.06159263412 | 2492.98183607542 | 2389.57964977354 | 2463.34089679878 | 2585.68865421464 | 2377.25989903865 |
| YDR094W | 8.14990165556645 | 0.924325166710444 | 0.681686869354137 | 1.35593805347343 | 0.175118878604216 | 0.224204707190475 | 7.60044013874454 | 4.10705409567614 | 5.14678693797379 | 9.33588861807875 | 11.4158439479675 | 11.293396194958 |
| YDR095C | 11.0476225710347 | 4.36393893829696 | 0.946981090189084 | 4.60826407571202 | 4.06044754933847e-06 | 9.88219992683276e-06 | 0 | 0.821410819135229 | 2.20576583056019 | 18.6717772361575 | 24.2586683894309 | 20.3281131509244 |
| YDR096W | 441.610934376739 | -2.01706402049474 | 0.157445677562755 | -12.8111743156034 | 1.41969618240358e-37 | 4.62721077340182e-36 | 675.67912833439 | 671.914050052617 | 777.164827634042 | 180.049280491519 | 169.810678726016 | 175.047641021849 |
| YDR097C | 728.617015897074 | -0.240314431349186 | 0.210873826504724 | -1.13961241815756 | 0.254447809433729 | 0.312802974220464 | 911.29277263547 | 841.94608961361 | 614.673411449441 | 729.533010584154 | 586.48898282683 | 687.767828272941 |
| YDR098C | 2930.87051808605 | 1.03772901786698 | 0.128625222677447 | 8.06785011730776 | 7.15468017940741e-16 | 4.51412992642378e-15 | 1789.14360866047 | 1864.60255943697 | 2106.50636818499 | 3790.37077893997 | 3808.61093714065 | 4225.98885615328 |
| YDR099W | 3969.58886040623 | -1.51165930875977 | 0.191567454492032 | -7.8910027424447 | 2.99768065907288e-15 | 1.74846733405995e-14 | 5265.58492812222 | 5238.13679362536 | 7130.50567492426 | 2012.55084638298 | 1852.22068055772 | 2318.53423882487 |
| YDR100W | 1694.30336573386 | 2.04602153529383 | 0.136443761229558 | 14.9953469243019 | 7.87501383357547e-51 | 5.2119734412827e-49 | 631.596575529671 | 630.022098276721 | 719.814916039477 | 2486.01376915697 | 2922.45605067968 | 2775.91678472067 |
| YDR101C | 5255.84427702709 | -0.350200216563415 | 0.201466632468391 | -1.73825418270373 | 0.0821660356801782 | 0.1119833804206 | 6145.71589618884 | 6512.1449741041 | 5014.44098814018 | 5528.17976027663 | 3795.76811269919 | 4538.81593075361 |
| YDR102C | 17.0678185619348 | 1.25879150704364 | 0.487688420462802 | 2.58113880548791 | 0.00984749786657936 | 0.0155365777578773 | 9.8805721803679 | 11.4997514678932 | 8.82306332224078 | 29.3413642282475 | 21.404707402439 | 21.4574527704202 |
| YDR103W | 1239.60695998656 | -0.0656652876602297 | 0.11639123808581 | -0.564177241690804 | 0.57263351457421 | 0.632833698335036 | 1263.19315105934 | 1203.36685003311 | 1337.42934859633 | 1197.6611398621 | 1158.7081607187 | 1277.28310964975 |
| YDR104C | 323.163544720633 | -1.29341145076902 | 0.176175457611836 | -7.34160971285099 | 2.11040052304572e-13 | 1.0377602572005e-12 | 441.585572061058 | 497.774956395949 | 438.947400281479 | 185.384073987564 | 164.102756752033 | 211.186508845714 |
| YDR105C | 1794.93283072394 | 0.844624128592362 | 0.102427688051434 | 8.24605284626007 | 1.63712292685581e-16 | 1.09476550993465e-15 | 1285.2344274617 | 1306.04320242501 | 1260.22754452672 | 2332.63845614568 | 2400.18119006016 | 2185.27216372437 |
| YDR106W | 50.27330431527 | 1.01483160442851 | 0.332240290004167 | 3.05451095174454 | 0.00225427783792853 | 0.00390107952422743 | 25.8414964717314 | 34.4992544036796 | 39.7037849500835 | 68.0186170745738 | 55.6522392463415 | 77.92443374521 |
| YDR107C | 476.327123742864 | 1.04981916756177 | 0.222685591140537 | 4.71435606670765 | 2.42476330387772e-06 | 6.07924808231576e-06 | 255.374788661817 | 280.922500144248 | 394.096828393422 | 681.519869119749 | 667.826870956098 | 578.221885181849 |
| YDR108W | 589.667660549868 | -0.249598048262522 | 0.198952354506766 | -1.2545619220306 | 0.20963783371546 | 0.263051071673143 | 558.632350197724 | 639.879028106343 | 723.491192423744 | 618.83604554122 | 443.790933477236 | 553.376413552941 |
| YDR109C | 311.476557923885 | -0.529980553575511 | 0.17722388667696 | -2.99045779614093 | 0.00278559619689859 | 0.00476460362159395 | 357.980730534868 | 367.170636153447 | 380.126978133207 | 241.399405696036 | 224.035937478862 | 298.145659546891 |
| YDR110W | 277.615247518589 | -3.05017332166683 | 0.312381758941044 | -9.76424914184089 | 1.60294960330316e-22 | 1.69604096688814e-21 | 575.353318502962 | 579.094627490337 | 332.335385137736 | 50.6805382124275 | 57.0792197398374 | 71.1483960282353 |
| YDR111C | 933.063745986404 | -0.740874620863838 | 0.224233464602183 | -3.30403234940083 | 0.000953048342471286 | 0.00173053514817154 | 1385.56023729313 | 1253.47291000036 | 863.18969502589 | 714.86232847003 | 747.73777859187 | 633.559526537143 |
| YDR113C | 105.42054118256 | -2.23172139533311 | 0.604016312701694 | -3.69480318395853 | 0.000220057176399535 | 0.000436346941647014 | 253.094656620193 | 224.245153623918 | 44.1153166112039 | 34.6761577242925 | 41.3824343113821 | 35.0095282043697 |
| YDR114C | 9.53701224604277 | 0.877424707446542 | 0.660047587982604 | 1.32933552583434 | 0.183737298622808 | 0.233991776726395 | 8.360484152619 | 8.21410819135229 | 3.67627638426699 | 12.0032853661013 | 11.4158439479675 | 13.5520754339496 |
| YDR115W | 1764.46476351185 | 2.25238157068426 | 0.103425037720048 | 21.7779139397659 | 3.75798091966002e-105 | 4.06237737415248e-102 | 636.156839612918 | 607.022595340934 | 593.351008420692 | 3011.4909285174 | 2845.3991040309 | 2893.36810514823 |
| YDR116C | 1258.0245615794 | 1.32508514513915 | 0.200220027430074 | 6.6181448586702 | 3.63734511165019e-11 | 1.46169890917987e-10 | 674.159040306641 | 599.629897968717 | 879.365311116664 | 1813.8297886553 | 1608.20701616992 | 1972.95631525916 |
| YDR117C | 497.009793695181 | -2.54400338010085 | 0.166329186580257 | -15.2949908095253 | 8.25724795418033e-53 | 6.08596707168336e-51 | 877.850836024995 | 875.623933198154 | 793.340443724817 | 133.369837401125 | 136.99012737561 | 164.883584446386 |
| YDR118W | 309.689385799462 | -0.429605508121127 | 0.18548870099668 | -2.31607373286212 | 0.0205542432124385 | 0.031061235199412 | 383.062182992725 | 364.706403696042 | 318.365534877521 | 305.416927648576 | 248.294605868293 | 238.290659713613 |
| YDR118W-A | 11.2867626859452 | -0.208084923195601 | 0.59132095962436 | -0.351898439939941 | 0.724914429213919 | 0.77179825802388 | 12.9207482358657 | 10.678340648758 | 12.4993397065078 | 12.0032853661013 | 12.8428244414634 | 6.77603771697479 |
| YDR119W | 4748.82153908098 | 0.940905415046975 | 0.130658976926628 | 7.20123054059536 | 5.96715556598031e-13 | 2.82091625371343e-12 | 3232.46719100805 | 3291.39315227486 | 3235.12321815495 | 6977.90989282686 | 5596.61749549106 | 6159.41828473008 |
| YDR119W-A | 342.826541248039 | 2.25743665275305 | 0.350060567380584 | 6.4487030620012 | 1.1281134058037e-10 | 4.32188041939915e-10 | 141.368186580648 | 145.389714986936 | 69.1139960242194 | 502.804287002241 | 742.029856617887 | 456.253206276302 |
| YDR120C | 1318.4543830425 | -3.59381374573574 | 0.219151346081027 | -16.3987755950493 | 1.9513128782386e-60 | 2.22038865407992e-58 | 2494.46445353596 | 2308.16440176999 | 2505.01472823953 | 174.714486995474 | 164.102756752033 | 264.265470962017 |
| YDR121W | 466.117065928192 | -2.87591106031965 | 0.283355283848883 | -10.1494880252645 | 3.33106296776391e-24 | 4.11529036360319e-23 | 925.733608899085 | 919.980117431457 | 616.879177280001 | 105.362171546889 | 84.1918491162602 | 144.555471295462 |
| YDR122W | 698.215441637403 | -0.853210071625507 | 0.182905685812129 | -4.66475423023141 | 3.08986110192303e-06 | 7.64917523170717e-06 | 833.008239206402 | 786.91156473155 | 1076.41372531338 | 468.128129277949 | 522.274860619513 | 502.55613067563 |
| YDR123C | 345.895097020415 | -2.46578701656227 | 0.319412532862836 | -7.71975662464423 | 1.16552034881588e-14 | 6.45014076998276e-14 | 444.625748116556 | 457.525826258323 | 855.101886980502 | 98.6936796768325 | 109.877497999187 | 109.545943091092 |
| YDR124W | 230.930888844154 | 1.15561995759048 | 0.431562441238299 | 2.67775841260565 | 0.00741166555023257 | 0.0119641768936806 | 98.805721803679 | 97.7478874770923 | 232.340667485674 | 258.737484558183 | 449.49885545122 | 248.454716289076 |
| YDR125C | 194.479859891683 | -0.00403247614159874 | 0.196268505591215 | -0.0205457117506032 | 0.983608047057263 | 0.986955722959995 | 171.769947135627 | 191.388720858508 | 220.57658305602 | 200.054756101688 | 206.912171556911 | 176.176980641344 |
| YDR126W | 453.207588424936 | 0.270907509717694 | 0.199434456004737 | 1.35837866306943 | 0.17434356114281 | 0.223388450725458 | 444.625748116556 | 420.562339397237 | 366.892383149846 | 410.779099195465 | 590.769924307318 | 485.616036383193 |
| YDR127W | 9840.42585493094 | 0.399304538754158 | 0.128540536195186 | 3.10644836697914 | 0.00189349372684608 | 0.00331565883162086 | 7795.77145031028 | 8306.10620309544 | 9359.06441906691 | 11329.7676872256 | 11756.892285913 | 10494.9530839744 |
| YDR128W | 906.488056721236 | -0.368539045729751 | 0.116972826943204 | -3.15063810425556 | 0.00162914215106466 | 0.00287370573614506 | 1037.46007893863 | 992.264269515357 | 1035.97468508644 | 752.205882942345 | 776.277388461789 | 844.746035382857 |
| YDR129C | 8978.6078074749 | 0.841255643081792 | 0.163376090238819 | 5.14919681241035 | 2.61604268645425e-07 | 7.21107219054069e-07 | 5823.45723430607 | 5993.83474722977 | 7479.75193142962 | 10853.6373677036 | 12795.7340851781 | 10925.2314790023 |
| YDR130C | 98.3243519243754 | -2.56706281868726 | 0.495160477349512 | -5.18430475798108 | 2.16822144523833e-07 | 6.03306919511619e-07 | 221.172808037466 | 207.816937241213 | 75.7312935159 | 38.6772528463263 | 22.831687895935 | 23.7161320094118 |
| YDR131C | 359.841843722682 | -0.849699651683692 | 0.190683809699833 | -4.45606605522124 | 8.34773204606436e-06 | 1.9767575776113e-05 | 428.664823825192 | 436.169144960807 | 522.766501842766 | 241.399405696036 | 299.665903634147 | 230.385282377143 |
| YDR132C | 395.604573724328 | -0.113064407886751 | 0.132644621613049 | -0.852385920452792 | 0.393999932034528 | 0.459371482864632 | 408.143635450582 | 421.383750216373 | 403.655146992516 | 361.432259357049 | 399.554538178862 | 379.458112150588 |
| YDR133C | 22888.347677932 | 1.81445458518754 | 0.328285387769604 | 5.5270647210803 | 3.25632858564796e-08 | 9.77803111412625e-08 | 7210.53755962695 | 7413.23264269544 | 15777.1077307202 | 32666.2742746576 | 36676.2526438325 | 37586.6812160591 |
| YDR135C | 3976.08068371649 | 0.206810970926763 | 0.123655615155732 | 1.67247537175166 | 0.0944306203706832 | 0.127096286309245 | 3423.23823849054 | 3559.17307931295 | 4092.43087096601 | 4269.16849521001 | 4139.67041163171 | 4372.80300668773 |
| YDR136C | 12.8930030830438 | 2.62682158643531 | 0.620297498768644 | 4.23477700885434 | 2.28778613006458e-05 | 5.11146429197344e-05 | 4.56026408324673 | 3.28564327654092 | 2.94102110741359 | 24.0065707322025 | 19.9777269089431 | 22.586792389916 |
| YDR137W | 529.788054469449 | -1.7692313485524 | 0.244549078019783 | -7.23466783387046 | 4.66669903275075e-13 | 2.22724134852254e-12 | 918.133168760341 | 953.657961016001 | 585.998455652158 | 261.404881306205 | 226.889898465854 | 232.643961616134 |
| YDR138W | 306.410652013981 | -1.70517809427962 | 0.280513442605144 | -6.07877497222071 | 1.2110416886957e-09 | 4.22301956606468e-09 | 544.951557947984 | 494.489313119408 | 367.627638426699 | 189.385169109598 | 109.877497999187 | 132.132735481008 |
| YDR139C | 1520.77646930001 | 1.47215457966307 | 0.200314465659184 | 7.3492175156626 | 1.99370520909285e-13 | 9.84107457091036e-13 | 883.93118813599 | 907.658955144428 | 626.437495879095 | 2407.32556509031 | 1992.06476892033 | 2307.24084262992 |
| YDR140W | 1178.16626455519 | 0.817552919137627 | 0.131599952910729 | 6.21241042306615 | 5.21779262832926e-10 | 1.89064821158344e-09 | 776.004938165818 | 838.660446337069 | 944.067775479763 | 1475.07040165644 | 1535.43101100163 | 1499.76301469042 |
| YDR141C | 1973.17294572103 | 0.020111145541096 | 0.142274424985832 | 0.141354607780694 | 0.887589811871927 | 0.910183007083213 | 1800.54426886858 | 1933.60106824433 | 2143.26913202766 | 2141.91958866207 | 2026.31230076423 | 1793.39131575933 |
| YDR142C | 962.334299797965 | 0.45787237894731 | 0.156059114556833 | 2.93396755612478 | 0.00334659187830368 | 0.00566440368545868 | 854.289471594887 | 804.161191933389 | 773.488551249775 | 1032.28254148471 | 1289.99036612033 | 1019.79367640471 |
| YDR143C | 1019.56666784585 | -1.4705910330394 | 0.136200381937598 | -10.7972607133596 | 3.54625849300703e-27 | 5.29977709346627e-26 | 1475.24543093032 | 1391.46992761508 | 1629.32569350713 | 548.150031718624 | 505.151094697561 | 568.057828606386 |
| YDR144C | 5003.35534039078 | 0.0797763267164953 | 0.139254967189066 | 0.572879577129794 | 0.566726233522793 | 0.627374355799426 | 4862.00155675488 | 5454.98924987706 | 4278.45045600992 | 5330.79240092297 | 5067.20773240407 | 5026.6906463758 |
| YDR145W | 347.246651736627 | -1.66870259881723 | 0.424060906752918 | -3.93505407417692 | 8.31779666038496e-05 | 0.000172803424533174 | 675.67912833439 | 684.235212339646 | 224.98811471714 | 189.385169109598 | 161.248795765041 | 147.94349015395 |
| YDR146C | 186.978219224345 | -3.6989837487549 | 0.496856568364282 | -7.44477175964977 | 9.71121675569427e-14 | 4.95570038374768e-13 | 487.188212893525 | 416.455285301561 | 138.227992048439 | 26.673967480225 | 22.831687895935 | 30.4921697263865 |
| YDR147W | 377.568752205215 | -0.283991908960359 | 0.157476387787305 | -1.8033935941173 | 0.071326422947215 | 0.0982214817910056 | 420.304339672573 | 441.919020694753 | 381.597488686914 | 376.102941471173 | 322.497591530082 | 322.991131175798 |
| YDR148C | 3074.37235003225 | 0.871856047730396 | 0.170315282392796 | 5.11907114547506 | 3.07044124829649e-07 | 8.40645079630689e-07 | 1958.63342375447 | 1931.95824660606 | 2627.06710419719 | 3963.75156756144 | 4015.52310869756 | 3949.3006493768 |
| YDR150W | 681.145405109791 | -3.60702296820879 | 0.489581553885893 | -7.3675630537532 | 1.73775252217231e-13 | 8.60386477771724e-13 | 1788.38356464659 | 1575.46595110137 | 413.21346559161 | 102.694774798866 | 94.1807125707318 | 112.93396194958 |
| YDR151C | 751.517402420144 | -1.51429451833272 | 0.133870722081412 | -11.3116183642591 | 1.14949497832503e-29 | 2.03705585503173e-28 | 1149.18654897817 | 1136.01116286402 | 1054.35606700777 | 360.098560983038 | 420.959245581301 | 388.492829106554 |
| YDR152W | 625.822304515925 | -3.63491828357273 | 0.317216507036493 | -11.458792978748 | 2.12454934800313e-30 | 3.97113172079201e-29 | 1457.00437459733 | 1301.1147375102 | 717.609150208917 | 88.0240926847425 | 82.7648686227643 | 108.416603471597 |
| YDR153C | 515.12583267302 | -1.9822830539124 | 0.551339331641865 | -3.59539568492102 | 0.00032389889755383 | 0.000628420056695825 | 1185.66866164415 | 1071.94111897147 | 208.812498626365 | 184.050375613553 | 248.294605868293 | 191.987735314286 |
| YDR154C | 3668.77469057362 | 2.64101991321821 | 0.329744791359485 | 8.00928470266262 | 1.15377530748163e-15 | 7.12703489935798e-15 | 725.842033250104 | 727.769985753813 | 1587.41614272649 | 6361.74124403366 | 6365.75998148537 | 6244.11875619227 |
| YDR155C | 17784.5879048381 | 2.53974213737337 | 0.339660780783914 | 7.47728993471611 | 7.58709058166738e-14 | 3.91175433328256e-13 | 3680.13311518011 | 3711.13408085296 | 8266.47507766276 | 28273.0718306645 | 32860.5068042244 | 29916.2065204437 |
| YDR156W | 3000.30276644738 | 1.41669630306974 | 0.164688631683373 | 8.60227138078026 | 7.81537257233812e-18 | 5.87375509897857e-17 | 1519.32798373503 | 1634.60753007911 | 1752.11332474165 | 4013.09840739985 | 3944.17408402277 | 5138.49526870588 |
| YDR157W | 176.452079624116 | 1.25907808212296 | 0.276386884709607 | 4.55549142082425 | 5.22632488192943e-06 | 1.26249322846161e-05 | 123.127130247662 | 119.925979593743 | 69.1139960242194 | 257.403786184171 | 249.721586361789 | 239.419999333109 |
| YDR158W | 29887.205261991 | 0.923639030475188 | 0.123114243107587 | 7.5022922381778 | 6.27113642815835e-14 | 3.26179557923297e-13 | 19022.3815792498 | 19843.6425686689 | 23035.547823817 | 39650.8526593545 | 38848.1169549334 | 38922.6899859227 |
| YDR159W | 394.23127386653 | -2.17158059104677 | 0.248620070146665 | -8.73453454407649 | 2.44661483425415e-18 | 1.90501126230161e-17 | 760.044013874454 | 709.698947732838 | 466.151845525055 | 149.37421788926 | 135.563146882114 | 144.555471295462 |
| YDR160W | 709.468226529884 | -0.0779017169064773 | 0.117415072072558 | -0.663472887521095 | 0.507027738212621 | 0.570096468085714 | 702.280668819996 | 723.662931658137 | 759.51870098956 | 717.529725218053 | 686.377617371545 | 667.439715122017 |
| YDR161W | 1323.88534249393 | -1.85739531598754 | 0.201662812866935 | -9.21040071583805 | 3.24909707041085e-20 | 2.92283545058041e-19 | 2297.61305394248 | 2191.52406545279 | 1737.40821920458 | 594.829474809018 | 468.049601866667 | 653.887639688067 |
| YDR162C | 335.040889073633 | -1.93345266502607 | 0.191767772869742 | -10.0822606222755 | 6.61855598132242e-24 | 7.92028673336849e-23 | 568.512922378092 | 459.990058715728 | 565.411307900263 | 136.037234149148 | 125.574283427642 | 154.719527870924 |
| YDR163W | 64.6572287585721 | -1.93977724864222 | 0.413865571746548 | -4.68697417969848 | 2.7727393287689e-06 | 6.90099281903113e-06 | 123.887174261536 | 122.390212051149 | 61.7614432556855 | 28.0076658542363 | 21.404707402439 | 30.4921697263865 |
| YDR164C | 682.446970292731 | -2.07965531073601 | 0.16930221449679 | -12.2836864060951 | 1.10830615088077e-34 | 2.80799753695808e-33 | 1076.22232364623 | 1091.65497863072 | 1144.79246606074 | 281.410356916374 | 212.620093530894 | 287.981602971428 |
| YDR165W | 1937.5655041453 | -1.39308033350302 | 0.204308424207359 | -6.81851636273763 | 9.19855008936997e-12 | 3.8943730991941e-11 | 3073.61799210829 | 3088.50467994846 | 2257.96895521679 | 1224.33510734233 | 904.705632876423 | 1076.2606573795 |
| YDR166C | 705.027353457038 | -1.24145623039554 | 0.168325243105501 | -7.37534197183625 | 1.63923735424803e-13 | 8.15344591997909e-13 | 1080.0225437156 | 1039.9060970252 | 852.160865873089 | 421.448686187555 | 453.779796931708 | 382.846131009075 |
| YDR167W | 1395.44917921669 | -0.0795157864395391 | 0.190109848819511 | -0.418262320091743 | 0.675755335612219 | 0.727943714795026 | 1561.13040449813 | 1491.68204754958 | 1249.93397065078 | 1381.71151547566 | 1117.32572640732 | 1570.91141071865 |
| YDR168W | 940.745043409179 | -1.73505391272459 | 0.2636863589676 | -6.57999116646677 | 4.70476219621908e-11 | 1.86636621435333e-10 | 1666.0164784128 | 1679.78512513154 | 995.535644859501 | 432.118273179645 | 369.587947815447 | 501.426791056134 |
| YDR169C | 215.691807117304 | -2.25158853345655 | 0.422538857738693 | -5.32871354248082 | 9.89108558763429e-08 | 2.85634822446109e-07 | 476.547596699283 | 430.41926922686 | 162.491416184601 | 69.352315448585 | 84.1918491162602 | 71.1483960282353 |
| YDR169C-A | 1.7046045250434 | -0.699842658111252 | 1.5084901665655 | -0.463935843681794 | 0.642693711452833 | 0.697892417961339 | 0.760044013874454 | 4.10705409567614 | 1.4705105537068 | 1.33369837401125 | 1.42698049349594 | 1.1293396194958 |
| YDR170C | 2992.57251201019 | -0.637241758639309 | 0.120500842208637 | -5.288276388442 | 1.23474311980029e-07 | 3.52954776334273e-07 | 3748.53707642881 | 3895.95151515839 | 3284.38532170413 | 2332.63845614568 | 2378.77648265772 | 2315.14621996639 |
| YDR170W-A | 37.5219416181608 | -3.77530232030276 | 0.518725529840423 | -7.27803453488046 | 3.38718636307721e-13 | 1.64435029654399e-12 | 71.4441373041987 | 73.9269737221706 | 64.702464363099 | 4.00109512203375 | 4.28094148048781 | 6.77603771697479 |
| YDR171W | 435.592784387147 | -1.72484358495294 | 0.28848386653581 | -5.97899496309903 | 2.24518498998149e-09 | 7.62422504974868e-09 | 892.291672288609 | 646.450314659425 | 468.357611355615 | 192.05256585762 | 178.372561686992 | 236.031980474622 |
| YDR172W | 3895.030729513 | -1.89485494605453 | 0.404080800130462 | -4.68929715404134 | 2.74145040018534e-06 | 6.83098244164506e-06 | 7833.01360699013 | 7902.79349090004 | 2682.2112499612 | 1803.16020166321 | 1301.40621006829 | 1847.59961749513 |
| YDR173C | 176.562120597323 | 0.731673972805852 | 0.235621403558555 | 3.10529502734254 | 0.00190089318423424 | 0.00332681953398362 | 162.649418969133 | 120.747390412879 | 114.69982318913 | 253.402691062138 | 201.204249582927 | 206.669150367731 |
| YDR174W | 4273.46312921269 | -1.27311461897225 | 0.218327918397475 | -5.83120394458438 | 5.50288767937516e-09 | 1.79535862617844e-08 | 6080.35211099563 | 5870.62312435948 | 6187.17315472135 | 2208.60450736263 | 2023.45833977724 | 3270.56753805983 |
| YDR175C | 700.939816231359 | 0.270657547976199 | 0.146660171456578 | 1.84547410035133 | 0.0649686026631924 | 0.0898669986934242 | 614.115563210559 | 634.129152372397 | 658.788728060645 | 782.880945544604 | 672.107812436586 | 843.616695763361 |
| YDR176W | 33.1215730761227 | -2.65891355730145 | 0.684985496112142 | -3.88170782066624 | 0.000103725456023402 | 0.000213169615896004 | 87.4050615955622 | 65.7128655308183 | 18.381381921335 | 9.33588861807875 | 9.98886345447155 | 7.90537733647059 |
| YDR177W | 1389.87298827118 | -0.645581486538425 | 0.294962519433132 | -2.18868989788643 | 0.0286193867294805 | 0.0422644222056945 | 1598.37256117798 | 1424.32636038049 | 2065.3320726812 | 1182.99045774798 | 694.939500332521 | 1373.27697730689 |
| YDR178W | 2843.81233305795 | 1.47517678124779 | 0.157806435943147 | 9.34801405551832 | 8.93088060274632e-21 | 8.27509879848752e-20 | 1412.16177777874 | 1485.11076099649 | 1616.09109852377 | 3675.67271877501 | 4821.76708752277 | 4052.07055475092 |
| YDR179C | 2.7552089873655 | -1.62747243606069 | 1.32582630306254 | -1.22751557447712 | 0.219628871738722 | 0.274419738412127 | 4.56026408324673 | 4.10705409567614 | 3.67627638426699 | 1.33369837401125 | 2.85396098699187 | 0 |
| YDR179W-A | 879.416453204628 | 1.04974936836022 | 0.169352491708727 | 6.19860598310954 | 5.69654410453708e-10 | 2.05722633975654e-09 | 563.192614280971 | 520.774459331735 | 634.525303924483 | 1349.70275449939 | 1041.69576025203 | 1166.60782693916 |
| YDR180W | 632.833505311868 | -1.11427053601183 | 0.195876530617491 | -5.6886372884957 | 1.28057137709026e-08 | 4.016337500874e-08 | 966.775985648306 | 899.444846953076 | 730.843745192278 | 468.128129277949 | 368.160967321951 | 363.647357477647 |
| YDR181C | 211.793318920934 | -1.46621203145395 | 0.413114105532016 | -3.54916961638417 | 0.000386448068266302 | 0.000741130151027568 | 404.34341538121 | 387.705906631828 | 141.169013155852 | 113.364361790956 | 105.596556518699 | 118.580660047059 |
| YDR182W | 1736.81370856703 | 0.255037538326719 | 0.148336248835954 | 1.71932039759726 | 0.0855560478203691 | 0.11601850850155 | 1440.28340629209 | 1488.39640427304 | 1821.96257604272 | 1828.50047076942 | 1974.94100299838 | 1866.79839102655 |
| YDR182W-A | 69.2875049870566 | 1.00032634442703 | 0.292438308821048 | 3.42064057359584 | 0.000624738392102834 | 0.00115971757618173 | 34.2019806243504 | 48.4632383289785 | 55.8794010408583 | 90.691489432765 | 92.7537320772358 | 93.7351884181512 |
| YDR183C-A | 140.519547509496 | -0.545740913715366 | 0.271873569639763 | -2.00733346179433 | 0.0447141684804448 | 0.0636978029352438 | 174.05007917725 | 157.710877273964 | 169.108713676282 | 138.70463089717 | 77.0569466487805 | 126.486037383529 |
| YDR183W | 234.973593678059 | -1.6931953170649 | 0.290759944288889 | -5.82334448166835 | 5.76814893295642e-09 | 1.87906649819967e-08 | 444.625748116556 | 393.455782365775 | 238.957964977354 | 108.029568294911 | 108.450517505691 | 116.321980808067 |
| YDR184C | 474.081477650384 | -3.48830347149088 | 0.323124368535166 | -10.7955444131452 | 3.61314766656998e-27 | 5.38732776215469e-26 | 1042.78038703575 | 1009.5138967172 | 560.26452096229 | 72.0197121966075 | 62.7871417138212 | 97.1232072766386 |
| YDR185C | 440.637878509436 | 1.40006320779801 | 0.195732553944062 | 7.15293996622624 | 8.49387581676435e-13 | 3.95486565308927e-12 | 228.013204162336 | 237.387726730081 | 261.015623282956 | 513.473873994331 | 726.333071189431 | 677.603771697479 |
| YDR186C | 374.510009375113 | -4.46315085973171 | 0.547425623347379 | -8.15298128070914 | 3.55060589181477e-16 | 2.29832632877351e-15 | 1050.3808271745 | 883.838041389506 | 215.429796118046 | 38.6772528463263 | 27.1126293764228 | 31.6215093458823 |
| YDR188W | 6111.63200699918 | 0.538789826127482 | 0.125087112795519 | 4.30731682973806 | 1.65246835892512e-05 | 3.761990093362e-05 | 4895.44349336536 | 4811.00316767504 | 5244.57588979529 | 7614.08401723023 | 6482.77238195204 | 7621.91309197714 |
| YDR189W | 917.064315639069 | -2.57372673809653 | 0.228231150700386 | -11.2768424914758 | 1.70765659048812e-29 | 2.97738189406074e-28 | 1516.28780767954 | 1593.53698912234 | 1602.85650354041 | 280.076658542363 | 185.507464154472 | 324.120470795294 |
| YDR190C | 2697.2038534818 | -1.18816859362658 | 0.226174634799833 | -5.25332380741159 | 1.49378573444956e-07 | 4.23456917554188e-07 | 4187.84251644824 | 3944.41475348737 | 3116.0118633047 | 1829.83416914344 | 1237.19208786098 | 1867.92773064605 |
| YDR191W | 251.33497043353 | 0.0846228521281455 | 0.264062684226669 | 0.320465015251856 | 0.748615846841203 | 0.792091742677331 | 274.375889008678 | 289.958019154736 | 167.638203122575 | 276.075563420329 | 258.283469322764 | 241.678678572101 |
| YDR192C | 379.99552375046 | -1.45827938135805 | 0.262116784910428 | -5.56347195337521 | 2.64459383743348e-08 | 8.00785977105206e-08 | 532.030809712118 | 515.845994416924 | 624.966985325389 | 202.72215284971 | 141.271068856098 | 263.136131342521 |
| YDR193W | 2.02343417612551 | 0.705288585892692 | 1.31185122432773 | 0.537628484704218 | 0.590833560887696 | 0.649406282988916 | 2.28013204162336 | 1.64282163827046 | 0.735255276853398 | 2.6673967480225 | 1.42698049349594 | 3.38801885848739 |
| YDR194C | 956.639722383473 | -1.23762136344945 | 0.246082756335079 | -5.02928925976528 | 4.92301242434937e-07 | 1.32163321955008e-06 | 1290.55473555882 | 1085.08369207764 | 1655.79488347385 | 574.823999198849 | 448.071874957724 | 685.509149033949 |
| YDR195W | 213.137822101907 | -2.78853603713677 | 0.368641500529824 | -7.56435733125271 | 3.89786878950536e-14 | 2.06380220152913e-13 | 487.188212893525 | 404.134123014533 | 226.458625270847 | 49.3468398384163 | 42.8094148048781 | 68.8897167892437 |
| YDR196C | 584.983323859876 | -0.668677001257358 | 0.228692571233347 | -2.92391220952722 | 0.00345662219438282 | 0.00583237553401846 | 839.848635331272 | 794.304262103767 | 520.560736012206 | 466.794430903938 | 436.656031009756 | 451.735847798319 |
| YDR197W | 697.954477118975 | 0.700158627150277 | 0.144230215966071 | 4.85445176976637 | 1.20720245218891e-06 | 3.11204892881451e-06 | 521.390193517876 | 482.989561651515 | 590.409987313279 | 866.903943107313 | 906.132613369919 | 819.900563753949 |
| YDR198C | 530.228837041503 | -0.899841226485138 | 0.138936649592708 | -6.47662966627609 | 9.37940171024838e-11 | 3.62759686897263e-10 | 721.281769166857 | 699.02060708408 | 651.436175292111 | 394.77471870733 | 341.048337945529 | 373.811414053109 |
| YDR199W | 2.0591019784908 | 3.80599790359663 | 2.25773171362867 | 1.68576181156598 | 0.0918416859778202 | 0.123880381046452 | 0.760044013874454 | 0 | 0 | 9.33588861807875 | 0 | 2.2586792389916 |
| YDR200C | 33.1789224149801 | -1.91115032870669 | 0.601485428758874 | -3.17738425126977 | 0.00148609997176458 | 0.00263500394118783 | 73.724269345822 | 62.4272222542774 | 21.3224030287486 | 14.6706821141238 | 9.98886345447155 | 16.940094292437 |
| YDR201W | 283.583253952142 | -0.176203318615904 | 0.235001097956117 | -0.749797852641553 | 0.453376462255412 | 0.518076063105815 | 342.779850257379 | 330.207149292362 | 229.39964637826 | 294.747340656486 | 273.98025475122 | 230.385282377143 |
| YDR202C | 323.135163336607 | -3.11618849290794 | 0.206265439038894 | -15.1076617945692 | 1.44165185913237e-51 | 1.00543590949812e-49 | 589.794154766576 | 570.059108479849 | 579.381158160478 | 77.3545056926525 | 49.9443172723578 | 72.2777356477311 |
| YDR203W | 1.51499441494385 | 0.387613387746896 | 1.70653824743938 | 0.227134310249711 | 0.820319305450324 | 0.853616399029488 | 0.760044013874454 | 2.46423245740569 | 0.735255276853398 | 4.00109512203375 | 0 | 1.1293396194958 |
| YDR204W | 1053.3702689932 | 1.53125372831431 | 0.283778224985274 | 5.39595216790779 | 6.81611046640315e-08 | 1.99861177599868e-07 | 441.585572061058 | 416.455285301561 | 766.135998481241 | 1375.0430236056 | 1806.55730476585 | 1514.44442974386 |
| YDR205W | 1072.61617175375 | 0.561070620727708 | 0.119265521193744 | 4.70438241590596 | 2.54635568975337e-06 | 6.36343204291098e-06 | 829.20801913703 | 900.266257772211 | 869.80699251757 | 1299.02221628696 | 1347.06958586016 | 1190.32395894857 |
| YDR206W | 1920.36915622775 | -0.234601453640908 | 0.185582655501689 | -1.2641345873983 | 0.206181724159011 | 0.259266123089443 | 1967.75395192096 | 1909.78015448941 | 2351.34637537717 | 2007.21605288693 | 1449.81218139187 | 1836.30622130017 |
| YDR207C | 135.579558925833 | -4.01895428416879 | 0.673662907554266 | -5.96582391445538 | 2.43402666251674e-09 | 8.21389018370633e-09 | 380.022006937227 | 331.849970930633 | 54.4088904871515 | 12.0032853661013 | 17.1237659219512 | 18.0694339119328 |
| YDR208W | 1010.32680745607 | -0.742975156845935 | 0.133564794712859 | -5.56265712415612 | 2.65697616121005e-08 | 8.04159933812803e-08 | 1293.59491161432 | 1259.22278573431 | 1241.84616260539 | 844.231070749121 | 693.512519839025 | 729.553394194285 |
| YDR209C | 2.10637657420676 | 1.64750572505726 | 1.6344099278725 | 1.00801255362038 | 0.313448435653653 | 0.376905182359954 | 2.28013204162336 | 0.821410819135229 | 0 | 1.33369837401125 | 1.42698049349594 | 6.77603771697479 |
| YDR210C-D | 5.62065303524183 | -5.56346238181078 | 1.59608420409798 | -3.48569478197107 | 0.000490860717934367 | 0.000926578177101952 | 14.4408362636146 | 15.6068055635694 | 3.67627638426699 | 0 | 0 | 0 |
| YDR210W | 1495.79888025306 | 0.225040244899419 | 0.133439053343621 | 1.68646463880341 | 0.0917063404610365 | 0.1237377416747 | 1494.24653127718 | 1291.25780768058 | 1352.1344541334 | 1756.48075857282 | 1563.97062087155 | 1516.70310898286 |
| YDR210W-B | 3.58211160709671 | -2.80628539153188 | 1.87003318913744 | -1.50066074112101 | 0.133443332136406 | 0.17421768362253 | 12.1607042219913 | 6.57128655308183 | 0 | 1.33369837401125 | 1.42698049349594 | 0 |
| YDR211W | 2144.73131343219 | -0.93456193564879 | 0.243579247456785 | -3.83678800803667 | 0.000124653987548187 | 0.000254007465673121 | 3123.78089702401 | 2959.54318134423 | 2366.05148091424 | 1576.4314780813 | 1055.96556518699 | 1786.61527804235 |
| YDR212W | 8971.37343634826 | 0.802844101752963 | 0.0955576023020866 | 8.40167691959168 | 4.40147416565735e-17 | 3.08293320069693e-16 | 6376.0092323928 | 6701.0694625052 | 6535.68415594986 | 12033.9604287035 | 11148.9985956837 | 11032.5187428545 |
| YDR213W | 602.993079710005 | -0.467300772355342 | 0.28161013854651 | -1.65938902188411 | 0.0970374270168033 | 0.130375647230584 | 839.848635331272 | 806.625424390795 | 452.917250541693 | 445.455256919758 | 575.073138878862 | 498.038772197647 |
| YDR214W | 4835.62990078271 | 1.5069681622993 | 0.486241707718819 | 3.09921616837267 | 0.00194033392518146 | 0.00339127077303341 | 1361.23882884915 | 1331.50693781821 | 4858.56686944726 | 6875.215118028 | 6803.84299298862 | 7783.40865756504 |
| YDR215C | 19.8675150356295 | 0.998267328716252 | 0.810005391804911 | 1.23242059721583 | 0.217792010724672 | 0.2723869999152 | 6.84039612487009 | 4.10705409567614 | 28.6749557972825 | 17.3380788621463 | 38.5284733243903 | 23.7161320094118 |
| YDR216W | 720.848671016095 | -0.59357245952442 | 0.29210368165385 | -2.03206086333351 | 0.0421474947825909 | 0.060266457486747 | 620.955959335429 | 789.375797188955 | 1190.37829322565 | 521.476064238399 | 699.220441813009 | 503.685470295126 |
| YDR217C | 288.351016723906 | -0.817646514443734 | 0.292676758354786 | -2.79368446965158 | 0.00521112977287006 | 0.00856765214368447 | 440.825528047183 | 428.77644758859 | 234.546433316234 | 213.3917398418 | 191.215386128455 | 221.350565421176 |
| YDR218C | 13.7612531183717 | -2.27662298049486 | 0.626939716896438 | -3.63132677534756 | 0.000281967889061352 | 0.000552353889595871 | 25.8414964717314 | 20.5352704783807 | 22.057658305602 | 5.334793496045 | 4.28094148048781 | 4.51735847798319 |
| YDR219C | 209.83828418279 | -3.27551199925901 | 0.388865444143336 | -8.42325294929434 | 3.66172736376203e-17 | 2.58996332403059e-16 | 465.90698050504 | 449.31171806697 | 225.723369993993 | 53.34793496045 | 34.2475318439025 | 30.4921697263865 |
| YDR220C | 1.43412615162724 | -1.2947890297528 | 2.15746567508494 | -0.600143513153144 | 0.548410595391286 | 0.610537439359403 | 5.32030809712118 | 0.821410819135229 | 0 | 1.33369837401125 | 0 | 1.1293396194958 |
| YDR221W | 523.68529789476 | -1.06335972317471 | 0.157833789486058 | -6.73721214346592 | 1.61454371310323e-11 | 6.66577372577182e-11 | 652.117763904282 | 680.949569063105 | 792.605188447963 | 346.761577242925 | 313.935708569106 | 355.741980141176 |
| YDR222W | 1614.50944662153 | -0.79028774790406 | 0.136987073243549 | -5.76906805285933 | 7.97111127065343e-09 | 2.54934061644271e-08 | 2149.40447123696 | 1991.92123640293 | 1995.48282138012 | 1303.02331140899 | 1195.80965354959 | 1051.41518575059 |
| YDR223W | 37.0314247855004 | -0.0395888280863834 | 0.333449535529118 | -0.118725095908632 | 0.905493153507929 | 0.924449644837467 | 35.7220686520993 | 41.8919517758967 | 35.2922532889631 | 34.6761577242925 | 32.8205513504065 | 41.7855659213445 |
| YDR224C | 17051.4926527862 | 0.0739570146906793 | 0.176713691187183 | 0.41851321306135 | 0.675571928027364 | 0.727867030761708 | 15799.7949604222 | 13898.2710597681 | 20145.2593305063 | 17615.4881239406 | 17200.8228686 | 17649.3195734803 |
| YDR225W | 47378.1492183809 | 1.6852720498747 | 0.0867298434942469 | 19.4312820359983 | 4.19746286487064e-84 | 1.94462458153936e-81 | 23270.2675727942 | 22003.9530229945 | 22151.7709810392 | 70056.508190063 | 73378.190936548 | 73408.2046068464 |
| YDR226W | 15279.5344977288 | 1.05212926673075 | 0.119311718893494 | 8.8183229316305 | 1.1618675787392e-18 | 9.36133306298438e-18 | 9524.87158187466 | 9865.96534863324 | 10436.948654934 | 18826.4862475428 | 20811.0835171447 | 22211.8516362434 |
| YDR227W | 275.24554191151 | -2.04022555476995 | 0.232052234834777 | -8.7920961253513 | 1.4679508850962e-18 | 1.17399869799432e-17 | 488.708300921274 | 494.489313119408 | 345.569980121097 | 114.698060164968 | 98.4616540512196 | 109.545943091092 |
| YDR228C | 306.793119514182 | -1.42116703927551 | 0.452743356245822 | -3.13901246626769 | 0.00169518235995473 | 0.00298046971717712 | 610.315343141187 | 552.809481278009 | 177.196521721669 | 202.72215284971 | 144.12502984309 | 153.590188251429 |
| YDR229W | 125.18059869776 | -3.7199908861241 | 0.544223594776478 | -6.83540905214146 | 8.1771362956925e-12 | 3.48468502062165e-11 | 339.739674201881 | 244.780424102298 | 113.964567912277 | 24.0065707322025 | 7.13490246747968 | 21.4574527704202 |
| YDR231C | 501.08979670707 | 1.30488636228961 | 0.155825664197162 | 8.37401444115499 | 5.5687930528061e-17 | 3.88378405811832e-16 | 307.05778160528 | 262.851462123273 | 296.30787657192 | 638.841521151389 | 739.175895630895 | 762.304243159664 |
| YDR232W | 6363.88307931414 | 0.977597822168513 | 0.143256691789838 | 6.82409882536362 | 8.84789599661465e-12 | 3.75326706566662e-11 | 3807.82050951102 | 4171.94555038783 | 4879.889272476 | 8030.19790992174 | 8700.30006884472 | 8593.14516474353 |
| YDR233C | 30845.3616635929 | 0.738679443208114 | 0.100934252888164 | 7.31842186444455 | 2.50904041400402e-13 | 1.22727270929337e-12 | 22880.3649936766 | 24484.6136967829 | 21985.6032884703 | 37614.2952422393 | 40185.197677339 | 37922.0950830494 |
| YDR234W | 6654.92376048479 | 0.478119556495548 | 0.113776490263452 | 4.20227021758581 | 2.64251429375871e-05 | 5.8636153641187e-05 | 5205.54145102614 | 5558.4870130881 | 5921.74599977727 | 8092.88173350027 | 7865.5164801496 | 7285.36988536739 |
| YDR235W | 669.346562479543 | -0.299728557789296 | 0.182567135990805 | -1.64174431593423 | 0.100643003405393 | 0.134786396879492 | 803.366522665298 | 786.91156473155 | 624.966985325389 | 629.50563253331 | 650.703105034147 | 520.625564587563 |
| YDR236C | 1101.56245954326 | 1.54434493928357 | 0.127415248766604 | 12.1205660565202 | 8.21895038195161e-34 | 1.9743745250866e-32 | 514.549797393006 | 583.201681586013 | 589.674732036425 | 1557.75970084514 | 1726.64639713008 | 1637.54244826891 |
| YDR237W | 1701.68760190625 | 1.50715950953057 | 0.273346855845804 | 5.51372542723068 | 3.51316545236502e-08 | 1.05297556026061e-07 | 747.123265638588 | 706.413304456297 | 1203.61288820901 | 2460.67350005076 | 2063.41379359512 | 3028.88885948773 |
| YDR238C | 5921.30182194284 | 0.135248996089393 | 0.127379279145829 | 1.06178176699017 | 0.28833476898484 | 0.3500167156346 | 5117.3763454167 | 5511.66659639739 | 6302.60823318733 | 6157.68539280994 | 6231.62381509675 | 6206.8505487489 |
| YDR239C | 327.236874844121 | -1.9688019527342 | 0.328305161097737 | -5.99686567872165 | 2.01162315598965e-09 | 6.8742822917539e-09 | 674.159040306641 | 578.273216671201 | 311.748237385841 | 116.031758538979 | 134.136166388618 | 149.072829773445 |
| YDR240C | 347.211864154364 | -0.64674313989926 | 0.150617916757986 | -4.29393231443011 | 1.75536009297032e-05 | 3.97722481764961e-05 | 399.023107284088 | 434.526323322536 | 437.476889727772 | 294.747340656486 | 261.137430309756 | 256.360093625546 |
| YDR241W | 62.1381782868854 | 1.47530170554338 | 0.325490987504163 | 4.5325424118679 | 5.82779701172762e-06 | 1.39841255708714e-05 | 36.4821126659738 | 38.6063084993558 | 23.5281688593087 | 90.691489432765 | 105.596556518699 | 77.92443374521 |
| YDR242W | 777.150383427944 | 0.0396939350462526 | 0.177413130305266 | 0.223737301618843 | 0.822961719123422 | 0.855453744005618 | 762.324145916078 | 676.842514967429 | 859.513418641623 | 702.859043103929 | 904.705632876423 | 756.657545062185 |
| YDR243C | 258.53971115792 | -1.45175971299345 | 0.227897014318611 | -6.37024454810899 | 1.88727035433504e-10 | 7.11262958641316e-10 | 384.582271020474 | 441.919020694753 | 309.542471555281 | 160.04380488135 | 124.147302934146 | 131.003395861513 |
| YDR244W | 257.854001891321 | -1.45982530898802 | 0.367392804210548 | -3.97347278514311 | 7.08322546765292e-05 | 0.000148678965641414 | 465.90698050504 | 473.954042641027 | 194.842648366151 | 133.369837401125 | 141.271068856098 | 137.779433578487 |
| YDR245W | 2961.2133258282 | -0.767032357417856 | 0.185655110513645 | -4.131490672655 | 3.60418318658522e-05 | 7.86565684663249e-05 | 4042.67410979822 | 4186.73094513226 | 2962.34351044234 | 2271.28833094116 | 2004.90759336179 | 2299.33546529344 |
| YDR246W | 186.548748941629 | -3.24957204614621 | 0.413514823828877 | -7.85841730184494 | 3.89017423502799e-15 | 2.24881195083704e-14 | 430.944955866816 | 402.491301376262 | 179.402287552229 | 26.673967480225 | 41.3824343113821 | 38.3975470628571 |
| YDR246W-A | 23.3108484639817 | 0.796316018174292 | 0.414673465790949 | 1.92034476248775 | 0.0548143657920572 | 0.0767543127217795 | 15.2008802774891 | 20.5352704783807 | 15.4403608139214 | 33.3424593502813 | 27.1126293764228 | 28.2334904873949 |
| YDR247W | 784.131272495519 | -0.263908468710962 | 0.298202999998075 | -0.884996021879945 | 0.376158776935652 | 0.441586575059664 | 931.053916996206 | 769.66193752971 | 865.39546085645 | 558.819618710714 | 1044.54972123903 | 535.306979641008 |
| YDR248C | 200.061145372822 | -1.25789992884649 | 0.278771589572445 | -4.51229600109446 | 6.41296046255405e-06 | 1.53428482331706e-05 | 286.536593230669 | 259.565818846732 | 301.454663509893 | 113.364361790956 | 81.3378881292684 | 158.107546729412 |
| YDR249C | 861.710705190327 | 0.0653620360642525 | 0.158153700877769 | 0.413281736067423 | 0.679400209480742 | 0.73102019885403 | 761.564101902203 | 873.981111559884 | 890.394140269465 | 812.222309772852 | 1003.16728692764 | 828.935280709916 |
| YDR250C | 9.91072753067303 | -0.920276498048694 | 0.698381382482873 | -1.31772770742677 | 0.187594815843048 | 0.23829611742225 | 12.9207482358657 | 9.03551901048752 | 16.9108713676282 | 10.66958699209 | 4.28094148048781 | 5.64669809747899 |
| YDR251W | 1275.80401339145 | -1.81176131753907 | 0.167661600426987 | -10.806060021645 | 3.22213678582493e-27 | 4.83076257011791e-26 | 2058.19918957202 | 2233.41601722869 | 1666.82371262665 | 548.150031718624 | 550.814470489431 | 597.420658713277 |
| YDR252W | 107.486035665178 | -0.0162142134767971 | 0.231032298475622 | -0.0701815875259881 | 0.944049129210837 | 0.955838690612158 | 114.766646095043 | 116.640336317203 | 92.6421648835282 | 114.698060164968 | 111.304478492683 | 94.864528037647 |
| YDR253C | 12.2961157830623 | -1.74960383395899 | 0.650559039942043 | -2.68938516958408 | 0.00715837705017454 | 0.0115697068396292 | 22.0412764023592 | 14.7853947444341 | 19.8518924750418 | 8.0021902440675 | 5.70792197398374 | 3.38801885848739 |
| YDR254W | 168.095078003438 | -0.416603794785063 | 0.244489999791515 | -1.70397069467183 | 0.0883865622552683 | 0.11955688066479 | 179.370387274371 | 184.817434305427 | 212.488775010632 | 181.38297886553 | 112.731458986179 | 137.779433578487 |
| YDR255C | 140.422915311845 | -1.35754398632052 | 0.260070434617345 | -5.21990893858404 | 1.79011126699795e-07 | 5.02843727923288e-07 | 202.931751704479 | 234.10208345354 | 168.373458399428 | 76.0208073186413 | 95.6076930642277 | 65.5016979307563 |
| YDR256C | 137.520726245709 | 0.697167012335787 | 0.195478755875531 | 3.56645922577746 | 0.000361837077889162 | 0.00069764425897417 | 114.006602081168 | 95.2836550196866 | 105.141504590036 | 173.380788621463 | 176.945581193496 | 160.366225968403 |
| YDR257C | 428.860036492765 | -3.06414522182489 | 0.231276581599055 | -13.2488347961531 | 4.58262967542393e-40 | 1.70821471694251e-38 | 867.970263844627 | 837.839035517934 | 592.615753143839 | 93.3588861807875 | 89.899771090244 | 91.4765091791596 |
| YDR258C | 698.156817526397 | -0.121452302068452 | 0.38980586417004 | -0.311571254391064 | 0.755366383143825 | 0.79780269680359 | 560.912482239347 | 424.669393492913 | 1196.99559071733 | 678.852472371726 | 576.500119372358 | 751.010846964706 |
| YDR259C | 25.7048855255022 | -1.8420277909143 | 0.682292515784383 | -2.69976256268421 | 0.00693889783019266 | 0.0112401826490084 | 55.4832130128352 | 52.5702924246547 | 12.4993397065078 | 10.66958699209 | 12.8428244414634 | 10.1640565754622 |
| YDR260C | 440.900803027413 | 0.679667195798882 | 0.157657514734005 | 4.31103583578365 | 1.62491566416377e-05 | 3.70576758008657e-05 | 321.498617868894 | 323.63586273928 | 372.03917008782 | 544.14893659659 | 492.308270256098 | 591.773960615798 |
| YDR261C | 2712.60354485603 | 0.221354828991638 | 0.22209832473449 | 0.996652402742158 | 0.318933260548121 | 0.38208369558831 | 2071.11993780789 | 2154.56057859171 | 3288.79685336525 | 2780.76110981346 | 3023.77166571789 | 2956.61112384 |
| YDR261C-D | 105.548800916306 | -0.815972654031734 | 0.24462567993363 | -3.33559687704545 | 0.000851164914575145 | 0.00155423863624279 | 133.007702428029 | 152.782412359153 | 118.376099573397 | 86.6903943107313 | 65.641102700813 | 76.7950941257143 |
| YDR261W-B | 16.9359346819117 | -4.66002964304399 | 1.04902574674659 | -4.44224525231764 | 8.90250042185013e-06 | 2.09740710992081e-05 | 41.0423767492205 | 42.7133625950319 | 13.9698502602146 | 1.33369837401125 | 1.42698049349594 | 1.1293396194958 |
| YDR262W | 1346.6602498363 | 0.309480586796247 | 0.155480212911559 | 1.99048213917927 | 0.0465378495798123 | 0.0661214660185461 | 1211.51015811588 | 1166.40336317203 | 1229.34682289888 | 1411.0528797039 | 1738.06224107805 | 1323.58603404908 |
| YDR263C | 110.218784832904 | -1.51394895653432 | 0.25394138460094 | -5.961804764172 | 2.49467107604679e-09 | 8.40979033224507e-09 | 186.210783399241 | 165.103574646181 | 138.227992048439 | 53.34793496045 | 64.2141222073171 | 54.2083017357983 |
| YDR264C | 3179.97373677634 | 0.0346369090941099 | 0.183139862764827 | 0.189128180895209 | 0.849992354890961 | 0.87829383683651 | 3290.99058007639 | 3603.52926354625 | 2530.7486629294 | 3415.60153584281 | 3310.59474491057 | 2928.3776333526 |
| YDR265W | 528.88893462443 | 0.793324296418736 | 0.192186854285818 | 4.12788012669647 | 3.6612287940184e-05 | 7.9794119482538e-05 | 339.739674201881 | 359.77793878123 | 461.005058587081 | 634.840426029355 | 753.445700565854 | 624.524809581176 |
| YDR266C | 685.260356662173 | -4.00928964845973 | 0.263221199279757 | -15.2316365833383 | 2.18063813218904e-52 | 1.5715132139309e-50 | 1473.72534290257 | 1347.93515420091 | 1049.94453534665 | 102.694774798866 | 57.0792197398374 | 80.1831129842017 |
| YDR267C | 1326.98992989542 | 0.0697822272681739 | 0.203828652730855 | 0.342357300277688 | 0.732082018473195 | 0.778282168066483 | 1453.96419854183 | 1427.61200365703 | 1002.88819762804 | 1316.3602951491 | 1515.45328409268 | 1245.66160030387 |
| YDR268W | 581.43578290088 | 1.27946458187351 | 0.276067821872161 | 4.63460237124629 | 3.57624224204558e-06 | 8.76294188965154e-06 | 270.575668939306 | 265.315694580679 | 481.592206338976 | 802.886421154773 | 861.896218071545 | 806.34848832 |
| YDR269C | 1.60585269440112 | 0.169176773700728 | 1.63698188349156 | 0.103346759916418 | 0.917687765224652 | 0.934252526329791 | 0.760044013874454 | 0.821410819135229 | 2.94102110741359 | 0 | 2.85396098699187 | 2.2586792389916 |
| YDR270W | 398.173645807256 | -0.437331380906722 | 0.171459534413795 | -2.55063903213035 | 0.0107525627300817 | 0.0168864701857893 | 455.266364310798 | 489.560848204597 | 429.389081682385 | 392.107321959308 | 305.37382560813 | 317.344433078319 |
| YDR271C | 6.43496265204085 | 0.475084646558734 | 0.808278561936595 | 0.58777340997447 | 0.556684393948223 | 0.618262838895236 | 6.84039612487009 | 3.28564327654092 | 5.88204221482719 | 6.66849187005625 | 11.4158439479675 | 4.51735847798319 |
| YDR272W | 593.479264778818 | -1.38237735052945 | 0.17504469766911 | -7.89728205959474 | 2.85050822859378e-15 | 1.67013517350129e-14 | 893.811760316358 | 748.305256232194 | 932.303691050109 | 342.760482120891 | 286.823079192683 | 356.871319760672 |
| YDR273W | 40.8475958889232 | -0.824689938206981 | 0.341247959582549 | -2.41668826156742 | 0.0156624245059384 | 0.0240840410966137 | 47.8827728740906 | 48.4632383289785 | 60.2909327019787 | 24.0065707322025 | 32.8205513504065 | 31.6215093458823 |
| YDR274C | 5.84546178202534 | 1.85229828655635 | 0.882814525244765 | 2.09817377669765 | 0.035889797213624 | 0.0520414095076158 | 3.04017605549782 | 1.64282163827046 | 2.94102110741359 | 5.334793496045 | 8.56188296097562 | 13.5520754339496 |
| YDR275W | 440.270781122756 | -0.901851970486401 | 0.175157625199259 | -5.14880222576925 | 2.62155117293637e-07 | 7.2231864518544e-07 | 628.556399474174 | 573.34475175639 | 519.825480735353 | 313.419117892644 | 265.418371790244 | 341.060565087731 |
| YDR276C | 10387.0904250051 | 1.42656826539974 | 0.203127588666412 | 7.02301580383809 | 2.17129640701468e-12 | 9.74604048158976e-12 | 6233.88100179827 | 6242.72222542774 | 4421.82523499634 | 14166.5441287475 | 17503.3427332212 | 13754.2272258393 |
| YDR277C | 139.875234769729 | -2.02331313797485 | 0.285589654439363 | -7.08468639015228 | 1.39359528971735e-12 | 6.36539369655404e-12 | 247.014304509198 | 261.208640485003 | 165.432437292015 | 60.0164268305063 | 51.3712977658537 | 54.2083017357983 |
| YDR279W | 358.033430619045 | -2.63439054122021 | 0.207287043683907 | -12.7089011179947 | 5.27707700926835e-37 | 1.591959138703e-35 | 636.156839612918 | 626.73645500018 | 588.939476759572 | 93.3588861807875 | 79.9109076357724 | 123.098018525042 |
| YDR280W | 1212.89261420294 | -0.772311468942643 | 0.191996732274829 | -4.02252402836282 | 5.7577778094933e-05 | 0.000122683793930268 | 1725.29991149501 | 1650.21433564268 | 1214.64171736181 | 980.268304898269 | 836.210569188618 | 870.72084663126 |
| YDR281C | 111.655204798319 | 1.30205768859656 | 0.258795798406684 | 5.03121649042557 | 4.8737742045449e-07 | 1.3089565006492e-06 | 68.4039612487009 | 69.8199196264945 | 55.1441457640049 | 194.719962605643 | 138.417107869106 | 143.426131675966 |
| YDR282C | 255.911294061587 | -0.467444705231482 | 0.175576979942413 | -2.66233480826927 | 0.00776006487641832 | 0.0124954768590986 | 316.178309771773 | 316.243165367063 | 258.809857452396 | 205.389549597733 | 219.754995998374 | 219.091886182185 |
| YDR283C | 1091.27903609719 | -0.632498571785647 | 0.112216100372504 | -5.63643336104223 | 1.73608171583029e-08 | 5.36456694086482e-08 | 1379.47988518213 | 1338.89963519042 | 1262.43331035728 | 872.238736603358 | 820.513783760163 | 874.108865489748 |
| YDR284C | 3221.35128779686 | 1.11724092216981 | 0.124823223222742 | 8.95058542252296 | 3.53602021342407e-19 | 2.96313011683056e-18 | 1933.55197129661 | 2051.06281538067 | 2113.12366567667 | 4421.2101098473 | 4793.22747765285 | 4015.93168692706 |
| YDR285W | 13.2403238852687 | -0.538917918049517 | 0.586839065156197 | -0.918340223151427 | 0.358440775296983 | 0.422930119806482 | 12.1607042219913 | 16.4282163827046 | 18.381381921335 | 16.004380488135 | 8.56188296097562 | 7.90537733647059 |
| YDR286C | 279.632193918323 | 1.03010493526919 | 0.197267019499844 | 5.2218811734518 | 1.77114611269421e-07 | 4.97947710747058e-07 | 198.371487621233 | 193.031542496779 | 160.285650354041 | 434.785669927668 | 331.059474491057 | 360.25933861916 |
| YDR287W | 711.656474485664 | 1.02206958784226 | 0.219629098965596 | 4.65361644998764 | 3.26163261389354e-06 | 8.0437068949481e-06 | 395.982931228591 | 420.562339397237 | 591.880497866986 | 853.5669593672 | 1077.37027258943 | 930.575846464537 |
| YDR288W | 419.09351095229 | -1.02080003448417 | 0.271771779396334 | -3.75609283918881 | 0.000172586692118846 | 0.00034666995512011 | 681.759480445385 | 625.915044181045 | 377.185957025793 | 278.742960168351 | 248.294605868293 | 302.663018024874 |
| YDR289C | 133.075417002315 | -2.44131053169181 | 0.364073799431826 | -6.70553754623847 | 2.00666286164155e-11 | 8.22186691131214e-11 | 305.537693577531 | 225.887975262188 | 142.639523709559 | 44.0120463423713 | 44.236395298374 | 36.1388678238655 |
| YDR291W | 673.252239131898 | -0.570223014552674 | 0.117553571106009 | -4.8507502510362 | 1.22995340434222e-06 | 3.16277834116785e-06 | 772.204718096446 | 840.303267975339 | 801.428251770204 | 532.145651230489 | 547.960509502439 | 545.47103621647 |
| YDR292C | 2048.42898254791 | -1.11378743580741 | 0.204318250916894 | -5.4512381092203 | 5.0020343136495e-08 | 1.48142440905619e-07 | 3050.05662767818 | 3252.78684377551 | 2103.56534707757 | 1276.34934392877 | 1305.68715154878 | 1302.12858127866 |
| YDR293C | 4113.9306481019 | -1.46306467300486 | 0.144470426949497 | -10.1270876254578 | 4.1894171133619e-24 | 5.11724282434374e-23 | 5371.99109006464 | 5863.23042698726 | 6878.31311496354 | 2096.57384394569 | 2226.08956985366 | 2247.38584279664 |
| YDR294C | 4898.6062683617 | 0.633976628439353 | 0.177013631520641 | 3.58151303373167 | 0.000341610092103639 | 0.000660522511011496 | 3360.91462935284 | 3515.63830589878 | 4640.93130749865 | 5544.18414076477 | 6328.65848865448 | 6001.31073800067 |
| YDR295C | 126.753446762538 | -3.40470101551145 | 0.6205164349367 | -5.48688289917538 | 4.09088121892676e-08 | 1.22049013734862e-07 | 353.420466451621 | 283.386732601654 | 58.0851668714185 | 21.33917398418 | 22.831687895935 | 21.4574527704202 |
| YDR296W | 452.291577764294 | -0.859829519825426 | 0.167667587825228 | -5.12817969756736 | 2.92557127255283e-07 | 8.01658439956807e-07 | 589.034110752702 | 584.844503224283 | 577.175392329918 | 304.083229274565 | 282.542137712195 | 376.070093292101 |
| YDR297W | 7134.99983748353 | 0.496996782431374 | 0.190038270614775 | 2.61524576509556 | 0.00891632726324198 | 0.014167393098821 | 6729.42969884442 | 6388.11194041468 | 4636.51977583753 | 7966.1803879692 | 9109.84347047806 | 7979.91375135731 |
| YDR298C | 7138.83287393497 | 0.682814126833508 | 0.196471000858732 | 3.47539394541218 | 0.000510103646391058 | 0.000960665577959466 | 6005.10775362206 | 6273.11442573574 | 4163.01537754394 | 9198.5176855556 | 8544.75919505366 | 8648.48280609882 |
| YDR299W | 350.166299463149 | -1.26500976799771 | 0.296035157721416 | -4.27317409774737 | 1.92709871654995e-05 | 4.34602304434735e-05 | 550.271866045105 | 561.845000288497 | 372.03917008782 | 258.737484558183 | 141.271068856098 | 216.833206943193 |
| YDR300C | 1539.93632484575 | -0.174264563372144 | 0.191322299117385 | -0.910842929319101 | 0.362378139525016 | 0.427141507315466 | 1818.02528118769 | 1793.1398181722 | 1287.4319897703 | 1583.09996995135 | 1375.60919573008 | 1382.31169426286 |
| YDR301W | 743.42365580213 | -0.596826845638733 | 0.140661684267235 | -4.24299516067826 | 2.20556193631941e-05 | 4.9362576670006e-05 | 842.88881138677 | 848.517376166692 | 994.065134305795 | 586.82728456495 | 569.365216904878 | 618.878111483697 |
| YDR302W | 1967.85335422148 | 1.72049171264687 | 0.166523033687481 | 10.3318542459164 | 5.05733710323983e-25 | 6.6266441316391e-24 | 841.368723359021 | 848.517376166692 | 1058.76759866889 | 3222.21527161118 | 2668.4535228374 | 3167.79763268571 |
| YDR303C | 828.780699640344 | -1.01757793449886 | 0.15285222851507 | -6.65726593838004 | 2.78968159498897e-11 | 1.13299153569809e-10 | 1118.02474440932 | 1177.90311463992 | 1033.03366397902 | 616.168648793198 | 486.600348282114 | 540.953677738487 |
| YDR304C | 7244.63822183389 | 1.33734558777752 | 0.137489720170253 | 9.72687693393723 | 2.31594227562609e-22 | 2.41498418001782e-21 | 3829.86178591337 | 3858.16661747817 | 4636.51977583753 | 9695.98717906179 | 10660.9712669081 | 10786.3227058044 |
| YDR305C | 468.533553545951 | 0.34909975016397 | 0.213654823313414 | 1.63394275284799 | 0.102270871598732 | 0.136684292847594 | 446.905880158179 | 376.206155163935 | 413.948720868463 | 545.482634970601 | 405.262460152846 | 623.39546996168 |
| YDR306C | 544.052300693427 | -0.560337892832216 | 0.144525468165755 | -3.87708754687837 | 0.000105714327127207 | 0.000217050688745509 | 663.518424112399 | 676.021104148293 | 605.115092850347 | 412.112797569476 | 485.173367788618 | 422.373017691428 |
| YDR307W | 1300.21364050901 | 0.610419560048849 | 0.124204787586076 | 4.91462182668135 | 8.89539973532688e-07 | 2.32268770866869e-06 | 940.934489176574 | 1102.33331927948 | 1044.06249313183 | 1585.76736669938 | 1623.90380159838 | 1504.2803731684 |
| YDR308C | 534.323508551336 | -0.477417461379043 | 0.201971539234289 | -2.36378582442367 | 0.018089266849448 | 0.0275933642487111 | 662.758380098524 | 658.771476946454 | 544.824160148368 | 521.476064238399 | 359.599084360976 | 458.511885515294 |
| YDR309C | 1012.42973468385 | -1.7008193681111 | 0.132961714286328 | -12.7917978287227 | 1.82209401844946e-37 | 5.79318715865842e-36 | 1592.29220906698 | 1588.60852420753 | 1466.09902204568 | 478.797716270039 | 433.802070022764 | 514.978866490084 |
| YDR310C | 551.446352245586 | -2.23977118965304 | 0.439056492186234 | -5.10132802842828 | 3.37278404547331e-07 | 9.17227560542552e-07 | 1221.39073029625 | 1062.90559996099 | 447.035208326866 | 160.04380488135 | 132.709185895122 | 284.593584112941 |
| YDR311W | 472.77048528833 | -2.42959290800583 | 0.373858799469609 | -6.4986912477456 | 8.10217496969042e-11 | 3.15430413285787e-10 | 1033.65985886926 | 959.407836749948 | 399.243615331395 | 156.042709759316 | 158.394834778049 | 129.874056242017 |
| YDR312W | 87.4541446059318 | -3.17730328791585 | 0.534392851704475 | -5.94563209029026 | 2.75392068066533e-09 | 9.22620327210501e-09 | 224.973028106838 | 181.531791028886 | 66.1729749168059 | 20.0054756101688 | 12.8428244414634 | 19.1987735314286 |
| YDR313C | 465.546996718547 | 0.9332223088389 | 0.252331447176779 | 3.6983987500579 | 0.000216963862915911 | 0.000430608205285373 | 259.935052745063 | 273.529802772031 | 426.448060574971 | 533.4793496045 | 633.579339112195 | 666.310375502521 |
| YDR314C | 118.247208844736 | -3.63682091103845 | 0.419179417822214 | -8.67604838504005 | 4.09757888710927e-18 | 3.14892140542544e-17 | 254.614744647942 | 252.173121474515 | 150.727331754947 | 16.004380488135 | 9.98886345447155 | 25.9748112484034 |
| YDR315C | 180.592846283762 | -0.482451042965706 | 0.190970007631927 | -2.52631839391018 | 0.0115264964965304 | 0.0180233501148737 | 215.852499940345 | 200.424239868996 | 214.694540841192 | 172.047090247451 | 148.405971323577 | 132.132735481008 |
| YDR316W | 91.4220720878785 | -1.19124119009598 | 0.357544935103229 | -3.33172441598719 | 0.000863096794814239 | 0.00157425360268986 | 153.52889080264 | 133.889963519042 | 94.112675437235 | 77.3545056926525 | 39.9554538178862 | 49.6909432578151 |
| YDR317W | 69.4604355063239 | -0.385554981298928 | 0.291665075557765 | -1.32191000434869 | 0.186198120635063 | 0.236800198125297 | 91.965325678809 | 82.9624927326581 | 61.0261879788321 | 62.6838235785288 | 62.7871417138212 | 55.3376413552941 |
| YDR318W | 241.505238793258 | -0.714683429928666 | 0.227327587053075 | -3.14384822006581 | 0.0016674189965559 | 0.00293563507374092 | 324.538793924392 | 315.421754547928 | 259.54511272925 | 192.05256585762 | 214.04707402439 | 143.426131675966 |
| YDR319C | 1185.49572615792 | 0.363892751914782 | 0.182733733006673 | 1.99138246632051 | 0.0464388553842294 | 0.0659952708199193 | 1070.90201554911 | 1181.18875791646 | 858.042908087916 | 1284.35153417283 | 1489.76763520976 | 1228.72150601143 |
| YDR320C | 420.325505120443 | -1.45640273073165 | 0.163240265188768 | -8.92183511860565 | 4.58624660129482e-19 | 3.8136404430767e-18 | 599.674726946944 | 570.059108479849 | 678.640620535687 | 204.055851223721 | 236.878761920325 | 232.643961616134 |
| YDR320C-A | 1275.91846072834 | 1.40481449033685 | 0.195103291034776 | 7.20036285849454 | 6.00525311777396e-13 | 2.8347941573422e-12 | 798.046214568177 | 629.200687457585 | 671.288067767153 | 2216.6066976067 | 1585.37532827398 | 1754.99376869647 |
| YDR320W-B | 22.2384191493826 | 0.734163184858027 | 0.45584590699175 | 1.6105512270648 | 0.107277571562681 | 0.142816570023717 | 19.0011003468614 | 16.4282163827046 | 14.705105537068 | 37.343554472315 | 19.9777269089431 | 25.9748112484034 |
| YDR321W | 7114.7370234252 | 0.423900600923108 | 0.171234974394235 | 2.47554918276894 | 0.0133031437463138 | 0.0206224164289175 | 6583.50124818052 | 6767.60373885515 | 4879.889272476 | 8159.56665220083 | 8377.80247731464 | 7920.05875152403 |
| YDR322C-A | 4068.73642347227 | 1.63042397888291 | 0.129433041729859 | 12.5966596866802 | 2.20277266738863e-36 | 6.40680875366934e-35 | 2176.00601172256 | 1945.92223053136 | 1838.1381921335 | 6604.47434810371 | 5814.94551099594 | 6032.93224734655 |
| YDR322W | 902.305171888165 | 0.402688906124627 | 0.167996195298442 | 2.39701205976276 | 0.0165293796749838 | 0.02536303680434 | 685.559700514758 | 726.127164115543 | 919.804351343601 | 1044.28582685081 | 988.897481992684 | 1049.1565065116 |
| YDR323C | 192.532694633384 | -1.18735753414019 | 0.194519732888153 | -6.10404670267002 | 1.03416046351712e-09 | 3.63356704570534e-09 | 288.816725272293 | 279.279678505978 | 234.546433316234 | 124.033948783046 | 115.585419973171 | 112.93396194958 |
| YDR324C | 1619.21085214371 | -3.25784478524842 | 0.338004292416498 | -9.63847163583952 | 5.50007388652008e-22 | 5.55661670217589e-21 | 3312.27181246487 | 3325.07099585941 | 2159.44474811843 | 354.763767486993 | 174.091620206504 | 389.62216872605 |
| YDR325W | 363.922762673217 | -1.67207025370465 | 0.21679759203767 | -7.71258683267166 | 1.23292735079458e-14 | 6.79997176637217e-14 | 635.396795599044 | 530.631389161358 | 497.032567152897 | 148.040519515249 | 161.248795765041 | 211.186508845714 |
| YDR326C | 924.135831650647 | -3.08661036138271 | 0.298936622199342 | -10.3253001879591 | 5.41491593351182e-25 | 7.08087595660437e-24 | 1975.35439205971 | 1962.35044691406 | 1022.74009010308 | 208.056946345755 | 206.912171556911 | 169.40094292437 |
| YDR327W | 1.84937108594007 | 1.94891038940176 | 1.8172487514111 | 1.07245108182819 | 0.283517474678847 | 0.344814239783799 | 2.28013204162336 | 0 | 0 | 4.00109512203375 | 1.42698049349594 | 3.38801885848739 |
| YDR328C | 2072.08969715548 | -0.284204305994339 | 0.193293055731813 | -1.47032858950019 | 0.141472781568382 | 0.183923123121373 | 2236.80953283252 | 2152.0963461343 | 2438.84175332272 | 1841.83745450954 | 1499.75649866423 | 2263.19659746958 |
| YDR329C | 744.250508457509 | -1.11670705125758 | 0.131972379033626 | -8.4616725062829 | 2.63569819463294e-17 | 1.88794322844848e-16 | 1036.70003492476 | 1079.33381634369 | 940.391499095496 | 446.788955293769 | 482.319406801626 | 479.969338285714 |
| YDR330W | 209.692866914863 | -2.87907370078489 | 0.217134410755451 | -13.2594078053684 | 3.98026408650971e-40 | 1.50092981773849e-38 | 366.341214687487 | 353.206652228149 | 388.950041455448 | 50.6805382124275 | 41.3824343113821 | 57.5963205942857 |
| YDR331W | 1506.01298470346 | 0.652523648797083 | 0.106546042195144 | 6.12433493871085 | 9.10631592360052e-10 | 3.21346926444358e-09 | 1127.14527257582 | 1219.79506641582 | 1166.11486908949 | 1897.85278621801 | 1867.91746598618 | 1757.25244793546 |
| YDR332W | 239.166597737998 | -2.75113742021324 | 0.260203896482904 | -10.5730062362613 | 3.9754292828974e-26 | 5.5690354921971e-25 | 479.587772754781 | 436.169144960807 | 334.541150968296 | 61.3501252045175 | 49.9443172723578 | 73.4070752672269 |
| YDR333C | 1124.35230625176 | -0.434431811354397 | 0.135058694312843 | -3.21661492112533 | 0.00129712572066054 | 0.00231444535001049 | 1348.31808061328 | 1359.4349056688 | 1169.0558901969 | 1020.27925611861 | 948.942028174797 | 900.083676738151 |
| YDR334W | 390.716264760517 | -0.820528021189323 | 0.198079041508955 | -4.14242726004017 | 3.43649378224075e-05 | 7.52247677070991e-05 | 478.827728740906 | 561.845000288497 | 457.328782202814 | 273.408166672306 | 239.732722907317 | 333.15518775126 |
| YDR335W | 1624.35835755152 | -0.303522437434611 | 0.166520676270699 | -1.8227312321336 | 0.0683441075454922 | 0.0943148684127793 | 1656.13590623244 | 1642.00022745132 | 2085.91922043309 | 1552.4249073491 | 1304.26017105529 | 1505.4097127879 |
| YDR336W | 502.853706295053 | -0.063849248909025 | 0.224712596350834 | -0.284137382353679 | 0.776305100052075 | 0.815030959704554 | 592.834330822074 | 570.880519298984 | 378.6564675795 | 480.13141464405 | 469.476582360163 | 525.142923065546 |
| YDR337W | 312.862264666141 | -1.49385611345458 | 0.345091545310678 | -4.32886906026544 | 1.49877006089481e-05 | 3.42772306592515e-05 | 497.828829087768 | 386.063084993558 | 502.179354090871 | 197.387359353665 | 87.0458101032521 | 206.669150367731 |
| YDR338C | 809.013910999147 | -0.0560927727040327 | 0.134407271370278 | -0.417334360947651 | 0.676433858761573 | 0.728311754304044 | 832.248195192527 | 846.053143709286 | 795.546209555377 | 722.864518714098 | 874.739042513009 | 782.632356310588 |
| YDR339C | 1411.92689454886 | -1.3998072691187 | 0.248966968818204 | -5.62246178986435 | 1.88255215571867e-08 | 5.789584296819e-08 | 2425.30044827338 | 2289.27195292988 | 1429.33625820301 | 821.55819839093 | 669.253851449594 | 836.840658046386 |
| YDR340W | 1.18864030292905 | 1.15478734468271 | 2.45116699773552 | 0.471117368073877 | 0.637556922967247 | 0.693010591983503 | 0 | 0 | 2.20576583056019 | 2.6673967480225 | 0 | 2.2586792389916 |
| YDR341C | 13406.124151473 | 0.511371242466779 | 0.123033669726911 | 4.1563520262529 | 3.23369446163225e-05 | 7.10252024319228e-05 | 10267.43458343 | 11067.6893770281 | 11828.7868940175 | 17079.3413775881 | 15217.3199826407 | 14976.1726941338 |
| YDR342C | 5946.97577875002 | 2.13688310167806 | 0.217629315717121 | 9.81891200933437 | 9.33455226838535e-23 | 1.00571272446424e-21 | 2313.57397823384 | 2301.59311521691 | 1994.74756610327 | 7970.18148309123 | 12507.4840254919 | 8594.27450436302 |
| YDR343C | 956.306232621193 | 3.04745430554674 | 0.448374778591793 | 6.79666754476658 | 1.07066777431668e-11 | 4.49763677734327e-11 | 179.370387274371 | 174.139093656669 | 265.427154944077 | 902.913799205617 | 2989.52413387399 | 1226.46282677244 |
| YDR344C | 14.7039073789762 | -0.111714542037213 | 0.72865633913412 | -0.153315817124389 | 0.878149227876256 | 0.902213827341264 | 8.360484152619 | 11.4997514678932 | 25.7339346898689 | 9.33588861807875 | 24.2586683894309 | 9.03471695596638 |
| YDR345C | 47054.4281833867 | 0.670986150842426 | 0.411363316860166 | 1.63112782142048 | 0.10286335131949 | 0.137362918809597 | 47486.789942862 | 46775.2390956556 | 14653.6376676882 | 58612.0424426724 | 61969.481891048 | 52829.3780603939 |
| YDR346C | 3266.24863208973 | -1.37453674815806 | 0.218710669710026 | -6.28472652925651 | 3.28431311831575e-10 | 1.22144810122683e-09 | 5231.38294749787 | 5513.30941803566 | 3398.34988961641 | 1820.49828052536 | 1817.97314871382 | 1815.97810814924 |
| YDR347W | 2089.70088711815 | 1.06330394914594 | 0.247450053861989 | 4.29704472700973 | 1.73090279847359e-05 | 3.92539704576913e-05 | 1064.06161942424 | 1140.1182169597 | 1853.57855294742 | 2892.7917732304 | 2786.89290379756 | 2800.76225634958 |
| YDR348C | 472.084905103989 | -5.13531008914422 | 0.274006702455486 | -18.7415491779019 | 2.2692358417229e-78 | 7.00869698543557e-76 | 1027.57950675826 | 969.26476657957 | 757.312935159 | 30.6750626022588 | 22.831687895935 | 24.8454716289076 |
| YDR349C | 4342.53735065317 | -0.0133711258155857 | 0.117335307699897 | -0.113956541110237 | 0.909272244118254 | 0.927869694045154 | 4338.33123119539 | 4605.65046289123 | 4143.1634850689 | 4422.54380822131 | 4563.4836182 | 3982.05149834218 |
| YDR350C | 1380.17751488336 | -0.0531664141156767 | 0.209638945365002 | -0.253609433223911 | 0.799797308196199 | 0.835882265704245 | 1190.2289257274 | 1340.54245682869 | 1685.20509454799 | 1301.68961303498 | 1596.79117222195 | 1166.60782693916 |
| YDR351W | 615.370522547575 | -2.0431295763319 | 0.263981693004799 | -7.7396638875816 | 9.96799741108272e-15 | 5.56868485859453e-14 | 1185.66866164415 | 1113.01165992824 | 672.75857832086 | 262.738579680216 | 219.754995998374 | 238.290659713613 |
| YDR352W | 2693.42071699197 | 0.591090882241115 | 0.199012314354052 | 2.97012214625843 | 0.00297681361136343 | 0.00507560806606289 | 2324.97463844196 | 2469.98233313963 | 1652.85386236644 | 3462.28097893321 | 3273.49325207968 | 2976.93923699092 |
| YDR353W | 13216.6066021215 | 1.06940271903535 | 0.177649605908883 | 6.01973032005401 | 1.74707900272854e-09 | 6.00506328123864e-09 | 7475.79292046913 | 7700.72642939277 | 10415.6262519052 | 17001.9868718954 | 18082.6968135805 | 18622.8103254857 |
| YDR354W | 4718.63969690227 | 0.426739690665207 | 0.155719268691804 | 2.74044242726185 | 0.0061356530332129 | 0.00999895617422586 | 4146.80013969902 | 4548.9731163709 | 3382.17427352563 | 5544.18414076477 | 5232.7374696496 | 5456.9690414037 |
| YDR356W | 168.281491303317 | 0.960922864743342 | 0.191203545193309 | 5.02565401583867 | 5.01719718050278e-07 | 1.34387190189909e-06 | 112.486514053419 | 114.997514678932 | 115.435078465984 | 193.386264231631 | 228.31687895935 | 245.066697430588 |
| YDR357C | 339.404084164519 | 0.214905465310676 | 0.22781642986338 | 0.943327333500716 | 0.345513499044597 | 0.409539575073694 | 329.099058007639 | 313.778932909657 | 299.984152956187 | 449.456352041791 | 272.553274257724 | 371.552734814118 |
| YDR358W | 199.187748454011 | -2.16061339052738 | 0.275396090422696 | -7.84547590058788 | 4.3131467149622e-15 | 2.48005936110327e-14 | 373.941654826231 | 324.457273558415 | 279.397005204291 | 73.3534105706188 | 51.3712977658537 | 92.6058487986554 |
| YDR359C | 275.80720372876 | -1.1671339120981 | 0.307400123695163 | -3.79679063908088 | 0.000146581484105961 | 0.000296269088785061 | 481.10786078253 | 428.77644758859 | 235.281688593087 | 173.380788621463 | 161.248795765041 | 175.047641021849 |
| YDR360W | 2.01753690873533 | -0.478891045820912 | 1.58262238064745 | -0.30259337393232 | 0.762199764718453 | 0.80318889910055 | 3.80022006937227 | 3.28564327654092 | 0 | 1.33369837401125 | 1.42698049349594 | 2.2586792389916 |
| YDR361C | 1749.20582659437 | -0.596353485655015 | 0.226560139454628 | -2.63220832707178 | 0.00848318278614346 | 0.0135622192632306 | 2358.41657505243 | 2243.27294705831 | 1716.08581617583 | 1585.76736669938 | 1065.95442864146 | 1525.73782593882 |
| YDR362C | 555.437353079752 | 0.0147391384616258 | 0.118782028257555 | 0.124085593400265 | 0.901247504799129 | 0.921565712774263 | 556.3522181561 | 542.131140629251 | 559.529265685436 | 574.823999198849 | 535.117685060976 | 564.669809747899 |
| YDR363W | 200.513765580811 | -3.10484978450573 | 0.477165522200279 | -6.50686112062082 | 7.67372936485466e-11 | 2.99109427045958e-10 | 464.386892477291 | 470.668399364486 | 142.639523709559 | 37.343554472315 | 48.5173367788618 | 39.5268866823529 |
| YDR363W-A | 369.514450963216 | -1.6129078172796 | 0.352684123205447 | -4.57323625067194 | 4.80248108413004e-06 | 1.1648800415732e-05 | 631.596575529671 | 509.274707863842 | 530.854309888154 | 212.058041467789 | 92.7537320772358 | 240.549338952605 |
| YDR364C | 562.090800084748 | -0.473424259244046 | 0.143954428721549 | -3.28870923561366 | 0.00100647942454668 | 0.0018214356996679 | 670.358820237269 | 668.628406776076 | 621.290708941122 | 525.477159360433 | 445.217913970732 | 441.571791222857 |
| YDR365C | 82.01568651249 | -3.342499667727 | 0.714964817876519 | -4.67505474976292 | 2.93875208336203e-06 | 7.29179265978811e-06 | 146.68849467777 | 256.280175570191 | 44.8505718880573 | 5.334793496045 | 24.2586683894309 | 14.6814150534454 |
| YDR366C | 4.8378460028989 | -0.0680922278976472 | 0.886044922031226 | -0.076849633923242 | 0.938743165293711 | 0.951506199421005 | 4.56026408324673 | 7.39269737221706 | 2.94102110741359 | 5.334793496045 | 4.28094148048781 | 4.51735847798319 |
| YDR367W | 2726.32479545335 | 0.639209193340966 | 0.20008657637608 | 3.19466305495436 | 0.0013999417902434 | 0.00248904124219263 | 2280.89208563724 | 2482.30349542666 | 1633.0019698914 | 3314.24045941796 | 3547.4735068309 | 3100.03725551597 |
| YDR368W | 2484.49041247983 | 1.60058374028116 | 0.310315591676287 | 5.15792239647066 | 2.497050659877e-07 | 6.90066918617905e-07 | 905.212420524475 | 924.908582346268 | 1866.07789265392 | 3753.02722446766 | 3907.07259119187 | 3550.64376369479 |
| YDR369C | 275.495188466686 | -1.17219246104697 | 0.274900598831839 | -4.26405932190792 | 2.00746110264184e-05 | 4.52411143562717e-05 | 454.506320296924 | 399.205658099721 | 290.425834357092 | 172.047090247451 | 206.912171556911 | 129.874056242017 |
| YDR370C | 475.542664977716 | -0.482583016601745 | 0.165475194049146 | -2.91634658218572 | 0.0035415684954284 | 0.00596794317000483 | 589.034110752702 | 591.415789777365 | 483.062716892683 | 424.116082935578 | 362.453045347968 | 403.17424416 |
| YDR371C-A | 2.32230869374011 | 1.02413042726276 | 1.33988017276507 | 0.764344788496491 | 0.444661801350626 | 0.510094878592176 | 0.760044013874454 | 0.821410819135229 | 2.94102110741359 | 4.00109512203375 | 4.28094148048781 | 1.1293396194958 |
| YDR371W | 306.403864947201 | -1.22382416719415 | 0.176798057148943 | -6.92215846106922 | 4.44813264743845e-12 | 1.94542065753782e-11 | 433.985131922313 | 459.168647896593 | 394.832083670275 | 200.054756101688 | 158.394834778049 | 191.987735314286 |
| YDR372C | 2737.50342302667 | -0.29523674261767 | 0.126307461519726 | -2.33744498595248 | 0.0194160583270915 | 0.0294234939975503 | 3119.98067695463 | 3141.89638319225 | 2788.82326510494 | 2604.71292444397 | 2248.9212577496 | 2520.68603071462 |
| YDR373W | 1483.16020318857 | 0.775248301332141 | 0.122325679905263 | 6.33757606687775 | 2.33407984427073e-10 | 8.75583682471947e-10 | 1153.74681306142 | 1125.33282221526 | 1002.88819762804 | 1971.20619678863 | 1842.23181710325 | 1803.55537233479 |
| YDR374C | 31.5916166156414 | 0.950599862058637 | 0.356969561415873 | 2.66297176232115 | 0.0077453913490298 | 0.0124780447813729 | 19.0011003468614 | 20.5352704783807 | 24.9986794130155 | 42.67834796836 | 42.8094148048781 | 39.5268866823529 |
| YDR374W-A | 519.770963041569 | 1.98671874767865 | 0.223244602485823 | 8.89929129554125 | 5.62044069896467e-19 | 4.64384437878788e-18 | 239.413864370453 | 232.45926181527 | 156.609373969774 | 770.877660178503 | 933.245242746342 | 786.020375169075 |
| YDR375C | 1116.02945840334 | 1.32118176226684 | 0.275150499476579 | 4.80166950370846 | 1.57348235901788e-06 | 4.00691267396545e-06 | 487.9482569074 | 527.345745884817 | 898.481948314853 | 1645.78379352988 | 1409.85672757398 | 1726.76027820908 |
| YDR376W | 1128.29435799166 | 1.06380530268283 | 0.227383536752789 | 4.67846229271823 | 2.89034308752048e-06 | 7.18266868416008e-06 | 585.23389068333 | 643.98608220202 | 960.978646847392 | 1553.75860572311 | 1555.40873791057 | 1470.40018458353 |
| YDR377W | 5133.50765530978 | 1.66311210153179 | 0.175640703556839 | 9.46883078838039 | 2.82993470717081e-21 | 2.71925281640147e-20 | 2733.87831790641 | 2680.26350283825 | 1977.83669473564 | 8338.28223431834 | 7557.28869355448 | 7513.49648850554 |
| YDR378C | 1518.07594025455 | 1.122861376959 | 0.290715437419223 | 3.86240712542481 | 0.000112275254252625 | 0.000229939153483589 | 1193.26910178289 | 1090.01215699245 | 583.057434544745 | 2095.24014557167 | 2284.59577008699 | 1862.28103254857 |
| YDR379C-A | 430.093977976655 | 2.5294595919737 | 0.40538981833223 | 6.23957356003631 | 4.38765386279799e-10 | 1.61091632015601e-09 | 86.6450175816878 | 76.3912061795763 | 217.635561948606 | 792.216834162683 | 706.355344280488 | 701.31990370689 |
| YDR379W | 198.64445039867 | -1.9588297454732 | 0.338720411826412 | -5.78302835341692 | 7.33676912468527e-09 | 2.36043078088833e-08 | 400.543195311837 | 351.563830589878 | 195.577903643004 | 93.3588861807875 | 84.1918491162602 | 66.6310375502521 |
| YDR380W | 3193.36438932333 | 0.84191054316331 | 0.493022233484338 | 1.70765228418457 | 0.087700880793869 | 0.118728430980805 | 1151.4666810198 | 1288.79357522317 | 4421.08997971948 | 4581.25391472865 | 3523.21483844147 | 4194.36734680739 |
| YDR381C-A | 219.538350162282 | 0.586432311394188 | 0.347123926238886 | 1.6894033141081 | 0.0911421663336034 | 0.123027698405776 | 180.89047530212 | 128.961498604231 | 216.900306671753 | 320.0876097627 | 159.821815271545 | 310.568395361344 |
| YDR381W | 4740.51784534745 | -1.10362096265852 | 0.175685257467903 | -6.28180746958886 | 3.34659139515902e-10 | 1.2438963775932e-09 | 7093.49078149028 | 6936.81436759701 | 5381.33337129002 | 2931.46902607673 | 2725.53274257724 | 3374.46678305344 |
| YDR382W | 81290.2130140724 | 0.651289713695089 | 0.296439062984771 | 2.19704416529122 | 0.0280172921343135 | 0.0414602228572114 | 78416.0210434691 | 74547.1388906177 | 36777.468948207 | 96259.680144262 | 102891.001503031 | 98849.9675548477 |
| YDR383C | 65.6872989543483 | -1.23965695091801 | 0.370969565780473 | -3.34166752550153 | 0.000832767457588334 | 0.00152164756726414 | 116.286734122792 | 93.6408333814161 | 66.9082301936593 | 52.0142365864388 | 31.3935708569106 | 33.8801885848739 |
| YDR384C | 1888.5056474545 | -0.369138582341752 | 0.170787719524103 | -2.16138832095394 | 0.0306653555091337 | 0.0450091640263048 | 2362.97683913568 | 2302.41452603605 | 1721.23260311381 | 1637.78160328582 | 1651.0164309748 | 1655.61188218084 |
| YDR385W | 26189.4074042608 | 0.556521097560902 | 0.134982554293669 | 4.12291129378195 | 3.74113797041263e-05 | 8.13716327166207e-05 | 22055.7172386228 | 23081.6440176999 | 18462.2600017888 | 30853.7781843763 | 32313.9732752155 | 30369.0717078615 |
| YDR386W | 149.78775967701 | -1.51441186629996 | 0.218769808624961 | -6.92239882558991 | 4.44058934257638e-12 | 1.94474425901083e-11 | 248.534392536947 | 216.031045432565 | 201.459945857831 | 84.0229975627088 | 68.4950636878049 | 80.1831129842017 |
| YDR387C | 1426.79040531491 | 0.527525779780136 | 0.161460378298451 | 3.2672150613015 | 0.0010861113845084 | 0.00195448620332262 | 1153.74681306142 | 1140.93962777883 | 1210.96544097755 | 1501.74436913667 | 1989.21080793333 | 1564.13537300168 |
| YDR388W | 3254.7571387001 | 0.1292176832112 | 0.235166798381051 | 0.549472476985559 | 0.582681247606381 | 0.641750818810492 | 2563.62845879853 | 2600.58665338214 | 4163.01537754394 | 3208.87828787107 | 3354.83114020895 | 3637.60291439596 |
| YDR389W | 461.848154157485 | -1.34871679938491 | 0.164299918954093 | -8.20887075277109 | 2.23278414914263e-16 | 1.4747289196883e-15 | 613.355519196685 | 651.378779574237 | 725.696958254304 | 278.742960168351 | 229.743859452846 | 272.170848298487 |
| YDR390C | 1067.8733371902 | -0.942513460636679 | 0.175785486736489 | -5.36172512381271 | 8.24309083334996e-08 | 2.39859520615109e-07 | 1402.28120559837 | 1320.82859716945 | 1492.5682120124 | 750.872184568334 | 599.331807268293 | 841.358016524369 |
| YDR391C | 1462.35580900294 | 0.351361151194932 | 0.209221245711188 | 1.67937605954204 | 0.0930787760543654 | 0.125380880890678 | 1417.48208587586 | 1289.61498604231 | 1147.73348716815 | 1423.05616507 | 2061.98681310163 | 1434.26131675966 |
| YDR392W | 331.821141997842 | -2.28011625163625 | 0.219230928629223 | -10.4005227086025 | 2.46576838353213e-25 | 3.29752035785349e-24 | 611.835431168936 | 600.451308787852 | 438.947400281479 | 120.032853661013 | 105.596556518699 | 114.063301569076 |
| YDR393W | 224.564454877648 | -2.23979185572228 | 0.483577764791946 | -4.63170976582416 | 3.62658306758717e-06 | 8.87958390953959e-06 | 502.389093171014 | 477.239685917568 | 132.345949833612 | 82.6892991886975 | 77.0569466487805 | 75.6657545062185 |
| YDR394W | 2748.47906386758 | -0.175113003616237 | 0.196391826743212 | -0.891651177750907 | 0.372579929789305 | 0.437860740100277 | 2591.75008731189 | 2556.23046914883 | 3597.60406964368 | 2536.6943073694 | 2350.23687278781 | 2858.35857694386 |
| YDR395W | 5292.95827298851 | 0.0617081169592493 | 0.115310899597993 | 0.535145568843724 | 0.59254920152938 | 0.650850824914404 | 5013.2503155159 | 5225.81563133833 | 5299.7200355593 | 5905.61640012182 | 5258.42311853252 | 5054.92413686319 |
| YDR396W | 44.3897596083216 | 0.961440313482393 | 0.334358332179451 | 2.87547885292832 | 0.00403415167878115 | 0.00673847741142789 | 34.2019806243504 | 22.1780921166512 | 33.8217427352563 | 64.01752195254 | 55.6522392463415 | 56.4669809747899 |
| YDR397C | 923.064593343816 | -0.93097503657549 | 0.219267254736077 | -4.24584618298827 | 2.17769992692444e-05 | 4.88232344487795e-05 | 1390.12050137638 | 1265.79407228739 | 977.88951821502 | 678.852472371726 | 510.859016671545 | 714.87197914084 |
| YDR398W | 1187.0063574846 | -4.06449173022595 | 0.301415164298004 | -13.4846955682942 | 1.92458238061422e-41 | 8.00182135939989e-40 | 2567.42867886791 | 2631.80026450927 | 1521.97842308653 | 149.37421788926 | 95.6076930642277 | 155.84886749042 |
| YDR399W | 5572.56000805395 | 0.0736273025994686 | 0.160353914587285 | 0.459155005906584 | 0.646122858672393 | 0.700794792867749 | 5609.88486640735 | 5801.62461555212 | 4879.889272476 | 6584.46887249354 | 5095.74734227399 | 5463.74507912067 |
| YDR400W | 1971.11526799003 | 1.21714854879852 | 0.12199975252436 | 9.97664768668678 | 1.92873224639424e-23 | 2.20630641095468e-22 | 1089.14307188209 | 1177.08170382078 | 1290.37301087771 | 2788.76330005752 | 2823.99439662846 | 2657.33612467361 |
| YDR401W | 123.878186979467 | 1.59747698228112 | 0.218513475837969 | 7.31065659065199 | 2.65840144827274e-13 | 1.29642043560128e-12 | 58.523389068333 | 59.1415789777365 | 66.9082301936593 | 162.711201629373 | 198.350288595935 | 197.634433411765 |
| YDR402C | 27.3486847966126 | -0.469495575740208 | 0.398452566545873 | -1.17829728092906 | 0.238678111424443 | 0.295658180041814 | 28.1216285133548 | 32.0350219462739 | 35.2922532889631 | 26.673967480225 | 17.1237659219512 | 24.8454716289076 |
| YDR403W | 138.421157458587 | 0.0674764759788044 | 0.300892158508615 | 0.224254684180719 | 0.822559134990802 | 0.855398196176101 | 159.609242913635 | 158.532288093099 | 87.4953779455544 | 138.70463089717 | 148.405971323577 | 137.779433578487 |
| YDR404C | 3756.64085329897 | 0.423054201215502 | 0.177001746834923 | 2.39011314170846 | 0.0168431831576354 | 0.0258201101300929 | 3336.59322090885 | 3713.59831331037 | 2579.27551120172 | 4235.82603585973 | 4483.57271056423 | 4190.97932794891 |
| YDR405W | 235.764145561556 | -1.01086560416251 | 0.38276945987791 | -2.64092543978023 | 0.00826799127613388 | 0.0132410349177789 | 386.102359048223 | 400.848479737992 | 158.815139800334 | 157.376408133328 | 134.136166388618 | 177.30632026084 |
| YDR406W | 729.558599616342 | 1.06132388796531 | 0.273323067607707 | 3.88303811037493 | 0.000103159392068756 | 0.000212140715585907 | 380.782050951102 | 400.027068918857 | 636.731069755043 | 905.581195953639 | 1207.22549749756 | 847.004714621848 |
| YDR406W-A | 38.8936948420139 | 2.24726757614396 | 0.382442727936477 | 5.87608918142961 | 4.20072079422707e-09 | 1.38444487151203e-08 | 10.6406161942424 | 11.4997514678932 | 18.381381921335 | 64.01752195254 | 59.9331807268293 | 68.8897167892437 |
| YDR407C | 682.139170813412 | -1.73967862792673 | 0.139460958544099 | -12.4743056844589 | 1.03100956956222e-35 | 2.88238278800887e-34 | 1040.50025499413 | 1070.2982973332 | 1040.38621674756 | 316.086514640666 | 281.115157218699 | 344.448583946218 |
| YDR408C | 1681.21555631102 | 1.18664527250182 | 0.214313029422583 | 5.53697213696696 | 3.07745486819264e-08 | 9.26235372394314e-08 | 895.331848344107 | 882.195219751236 | 1301.40184003052 | 2263.28614069709 | 2120.49301333496 | 2624.58527570823 |
| YDR409W | 235.959551025218 | -1.0238765720739 | 0.252251545581291 | -4.05895063879378 | 4.92937311566262e-05 | 0.000105867264993999 | 313.138133716275 | 257.101586389327 | 379.391722856354 | 162.711201629373 | 122.72032244065 | 180.694339119328 |
| YDR410C | 1687.38261330384 | 1.07102074953777 | 0.143435053981196 | 7.46693865837134 | 8.20820079158127e-14 | 4.22191834529708e-13 | 1140.06602081168 | 1187.76004446954 | 937.450477988083 | 2236.61217321687 | 2344.52895081382 | 2277.87801252302 |
| YDR411C | 2346.01817048234 | 1.18180489448621 | 0.139595586368615 | 8.46591876741396 | 2.54141255768835e-17 | 1.82340728419985e-16 | 1327.79689223867 | 1367.64901386016 | 1610.9443115858 | 3032.83010250158 | 3299.1789009626 | 3437.70980174521 |
| YDR412W | 402.372257909459 | -4.41845786398525 | 0.554996817054928 | -7.96123099846166 | 1.70336043798346e-15 | 1.03038634207109e-14 | 1030.61968281376 | 1038.26327538693 | 237.487454423648 | 22.6728723581913 | 45.6633757918699 | 39.5268866823529 |
| YDR413C | 4.25045171011048 | -1.24227484835208 | 1.07614043036161 | -1.15437986837335 | 0.248344465006749 | 0.306053999626406 | 7.60044013874454 | 8.21410819135229 | 2.20576583056019 | 2.6673967480225 | 1.42698049349594 | 3.38801885848739 |
| YDR414C | 1082.73799208026 | 0.578240584172048 | 0.154457863893135 | 3.74367849973711 | 0.00018134566585223 | 0.000363813173126373 | 770.684630068697 | 858.374305996314 | 976.419007661313 | 1408.38548295588 | 1262.8777367439 | 1219.68678905546 |
| YDR415C | 1133.16788620841 | 0.833764809131673 | 0.13876309864271 | 6.00854850667805 | 1.87191613079373e-09 | 6.42054364057543e-09 | 764.604277957701 | 762.269240157493 | 916.128074959334 | 1465.73451303836 | 1501.18347915772 | 1389.08773197983 |
| YDR416W | 666.882679486547 | -0.830018570054751 | 0.207166239050941 | -4.00653395001613 | 6.16162722363415e-05 | 0.000130431834766616 | 957.655457481812 | 952.836550196866 | 650.700920015258 | 477.464017896028 | 462.341679892683 | 500.297451436638 |
| YDR417C | 21.4804809364342 | 0.61955741292884 | 0.464631636600476 | 1.33343785511872 | 0.182388156589071 | 0.232639052829245 | 23.5613644301081 | 14.7853947444341 | 12.4993397065078 | 26.673967480225 | 24.2586683894309 | 27.1041508678992 |
| YDR418W | 88966.5150620865 | 0.843586079968935 | 0.179833379891697 | 4.69093157497777 | 2.71963919710364e-06 | 6.77923898248047e-06 | 70370.1951125941 | 70587.1173315668 | 50060.5907798405 | 113061.612260056 | 111030.498237932 | 118689.07665053 |
| YDR419W | 320.865232413695 | -1.3323221634306 | 0.182156446955749 | -7.314164201689 | 2.58988293034265e-13 | 1.26490818420199e-12 | 501.62904915714 | 444.383253152159 | 433.065358066652 | 173.380788621463 | 162.675776258537 | 210.057169226218 |
| YDR420W | 1076.70112874031 | -2.27143597754158 | 0.13863252999161 | -16.3845814375533 | 2.46459647265705e-60 | 2.75609874511269e-58 | 1737.460615717 | 1787.38994243826 | 1828.5798735344 | 373.43554472315 | 326.778533010569 | 406.562263018487 |
| YDR421W | 277.274347540878 | -3.25975584283354 | 0.234165482486122 | -13.9207359181417 | 4.73998322030887e-44 | 2.29429337066592e-42 | 513.789753379131 | 512.560351140383 | 478.651185231562 | 60.0164268305063 | 61.3601612203252 | 37.2682074433613 |
| YDR422C | 687.462440493406 | -0.904077979754914 | 0.167252960538404 | -5.40545277551199 | 6.46448820269879e-08 | 1.89894340954277e-07 | 836.808459275774 | 814.839532582147 | 1036.70994036329 | 508.139080498286 | 443.790933477236 | 484.486696763697 |
| YDR423C | 299.616731048207 | -0.666242856406102 | 0.228818227486218 | -2.91166863639056 | 0.00359503790274167 | 0.00605332706053542 | 366.341214687487 | 317.885987005334 | 418.360252529584 | 180.049280491519 | 271.126293764228 | 243.937357811092 |
| YDR424C | 1645.99067494067 | 1.27495416245986 | 0.127279847106019 | 10.016936627822 | 1.28421732948666e-23 | 1.4873988569733e-22 | 1030.61968281376 | 905.194722687022 | 951.420328248297 | 2504.68554639313 | 2314.56236045041 | 2169.46140905143 |
| YDR425W | 160.196763214671 | -1.02826221232461 | 0.220081470534849 | -4.67218893905831 | 2.98006631548499e-06 | 7.38865065834696e-06 | 243.9741284537 | 190.567310039373 | 210.283009180072 | 121.366552035024 | 95.6076930642277 | 99.3818865156302 |
| YDR426C | 7.43648882065022 | 0.996462029194435 | 0.84078114545033 | 1.18516219659127 | 0.235953323519263 | 0.292730156148803 | 6.08035211099563 | 7.39269737221706 | 1.4705105537068 | 6.66849187005625 | 12.8428244414634 | 10.1640565754622 |
| YDR427W | 3079.31415625085 | 0.15067499712266 | 0.17201129311491 | 0.87595991166698 | 0.381051827534555 | 0.446119522272405 | 2625.95206793624 | 2660.54964317901 | 3469.66965147119 | 3354.25141063829 | 2942.43377758862 | 3423.02838669176 |
| YDR428C | 1167.00755915072 | 0.030876398587579 | 0.275915205651793 | 0.111905389609246 | 0.910898423158756 | 0.928798486497044 | 1337.67746441904 | 1398.04121416816 | 727.902724084864 | 1137.6447130316 | 1271.43961970488 | 1129.3396194958 |
| YDR429C | 3835.53383969388 | -1.11003204834708 | 0.169958089868088 | -6.53121042492671 | 6.5240261038126e-11 | 2.55678751113768e-10 | 5947.3444085676 | 5468.13182298322 | 4312.27219874518 | 2458.00610330273 | 2271.75294564553 | 2555.69555891899 |
| YDR430C | 1044.09184053236 | 0.456525951985786 | 0.250061081381043 | 1.82565775315564 | 0.0679018305658675 | 0.0937643757824605 | 691.640052625753 | 746.662434593923 | 1202.14237765531 | 1200.32853661013 | 1257.16981476992 | 1166.60782693916 |
| YDR431W | 7.1944789679376 | 0.867285692015684 | 0.708735062254768 | 1.22370930719373 | 0.221061907374024 | 0.276050737625706 | 5.32030809712118 | 4.10705409567614 | 5.88204221482719 | 8.0021902440675 | 8.56188296097562 | 11.293396194958 |
| YDR432W | 1987.16530708908 | -1.84850186581313 | 0.211282451605596 | -8.74896070054959 | 2.15326629800386e-18 | 1.69080934731877e-17 | 2603.91079153388 | 2853.58118567579 | 3874.79530901741 | 765.542866682458 | 856.188296097562 | 968.973393527395 |
| YDR433W | 115.890504881906 | -0.59087678162417 | 0.269308934298508 | -2.19404819659354 | 0.028231944852168 | 0.0417397753159702 | 161.889374955259 | 149.496769082612 | 106.612015143743 | 105.362171546889 | 82.7648686227643 | 89.217829940168 |
| YDR434W | 1924.01981192375 | -0.234591065610451 | 0.17315183578632 | -1.35482863664207 | 0.175472160554222 | 0.224524282678103 | 2134.96363497334 | 2267.09386081323 | 1837.40293685664 | 1879.18100898185 | 1939.26649066098 | 1486.21093925647 |
| YDR435C | 1018.20596946327 | 1.36097302509641 | 0.201409751876959 | 6.75723500184748 | 1.40649965066393e-11 | 5.85529957266125e-11 | 494.028609018395 | 530.631389161358 | 686.728428581074 | 1371.04192848357 | 1700.96074824716 | 1325.84471328807 |
| YDR436W | 591.410100367951 | -0.596003724744511 | 0.26897268483918 | -2.21585223458979 | 0.026701623728922 | 0.0396127016252946 | 547.991734003481 | 614.415292713151 | 972.742731277046 | 437.45306667569 | 536.544665554472 | 439.313111983865 |
| YDR437W | 120.067958704338 | 0.478693116318851 | 0.205954821396933 | 2.32426273428324 | 0.0201114168116919 | 0.0304274899558277 | 89.6851936371856 | 101.033530753633 | 110.28829152801 | 134.703535775136 | 141.271068856098 | 143.426131675966 |
| YDR438W | 631.443955401188 | 0.236730206150261 | 0.182039441432766 | 1.30043359992233 | 0.193452400236069 | 0.244825808376808 | 623.236091377053 | 644.807493021155 | 471.298632463028 | 648.177409769468 | 729.187032176423 | 671.9570736 |
| YDR439W | 188.079807057603 | -3.19576029468993 | 0.498758899497657 | -6.40742510641646 | 1.47997744699729e-10 | 5.61352849194411e-10 | 428.664823825192 | 462.454291173134 | 126.463907618785 | 32.00876097627 | 37.1014928308943 | 41.7855659213445 |
| YDR440W | 248.676190676189 | -2.22788017795311 | 0.190781309572747 | -11.677664771997 | 1.65787713307769e-31 | 3.34984145954577e-30 | 424.104559741945 | 436.169144960807 | 369.833404257259 | 84.0229975627088 | 84.1918491162602 | 93.7351884181512 |
| YDR441C | 2634.39679581765 | 0.763992186679843 | 0.209260037395625 | 3.65092253728048 | 0.000261300046978041 | 0.000514196633707395 | 2213.24816840241 | 2207.13087101636 | 1438.15932152525 | 3340.91442689818 | 3212.13309085935 | 3394.79489620437 |
| YDR443C | 509.446232079279 | 0.00468510392117623 | 0.145448977802858 | 0.0322113224303745 | 0.974303526871825 | 0.98019740581521 | 472.74737662991 | 492.025080662002 | 560.999776239143 | 481.465113018061 | 526.5558021 | 522.884243826554 |
| YDR444W | 376.918569074287 | -1.55995559561936 | 0.182320779444725 | -8.5561042486235 | 1.16745733694914e-17 | 8.63412575536159e-17 | 554.072086114477 | 595.522843873041 | 540.412628487248 | 201.388454475699 | 155.540873791057 | 214.574527704202 |
| YDR445C | 2.58333899372961 | 2.51053051752013 | 1.43539575288991 | 1.74901626430595 | 0.0802882080542418 | 0.109677615299034 | 0 | 0.821410819135229 | 1.4705105537068 | 6.66849187005625 | 4.28094148048781 | 2.2586792389916 |
| YDR446W | 0.994519765622902 | -1.7393260536945 | 2.02093855110059 | -0.860652617442164 | 0.389429396913372 | 0.454696501958619 | 1.52008802774891 | 1.64282163827046 | 1.4705105537068 | 1.33369837401125 | 0 | 0 |
| YDR447C | 43481.8367970496 | 0.548501061248021 | 0.284851106217181 | 1.9255711116313 | 0.0541579487271141 | 0.0759007034235224 | 43253.3447855813 | 41116.539962633 | 21573.1250781556 | 57206.3243564646 | 47763.891078296 | 49977.795521167 |
| YDR448W | 207.623231454775 | -1.17865724731754 | 0.212527962687281 | -5.54589256121487 | 2.92458375733924e-08 | 8.8186193631345e-08 | 311.618045688526 | 269.422748676355 | 283.808536865412 | 138.70463089717 | 99.8886345447155 | 142.296792056471 |
[truncated: 1,198,248 more chars]
